# S,O‐Ligand Promoted *meta*‐C−H Arylation of Anisole Derivatives via Palladium/Norbornene Catalysis

**DOI:** 10.1002/anie.202201750

**Published:** 2022-06-21

**Authors:** Verena Sukowski, Manuela van Borselen, Simon Mathew, M. Ángeles Fernández‐Ibáñez

**Affiliations:** ^1^ Van't Hoff Institute for Molecular Sciences University of Amsterdam Science Park 904 1098 XH Amsterdam The Netherlands

**Keywords:** Arylation, C−H Activation, Ligand Design, Palladium, Reaction Mechanisms

## Abstract

Reversing the conventional site‐selectivity of C−H activation processes provides new retrosynthetic disconnections to otherwise unreactive bonds. Here, we report a new catalytic system based on palladium/norbornene and an S,O‐ligand for the *meta*‐C−H arylation of aryl ethers that significantly outperforms previously reported systems. We demonstrate the unique ability of this system to employ alkoxyarene substrates bearing electron donating and withdrawing substituents. Additionally, *ortho*‐substituted aryl ethers are well tolerated, overcoming the “*ortho* constraint”, which is the necessity to have a *meta*‐substituent on the alkoxyarene to achieve high reaction efficiency, by enlisting novel norbornene mediators. Remarkably, for the first time the monoarylation of alkoxyarenes is achieved efficiently enabling the subsequent introduction of a second, different aryl coupling partner to rapidly furnish unsymmetrical terphenyls. Further insight into the reaction mechanism was achieved by isolation and characterization of some Pd‐complexes—before and after *meta* C−H activation—prior to evaluation of their respective catalytic activities.

## Graphical abstract

A highly efficient *meta*‐selective C−H arylation of aryl ethers has been realized by a new catalytic system based on palladium/norbornene (Pd/NBE) and an S,O‐ligand. The reaction proceeds with a broad range of aryl ethers. For the first time, unsymmetrical *meta*‐diarylation of aryl ethers was accomplished using a novel norbornene. The isolation of palladium intermediates provides insight to the mechanism.

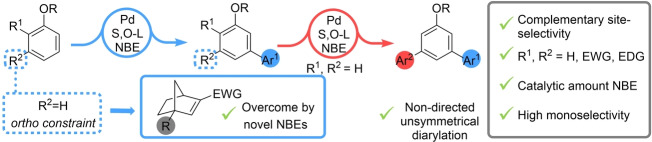

## Introduction

**Scheme 1:**
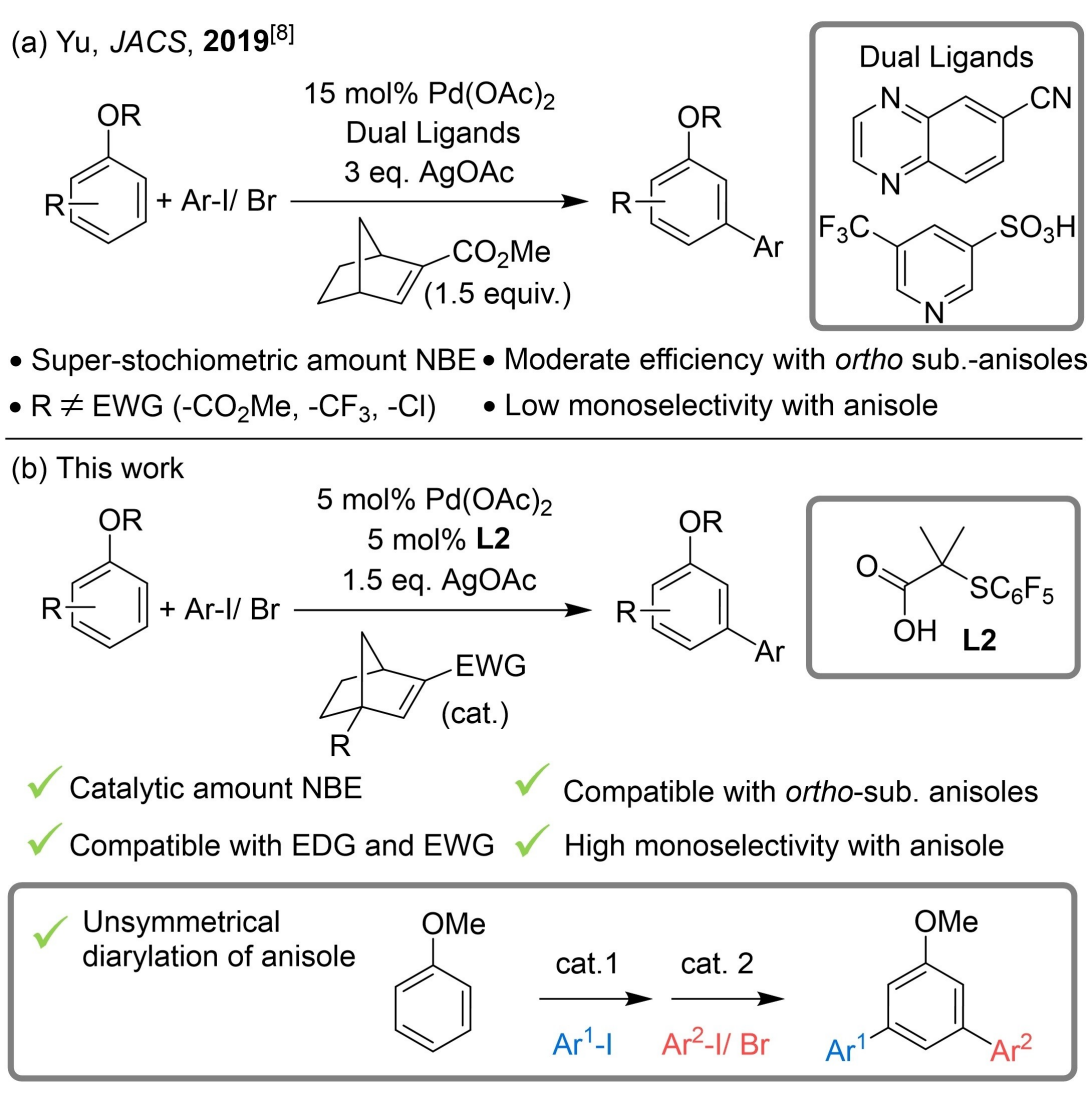
Direct *meta*‐C−H arylation of aryl ethers.

Controlling the site‐selectivity in C−H functionalization reactions is a major challenge given that the C−H bond is ubiquitous within organic molecules. In the last two decades, efficient and site‐selective metal‐catalyzed C−H functionalization reactions have been accomplished using directing groups (DGs).^[1]^ In recent years, alternative catalytic systems have been developed for the non‐directed palladium‐catalyzed C−H functionalization of arenes,^[2]^ greatly broadening the substrate scope beyond those bearing DGs. In the particular case of Pd‐catalyzed C−H arylation reactions, in most of the cases, an excess of the arene is needed, hampering the applicability of these methodologies to substrates of increasing complexity.[^3a^, ^3b^, ^3c^, ^3d^, ^3e^, ^3f^, ^3g^, ^3h^, ^3i^] Arene‐limited, non‐directed Pd‐catalyzed C−H arylation reactions remain challenging, with only few examples reported.[^3j^, ^3k^, ^3l^, ^3m^] Similar trend is also observed for other transition metals.[^3n^, ^3o^, ^3p^] Achieving selectivity in non‐directed C−H functionalization reactions remains equally challenging. The majority of examples invoke site‐selectivity that is predominantly controlled via electronic and steric effects, with functionalization occurring at the most electron rich or sterically accessible positions of the arene. Consequently, realizing complementary site‐selectivity—favoring the functionalization at electron‐deficient position—is a major challenge in the field. Beyond examples that use templates or traceless DGs to achieve reverse site‐selectivity,^[4]^ only few methodologies using non‐directed arenes have been reported, albeit with low levels of site‐selectivity.^[3g]^ A more general approach for reversing conventional site‐selectivity has been enabled using palladium/norbornene (Pd/NBE) cooperative catalysis, known as the Catellani‐type reaction.[^5^, ^6^] Although the first reports of C−H activation using the Pd/NBE strategy focused on substrates bearing DGs,[^5e^, ^7^] in 2019 the *meta*‐arylation of electron‐rich alkoxyarenes was achieved by Yu and co‐workers (Scheme 1a).^[8]^ In the same year, the group of Dong reported the direct vicinal difunctionalization of thiophenes using the Pd/NBE strategy.^[9]^ Following a similar approach, the functionalization of fluoroarenes, simple arenes^[10]^ and five‐membered heteroarenes^[11]^ at the less reactive (i.e. electron‐deficient) site was achieved. Although these examples represent the current state‐of‐the‐art for the palladium‐catalyzed C−H functionalization of non‐directed arenes for non‐conventional site‐selectivity, several drawbacks persist. In the particular case of electron rich alkoxyarenes, the main limitations of the methodology are: i) the use of super‐stochiometric amounts of NBE, ii) the lack of reactivity for substrates bearing electron‐withdrawing substituents, iii) the relatively low reactivity observed for aryl ethers bearing small *ortho*‐substituents and iv) the impossibility to control the monoarylation of unsubstituted aryl ethers.^[8]^ The last two limitations are a consequence of the so‐called “*ortho* constraint”, which is the necessity to have an *ortho*‐substituent next to the first activated C−H bond to promote the NBE extrusion from the Pd‐complex formed after the *meta*‐C−H functionalization.[^5e^, ^12^, ^13^] Therefore, the development of a new catalytic system that overcomes the limitations for non‐directed *meta*‐C−H arylation of alkoxyarenes will be of great importance, given the ubiquity of this motif in natural products and pharmaceuticals.

Recently, our group has disclosed a new catalytic system based on Pd/S,O‐ligand, capable of promoting Pd‐catalyzed C−H functionalization reactions on a wide variety of arenes including simple arenes, thiophenes, anilines and anisoles.[^2e^, ^14^] A unique feature of the Pd/S,O‐ligand system is it's high catalytic activity, allowing for the functionalization of aniline and anisole derivatives bearing several electron withdrawing substituents, substrates that are unreactive using other catalytic systems. Additionally, in 2022 the group of Jiao introduced unstrained olefin ligands that bear a S coordination site for use in the Pd‐catalysed Catellani reaction.^[15]^ We hypothesized that by using our Pd/S,O‐ligand catalytic system in conjunction with the appropriate choice of the NBE mediator[^13^, ^16^] could overcome the previously mentioned limitations for the *meta*‐arylation of alkoxyarenes. Herein, we report a general and efficient C−H arylation of alkoxyarenes with non‐conventional site‐selectivity, promoted by Pd/NBE catalysis using the Pd/S,O‐ligand catalytic system (Scheme 1b). The reaction proceeds using catalytic amounts of NBE on a broad range of alkoxyarene derivatives bearing both electron donating and withdrawing substituents. *Ortho*‐substituted alkoxyarenes are efficiently arylated by overcoming the *ortho* constraint through simple selection of the appropriate NBE mediator. Remarkably, the monoarylation of alkoxyarenes is efficiently achieved allowing, for the first time, the introduction of two different aryl coupling partners.

## Results and Discussion

Initially, we applied the conditions reported for the *meta*‐arylation of anisole derivatives^[8]^ using anisole and methyl 4‐iodobenzoate as model substrates in the presence of 15 mol % of Pd(OAc)_2_ and the S,O‐ligand **L1**, previously used for the C−H olefination of anisoles,^[14e]^ and 1.5 equiv of NBE **N1** (Table 1). Under these conditions, we observed the formation of the *meta*‐monoarylated product **3** **a** in 20 % ^1^H‐NMR yield. Next, modified norbornenes **N2** (NBE‐CO_2_Me) and **N3** (NBE‐CONHMe), pioneered by the group of Yu^[7b]^ and Dong,^[17]^ respectively were evaluated in the reaction under previously mentioned conditions. The reaction using **N2** and **N3** afforded a mixture of mono‐ and diarylated products **3** **a** in 39 % and 36 % ^1^H‐NMR yield, respectively (Table 1a).

**Table 1:**
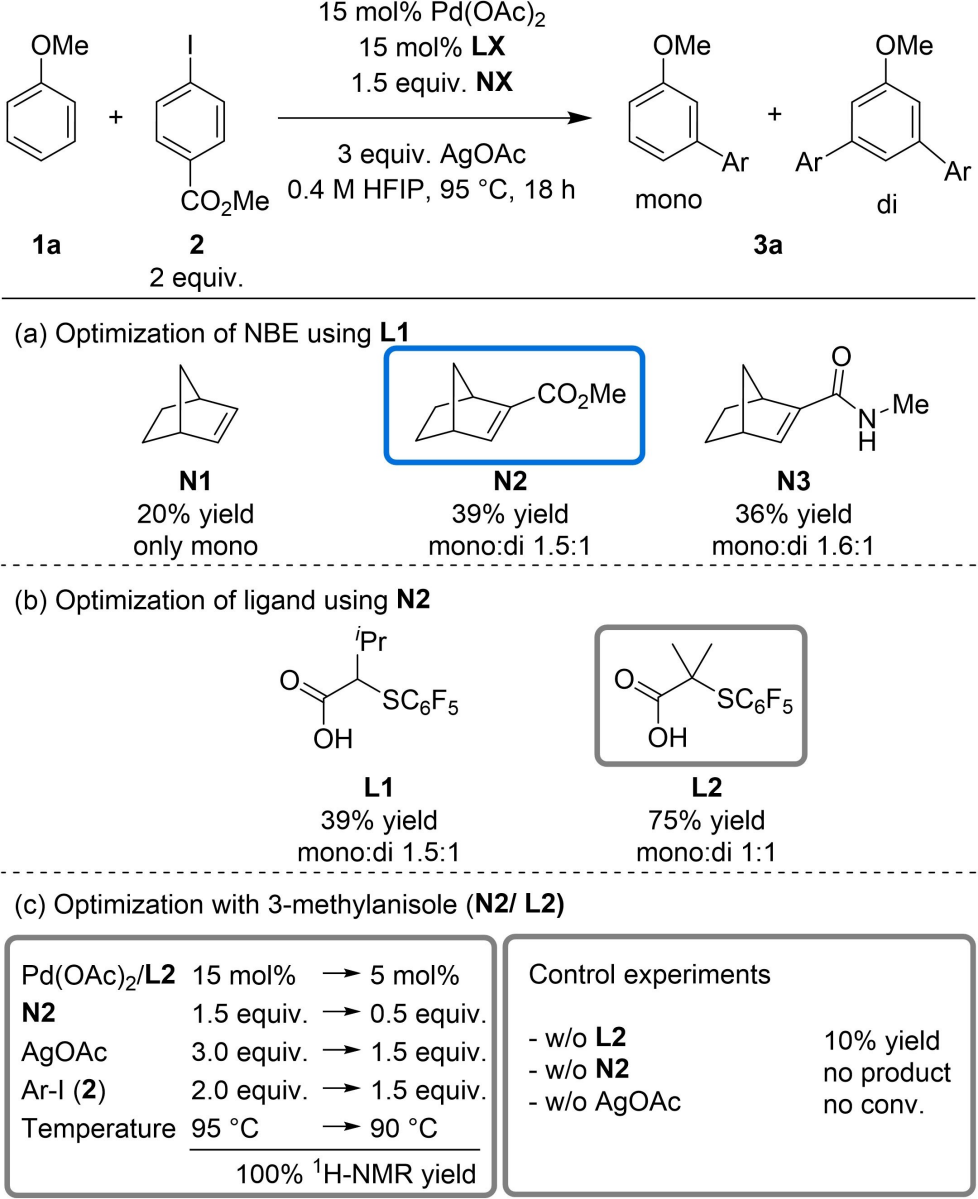
Selected optimization for *meta*‐C−H arylation.

Following this, we evaluated the influence of the S,O‐ligand in the reaction using **N2** as a mediator (Supporting Information, Table S4). To our delight, the reaction using a slightly modified S,O‐ligand **L2**, bearing a *gem*‐dimethyl group in place of an isopropyl group, furnished **3** **a** in 75 % ^1^H‐NMR yield and a 1 : 1 ratio of mono‐, and diarylated products (Table 1b). The same reactions conditions employing 3‐methyl anisole as a substrate provided the *meta*‐arylated product **3** **b** in quantitative yield. Encouraged by this result, we performed an exhaustive optimization of reaction conditions (Supporting Information, Table S5) enabling reductions in catalyst loading to 5 mol %, the use catalytic amounts of **N2**, decreased amounts of AgOAc and aryl iodide, while maintaining the quantitative yield for **3** **b** (Table 1c). Additionally, further control experiments were performed to enable greater insight into the role of each reagent within the catalytic system. As expected, the reaction without the S,O‐ligand dramatically reduced the yield of **3** **b** to 10 %, highlighting the key role of this component in this transformation. Interestingly, the reaction without **N2** did not provide **3** **b** and only trace amounts of arylated anisole were detected, highlighting the crucial role of NBE not only to reverse the site‐selectivity but also to enable the C−H arylation reaction. Moreover, no product was observed in the absence of AgOAc.^[18]^

With the optimized conditions in hand, the scope of *meta*‐substituted anisole derivatives was evaluated (Table 2). Anisole derivatives with a OMe‐, OCF_3_‐, TMS‐, and Ph‐ groups at the *meta*‐position provided the desired *meta*‐arylated products **3** **c**–**3** **f** with excellent isolated yields (75–82 %) and perfect regioselectivity. Then, we moved our attention to anisoles bearing electron‐withdrawing substituents, as they were demonstrated to be unreactive substrates in the previously reported methodology.^[8]^ To our delight, the reaction with 3‐fluoroanisole (**1** **g**) provided **3** **g** in 79 % yield. When the reaction was performed with 3‐chloroanisole (**1** **h**), only 18 % yield was obtained. However, upon substitution of **N2** with 30 mol % NBE‐CONHMe (**N3**), the yield improved to 73 %. Likewise, 3‐substituted anisoles with CF_3_‐ and CO_2_Me‐groups **1** **i**–**1** **j** revealed low reactivity under standard reaction conditions. Nevertheless, after optimization, the *meta*‐arylated products **3** **i** and **3** **j** were obtained in 52 % yield. In both cases, a higher amount of anisole substrate was needed to obtain these results (3.6 equiv of **1** **i** and 2 equiv of **1** **j**). Anisoles bearing other electron‐withdrawing groups, such as NO_2_, were unreactive under the standard conditions (Supporting Information, Table S9). After demonstrating the generality of the methodology with *meta*‐substituted anisoles, we studied the reaction using disubstituted anisoles. 2,3‐Dimethoxy‐, difluoro‐, dichloro‐ and 3‐chloro‐2‐methylanisoles **1** **k**–**1** **n** were *meta*‐arylated in good yields (57–88 %). Similar to the reaction with 3‐chloroanisole (**1** **h**), the use of 30 mol % **N3** provided the best result for the arylation of 2,3‐dichloroanisole (**1** **m**). The reaction with 5‐methoxytetralin (**1** **o**) and 7‐methoxy‐1‐indanone (**1** **p**) furnished the arylated products **3** **o**–**3** **p** in synthetically useful yields (42–64 %). Additionally, we evaluated other successfully reported ligands for C−H functionalization reactions under the optimized reaction conditions utilizing 3‐methylanisole (**1** **b**) and methyl 3‐methoxybenzoate (**1** **j**) as substrates (Supporting Information, Tables S6 and S7). From all the ligands tested, the S,O‐ligand **L2** significantly outperformed the others, confirming the superiority of the Pd/S,O‐ligand catalyst.

**Table 2:**
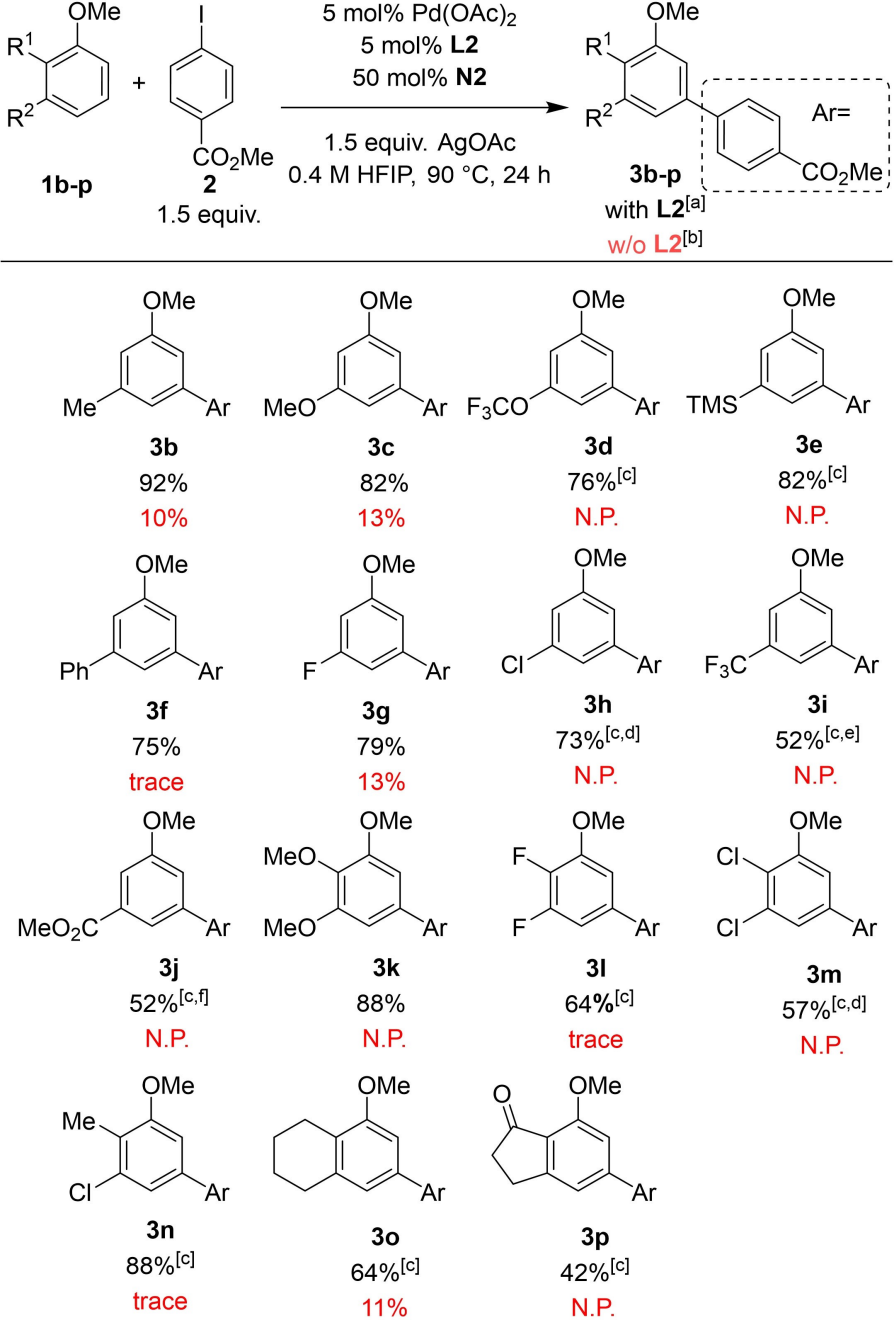
Scope of *meta*‐substituted anisoles.

[a] Isolated yield. [b] ^1^H‐NMR yield of the crude mixture using CH_2_Br_2_ as internal standard. [c] 10 mol % Pd(OAc)_2_/**L2** was used. [d] 30 mol % NBE **N3** instead of NBE **N2** was used. [e] 3.6 equiv anisole **1** **i**, 1.0 equiv aryl iodide **2**, 20 mol %. **N2**, 2 equiv AgOAc, 0.2 M HFIP, 70 °C, 48 h. [f] 2.0 equiv anisole **1** **j**, 1.0 equiv aryl iodide **2**, 48 h. N.P.: no product. w/o: without.

Next, we decided to explore the generality of the reaction with respect to the aryl halide employed, using 3‐methylanisole (**1** **b**) as model substrate (Table 3). The starting point was iodobenzene affording the product **4** **a** in a 75 % isolated yield. Electron‐withdrawing substituents at the *para*‐position of the aryl iodide, including F‐, Br‐, Ac‐ and NO_2_‐, were well tolerated, affording the arylated products **4** **b**–**4** **e** in 75–91 % isolated yields. A slightly lower yield of 64 % was obtained when using *p*‐tolyl iodide and a moderate yield of 39 % was obtained when using 4‐iodoanisole. Aryl iodides featuring a *meta*‐chloro or trifluoromethyl group were also suitable coupling partners, affording the desired products in 83 % and 90 % isolated yields, respectively. Further evaluation of the reaction with the thiophene iodide derivative **2** **j** afforded **4** **j** in 47 % isolated yield. The *ortho*‐substituted aryl bromides with coordinating functional groups that facilitate the oxidative addition (i.e., ester, nitro and amide)[^5d^, ^7c^, ^9^] were also compatible, providing the desired products **4** **k**–**4** **m** in excellent yields. It is worth mentioning, that we also performed the reactions outlined in Table 2 and 3 without S,O‐ligand, and in the vast majority of cases low yields or no product formation were observed, highlighting the key role of the S,O‐ligand in the reaction.

**Table 3:**
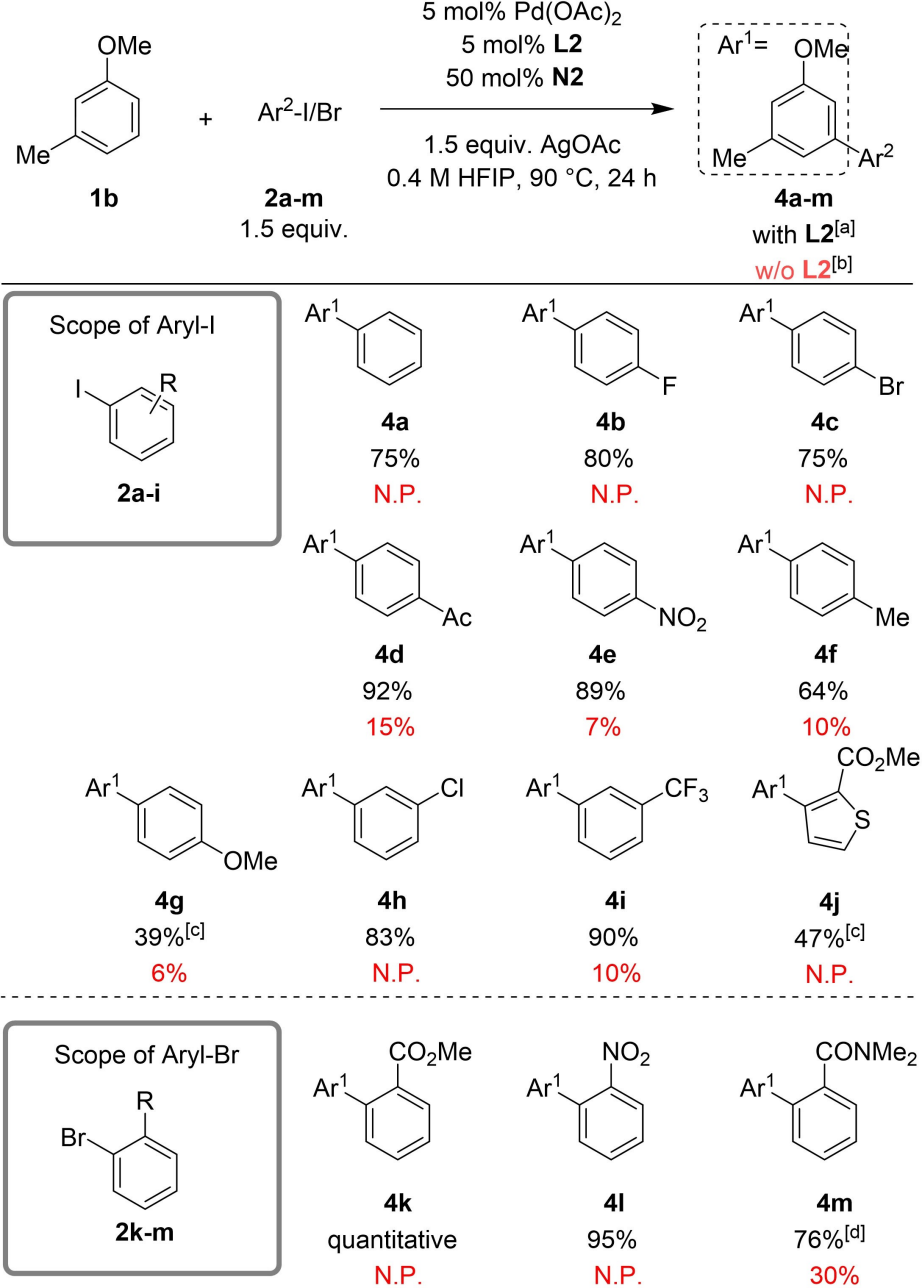
Scope of aryl halides.

[a] Isolated yield. [b] ^1^H‐NMR yield of the crude mixture using CH_2_Br_2_ as internal standard. [c] 10 mol % Pd(OAc)_2_/**L2** was used. [d] 1.0 equiv of aryl‐Br **2** **m** and 1.5 equiv of 2‐methylanisole **1** **b** were used. N.P.: no product. w/o: without.

Further evaluation of the reaction was performed with *ortho*‐substituted anisoles (Table 4). Expectedly, the reaction with 2‐methylanisole (**5** **a**) under the standard conditions, afforded the desired product in only 12 % ^1^H‐NMR yield and 24 % yield using 10 mol % of catalyst. The lack of reactivity against *ortho*‐substituted anisoles is the consequence of the *ortho* constraint (see above).[^5e^, ^12^, ^13^] Therefore, we decided to explore the effect of NBE modification with the goal of promoting β‐carbon elimination. Modification of NBEs **N2** and **N3** to increase steric bulk did not improve yields, as demonstrated by the performance of the *tert*‐butyl ester **N4**, tertiary acyclic amide **N6**, or parent carboxylic acid **N5**. In 2018, the group of Dong overcame the *ortho* constraint in the Catellani reaction by using bridgehead‐modified NBEs, which facilitates the β‐carbon elimination.^[13]^ Inspired by this work, several NBEs with substituents at the bridgehead position were evaluated. To our delight, NBE **N7**, featuring a hexyl group at the bridgehead position, dramatically improved the yield to 77 %. However, increasing the steric hindrance at the bridgehead with NBE **N8** (cyclohexyl) only afforded a 39 % yield. Next, we tested NBE **N9** bearing a hexyl group at the bridgehead position and an amide. Under the standard conditions, 98 % ^1^H‐NMR yield was obtained. Further optimization of the reaction conditions allowed to reduce the amount of **N9** to 20 mol % while retaining the high yield. The effectiveness of amide‐substituted NBEs has been attributed to facilitate the migratory insertion and *ortho*‐palladation steps.^[17]^ Additionally, the use of mixture of HFIP and DCE in a ratio 1.5 : 1, using **N7** provided **6** **a** in 80 % isolated yield as a mixture of regioisomers 11 : 1 in favor of the *meta*‐product.

**Table 4:**
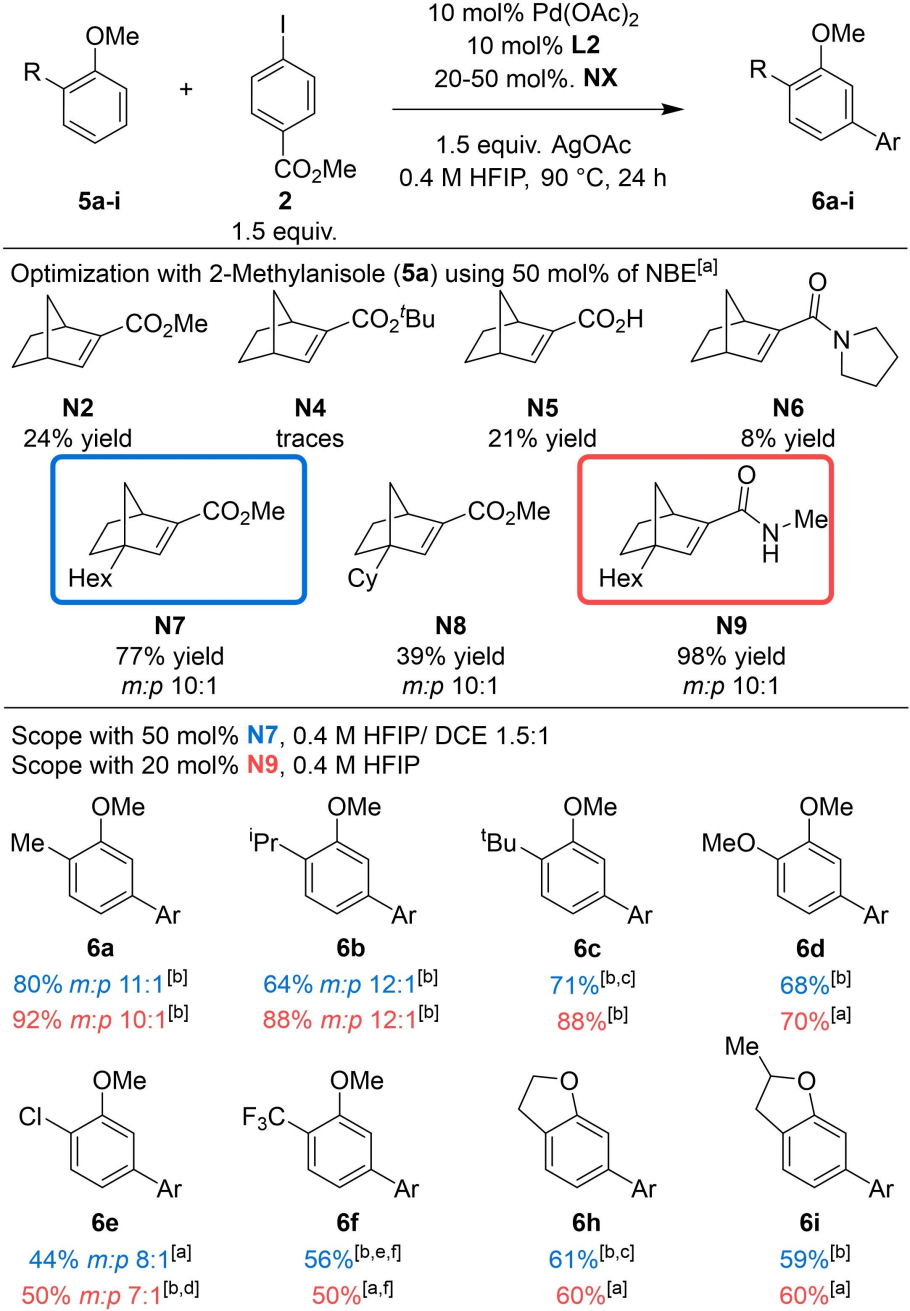
Scope of *ortho*‐substituted anisoles.

[a] ^1^H‐NMR yield of the crude mixture using CH_2_Br_2_ as internal standard. [b] Isolated yield. [c] 2.0 equiv of Ar−I **2** was used. [d] 48 h. [e] HFIP (0.4 M) was used as sole solvent. [f] 3.0 equiv anisole **5** **f**, 1.0 equiv Ar−I **2**, 40 h.

With the optimized reaction conditions in hand, the scope of the reaction was investigated with both **N7** and **N9**. The *ortho*‐substituted anisoles with an isopropyl, *tert*‐butyl and methoxy groups **5** **b**–**5** **d** were arylated in high yields (64–71 %) with **N7**. When using 20 mol % of NBE **N9**, higher yields (88–92 %) were obtained for substrates **5** **a**–**5** **c**. Similarly to the reaction of 2‐methylanisole, 2‐isopropyl anisole provided a mixture of regioisomers (*meta*:*para* in a 12 : 1 ratio) for both **N7** and **N9**. Electron withdrawing substituents, Cl‐ and CF_3_‐, were well tolerated. For substrate **5** **e**, NBE **N9** provided higher yield (50 %) than when using **N7**. Longer reaction times and 3 equiv of 2‐trifluoromethylanisole (**5** **f**) were needed to obtain the arylated product **6** **f** in synthetical useful yields. For this substrate, **N7** provided slightly higher yield than **N9**. 2,3‐Dihydrobenzofuran (**5** **h**) and 2,3‐dihydro‐2‐mehtylbenzofuran (**5** **i**) were successfully *meta*‐arylated in good yields (59–61 %) with perfect regioselectivity using **N7**. Similar results were observed using **N9**.

Encouraged by our promising results with *ortho*‐substituted anisoles, we concentrated our efforts to find a suitable catalytic system for the selective monoarylation of anisole (Table 5). The reaction of anisole employing our previously optimized conditions for *ortho*‐substituted anisoles in the presence of **N2** provided the *meta*‐arylated products in 46 % NMR yield as a 1 : 1 mixture of mono‐ and diarylated products **3** **a**. As expected, both an increase in yield and mono/di selectivity was achieved using bridgehead‐substituted NBEs. **N7** provided the arylated products in 67 % ^1^H‐NMR yield, with a 3 : 1 improvement of selectivity in favor of the monoarylated product, while **N8** afforded the arylated products in 57 % ^1^H‐NMR yield with a 9 : 1 regioselectivity. The yield and regioselectivity were further improved to 71 % and 12 : 1 (mono:diarylated products) respectively by converting the ester group to an amide (20 mol %, **N10**). Under these conditions, the monoarylated anisole derivative **3** **a** was isolated pure in 54 % yield. Other NBEs with two substituents at the bridgehead positions **N11** and **N12** ^[13]^ were evaluated, but low yields were obtained. Additionally, **N13** and **N14** ^[19]^ were tested, but only trace amounts of product were detected. Next, with the suitable conditions for the highly selective monoarylation of anisole, other unsubstituted aryl ethers were evaluated (Table 5). The arylation reaction of butyl phenyl ether and benzyl phenyl ether under the optimized reaction conditions provided the monoarylated products **8** **a** and **8** **b** in 56 % and 42 % isolated yields, respectively.

**Table 5:**
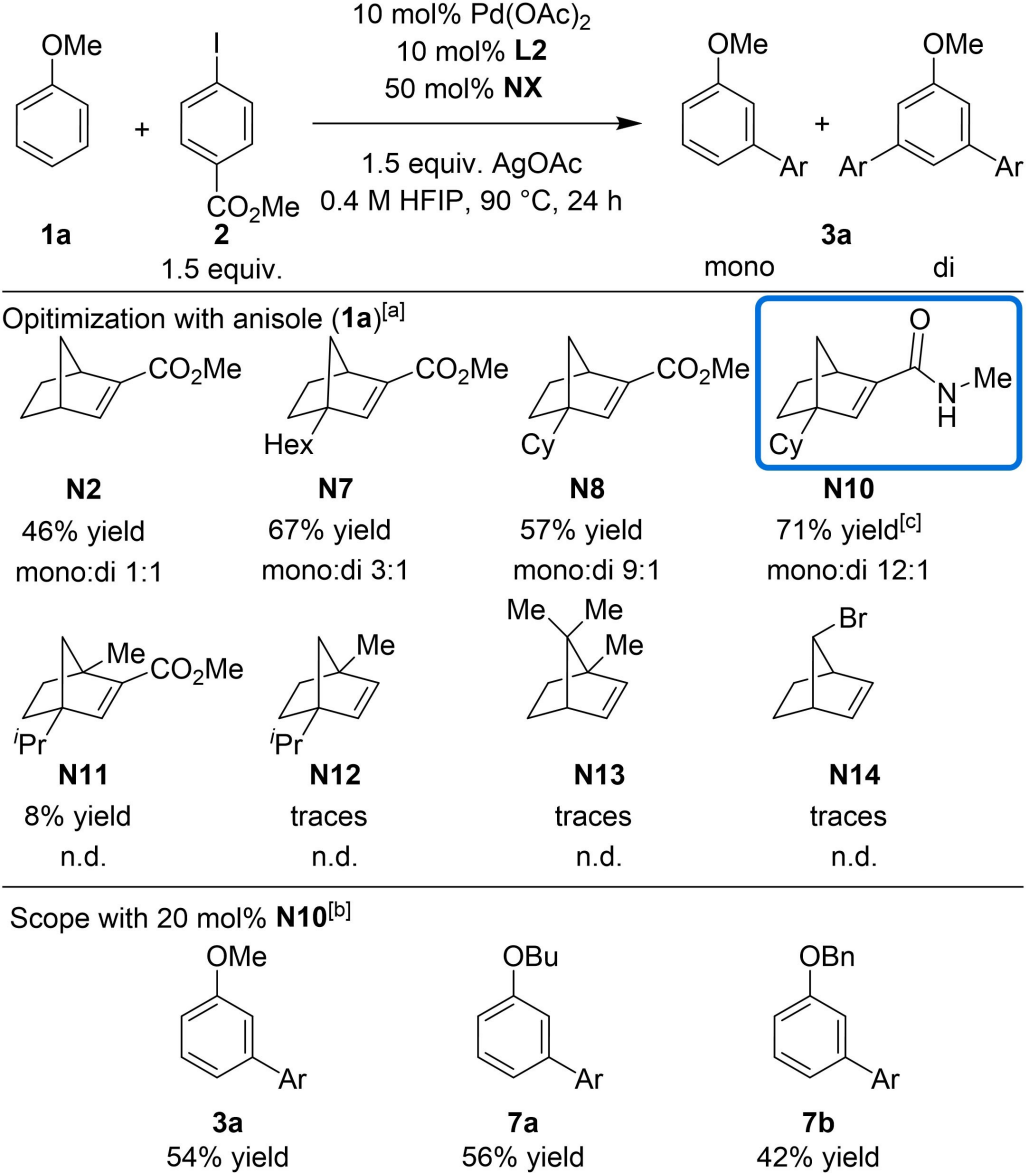
Non‐directed monoarylation of aryl ethers.

[a] ^1^H‐NMR yield of the crude mixture using CH_2_Br_2_ as internal standard. [b] Isolated yield of the monoarylated product. [c] 20 mol % **N10** was used. n.d.: not determined.

With this result in hand, we envisioned that a challenging unsymmetrical diarylation of anisole could be achieved in a sequential fashion through the use of two different NBEs. In the first arylation reaction, the aryl iodide is employed as the limiting reagent to ensure its complete consumption, together with 1.5 equiv of anisole and 20 mol % of **N10** (Table 6). After a simple filtration through a pad of Celite® and evaporation (i.e., no further purification) the crude material was reacted with the second aryl halide in the presence of 5 mol % of Pd/**L2** and 50 mol % of **N2**. In this stepwise approach, we introduced aryl iodides with *para*‐CO_2_Me, ‐Br and *meta*‐Cl substituents in the first step. In the second step, we used aryl iodides with *para*‐CO_2_Me and ‐Ac groups and aryl bromides bearing NO_2_ and CO_2_Me groups at the *ortho*‐position. Under these conditions, we were able to obtain the desired unsymmetrical *meta*‐arylated products in synthetically useful yields over two steps. It is worth mentioning that (as far as we know) this is the first example of unsymmetrical diarylation of non‐directed arenes with non‐conventional site‐selectivity.

**Table 6:**
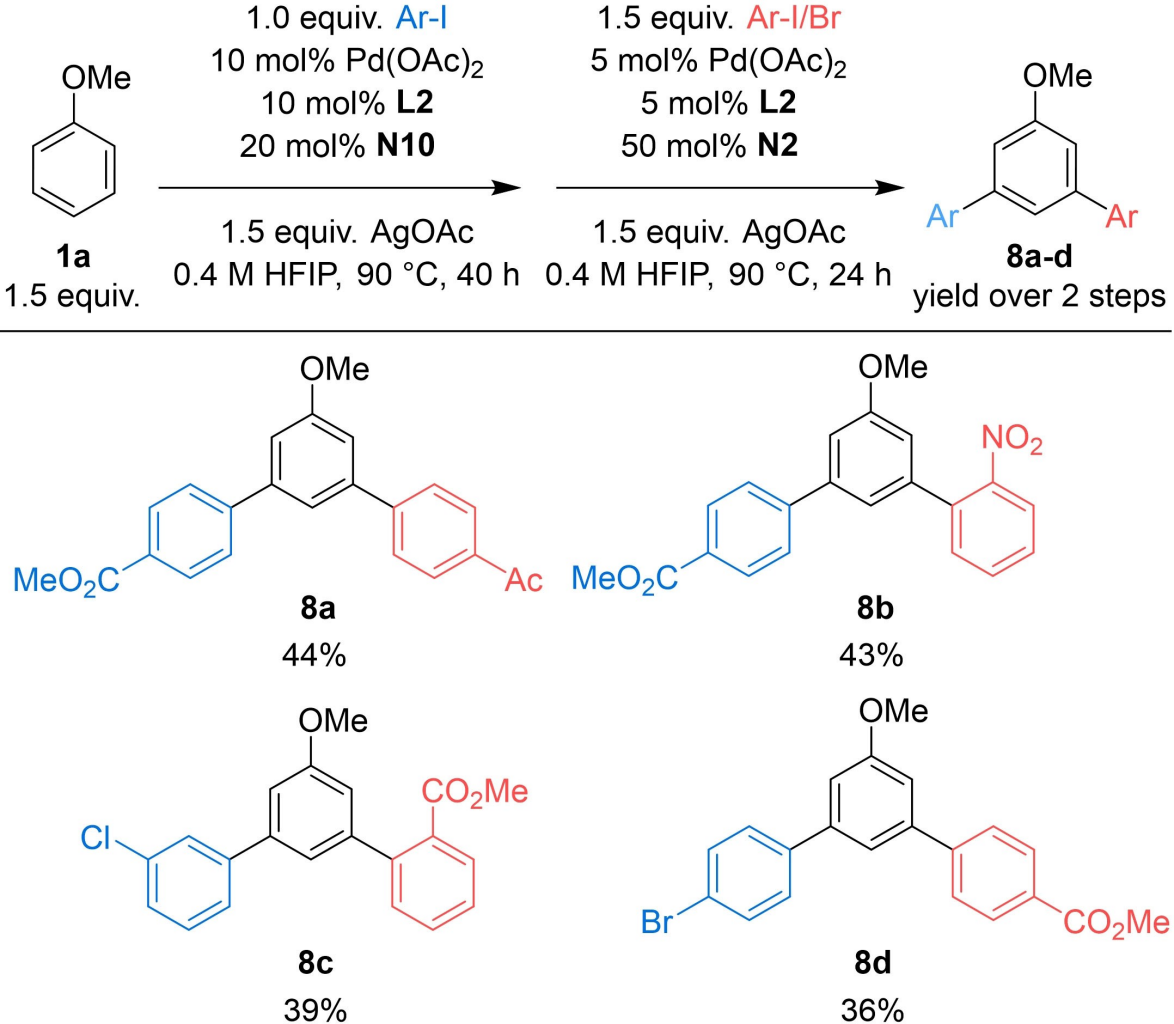
Non‐directed unsymmetrical *meta*‐diarylation of anisole.^[a]^

[a] Isolated yield of the diarylated product over 2 steps.

**Scheme 2:**
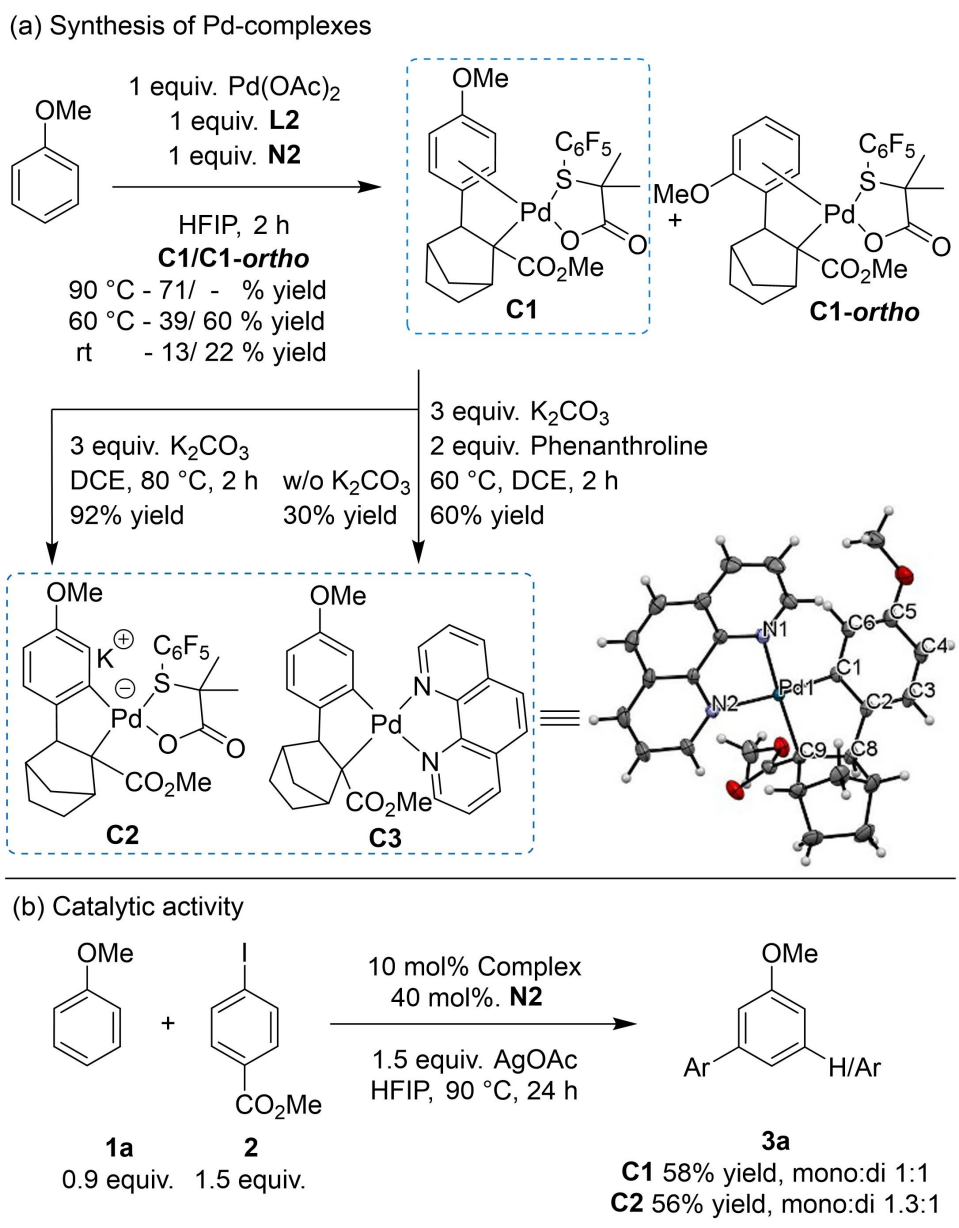
a) Synthesis of Pd complexes, featuring ORTEP of **C3** (50 % probability); selected bond lengths [Å] and bond angles [°]: Pd1−C1 1.998(2), Pd1−C9 2.095(2), Pd1−N1 2.1511(19), Pd1−N2 2.1739(18), C1−C2 1.403(3), C1−C6 1.410(3), C2−C3 1.400(3), C4−C5 1.395(4), C3−C4 1.386(4), C5−C6 1.396(3), C2−C8 1.492(3), C8−C9 1.565(3); C1‐Pd1‐C9 81.91(9), N1‐Pd1‐N2 77.36(7). b) General procedure for assessment of catalytic activity.

To obtain further insights into the mechanism of this transformation, we focused our efforts to isolate the intermediates proposed in the catalytic cycle established by Yu and co‐workers.^[8]^ To our delight, the stoichiometric reaction of anisole, Pd(OAc)_2_, **L2** and **N2** in HFIP at 90 °C (2 h) afforded palladium complex **C1**, isolated in 71 % yield and fully characterized by NMR and MS. **C1** corresponds to the Pd‐complex after the first *para*‐C−H activation and NBE insertion (Scheme 2a). The chemical shift of the anisyl ring protons along with the presence of four broad signals corresponding to the two *ortho‐* and two *meta* protons, suggests slow rotation around the Csp^2^−Csp^3^ bond at r.t, indicating the coordination of palladium to the arene ring is similar to previously reported examples.^[20]^ Interestingly, **C1** was also obtained at 60 °C and at r.t. in 39 % and 13 % yield, respectively. These results suggest that the first C−H activation and NBE insertion are not rate limiting steps under our conditions. Indeed, we observed a zero‐order dependence of the rate on **1** **b**, further indicating that the first C−H activation is not the rate limiting step (Supporting Information, Table S28).^[21]^ In addition to **C1**, we observed the formation of a new complex **C1**‐*ortho* at both 60 °C and r.t. that we assigned as the complex arising from the first *ortho*‐C−H activation and NBE insertion (Scheme 2a), suggesting that Pd‐complex **C1** is the thermodynamic product. Furthermore, **C1** was also obtained using DCE as solvent albeit in lower yields, <10 % at 60 °C for 2 h (50 % yield after 18 h). These results suggest that HFIP has a positive effect on the first C−H activation step, consistent with previously reported observations.^[22]^ Although we did not observe the formation of the complex after the second C−H activation step under the previously mentioned conditions, we were pleased to find that the addition of 3 equiv of K_2_CO_3_ to **C1** in DCE at 80 °C afforded the anionic complex **C2** (92 % isolated yield) featuring palladium attachment at the *meta*‐position of anisole. Additionally, the reaction of **C1** with 3 equiv of K_2_CO_3_ in the presence of phenanthroline in DCE at 60 °C provided complex **C3** in 60 % yield. Single crystals of **C3** were grown by layering heptane onto a CH_2_Cl_2_ solution, enabling confirmation of the structure by single‐crystal X‐ray crystallography, with the ORTEP diagram (50 % probability ellipsoids) shown in Scheme 2.^[23]^ Complex **C3** corresponds to the complex after the *meta*‐C−H activation bearing a phenanthroline ligand and with the typical insertion of NBE in a *cis‐*, *exo‐*manner.^[5]^ Alternatively, complex **C3** was obtained in 30 % yield without adding K_2_CO_3_.

With the complexes **C1** and **C2** in hand, we evaluated their catalytic activity in the reaction of anisole using 10 mol % of **C1** or **C2** under standard reaction conditions, with the addition of 40 mol % **N2**. Gratifyingly, the *meta*‐arylated products were obtained in 58 % (**C1**) and 56 % yield (**C2**), indicating that both species are catalytically active (Scheme 2b).

**Scheme 3:**
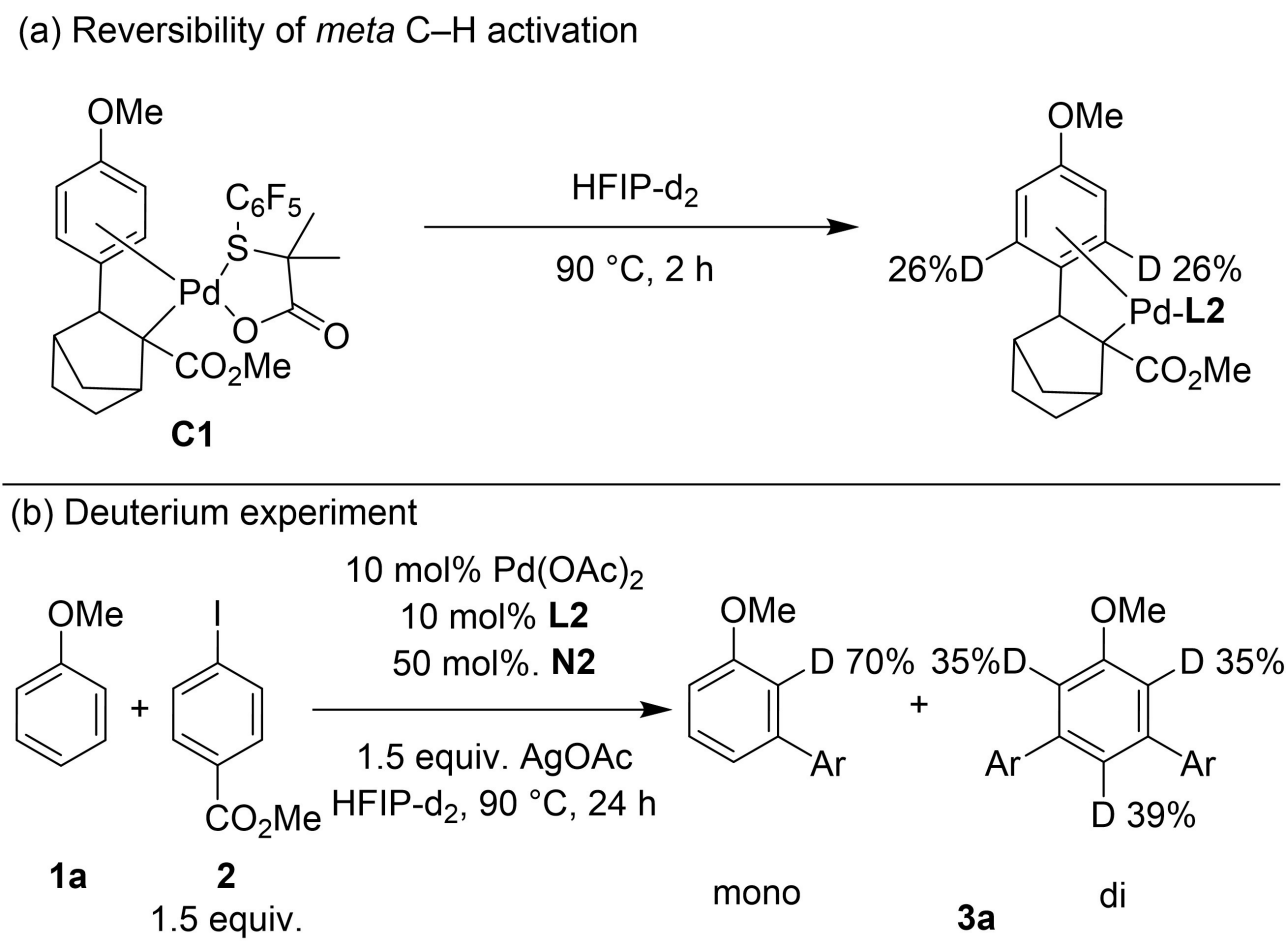
Deuteration experiments.

After establishing the catalytic activity of **C1** and **C2**, we investigated the reversibility of the first^[24]^ and second C−H activation step. We found that **C1** was partially deuterated at the *meta*‐position of anisole in the presence of HFIP‐*d*_2_ at 90 °C for 2 h, confirming that the second C−H activation step is reversible (Scheme 3a). Finally, the reaction of anisole and methyl 4‐iodobenzoate under standard reaction conditions using HFIP‐*d*_2_ as solvent was performed. We observed the monoarylated product with a 70 % deuterium incorporation exclusively at the *ortho*‐position and the diarylated product with around 35 % deuterium incorporation at the *ortho*‐ and *para*‐positions (Scheme 3b), consistent with previously reported results.^[8]^ Thus, it is reasonable to propose that the monoarylated product is derived only from the C−H activation at the *ortho*‐position and the diarylated product arrives from both C−H activation at the *para*‐position and *ortho*‐palladation of the monoarylated product.

**Scheme 4:**
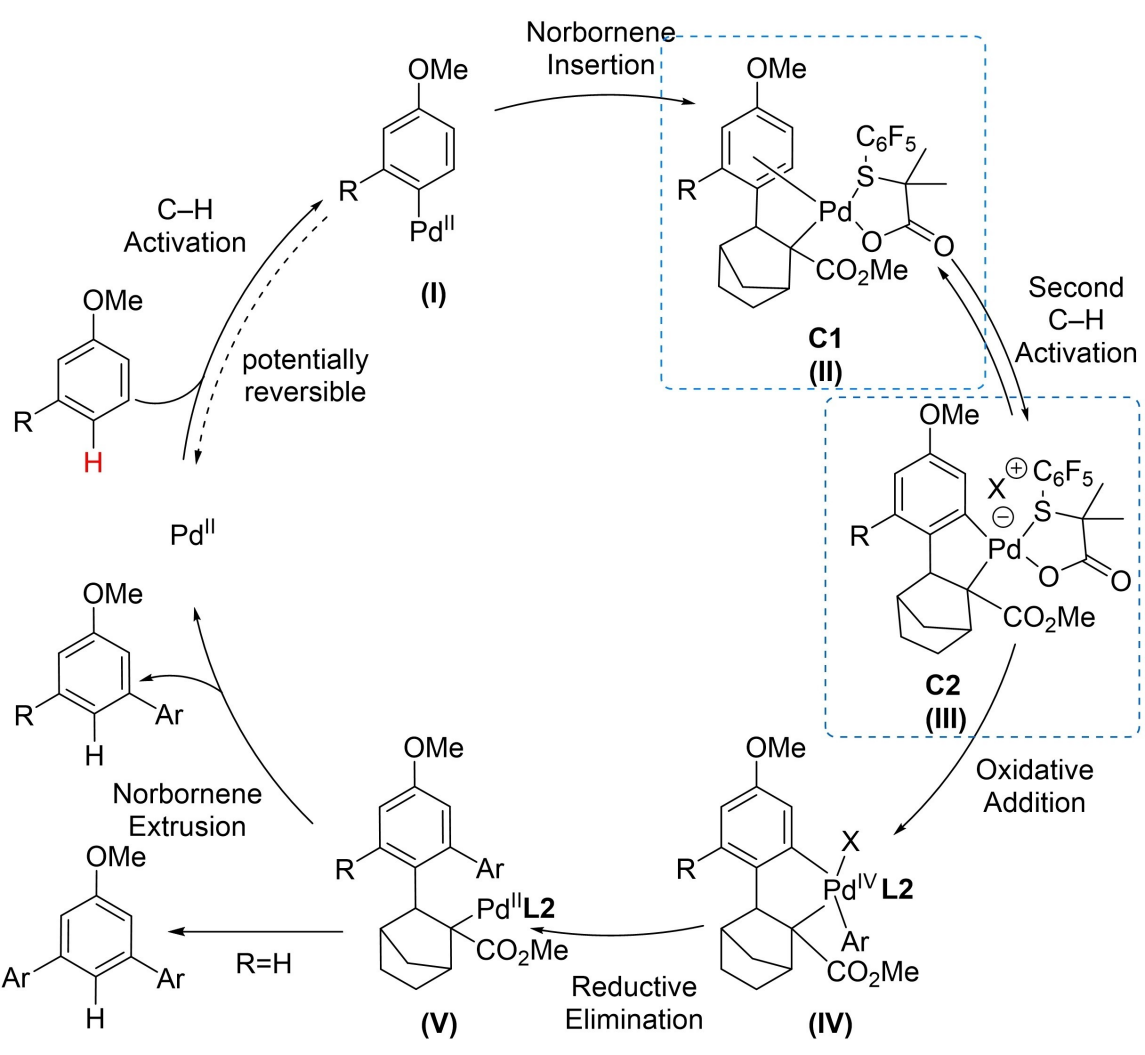
Proposed catalytic cycle.

Based on our results, we propose the catalytic cycle outlined in Scheme 4. We have demonstrated that the first and second C−H activation steps are reversible when investigated separately. However, the reversibility of the first C−H activation step cannot be observed in the complete catalytic cycle since the follow‐up step is faster. Furthermore, we suggest that the first C−H activation step and norbornene insertion are not rate‐determining steps.

## Conclusion

In conclusion, we have developed a new catalytic system based on Pd/NBE and an S,O‐ligand for the *meta* C−H arylation of aryl ethers that allows—for the first time—to use alkoxyarenes bearing both electron‐donating and ‐withdrawing substituents. *ortho*‐Substituted aryl ethers are well tolerated by overcoming the *ortho* constraint in Catellani‐type reactions by judicious selection of an appropriate NBE mediator. Remarkably, the monoarylation of alkoxyarenes is efficiently achieved allowing for the unprecedented introduction of two different aryl coupling partners to yield unsymmetrical terphenyls. Moreover, the new catalytic system based on Pd/S,O‐ligand only requires catalytic amounts of NBE to obtain the *meta*‐arylated products in good yield. Preliminary mechanistic investigations exclude the first C−H activation to be rate‐limiting step. Further applications of this new catalytic system are currently underway in our laboratory.

## Supporting Information

Experimental procedures, compounds characterizations, crystallographic data and mechanistic studies.

## Conflict of interest

The authors declare no conflict of interest.

1

## Supporting information

As a service to our authors and readers, this journal provides supporting information supplied by the authors. Such materials are peer reviewed and may be re‐organized for online delivery, but are not copy‐edited or typeset. Technical support issues arising from supporting information (other than missing files) should be addressed to the authors.

Supporting Information

Supporting Information

## Data Availability

The data that support the findings of this study are available from the corresponding author upon reasonable request.
