# Supplementary material for: S,O‐Ligand Promoted meta‐C−H Arylation of Anisole Derivatives via Palladium/Norbornene Catalysis
Source: Angew Chem Int Ed Engl. 2022 Jun 21;61(31):e202201750. doi: 10.1002/anie.202201750 (PMC9401001; doi:10.1002/anie.202201750)

checkCIF/PLATON report

Structure factors have been supplied for datablock(s) 20201124_VI364A_1_0m_a

THIS REPORT IS FOR GUIDANCE ONLY. IF USED AS PART OF A REVIEW PROCEDURE FOR PUBLICATION, IT SHOULD NOT REPLACE THE EXPERTISE OF AN EXPERIENCED CRYSTALLOGRAPHIC REFEREE.

No syntax errors found.　　CIF dictionary　　Interpreting this report

Datablock: 20201124_VI364A_1_0m_a

```
Bond precision:   C-C = 0.0039 A          Wavelength=0.71073

Cell:           a=10.2156(4)       b=11.4530(4)      c=12.6393(4)
                alpha=99.681(1)    beta=99.305(1)    gamma=112.104(1)
Temperature:    150 K

                 Calculated                        Reported
Volume           1308.98(8)                        1308.97(8)
Space group      P -1                              P -1
Hall group       -P 1                              -P 1
Moiety formula   C28 H26 N2 O3 Pd, C H2 Cl2 C28 H26 N2 O3 Pd, C1 H2 Cl2
Sum formula      C29 H28 Cl2 N2 O3 Pd              C29 H28 Cl2 N2 O3 Pd
Mr               629.83                            629.83
Dx,g cm-3        1.598                             1.598
Z                2                                 2
Mu (mm-1)        0.948                             0.948
F000             640.0                             640.0
F000'            638.81
h,k,lmax         16,18,20                          16,18,20
Nref             12085                             12073
Tmin,Tmax        0.738,0.903                       0.676,0.748
Tmin'            0.723

Correction method= # Reported T Limits: Tmin=0.676 Tmax=0.748
AbsCorr = MULTI-SCAN

Data completeness= 0.999           Theta(max)= 35.630

R(reflections)= 0.0483( 10951)                         wR2(reflections)=
                                                       0.1355( 12073)
S = 1.098                  Npar= 346
```

The following ALERTS were generated. Each ALERT has the format
test-name_ALERT_alert-type_alert-level.
Click on the hyperlinks for more details of the test.

Alert level C

```
PLAT243_ALERT_4_C High   'Solvent' Ueq as Compared to Neighbors of       Cl1B Check
PLAT329_ALERT_4_C Carbon Atom Hybridisation Unclear for .........         C1B Check
PLAT906_ALERT_3_C Large K Value in the Analysis of Variance ......      2.338 Check
PLAT910_ALERT_3_C Missing # of FCF Reflection(s) Below Theta(Min).          7 Note
PLAT918_ALERT_3_C Reflection(s) with I(obs) much Smaller I(calc) .          1 Check
PLAT934_ALERT_3_C Number of (Iobs-Icalc)/Sigma(W) > 10 Outliers ..          1 Check
PLAT971_ALERT_2_C Check Calcd Resid. Dens.  0.61A   From Pd1             2.33 eA-3
PLAT971_ALERT_2_C Check Calcd Resid. Dens.  0.90A   From Cl2B            2.05 eA-3
PLAT971_ALERT_2_C Check Calcd Resid. Dens.  0.93A   From C1B'            2.02 eA-3
PLAT972_ALERT_2_C Check Calcd Resid. Dens.  0.52A   From Cl2B           -2.13 eA-3
PLAT972_ALERT_2_C Check Calcd Resid. Dens.  0.46A   From Cl2B           -1.82 eA-3
PLAT972_ALERT_2_C Check Calcd Resid. Dens.  1.99A   From O1             -1.71 eA-3
PLAT972_ALERT_2_C Check Calcd Resid. Dens.  0.44A   From Cl1B           -1.67 eA-3
PLAT972_ALERT_2_C Check Calcd Resid. Dens.  0.57A   From Cl1B           -1.63 eA-3
PLAT972_ALERT_2_C Check Calcd Resid. Dens.  0.35A   From Cl1B           -1.57 eA-3
PLAT972_ALERT_2_C Check Calcd Resid. Dens.  0.46A   From Cl2B           -1.52 eA-3
```

Alert level G

```
PLAT154_ALERT_1_G The s.u.'s on the Cell Angles are Equal ..(Note)      0.001 Degree
PLAT302_ALERT_4_G Anion/Solvent/Minor-Residue Disorder (Resd  2  )        33% Note
PLAT411_ALERT_2_G Short Inter H...H Contact  H18      ..H1BB     .       1.87 Ang.
                                               1-x,1-y,1-z  =      2_666 Check
PLAT432_ALERT_2_G Short Inter X...Y Contact  C18      ..C1B              2.97 Ang.
                                               1-x,1-y,1-z  =      2_666 Check
PLAT720_ALERT_4_G Number of Unusual/Non-Standard Labels .........           5 Note
PLAT793_ALERT_4_G Model has Chirality at C8          (Centro SPGR)          R Verify
PLAT793_ALERT_4_G Model has Chirality at C9          (Centro SPGR)          R Verify
PLAT793_ALERT_4_G Model has Chirality at C10         (Centro SPGR)          S Verify
PLAT793_ALERT_4_G Model has Chirality at C13         (Centro SPGR)          R Verify
PLAT794_ALERT_5_G Tentative Bond Valency for Pd1       (II)      .       1.61 Info
PLAT912_ALERT_4_G Missing # of FCF Reflections Above STh/L=  0.600          4 Note
PLAT933_ALERT_2_G Number of OMIT Records in Embedded .res File ...          6 Note
PLAT978_ALERT_2_G Number C-C Bonds with Positive Residual Density.         11 Info
```

0 **ALERT level A** = Most likely a serious problem - resolve or explain
0 **ALERT level B** = A potentially serious problem, consider carefully
16 **ALERT level C** = Check. Ensure it is not caused by an omission or oversight
13 **ALERT level G** = General information/check it is not something unexpected

1 ALERT type 1 CIF construction/syntax error, inconsistent or missing data
14 ALERT type 2 Indicator that the structure model may be wrong or deficient
4 ALERT type 3 Indicator that the structure quality may be low
9 ALERT type 4 Improvement, methodology, query or suggestion
1 ALERT type 5 Informative message, check

It is advisable to attempt to resolve as many as possible of the alerts in all categories. Often the minor alerts point to easily fixed oversights, errors and omissions in your CIF or refinement strategy, so attention to these fine details can be worthwhile. In order to resolve some of the more serious problems it may be necessary to carry out additional measurements or structure refinements. However, the purpose of your study may justify the reported deviations and the more serious of these should normally be commented upon in the discussion or experimental section of a paper or in the "special_details" fields of the CIF. checkCIF was carefully designed to identify outliers and unusual parameters, but every test has its limitations and alerts that are not important in a particular case may appear. Conversely, the absence of alerts does not guarantee there are no aspects of the results needing attention. It is up to the individual to critically assess their own results and, if necessary, seek expert advice.

Publication of your CIF in IUCr journals

A basic structural check has been run on your CIF. These basic checks will be run on all CIFs submitted for publication in IUCr journals (*Acta Crystallographica*, *Journal of Applied Crystallography*, *Journal of Synchrotron Radiation*); however, if you intend to submit to *Acta Crystallographica Section C* or *E* or *IUCrData*, you should make sure that full publication checks are run on the final version of your CIF prior to submission.

Publication of your CIF in other journals

Please refer to the *Notes for Authors* of the relevant journal for any special instructions relating to CIF submission.

PLATON version of 13/07/2021; check.def file version of 13/07/2021

Datablock 20201124_VI364A_1_0m_a - ellipsoid plot

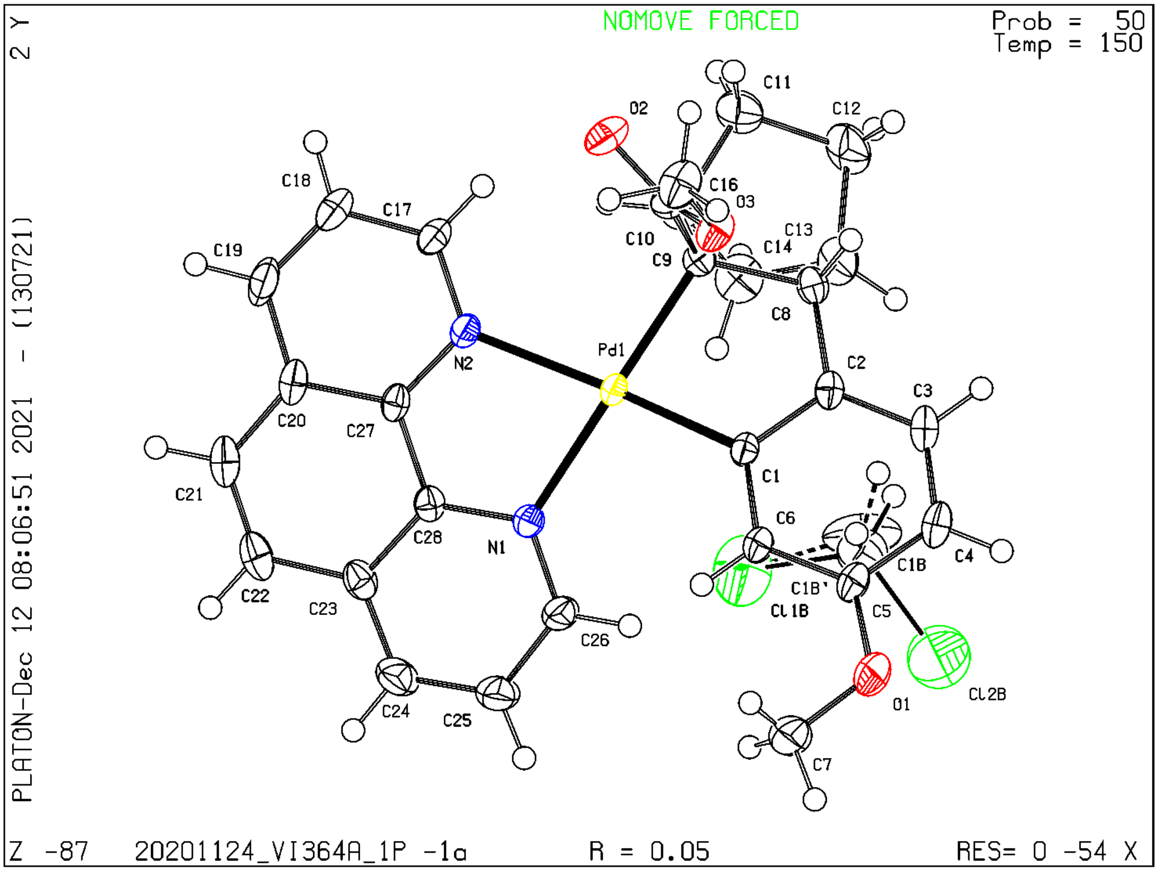

Supplement: Supplementary file 1 — Supporting Information [file ANIE-61-0-s001.pdf]
