# Supplementary material for: S,O‐Ligand Promoted meta‐C−H Arylation of Anisole Derivatives via Palladium/Norbornene Catalysis
Source: Angew Chem Int Ed Engl. 2022 Jun 21;61(31):e202201750. doi: 10.1002/anie.202201750 (PMC9401001; doi:10.1002/anie.202201750)
Supplement: Supplementary file 2 — Supporting Information [file ANIE-61-0-s002.pdf]

Angewandte Chemie
Eine Zeitschrift der Gesellschaft Deutscher Chemiker

Supporting Information

S,O-Ligand Promoted *meta*-C–H Arylation of Anisole Derivatives via Palladium/Norbornene Catalysis

*V. Sukowski, M. van Borselen, S. Mathew, M. Á. Fernández-Ibáñez**

Table of Contents

1. General Information ... 1
2. Synthesis of Aryl Ethers ... 1
3. Synthesis of S,O-Ligands ... 2
4. Synthesis and Characterization of Norbornenes ... 3
4.1. Synthesis of Norbornenes ... 3
4.2. Determination of Stereochemistry of Norbornane Intermediates ... 15
5. Optimization of the *meta*-C–H Arylation of Aryl Ethers ... 23
5.1. Optimization of the *meta*-C–H Arylation of Anisole ... 23
5.2 Optimization of the *meta*-Arylation of 3-Methylanisole (*meta*-substituted substrates) ... 25
5.3 Evaluation of Other Reported Ligands Under the Optimized Reaction Conditions ... 25
6. General Procedure A for the *meta*-C–H Arylation of *meta*-Substituted Anisoles ... 27
7. Optimization of the *meta* Arylation of 2-Methylanisole (*ortho*-substituted substrates) ... 35
8. General Procedure B for the *meta*-C–H Arylation of *ortho*-Substituted Anisoles ... 36
9. Optimization of the *meta* Arylation of Anisole ... 39
10. General Procedure C for the *meta*-C–H Arylation of Unsubstituted Aryl Ethers ... 39
10.1. General Procedure D for Unsymmetrical Diarylation of Anisole ... 40
11. Mechanistic Studies ... 42
12. Single-Crystal X-Ray Diffraction Studies ... 57
13. References ... 59
14. ^{1}H and ^{13}C NMR Spectra ... 61

1. General Information

Chromatography: Flash column chromatography was performed using Macherey-Nagel Silica 60 (particle size 0.04–0.063 mm) under compressed air flow or a Buchi C-850 automatic column machine with FlashPure silica cartridges, TLC: Merck TLC plates (0.25 mm) precoated with silica gel 60 F_{254}. Visualization of the TLC was performed by UV, $KMnO_4$ and Phosphomolybdic Acid (PMA) staining. Anhydrous CH_2Cl_2, Et_2O and THF were obtained from pre-dried materials via an MBRAUN SPS-800 machine and stored under N_2 atmosphere. High-resolution mass spectra (HRMS) were recorded on an AccuTOF GC v 4g, JMST100GCV mass spectrometer (JEOL, Japan) and HR-ToF Bruker Daltonik GmbH (Bremen, Germany) Impact II, an ESI-ToF MS capable of resolution of at least 40,000 FWHM. The FD/FI probe was equipped with an FD Emitter, Carbotec, FD = 10 µm. Current rate = 51.2 mA/min over 1.2 min using field desorption (FD) as an ionization method. Bruker DRX-300, 400 and 500 MHz instruments were used to record NMR spectra. Chemical shift values are reported in ppm with the solvent resonance as the internal standard ($CDCl_3$: δ 7.26 for ^1H, δ 77.16 for ^{13}C). Data are reported as follows: chemical shifts, multiplicity (s = singlet, d = doublet, dd = doublet of doublets, t = triplet, dt = doublet triplet, bs = broad singlet, m = multiplet), coupling constants (Hz), and integration. ATR technique was used in IR spectroscopy on a Bruker Alpha-P. Melting points (M.P.) were measured in Buchi M-565 melting point apparatus. All reagents and solvents were used as received. $Pd(OAc)_2$ was purchased from Strem.

2. Synthesis of Aryl Ethers

Substrates **1a-d**, **1f-n**, **1p**, **2a-m**, **5a**, **5b** and **5d-i** were purchased from Merck KGaA, Alfa Aesar GmbH & Co KG or Fluorochem Ltd and were used without further purification.

3-(Methoxyphenyl)trimethylsilane (1e)

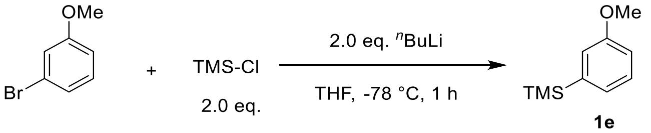

1e
73% yield

1-Bromo-3-methoxybenzene (0.63 mL, 5 mmol, 1 eq.) was dissolved in THF (10 mL, 0.5 M) under nitrogen atmosphere and the solution was cooled to -78 °C followed by the slow addition of ^{n}BuLi (4 mL, 10 mmol, 2 eq., 2.5 M in hexanes). After stirring the mixture for 1 h at -78 °C, chlorotrimethylsilane (1.27 mL, 10 mmol, 2 eq.) was added and the reaction was allowed to warm to room temperature and stirred for an additional 2 h. Water was added, and the aqueous layer was extracted with Et_2O (× 3). The combined organic layers were dried over anhydrous $MgSO_4$, filtered and concentrated under reduced pressure. Purification by column chromatography on silica gel using Cy / EtOAc (9:1 v/v) as eluent provided the pure compound as a clear oil (657 mg, 73% yield). **^{1}H NMR** (400 MHz, $CDCl_3$) δ 7.30 (t, *J* = 7.7 Hz, 1H), 7.17 – 7.09 (m, 1H), 7.06 (d, *J* = 2.4 Hz, 1H), 6.96 – 6.83 (m, 1H), 3.83 (s, 3H), 0.27 (s, 9H). **^{13}C NMR** (101 MHz, $CDCl_3$) δ 159.08, 142.35, 129.10, 125.72, 119.13, 113.96, 55.23, -1.01. ^{1}H NMR of the isolated material matched with that reported in the literature.[1]

5-Methoxy-1,2,3,4-tetrahydronaphthalene (1o)

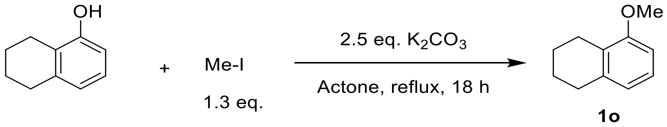

1o
47% yield

A procedure described in the literature was followed.[2] 5,6,7,8-Tetrahydronaphthalenol (0.45 g, 3 mmol, 1 eq.), iodomethane (0.2 mL, 3.3 mmol, 1.3 eq.) and K_2CO_3 (1.04 g, 7.5 mmol, 2.5 eq.) were dissolved in acetone and refluxed overnight. The reaction was quenched with MeOH (0.4 mL), cooled to room temperature, filtered and concentrated under reduced pressure. Then, water was added and extracted with EtOAc (× 3). The combined organic layers were dried over anhydrous $MgSO_4$, filtered and concentrated under reduced pressure. The crude was dissolved in hexane, filtered through silica and rinsed with hexanes. The product was obtained as a clear oil (210, 6 mg, 47% yield). **^{1}H NMR** (400 MHz, $CDCl_3$) δ 7.07 (t, *J* = 7.9 Hz, 1H), 6.70 (d, *J* = 7.6 Hz, 1H), 6.66 (d, *J* = 8.2 Hz, 1H), 3.82 (s, 3H), 2.76 (t, *J* = 6.0 Hz, 2H), 2.65 (t, *J* = 6.0 Hz, 2H), 1.91 – 1.68 (m, 4H). ^{1}H NMR of the isolated material matched with that reported in the literature.[3]

2-*tert*-Butylanisole (5c)

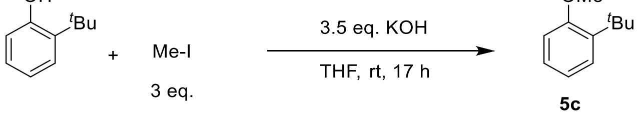

5c
69% yield

A procedure described in the literature was followed.[4] A solution of 2-*tert*-butylphenol (0.46 mL, 1 eq., 3 mmol) in THF (0.75 mL) was slowly added to a suspension of freshly powdered KOH (0.59 g, 3.5 eq, 10.5 mmol) in THF (1.5 mL), while the temperature was kept below 10 °C. The mixture was stirred for 2 h at room temperature, followed by the dropwise addition of iodomethane (0.56 mL, 3 eq., 9 mmol) at 0 °C. The mixture was stirred for 17 h at room temperature. Then, the mixture was filtered and concentrated under reduced pressure, after which the product was obtained as a yellow oil (0.338g, 69% yield). **^{1}H NMR** (400 MHz, $CDCl_3$) δ 7.29 (dd, *J* = 7.6, 1.6 Hz, 1H), 7.20 (td, *J* = 7.6, 1.7 Hz, 1H), 6.94 – 6.87

(m, 2H), 3.85 (s, 3H), 1.39 (s, 9H). **^{13}C NMR** (101 MHz, $CDCl_3$) δ 158.67, 138.37, 127.13, 126.65, 120.40, 111.67, 55.10, 34.95, 29.86. **HRMS** (FI) calculated for $C_{11}H_{16}O[M]^+$: 164.1201; found: 164.1312. **IR** (neat): v_{max} (cm^{-1}): 2953, 1489, 1461, 1449, 1234, 1095, 1030, 744. ^{1}H NMR of the isolated material matched with that reported in the literature.[4]

3. Synthesis of S,O-Ligands

Ligands **L1, L3-L9 and L11-L16** were synthesized according to reported procedures.[5]

Table S1. Overview of screened S,O-Ligands

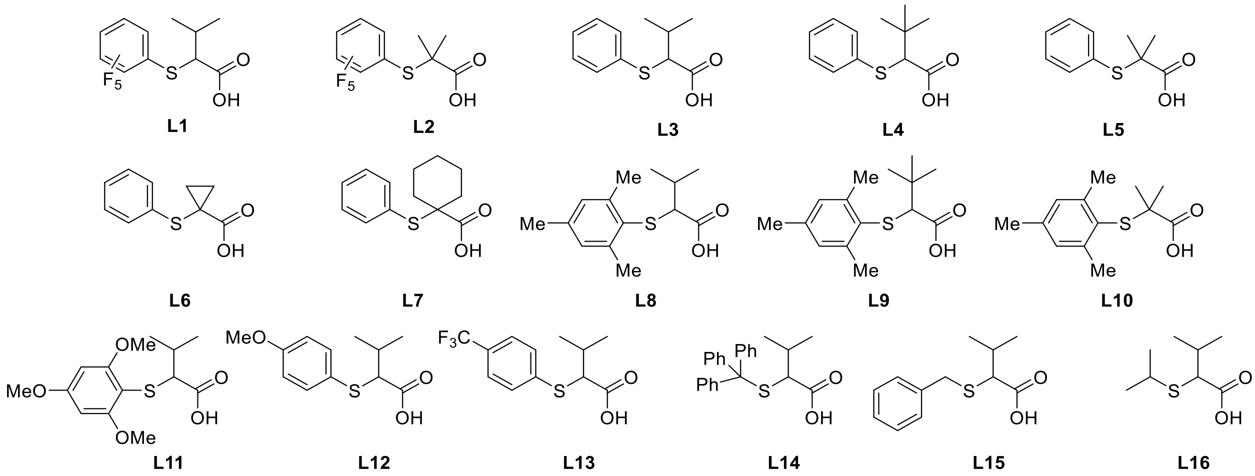

2-Methyl-2-[(perfluorophenyl)thio]propanoic acid (L2)

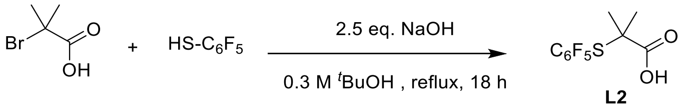

L2
76% yield

2,3,4,5,6-Pentafluorothiophenol (1.4 mL, 10 mmol, 1.0 eq.) was added to a mixture of 2-bromo-2-methylpropanoic acid (1.7 g, 10 mmol, 1 eq.) and NaOH (1.0 g, 25 mmol, 2.5 eq) in ^{t}BuOH (34 mL, 0.3 M) at room temperature. The reaction was refluxed overnight and concentrated under reduced pressure. The resulting crude was dissolved in water and acidified (6 M HCl solution) until pH = 1. The aqueous layer was extracted with EtOAc (× 3) and the combined organic layers were dried over anhydrous $MgSO_4$, filtered and concentrated under reduced pressure. Purification by column chromatography on silica gel using Cy / EtOAc (9:1 v/v) as eluent provided the pure compound as white crystals (2.2 g, 76% yield). **^{1}H NMR** (400 MHz, $CDCl_3$) δ 1.56 (s, 6H). **^{19}F NMR** (376 MHz, $CDCl_3$) δ -128.46 – -129.66 (m), -147.81 – -149.16 (m), -160.15 – -161.52 (m). **^{13}C NMR** (101 MHz, $CDCl_3$) δ 178.71, 150.71 – 149.63 (m), 148.52 – 147.16 (m), 144.69 – 143.69 (m), 141.90 – 141.27 (m), 139.19 – 138.48 (m), 136.65 – 135.97 (m), 51.72, 24.82. **HRMS** (ESI): *m/z* calculated for $C_{20}H_{12}F_{10}Na_1O_4S_2$ [2M+Na]$^-$= 592.9915; found= 592.9908 **IR** (neat): v_{max} (cm^{-1}): 2980, 2902, 2659, 2550, 2324, 2079, 1701, 1638, 1511, 1483, 1403, 1385, 1370, 1287, 1173, 1124, 1093, 1013, 978, 930, 862, 815, 756, 728, 664, 634, 551, 514, 432.

2-(Mesitylthio)-2-methylpropanoic acid (L10)

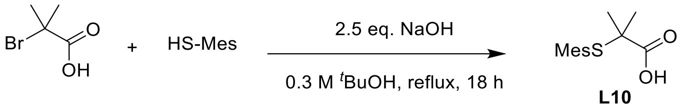

L10
5% yield

Mesityl thiophenol (152.0 µL, 1.0 mmol, 1 eq.) was added to a mixture of 2-bromo-2-methylpropanoic acid (170 mg, 1.0 mmol, 1 eq.) and NaOH (0.1 g, 2.5 mmol, 2.5 eq) in ^{t}BuOH (3.4 mL, 0.3 M) at room temperature. The reaction was refluxed overnight and concentrated under reduced pressure. The resulting crude was dissolved in water and acidified (6 M HCl solution) until pH = 1. The aqueous layer was extracted with EtOAc (× 3) and the combined organic layers were dried over anhydrous $MgSO_4$, filtered and concentrated under reduced pressure. Purification by column chromatography on silica gel using Cy / DCM (7:3 v/v) as an eluent provided the pure compound as white crystals (40.1 mg, 5% yield). **^{1}H NMR** (400 MHz, $CDCl_3$) δ 6.94 (s, 2H), 2.49 (s, 6H), 2.27 (s, 3H), 1.45 (s, 6H). **^{13}C NMR** (101 MHz, $CDCl_3$) δ 180.57, 145.48, 139.55, 129.20, 126.96, 50.57, 25.56, 22.57, 21.24. **HRMS** (ESI): *m/z* calculated for $C_{13}H_{18}O_2S_1$ [M-H]$^-$: 237.0949; found: 237.0958.

4. Synthesis and Characterization of Norbornenes

4.1. Synthesis of Norbornenes

Norbornene **N1** was purchased from Alfa Aesar GmbH & Co KG and was used without further purification. Norbornenes **N3**,[7] **N4,**[6] **N5,**[6] **N6**[7] and **N12**[8] were synthesized according to reported procedures.

Table S2. Overview of norbornenes

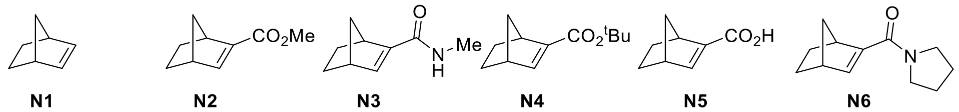

N1 **N2** **N3** **N4** **N5** **N6**

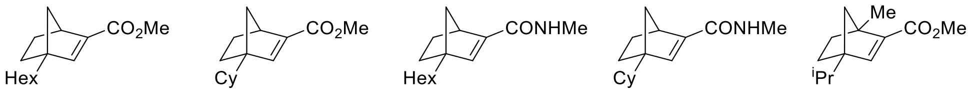

N7 **N8** **N9** **N10** **N11**

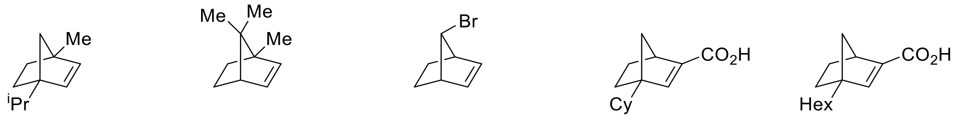

N12 **N13** **N14** **N15** **N16**

N-Methylbicyclo[2.2.1]hept-2-ene-2-carboxamide (N3)

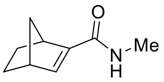

N3

White solid, **R_f** = 0.13 (Cy / EtOAc = 1.5:1). **^{1}H NMR** (400 MHz, $CDCl_3$) δ 6.62 (d, J = 3.1 Hz, 1H), 5.65 (s, 1H), 3.14 (s, 1H), 2.99 (s, 1H), 2.87 (d, J = 4.6 Hz, 3H), 1.75 (ddq, J = 10.6, 5.2, 2.9 Hz, 2H), 1.48 (dp, J = 8.3, 2.1 Hz, 1H), 1.22 – 1.19 (m, 1H), 1.16 – 1.05 (m, 2H). ^{1}H NMR of the isolated material matched with that reported in the literature.[7]

tert-Butylbicyclo[2.2.1]hept-2-ene-2-carboxylate (N4)

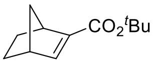

N4

Clear oil, **R_f** = 0.232 (Cy / DCM = 1:1). **^{1}H NMR** (400 MHz, $CDCl_3$) δ 6.80 (d, J = 3.2 Hz, 1H), 3.20 (s, 1H), 2.98 (s, 1H), 1.82 – 1.64 (m, 2H), 1.51 – 1.43 (m, 10H), 1.17 (dd, J = 8.5, 1.3 Hz, 1H), 1.08 (dd, J = 7.9, 2.4 Hz, 2H). ^{1}H NMR of the isolated material matched with that reported in the literature.[6]

Bicyclo[2.2.1]hept-2-ene-2-carboxylic acid (N5)

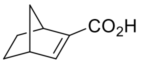

N5

Clear oil, **R_f** = 0.64 (Cy / EtOAc = 1.5:1). **^{1}H NMR** (400 MHz, $CDCl_3$) δ 7.08 (d, J = 3.2 Hz, 1H), 3.26 (s, 1H), 3.05 (s, 1H), 1.90 – 1.66 (m, 2H), 1.51 (dp, J = 8.5, 2.1 Hz, 1H), 1.22 (dd, J = 8.6, 1.3 Hz, 1H), 1.10 (ttd, J = 8.9, 6.6, 3.3 Hz, 2H). ^{1}H NMR of the isolated material matched with that reported in the literature.[6]

Bicyclo[2.2.1]hept-2-en-2-yl)(pyrrolidin-1-yl)methanone (N6)

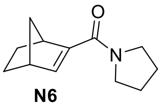

N6

White solid, **R_f** = 0.15 (Cy / EtOAc = 1.5:1). **^{1}H NMR** (400 MHz, $CDCl_3$) δ 6.33 (d, *J* = 3.2 Hz, 1H), 3.75 – 3.43 (m, 4H), 3.28 (s, 1H), 3.00 (t, *J* = 2.6 Hz, 1H), 2.03 – 1.79 (m, 4H), 1.73 (dd, *J* = 8.6, 2.9 Hz, 2H), 1.39 (dt, *J* = 8.4, 2.1 Hz, 1H), 1.26 (ddd, *J* = 9.5, 7.8, 2.2 Hz, 1H), 1.13 (d, *J* = 8.4 Hz, 1H), 1.07 (ddd, *J* = 9.6, 8.0, 2.2 Hz, 1H). ^{1}H NMR of the isolated material matched with that reported in the literature.[7]

1-Isopropyl-4-methylbicyclo[2.2.1]hept-2-ene (N12)

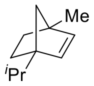

N12

Clear oil, **R_f** = 0.78 (Cy / DCM) = 1:1), **^{1}H NMR** (400 MHz, $CDCl_3$) δ 5.93 (d, *J* = 5.6 Hz, 1H), 5.79 (d, *J* = 5.6 Hz, 1H), 1.85 (hept, *J* = 6.9 Hz, 1H), 1.68 – 1.58 (m, 1H), 1.55 – 1.46 (m, 1H), 1.28 (s, 3H), 1.17 – 1.06 (m, 3H), 0.95 (t, *J* = 7.1 Hz, 6H). ^{1}H NMR of the isolated material matched with that reported in the literature.[8]

4.1.1. Synthesis of Norbornene N2

A procedure described in the literature was adapted for the synthesis of **N2**.[9]

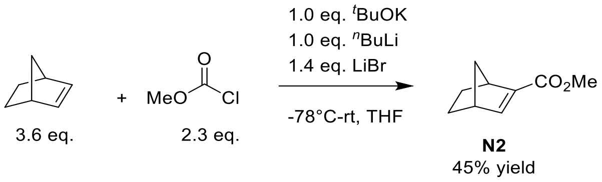

Norbornene (25.6 g, 273 mmol, 3.6 eq.) was dissolved in anhydrous THF (34 mL) under nitrogen atmosphere, and the solution was cooled to -78 °C. Then, a solution of KO^{t}Bu (50.5 mL, 75 mmol, 1.0 eq., 1.5 M in THF) was added followed by the slow addition of a solution of ^{n}BuLi (30 mL, 75 mmol, 1.0 eq., 2.5 M in hexanes). The reaction mixture was allowed to warm to -40 °C and stirred for an additional 30 min (-35 °C- -40 °C). The mixture was cooled to -50 °C and a solution of LiBr (34 mL, 102 mmol, 1.4 eq., 3 M in THF) was added [Note: The LiBr solution was prepared by drying LiBr under vacuum at 150 °C. After cooling down to room temperature, anhydrous THF was added providing a clear colorless solution]. The reaction mixture was slowly added via cannular transfer over 10 min to a second flask at -50 °C charged with methyl chloroformate (13.2 ml, 170 mmol, 2.3 eq.) and THF (34 mL). The reaction mixture was allowed to warm to room temperature and stirred for an additional hour. Water (200 mL) was added and the mixture was extracted with Et_2O (× 4). The combined organic layers were dried over anhydrous $MgSO_4$, filtered and concentrated under reduced pressure. The product was purified by distillation (85 °C, 20 mbar) to give **N2** as a clear colorless oil (45%, 5.4 g, 35 mmol). Alternatively, purification can be performed by flash column chromatography (Cy / Et_2O = 20:1 v/v).

Previous synthesis of **N2** are reported by the group of Yu[10] (3 steps starting from norcamphor) and by the group of Huang[11] (1 step with 1.0 eq. of NBE).

Methylbicyclo[2.2.1]hept-2-ene-2-carboxylate (N2)

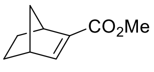

N2

Clear oil, **R$_f$** = 0.24 (Cy / DCM = 1:1). **^{1}H NMR** (400 MHz, $CDCl_3$) δ 6.92 (d, J = 3.3 Hz, 1H), 3.72 (s, 3H), 3.25 (s, 1H), 3.01 (dq, J = 3.3, 1.6 Hz, 1H), 1.93 – 1.63 (m, 2H), 1.48 (dp, J = 8.4, 2.1 Hz, 1H), 1.20 (dq, J = 8.5, 1.3 Hz, 1H), 1.08 (dddd, J = 13.0, 9.0, 6.6, 2.2 Hz, 2H). **^{13}C NMR** (101 MHz, $CDCl_3$) δ 165.49, 147.16, 140.85, 51.42, 48.34, 43.63, 42.05, 24.74, 24.61. **HRMS** (FI): *m/z* calculated for $C_9H_{12}O_2$ $[M]^{+}$= 152.0837; found= 152.081. ^{1}H NMR of the isolated material matched with that reported in the literature.[9]

4.1.2. Synthesis of Norbornenes N7 and N8

Scheme S1. Synthesis of **N7** and **N8.**

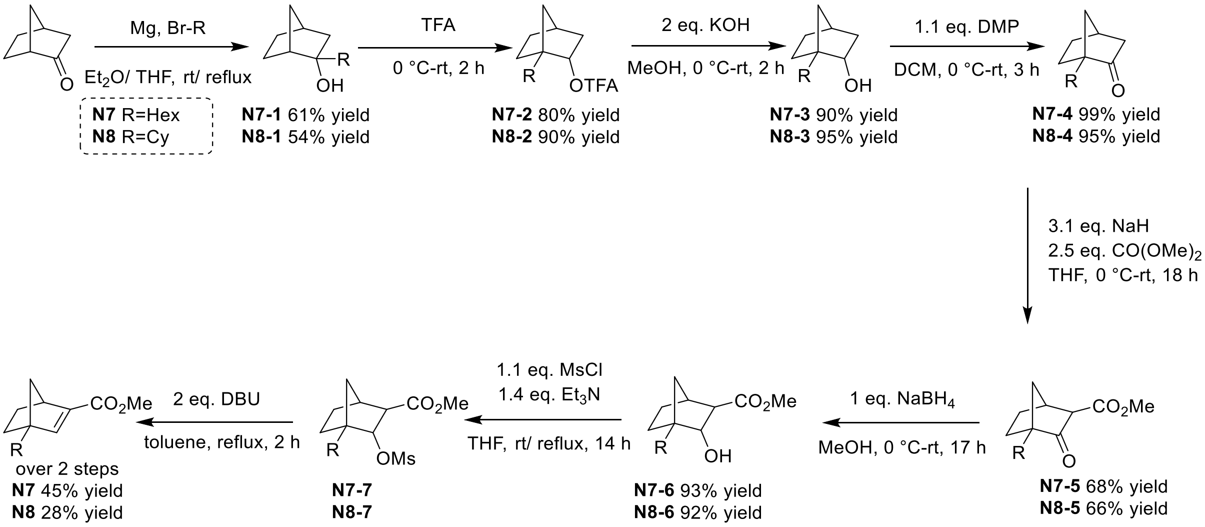

A procedure described in the literature was adapted for the synthesis of **N7-4 and N8-4**.[8]

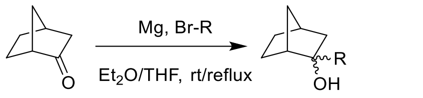

N7-1 61% yield, 3:1 dr
N8-1 54% yield, 12:1 dr

N7-1 (R = Hex): A dry three-neck round-bottom flask under nitrogen atmosphere was charged with magnesium (0.875 g, 36 mmol, 1.2 eq.), anhydrous Et_2O (67 mL, 0.45 M) and a small amount of iodine. 1-Bromohexane (5.16 ml, 36 mmol, 1.2 eq.) was added dropwise over 0.5 h under slightly elevated temperature (30 °C). The reaction mixture was stirred at room temperature for 1.5 h. Then, norcamphor (3.37 g, 30 mmol, 1.0 eq.) was slowly added to the mixture at -20 °C and stirred for 1 h and an additional 1 h at 0 °C. The reaction was quenched by pouring it slowly into cooled sat. NH_4Cl solution. The water layer was extracted with Et_2O (× 3) and the combined organic layers were washed once with brine, dried over anhydrous $MgSO_4$, filtered and concentrated under reduced pressure. A yellow oil was obtained, which was purified by flash column chromatography (Hex / Et_2O = 3:1, visualization on TLC with $KMnO_4$) to yield **N7-1** as colorless oil as a 3:1 mixture of diastereomers (61% yield, 3:1 dr, 3.6 g, 18.3 mmol). Trace amounts of the major diastereomer were isolated for characterization purpose.

N8-1 (R = Cy): A dry three-neck round-bottom flask under nitrogen atmosphere was charged with magnesium (7.3 g, 300 mmol, 3.0 eq.), anhydrous THF (225 mL, 0.45 M) and a small amount of iodine. 1-Bromocyclohexane (37.8 mL, 300 mmol, 3.0 eq.) was added dropwise over 0.5 h at 0 °C and the reaction mixture was stirred under reflux for 3.0 h. Then, norcamphor (3.37 g, 30 mmol, 1.0 eq.) was slowly added to the mixture at -20 °C and stirred for 1 h and an additional 1 h at 0 °C. The reaction was quenched by pouring it slowly into cooled sat. NH_4Cl solution. The water layer was extracted with Et_2O (× 3) and the combined organic layers were washed once with brine, dried over anhydrous $MgSO_4$, filtered and concentrated under reduced pressure. The crude was purified by flash column chromatography (Hex / Et_2O = 3:1, visualization on TLC with $KMnO_4$) to yield **N8-1** as a white solid as a 12:1 mixture of diastereomers (54% yield, 12:1 dr, 10.4 g, 53 mmol).

2-Hexanebicyclo[2.2.1]heptan-2-ol (N7-1)

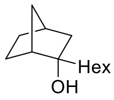

N7-1

Clear oil, **R$_f$** = 0.78 (Cy / Et_2O = 1:1). Major diastereomer: **^{1}H NMR** (400 MHz, $CDCl_3$) δ 2.17 (t, J = 4.3 Hz, 1H), 2.07 (d, J = 3.3 Hz, 1H), 2.00 – 1.88 (m, 1H), 1.66 – 1.53 (m, 2H), 1.52 – 1.45 (m, 3H), 1.42 – 1.22 (m, 11H), 1.07 (dd, J = 12.7, 3.3 Hz, 1H), 0.92 – 0.84 (m, 3H). **^{13}C NMR** (101 MHz, $CDCl_3$) δ 79.71, 46.73, 46.01, 42.55, 39.79, 37.22, 32.06, 30.02, 28.68, 23.38, 22.84, 22.31, 14.25. **IR** (neat): v_{max} (cm^{-1}): 3373, 2950, 2929, 2869, 2858, 1457, 1378, 1321, 1307, 1292, 1255, 1228, 1195, 1166, 1130, 1114, 1027, 1002, 979, 939, 724, 573, 517, 484, 442. Mixture of diastereomers in a ratio of a:b 2:1 **^{1}H NMR** (400 MHz, $CDCl_3$) δ 2.66 (s, 1H$_b$), 2.59 (d, *J* = 4.5 Hz, 1H$_b$), 2.17 (t, *J* = 4.7 Hz, 1H$_a$), 2.02-2.09 (m, 1H$_a$+1H$_b$), 1.99 – 1.89 (m, 1H$_a$), 1.88 – 1.70 (m, 4H$_b$), 1.65 – 1.22 (m, 16H$_a$+13H$_b$), 1.07 (dd, *J* = 12.8, 3.4 Hz, 1H$_a$), 0.94 – 0.83 (m, 3H$_a$+3H$_b$). **^{13}C NMR** (101 MHz, $CDCl_3$) δ 79.71$_{a+b}$, 50.00$_b$, 46.73$_a$, 46.01$_a$, 45.41$_b$, 42.55 $_{a+b}$, 38.87$_a$, 37.84 $_b$, 37.22 $_a$, 35.45 $_b$, 32.06 $_{a+b}$, 30.02 $_{a+b}$, 28.68$_a$, 27.33$_b$, 24.32$_b$, 23.37$_a$, 22.79 $_{a+b}$, 22.31 $_{a+b}$, 14.25$_{a+b}$.

2-Cyclohexylbicyclo[2.2.1]heptan-2-ol (N8-1)

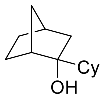

N8-1

White solid, **R$_f$** = 0.55 (Hex / Et_2O = 1:1). A mixture of two diastereomers was obtained in a ratio of 12:1, only the NMR peaks of the major diastereomer are listed here. **^{1}H NMR** δ 2.32 (dt, *J* = 3.1, 1.4 Hz, 1H), 2.18 (t, *J* = 4.2 Hz, 1H), 1.97 – 1.86 (m, 1H), 1.86 – 1.74 (m, 4H), 1.69 (tdd, *J* = 13.0, 4.1, 2.4 Hz, 2H), 1.61 – 1.50 (m, 1H), 1.44 (dt, *J* = 10.0, 2.1 Hz, 1H), 1.39 – 1.03 (m, 10H), 0.98 (dd, *J* = 12.9, 3.4 Hz, 1H). **^{13}C NMR** (101 MHz, $CDCl_3$) δ 81.50, 46.86, 45.10, 43.75, 38.72, 37.33, 28.42, 27.62, 27.06, 26.90, 26.89, 26.02, 22.81. **HRMS** (FI) calculated for $C_{13}H_{22}O$ $[M]^+$= 194.1671; found= 194.1671. **IR** (neat): v_{max} (cm^{-1}): 3469, 2926, 2851, 1717, 1449, 1375, 1344, 1308, 1262, 1242, 1187, 1168, 1145, 1120, 1104, 1052, 1011, 976, 940, 909, 892, 846, 803, 758, 742, 599, 542, 492, 477, 455.

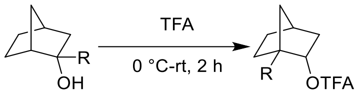

N7-2 80% yield
N8-2 90% yield

N7-2 (R = Hex): To an open flask charged with trifluoracetic acid (7.5 mL) at 0 °C, **N7-1** (3.08 g, 15.7 mmol, 1.0 eq.) was added dropwise and the reaction was allowed to warm to room temperature and stirred for 2 h [Note: The reaction can be followed by TLC (Hex / Et_2O = 1:1) using phosphomolybdic stain]. After pouring the mixture into ice water, the mixture was extracted with Et_2O (× 3) and the combined organic layers were washed with water (× 3), with sat. Na_2CO_3 solution (× 2) and with brine, dried over anhydrous $MgSO_4$, filtered and concentrated under reduced pressure to provide **N7-2** as a brown oil (80% yield, 3.67 g, 12.6 mmol), which was used in the next step without further purification.

N8-2 (R = Cy): Following the same procedure using **N8-1** (10.2 g, 52.2 mmol), **N8-2** was obtained as yellow oil (90 % yield, 13.6 g, 46.5 mmol), which was used in the next step without further purification.

1-Hexylbicyclo[2.2.1]heptan-2-yl 2,2,2-trifluoroacetate (N7-2)

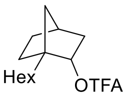

N7-2

Clear oil, $\mathbf{R_f}$ = 0.57 (Hex / Et_2O = 1:1), **^{1}H NMR** (400 MHz, $CDCl_3$) δ 4.84 (d, *J* = 7.0 Hz, 1H), 2.26 (t, *J* = 4.3 Hz, 1H), 2.04 – 1.89 (m, 1H), 1.69 – 1.36 (m, 6H), 1.31 – 1.12 (m, 11H), 0.94 – 0.78 (m, 3H). **^{13}C NMR** (101 MHz, $CDCl_3$) δ 161.35 – 153.14 (m), 122.81 – 105.34 (m), 82.75, 50.85, 40.68, 40.44, 35.55, 31.80, 30.45, 30.17, 29.77, 29.47, 25.54, 22.73, 14.19. **^{19}F NMR** (376 MHz, $CDCl_3$) δ -75.10. **HRMS** (FI): *m/z* calculated for $C_{15}H_{23}F_3O_2$ $[M]^+$= 292.1650; found= 292.1636. **IR** (neat): ν_{max} (cm^{-1}): 2957, 2930, 2873, 2860, 1779, 1347, 1220, 1161, 1149, 776, 723.

1-Cyclohexylbicyclo[2.2.1]heptan-2-yl 2,2,2-trifluoroacetate (N8-2)

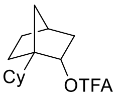

N8-2

Clear oil, $\mathbf{R_f}$ = 0.57 (Cy / Et_2O = 1:1). A mixture of two diastereomers was obtained in a ratio of 5:1, only NMR peaks of the major diastereomer are listed here. **^{1}H NMR (**300 MHz, $CDCl_3$) δ 4.93 (dt, *J* = 7.0, 1.9 Hz, 1H), 2.26 (d, *J* = 4.3 Hz, 1H), 1.97 (ddd, *J* = 13.9, 7.0, 2.4 Hz, 1H), 1.84 – 1.42 (m, 9H), 1.42 – 0.86 (m, 9H). ^{13}C NMR (75 MHz, $CDCl_3$) δ 156.92 (q, $J_{C\text{-}F}$ = 41.7 Hz), 114.66 (q, $J_{C\text{-}F}$ = 286.2 Hz)., 82.04, 77.44, 54.37, 40.46, 37.83, 37.15, 35.16, 29.41, 29.24, 28.45, 26.69, 26.55, 25.05. **HRMS** (FI) calculated for $C_{15}H_{21}F_3O_2$ $[M]^+$: 290.1494; found: 290.1504. **IR** (neat): ν_{max} (cm^{-1}): 2926, 2874, 2854, 1777, 1450, 1387, 1345, 1218, 1158, 1148, 1055, 988, 975, 949, 938, 870, 845, 776, 724, 526.

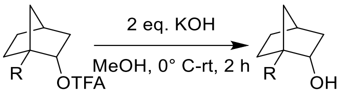

N7-3 90% yield, 15:1 dr
N8-3 95% yield

N7-3 (R = Hex): To an open flask charged with KOH (1.4 g, 25 mmol, 2.0 eq.) and methanol (25 mL, 1 M) at 0 °C, **N7-2** (3.67 g, 12.5 mmol, 1.0 eq.) was slowly added and stirred at room temperature for 2 h. Then, the reaction was quenched by slowly pouring it into ice water and neutralizing it with sat. NH_4Cl solution. After removing the methanol under reduced pressure, the water layer was extracted with Et_2O (× 3) and the combined organic layers were dried over anhydrous $MgSO_4$, filtered and concentrated under reduced pressure to obtain **N7-3** as a yellow oil (90% yield, 2.17 g, 11.3 mmol), which was used in the next step without further purification.

N8-3 (R = Cy): Following the same procedure using **N8-2** (13.5 g, 46.5 mmol), **N8-3** was obtained as a light yellow solid (95% yield, 9.4 g, 46.5 mmol), which was used in the next step without further purification.

1-Hexylbicyclo [2.2.1]heptan-2-ol (N7-3)

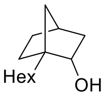

N7-3

Clear oil, $\mathbf{R_f}$ = 0.49, 0.38 (Cy / Et_2O = 1:1). A mixture of two diastereomers was obtained in a ratio of 15:1, only NMR peaks of the major diastereomer are listed here. **^{1}H NMR** (400 MHz, $CDCl_3$) δ 3.60 (dt, *J* = 7.0, 1.8 Hz, 1H), 2.14 (t, *J* = 4.2 Hz, 1H), 1.78 (ddd, *J* = 13.3, 6.9, 2.4 Hz, 1H), 1.57 – 1.43 (m, 3H), 1.39 – 1.23 (m, 12H), 1.19 – 1.07 (m, 1H), 1.05 – 0.96 (m, 2H), 0.93 – 0.84 (m, 3H). **^{13}C NMR** (101 MHz, $CDCl_3$) δ 75.76, 51.31, 43.04, 39.34, 35.44, 32.07, 30.78, 30.52, 30.25, 29.81, 25.74, 22.84, 14.27. **HRMS** (FI): *m/z* calculated for $C_{13}H_{24}O$ $[M]^+$= 196.1827; found= 196.1831. **IR** (neat): ν_{max} (cm^{-1}): 3376, 2950, 2925, 2857, 1456, 1378, 1339, 1299, 1258, 1212, 1174, 1124, 1065, 1035, 1011, 946, 936, 923, 852, 766, 723, 605, 488, 414.

1-Cyclohexylbicyclo [2.2.1]heptan-2-ol (N8-3)

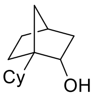

N8-3

White solid, $\mathbf{R_f}$ = 0.49 (Cy / Et_2O = 1:1). **^{1}H NMR** (400 MHz, $CDCl_3$) δ 3.70 (dt, *J* = 7.0, 1.8 Hz, 1H), 2.19 – 2.11 (m, 1H), 1.85 – 1.63 (m, 6H), 1.58 (ddt, *J* = 12.4, 3.5, 2.0 Hz, 1H), 1.48 – 1.41 (m, 3H), 1.39 – 1.08 (m, 7H), 1.06 – 0.93 (m, 1H), 0.81 (ddd, *J* = 9.4, 7.2, 2.1 Hz, 1H). **^{13}C NMR** (101 MHz, $CDCl_3$) δ 75.05, 54.90, 42.95, 36.97, 36.78, 35.14, 30.02, 29.29, 28.79, 27.06, 27.04, 26.91, 25.18. **HRMS** (FI) calculated for $C_{13}H_{22}O$ $[M]^+$: 194.1671; found: 194.1676. **IR** (neat): ν_{max} (cm^{-1}): 3358, 2921, 2867, 2850, 1448, 1333, 1298, 1263, 1242, 1213, 1122, 1075, 1062, 1027, 1010, 946, 890, 862, 845.

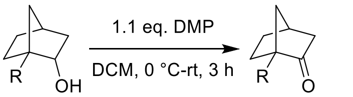

N7-4 99% yield
N8-4 95% yield

N7-4 (R = Hex): To an open flask charged with **N7-3** (2.17 g, 11.07 mmol, 1.0 eq.) and DCM (11 mL, 1 M) at 0 °C, Dess-Martin periodinane (5.05 g, 11.9 mmol, 1.075 eq.) was slowly added and stirred for 3 h. After removal of the solvent under reduced pressure, hexane was added to the residue and filtered through a pad of silica gel. The hexane was discarded and then the silica was rinsed with Et_2O (250 mL), and the crude was concentrated under reduced pressure to provide **N7-4** as a yellow oil (99% yield, 2.13 g, 10.95 mmol), which was used in the next step without further purification.

N8-4 (R = Cy): Following the same procedure using **N8-3** (9.4 g, 46.5 mmol), **N8-4** was obtained as an orange oil (95% yield, 8.5g, 44.2 mmol), which was used in the next step without further purification.

1-Hexylbicyclo[2.2.1]heptan-2-one (N7-4)

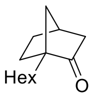

N7-4

Clear oil, **^{1}H NMR** (400 MHz, $CDCl_3$) δ 2.57 (t, *J* = 4.2 Hz, 1H), 2.10 (ddd, *J* = 17.7, 4.4, 2.7 Hz, 1H), 1.99 – 1.91 (m, 1H), 1.90 – 1.77 (m, 1H), 1.74 – 1.14 (m, 15H), 0.93 – 0.83 (m, 3H). ^{1}H NMR of the isolated material matched with that reported in the literature.[12]

1-Cyclohexylbicyclo [2.2.1]heptan-2-one (N8-4)

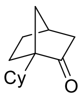

N8-4

Pale yellow solid, **R_f** = 0.45 (Cy / Et_2O = 1:1) ($KMnO_4$ stain). **^{1}H NMR** (400 MHz, $CDCl_3$) δ 2.54 (dq, *J* = 4.7, 1.9 Hz, 1H), 2.07 (ddd, *J* = 17.6, 4.5, 2.0 Hz, 1H), 1.95 (dd, *J* = 17.6, 4.2 Hz, 1H), 1.88 – 1.75 (m, 3H), 1.76 – 1.67 (m, 3H), 1.67 – 1.62 (m, 1H), 1.60 (dp, *J* = 10.1, 2.4 Hz, 2H), 1.53 – 1.41 (m, 2H), 1.35 – 1.21 (m, 3H), 1.20 – 1.11 (m, 2H), 1.01 (qd, *J* = 12.4, 3.4 Hz, 1H). ^{13}C NMR (126 MHz, $CDCl_3$) δ 218.17, 61.46, 47.21, 39.33, 36.77, 33.82, 29.64, 28.82, 28.30, 26.93, 26.87, 26.74, 26.65. **HRMS** (FI) calculated for $C_{13}H_{20}O$ $[M]^+$: 192.1514; found: 192.1515. **IR (neat):** ν_{max} (cm^{-1}): 2923, 2851, 1740, 1449, 1408, 1294, 1170, 1063, 964.

A procedure described in the literature was adapted for the synthesis of **N7-5** and **N8-5**.[13]

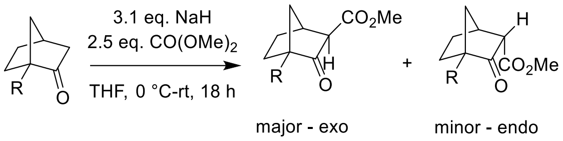

N7-5 68% yield, 1.6:1 dr
N8-5 66% yield, 1.8:1 dr

N7-5 (R = Hex): In a pre-dried round bottom flask under nitrogen atmosphere was added NaH (1.2 g (60% in oil), 30.4 mmol, 3.1 eq.) and anhydrous THF (6 mL, 1.6 M). The mixture was treated with dimethyl carbonate (2.05 mL, 24.3 mmol, 2.5 eq.) and refluxed. A solution of **N7-4** (1.89 g, 9.7 mmol, 1 eq.) in THF (5 mL) was added dropwise to the reaction mixture and refluxed overnight. Afterwards, the reaction was cooled to 0 °C, aq. AcOH (10 mL, 3 M) was slowly added, and the mixture was poured into brine (20 mL). The mixture was extracted with Et_2O (× 3) and the combined organic layers were dried over anhydrous $MgSO_4$, filtered and concentrated under reduced pressure. The crude was purified by flash column chromatography (Hex / Et_2O = 4:1, visualization on TLC with $KMnO_4$) to yield **N7-5** as a light brown oil as a 1.6:1 mixture of diastereomers (68% yield, 1.6:1 dr, 1.68 g, 6.6 mmol).

N8-5 (R = Cy): Following the same procedure using **N8-4** (8.5 g, 44.2 mmol), **N8-5** was obtained as a light brown oil as a 1.8:1 mixture of diastereomers (66% yield, 1.8:1 dr, 7.3 g, 29.2 mmol).

Methyl-4-hexyl-3-oxobicyclo[2.2.1]heptane-2-carboxylate (N7-5)

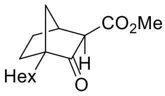

N7-5-1
CO_2Me - *exo*

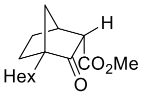

N7-5-2
CO_2Me - *endo*

exo:endo 1.5:1

Brown oil, **R_f** = 0.22 (Hex / Et_2O = 4:1). A mixture of two diastereomers was obtained in a ratio of a:b 1.5:1 dr. **^{1}H NMR** (400 MHz, $CDCl_3$) δ 3.72 (s, $3H_b$), 3.71 (s, $3H_a$).3.06 (d, *J* = 4.1 Hz, $1H_b$), 2.96 (d, *J* = 3.7 Hz, $1H_a$), 2.86 (t, *J* = 3.9 Hz, $1H_b$), 2.82 (d, *J* = 4.2 Hz, $1H_a$), 2.09 (dd, *J* = 10.7, 1.8 Hz, 1_a), 1.97 – 1.63 (m, 3 H_a + 4 H_b), 1.55-1.62 (m, $2H_{a+b}$), 1.53 – 1.37 (m, $3H_{a+b}$), 1.27 (s, $9H_{a+b}$), 0.92 – 0.83 (m, $3H_{a+b}$). **^{13}C NMR** (101 MHz, $CDCl_3$) δ 210.95_a, 210.73_b, 169.40_b, 168.53_a, 60.20_a, 58.90_b, 58.82_b, 57.38_a, 52.48_a, 52.15_b, 40.32_b, 39.57_a, 38.46_a, 38.21_b, 31.84_a, 30.11_b, 30.09_b, 30.08_a, 29.57_b, 28.96_b, 28.77_a, 28.42_a, 25.82_b, 25.80_a, 24.51_{a+b}, 22.76_a, 22.73_b, 14.22_{a+b}. **HRMS** (FI): *m/z* calculated for $C_{15}H_{24}O_3$ $[M]^+$= 252.1725; found= 252.1713. **IR** (neat): ν_{max} (cm^{-1}): 2954, 2928, 2857, 1758, 1726, 1455, 1434, 1334, 1292, 1254, 1196, 1162, 959.

Methyl-4-cyclohexyl-3-oxobicyclo[2.2.1]heptane-2-carboxylate (N8-5)

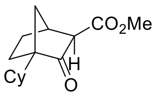

N8-5-1
CO_2Me - *exo*

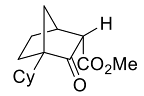

N8-5-2
CO_2Me - *endo*

exo:endo 1.5:1

Brown oil, **R_f** = 0.30 (Cy / Et_2O = 4:1). A mixture of two diastereomers was obtained in a ratio of a:b 1.5:1. **^{1}H NMR** (400 MHz, $CDCl_3$) δ 3.72 (s, $3H_a$), 3.70 (s, $3H_b$), 3.04 (dd, *J* = 4.5, 1.7 Hz, $1H_b$), 2.95 (d, *J* = 3.6 Hz, $1H_a$), 2.83 (dq, *J* = 4.5, 2.1 Hz, $1H_b$), 2.78 (dt, *J* = 3.6, 1.8 Hz, $1H_a$), 2.09 (dq, *J* = 10.6, 2.1 Hz, $1H_a$), 1.91 – 1.81 (m, $2H_a$ +$3H_b$), 1.78 – 1.62 (m, $5H_{a+b}$), 1.60 – 1.31 (m, $4H_{a+b}$), 1.29 – 1.10 (m, $4H_{a+b}$), 1.01 (qdd, *J* = 12.4, 3.4, 2.1 Hz, $1H_{a+b}$). **^{13}C NMR** (101 MHz, $CDCl_3$) δ 210.94_a, 210.48_b, 169.43_b, 168.62_a, 62.27_{a+b}, 60.81_a, 59.56_b, 52.42_a, 52.10_b, 38.56_b, 38.16_a, 37.87_b, 37.72_a, 37.11_b, 36.86_a, 29.40_a, 29.35_b, 28.78_a, 28.71_b, 28.19_{a+b}, 26.84_a, 26.83_a, 26.79_a, 26.76_b, 26.65_a, 26.65_b, 26.42_b, 24.31_b. **HRMS** (FI) calculated for $C_{15}H_{22}O_3$ $[M]^+$: 250.1569; found: 250.1569. **IR (neat):** ν_{max} (cm^{-1}): 2924, 2851, 1755, 1723, 1449, 1434, 1334, 1292, 1245, 1195, 1161, 960, 944.

A procedure described in the literature was adapted for the last three steps of the synthesis of **N7** and **N8**.[10]

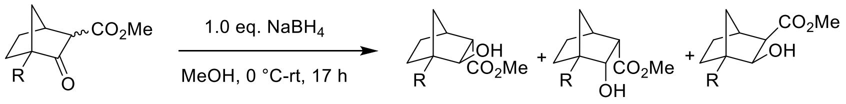

N7-6 93% yield, 6:4:1 dr
N8-6 92% yield, 1.5:1.5:1 dr

N7-6 (R = Hex): To a solution of **N7-5** (1.67 g, 6.6 mmol, 1.0 eq.) in methanol (18.8 mL, 0.35 M) at 0 °C, $NaBH_4$ (0.252 g, 6.6 mmol, 1.0 eq.) was slowly added and the reaction mixture was stirred overnight (17 h). 5% aq. HCl was added to the reaction mixture, and the mixture was extracted with Et_2O (× 3). The combined organic layers were dried over anhydrous $MgSO_4$, filtered and the solvent removed under reduced pressure to provide **N7-6** as a yellow oil as mixture of diastereomers (93%, 6:4:1 dr, 1.57 g, 6.1 mmol), which was used in the next step without further purification.

N8-6 (R = Cy): Following the same procedure, but adding an additional 0.8 eq. of $NaBH_4$ 4 h after the first addition of $NaBH_4$, **N8-6** was obtained as an oil as mixture of diastereomers (92% yield, 1.5:1.5:1 dr, 6.8 g, 26.9 mmol), which was used in the next step without further purification.

Methyl -4-hexyl-3-oxobicyclo[2.2.1]heptane-2-carboxylate (N7-6)

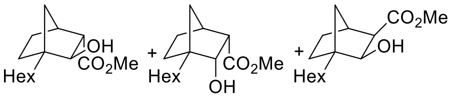

N7-6-1 **N7-6-2** **N7-6-3**

6:4:1 dr

Clear yellow oil, $\mathbf{R_f}$ = 0.48, 0.56, 0.41 (Cy / Et_2O = 1:1). A mixture of three diastereomers was obtained in a ratio of **N7-6-1**:**N7-6-2**:**N7-6-3** 6:4:1. The NMR peaks of diastereomer **N11-6-1** are listed here. **^{1}H NMR** (400 MHz, $CDCl_3$) δ 4.13 (dd, *J* = 4.3, 1.9 Hz, 1H), 3.69 (s, 3H), 2.38 (d, *J* = 4.5 Hz, 1H), 2.15 (dd, *J* = 4.4, 2.6 Hz, 1H), 1.69 (tt, *J* = 12.6, 4.8 Hz, 3H), 1.54 – 1.42 (m, 3H), 1.36 – 1.16 (m, 10H), 0.92 – 0.84 (m, 3H). **^{13}C NMR** (101 MHz, $CDCl_3$) δ 175.48, 79.01, 56.90, 51.94, 51.46, 40.57, 39.92, 32.51, 31.98, 30.96, 30.38, 25.93, 24.41, 22.80, 14.25.

N7-6-2 was isolated pure for characterization purpose by flash column chromatography (Hex / Et_2O = 4:1). **N7-6-2** **^{1}H NMR** (400 MHz, $CDCl_3$) δ 4.52 (bs, 1H), 3.96 (d, *J* = 9.2 Hz, 1H), 3.70 (s, 3H), 2.84 (ddd, *J* = 9.8, 4.6, 1.7 Hz, 1H), 2.42 (td, *J* = 3.9, 3.3, 1.7 Hz, 1H), 1.79 (dddd, *J* = 12.6, 9.1, 5.5, 2.1 Hz, 1H), 1.56 – 1.41 (m, 4H), 1.36 – 1.15 (m, 11H), 0.93 – 0.80 (m, 3H). **^{13}C NMR** (101 MHz, $CDCl_3$) δ 175.48, 74.67, 52.42, 51.75, 46.76, 40.54, 40.27, 33.04, 32.02, 30.38, 25.88, 25.74, 24.55, 22.82, 14.27.

Methyl -4-cyclohexyl-3-oxobicyclo[2.2.1]heptane-2-carboxylate (N8-6)

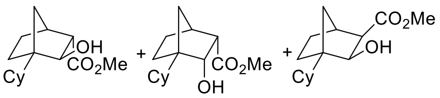

N8-6-1 **N8-6-2** **N8-6-3**

1.5:1.5:1 dr

The crude contained three diastereomers **N8-6-1:N8-6-2:N-8-6-3** in a ratio of 6:4:1. Each diastereomer was isolated pure for characterization purpose by flash column chromatography (Hex / Et_2O = 4:1, visualization on TLC with $KMnO_4$).

N8-6-1: Clear oil, $\mathbf{R_f}$ = 0.48 (Cy / Et_2O =1:1). **^{1}H NMR** (400 MHz, CD_3Cl) δ 4.31 (dd, *J* = 4.1, 1.9 Hz, 1H), 3.68 (s, 3H), 2.39 – 2.32 (m, 1H), 2.13 (dd, *J* = 4.0, 2.8 Hz, 1H), 1.84 (dddd, *J* = 14.5, 12.4, 6.8, 2.7 Hz, 2H), 1.80 – 1.72 (m, 4H), 1.70 – 1.61 (m, 2H), 1.46 (dddd, *J* = 9.3, 7.7, 5.0, 2.6 Hz, 2H), 1.31 (tdd, *J* = 12.6, 4.0, 2.1 Hz, 1H), 1.25 – 1.00 (m, 6H). **^{13}C NMR** (101 MHz, $CDCl_3$) δ 175.50, 77.35, 57.76, 54.81, 51.90, 41.07, 39.99, 38.75, 30.67, 30.03, 29.92, 27.24, 27.06, 26.73, 22.53. **HRMS** (FI) calculated for $C_{15}H_{24}O_3$ $[M]^+$: 252.1725; found: 252.1714. **IR (neat):** ν_{max} (cm^{-1}): 3467, 2923, 2871, 2850, 1731, 1713, 1447, 1435, 1362, 1325, 1298, 1251, 1193, 1171, 1133, 1070, 1041, 982, 952, 922, 890, 850, 752, 559, 504.

N8-6-2: Clear yellow oil, $\mathbf{R_f}$ = 0.56 (Cy / Et_2O =1:1). **^{1}H NMR** (400 MHz, $CDCl_3$) δ 4.43 (bs, 1H), 4.15 (d, *J* = 9.7 Hz, 1H), 3.70 (s, 4H), 2.81 (dd, *J* = 9.7, 4.6 Hz, 1H), 2.40 (s, 1H), 1.86 (dddd, *J* = 12.5, 8.1, 6.0, 2.0 Hz, 2H), 1.78 – 1.68 (m, 3H), 1.67 – 1.61 (m, 1H), 1.55 – 1.38 (m, 3H), 1.27 (tt, *J* = 8.1, 2.4 Hz, 3H), 1.22 – 1.09 (m, 3H), 1.03 (qd, *J* = 12.5, 3.3 Hz, 1H). **^{13}C NMR** (101 MHz, $CDCl_3$) δ 175.31, 73.14, 55.84, 51.72, 47.66, 41.65, 39.89, 39.13, 29.74, 29.72, 27.24, 27.12, 26.79, 25.61, 22.65. **IR (neat):** ν_{max} (cm^{-1}): 3469, 2923, 2876, 2850, 1707, 1438, 1358, 1299, 1263, 1199, 1176, 1134, 1113, 1059, 1045, 997, 939, 752, 496.

N8-6-3: White solid, $\mathbf{R_f}$ = 0.44 (Cy / Et_2O = 1:1). **^{1}H NMR** (400 MHz, $CDCl_3$) δ 3.94 (d, *J* = 6.9 Hz, 1H), 3.68 (s, 3H), 2.68 (dd, *J* = 7.2, 1.6 Hz, 1H), 2.53 (bs, 1H), 2.41 (s, 1H), 1.85 – 1.61 (m, 6H), 1.61 – 1.44 (m, 3H), 1.38 – 1.05 (m, 6H), 1.03 – 0.84 (m, 2H). **^{13}C NMR** (101 MHz, $CDCl_3$) δ 173.84, 75.90, 54.78, 54.39, 51.69, 39.05, 36.86, 36.33, 29.98, 29.11, 28.70, 26.98, 26.94, 26.85, 25.24. **IR (neat):** ν_{max} (cm^{-1}): 3496, 2923, 2873, 2850, 1724, 1448, 1436, 1360, 1294, 1265, 1230, 1195, 1171, 1137, 1075, 1048, 1030.

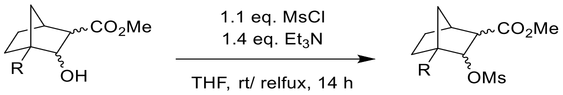

N7-7
N8-7

N7-7 (R = Hex): To a solution of **N7-6** (1.57 g, 6.16 mmol, 1.0 eq.) in THF (30 mL, 0.2 M) at 0 °C, Et_3N (1.2 ml, 8.6 mmol, 1.4 eq.) and methanesulfonyl chloride (0.55 ml, 6.8 mmol, 1.1 eq.) were added. The reaction mixture was stirred at room temperature overnight (14 h) and then the mixture was quenched with water, extracted with EtOAc (× 3) and the combined organic layers were dried over anhydrous $MgSO_4$, filtered and the solvent removed under reduced pressure to obtain a mixture of the mesylated product (**N7-7**) coming from the major starting diastereomer (**N7-6-1**), together with the unreacted two minor diastereomers of the **N7-6-2** and **N7-6-3** (1.8 g, **N7-7**: **N7-6** 1.5:1). The mixture was used in the next step without purification

N8-7 (R = Cy): Following the same procedure, but stirring overnight under reflux, the mesylated product (**N8-7**) coming from the major starting diastereomer (**N8-6-1**) together with the unreacted two minor diastereomers of the alcohol **N8-6-2** and **N8-6-3** were obtained. The mixture was purified by flash column chromatography (Hex / Et_2O = 9:1) to yield **N8-7** as a clear oil (35% yield, 3.0 g, 9.2 mmol) [Note: It is also possible to use the crude mixture in the next step].

Methyl-4-cyclohexyl-3-((methylsulfonyl)oxy)bicyclo[2.2.1]heptane-2-carboxylate (N8-7)

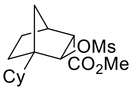

N8-7

Clear oil, **R$_f$** = 0.22 (Cy / Et_2O = 4:1). **^{1}H NMR** (400 MHz, $CDCl_3$) δ 5.17 (dd, *J* = 3.5, 2.0 Hz, 1H), 3.71 (s, 3H), 3.02 (s, 3H), 2.52 (t, *J* = 3.1 Hz, 1H), 2.40 (dd, *J* = 4.1, 1.9 Hz, 1H), 1.90 – 1.68 (m, 6H), 1.69 – 1.63 (m, 1H), 1.62 – 1.49 (m, 3H), 1.41 (tdd, *J* = 12.1, 4.2, 2.0 Hz, 1H), 1.29 – 1.02 (m, 6H). **^{13}C NMR** (101 MHz, $CDCl_3$) δ 173.98, 84.18, 55.13, 55.06, 52.31, 40.86, 40.36, 38.06, 37.50, 30.30, 29.63, 29.51, 27.09, 26.89, 26.61, 23.21. **HRMS** (FI) calculated for $C_{16}H_{26}O_5S$ $[M]^+$: 330.1501; found: 330.1486. **IR** (neat): v_{max} (cm^{-1}): 2926, 2852, 1729, 1449, 1437, 1355, 1301, 1256, 1198, 1171, 952, 918, 898, 846, 821, 759, 544, 528.

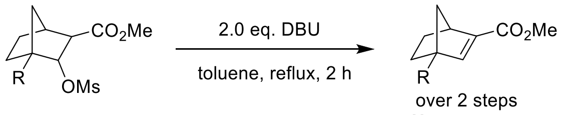

over 2 steps
N7 45% yield
N8 28% yield

N7 (R = Hex): N7-7 (mixture of **N7-7**: **N7-6** 1.5:1) (1.8 g, 5.4 mmol, 1 eq.) was dissolved in toluene (33 mL, 0.2 M) and 1,8-diazabicyclo[5.4.0]undeca-7-ene (DBU) (1.6 mL, 11 mmol, 2.0 eq.) was added and the reaction mixture was refluxed for 2 h. After the reaction was cooled to room temperature, 5% aq. HCl was added to the reaction mixture, extracted with Et_2O (× 3) and the combined organic layers were dried over anhydrous $MgSO_4$ and the solvent was removed under reduced pressure. The crude was purified by flash column chromatography (Hex / Et_2O = 15:1) to yield **N7** as a clear oil (45% yield over two steps, 0.652 g, 2.75 mmol).

N8 (R = Cy): N8-7 (3.0 g, 9.2 mmol, 1 eq.) was dissolved in toluene (46 mL, 0.2 M) and 1,8-diazabicyclo[5.4.0]undeca-7-ene (DBU) (2.75 mL, 18.4 mmol, 2.0 eq.) was added and the reaction mixture was refluxed for 2 h. After the reaction was cooled to room temperature, water and 5% aq. HCl were added to the reaction mixture, extracted with Et_2O (x 3) and the combined organic layers were dried over anhydrous $MgSO_4$ and the solvent was removed under reduced pressure. The crude was purified by flash column chromatography (Hex / Et_2O = 15:1) to yield **N8** as clear oil (yield 81%, 1.73 g, 7.4 mmol).

Methyl-4-hexylbicyclo[2.2.1]hept-2-ene-2-carboxylate (N7)

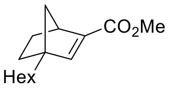

N7

Clear oil, **R$_f$** = 0.43 (Cy / Et_2O = 10:1). **^{1}H NMR** (400 MHz, $CDCl_3$) δ 6.81 (s, 1H), 3.72 (s, 3H), 3.20 (s, 1H), 1.89 – 1.79 (m, 1H), 1.67 (ddd, *J* = 15.9, 9.8, 5.7 Hz, 1H), 1.60 – 1.51 (m, 1H), 1.44 – 1.24 (m, 10H), 1.21 – 1.06 (m, 3H), 0.92 – 0.85 (m, 3H). **^{13}C NMR** (101 MHz, $CDCl_3$) δ 165.45, 150.04, 140.72, 56.40, 51.80, 51.43, 42.49, 33.11, 31.98, 30.67, 30.20, 26.95, 26.93, 22.78, 14.26. **HRMS** (FI) calculated for $C_{15}H_{24}O_2$ $[M]^+$: 236.1776; found: 236.1775. **IR** (neat): v_{max} (cm^{-1}): 2952, 2923, 2857, 1715, 1600, 1435, 1343, 1319, 1278, 1256, 1223, 1184, 1118, 1071, 876, 773, 753.

Methyl-4-cyclohexylbicyclo[2.2.1]hept-2-ene-2-carboxylate (N8)

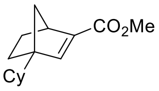

N8

Clear oil, **R$_f$** = 0.52 (Cy / Et_2O = 15:1). **^{1}H NMR** (400 MHz, $CDCl_3$) δ 6.91 (s, 1H), 3.72 (s, 3H), 3.19 (s, 1H), 1.86 – 1.73 (m, 5H), 1.72 – 1.66 (m, 1H), 1.61 (ddd, *J* = 11.5, 9.4, 3.4 Hz, 1H), 1.57 – 1.51 (m, 1H), 1.44 (dq, *J* = 8.4, 2.3 Hz, 1H), 1.35 – 0.96 (m, 8H). **^{13}C NMR** (101 MHz, $CDCl_3$) δ 165.41, 148.67, 140.60, 60.76, 51.40, 49.67, 42.15, 40.35, 30.55, 30.02, 28.06, 26.84, 26.77, 26.74. **HRMS** (FI) calculated for $C_{15}H_{22}O_2$ $[M]^+$: 234.1620; found: 234.1610. **IR** (neat): v_{max} (cm^{-1}): 2922, 2851, 1714, 1447, 1435, 1317, 1278, 1261, 1189, 1122, 1075, 754.

4.1.3. Synthesis of Norbornenes N9 and N10

A procedure described in the literature was adapted for the synthesis of **N9** and **N10**.[7]

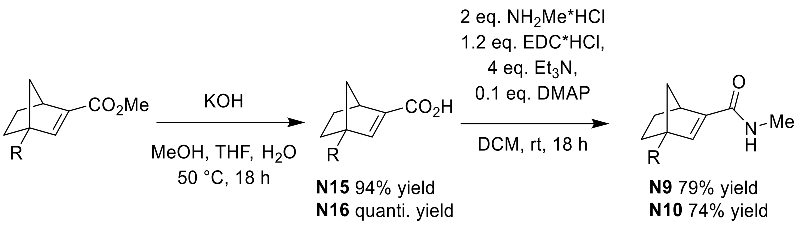

N15 (R = Hex): N7 (0.398 g, 1.7 mmol, 1 eq.) was dissolved in THF (4.3 mL) and methanol (1.7 mL) was added. While stirring at room temperature, 30 % aq. KOH (17 mL) was added. The resulting solution was stirred at 50 °C overnight (18 h). After cooling the solution to room temperature, the mixture was diluted with water and the aqueous layer washed with Et_2O. The water layer was acidified with 2 M aq. HCl and extracted with DCM (× 3) and the combined organic layers were dried over anhydrous $MgSO_4$ and the solvent was removed under reduced pressure to give **N15** as a clear oil (94 % yield, 0.355 g, 1.6 mmol), which was used in the next step without further purification.

N16 (R = Cy): Following the same procedure using **N8** (0.468 g, 2 mmol), **N16** was obtained as a clear oil (quantitative yield, 0.441 g, 2 mmol), which was used in the next step without further purification.

4-Hexylbicyclo[2.2.1]hept-2-ene-2-carboxylic acid (N15)

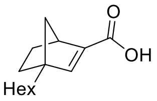

N15

Clear oil, $\mathbf{R_f}$ = 0.34 (Cy / Et_2O = 10:1). **^{1}H NMR** (400 MHz, $CDCl_3$) δ 6.96 (s, 1H), 3.20 (dd, J = 3.2, 1.6 Hz, 1H), 1.85 (ddt, J = 10.8, 8.5, 3.5 Hz, 1H), 1.77 – 1.63 (m, 2H), 1.62 – 1.55 (m, 1H), 1.49 – 1.25 (m, 9H), 1.24 – 1.09 (m, 3H), 0.93 – 0.84 (m, 3H). ^{13}C NMR (101 MHz, $CDCl_3$) δ 169.69, 152.85, 140.21, 56.75, 51.81, 42.25, 33.02, 31.97, 30.61, 30.19, 26.96, 26.85, 22.78, 14.25. **HRMS** (ESI) calculated for $C_{15}H_{24}O_2$ $[M+H]^+$: 223.1698; found: 223.1689. **IR** (neat): ν_{max} (cm^{-1}): 2954, 2923, 2856, 1677, 1601, 1418, 1318, 1283, 1267, 1191, 931, 881, 760, 743.

4-Cyclohexylbicyclo[2.2.1]hept-2-ene-2-carboxylic acid (N16)

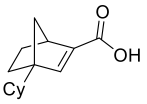

N16

Clear oil, **^{1}H NMR** (400 MHz, $CDCl_3$) δ 7.06 (s, 1H), 3.23 – 3.18 (m, 1H), 1.88 – 1.74 (m, 5H), 1.73 – 1.63 (m, 2H), 1.56 (tt, J = 11.9, 3.2 Hz, 1H), 1.47 (dd, J = 8.6, 2.3 Hz, 1H), 1.32 – 1.00 (m, 8H). **^{13}C NMR** (101 MHz, $CDCl_3$) δ 169.80, 151.57, 140.10, 61.11, 49.65, 41.88, 40.27, 30.56, 30.01, 27.98, 26.81, 26.71, 26.67 **HRMS** (ESI) calculated for $C_{14}H_{20}O_2$ $[M+H]^+$: 221.1542; found: 221.1539. **IR** (neat): ν_{max} (cm^{-1}): 2924, 2852, 1678, 1600, 1448, 1420, 1316, 1285, 1270, 761. **M.P.** = 66−70 °C.

N9 (R = Hex): To an open flask, **N16** (286 mg, 1.25 mmol, 1 eq.), methylammonium chloride (172 mg, 2.5 mmol, 2 eq.), 1-ethyl-3-(3′-dimethylaminopropyl)carbodiimide HCl (288 mg, 1.5 mmol, 1.2 eq.), triethylamine (0.7 mL, 5 mmol, 4.0 eq.), 4-(dimethylamino)pyridine (15.3 mg, 0.125 mmol, 0.1 eq.) and DCM (5 mL, 0.25 M) were added. The mixture was stirred overnight (18 h) at room temperature and then diluted with DCM and washed with water. The organic layer was dried over anhydrous $MgSO_4$ and concentrated under reduced pressure. The crude product was purified by flash column chromatography (Cy / EtOAc = 4:1) to yield **N9** as a clear oil (79 % yield, 231 mg, 0.98 mmol).

N10 (R = Cy): Following the same procedure using **N16** (660 mg, 3 mmol), **N10** was obtained as a white solid (74 % yield, 514.2 mg, 2.2 mmol).

4-Hexyl-N-methylbicyclo[2.2.1]hept-2-ene-2-carboxamide (N9)

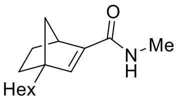

N9

White oil, $\mathbf{R_f}$ = 0.14 (Cy / EtOAc = 4:1). **^{1}H NMR** (400 MHz, $CDCl_3$) 6.51 (s, 1H), 5.67 (s, 1H), 3.07 (dd, J = 3.6, 1.6 Hz, 1H), 2.86 (dd, J = 5.0, 1.4 Hz, 3H), 1.84 (tdd, J = 9.6, 4.7, 2.0 Hz, 1H), 1.75 – 1.61 (m, 2H), 1.60 – 1.52 (m, 1H), 1.43 – 1.25 (m, 9H), 1.24 – 1.17 (m, 1H), 1.15 – 1.07 (m, 2H), 0.88 (t, J = 6.0 Hz, 3H). **^{13}C NMR** (101 MHz, $CDCl_3$) δ 165.55, 143.73, 143.34, 55.90, 51.78, 42.57, 33.07, 31.85, 30.86, 30.07, 27.02, 26.78, 26.13, 22.63, 14.11. **HRMS** (FI) calculated for $C_{15}H_{25}NO$ $[M]^+$: 235.1936; found: 235.1938. **IR** (neat): ν_{max} (cm^{-1}): 3309, 2953, 2923, 2857, 1633, 1588, 1536, 1466, 1408, 1325, 1287, 1154, 753, 723, 699, 669.

4-Cyclohexyl-N-methylbicyclo[2.2.1]hept-2-ene-2-carboxamide (N10)

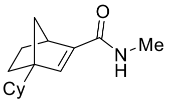

N10

White solid, $\mathbf{R_f}$ = 0.14 (Cy / EtOAc = 4:1). **^{1}H NMR** (400 MHz, $CDCl_3$) δ 6.60 (s, 1H), 5.85 (bs, 1H), 3.06 (dd, J = 3.5, 1.7 Hz, 1H), 2.83 (d, J = 4.9 Hz, 3H), δ 1.94 – 1.71 (m, 3H), 1.69 – 1.63 (m, 1H), 1.62 – 1.56 (m, 1H), 1.55 – 1.46 (m, 1H), 1.41 (dq, J = 8.3, 2.2 Hz, 1H), 1.30 – 0.90 (m, 9H). **^{13}C NMR** (101 MHz, $CDCl_3$) δ 165.69, 143.64, 142.16, 60.31, 49.73, 42.30, 40.39, 30.48, 29.94, 28.34, 26.94, 26.81, 26.71, 26.25. **HRMS** (FI) calculated for $C_{15}H_{23}NO$ $[M]^+$: 233.1780; found: 233.1790. **IR** (neat): ν_{max} (cm^{-1}): 3307, 2924, 2851, 1634, 1589, 1538, 1448, 1408, 1346, 1321, 1287, 1154. **M.P.** = 113−115 °C.

4.1.4. Synthesis of Norbornene N11

Scheme S2. Synthesis of **N11**.

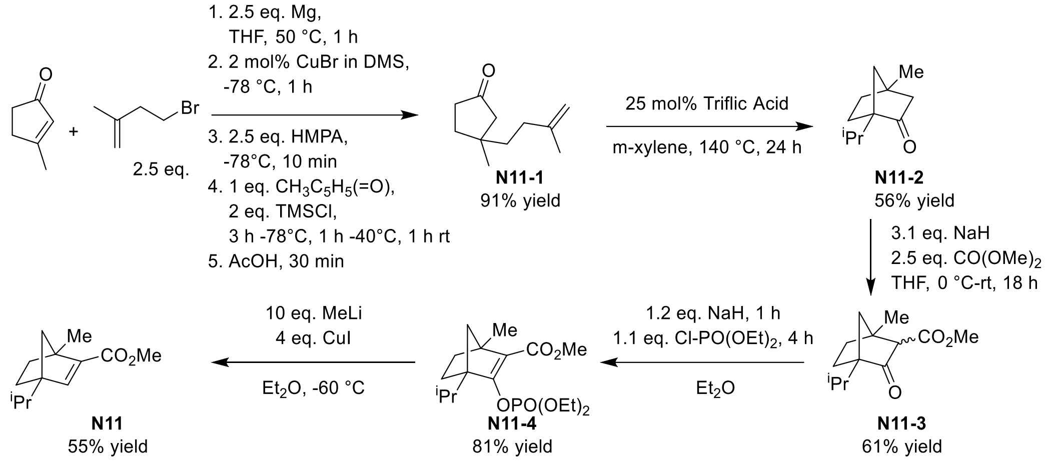

A procedure described in the literature was adapted for the synthesis of 4-bromo-2-methylbutene.[14]

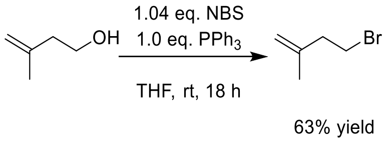

Triphenylphosphine (10.5 g, 40 mmol, 1.0 eq.) was dissolved in anhydrous THF (40 mL, 1M) under nitrogen atmosphere and 3-methylbut-3-enol (4.2 mL, 40 mmol, 1 eq.) was added followed by the slow addition in portions over 15 min of *N*-bromo succinimide (7.4 g, 41.6 mmol, 1.04 eq.). The reaction was stirred at room temperature overnight (18 h) and then, the mixture was diluted with pentane. The suspension was filtered through a pad of silica, rinsed with pentane and the solvent was removed under reduced pressure. The crude product was purified by distillation (85 °C, 340 mbar, 100-140 °C oil bath) to yield the product as a clear oil (63% yield, 3.75g, 25.2 mmol).

4-Bromo-2-methylbutene

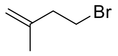

Clear oil, **^{1}H NMR** (400 MHz, $CDCl_3$) δ 4.82 (d, *J* = 33.9 Hz, 2H), 3.48 (t, *J* = 7.4 Hz, 2H), 2.58 (t, *J* = 7.4 Hz, 2H), 1.75 (s, 3H). ^{1}H NMR of the isolated material matched with that reported in the literature. [14]

A procedure described in the literature was adapted for **N11-1** and **N11-2**.[15]

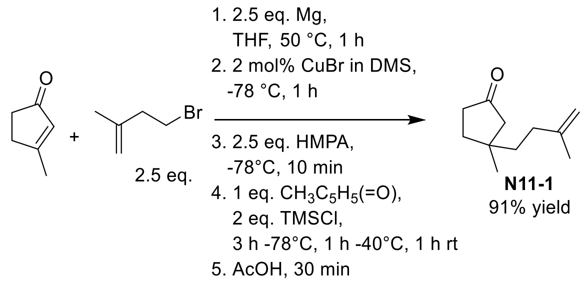

A dry three-neck round-bottom flask under nitrogen atmosphere was charged with magnesium (0.911 g, 37.5 mmol, 2.5 eq.) and anhydrous THF (45 mL). 4-Bromo-2-methylbutene (5.59 g, 37.5 mmol, 2.5 eq) was added dropwise and the reaction mixture was stirred at 50 °C for 1 h. Then, the reaction was cooled to -78 °C and a previously prepared solution of CuBr in DMS (108 mg CuBr in 2.4mL dimethylsulfide) was added and the resulting mixture stirred for an additional hour. After, HMPA (6.5 mL, 37.5 mmol, 2.5 eq.) was added at -78 °C and stirred for 10 min. Then, a solution of 3-methyl-2-cyclopentenone (1.52 mL, 15 mmol, 1 eq.) and TMS-Cl (3.0 mL, 30 mmol, 2 eq.) in THF (40 mL) was added via cannular transfer to the reaction mixture at -78 °C and stirred for 2 h. The mixture was allowed to warm to -40 °C and after 30min was left to warm to room temperature and stirred for additional 30 min. Finally, acetic acid (7.2 mL, 126 mmol) was added over a period of 30 min. The mixture was diluted with Et_2O (36 mL) and sat. NH_4Cl (18 mL). The aqueous layer was adjusted to pH 9 using 30% NH_4OH, and the organic layer was separated, and the water layer was extracted with Et_2O (× 3). The combined organic layers were washed with brine, dried over anhydrous $MgSO_4$, filtered and concentrated under

reduced pressure. The crude was purified by flash column chromatography (Hex / EtOAc = 10:1, visualization on TLC with $KMnO_4$ solution) to yield **N11-1** a clear oil (91% yield, 2.274 g, 13.65 mmol).

3-Methyl-3-(3-methylbut-3-en-1-yl)cyclopentanone (N11-1)

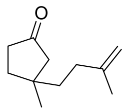

N11-1

Clear oil, **R_f** = 0.49 (Cy / EtOAc) = 9:1). **^{1}H NMR** (300 MHz, $CDCl_3$) δ 4.70 (d, *J* = 5.8 Hz, 2H), 2.47 – 2.19 (m, 2H), 2.13 – 1.91 (m, 4H), 1.89 – 1.76 (m, 2H), 1.73 (s, 3H), 1.63 – 1.46 (m, 2H), 1.07 (s, 3H). ^{1}H NMR of the isolated material matched with that reported in the literature.[15]

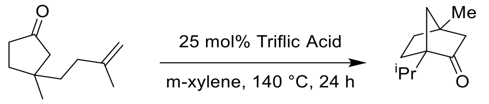

N11-2
56% yield

N11-1 (6.1 g, 36.6 mmol, 1.0 eq.) was dissolved in *m*-xylene (970 mL, 0.1 M) and triflic acid (0.81 mL, 9.15 mmol, 0.25 eq.) was added and the reaction mixture was refluxed (140 °C) for 24 h. After, the reaction was cooled to room temperature, the mixture was filtered through a short pad of silica and the silica was rinsed with DCM. The solvent was removed under reduced pressure and the crude purified by flash column chromatography (Hex / EtOAc = 10:1, visualization on TLC with phosphomolybdic stain) to yield **N11-2** a yellow oil (56% yield, 3.42 g, 20.6 mmol).

1-Isopropyl-4-methylbicyclo[2.2.1]heptan-2-one (N11-2)

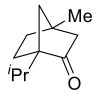

N11-2

Clear oil, **R_f** = 0.33 (Cy / Et_2O = 10:1) (phosphomolybdic stain). **^{1}H NMR** (400 MHz, $CDCl_3$) δ 2.07 – 1.80 (m, 4H), 1.59 (tdq, *J* = 9.5, 4.8, 2.4, 1.7 Hz, 1H), 1.51 – 1.42 (m, 3H), 1.39 – 1.30 (m, 1H), 1.23 (s, 3H), 0.95 (d, *J* = 6.9 Hz, 3H), 0.92 (d, *J* = 6.9 Hz, 3H). ^{1}H NMR of the isolated material matched with that reported in the literature.[15]

A procedure described in the literature was adapted for the synthesis of **N11-3**.[13]

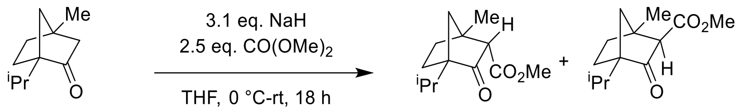

N11-3
61% yield, 4:1 dr

A pre-dried reflux set up under nitrogen atmosphere was charged with NaH [0.6 g (60% in oil), 15 mmol, 3.0 eq.], anhydrous THF (3.15 mL, 1.6 M), dimethyl carbonate (1.05 mL, 12.5 mmol, 2.5 eq.) and the mixture was heated to reflux. Then, a solution of **N11-2** (0.83 g, 5 mmol, 1eq.) in anhydrous THF (2.3 mL) was added dropwise to the reaction mixture and stirred under reflux overnight (18 h). The reaction was cooled to 0 °C and. AcOH (10 mL, 3 M aq.) was slowly added and the mixture was poured into brine. The mixture was extracted with DCM (x 3) and the combined organic layers were dried over anhydrous $MgSO_4$, filtered and concentrated under reduced pressure. The crude was purified by flash column chromatography (Hex / EtOAc = 5:1, visualization on TLC with $KMnO_4$ stain) to yield **N11-3** as a light brown oil as a 4:1 mixture of diastereomers (61% yield, 4:1 dr, 0.684 g, 3.0 mmol). Each diastereomers was isolated pure for characterization purpose.

Methyl-4-isopropyl-1-methyl-3-oxobicyclo[2.2.1]heptane-2-carboxylate (N11-3)

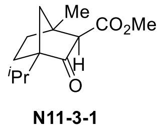

N11-3-1

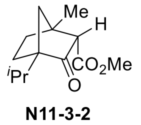

N11-3-2

dr 1:4

The crude contained two diastereomers **N11-3-1:N11-3-2** in a ratio of 1:4. Each diastereomer was isolated pure for characterization purpose by flash column chromatography (Hex / EtOAc = 5:1).

N11-3-1: Clear oil, **R_f** = 0.58 (Cy / Et_2O = 1:1). **^{1}H NMR** (400 MHz, $CDCl_3$) δ 3.69 (s, 3H), 2.94 (d, *J* = 3.3 Hz, 1H), 2.25 (dt, *J* = 10.5, 2.4 Hz, 1H), 2.04 (p, *J* = 6.9 Hz, 1H), 1.89 (td, *J* = 12.7, 4.3 Hz, 1H), 1.68 (td, *J* = 12.4, 5.1 Hz, 1H), 1.55 – 1.48 (m, 1H), 1.43 (dd, *J* = 10.5, 3.3 Hz, 1H) 1.44 – 1.34 (m, 1H), 1.21 (s, 3H), 0.96 (dd, *J* = 8.3, 6.9 Hz, 6H). **^{13}C NMR** (101 MHz, $CDCl_3$) δ 212.11, 169.29, 64.71, 62.72, 52.06, 45.18, 42.14, 37.07, 27.76, 26.76, 19.27, 18.67, 18.59.

N11-3-2: Clear oil, **R_f** = 056. (Cy / Et_2O = 1:1). **^{1}H NMR** (400 MHz, $CDCl_3$) δ 3.73 (s, 3H), 2.86 (d, *J* = 1.8 Hz, 1H), 2.02 (p, *J* = 6.9 Hz, 1H), 1.96 – 1.84 (m, 2H), 1.54 (d, *J* = 10.3 Hz, 1H), 1.60 – 1.40 (m, 2H), 1.43 (dt, *J* = 10.3, 2.2 Hz, 1H), 1.31 (s, 3H), 0.97 (d, *J* = 6.9 Hz, 3H), 0.93 (d, *J* = 6.9 Hz, 3H). **^{13}C NMR** (101 MHz, $CDCl_3$) δ 210.96, 169.19, 64.75, 63.70, 52.05, 45.71, 45.25, 30.95, 28.20, 27.03, 20.62, 19.30, 18.55.

A procedure described in the literature was adapted for the synthesis of **N11-4**.[13]

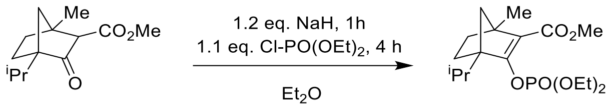

N11-4
81% yield

To a suspension of NaH [93.3 mg (60% in oil], 2.3 mmol, 1.2 eq.) and anhydrous Et_2O (0.5 mL) at 0 °C, a solution of **N11-3** (436 mg, 1.95 mmol, 1 eq.) in Et_2O (0.5 mL) was added dropwise under nitrogen atmosphere and the mixture was stirred for 1 h at room temperature. Then, diethyl chlorophosphite (309 µL, 2.14 mmol, 1.1 eq.) was added and the mixture was stirred for 4 h at room temperature. The reaction mixture was filtered through a short pad of Celite®, rinsed with Et_2O and concentrated under reduced pressure. The crude was purified by flash column chromatography (Hex / EtOAc = 5:1) to yield **N11-4** as a clear oil (81% yield, 0.570 g, 1.58 mmol).

Methyl -3-((diethoxyphosphoryl)oxy)-4-isopropyl-1-methylbicyclo[2.2.1]hept-2-ene-2-carboxylate (N11-4)

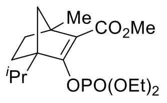

N11-4

Clear oil, **R$_f$** = 0.04 (Cy / EtOAc = 5:1). **^{1}H NMR** (400 MHz, $CDCl_3$) δ 4.25 – 4.10 (m, 4H), 3.73 (s, 3H), 2.20 (hept, *J* = 6.8 Hz, 1H), 2.01 – 1.88 (m, 1H), 1.64 – 1.53 (m, 2H), 1.49 – 1.39 (m, 4H), 1.34 (qd, *J* = 7.0, 1.2 Hz, 6H), 1.29 – 1.23 (m, 1H), 1.08 – 1.02 (m, 1H), 0.99 (d, *J* = 6.8 Hz, 3H), 0.94 (d, *J* = 6.8 Hz, 3H).

A procedure described in the literature was adapted for the synthesis of **N11**.[16]

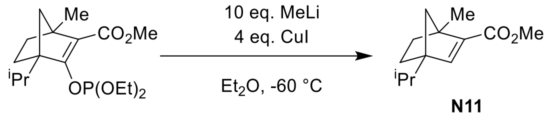

N11
55% yield

To a dried Schlenk flask charged with copper iodide (371.4 mg, 1.95 mmol, 3.9 eq.) and anhydrous and oxygen free Et_2O (5 mL) at -78 °C, a solution of MeLi (3.13 mL, 5 mmol, 10 eq., 1.6 M in Et_2O) was added dropwise and the mixture was stirred for 30 min at -78 °C. Then, a solution of **N11-4** (180 mg, 0.5 mmol, 1.0 eq.) in anhydrous Et_2O (3 mL) was added and the resulting mixture was stirred for 3 h between -60/ -50 °C. The mixture was poured on ice cooled sat. NH_4Cl solution and extracted with Et_2O (× 3). The combined organic layers were washed with 10% ammonium hydroxide solution and brine, dried over anhydrous $MgSO_4$, filtrated and concentrated under reduced pressure. The crude was purified by flash column chromatography (Hex / Et_2O = 19:1) to yield **N11** as a clear oil (55% yield).

Methyl -4-isopropyl-1-methylbicyclo[2.2.1]hept-2-ene-2-carboxylate (N11)

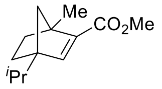

N11

Clear oil, **R$_f$** = 0.33 (Cy / DCM = 1:1). **^{1}H NMR** (400 MHz, $CDCl_3$) δ 6.90 (s, 1H), 3.71 (s, 3H), 1.88 (p, *J* = 6.9 Hz, 1H), 1.75 (ddd, *J* = 11.5, 9.2, 3.5 Hz, 1H), 1.59 (ddd, *J* = 13.0, 9.3, 4.1 Hz, 1H), 1.48 (s, 3H), 1.36 – 1.25 (m, 2H), 1.16 (tdd, *J* = 11.6, 5.3, 3.3 Hz, 1H), 1.05 (dd, *J* = 8.2, 1.1 Hz, 1H), 0.99 (d, *J* = 6.9 Hz, 3H), 0.96 (d, *J* = 6.8 Hz, 3H). **^{13}C NMR** (101 MHz, $CDCl_3$) δ 165.74, 149.39, 141.78, 59.62, 56.07, 51.11, 50.62, 34.37, 31.10, 30.04, 19.96, 19.49, 18.25. IR 2955, 2870, 1716, 1434, 1311, 1283, 1255, 1234, 1192, 1180, 1094, 758. **HRMS** (FI) calculated for $C_{13}H_{20}O_2$ $[M]^+$: 208.1463; found: 208.1463. **IR** (neat): v_{max} (cm^{-1}): 2955, 2870, 1716, 1434, 1311, 1283, 1255, 1234, 1192, 1180, 1094, 758.

4.1.5. Synthesis of Norbornene N13

A procedure described in the literature was adapted for the synthesis of **N13.**[8]

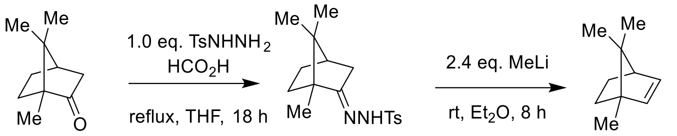

Camphor **N13-1** 72% yield **N13** 27% yield

N13-1: Sulfonyl hydrazine (1.77 g, 9.5 mmol, 1 eq.) was dissolved with a minimum amount of refluxing methanol (3 mL). Then, a few drops of formic acid and camphor (1.52 g, 10 mmol, 1.05 eq.) were added. The reaction was refluxed overnight (18 h). A precipitate was formed, and the suspension was filtered and washed with hexanes to yield **N13-1** as a white solid (72% yield 2.3 g, 7.2 mmol).
N13: To a solution of **N13-1** (2.1 g, 6.5 mmol, 1 eq.) in Et_2O (25 mL, 0.25 M) at 0 °C, a solution of MeLi (9.75 mL, 15.6 mmol, 2.4 eq., 1.6 M in Et_2O) was slowly added under nitrogen atmosphere, the reaction mixture turned yellow and bubbles evolved. The reaction was stirred at room temperature for 8 h. The reaction was slowly poured into an ice cooled sat. NH_4Cl solution and extracted with pentane (× 3). After careful concentration [Note: product is volatile] under reduced pressure, the residue was purified by Kugelrohr distillation (60 mbar, 120-200 °C) to yield **N13** as a clear oil (27% yield, 242 mg, 1.76 mmol).

1,7,7-Trimethylbicyclo[2.2.1]hept-2-ene (N13)

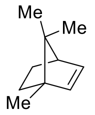

N13

Clear yellow oil. **^{1}H NMR** (400 MHz, $CDCl_3$) δ 5.90 (dd, *J* = 5.8, 3.1 Hz, 1H), 5.65 (d, *J* = 5.8 Hz, 1H), 2.27 (t, *J* = 3.4 Hz, 1H), 1.85 – 1.74 (m, 1H), 1.60 – 1.49 (m, 1H), 1.02 (s, 3H), 1.01 – 0.86 (m, 2H), 0.82 (s, 3H), 0.76 (s, 3H). **^{13}C NMR** (101 MHz, $CDCl_3$) δ 139.49, 134.02, 52.30, 44.81, 31.65, 27.21, 24.66, 19.76, 19.67, 13.38.

4.1.6. Synthesis of Norbornene N14

Procedures described in the literature were adapted for the synthesis of **N14.**[17,18]

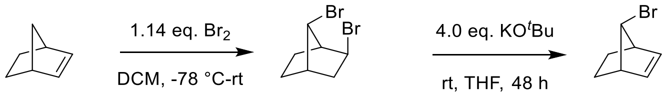

N14-1 17% yield **N14** quantitative

N14-1: To a solution of norbornene (1.02 g, 10.8 mmol, 1 eq.) in DCM (200 mL, 0.05 M) at -78 °C, a solution of bromine (2.0 g, 12.4 mmol, 1.15 eq.) in DCM (4 mL) was slowly added over a period of 30 min. The reaction was quenched with saturated Na_2CO_3, extracted with Et_2O (× 3) and the combined organic layers were dried over anhydrous $MgSO_4$, filtered and concentrated under reduced pressure [Note: a mixture of different dibrominated products was obtained with **N14-1** as the major product). The mixture was purified by distillation at 1×10^{-3} mbar. The oil bath was heated up to 100 °C (vapor temperature up to 55 °C) which removed most of the byproducts. By increasing the temperature, **N14-1** was isolated as a clear oil (17% yield, 460 mg, 1.84 mmol).

2,7-Dibromobicyclo[2.2.1]heptane (N14-1)

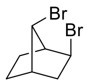

N14-1

Clear oil, **^{1}H NMR** (400 MHz, $CDCl_3$) δ 4.03 – 3.88 (m, 2H), 2.75 – 2.64 (m, 2H), 2.45 (t, *J* = 3.8 Hz, 1H), 2.24 (dd, *J* = 13.9, 8.5 Hz, 1H), 1.68 (tdd, *J* = 18.3, 9.5, 5.4 Hz, 2H), 1.40 – 1.25 (m, 2H). ^{1}H NMR of the isolated material matched with that reported in the literature.[18]
N14. To a solution of **N14-1** (126 mg, 0.5 mmol, 1 eq.) in THF (3.9 mL, 0.13 M), potassium *tert*-butoxide (224 mg, 2 mmol, 4 eq.) was added under nitrogen atmosphere and the reaction was stirred 48h at room temperature. The mixture was diluted with water, extracted with Et_2O (x 3) and the combined organic layers were washed with water (× 2), dried over anhydrous $MgSO_4$, filtered and concentrated under reduced pressure to provide **N14** as a clear oil (quantitative, 85.5 mg, 0.5 mmol), which was used without further purification.

7-Bromobicyclo[2.2.1]hept-2-ene (14)

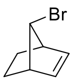

N14

Clear oil, **^{1}H NMR** (400 MHz, $CDCl_3$) ^{1}H NMR (400 MHz, $CDCl_3$) δ 6.03 (s, 2H), 3.88 (s, 1H), 3.04 (s, 2H), 1.77 (d, *J* = 8.9 Hz, 2H), 1.17 – 1.03 (m, 2H). ^{1}H NMR of the isolated material matched with that reported in the literature.[18]

4.2. Determination of Stereochemistry of Norbornane Intermediates

4.2.1. Determination of Stereochemistry of N8-5

N8-5 was isolated as a mixture of diastereomers in a ratio of 1.5:1. The stereochemistry can be unambiguously assigned using ^{1}H NMR spectroscopy.

The major diastereomer (**N8-5-1**) shows a coupling constant of J = 3.6 Hz for H_a and H_d. This coupling constant is consistent with literature data[19,22] for long-range coupling (^{4}J) between *endo*-H_a and H_d and therefore implies the *exo*-isomer. For the minor diastereomer (**N8-5-2**), a vicinal coupling constant (^{3}J) of 4.5 Hz for H_a and H_b was observed, which according to literature data,[19,22] suggests the *exo*-H_a and therefore the *endo* configuration for **N8-5-2** (Figure S1). Additionally, the stereochemical assignments were confirmed by nuclear Overhauser effect spectroscopy (NOE). For the major diastereomer (*exo*-**N8-5-1**) the peaks of H_a, H_b and H_c were selectively excited for each isomer (Figure S2). Exciting H_c clearly elucidates the peak of H_d and the coupling constants can be determined in the NOE experiment (H_d J = 10.7, 3.6, 2.0 Hz), which further confirms the coupling of H_a and H_d (Figure S1). By selectively exciting H_b coupling with $H_{a,c,d,e \text{ and } f}$ is observed in the NOE experiment. If H_a is selectively excited, a peak is observed, which results from the interaction with H_f (Figure S2). From the combined information of the NOE experiments and coupling constants, the major isomer (**N8-5-1**) can be assigned as the *exo*-isomer. NOE experiments were also conducted for the minor diastereomer (**N8-5-2**). Selectively exciting H_a or H_b result both in the same peak, which corresponds with H_c, confirming the *endo* configuration (Figure S3).

Figure S1. Determination of stereochemistry of **N8-5** via constant coupling, chemical shift and NOE experiments (^{3}J = vicinal coupling constant, ^{4}J = long-range coupling constant).

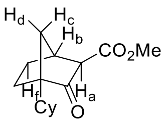

exo-**N8-5-1 major**

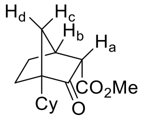

endo-**N8-5-2 minor**

H_a 2.95 ppm J= 3.6 Hz
H_b 2.79 ppm J= 3.8, 1.8 Hz
H_c 2.09 ppm J= 10.8, 2.0 Hz
H_d 1.56 ppm J= 10.7, 3.6, 2.0 Hz

Indicative Coupling:
$^4J_{Ha\text{-}Hd}$ = 3.6 Hz,
$J_{Hc\text{-}Hd}$ = 10.8 Hz.

H_a 3.04 ppm J= 4.5, 1.6 Hz

Indicative Coupling:
$^3J_{Ha\text{-}Hb}$ = 4.5 Hz,

Figure S2. The structure assignment of the diastereomer **N8-5-1** by NOE NMR spectroscopy.

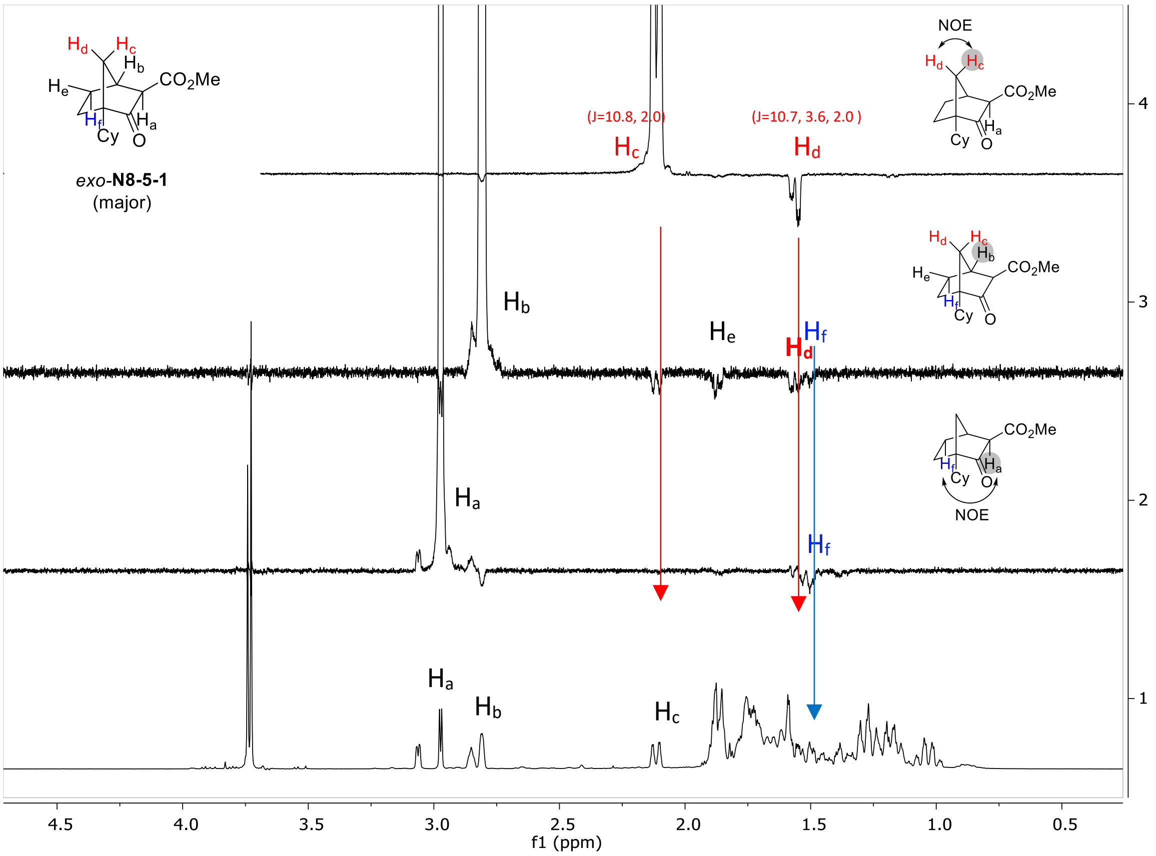

Figure S3. The structure assignment of the diastereomer **N8-5-2** by NOE NMR spectroscopy.

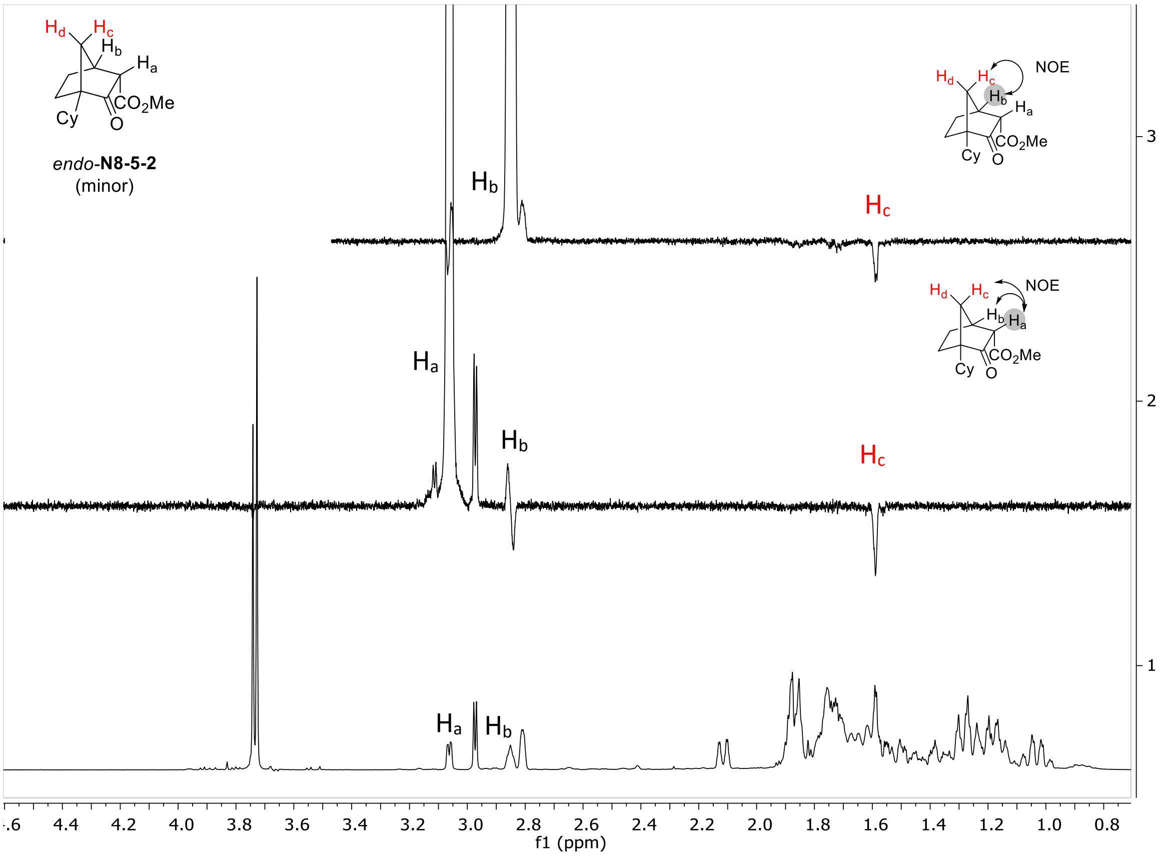

4.2.2. Determination of Stereochemistry of N7-5

The indicative signal of the NMR of **N8-5** can be compared with the ones of **N7-5**, by doing so the stereochemistry of **N7-5** can be assigned (Figure S4).

Figure S4. The structure assignment of the diastereomer **N7-5** by NOE NMR spectroscopy.

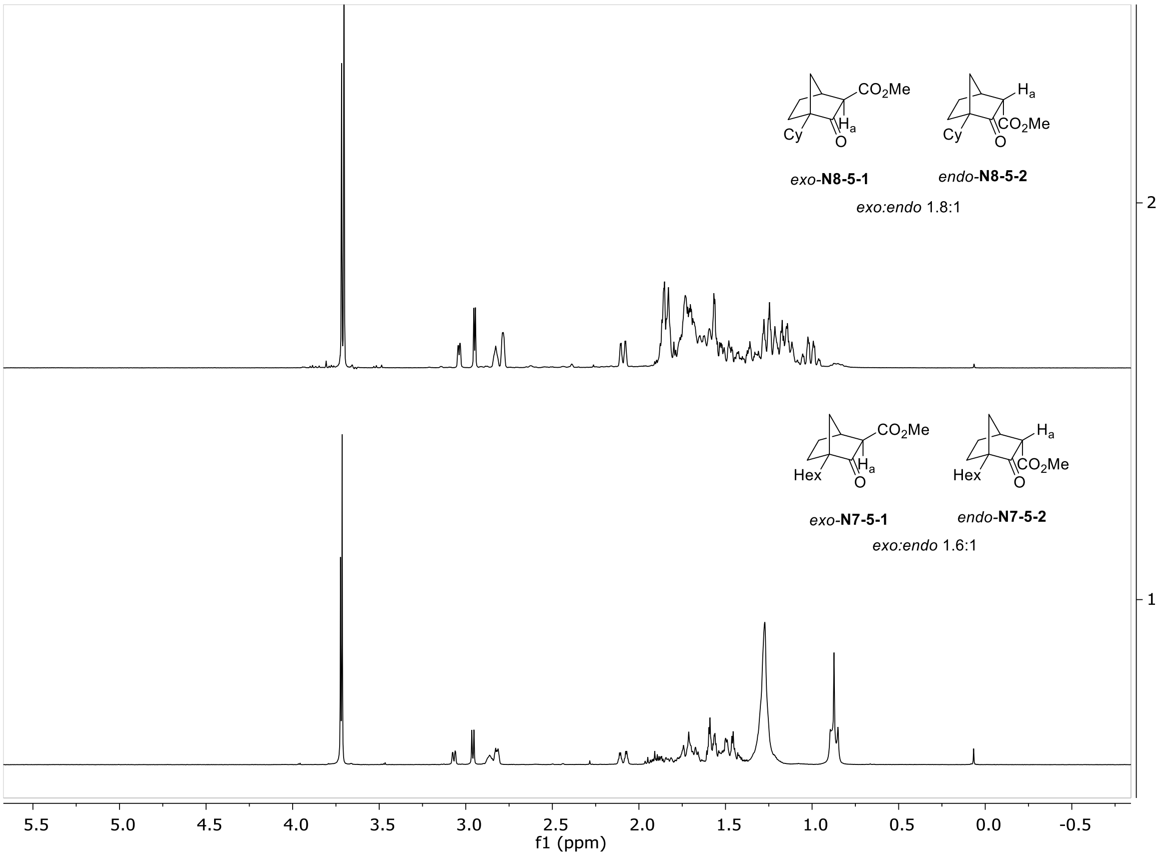

4.2.3. Determination of Stereochemistry of N8-6

According to the literature data[19–23] the *exo-exo* and endo-*endo* vicinal coupling constant of protons H_a and H_b show a coupling constant between $^3J_{Ha\text{-}Hb}$ = 7.5-9.2[19,22] Hz. On the other hand, if protons H_a and H_b are positioned on opposite sides (*exo*-H and *endo*-H) a smaller coupling constants of 2.2 – 5.8 Hz were reported. [19,22] Hence, it can be concluded, that in the case of **N8-6-2** and **N8-6-3** the H_a and H_b are positioned on the same side due to coupling constants of $^3J_{Ha\text{-}Hb}$ = 9.6 Hz and 7.0 Hz, respectively. For **N8-6-1** a smaller coupling constant of $^3J_{Ha\text{-}Hb}$ = 4.0 Hz was observed, which suggests that H_a and H_b are positioned on opposite sides. This is supported by the different coupling constant of each proton H_b (J = 2.8 Hz) and H_a (J = 1.9 Hz) (Figure S5).

The stereochemical assignments were confirmed by nuclear Overhauser effect spectroscopy (NOE). Additionally, *exo*- and *endo*-configuration for each isomer for the hydroxy and ester group could be assigned. The peaks of H_a, H_b and H_c were selectively excited for each diastereomer. For the diastereomer **N8-6-1** strong NOE peaks result from the interaction of *exo*-H_b and H_d as well as H_c and H_d (Figure S6). On the other hand, for H_a this interaction was not observed, but an interaction with H_h suggesting the *endo*-H_a. Therefore, by comparing coupling and intensity of the peaks, the configuration of *exo*-OH and *endo*-CO_2Me can be assigned for **N8-6-1**.

The NOE experiments performed on **N8-6-2**, show interaction of H_a and H_b with H_d suggesting *exo*-H_a and *exo*-H_b (Figure S7). For **N8-6-3** the NOE experiment confirmed *endo*-H_a and *endo*-H_b, because of the interaction of H_a with H_h and H_b with H_f (Figure S8) [Note: The switch on the configuration from mainly *exo*-CO_2Me in **N8-5** to *endo*-CO_2Me in **N8-6** can be attributed to the epimerization of the α-carbon under the basic condition used during the reduction].

Figure S5. Determination of stereochemistry of **N8-6** via constant coupling, chemical shift and NOE experiments, (^{3}J = vicinal coupling constant, ^{4}J = long-range coupling constant).

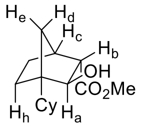

N8-6-1 major
exo-OH, *endo*-CO_2Me
H_a 4.34 ppm 4.1, 1.9 Hz
H_b 2.16 ppm 4.0, 2.8 Hz
H_c 2.38 ppm 4.7 Hz
Indicative Coupling:
$^3J_{Ha\text{-}Hb}$ = 4.0 Hz,
$^4J_{Ha\text{-}He}$ = 1.9 Hz.

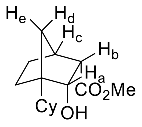

N8-6-2 major
endo-OH, *endo*-CO_2Me
H_a 4.18 ppm, 9.6 Hz
H_b 2.83 ppm, 9.7, 4.8 Hz
H_c 2.42 ppm, 4.6, 2.6 Hz
Indicative Coupling:
$^3J_{Ha\text{-}Hb}$ = 9.6 Hz,
$^3J_{Hb\text{-}Hc}$ = 4.7 Hz

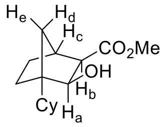

N8-6-3 minor
exo-OH, *exo*-CO_2Me
H_a 3.97 ppm 6.9, 1.6 Hz
H_b 2.71 ppm 7.2, 1.7 Hz
H_c 2.43 ppm 2.4 Hz
Indicative Coupling:
$^3J_{Ha\text{-}Hb}$ = 7.0 Hz,
$^4J_{Ha\text{-}He}$ = 1.6 Hz,
$^4J_{Hb\text{-}He}$ = 1.7 Hz,

Figure S6. The structure assignment of the diastereomer **N8-6-1** by NOE NMR spectroscopy.

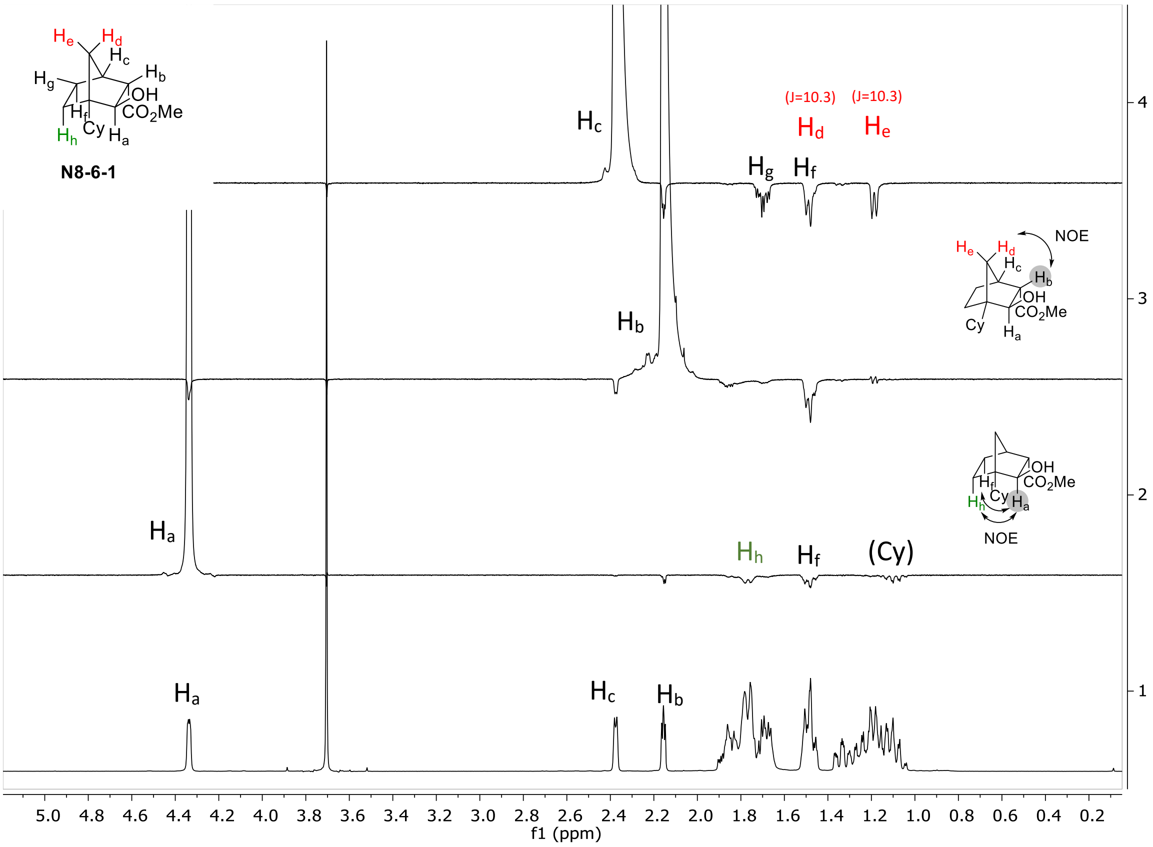

Figure S7. The structure assignment of the diastereomer **N8-6-1** by NOE NMR spectroscopy.

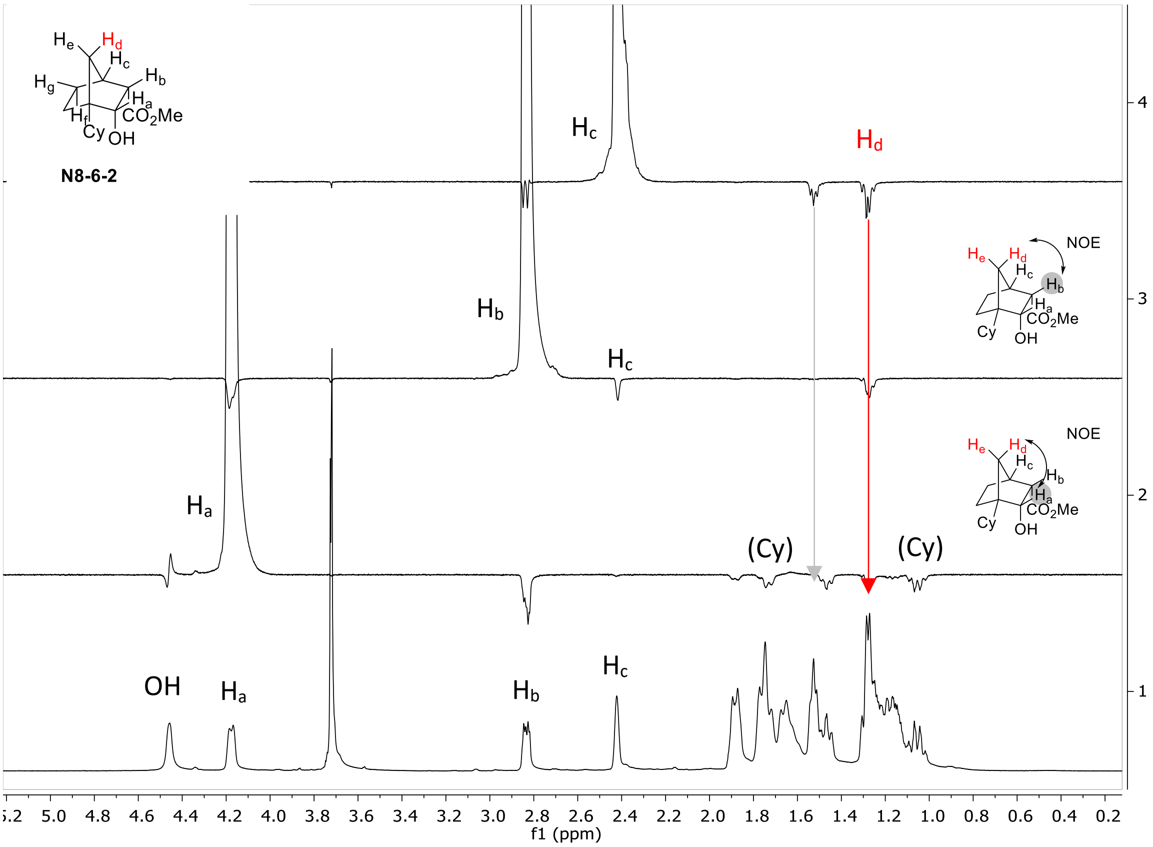

Figure S8. The structure assignment of the diastereomer **N8-6-3** by NOE NMR spectroscopy.

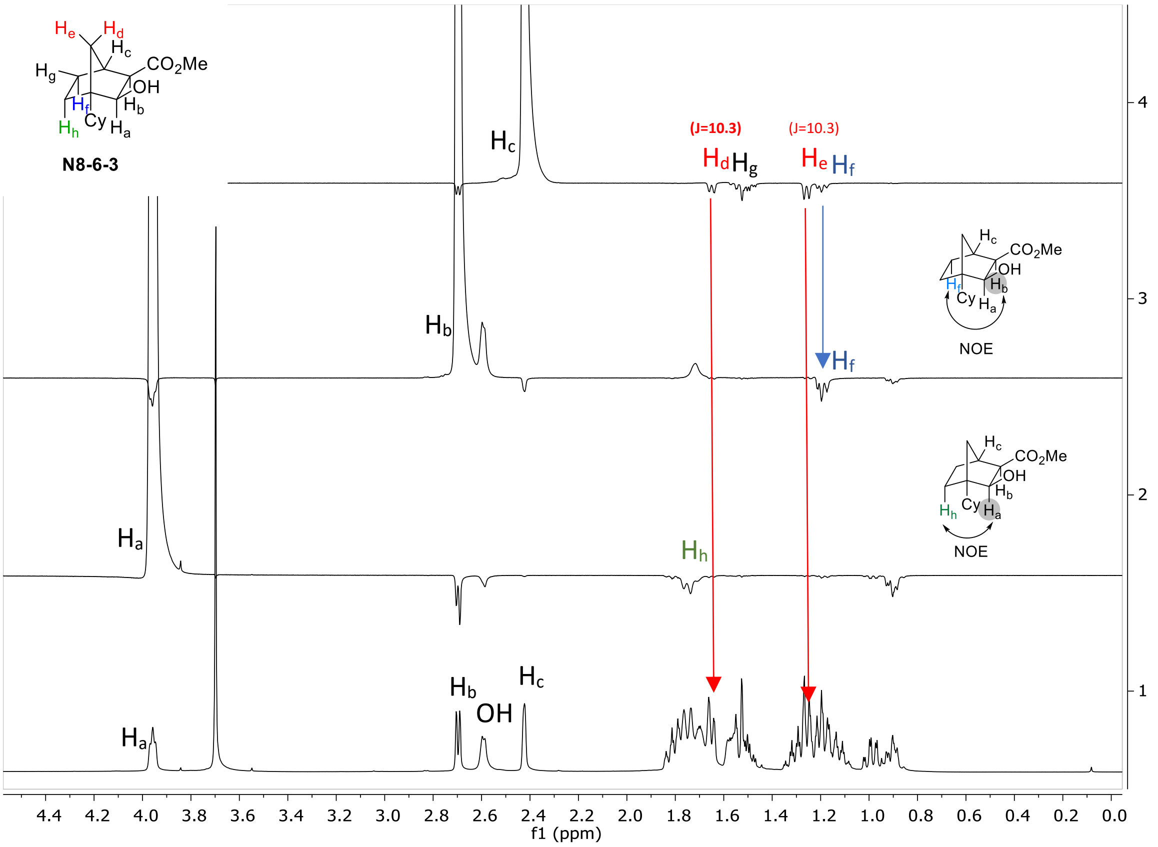

4.2.4. Determination of Stereochemistry of N11-3

The stereochemistry can be unambiguously assigned using ^{1}H NMR spectroscopy.

The minor diastereomer (**N11-3-1**) shows a long-range coupling constant of $^{3}J_{Ha\text{-}Hb}$ = 3.3 Hz. According to the literature data[19–23] this coupling constant is consistent with *endo*-H$_a$. For the major diastereomer (**N11-3-2**) the coupling constant of H$_a$ is significantly smaller (J = 1.8 Hz), which suggests the *endo* configuration (*exo*-H$_a$) (Figure S9). [19–23] Additionally, the stereochemical assignments were confirmed by nuclear Overhauser effect spectroscopy (NOE). The peak of H$_a$ was selected to be excited for each isomer. For the minor diastereomer (**N11-3-1**) the observed NOE peak resulted from the interaction of *endo*-H$_a$ and H$_d$ (Figure S10). On the other hand, for the major diastereomer (**N11-3-2**) an interaction between *exo*-H$_a$ and H$_b$ was observed (Figure S11).

Figure S9. Determination of stereochemistry of **N11-3** via constant coupling, chemical shift and NOE experiments, (^{4}J = long-range coupling constant).

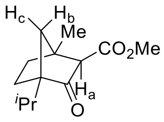

exo-**N11-3-1 (minor)**

H$_a$ 2.94 ppm J= 3.3 Hz
H$_b$ 2.25 ppm J= 10.5, 2.4 Hz
H$_c$ 1.43 ppm J= 10.5, 3.3 Hz

Indicative Coupling:
$^{4}J_{Ha\text{-}Hc}$ = 3.3 Hz,
$J_{Hb\text{-}Hc}$ = 10.5 Hz.

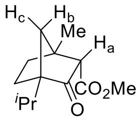

endo-**N11-3-2 (major)**

H$_a$ 2.86 ppm J= 1.8 Hz
H$_b$ 1.43 ppm J= 10.3, 2.2 Hz
H$_c$ 1.54 ppm J= 10.3 Hz

Indicative Coupling:
$J_{Hb\text{-}Hc}$ = 10.3 Hz,

Figure S10. The structure assignment of the minor diastereomer **N11-3-1** by NOE NMR spectroscopy.

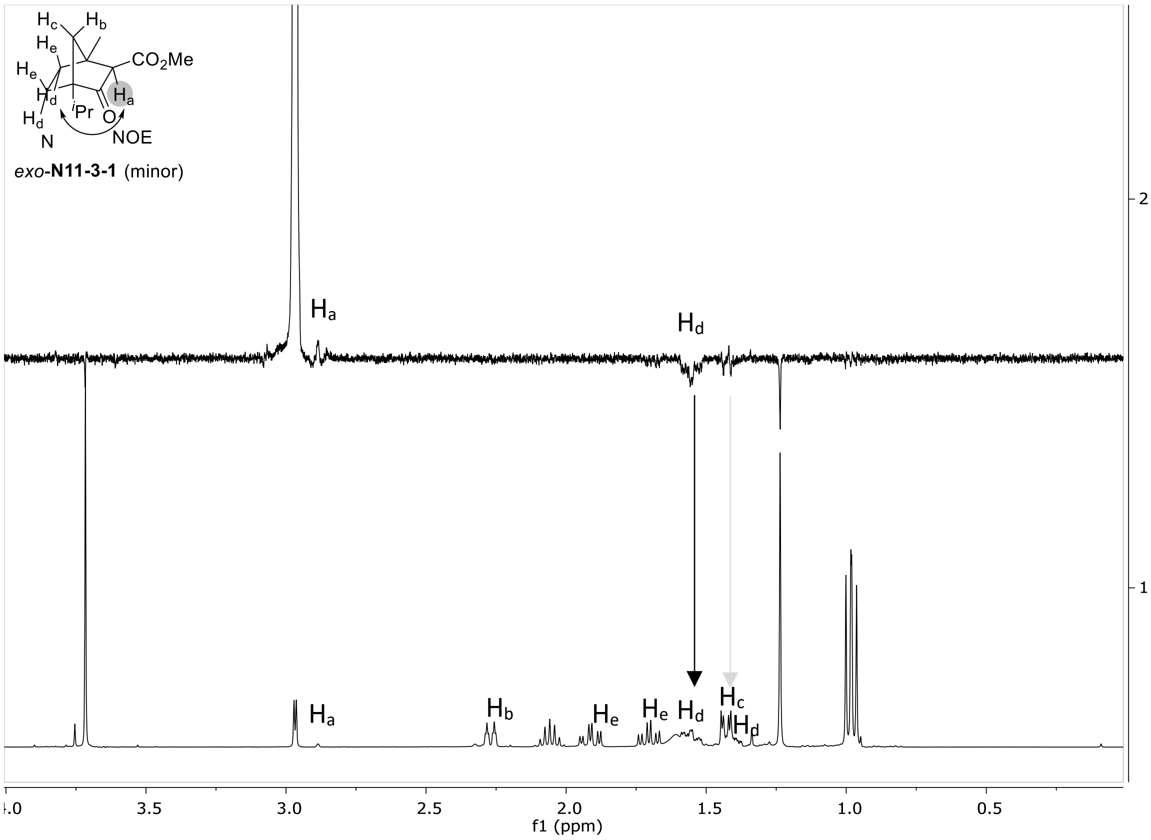

Figure S11. The structure assignment of the major diastereomer **N11-3-2** by NOE NMR spectroscopy.

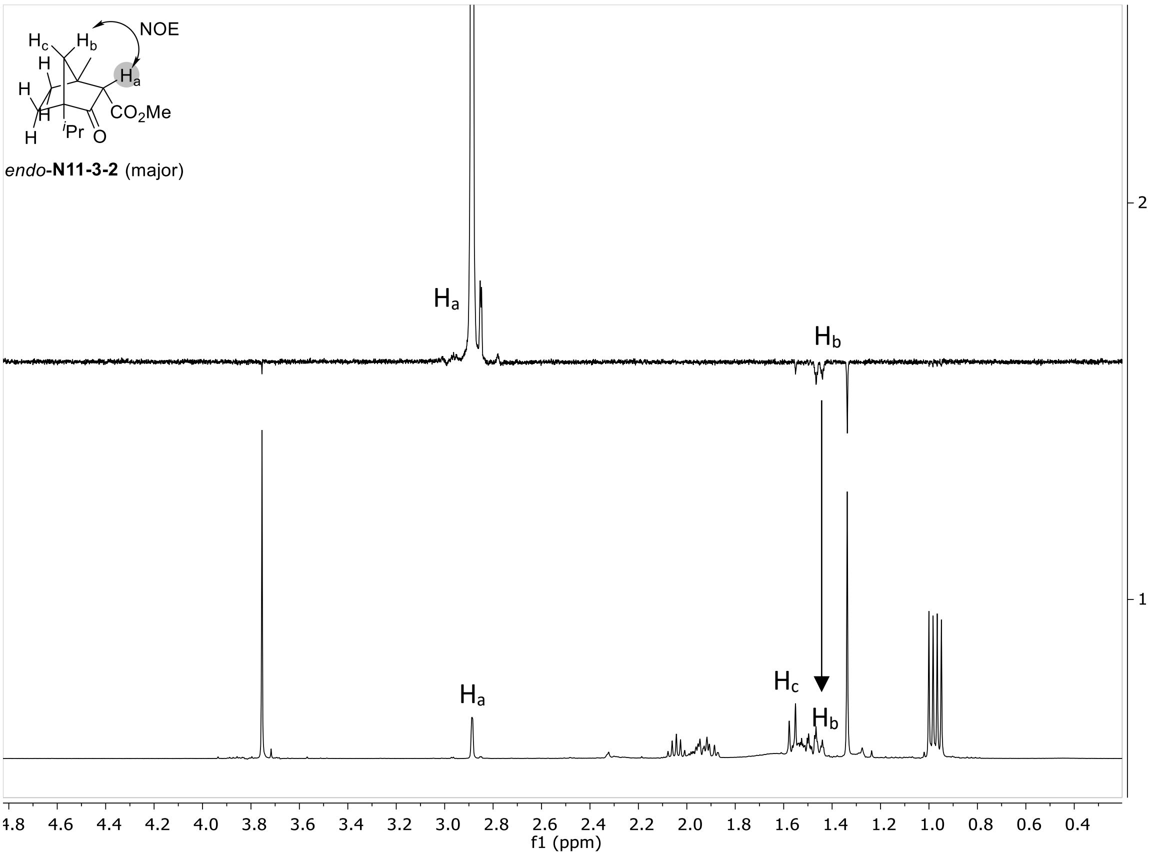

5. Optimization of the *meta-C–H* Arylation of Aryl Ethers

5.1. Optimization of the *meta*-C–H Arylation of Anisole

In a pressure tube containing a suitable stirring bar was added the corresponding amount of NBE, $Pd(OAc)_2$, S,O-ligand, methyl 4-iodobenzoate, AgOAc, anisole (0.1 mmol, 1.0 eq.) and solvent. The tube was put into a pre-heated oil bath at the appropriate temperature and was stirred overnight (~18 h). After cooling to room temperature, the reaction was filtered through Celite®, rinsed with EtOAc and concentrated under reduced pressure. To the crude mixture, CH_2Br_2 (7.08 µL, 0.1 mmol) was added as internal standard, then the mixture was dissolved in $CDCl_3$ and ^{1}H NMR was measured.

5.1.1. Initial Norbornene and Solvent Screening

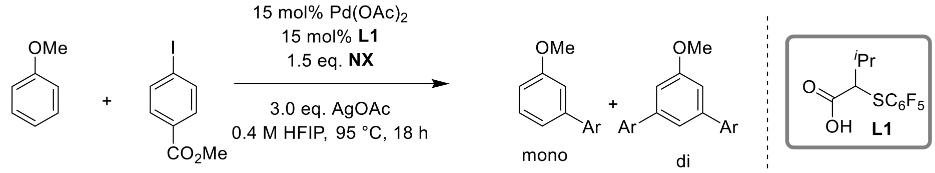

1a 2 2 eq. 3a

Table S3. Initial screening of solvent and NBEs

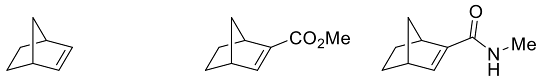

N1 20% yield only mono | **N2** 39% yield mono:di 1.5:1 | **N3** 36% yield mono:di 1.6:1

#	Solvent (Screening with **N2**)	^{1}H-NMR yield [%] **3a**	ratio mono : di
1	HFIP	39	1.5 : 1
2	AcOH	24	1.5 : 1
3	^{t}AmOH	4	3 : 1
4	DCE	25	1.5 : 1
5	DCM	21	2.5 : 1
6	DMF	-	- : -
7	Toluene	11	2.5 : 1
8	Dioxane	traces	- : -

5.1.2. Screening of Ligands

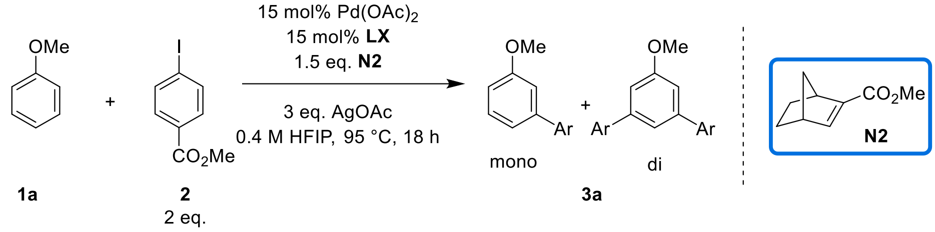

Table S4. Ligand screening (^{1}H-NMR yield).

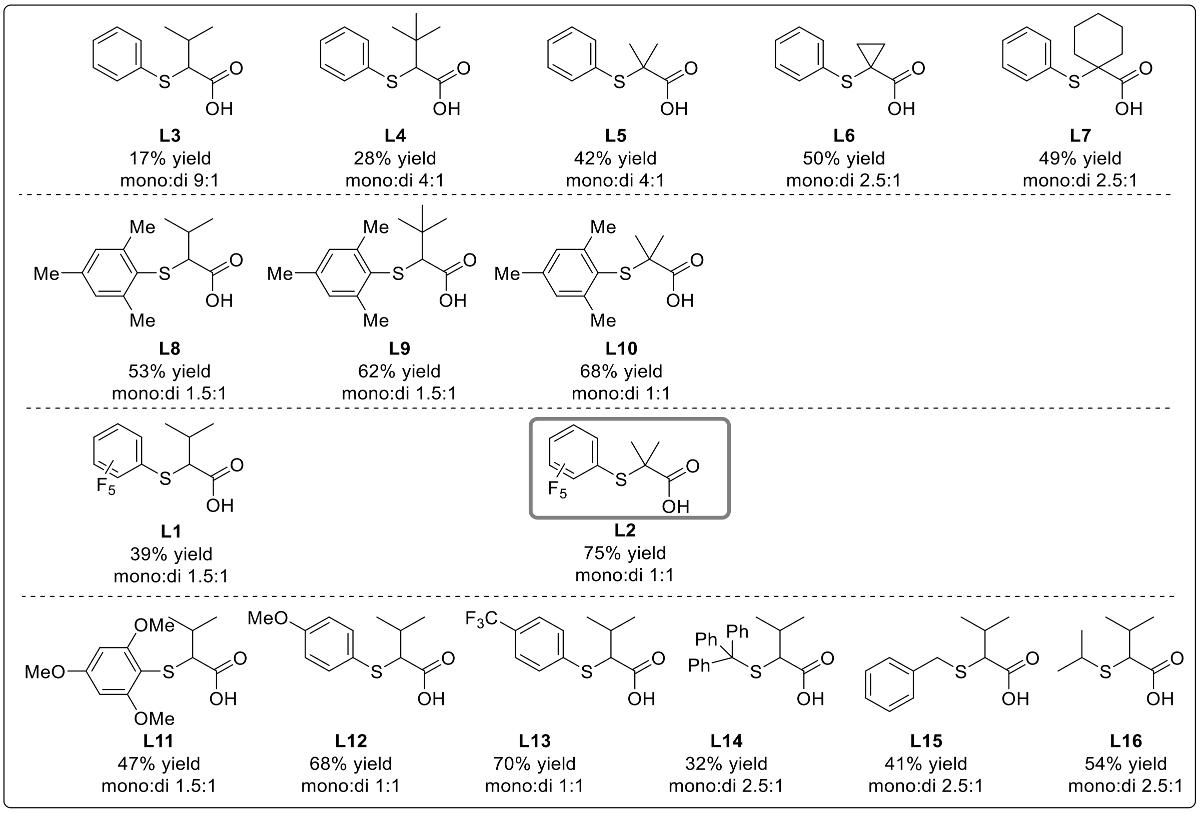

5.2 Optimization of the *meta*-Arylation of 3-Methylanisole (*meta*-substituted substrates)

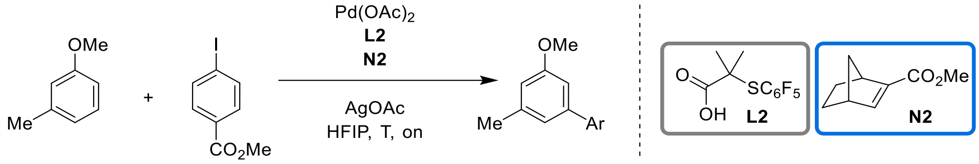

Table S5. Optimization of *meta* arylation with 3-methylanisole **1b**.

#	Pd/**L2** [mol%]	**N2** [eq.]	**2** [eq.]	AgOAc [eq.]	Temp. [°C]	Solvent	^{1}H-NMR yield [%] **3b**
1	15	1.5	2	3	95	0.4 M HFIP	100
2	15	1.5	2	3	95	**0.4 M DCE**	60
3	15	1.5	2	**1.5**	95	0.4 M HFIP	100
4	**10**	1.5	2	1.5	95	0.4 M HFIP	95
5	**5**	1.5	2	1.5	95	0.4 M HFIP	95
6	5	1.5	2	1.5	95	**0.66 M HFIP**	92
7	5	1.5	2	1.5	95	**1.0 M HFIP**	95
8	5	1.5	2	**1.0**	95	0.4 M HFIP	65
9	5	1.5	2	1.5	**90**	0.4 M HFIP	100
10	5	1.5	2	1.5	**80**	0.4 M HFIP	30
11	5	1.5	1.5	1.5	90	0.4 M HFIP	100
12	5	0.75	1.5	1.5	90	0.4 M HFIP	100
13	5	0.5	1.5	1.5	90	0.4 M HFIP	100
14	5	0.2	1.5	1.5	90	0.4 M HFIP	85
Control Experiments							
15	**w/o L2**	0.5	1.5	1.5	90	0.4 M HFIP	10
16	5	**w/o N2**	1.5	1.5	90	0.4 M HFIP	nP[a]
17	5	0.5	1.5	**w/o Ag**	90	0.4 M HFIP	nP
18	5	0.5	1.5	**CsOAc** instead of AgOAc	90	0.4 M HFIP	nP

[a] The reaction without **N2** provided only trace amounts of arylated anisole. Mainly the homocoupling and acetylation of **2** were observed.

5.3 Evaluation of Other Reported Ligands Under the Optimized Reaction Conditions

To compare the performance of our S,O-ligand with other successfully reported ligands in C–H functionalization reactions, we have performed the reaction of 3-methylanisole (**1b**) and methyl 3-methoxybenzoate (**1j**) under the optimized reaction conditions in the presence of different ligands. The ratio Pd/ligand was adjusted to the ratio reported for each ligand. The following ligands have been tested: $AsPh_3$ (**L17**),[24] quinoxaline (**L18**),[25] **L19**,[26] **L20**,[27] methyl phenyl thioether (**L21**), pivalic acid (**L22**) and a combination of methyl phenyl thioether (**L21)** and pivalic acid (**L22**). The results are presented in Table S6 and S7. From all the ligands tested, the S,O-ligand **L2** significantly outperformed the others.

Table S6. Screening of ligands reported in literature with substrate **1b**.

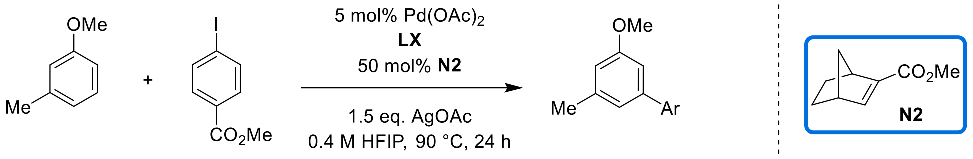

1b **2** 1.5 eq. **3b**

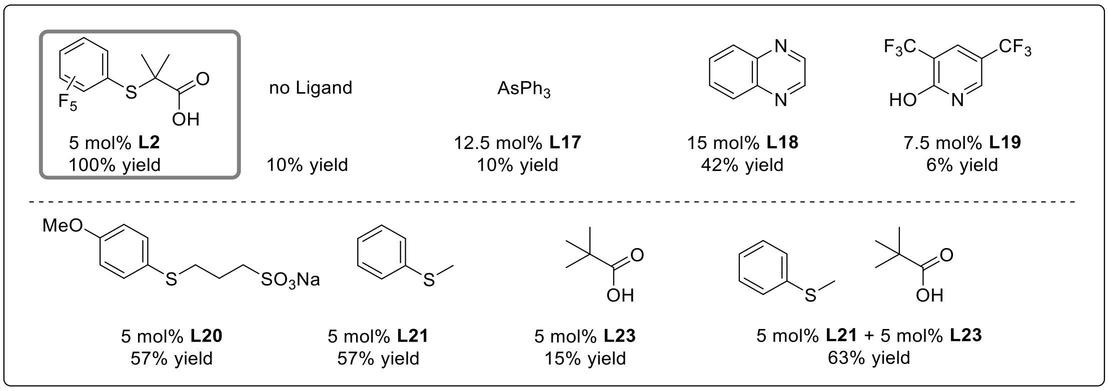

Table S7. Screening of ligands reported in literature with substrate **1j**.

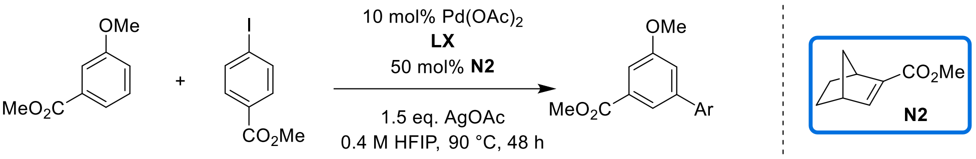

1j 2.0 eq. **2** 1.0 eq. **3j**

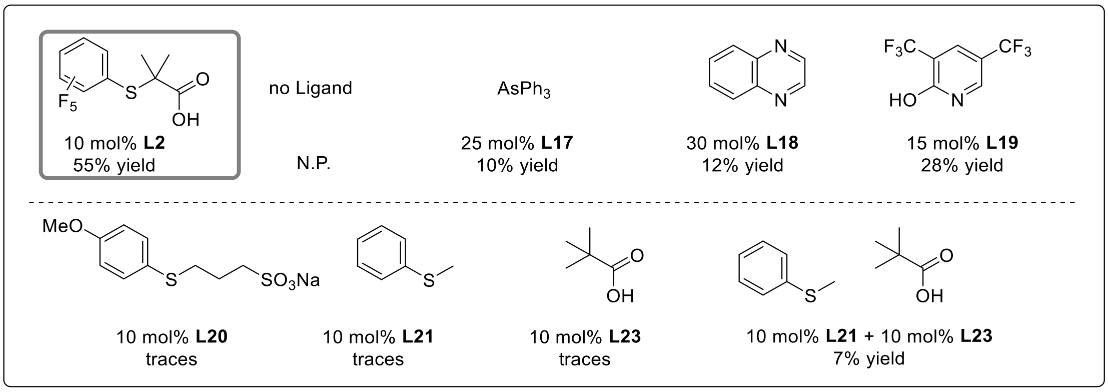

6. General Procedure A for the *meta*-C–H Arylation of *meta*-Substituted Anisoles

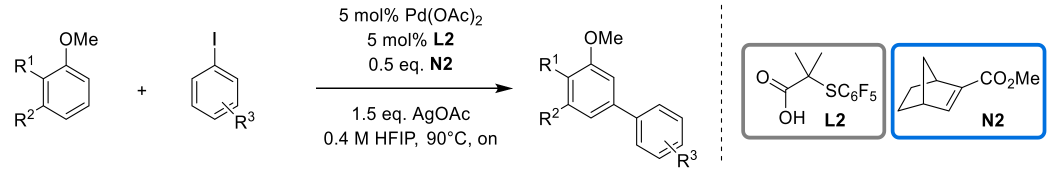

1 a-o 1.0 eq. **2a-f** 1.5 eq. **3a-o and 4a-f**

In a pressure tube containing a suitable stirring bar, **N2** (19.0 mg, 0.125 mmol, 0.5 eq.), Pd(OAc)$_2$ (2.8 mg, 0.0125 mmol, 5 mol%), silver acetate (62.6 mg, 0.375 mmol, 1.5 eq.), aryl halide **2** (0.375 mmol, 1.5 eq.), anisole derivative (0.25 mmol, 1.0 eq.), a stock solution of S,O-ligand **L2** in HFIP (125 µL, 0.1 M, 0.0125 mmol, 5 mol%),HFIP (0.5 mL, 0.4 M) were added. The tube was put into a pre-heated oil bath at 90 °C and was stirred for 24 h. After cooling to room temperature, the reaction was filtered through Celite® and rinsed with EtOAc. The solvent was evaporated under reduced pressure. To the crude mixture CH_2Br_2 (17.7 µL, 0.25 mmol) was added as internal standard, the mixture was dissolved in $CDCl_3$ and ^{1}H NMR was measured. Subsequentially, the product was purified by flash column chromatography.

Table S9. Scope of *meta* substituted aryl ethers

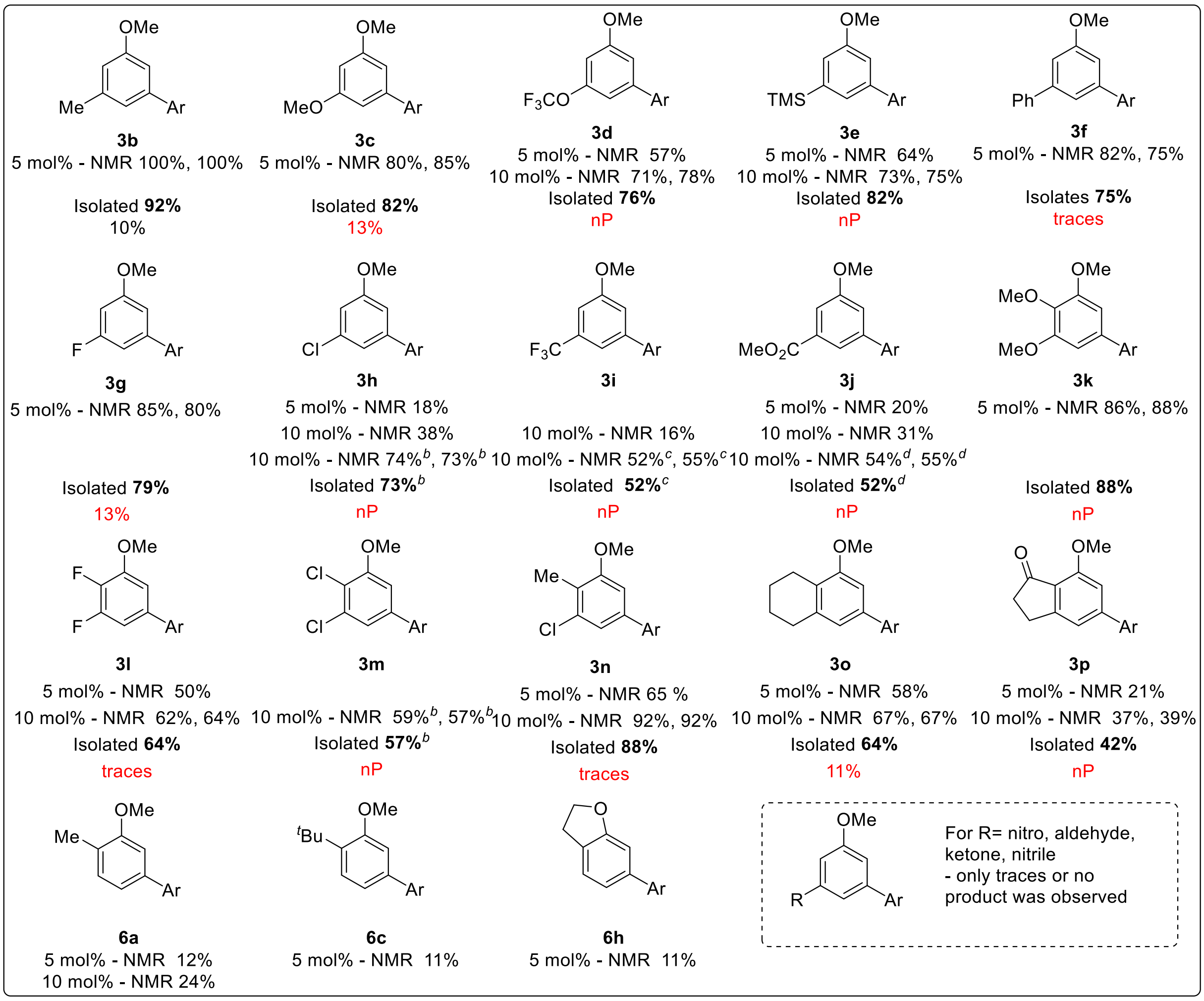

All reaction were first performed on a 0.1 mmol scale. With the optimal conditions, the isolated yield was obtained from the reaction on 0.25 mmol scale. Yields in red corresponds to the reaction without ligand. [b] 30 mol% NBE **N3** instead of NBE **N2** was used. [c]3.6 eq. anisole **1i**, 1.0 eq. aryl iodide **2,** 20 mol%. **N2**, 2 eq. AgOAc, 0.2M HFIP, 70 °C, 48 h. [d] 2.0 eq. anisole **1j**, 1.0 eq. aryl iodide **2**, 48 h.

Methyl 3'-methoxy-5'-methyl-[1,1'-biphenyl]-4-carboxylate (3b)

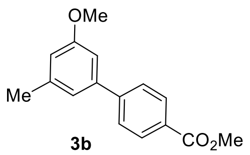

3b

Genereal procedure **A** was followed using 3-methylanisole (31.8 µL, 0.25 mmol, 1.0 eq.) as substrate and methyl 4-iodobenzoate (100.3 mg, 0.375 mmol, 1.5 eq.), providing the arylated product in 100% ^{1}H NMR yield. Purification by column chromatography on silica gel using Cy / DCM (9:1-7:3 v/v) as an eluent provided the title compound as a white solid (58.7 mg, 92% yield). **R$_f$** = 0.34 (Cy / DCM = 1:1). **^{1}H NMR** (400 MHz, $CDCl_3$) δ 8.09 (d, J = 8.2 Hz, 2H), 7.64 (d, J = 8.2 Hz, 2H), 7.03 (s, 1H), 6.96 (s, 1H), 6.77 (s, 1H), 3.94 (s, 3H), 3.86 (s, 3H), 2.41 (s, 3H). **^{13}C NMR** (101 MHz, CDCl3) δ 167.08, 160.16, 145.78, 141.39, 139.83, 130.11, 129.03, 127.18, 120.77, 114.48, 110.20, 55.41, 52.20, 21.76. **HRMS** (EI): *m/z* calculated for $C_{16}H_{16}O_3$ $[M]^+$= 256.1099; found= 256.1070. **IR** (neat): v_{max} (cm^{-1}): 2997, 2950, 2076, 1928, 1716, 1606, 1592, 1510, 1335, 1316, 1272, 1216, 1182, 1167, 1084, 1061, 1048, 1017, 995, 966, 932, 837, 771, 706, 592, 553, 535, 490. ^{1}H NMR of the isolated material matched with that reported in the literature.[25]

A parallel reaction **without ligand** was also performed providing 10% of the title compound.

Methyl 3',5'-dimethoxy-[1,1'-biphenyl]-4-carboxylate (3c)

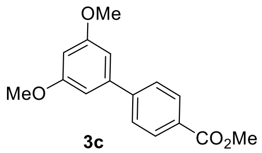

3c

General procedure **A** was followed using 1,3-dimethoxybenzene (32.7 µL, 0.25 mmol, 1.0 eq.) as substrate and methyl 4-iodobenzoate (100.3 mg, 0.375 mmol, 1.5 eq.), providing the arylated product in 85% ^{1}H NMR yield. Purification by column chromatography on silica gel using Cy / DCM (9:1-3:2 v/v) as an eluent provided the title compounds as beige crystals (55.6 mg, 82% yield). **R$_f$** = 0.16 (Cy / DCM = 1:1). **^{1}H NMR** (400 MHz, $CDCl_3$) δ 8.09 (d, *J* = 8.3 Hz, 2H), 7.63 (d, *J* = 8.3 Hz, 2H), 6.75 (d, *J* = 2.2 Hz, 2H), 6.50 (t, *J* = 2.2 Hz, 1H), 3.94 (s, 3H), 3.85 (s, 6H). **^{13}C NMR** (101 MHz, $CDCl_3$) δ 167.02, 161.26, 145.68, 142.27, 130.12, 129.24, 127.20, 105.67, 100.10, 55.55, 52.22. **HRMS** (EI): *m/z* calculated for $C_{16}H_{16}O_4$ $[M]^+$= 272.1049; found= 272.1035. **IR** (neat): v_{max} (cm^{-1}): 2999, 2950, 2838, 1928, 1715, 1590, 1567, 1513, 1398, 1313, 1272, 1218, 1203, 1152, 1102, 1080, 1063, 1033, 1016, 993, 966, 831, 817, 770, 640, 608, 574, 542, 492, 413. **M.P.** = 80−82 °C

A parallel reaction **without ligand** was also performed providing 13% of the title compound.

Methyl 3'-methoxy-5'-(trifluoromethoxy)-[1,1'-biphenyl]-4-carboxylate (3d)

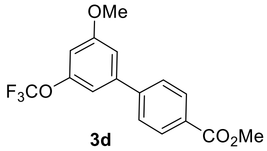

3d

General procedure **A** was followed using 3-trifluoromethoxyanisole (50.6 mg, 0.25 mmol, 1.0 eq.) as substrate and methyl 4-iodobenzoate (100.3 mg, 0.375 mmol, 1.5 eq.) using 10 mol% of $Pd(OAc)_2$ (5.6 mg, 0.025 mmol, 10 mol%) and **L2** (250 µL, 0.1 M, 0.025 mmol, 10 mol%), providing the arylated product in 78% ^{1}H NMR yield. Purification by column chromatography on silica gel using Cy / EtOAc (95:5 v/v) as an eluent provided the title compound as a white solid (62.1 mg, 76% yield). **R$_f$** = 0.36 (Cy / EtOAc = 9:1). **^{1}H NMR** (400 MHz, $CDCl_3$) δ 8.11 (d, *J* = 8.5 Hz, 2H), 7.61 (d, *J* = 8.5 Hz, 2H), 7.06 (dq, *J* = 2.2, 1.3 Hz, 2H), 6.79 (td, *J* = 2.2, 1.1 Hz, 1H), 3.94 (s, 3H), 3.87 (s, 3H). **^{13}C NMR** (101 MHz, $CDCl_3$) δ 166.86, 161.07, 150.61, 144.21, 142.80, 130.32, 129.84, 127.22, 120.61 (q, $J_{C\text{-}F}$ = 257.6 Hz), 112.17, 111.72, 106.53, 55.83, 52.32. **^{19}F NMR** (376 MHz, $CDCl_3$) δ -57.66. **HRMS** (FI): *m/z* calculated for $C_{16}H_{13}F_3O_4$ $[M]^+$= 326.0766; found= 326.0700. **IR** (neat): v_{max} (cm^{-1}): 3079, 3015, 2946, 2841, 1714, 1602, 1567, 1462, 1435, 1398, 1340, 1243, 1207, 1106, 1080, 1053, 1016, 989, 977, 873, 841, 819, 770, 730, 690, 523, 502, 477. **M.P.** = 60−62 °C.

A parallel reaction **without ligand** was also performed providing no title compound.

Methyl 3'-methoxy-5'-(trimethylsilyl)-[1,1'-biphenyl]-4-carboxylate (3e)

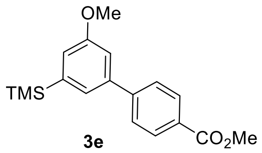

3e

General procedure **A** was followed using 3-trimethylsilylanisole (45.0 mg, 0.25 mmol, 1.0 eq.) as substrate and methyl 4-iodobenzoate (100.3 mg, 0.375 mmol, 1.5 eq.), using 10 mol% of $Pd(OAc)_2$ (5.6 mg, 0.025 mmol, 10 mol%) and **L2** (250 µL, 0.1 M, 0.025mmol, 10 mol%), providing the arylated product in 75% ^{1}H NMR yield. Purification by column chromatography on silica gel using Cy / DCM (9:1-7:3 v/v) as an eluent provided the title compounds as pale orange crystals (52.8 mg, 82% yield). **R$_f$** = 0.34 (Cy / DCM = 1:1). **^{1}H NMR** (400 MHz, $CDCl_3$) δ 8.12 (d, *J* = 8.5 Hz, 2H), 7.67 (d, *J* = 8.5 Hz, 2H), 7.34 (dd, *J* = 1.7, 0.9 Hz, 1H), 7.15 – 7.08 (m, 2H), 3.95 (s, 3H), 3.90 (s, 3H), 0.33 (s, 9H). **^{13}C NMR** (101 MHz, $CDCl_3$) δ 167.09, 159.58, 146.01, 143.14, 141.18, 130.16, 129.07, 127.36, 124.69, 118.78, 113.19, 55.40, 52.23, -0.99. ^{1}H NMR of the isolated material matched with that reported in the literature.[25]

A parallel reaction **without ligand** was also performed providing traces of the title compound.

Methyl 5'-methoxy-[1,1':3',1''-terphenyl]-4-carboxylate (3f)

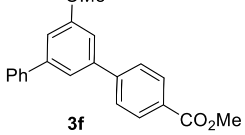

3f

General procedure **A** was followed using 3-methoxybiphenyl (43.9 µL, 0.25 mmol, 1.0 eq.) as substrate and methyl 4-iodobenzoate (100.3 mg, 0.375 mmol, 1.5 eq.), providing the arylated product in 82% ^{1}H NMR yield. Purification by column chromatography on silica gel using Cy / DCM (9:1-3:2 v/v) as an eluent provided the title compound as a white solid (59.2 mg, 75% yield). **R$_f$** = 0.75 (Cy / DCM = 1:1). **^{1}H NMR** (400 MHz, $CDCl_3$) δ 8.13 (d, *J* = 8.4 Hz, 2H), 7.71 (d, *J* = 8.4 Hz, 2H), 7.65 (d, *J* = 7.2 Hz, 2H), 7.47 (t, *J* = 7.5 Hz, 2H), 7.43 (t, *J* = 1.5 Hz, 1H), 7.39 (t, *J* = 7.3 Hz, 1H), 7.15 (dt, *J* = 9.6, 2.3 Hz, 2H), 3.96 (s, 3H), 3.94 (s, 3H). **^{13}C NMR** (101 MHz, $CDCl_3$) δ 167.06, 160.52, 145.63, 143.53, 142.01, 140.99, 130.22, 129.28, 128.95, 127.82, 127.40, 127.32, 119.08, 112.67, 111.98, 55.63, 52.27. ^{1}H NMR of the isolated material matched with that reported in the literature.[25]

A parallel reaction **without ligand** was also performed providing traces of the title compound.

Methyl 3'-fluoro-5'-methoxy-[1,1'-biphenyl]-4-carboxylate (3g)

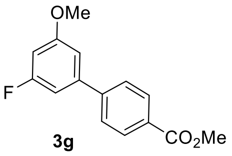

3g

General procedure **A** was followed using 3-fluoroanisole (28.9 µL, 0.25 mmol, 1.0 eq.) as substrate and methyl 4-iodobenzoate (100.3 mg, 0.375 mmol, 1.5 eq.), providing the arylated product in 85% ^{1}H NMR yield. Purification by column chromatography on silica gel using Cy / DCM (9:1-4:1 v/v) as an eluent provided the title compound as a white solid (51.2 mg, 79% yield). **R$_f$** = 0.24 (Cy / DCM = 1:1). ^{1}H NMR (400 MHz, $CDCl_3$) δ 8.09 (d, *J* = 8.2 Hz, 2H), 7.61 (d, *J* = 8.2 Hz, 2H), 6.91 (m, 2H), 6.64 (d, *J* = 10.5 Hz, 1H), 3.94 (s, 3H), 3.85 (s, 3H). ^{13}C NMR (101 MHz, $CDCl_3$) δ 166.90, 164.02 (d, J_{C-F} = 245.2 Hz), 161.36 (d, J_{C-F} = 11.5 Hz), 144.46, 142.73 (d, J_{C-F} = 9.8 Hz), 130.25, 129.67, 127.14, 109.20 (d, J_{C-F} = 2.6 Hz), 106.69 (d, J_{C-F} = 22.7 Hz), 101.16 (d, J_{C-F} = 25.2 Hz), 55.78, 52.29. **^{19}F NMR** (376 MHz, $CDCl_3$) δ -111.13 (t, *J* = 9.9 Hz). **HRMS** (EI): *m/z* calculated for $C_{15}H_{13}FO_3$ $[M]^+$= 260.0849; found= 260.0828. **IR** (neat): v_{max} (cm^{-1}): 3408, 3083, 2999, 2953, 2097, 1938, 1713, 1611, 1592, 1568, 1514, 1400, 1279, 1216, 1185, 1142, 1050, 1017, 998, 835, 819, 768, 637, 595, 537, 493. ^{1}H NMR of the isolated material matched with that reported in the literature.[28]

A parallel reaction **without ligand** was also performed providing 13% of the title compound.

Methyl 3'-chloro-5'-methoxy-[1,1'-biphenyl]-4-carboxylate (3h)

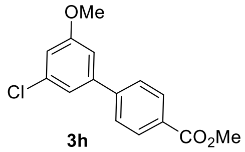

3h

General procedure **A** was followed using 3-chloroanisole (30.8 µL, 0.25 mmol, 1.0 eq.) as substrate and methyl 4-iodobenzoate (100.3 mg, 0.375 mmol, 1.5 eq.), using 10 mol% of $Pd(OAc)_2$ (5.6 mg, 0.025 mmol, 10 mol%) and **L2** (250 µL, 0.1 M, 0.025mmol, 10 mol%) and **N3** (11.3 mg, 0.075 mmol, 0.3 eq.), providing the arylated product in 74% ^{1}H NMR yield. Purification by column chromatography on silica gel using Cy / DCM (9:1-3:2 v/v) as an eluent provided the title compound as a white solid (50.6 mg, 73% yield). **R$_f$** = 0.18 (Cy / EtOAc = 30:1). **^{1}H NMR** (400 MHz, $CDCl_3$) δ 8.09 (d, *J* = 8.5 Hz, 2H), 7.60 (d, *J* = 8.5 Hz, 2H), 7.18 (t, *J* = 1.7 Hz, 1H), 7.01 (dd, *J* = 2.4, 1.5 Hz, 1H), 6.91 (t, *J* = 2.1 Hz, 1H), 3.94 (s, 3H), 3.85 (s, 3H). **^{13}C NMR** (101 MHz, $CDCl_3$) δ 166.88, 160.74, 144.25, 142.67, 135.52, 130.26, 129.68, 127.16, 119.98, 113.72, 111.94, 55.75, 52.31. **HRMS** (EI): *m/z* calculated for $C_{15}H_{13}ClO_3$ $[M]^+$= 276.0553; found= 272.0506. **IR** (neat): v_{max} (cm^{-1}): 3079, 3001, 2950, 2838, 1718, 1595, 1580, 1563, 1513, 1454, 1431, 1396, 1314, 1273, 1212, 1183, 1104, 1075, 1039, 1017, 993, 971, 890, 875, 841, 802, 770, 705, 686. **M.P.** = 100−102 °C

A parallel reaction **without ligand** was also performed providing traces of the title compound

Methyl 3'-methoxy-5'-(trifluoromethyl)-[1,1'-biphenyl]-4-carboxylate (3i)

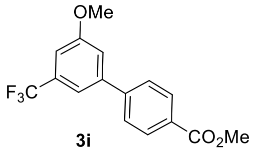

3i

General procedure **A** was followed using 3-trifluoromethylanisole (133.2 µL, 0.9 mmol, 3.6 eq.) as substrate and methyl 4-iodobenzoate (65.5 mg, 0.25 mmol, 1.0 eq.) at 70 °C, using 10 mol% of $Pd(OAc)_2$ (5.6 mg, 0.025 mmol, 10 mol%) and **L2** (250 µL, 0.1 M, 0.025mmol, 10 mol%), **N2** (3 mg, 0.02mmol, 0.2 eq.), AgOAc (83.5 mg, 0.5 mmol, 2 eq.) and the reaction was stirred for 48 h, providing the arylated product in 55% ^{1}H NMR yield. Purification by column chromatography on silica gel using Cy / DCM (9:1-7:3 v/v) as an eluent provided the title compound as a white solid (40.2 mg, 52% yield). **R$_f$** = 0.41 (Cy / DCM = 1:1). **^{1}H NMR** (400 MHz, $CDCl_3$) δ 8.12 (d, *J* = 8.5 Hz, 2H), 7.65 (d, *J* = 8.5 Hz, 2H), 7.44 (s, 1H), 7.29 (s, 1H), 7.15 (s, 1H), 3.95 (s, 3H), 3.91 (s, 3H). **^{13}C NMR** (101 MHz, $CDCl_3$) δ 166.89, 160.37, 144.19, 142.49, 132.60 (q, J_{C-F} = 32.4 Hz), 130.38, 129.90, 127.31, 129.73 – 117.56 (m), 116.71, 116.57 (q, J_{C-F} = 3.9 Hz), 110.16 (q, J_{C-F} = 3.7 Hz), 55.83, 52.37. ^{19}F NMR (376 MHz, $CDCl_3$) δ -62.69. **HRMS** (EI): *m/z* calculated for $C_{16}H_{13}F_3O_5$ $[M]^+$= 310.0817; found= 310.0804. **IR** (neat): v_{max} (cm^{-1}): 2953, 2848, 1719, 1602, 1570, 1461, 1436, 1401, 1358, 1323, 1309, 1279, 1259, 1216, 1165, 1105, 1074, 1038, 1017, 997, 967, 897, 879, 851, 814, 772, 715, 701, 670, 489. **M.P.** = 77−78 °C

A parallel reaction **without ligand** was also performed providing no title compound.

Dimethyl 5-methoxy-[1,1'-biphenyl]-3,4'-dicarboxylate (3j)

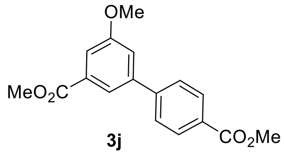

3j

General procedure **A** was followed using methyl 3-methoxybenzoate (72.7 µL, 0.5 mmol, 2.0 eq.) as substrate and methyl 4-iodobenzoate (65.5 mg, 0.25 mmol, 1.0 eq.), using 10 mol% of $Pd(OAc)_2$ (5.6 mg, 0.025 mmol, 10 mol%) and **L2** (250 µL, 0.1 M, 0.025 mmol, 10 mol%) the reaction was stirred for 48 h, providing the arylated product in 55% ^{1}H NMR yield. Purification by column chromatography on silica gel using Cy / DCM (9:1-4:1 v/v) as an eluent provided the title compound as a white solid (39.8 mg, 52% yield). **R$_f$** = 0.44 (Cy / EtOAc = 7:3. **^{1}H NMR** (400 MHz, $CDCl_3$) δ 8.11 (d, *J* = 8.2 Hz, 2H), 7.90 (s, 1H), 7.67 (d, *J* = 8.2 Hz, 2H), 7.58 (s, 1H), 7.34 (s, 1H), 3.95 (s, 6H), 3.91 (s, 3H). **^{13}C NMR** (101 MHz, $CDCl_3$) δ 166.97, 166.85, 160.20, 144.51, 141.72, 132.19, 130.30, 129.61, 127.26, 121.14, 118.46, 113.58, 55.82, 52.49, 52.33 **HRMS** (EI): *m/z* calculated for $C_{17}H_{16}O_5$ $[M]^+$= 300.0998; found= 300.0965. **IR** (neat): v_{max} (cm^{-1}): 3000, 2952, 2842, 2340, 2324, 1719, 1594, 1568, 1456, 1434, 1398, 1341, 1319, 1278, 1241, 1214, 1183, 1106, 1077, 1046, 1018, 990, 889, 853, 819, 765, 753, 705, 687, 638, 607, 534, 489. **M.P.** = 120−121 °C

A parallel reaction **without ligand** was also performed providing traces of the title compound.

Methyl 3',4',5'-trimethoxy-[1,1'-biphenyl]-4-carboxylate (3k)

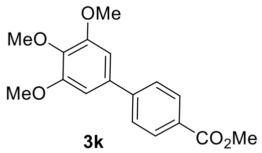

3k

General procedure **A** was followed using 1,2,3-trimethoxybenzene (42.1 µL, 0.25 mmol, 1.0 eq.) as substrate and methyl 4-iodobenzoate (100.3 mg, 0.375 mmol, 1.5 eq.), providing the arylated product in 88% ^{1}H NMR yield. Purification by column chromatography on silica gel using Cy / EtOAc (9:1-3:2 v/v) as an eluent provided the title compounds as a pale yellow solid (66.4 mg, 88% yield). **R$_f$** = 0.4 (Cy / EtOAc = 7:3). **^{1}H NMR** (400 MHz, $CDCl_3$) δ 8.09 (d, *J* = 8.5 Hz, 2H), 7.62 (d, *J* = 8.5 Hz, 2H), 6.80 (s, 2H), 3.94 (m, 9H), 3.90 (s, 3H). **^{13}C NMR** (101 MHz, $CDCl_3$) δ 167.06, 153.71, 145.82, 138.47, 136.00, 130.19, 129.00, 127.08, 104.72, 61.11, 56.39, 52.28. **HRMS** (FI): *m/z* calculated for $C_{17}H_{18}O_5$ $[M]^+$= 302.1154; found= 302.1144. **IR** (neat): ν_{max} (cm^{-1}): 2995, 2949, 2838, 1715, 1608, 1586, 1564, 1519, 1496, 1453, 1433, 1397, 1343, 1274, 1242, 1215, 1184, 1122, 1104, 1079, 1005, 966, 879, 867, 829, 770, 741, 707, 687, 533, 503. **M.P.** = 100−103 °C

A parallel reaction **without ligand** was also performed providing no title compound.

Methyl 3',4'-difluoro-5'-methoxy-[1,1'-biphenyl]-4-carboxylate (3l)

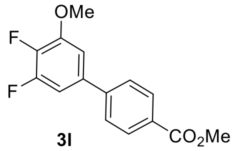

3l

General procedure A was followed using 2,3-difluoroanisole (36.8 mg, 0.25 mmol, 1.0 eq.) as substrate and methyl 4-iodobenzoate (100.3 mg, 0.375 mmol, 1.5 eq.), using 10 mol% of $Pd(OAc)_2$ (5.6 mg, 0.025 mmol, 10 mol%) and **L2** (250 µL, 0.1 M, 0.025mmol, 10 mol%), providing the arylated product in 64% ^{1}H NMR yield. Purification by column chromatography on silica gel using Cy / EtOAc (98:2 v/v) as an eluent provided the title compound as a white solid (44.8 mg, 65% yield). **R$_f$** = 0.23 (Cy / DCM = 1:1). **^{1}H NMR** (400 MHz, $CDCl_3$) δ 8.09 (d, *J* = 8.4 Hz, 2H), 7.57 (d, *J* = 8.4 Hz, 2H), 7.00 (ddd, *J* = 10.6, 6.5, 2.1 Hz, 1H), 6.95 (dt, *J* = 6.8, 1.9 Hz, 1H), 3.97 (s, 3H), 3.94 (s, 3H). **^{13}C NMR** (101 MHz, $CDCl_3$) δ 166.83, 151.44 (dd, J_{C-F} = 246.9, 10.6 Hz), 149.52 (dd, J_{C-F} = 8.0, 4.0 Hz), 143.99, 141.25 (dd, J_{C-F} = 249.6, 14.5 Hz), 135.97 (dd, J_{C-F} = 8.2, 4.8 Hz), 130.33, 129.65, 127.03, 108.26 (d, J_{C-F} = 18.9 Hz), 107.73 (d, J_{C-F} = 2.4 Hz), 56.92, 52.34. **^{19}F NMR** (376 MHz, $CDCl_3$) δ-136.51 – -136.64 (m), -160.23 (dt, *J* = 20.0, 6.7 Hz)· **HRMS** (FI): *m/z* calculated for $C_{15}H_{12}F_2O_3$ $[M]^+$= 278.0755; found= 278.0751. **IR** (neat): ν_{max} (cm^{-1}): 2958, 2848, 1718, 1624, 1571, 1532, 1507, 1473, 1457, 1434, 1401, 1362, 1318, 1284, 1250, 1233, 1189, 1103, 1065, 1019, 970, 876, 832, 770, 705, 689, 533. **M.P.** = 132−134 °C.

A parallel reaction **without ligand** was also performed providing traces of title compound.

Methyl 3',4'-dichloro-5'-methoxy-[1,1'-biphenyl]-4-carboxylate (3m)

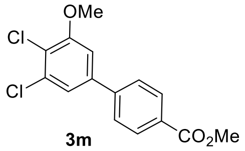

3m

General procedure **A** was followed using 2,3-dichloroanisole (45.6 mg, 0.25 mmol, 1.0 eq.) as substrate and methyl 4-iodobenzoate (100.3 mg, 0.375 mmol, 1.5 eq.), using 10 mol% of $Pd(OAc)_2$ (5.6 mg, 0.025 mmol, 10 mol%) and **L2** (250 µL, 0.1 M, 0.025 mmol, 10 mol%) and with **N3** (11.3mg, 0.075mmol, 0.3 eq.), providing the arylated product in 58% ^{1}H NMR yield. Purification by column chromatography on silica gel using Cy / DCM (9:1-4:1 v/v) as an eluent provided the title compound as a white solid (43.9 mg, 57% yield). **R$_f$** = 0.37 (Cy / DCM = 1:1). **^{1}H NMR** (400 MHz, $CDCl_3$) δ 8.10 (d, *J* = 8.4 Hz, 3H), 7.59 (d, *J* = 8.4 Hz, 2H), 7.31 (s, 1H), 7.02 (s, 1H), 3.97 (s, 3H), 3.94 (s, 3H). **^{13}C NMR** (101 MHz, $CDCl_3$) δ 166.77, 156.68, 143.70, 139.90, 134.34, 130.35, 129.88, 127.08, 121.63, 121.16, 109.09, 56.77, 52.36. **HRMS** (FI): *m/z* calculated for $C_{15}H_{12}Cl_2O_3$ $[M]^+$= 310.0163; found= 310.0156. **IR** (neat): ν_{max} (cm^{-1}): 2999, 2954, 2845, 1720, 1611, 1591, 1579, 1556, 1455, 1445, 1434, 1420, 1391, 1314, 1277, 1238, 1185, 1106, 1051, 1037, 1017, 971, 876, 837, 813, 769, 704, 652, 639, 575. **M.P.** = 159 **M.P.** = 160 °C

A parallel reaction **without ligand** was also performed providing traces of the title compound.

Methyl 3'-chloro-5'-methoxy-4'-methyl-[1,1'-biphenyl]-4-carboxylate (3n)

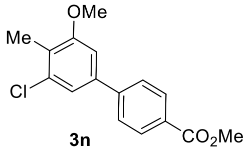

3n

General procedure **A** was followed using 2-methyl-3-chloroanisole (35.5 µL, 0.25 mmol, 1.0 eq.) as substrate and methyl 4-iodobenzoate (100.3 mg, 0.375 mmol, 1.5 eq.), using 10 mol% of $Pd(OAc)_2$ (5.6 mg, 0.025 mmol, 10 mol%) and **L2** (250 µL, 0.1 M, 0.025 mmol, 10 mol%) providing the arylated product in 92% ^{1}H NMR yield. Purification by column chromatography on silica gel using Cy / EtOAc (97:3 v/v) as an eluent provided the title compound as clear crystals (63.4 mg, 88% yield). **R$_f$** = 0.22 (Cy / DCM = 1:1). **^{1}H NMR** (400 MHz, $CDCl_3$) δ 8.09 (d, *J* = 8.5 Hz, 2H), 7.61 (d, *J* = 8.5 Hz, 2H), 7.23 (d, *J* = 1.5 Hz, 1H), 6.95 (d, *J* = 1.5 Hz, 1H), 3.94 (s, 3H), 3.90 (s, 3H), 2.31 (s, 3H). **^{13}C NMR** (101 MHz, $CDCl_3$) δ 166.95, 158.85, 144.68, 139.06, 135.68, 130.24, 129.33, 126.98, 125.17, 120.29, 107.53, 56.03, 52.28, 12.72. **HRMS** (FI): *m/z* calculated for $C_{16}H_{15}ClO_3$ $[M]^+$= 290.0710; found= 290.0708. **IR** (neat): ν_{max} (cm^{-1}): 3000, 2951, 2922, 2844, 1718, 1604, 1575, 1554, 1461, 1433, 1417, 1390, 1312, 1277, 1103, 1070, 1046, 889, 872, 832, 817, 767, 740. **M.P.** = 119−120 °C.

A parallel reaction **without ligand** was also performed providing traces of the titled compound.

Methyl 4-(4-methoxy-5,6,7,8-tetrahydronaphthalen-2-yl)benzoate (3o)

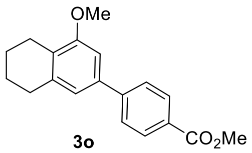

3o

General procedure **A** was followed using 5-methoxy-1,2,3,4-tetrahydronaphthalene (37.0 mg, 0.25 mmol, 1.0 eq.) as substrate and methyl 4-iodobenzoate (100.3 mg, 0.375 mmol, 1.5 eq.) using 10 mol% of $Pd(OAc)_2$ (5.6 mg, 0.025 mmol, 10 mol%) and **L2** (250 µL, 0.1 M, 0.025 mmol, 10 mol%), providing the arylated product in 67% ^{1}H NMR yield. Purification by column chromatography on silica gel using Cy / DCM (9:1-7:3 v/v) as an eluent provided the title compound as white solid (45.4 mg, 64% yield). **R$_f$** = 0.47 (Cy / EtOAc = 9:1). **^{1}H NMR** (400 MHz, $CDCl_3$) δ 8.08 (d, *J* = 8.5 Hz, 2H), 7.64 (d, *J* = 8.5 Hz, 2H), 6.97 (s, 1H), 6.88 (d, *J* = 1.7 Hz, 1H), 3.94 (s, 3H), 3.89 (s, 3H), 2.82 (t, *J* = 5.7 Hz, 2H), 2.74 – 2.66 (m, 2H), 1.81 (dq, *J* = 6.0, 3.8, 2.4 Hz, 4H). **^{13}C**

NMR (101 MHz, $CDCl_3$) δ 167.21, 157.97, 146.27, 139.13, 137.92, 130.14, 128.73, 127.06, 126.44, 120.55, 105.99, 55.53, 52.24, 29.97, 23.20, 22.98, 22.89. ^{1}H NMR of the isolated material matched with that reported in the literature.[25]

A parallel reaction **without ligand** was also performed providing 11% NMR yield of the title compound.

Methyl 4-(7-methoxy-1-oxo-2,3-dihydro-1H-inden-5-yl)benzoate (3p)

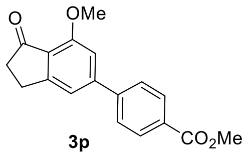

General procedure **A** was followed using 7-methoxy-1-indanone (40.7 mg, 0.25 mmol, 1.0 eq.) as substrate and methyl 4-iodobenzoate (100.3 mg, 0.375 mmol, 1.5 eq.), using 10 mol% of $Pd(OAc)_2$ (5.6 mg, 0.025 mmol, 10 mol%) and **L2** (250 µL, 0.1 M, 0.025 mmol, 10 mol%), providing the arylated product in 39% ^{1}H NMR yield. Purification by column chromatography on silica gel using Cy / EtOAc (9:1-7:3 v/v) as an eluent provided the title compound as yellow solid (31.4 mg, 42% yield). **R$_f$** = 0.15 (Cy / EtOAc = 1:1). **^{1}H NMR** (300 MHz, $CDCl_3$) δ 8.14 (d, J = 8.5 Hz, 2H), 7.68 (d, J = 8.5 Hz, 2H), 7.24 (s, 1H), 6.97 (s, 1H), 4.03 (s, 3H), 3.96 (s, 3H), 3.23 – 3.10 (m, 2H), 2.79 – 2.69 (m, 2H). **^{13}C NMR** (101 MHz, $CDCl_3$) δ 204.43, 166.85, 158.68, 158.41, 148.61, 144.89, 130.33, 130.19, 127.62, 124.88, 117.65, 108.50, 56.06, 52.42, 37.18, 25.84. **HRMS** (FI): *m/z* calculated for $C_{18}H_{16}O_4$ $[M]^{+}$= 296.1049; found= 296.1056. **IR** (neat): ν_{max} (cm^{-1}): 2951, 1714, 1601, 1588, 1566, 1465, 1435, 1397, 1334, 1278, 1236, 1195, 1108, 1083, 1061, 1020, 847, 773, 729, 707. **M.P.** = 197−198 °C.

A parallel reaction **without ligand** was also performed providing traces of the title compound.

Table S10. Overview of aryl-halide scope

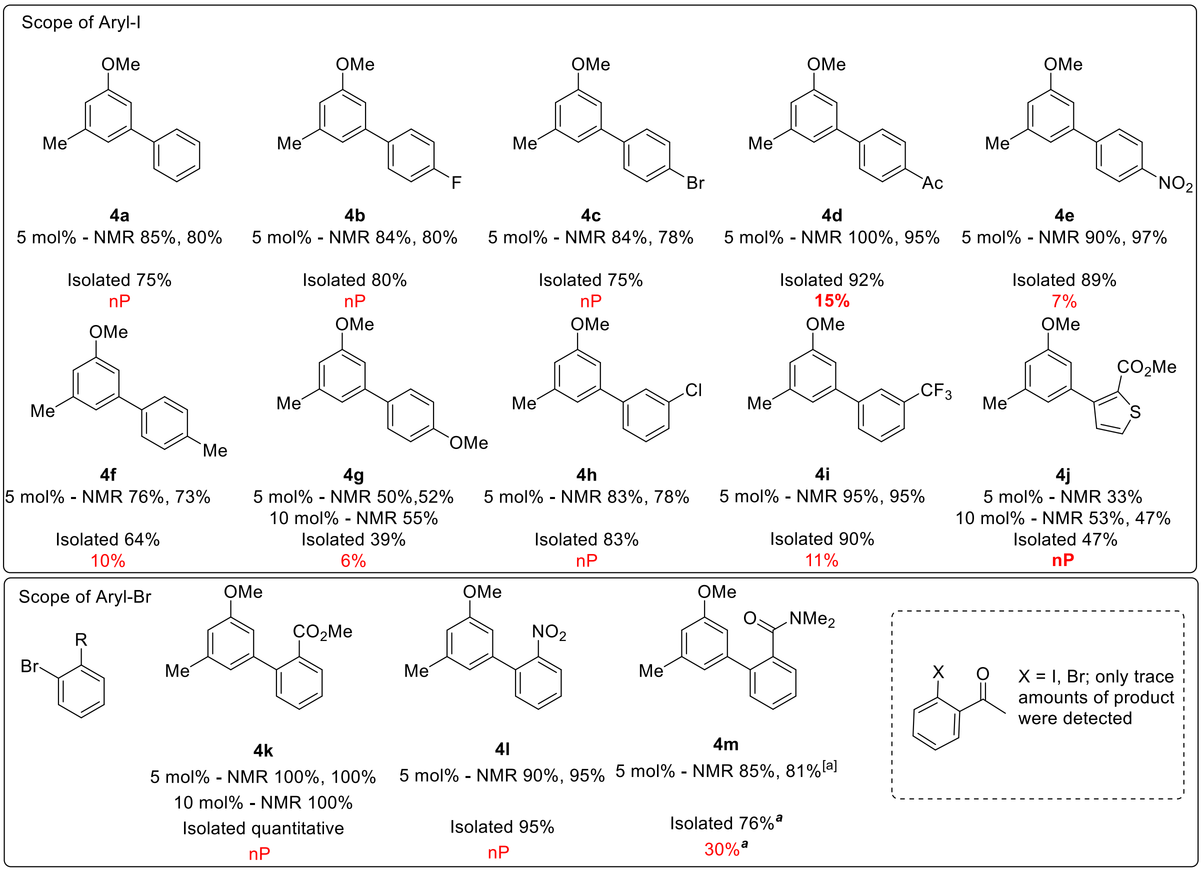

All reaction were first performed on a 0.1 mmol scale. Using the final selected conditions, the reaction was repeated on 0.25 mmol scale and the isolated. The yields in red corresponds to the reaction without ligand.[a] 1.0 eq. aryl-Br **2m** and 1.5 eq. of 2-methylanisole **1b** was used, for easier purification.

3-Methoxy-5-methyl-1,1'-biphenyl (4a)

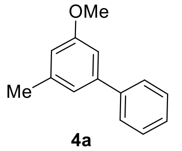

4a

General procedure **A** was followed using 3-methylanisole (31.8 μL, 0.25 mmol, 1.0 eq.) as substrate and Iodobenzene (42.8 μL, 0.375 mmol, 1.5 eq.), providing the arylated product in 83% ^{1}H NMR yield. Purification by column chromatography on silica gel using Cy / DCM (25:1 v/v) as an eluent provided the title compound as a light yellow oil (37.3 mg, 75% yield). **R$_f$** = 0.60 (Cy / DCM = 1:1). **^{1}H NMR** (400 MHz, $CDCl_3$) δ 7.61 (d, *J* = 7.5 Hz, 2H), 7.45 (t, *J* = 7.5 Hz, 2H), 7.36 (t, *J* = 7.3 Hz, 1H), 7.04 (s, 1H), 6.97 (s, 1H), 6.76 (s, 1H), 3.87 (s, 3H), 2.43 (s, 3H). **^{13}C NMR** (101 MHz, $CDCl_3$) δ 160.09, 142.71, 141.39, 139.87, 128.79, 127.44, 127.33, 120.77, 113.70, 110.09, 55.40, 21.81.**HRMS** (FI): *m/z* calculated for $C_{14}H_{14}O$ $[M]^+$= 198.0950; found= 198.1045. **IR** (neat): ν_{max} (cm^{-1}): 2920, 2836, 1595, 1576, 1498, 1465, 1419, 1334, 1216, 1166, 1153, 1063, 1053, 1030, 848, 762, 697, 648.

A parallel reaction **without ligand** was also performed providing no title compound.

4'-Fluoro-3-methoxy-5-methyl-1,1'-biphenyl (4b)

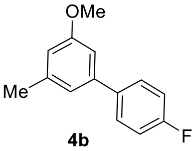

4b

General procedure **A** was followed using 3-methylanisole (31.8 μL, 0.25 mmol, 1.0 eq.) as substrate and 4-fluoroiodobenzene (43.2 μL, 0.375 mmol, 1.5 eq.), providing the arylated product in 82% ^{1}H NMR yield. Purification by column chromatography on silica gel using Cy:DCM (25:1 v/v) as an eluent provided the title compound as a colorless oil (43.4 mg, 80% yield). **R$_f$** = 0.74 (Cy / DCM = 1:1). **^{1}H NMR** (300 MHz, $CDCl_3$) δ 7.55 (dd, *J* = 8.9, 5.4 Hz, 2H), 7.13 (t, *J* = 8.8 Hz, 2H), 6.97 (s, 1H), 6.90 (s, 1H), 6.74 (s, 1H), 3.86 (s, 3), 2.41 (s, 3H). ^{1}H NMR of the isolated material matched with that reported in the literature.[25]

A parallel reaction **without ligand** was also performed providing no title compound.

4'-Bromo-3-methoxy-5-methyl-1,1'-biphenyl (4c)

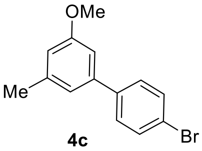

4c

General procedure **A** was followed using 3-methylanisole (31.8 μL, 0.25 mmol, 1.0 eq.) as substrate and 4-bromoiodobenzene (106.1 mg, 0.375 mmol, 1.5 eq.), providing the arylated product in 80% ^{1}H NMR yield. Purification by column chromatography on silica gel using Cy / DCM (25:1 v/v) as an eluent provided the title compound as a white solid (52.1 mg, 75% yield). **R$_f$** = 0.55 (Cy / DCM = 1:1). **^{1}H NMR** (400 MHz, $CDCl_3$) δ 7.55 (d, *J* = 8.6 Hz, 2H), 7.45 (d, *J* = 8.6 Hz, 2H), 6.97 (s, 1H), 6.90 (s, 1H), 6.75 (s, 1H), 3.85 (s, 3H), 2.41 (s, 3H). **^{13}C NMR** (101 MHz, $CDCl_3$) δ 160.19, 141.42, 140.28, 140.10, 131.89, 128.89, 121.66, 120.49, 113.97, 109.97, 55.43, 21.80. **HRMS** (FI): *m/z* calculated for $C_{14}H_{13}BrO$ $[M]^+$= 276.0150; found= 276.0151. **IR** (neat): ν_{max} (cm^{-1}): 2920, 2836, 1594, 1563, 1492, 1465, 1386, 1335, 1310, 1215, 1187, 1167, 1154, 1105, 1071, 1048, 1008, 856, 818, 693, 552, 470. **M.P.** = 66−68 °C.

A parallel reaction **without ligand** was also performed providing no title compound.

1-(3'-Methoxy-5'-methyl-[1,1'-biphenyl]-4-yl)ethenone (4d)

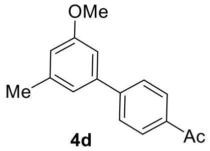

4d

General procedure **A** was followed using 3-methylanisole (31.8 μL, 0.25 mmol, 1.0 eq.) as substrate and 4′-iodoacetophenone (92.3 mg, 0.375 mmol, 1.5 eq.), providing the arylated product in 97% ^{1}H NMR yield. Purification by column chromatography on silica gel using Cy / DCM (9:1 v/v) as an eluent provided the title compound as pale orange crystals (55.4 mg, 92% yield). **R$_f$** = 0.18 (Cy / DCM = 1:1). ^{1}H NMR (300 MHz, $CDCl_3$) δ 8.01 (d, *J* = 8.5 Hz, 2H), 7.66 (d, *J* = 8.5 Hz, 2H), 7.03 (s, 1H), 6.96 (s, 1H), 6.77 (s, 1H), 3.85 (s, 3H), 2.63 (s, 3H), 2.41 (s, 3H). ^{1}H NMR of the isolated material matched with that reported in the literature.[25]

A parallel reaction **without ligand** was also performed providing 15% of the title compound.

3-Methoxy-5-methyl-4'-nitro-1,1'-biphenyl (4e)

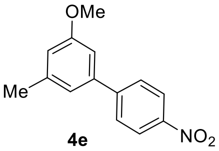

4e

General procedure **A** was followed using 3-methylanisole (31.8 μL, 0.25 mmol, 1.0 eq.) as substrate and 1-iodo-4-nitrobenzene (93.4 mg, 0.375 mmol, 1.5 eq.), providing the arylated product in 95% ^{1}H NMR yield. Purification by column chromatography on silica gel using Cy / EtOAc (50:1 v/v) as an eluent provided the title compound as a yellow solid (54.2 mg, 89% yield). **R$_f$** = 0.36 (Cy / DCM = 1:1). **^{1}H NMR** (300 MHz, $CDCl_3$) δ 8.26 (d, *J* = 8.9 Hz, 2H), 7.70 (d, *J* = 8.9 Hz, 2H), 7.02 (s, 1H), 6.94 (s, 1H), 6.81 (s, 1H), 3.86 (s, 3H), 2.42 (s, 3H). ^{1}H NMR of the isolated material matched with that reported in the literature.[25]

A parallel reaction **without ligand** was also performed providing 7% of the title compound.

3-Methoxy-4',5-dimethyl-1,1'-biphenyl (4f)

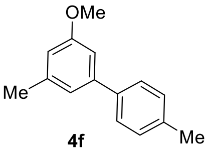

4f

General procedure **A** was followed using 3-methylanisole (31.8 μL, 0.25 mmol, 1.0 eq.) as substrate and 4-iodotoluene (81.8 mg, 0.375 mmol, 1.5 eq.), providing the arylated product in 73% ^{1}H NMR yield. Purification by column chromatography on silica gel using Cy / DCM (50:1 v/v) as an eluent provided the title compound as a light yellow oil (38.1 mg, 64% yield). **R$_f$** = 0.68 (Cy / DCM = 1:1). **^{1}H NMR** (400 MHz, $CDCl_3$) δ 7.50 (d, *J* = 8.1 Hz, 2H), 7.26 (d, *J* = 7.9 Hz, 2H), 7.02 (s, 1H), 6.95 (s, 1H), 6.73 (s, 1H), 3.86 (s, 3H), 2.42 (s, 6H). ^{1}H NMR of the isolated material matched with that reported in the literature.[25]

A parallel reaction **without ligand** was also performed providing 10% of the title compound.

3,4'-Dimethoxy-5-methyl-1,1'-biphenyl (4g)

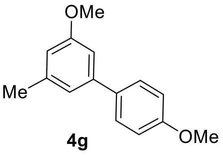

4g

General procedure **A** was followed using 3-methylanisole (31.8 µL, 0.25 mmol, 1.0 eq.) as substrate and 4-iodoanisole (87.8 mg, 0.375 mmol, 1.5 eq.), providing the arylated product in 52% ^{1}H NMR yield. Purification by column chromatography on silica gel using Cy / DCM (25:1 v/v) as an eluent provided the title compound as a light orange oil (22.2 mg, 39% yield). **R$_f$** = 0.54 (Cy / DCM = 1:1). **^{1}H NMR** (400 MHz, $CDCl_3$) δ 7.52 (d, *J* = 8.8 Hz, 2H), 7.00 – 6.93 (m, 3H), 6.90 (t, *J* = 2.0 Hz, 1H), 6.69 (s, 1H), 3.85 (s, *J* = 2.3 Hz, 3H + 3H), 2.39 (s, 3H). **^{13}C NMR** (101 MHz, $CDCl_3$) δ 160.09, 159.31, 142.30, 139.83, 133.92, 128.32, 120.39, 114.24, 113.08, 109.73, 55.49, 55.41, 21.83. **HRMS** (FI): *m/z* calculated for $C_{15}H_{16}O_2$ $[M]^+$= 228.1150; found= 228.1155. **IR** (neat): ν_{max} (cm^{-1}): 2935, 2836, 1608, 1594, 1576, 1515, 1463, 1440, 1335, 1290, 1247, 1215, 1180, 1166, 1154, 1062, 1052, 1034, 826.

A parallel reaction **without ligand** was also performed providing traces (<5%) of the title compound.

3'-Chloro-3-methoxy-5-methyl-1,1'-biphenyl (4h)

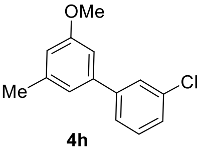

4h

General procedure **A** was followed using 3-methylanisole (31.8 µL, 0.25 mmol, 1.0 eq.) as substrate and 3-chloroiodobenzene (46.3 µL, 0.375 mmol, 1.5 eq.), providing the arylated product in 80% ^{1}H NMR yield. Purification by column chromatography on silica gel using Cy / EtOAc (50:1 v/v) as an eluent provided the title compound as a colorless oil (48.3 mg, 83% yield). **R$_f$** = 0.62 (Cy / DCM = 1:1). **^{1}H NMR** (300 MHz, $CDCl_3$) δ 7.56 (t, *J* = 1.9 Hz, 1H), 7.45 (dt, *J* = 7.2, 1.8 Hz, 1H), 7.39 – 7.28 (m, 2H), 6.97 (s, 1H), 6.90 (s, 1H), 6.75 (s, 1H), 3.85 (s, 3H), 2.40 (s, 3H). ^{1}H NMR of the isolated material matched with that reported in the literature.[25]

A parallel reaction **without ligand** was also performed providing no title compound.

3-Methoxy-5-methyl-3'-(trifluoromethyl)-1,1'-biphenyl (4i)

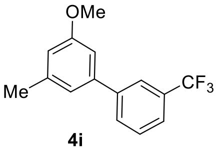

4i

General procedure **A** was followed using 3-methylanisole (31.8 µL, 0.25 mmol, 1.0 eq.) as substrate and 3-iodobenzotrifluoride (54.0 µL, 0.375 mmol, 1.5eq.), providing the arylated product in 95% ^{1}H NMR yield. Purification by column chromatography on silica gel using Cy / EtOAc (25:1 v/v) as an eluent provided the title compound as a colorless oil (59.9 mg, 90% yield). **R$_f$** = 0.53 (Cy / EtOAc = 9:1). **^{1}H NMR** (400 MHz, $CDCl_3$) δ 7.82 (s, 1H), 7.75 (d, *J* = 7.6 Hz, 1H), 7.60 (d, *J* = 7.8 Hz, 1H), 7.54 (t, *J* = 7.7 Hz, 1H), 7.00 (s, 1H), 6.92 (s, 1H), 6.77 (s, 1H), 3.86 (s, 3H), 2.42 (s, 3H). **^{19}F NMR** (376 MHz, $CDCl_3$) δ -62.56. **^{13}C NMR** (101 MHz, $CDCl_3$) δ 160.24, 142.17, 141.19, 140.28, 131.18 (q, $J_{C\text{-}F}$ = 32.2 Hz), 130.61, 130.59, 129.27, 124.34 (q, $J_{C\text{-}F}$ = 272.6 Hz), 124.11 (q, $J_{C\text{-}F}$ = 3.8 Hz), 120.72, 114.34, 110.19, 55.49, 21.81. **HRMS** (FI): *m/z* calculated for $C_{15}H_{13}F_3O$ $[M]^+$= 266.0918; found= 266.0905. **IR** (neat): ν_{max} (cm^{-1}): 2942, 2839, 1595, 1493, 1463, 1411, 1380, 1344, 1321, 1280, 1258, 1214, 1164, 1123, 1097, 1074, 1003, 936, 905, 889, 864, 847, 801, 701, 675, 645.

A parallel reaction **without ligand** was also performed providing 11% of the title compound.

Methyl 3-(3-methoxy-5-methylphenyl) thiophene-2-carboxylate (4j)

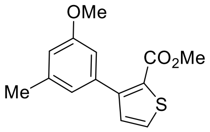

4j

General procedure **A** was followed using 3-methylanisole (31.8 µL, 0.25 mmol, 1.0 eq.) as substrate and methyl 3-iodothiophene-2-carboxylate (100.5 mg, 0.375 mmol, 1.5 eq.), using 10 mol% of $Pd(OAc)_2$ (5.6 mg, 0.025 mmol, 10 mol%) and **L2** (250 µL, 0.1 M, 0.025mmol, 10 mol%) providing the arylated product in 50% ^{1}H NMR yield. Purification by column chromatography on silica gel using Cy / DCM (4:1 - 3:2 v/v) as an eluent provided the title compound as a white solid (30.9 mg, 47% yield). **R$_f$** = 0.23 (Cy / DCM = 1:1). **^{1}H NMR** (400 MHz, $CDCl_3$) δ 7.49 (d, *J* = 5.1 Hz, 1H), 7.08 (d, *J* = 5.1 Hz, 1H), 6.85 (s, 1H), 6.82 (s, 1H), 6.74 (s, 1H), 3.81 (s, 3H), 3.78 (s, 3H). **^{13}C NMR** (101 MHz, $CDCl_3$) δ 162.54, 159.19, 148.68, 138.93, 136.85, 131.72, 130.18, 127.15, 122.60, 114.57, 112.17, 55.39, 52.06, 21.72. **HRMS** (FI): *m/z* calculated for $C_{14}H_{14}O_3S$ $[M]^+$= 262.0664; found= 262.0656. **IR** (neat): ν_{max} (cm^{-1}): 2950, 1718, 1698, 1592, 1534, 1455, 1434, 1405, 1372, 1320, 1300, 1276, 1260, 1236, 1214, 1189, 1166, 1153, 1118, 1094, 1064, 926, 848, 793, 776, 757, 698, 672. **M.P.** = 97−95 °C.

A parallel reaction **without ligand** was also performed providing no title compound.

Methyl 3'-methoxy-5'-methyl-[1,1'-biphenyl]-2-carboxylate (4k)

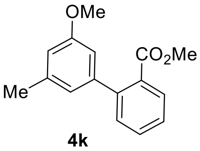

4k

General procedure **A** was followed using 3-methylanisole (31.8 µL, 0.25 mmol, 1.0 eq.) as substrate and methyl 2-bromobenzoate (52.7 µL, 0.375 mmol, 1.5 eq.), providing the arylated product in 100% ^{1}H NMR yield. Purification by column chromatography on silica gel using Cy / Et_2O (25:1 v/v) as an eluent provided the title compound as a colorless oil (64.0 mg, quantitative yield). **R$_f$** = 0.023 (Cy / Et_2O = 9:1). **^{1}H NMR** (400 MHz, $CDCl_3$) δ 7.78 (dd, *J* = 7.3, 1.5 Hz, 1H), 7.51 (td, *J* = 7.3, 1.5 Hz, 1H), 7.43 – 7.37 (m, 2H), 6.73 (m, 2H), 6.68 (t, *J* = 1.8 Hz, 1H), 3.81 (s, 3H), 3.67 (s, 3H), 2.37 (s, 3H). **^{13}C NMR** (101 MHz, $CDCl_3$) δ 169.47, 159.42, 142.51, 142.36, 139.20, 131.23, 131.18, 130.61, 129.63, 127.26, 121.81, 113.91, 111.02, 55.32, 52.14, 21.69. **HRMS** (FI): *m/z* calculated for $C_{16}H_{16}O_3$ $[M]^+$= 256.1099; found= 256.1097. **IR** (neat): ν_{max} (cm^{-1}): 2921, 2851, 1720, 1609, 1592, 1571, 1458, 1432, 1334, 1290, 1261, 1246, 1212, 1189, 1165, 1153, 1124, 1097, 1074, 1051, 1037, 964, 818, 791, 761, 725, 698.

A parallel reaction **without ligand** was also performed providing no title compound.

3'-Methoxy-5'-methyl-2-nitro-1,1'-biphenyl (4l)

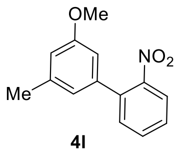

4l

General procedure **A** was followed using 3-methylanisole (31.8 µL, 0.25 mmol, 1.0 eq.) as substrate and 1-bromo-2-nitrobenzene (75 mg, 0.375 mmol, 1.5 eq.), providing the arylated product in 95% ^{1}H NMR yield. Purification by column chromatography on silica gel using Cy / DCM (25:1 – 9:1 v/v) as an eluent provided the title compound as a yellow oil (57.9 mg, 95% yield). **R$_f$** = 0.40 (Cy / DCM = 1:1). **^{1}H NMR** (400 MHz, $CDCl_3$) δ 7.82 (dd, *J* = 8.0, 1.1 Hz, 1H), 7.59 (td, *J* = 7.6, 1.3 Hz, 1H), 7.57-7.43 (m, 2H), 6.76 (s, 1H), 6.72 (s, 1H), 6.67 (s, 1H), 3.80 (s, 3H), 2.36 (s, 3H). **^{13}C NMR** (101 MHz, $CDCl_3$) δ 159.79, 149.47, 139.98, 138.54, 136.40, 132.25, 131.93, 128.21, 124.03, 121.18, 114.73, 110.77, 55.37, 21.69. **HRMS** (FI): *m/z* calculated for $C_{14}H_{13}NO_3$ $[M]^+$= 243.0895; found= 243.0889. **IR** (neat): ν_{max} (cm^{-1}): 2938, 2838, 1593, 1572, 1522, 1462, 1422, 1354, 1335, 1294, 1254, 1214, 1187, 1167, 1154, 1073, 1049, 1037, 994, 944, 933, 868, 841, 781, 750, 714, 695, 685, 638, 592, 541.

A parallel reaction **without ligand** was also performed providing no title compound.

3'-Methoxy-N, N,5'-trimethyl-[1,1'-biphenyl]-2-carboxamide (4m)

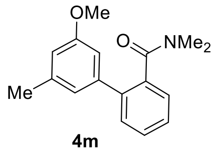

4m

General procedure **A** was followed using 3-methylanisole (31.8 µL, 0.25 mmol, 1.0 eq.) as substrate and 2-bromo-N,N-dimethylbenzamide (85.5 mg, 0.375 mmol, 1.5 eq.), providing the arylated product in 85% ^{1}H NMR yield. However, purification by column chromatography did not successfully separate the product from the 2-bromo-*N*,*N*-dimethylbenzamide. Therefore, the reaction was repeated using 3-methylanisole (47.8 µL, 0.375 mmol, 1.5 eq.) and 2-bromo-N,N-dimethylbenzamide (57.0 mg, 0.25 mmol, 1.0 eq.), providing the arylated product in 81% ^{1}H NMR yield. Purification by column chromatography on silica gel using Cy / EtOAc (4:1 – 1:1 v/v) as an eluent provided the title compound as a yellow oil (51.4 mg, 76% yield). **R$_f$** = 0.30 (Cy / DCM = 1:1). **^{1}H NMR** (300 MHz, $CDCl_3$) δ 7.47 – 7.35 (m, 4H), 6.87 (s, 1H), 6.83 (s, 1H), 6.72 (s, 1H), 3.80 (s, 3H), 2.88 (s, 3H), 2.46 (s, 3H), 2.35 (s, 3H). **^{13}C NMR** (101 MHz, $CDCl_3$) δ 170.06, 159.66, 141.30, 139.58, 138.88, 135.83, 129.42, 129.40, 127.82, 127.55, 121.89, 114.66, 110.78, 55.46, 38.25, 34.82, 21.70. **HRMS** (FI): *m/z* calculated for $C_{17}H_{19}NO_2$ $[M]^+$= 269.1416; found= 269.1423. **IR** (neat): ν_{max} (cm^{-1}): 2924, 1628, 1607, 1591, 1504, 1485, 1461, 1421, 1393, 1333, 1265, 1218, 1166, 1154, 1118, 1090, 1070, 1058, 1037, 849, 781, 762, 699.

A parallel reaction **without ligand** was also performed providing 30% of the title compound.

7. Optimization of the *meta*-Arylation of 2-Methylanisole (*ortho* substrates)

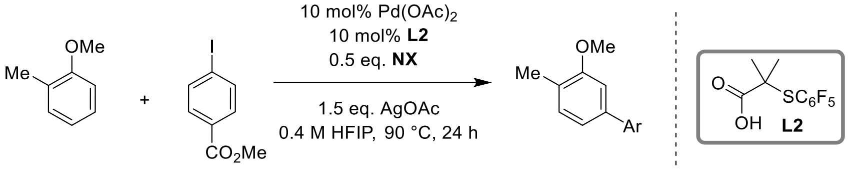

5a **2** 1.5 eq. **6a**

Table S11. Optimization of *meta* arylation with 2-methylanisole **5a**.

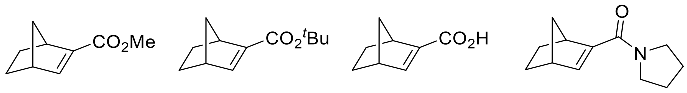

N2 24% yield **N4** traces **N5** 21% yield **N6** 8% yield

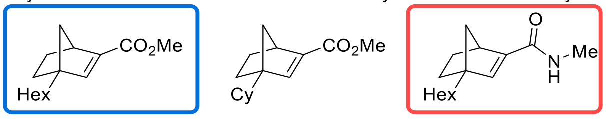

N7 77% yield *m:p* 10:1 **N8** 39% yield *m:p* 10:1 **N9** 98% yield *m:p* 10:1

#	Pd/ **L2** [mol%]	**N7** [eq.]	**N9** [eq.]	Temp. [°C]	Solvent (0.4 M)	^{1}H-NMR yield [%] **6a** (only *meta*-isomer)
1	10	0.5	-	90	HFIP	70
2	10	**0.3**	**-**	90	HFIP	65
3	**5**	0.5	-	90	HFIP	40
4	10	0.5	-	**100**	HFIP	70
5	10	0.5	-	**80**	HFIP	30
6	10	0.5	-	90	**DCE**	21
7	10	0.5	-	90	**HFIP:DCE (1.5:1)**	81
8	10	0.5	-	90	**HFIP:DCM (1.5:1)**	60
9	10	0.5	-	90	**HFIP:$CHCl_3$ (1.5:1)**	77
10	10	0.5	-	90	**HFIP:Pentane (1.5:1)**	76
11	10	0.5	-	90	**HFIP:EtOAc (1.5:1)**	65
12	10	0.5	-	90	**HFIP:^{t}AmOH (1.5:1)**	37
13	10	-	0.5	90	HFIP	90
14	10	-	0.2	90	HFIP	90
15	5	-	0.2	90	HFIP	66

8. General Procedure B for the *meta*-C–H Arylation of Anisole Derivatives with *ortho* Substituents

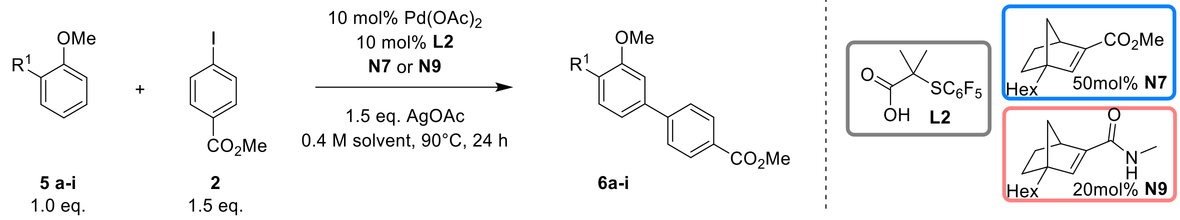

Gerenal Procedure **B-N7**: In a pressure tube containing a suitable stirring bar, **N7** (29.3 mg, 0.125 mmol, 0.5 eq.), $Pd(OAc)_2$ (5.6 mg, 0.025 mmol, 10 mol%), silver acetate (62.6 mg, 0.375 mmol, 1.5 eq.), methyl 4-iodobenzoate (100.3 mg, 0.375 mmol, 1.5 eq.), anisole derivative (0.25 mmol, 1.0 eq.), a stock solution of S,O-ligand **L2** in HFIP (250 µL, 0.1 M, 0.025 mmol, 10 mol%), HFIP (0.125 mL) and DCE (0.25 mL, 0.4 M HFIP / DCE 1.5:1) were added. The tube was put into a pre-heated oil bath at 90 °C and was stirred for 24 h. After cooling to room temperature, the reaction was filtered through Celite® and rinsed with EtOAc. The solvent was evaporated under reduced pressure. To the crude mixture CH_2Br_2 (17.7 µL, 0.25 mmol) was added as internal standard, the mixture was dissolved in $CDCl_3$ and ^1H NMR was measured. Subsequentially, the product was purified by flash column chromatography.

Gerenal Procedure **B-N9**: In a pressure tube containing a suitable stirring bar, **N9** (11.7 mg, 0.125 mmol, 0.2 eq.), $Pd(OAc)_2$ (5.6 mg, 0.025 mmol, 10 mol%), silver acetate (62.6 mg, 0.375 mmol, 1.5 eq.), methyl 4-iodobenzoate (100.3 mg, 0.375 mmol, 1.5 eq.), anisole derivative (0.25 mmol, 1.0 eq.), a stock solution of S,O-ligand **L2** in HFIP (250 µL, 0.1 M, 0.025 mmol, 10 mol%), HFIP (0.375 mL, 0.4M) were added. The tube was put into a pre-heated oil bath at 90 °C and was stirred for 24 h. After cooling to room temperature, the reaction was filtered through Celite® and rinsed with EtOAc. The solvent was evaporated under reduced pressure. To the crude mixture CH_2Br_2 (17.7 µL, 0.25 mmol) was added as internal standard, the mixture was dissolved in $CDCl_3$ and ^1H NMR was measured. Subsequentially, the product was purified by flash column chromatography

Table S12. Scope of *ortho* substituted anisoles

Gerenal procedure **B-N7**: Scope with 50 mol% **N7**, 0.4 M HFIP/ DCE 1.5:1
Gerenal procedure **B-N9**: Scope with 20 mol% **N9**, 0.4 M HFIP

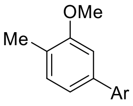	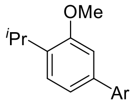	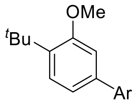	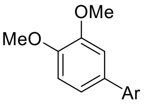
**6a**	**6b**	**6c**	**6d**
NMR - 78%, 80%<br>Isolated 80%<br>m:p 11:1	NMR - 70%, 67%<br>Isolated 64%<br>m:p 12:1	NMR - 70%[a], 70%[a]<br>Isolated 71%[a]	NMR - 67%, 68%<br>Isolated 68%
NMR - 98%, 96%<br>Isolated 92%<br>m:p 10:1	NMR - 95%, 90%<br>Isolated 88%<br>m:p 12:1	NMR - 80%, 88%<br>Isolated 88%	NMR - 70%
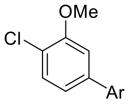	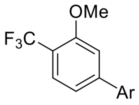	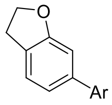	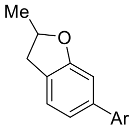
**6e**	**6f**	**6h**	**6i**
NMR - 44%<br>m:p 8:1	NMR - 58%[b,d], 60%[b,d]<br>Isolated 56%[b,d]	NMR - 64%[a], 65%[a]<br>Isolated 61%[a]	NMR - 60%, 60%[a]<br>Isolated 59%
NMR - 48%[b,c], 52%[b,c]<br>Isolated 50%[b,c]<br>m:p 7:1	NMR - 50%[d]	NMR - 60%	NMR - 60 %

All reaction were first performed on a 0.1 mmol scale. Using the final selected conditions, the reaction was repeated on 0.25 mmol scale and isolated. [a] 2.0 eq. Ar-I **2** was used. [b] HFIP (0.4M) as sole solvent. [c] 20 mol% NBE **N9** instead of NBE **N7**, 48h. [d] 3.0 eq. anisole **5f**, 1.0 eq. Ar-I **2**, 40 h.

Methyl 3'-methoxy-4'-methyl-[1,1'-biphenyl]-4-carboxylate (6a)

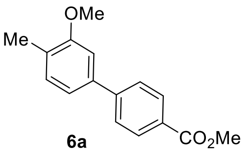

6a

General procedure B-N7 was followed using 2-methylanisole (31.4 µL, 0.25 mmol, 1.0 eq.) as substrate and methyl 4-iodobenzoate (100.3 mg, 0.375 mmol, 1.5 eq.), providing the arylated product in 80% ^{1}H NMR yield (*m:p* 11:1). Purification by column chromatography on silica gel using Cy / DCM (9:1-7:3 v/v) as an eluent provided the title compound as a white solid (51.2 mg, 80% yield (*m:p* 13:1). **R_f** = 0.37 (Cy / DCM = 1:1). General procedure B-N9 provided the title compound as a white solid (58.1 mg, 92% yield (*m:p* 12:1). **^{1}H NMR** (300 MHz, $CDCl_3$) δ 8.10 (d, *J* = 8.5 Hz, 2H), 7.65 (d, *J* = 8.5 Hz, 2H), 7.22 (d, *J* = 7.6 Hz, 1H), 7.13 (dd, *J* = 7.6, 1.5 Hz, 1H), 7.06 (d, *J* = 1.5 Hz, 1H), 3.95 (s, 3H), 3.91 (s, 3H), 2.28 (s, 3H). ^{1}H NMR of the isolated material matched with that reported in the literature.[25]

Methyl 4'-isopropyl-3'-methoxy-[1,1'-biphenyl]-4-carboxylate (6c)

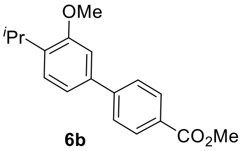

6b

General procedure B-N7 was followed using 2-*iso*-propylanisole (40.1 µL, 0.25 mmol, 1.0 eq.) as substrate and methyl 4-iodobenzoate (100.3 mg, 0.375 mmol, 1.5 eq.), providing the arylated product in 67% ^{1}H NMR yield (*m:p* 15:1). Purification by column chromatography on silica gel using Cy / DCM (9:1-4:1 v/v) as an eluent provided the title compound as a clear oil (45.2 mg, 64% yield (*m:p* 15:1)). General procedure B-N9 provided the title compound as a white solid (62.5 mg, 88% yield (*m:p* 12:1). **R_f** = 0.30 (Cy / DCM = 1:1). **^{1}H NMR** (400 MHz, $CDCl_3$) δ 8.11 (d, *J* = 8.4 Hz, 2H), 7.66 (d, *J* = 8.5 Hz, 2H), 7.31 (d, *J* = 7.9 Hz, 1H), 7.20 (dd, *J* = 7.9, 1.6 Hz, 1H), 7.09 (d, *J* = 1.6 Hz, 1H), 3.95 (s, 3H), 3.92 (s, 3H), 3.38 (hept, *J* = 6.9 Hz, 1H), 1.27 (s, 3H), 1.26 (s, 3H). ^{1}H NMR of the isolated material matched with that reported in the literature.[25]

Methyl 4'-(*tert*-butyl)-3'-methoxy-[1,1'-biphenyl]-4-carboxylate (6c)

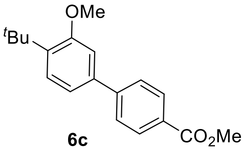

6c

General procedure B-N7 was followed using 2-*tert*-butylproylanisole (41.0 mg, 0.25 mmol, 1.0 eq.) as substrate and methyl 4-iodobenzoate (100.3 mg, 0.375 mmol, 1.5 eq.), providing the arylated product in 70% ^{1}H NMR yield. Purification by column chromatography on silica gel using Cy / DCM (9:1-7:3 v/v) as an eluent provided the title compound as a white solid (53.0 mg, 71% yield). **R_f** = 0.43 (Cy / DCM = 1:1). General procedure B-N9 provided the title compound as a white solid (65.3 mg, 88% yield). **^{1}H NMR** (400 MHz, $CDCl_3$) δ 8.10 (d, *J* = 8.5 Hz, 2H), 7.66 (d, *J* = 8.5 Hz, 2H), 7.37 (d, *J* = 8.0 Hz, 1H), 7.16 (dd, *J* = 8.0, 1.8 Hz, 1H), 7.12 (d, *J* = 1.8 Hz, 1H), 3.95 (s, 3H), 3.93 (s, 3H), 1.42 (s, 9H). **^{13}C NMR** (101 MHz, $CDCl_3$) δ 167.12, 159.02, 145.67, 139.01, 138.57, 130.16, 128.85, 127.24, 126.99, 119.28, 110.49, 55.22, 52.20, 34.90, 29.82. **HRMS** (FI): *m/z* calculated for $C_{19}H_{22}O_3$ $[M]^+$= 298.1569; found= 298.1564. **IR** (neat): v_{max} (cm^{-1}): 2951, 1714, 1606, 1554, 1490, 1452, 1436, 1418, 1395, 1307, 1270, 1220, 1182, 1151, 1098, 1049, 1030, 1015, 967, 908, 883, 858, 847, 822, 771, 727, 703, 529, 512. **M.P.** = 108−111 °C.

Methyl 3',4'-dimethoxy-[1,1'-biphenyl]-4-carboxylate (6d)

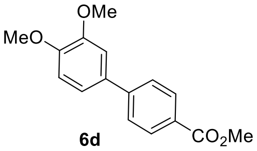

6d

General procedure B-N7 was followed using 1,2-dimethoxybenzene (31.8 µL, 0.25 mmol, 1.0 eq.) as substrate and methyl 4-iodobenzoate (100.3 mg, 0.375 mmol, 1.5 eq.), providing the arylated product in 68% ^{1}H NMR yield. Purification by column chromatography on silica gel using Cy / DCM (4:1-3:7 v/v) as an eluent provided the title compound as a white solid (45.9 mg, 68% yield). **R_f** = 0.21 (Cy / EtOAc = 4:1). **^{1}H NMR** (300 MHz, $CDCl_3$) δ 8.07 (d, *J* = 8.4 Hz, 2H), 7.61 (d, *J* = 8.4 Hz, 2H), 7.18 (dd, *J* = 8.3, 2.0 Hz, 1H), 7.13 (d, *J* = 2.0 Hz, 1H), 6.95 (d, *J* = 8.3 Hz, 1H), 3.95 (s, 3H), 3.93 (s, 6H). **^{13}C NMR** (101 MHz, $CDCl_3$) δ 167.09, 149.46, 149.40, 145.49, 132.93, 130.18, 128.50, 126.69, 119.87, 111.62, 110.49, 56.10, 56.09, 52.17. **HRMS** (FI): *m/z* calculated for $C_{16}H_{16}O_4$ $[M]^+$= 272.1049; found= 272.1039. **IR** (neat): v_{max} (cm^{-1}): 2949, 2250, 1713, 1599, 1527, 1500, 1431, 1400, 1319, 1270, 1218, 1170, 1147, 1106, 1023, 912, 874, 855, 815, 766, 727, 698, 646, 495. **M.P.** = 114−115 °C.

Methyl 4'-chloro-3'-methoxy-[1,1'-biphenyl]-4-carboxylate (6e)

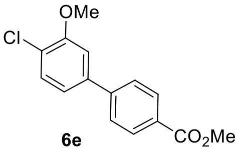

6e

General procedure B-N9 was followed using 2-chloroanisole (31.8 µL, 0.25 mmol, 1.0 eq.) as substrate and methyl 4-iodobenzoate (100.3 mg, 0.375 mmol, 1.5 eq.) in HFIP (0.4 M), using **N9** (11.8 mg, 0.05 mmol, 0.2 eq.) and stirring the reaction for 48 h, providing the arylated product in 52% ^{1}H NMR yield. Purification by column chromatography on silica gel using Cy / DCM (9:1-7:3 v/v) as an eluent provided the title compound as a white solid (34.9 mg, 50% yield (*m:p* 9:1). **R_f** = 0.23 (Cy / DCM = 1:1. **^{1}H NMR** (400 MHz, $CDCl_3$) δ 8.11 (d, *J* = 8.4 Hz, 2H), 7.62 (d, *J* = 8.4 Hz, 2H), 7.44 (d, *J* = 8.7 Hz, 1H), 7.17 – 7.12 (m, 2H), 3.98 (s, 3H), 3.95 (s, 3H). **^{13}C NMR** (101 MHz, $CDCl_3$) δ 166.97, 155.42, 144.86, 140.18, 130.69, 130.31, 129.43, 127.14, 122.83, 120.34, 111.12, 56.37, 52.34. **HRMS** (FI): *m/z* calculated for $C_{15}H_{13}ClO_3$ $[M]^+$= 276.0553; found= 276.0561. **IR** (neat): v_{max} (cm^{-1}): 2949, 1719, 1606, 1593, 1582, 1487, 1435, 1391, 1375, 1307, 1285, 1270, 1230, 1187, 1105, 1067, 1037, 1025, 855, 818, 768. **M.P.** = 118−120 °C.

Methyl 3'-methoxy-4'-(trifluoromethyl)-[1,1'-biphenyl]-4-carboxylate (6f)

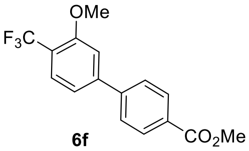
6f

General procedure **B-N7** was followed using 2-trifluoromethylanisole (112.5 µL, 0.75 mmol, 3.0 eq.) as substrate and methyl 4-iodobenzoate (65.5 mg, 0.25 mmol, 1.0 eq.) in HFIP (0.4 M), and a reaction time of 40 h, providing the arylated product in 58% ^{1}H NMR yield. Purification by column chromatography on silica gel using Cy / DCM (9:1-4:1 v/v) as an eluent provided the title compound as a white solid (43.7 mg, 56 % yield). **R$_f$** = 0.35 (Cy / EtOAc = 4:1). **^{1}H NMR** (400 MHz, $CDCl_3$) δ 8.13 (d, *J* = 8.1 Hz, 2H), 7.69 – 7.60 (m, 3H), 7.23 (d, *J* = 8.1 Hz, 1H), 7.19 (s, 1H), 3.99 (s, 3H), 3.96 (s, 3H). **^{19}F NMR** (376 MHz, $CDCl_3$) δ -62.30. **^{13}C NMR** (101 MHz, $CDCl_3$) δ 166.88, 158.04 – 157.94 (m), 145.52, 144.51, 130.36, 130.02, 127.80 (q, $J_{C\text{-}F}$ = 5.2 Hz), 127.42, 127.78 – 119.56 (m), 119.14, 118.50 (q, $J_{C\text{-}F}$ = 31.2 Hz), 111.03, 56.18, 52.41. **HRMS** (FI): *m/z* calculated for $C_{16}H_{13}F_3O_3$ $[M]^+$= 310.0817; found= 310.0805. **IR** (neat): ν_{max} (cm^{-1}): 2919, 2849, 1716, 1679, 1602, 1592, 1562, 1435, 1422, 1393, 1317, 1304, 1270, 1229, 1209, 1190, 1167, 1130, 1113, 1104, 1063, 1025, 1014, 955, 877, 854, 823, 771, 750, 731, 724, 703, 659. **M.P.** = 152−154 °C.

Methyl 4-(2,3-dihydrobenzofuran-6-yl)benzoate (6h)

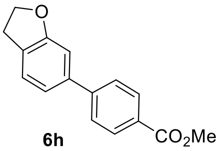
6h

General procedure **B-N7** was followed using 2,3-dihydrobenzofuran (28.2 µL, 0.25 mmol, 1.0 eq.) as substrate and methyl 4-iodobenzoate (100.3 mg, 0.375 mmol, 1.5 eq.), providing the arylated product in 65% ^{1}H NMR yield. Purification by column chromatography on silica gel using Cy / DCM (4:1-3:2 v/v) as an eluent provided the title compound as a white solid (38.5 mg, 61% yield). **R$_f$** = 0.22 (Cy / DCM = 1:1). **^{1}H NMR** (400 MHz, $CDCl_3$) δ 8.08 (d, *J* = 8.3 Hz, 2H), 7.62 (d, *J* = 8.3 Hz, 2H), 7.30 – 7.26 (m, 1H), 7.11 (dd, *J* = 7.6, 1.3 Hz, 1H), 7.04 (s, 1H), 4.63 (t, *J* = 8.7 Hz, 2H), 3.94 (s, 3H), 3.26 (t, *J* = 8.7 Hz, 3H). ^{1}H NMR of the isolated material matched with that reported in the literature.[25]

Methyl 4-(2-methyl-2,3-dihydrobenzofuran-6-yl)benzoate (6i)

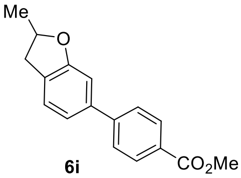
6i

General procedure **B-N7** was followed using 2-methyl-2,3-dihydrobenzofuran (33.5 mL, 0.25 mmol, 1.0 eq.) as substrate and methyl 4-iodobenzoate (100.3 mg, 0.375 mmol, 1.5 eq.), providing the arylated product in 60% ^{1}H NMR yield. Purification by column chromatography on silica gel using Cy / DCM (4:1-3:2 v/v) as an eluent provided the title compound as a white solid (39.3 mg, 59% yield). **R$_f$** = 0.22 (Cy / DCM = 1:1). **^{1}H NMR** (400 MHz, $CDCl_3$) δ 8.08 (d, *J* = 8.5 Hz, 2H), 7.62 (d, *J* = 8.5 Hz, 2H), 7.22 (d, *J* = 7.6 Hz, 1H), 7.09 (dd, *J* = 7.6, 1.4 Hz, 1H), 7.01 (d, *J* = 1.4 Hz, 1H), 4.99 (ddq, *J* = 8.8, 7.6, 6.2 Hz, 1H), 3.93 (s, 3H), 3.36 (dd, *J* = 15.7, 8.8 Hz, 1H), 2.85 (dd, *J* = 15.8, 7.4 Hz, 1H), 1.50 (d, *J* = 6.3 Hz, 3H). ^{1}H NMR of the isolated material matched with that reported in the literature.[25]

9. Optimization of the *meta*-Arylation of Anisole

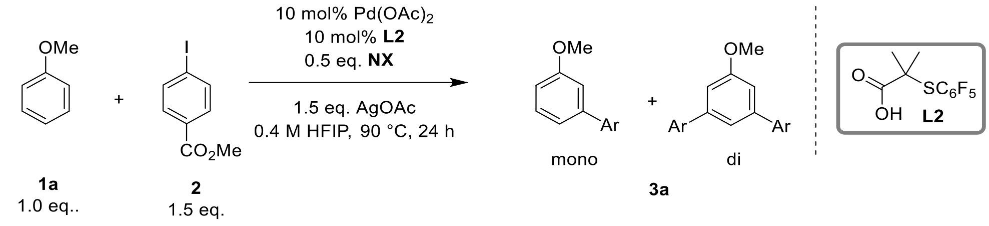

Table S13. Optimization of meta-monoarylation of anisole.

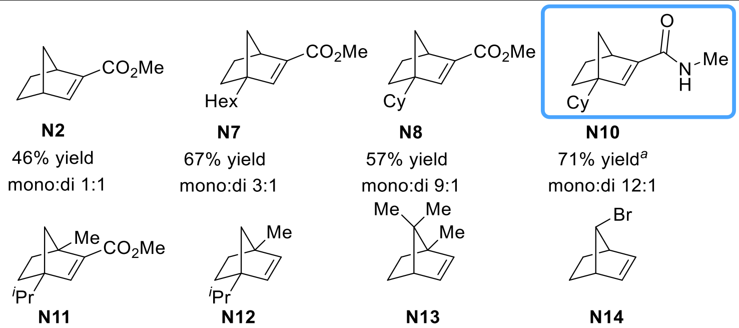

N2	N7	N8	N10
46% yield	67% yield	57% yield	71% yield[a]
mono:di 1:1	mono:di 3:1	mono:di 9:1	mono:di 12:1

N11	N12	N13	N14
8% yield	traces	traces	traces
n.d.	n.d.	n.d.	n.d.

#	**N10** [eq.]	Anisole **1a** [eq.]	Ar-I **2a** [eq.]	NMR yield [%] **3a**	ratio mono : di
1	0.5	1.5	1	56	13 : 1
2	0.3	1.5	1	57	19 : 1
3	0.2	1.5	1	64	19 : 1
4	0.2	1	1.5	71	12 : 1

10. General Procedure C for the *meta*-C–H Arylation of Unsubstituted Aryl Ethers

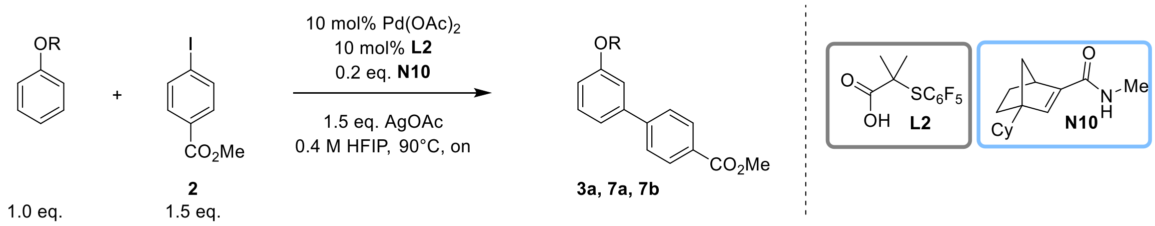

In a pressure tube containing a suitable stirring bar **N10** (11.7 mg, 0.05 mmol, 0.2 eq.), $Pd(OAc)_2$ (5.6 mg, 0.025 mmol, 10 mol%), silver acetate (62.6 mg, 0.375 mmol, 1.5 eq.), methyl 4-iodobenzoate (65.5 mg, 0.25 mmol, 1.0 eq.), anisole (0.375 mmol, 1.5 eq.), a stock solution of S,O-ligand **L2** in HFIP (250 µL, 0.1 M, 0.025mmol, 10 mol%) and HFIP (0.375 mL, 0.4 M HFIP) was added. The tube was put into a pre-heated oil bath at 90 °C and was stirred for 24 h. After cooling to room temperature, the reaction was filtered through Celite® and rinsed with EtOAc. The solvent was evaporated under reduced pressure. To the crude mixture CH_2Br_2 (17.7 µL, 0.25 mmol) was added as internal standard, the mixture was dissolved in $CDCl_3$ and ^1H NMR was measured. Subsequentially, the product was purified by flash column chromatography.

Methyl 3'-methoxy-[1,1'-biphenyl]-4-carboxylate (3a)

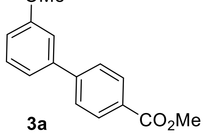

3a

General procedure **C** was followed using anisole (27.2 µL, 0.25 mmol, 1.0 eq.) as substrate and methyl 4-iodobenzoate (100.3 mg, 0.375 mmol, 1.5 eq.), providing the arylated product in 58% (mono:di 9:1) ^1H NMR yield. Purification by column chromatography on silica gel using Cy:DCM (4:1-3:7 v/v) as an eluent provided the title compound as a white solid (32.5 mg, 54% yield). **R_f** = 0.59 (Cy / EtOAc = 7:3). **^1H NMR** (400 MHz, $CDCl_3$) δ 8.10 (d, *J* = 8.6 Hz, 2H), 7.65 (d, *J* = 8.6 Hz, 2H), 7.38 (t, *J* = 7.8 Hz, 1H), 7.21 (ddd, *J* = 7.8, 1.6, 0.9 Hz, 1H), 7.17 – 7.13 (m, 1H), 6.94 (ddd, *J* = 8.2, 2.6, 0.9 Hz, 1H), 3.94 (s, 3H), 3.88 (s, 3H). **^{13}C NMR** (101 MHz, $CDCl_3$) δ 167.12, 160.17, 145.65, 141.66, 130.21, 130.09, 129.17, 127.25, 119.91, 113.65, 113.18, 55.51, 52.29. **IR** (neat): ν_{max} (cm^{-1}): 2999, 2951, 2079, 1937, 1719, 1606, 1565, 1399, 1278, 1214, 1182, 1108, 1053, 1030, 1016, 995, 967, 850, 725, 696, 637, 612, 568, 494. ^1H NMR of the isolated material matched with that reported in the literature.[29]

Methyl 3'-butoxy-[1,1'-biphenyl]-4-carboxylate (7a)

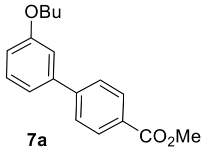

General procedure **C** was followed using butyl phenyl ether (40.2 µL, 0.25 mmol, 1.0 eq.) as substrate and methyl 4-iodobenzoate (100.3 mg, 0.375 mmol, 1.5 eq.), providing the arylated product in 58% (mono:di 9:1) ^{1}H NMR yield. Purification by column chromatography on silica gel using Cy / DCM (9:1-7:3 v/v) as an eluent provided the title compound as a clear oil (39.7 mg, 56% yield). **R$_f$** = 0.14 (Cy / DCM = 1:1). **^{1}H NMR** (400 MHz, $CDCl_3$)) δ 8.10 (d, *J* = 8.4 Hz, 2H), 7.66 (d, *J* = 8.4 Hz, 2H), 7.36 (t, *J* = 7.9 Hz, 1H), 7.19 (d, *J* = 8.0 Hz, 1H), 7.16 – 7.14 (m, 1H), 6.93 (ddd, *J* = 8.2, 2.6, 1.0 Hz, 1H), 4.03 (t, *J* = 6.5 Hz, 2H), 3.94 (s, 3H), 1.80 (dt, *J* = 14.5, 6.5 Hz, 2H), 1.52 (h, *J* = 7.4 Hz, 2H), 1.00 (t, *J* = 7.3 Hz, 3H). **^{13}C NMR** (101 MHz, $CDCl_3$) δ 167.12, 159.74, 145.70, 141.56, 130.17, 130.02, 129.10, 127.21, 119.67, 114.16, 113.80, 67.93, 52.25, 31.50, 19.42, 14.01. **HRMS (FD)**: *m/z* calculated for $C_{18}H_{20}O_3$ $[M]^+$= 284.1412; found= 284.1405. **IR** (neat): v_{max} (cm^{-1}): 2955, 2872, 1719, 1605, 1583, 1564, 1473, 1434, 1400, 1273, 1204, 1103, 1069, 1050, 1015, 971, 852, 822, 790, 765, 694.

Methyl 3'-(benzyloxy)-[1,1'-biphenyl]-4-carboxylate (7b)

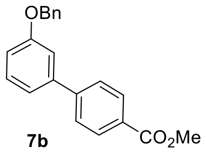

General procedure **C** was followed using benzyl phenyl ether (46.1 µL, 0.25 mmol, 1.0 eq.) as substrate and methyl 4-iodobenzoate (100.3 mg, 0.375 mmol, 1.5 eq.), providing the arylated product in 47% ^{1}H NMR yield. Purification by column chromatography on silica gel using Cy / DCM (9:1-7:3 v/v) as an eluent provided the title compound as a white solid (33.4 mg, 42% yield). **R$_f$** = 0.13 (Cy / DCM = 1:1). **^{1}H NMR** (400 MHz, $CDCl_3$) δ 7.96 (d, *J* = 8.3 Hz, 2H), 7.50 (d, *J* = 8.3 Hz, 2H), 7.33 (d, *J* = 7.1 Hz, 2H), 7.26 (t, *J* = 7.4 Hz, 2H), 7.24 – 7.17 (m, 2H), 7.12 – 7.06 (m, 2H), 6.87 (dd, *J* = 8.2, 2.3 Hz, 1H), 4.99 (s, 2H), 3.80 (s, 3H). ^{13}C NMR (101 MHz, $CDCl_3$) δ 167.08, 159.36, 145.53, 141.63, 136.95, 130.20, 130.10, 129.16, 128.76, 128.19, 127.65, 127.20, 120.13, 114.46, 114.21, 70.27, 52.26. **HRMS** (FD): *m/z* calculated for $C_{21}H_{18}O_3$ $[M]^+$= 318.1256; found= 318.1261. **IR** (neat): v_{max} (cm^{-1}): 3033, 2950, 1720, 1606, 1584, 1565, 1480, 1454, 1435, 1400, 1380, 1278, 1202, 1111, 1050, 1013, 967, 878, 852, 822, 790, 767, 733, 695. **M.P.** = 102−105 °C.

10.1. General Procedure D for Unsymmetrical Diarylation of Anisole

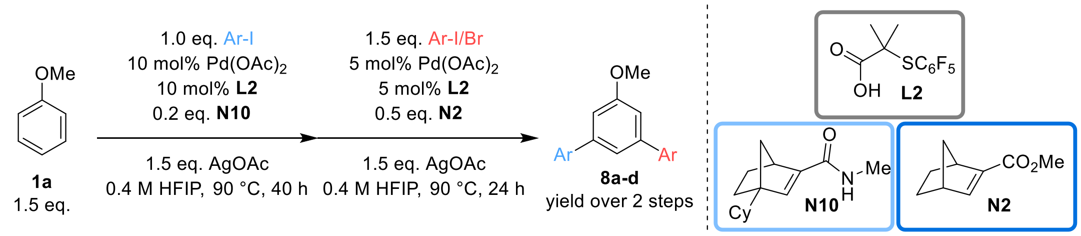

In a pressure tube containing a suitable stirring bar **N10** (11.7, 0.125 mmol, 0.5 eq.), $Pd(OAc)_2$ (5.6 mg, 0.025 mmol, 10 mol%), silver acetate (62.6 mg, 0.375 mmol, 1.5 eq.), methyl 4-iodobenzoate (100.3 mg, 0.375 mmol, 1.5 eq.), anisole derivative (0.25 mmol, 1.0 eq.), S,O-ligand **L2** stock solution in HFIP (250 µL, 0.1 M, 0.025mmol, 10 mol%) and HFIP (0.375 mL, 0.4 M) was added. The tube was put into a pre-heated oil bath at 90 °C and was stirred for 40 h. After cooling to room temperature, the reaction was filtered through Celite® and rinsed with EtOAc. The solvent was evaporated under reduced pressure. To the crude mixture CH_2Br_2 (17.7 µL, 0.25 mmol) was added as internal standard, the mixture was dissolved in $CDCl_3$ and ^{1}H NMR was measured. Subsequentially, the crude mixture was added to another pressure tube containing a suitable stirring bar and **N2** (19.0 mg, 0.125 mmol, 0.5 eq.), $Pd(OAc)_2$ (2.8 mg, 0.0125 mmol, 5 mol%), silver acetate (62.6 mg, 0.375 mmol, 1.5 eq.), aryl halide (0.375 mmol, 1.5 eq.),a stock solution of S,O-ligand **L2** in HFIP (125 µL, 0.1 M, 0.0125mmol, 5 mol%) and HFIP (0.5 mL, 0.4 M) was added. The tube was put into a pre-heated oil bath at 90 °C and was stirred for 24 h. After cooling to room temperature, the reaction was filtered through Celite® and rinsed with EtOAc. The solvent was evaporated under reduced pressure. To the crude mixture CH_2Br_2 (17.7 µL, 0.25 mmol) was added as internal standard, the mixture was dissolved in $CDCl_3$ and ^{1}H NMR was measured [Note: The second ^{1}H NMR yield is calculated from the initial amount of anisole]. Subsequentially, the product was purified by flash column chromatography. The isolated yield reported is over two steps.

Methyl 4''-acetyl-5'-methoxy-[1,1':3',1''-terphenyl]-4-carboxylate (8a)

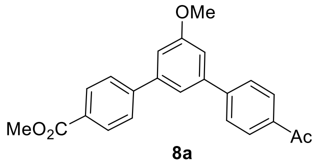

General procedure **D** was followed using anisole (40.8 µL, 0.375 mmol, 1.5 eq.) and methyl 4-iodobenzoate (65.5 mg, 0.25 mmol, 1.0 eq, providing the mono arylated product in 59% (mono:di 19:1) ^{1}H NMR yield. For the second arylation 4'-Iodoacetophenone (92.3 mg, 0.375 mmol, 1.5 eq.) was used, providing the diarylated product **8a** in 51% ^{1}H NMR yield. Purification by column chromatography on silica gel using Cy / EtOAc (20:1-10:1 v/v) as an eluent provided the title compound as a pale yellow solid (39.7 mg, 44% yield). **R$_f$** = 0.20 (Cy / EtOAc = 4:1). **^{1}H NMR** (300 MHz, $CDCl_3$) δ 8.12 (d, *J* = 8.4 Hz, 2H), 8.05 (d, *J* = 8.4 Hz, 2H), 7.76 – 7.67 (m, 4H), 7.43 (t, *J* = 1.6 Hz, 1H), 7.21 – 7.14 (m, 2H), 3.95 (s, 3H), 3.94 (s, 3H), 2.65 (s, 3H). **^{13}C NMR** (101 MHz, $CDCl_3$) δ 197.81, 167.01, 160.62, 145.50, 145.34, 142.32, 142.15, 136.33, 130.28, 129.45, 129.07, 127.52, 127.33, 119.10, 112.81, 55.71, 52.32, 26.82. **HRMS** (FI): *m/z* calculated for $C_{23}H_{20}O_4$ $[M]^+$= 360.1362; found= 360.1362. **IR** (neat): v_{max} (cm^{-1}): 2951, 2841, 1716, 1678, 1591, 1566, 1511, 1489, 1453, 1433, 1391, 1345, 1268, 1207, 1178, 1104, 1071, 1029, 1015, 995, 958, 909, 890, 878, 828, 772, 730, 703, 678, 647, 613, 597, 534, 488. **M.P.** = 114−116 °C.

Methyl 5'-methoxy-2''-nitro-[1,1':3',1''-terphenyl]-4-carboxylate (8b)

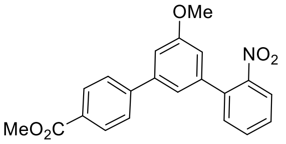

8b

General procedure **D** was followed using anisole (40.8 µL, 0.375 mmol, 1.5 eq.) and methyl 4-iodobenzoate (65.5 mg, 0.25 mmol, 1.0 eq.), providing the mono arylated product in 55% (mono:di 18:1) ^{1}H NMR yield. For the second arylation 1-bromo-2-nitrobenzene (78.9 mg, 0.375 mmol, 1.5 eq.) was used, providing the diarylated product **8b**. Purification by column chromatography on silica gel using Cy / EtOAc (30:1-20:1 v/v) as an eluent provided the title compound as a pale brown solid (39.2 mg, 43% yield). **R$_f$** = 0.08 (Cy / EtOAc = 9:1). **^{1}H NMR** (400 MHz, $CDCl_3$) δ 8.10 (d, *J* = 8.2 Hz, 2H), 7.88 (d, *J* = 7.9 Hz, 1H), 7.68 – 7.60 (m, 3H), 7.56 – 7.48 (m, 2H), 7.17 (s, 1H), 7.14 (s, 1H), 6.89 (s, 1H), 3.94 (s, 3H), 3.89 (s, 3H). **^{13}C NMR** (101 MHz, $CDCl_3$) δ 167.04, 160.26, 149.42, 145.04, 142.01, 139.45, 136.06, 132.48, 132.00, 130.24, 129.45, 128.63, 127.36, 124.24, 119.60, 113.36, 113.03, 55.67, 52.31. **HRMS** (FI): *m/z* calculated for $C_{21}H_{17}NO_5$ $[M]^+$= 363.1107; found= 363.1103. **IR** (neat): v_{max} (cm^{-1}): 2952, 1720, 1593, 1571, 1527, 1486, 1455, 1435, 1396, 1348, 1280, 1209, 1181, 1107, 1069, 1025, 1016, 850, 810, 773, 752, 733, 699. **M.P.** = 160−162 °C.

Methyl 3''-chloro-5'-methoxy-[1,1':3',1''-terphenyl]-2-carboxylate (8c)

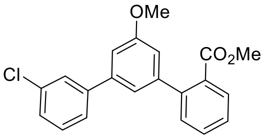

8c

General procedure **D** was followed using anisole (40.8 µL, 0.375 mmol, 1.5 eq.) and 3-chloroiodobenzene (31 µL, 0.25 mmol, 1.0 eq.), providing the mono arylated product in 53% (mono:di >20:1) ^{1}H NMR yield. For the second arylation methyl 2-bromobenzoate (53.2 mg, 0.375 mmol, 1.5 eq.) was used, providing the diarylated product **8c**. Purification by column chromatography on silica gel using Cy / EtOAc (50:1 v/v) as an eluent provided the title compound as a beige oil (39.2 mg, 43% yield). **R$_f$** = 0.43 (Cy / EtOAc = 4:1). **^{1}H NMR** (400 MHz, $CDCl_3$) δ 7.84 (d, *J* = 7.2 Hz, 1H), 7.59 (d, *J* = 1.9 Hz, 1H), 7.55 (td, *J* = 7.3, 1.4 Hz, 1H), 7.48 (dt, *J* = 7.4, 1.6 Hz, 1H), 7.46 – 7.41 (m, 2H), 7.39 – 7.30 (m, 2H), 7.11 – 7.07 (m, 2H), 6.88 (dd, *J* = 2.4, 1.4 Hz, 1H), 3.88 (s, 3H), 3.67 (s, 3H). **^{13}C NMR** (101 MHz, $CDCl_3$) δ 169.16, 159.88, 143.38, 142.84, 142.10, 141.02, 134.76, 131.44, 131.11, 130.73, 130.11, 129.89, 127.64, 127.62, 127.49, 125.49, 120.03, 113.51, 111.92, 55.60, 52.24. **HRMS** (FD): *m/z* calculated for $C_{21}H_{17}ClO_3$ $[M]^+$= 352.0866; found= 352.0856. **IR** (neat): v_{max} (cm^{-1}): 2998, 2949, 2838, 1726, 1591, 1566, 1483, 1456, 1432, 1395, 1343, 1292, 1256, 1207, 1177, 1126, 1100, 1070, 1046, 1027, 965, 901, 856, 818, 787, 763, 721, 695, 677.

Methyl 4''-bromo-5'-methoxy-[1,1':3',1''-terphenyl]-4-carboxylate (8d)

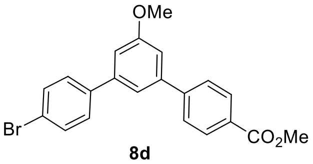

8d

General procedure **D** was followed using anisole (40.8 µL, 0.375 mmol, 1.5 eq.) and methyl 4-iodobenzoate (70.7mg, 0.25 mmol, 1.0 eq.), providing the mono arylated product in 56% (mono:di >20:1) ^{1}H NMR yield. For the second arylation methyl 4-iodobenzoate (100.3 mg, 0.375 mmol, 1.5 eq.) was used, providing the diarylated product **8d**. Purification by column chromatography on silica gel using Cy / EtOAc (50:1 v/v) as an eluent provided the title compoundas a white solid (35.5 mg, 36% yield). **R$_f$** = 0.33 (Cy / EtOAc = 4:1). **^{1}H NMR** (400 MHz, $CDCl_3$) δ 8.12 (d, *J* = 8.4 Hz, 2H), 7.69 (d, *J* = 8.4 Hz, 2H), 7.59 (d, *J* = 8.5 Hz, 2H), 7.50 (d, *J* = 8.5 Hz, 2H), 7.36 (t, *J* = 1.5 Hz, 1H), 7.14 (t, *J* = 1.9 Hz, 1H), 7.10 (t, *J* = 2.0 Hz, 1H), 3.95 (s, 3H), 3.93 (s, 3H). **^{13}C NMR** (101 MHz, $CDCl_3$) δ 167.05, 160.63, 145.46, 142.30, 142.29, 139.90, 132.08, 130.28, 129.42, 128.99, 127.35, 122.12, 118.84, 112.54, 112.28, 55.70, 52.33. **HRMS** (FD): *m/z* calculated for $C_{21}H_{17}BrO_3$ $[M]^+$= 396.0361; found= 396.0373. **IR** (neat): v_{max} (cm^{-1}): 3206, 2951, 2929, 1720, 1593, 1566, 1492, 1453, 1434, 1401, 1385, 1346, 1279, 1208, 1192, 1107, 1072, 1030, 1017, 1009, 822, 773. **M.P.** = 133−135 °C.

11. Mechanistical Studies

11.1. Reversibility of initial C−H Activation

In a pressure tube containing a suitable stirring bar, $Pd(OAc)_2$ (1.1 mg, 0.01 mmol, 5 mol%), **L2** (1.5 mg, 0.01 mmol, 5 mol%) and anisole (11 µl, 0.1 mmol, 1.0 eq), HFIP-d_2 (0.25 mL, 0.4 M) was added. The tube was put into a pre-heated oil bath at 90 °C and was stirred overnight (~18 h). After cooling to room temperature, the reaction was filtrated through Celite® and rinsed with DCM. The solvent was removed under reduced pressure and to the crude mixture was dissolved in $CDCl_3$ and ^1H NMR was measured.

Figure S12 Reversibility of first C−H activation in deuterated HFIP-d_2

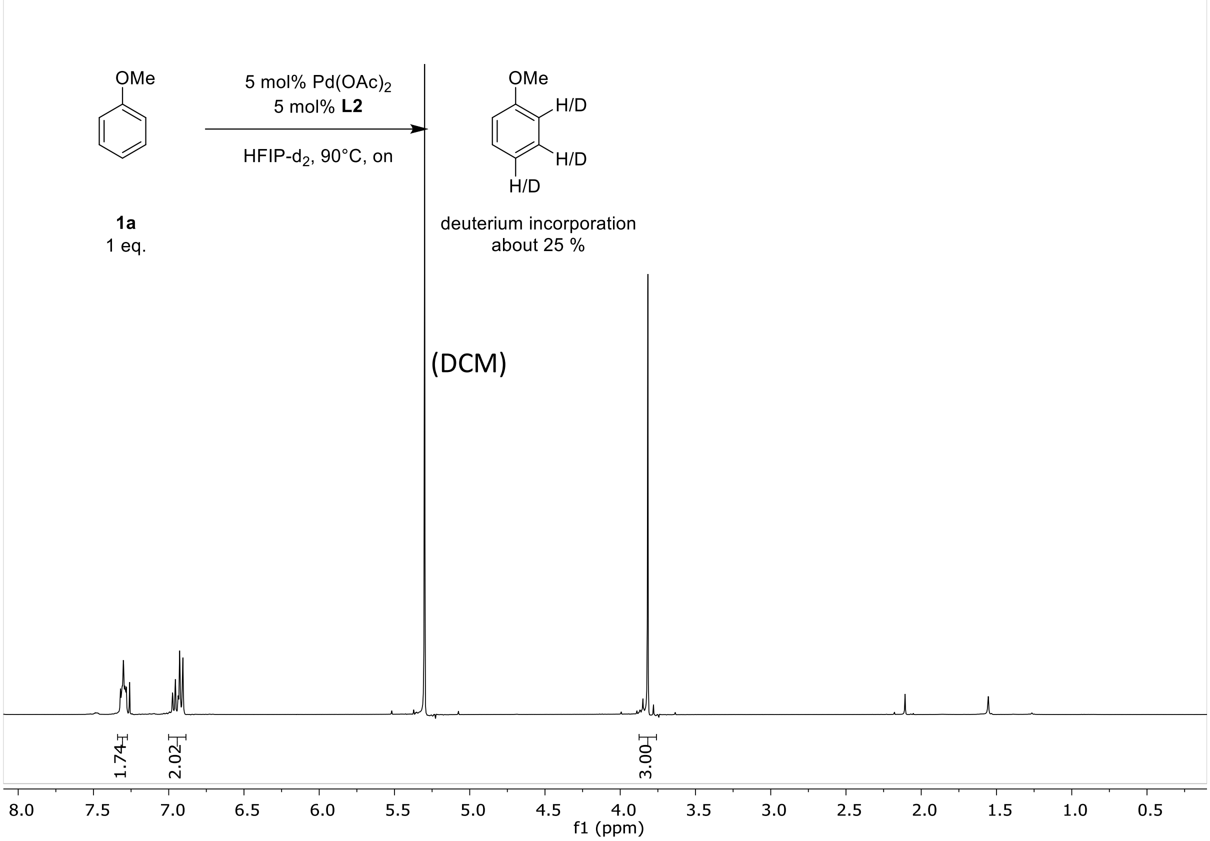

11.2. Synthesis of Complex C1

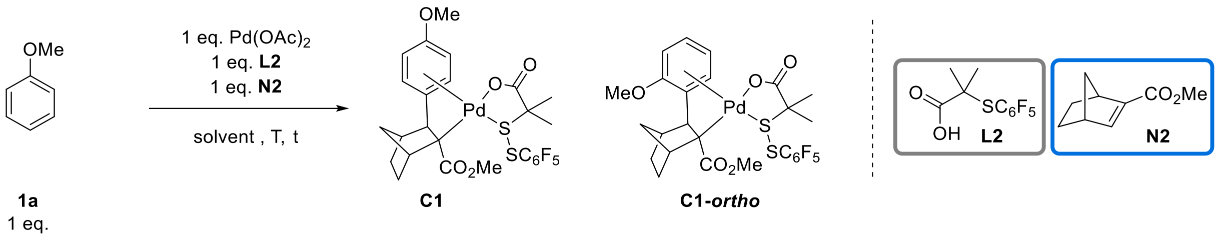

The optimization for the synthesis of complex **C1** was done by adding anisole (11 µL, 0.1 mmol, 1.0 eq), $Pd(OAc)_2$ (22.8 mg, 0.1 mmol, 1.0 eq), **N2** (15.2 mg, 0.1 mmol, 1.0 eq), **L2** (28.6 mg, 0.1 mmol, 1.0 eq) and solvent (0.25 mL, 0.4 M) in a pressure tube and the mixture was stirred at the indicated temperature and for the indicated time. After cooling to room temperature, the reaction was filtrated through Celite® and rinsed with DCM. The solvent was removed under reduced pressure and to the crude mixture CH_2Br_2 (17.7 µL, 0.25 mmol) was added as internal standard, the mixture was dissolved in $CDCl_3$ and ^1H NMR was measured.

Table S14. Conditions to obtain complex **C1.**

[a] Complex **C1**-*ortho* could not be isolated, however the indicative peaks in crude NMR are shown in Figure 12.

#	Solvent 0.2 M	Temperature [°C]	Time [h]	^{1}H-NMR yield [%] **C1**	^{1}H-NMR yield [%] **C1-*ortho***[a]
1	DCE	60	18	50	-
2	DCE	60	2	18	-
3	DCE	rt	2	-	-
4	HFIP	90	2	70	-
5	HFIP	60	2	39	60
6	HFIP	rt	2	13	22

Complex C1

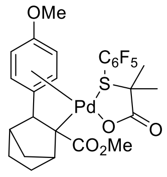

C1

Complex **C1** was synthesized by adding anisole (165 µL, 1.5 mmol, 1.0 eq), $Pd(OAc)_2$ (342 mg, 1.5 mmol, 1.0 eq), **N2** (228 mg, 1.5 mmol, 1.0 eq.), **L2** (429 mg, 1.5 mmol, 1.0 eq)and HFIP (3.75 mL, 0.4 M) in a pressure tube and the mixture was stirred for 2 h at 90 °C. After cooling to room temperature, the reaction was filtrated through Celite® and rinsed with DCM. Purification by column chromatography on silica gel using EtOAc / MeOH(1:0-9:1 v/v) as an eluent provided the title compound as a orange solid (71% yield, 692 mg, 1.07 mmol). $\mathbf{R_f}$ = 0.74 (EtOAc / MeOH = 50:1). **^{1}H NMR** (400 MHz, $CDCl_3$) δ 8.16 (d, *J* = 7.8 Hz, 1H), 7.83 (d, *J* = 8.6 Hz, 1H), 7.19 (d, *J* = 8.1 Hz, 1H), 6.99 (d, *J* = 7.0 Hz, 1H), 3.85 (s, 3H), 3.56 (s, 1H), 3.38 (s, 3H), 3.04 (d, *J* = 10.1 Hz, 1H), 2.64 (d, *J* = 3.6 Hz, 1H), 2.21 (s, 1H), 1.68 – 1.57 (m, 2H), 1.48 (s, 3H), 1.39 (ddd, *J* = 20.5, 10.5, 4.2 Hz, 1H), 1.27 (s, 3H), 1.24 – 1.17 (m, 1H), 1.13 – 1.04 (m, 1H). ^{13}C NMR (101 MHz, $CDCl_3$) δ 177.17, 174.58, 174.42, 164.50, 149.06 – 148.20 (m), 146.46 – 145.89 (m), 144.99 – 144.29 (m), 142.44 – 141.52 (m), 139.60 – 138.62 (m), 136.85 – 136.15 (m), 134.03, 122.42, 121.98, 113.53, 77.48, 62.52, 56.13, 52.30, 51.27, 43.11, 39.80, 39.38, 27.74, 27.39, 27.21, 26.27. **HRMS** (ESI): *m/z*: 365 (M^+ - 286(**L2**) (100), 406 (M^+ - 245), 651 (M^+ + H), calculated for $C_{26}H_{25}F_5O_5PdS$ $[M+H]^+$= 651.0460; found= 651.0479. **IR** (neat): ν_{max} (cm^{-1}): 2952, 2875, 1692, 1640, 1598, 1514, 1489, 1462, 1389, 1343, 1313, 1290, 1247, 1210, 1187, 1170, 1150, 1119, 1094, 1050, 1025, 983, 854. **M.P.** = 103−106 °C

Complex C1-*ortho*

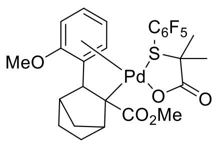

C1

Representative signals: **^{1}H NMR** (400 MHz, $CDCl_3$) δ 8.07 (d, *J* = 7.2 Hz, 1H), 7.60 (t, *J* = 7.6 Hz, 1H), 7.17 (t, *J* = 7.4 Hz, 1H), 7.09 (d, *J* = 8.3 Hz, 1H), 4.03 (s, 3), 3.20 – 2.96 (m, 1H), 2.41 – 2.20 (m, 1H), 1.60 (s, 3H), 1.34 (s, 3H). **^{13}C NMR** (101 MHz, $CDCl_3$) δ 179.71, 136.27, 129.53, 122.98, 113.98, 76.07, 60.91, 55.19, 44.50.

11.3. Synthesis of Complex C2

The optimization for the synthesis of complex **C2** was done by adding **C1** (34.9 mg, 0.05 mmol, 1.0 eq.), additive and DCE (0.25 mL, 0.4 M) in a pressure tube and the mixture was stirred at 80 °C for 2 h. After cooling to room temperature, the reaction was filtrated through Celite® and rinsed with DCM. The solvent was removed under reduced pressure and to the crude mixture CH_2Br_2 (7.08 µL, 0.1 mmol) was added as internal standard, the mixture was dissolved in $CDCl_3$ and ^1H NMR was measured.

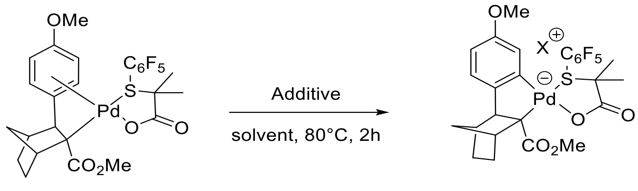

C1 **C2**

Table S15. Conditions to obtain complex **C2.**

#	Solvent 0.2 M	Additive	NMR yield [%] **C2**
1	DCE	-	-
2	DCE	3 eq. K_2CO_3 (X = K)	70
3	DCE	3 eq. KOAc (X = K)	50
4	DCE	3 eq. Na_2CO_3 (X = Na)	72
5	DCE	3 eq. Cs_2CO_3 (X = Cs)	74
6	DCE	3 eq. CsOAc (X = K)	50
7	DCE	3 eq. AgOAc (X = Ag)	-
8	DCE	3 eq. Ag_2CO_3 (X = Ag	-

Complex C2

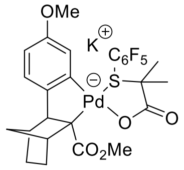

C2

Complex **C2** was synthesized by adding complex **C1** (157.4 mg, 0.23 mmol, 1.0 eq), K_2CO_3 (95.4 mg, 0.69 mmol, 3.0 eq)and DCE (1.15 mL, 0.2 M) in a pressure tube and the mixture was stirred for 2 h at 80 °C. After cooling to room temperature, the reaction was filtrated through Celite® and rinsed with DCM. Recrystallization (DCM/ Pentane) provided the title compound as a beige solid (86% yield, 128.37mg, 0.2 mmol). [Note: In acidic media complex **C2** converts back to **C1**. Therefore, **C2** cannot be purified by column chromatography or synthesized in HFIP].]. **^{1}H NMR** (400 MHz, $CDCl_3$) δ 6.85 (d, J = 8.4 Hz, 1H), 6.47 (dd, J = 8.4, 2.6 Hz, 1H), 6.31 (d, J = 2.6 Hz, 1H), 3.63 (s, 3H), 3.50 (s, 3H), 2.96 (s, 1H), 2.66 – 2.54 (m, 2H), 2.14 (d, J = 3.8 Hz, 1H), 1.60 (s, 3H), 1.57 – 1.49 (m, 1H), 1.46 (s, 3H), 1.42 – 1.34 (m, 2H), 1.24 – 1.15 (m, 2H). ^{13}C NMR (101 MHz, $CDCl_3$) δ 182.52, 179.52, 160.09, 155.40, 151.70, 149.36 – 148.56 (m), 146.84 – 146.05 (m), 144.50 – 143.68 (m), 141.95 – 141.00 (m), 139.64 – 138.87 (m), 137.10 – 136.16 (m), 123.74, 119.73, 110.33, 68.51, 58.59, 57.11, 54.78, 50.76, 46.20, 43.65, 38.74, 29.45, 27.53, 26.71, 26.13. **HRMS** (ESI): 635 (M^- - 14), 649 (M^-) (100), 973 (M^- + 324) (M^-) *m/z* calculated for $C_{26}H_{24}F_5O_5PdS$ $[M]^-$= 649.0315; found= 649.0590. **IR** (neat): v_{max} (cm^{-1}): 2947, 2869, 1598, 1578, 1512, 1486, 1433, 1389, 1350, 1294, 1277, 1241, 1217, 1188, 1163, 1145, 1119, 1092, 1044, 981, 907, 851, 809, 779, 727, 647, 633, 570, 517, 455, 439. **M.P.** = 175−180 °C.

11.4. Synthesis of Complex C3

The optimization for the synthesis of complex **C3** was done by adding **C1** (34.9 mg, 0.05 mmol, 1.0 eq), phenanthroline (18.0 mg, 0.1 mmol, 2.0 eq), K_2CO_3 (20.7 mg, 0.15 mmol, 3.0 eq) and DCE (0.25 mL, 0.4 M) in a pressure tube and the mixture was stirred at 60 °C for 2 h. After cooling to room temperature, the reaction was filtrated through Celite® and rinsed with DCM. The solvent was removed under reduced pressure and to the crude mixture CH_2Br_2 (7.08 µL, 0.1 mmol) was added as internal standard, the mixture was dissolved in $CDCl_3$ and ^{1}H NMR was measured.

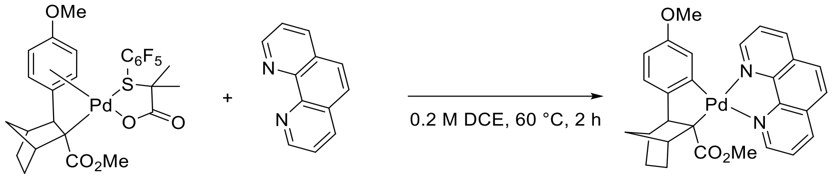

C1 2 eq. **C3**

Table S16. Conditions to obtain complex **C3**

#	Additive	NMR yield [%] **C3**
1	-	30
2	3 eq. K_2CO_3	60

Complex C3

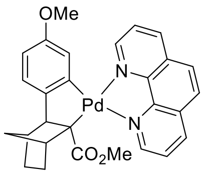

C3

Complex **C3** was synthesized by adding complex **C1** (104.5 mg, 0.16 mmol, 1.0 eq), Phenanthroline (57.6 mg, 0.32 mmol, 2.0 eq), K_2CO_3 (66.3 mg, 0.48 mmol, 3.0 eq) and DCE (0.8 mL, 0.2 M) in a pressure tube and the mixture was stirred for 2 h at 60 °C. After cooling to room temperature, the reaction was filtrated through Celite® and rinsed with DCM. Purification by column chromatography on silica gel using Cy / EtOAc (1:1-0:1 v/v) as an eluent provided the title compound as a yellow solid (55% yield, 48,0 mg, 0.9 mmol). **R$_f$** = 0.14 (Pentane / EtOAc = 3:7) **^{1}H NMR** (400 MHz, CD_2Cl_2) δ 10.24 (d, *J* = 5.0 Hz, 1H), 9.49 (d, *J* = 4.9 Hz, 1H), 8.48 (dd, *J* = 14.2, 8.1 Hz, 2H), 7.94 (m, 3H), 7.87 (dd, *J* = 8.1, 5.1 Hz, 1H), 6.97 (d, *J* = 8.2 Hz, 1H), 6.83 (d, *J* = 2.7 Hz, 1H), 6.59 (dd, *J* = 8.2, 2.7 Hz, 1H), 3.79 (s, 3H), 3.52 (s, 3H), 3.11 (d, *J* = 8.8 Hz, 1H), 3.02 (s, 1H), 2.86 – 2.80 (m, 1H), 2.24 (s, 1H), 1.72 (dd, *J* = 14.5, 5.7 Hz, 1H), 1.61 – 1.54 (m, 2H), 1.39 – 1.29 (m, 2H). **^{13}C NMR** (101 MHz, CD_2Cl_2) δ 179.93, 160.64, 157.69, 156.13, 152.68, 151.54, 146.38, 146.37, 137.93, 137.63, 130.00, 129.79, 127.70, 126.96, 125.44, 124.96, 123.20, 120.23, 109.26, 62.55, 61.60, 55.44, 50.61, 45.78, 45.38, 39.53, 30.90, 26.13. **HRMS** (FD): *m/z* calculated for $C_{28}H_{26}N_2O_3Pd$ $[M]^+$= 544.0989; found= 544.0992. **IR** (neat): ν_{max} (cm^{-1}): 2942, 2866, 2358, 1738, 1729, 1722, 1688, 1679, 1658, 1650, 1643, 1631, 1613, 1600, 1579, 1563, 1555, 1513, 1502, 1493, 1468, 1462, 1453, 1428, 1346, 1298, 1276, 1237, 1216, 1201, 1183, 1142, 1118, 1076, 1044, 866, 841, 805, 775, 725, 423, 415. **Thermal decomposition** around 194 °C.

11.5. NMR Experiments of Complex C1, C1-*ortho*, C2 and C3 Complex C1

Figure S12. Structure assignment of complex **C1** in ^{1}H-NMR.

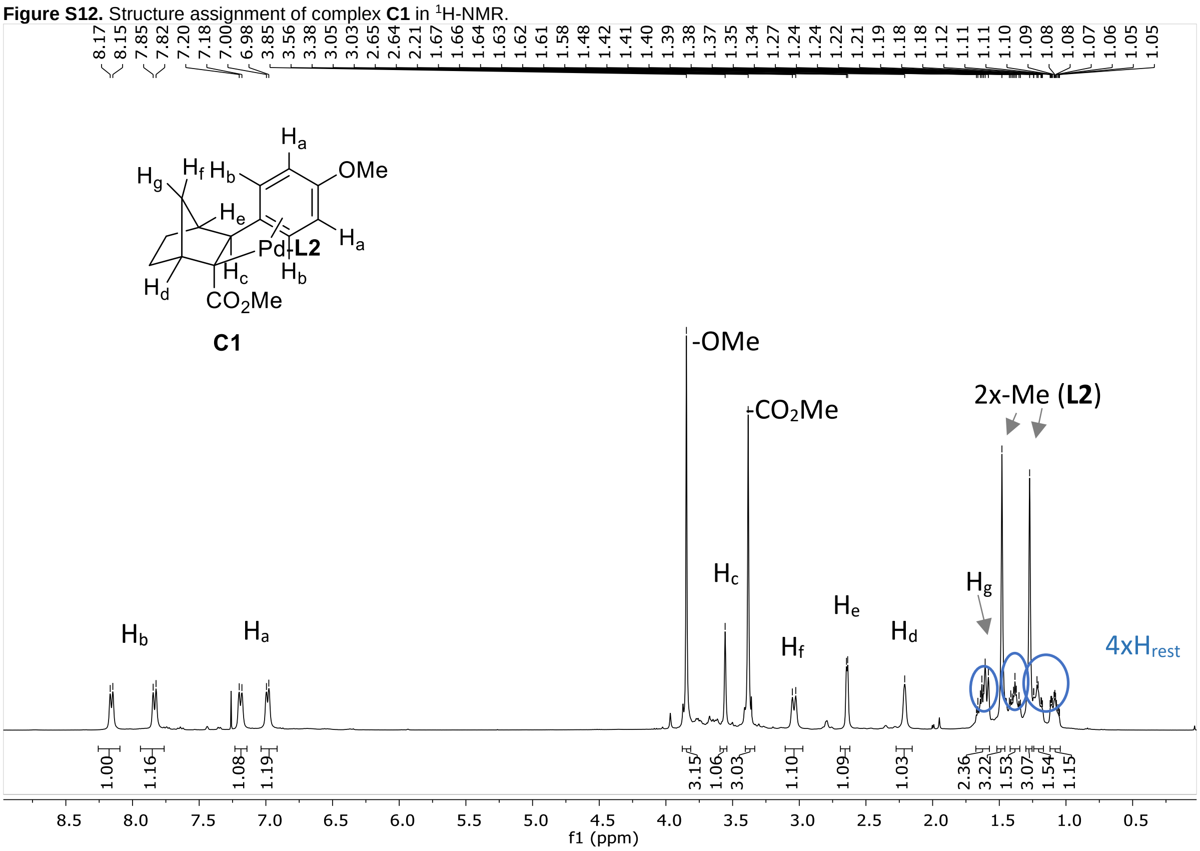

Figure S12. Structure assignment of Complex C1 in ^{13}C-NMR

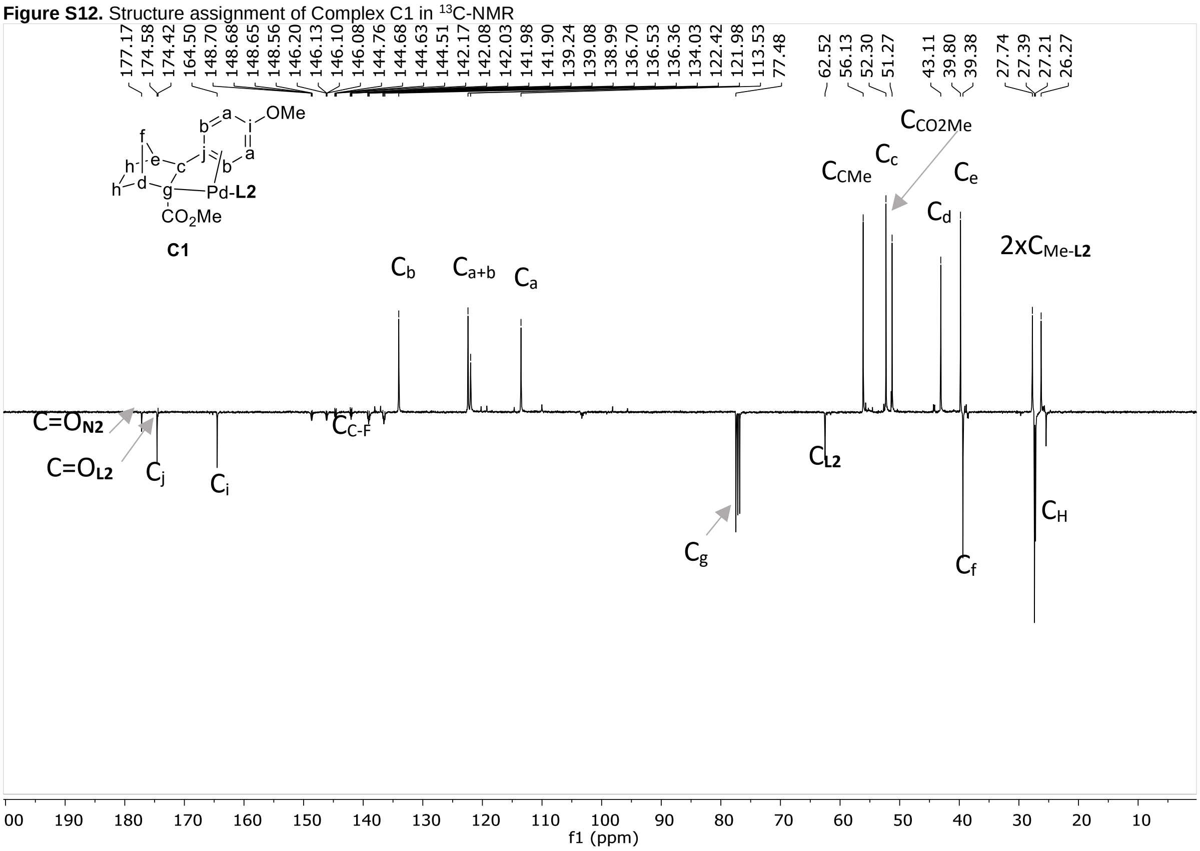

Figure S13. The structure assignment of complex **C1** by NOE NMR spectroscopy.

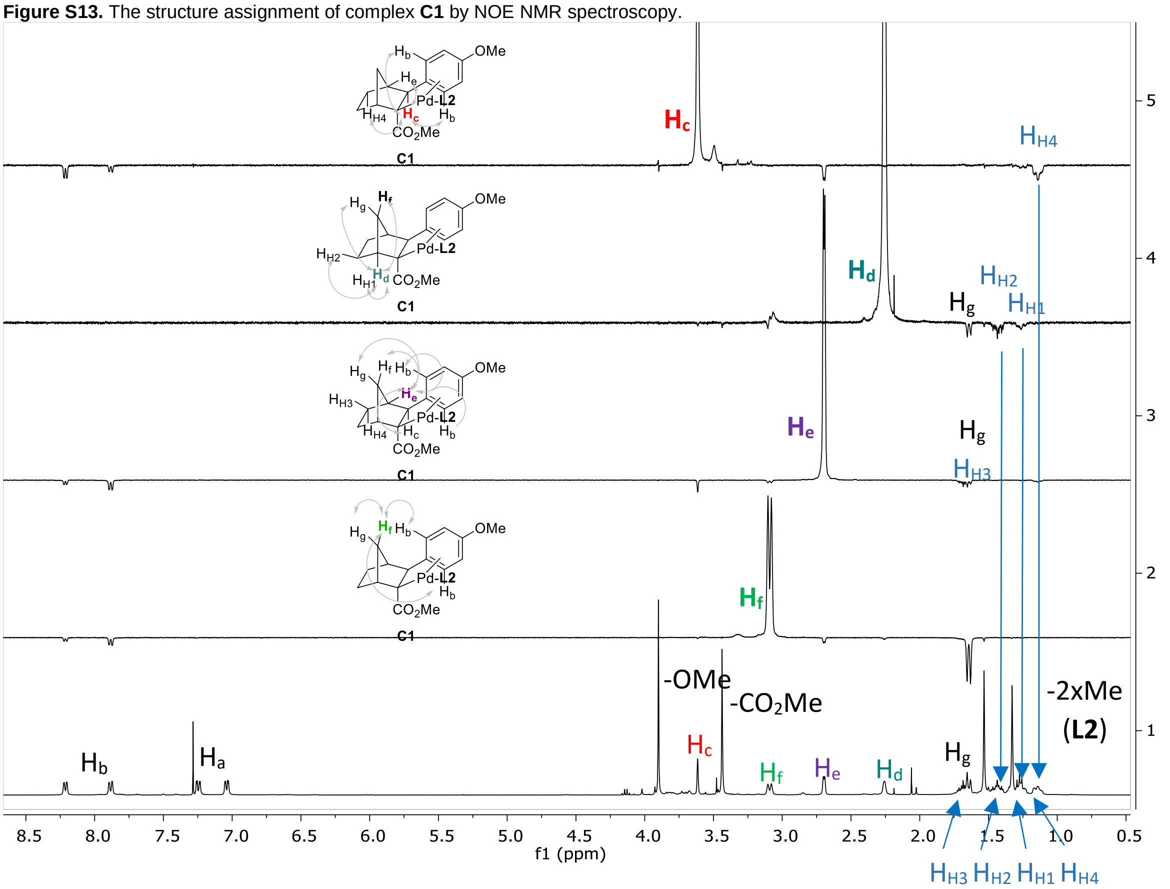

Figure S14 The structure assignment of complex **C1** by HSQC NMR spectroscopy.

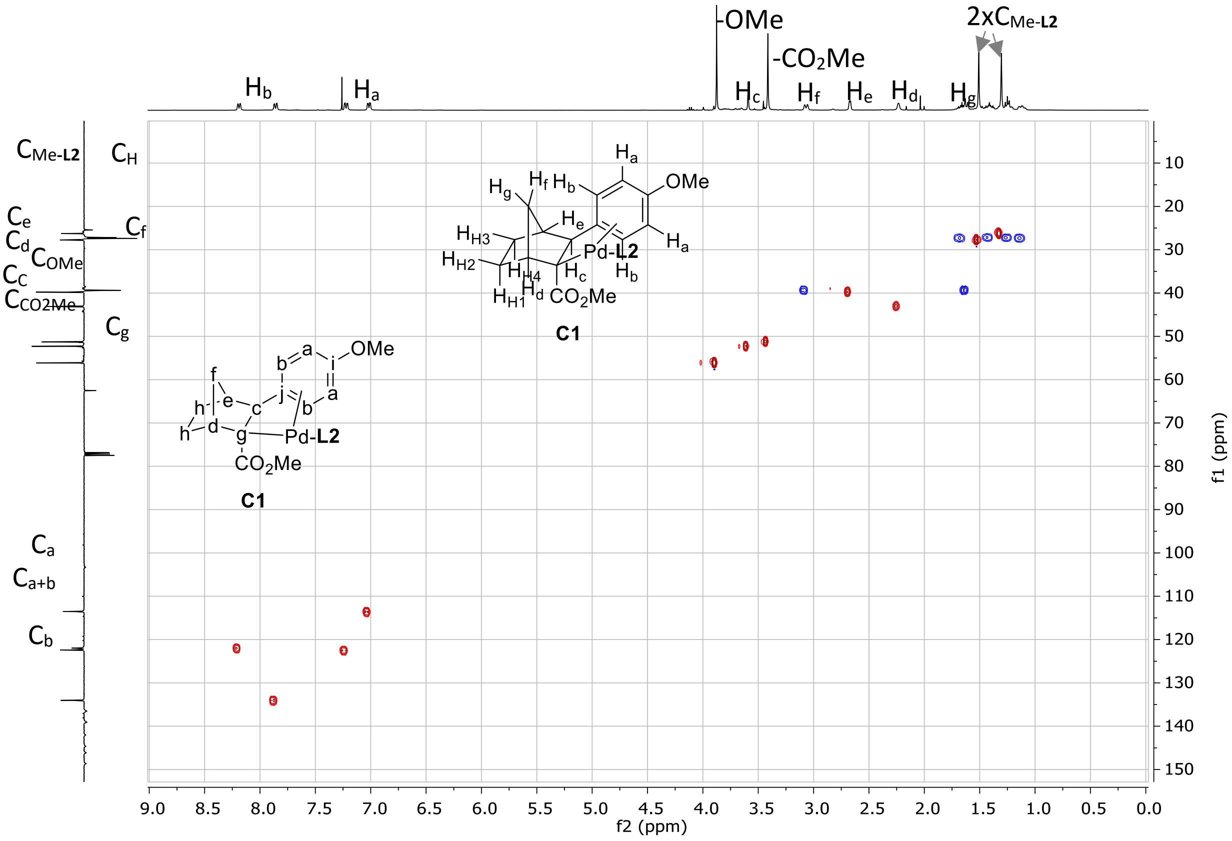

Complex C1-*ortho*

The attempts to isolate **C1-*ortho*** purely failed. Therefore, the crude ^{1}H NMR spectra is shown in Figure Xb, which shows a ratio of the **C1:C1-*ortho*** 1:1.5. [Note: Solvents (HFIP, DCM and the internal standard (IS)) are present in the spectra. The attempt to dry the crude mixture further resulted in the formation of byproducts.] The pure NMR of **C1** is shown in Figure Xa for comparison. All protons can be identified. For the carbons, representative signals are given and and confirmed with HSQC.

Figure S16. Indicative peaks of complex **C1**-*ortho* in crude NMR compared to NMR of complex **C1**. a) ^{1}H-NMR of Complex C1; b) Crude ^{1}H-NMR of complex C1-otho (A mixture of complex **C1:C1-*ortho*** 1:1.5*)*.

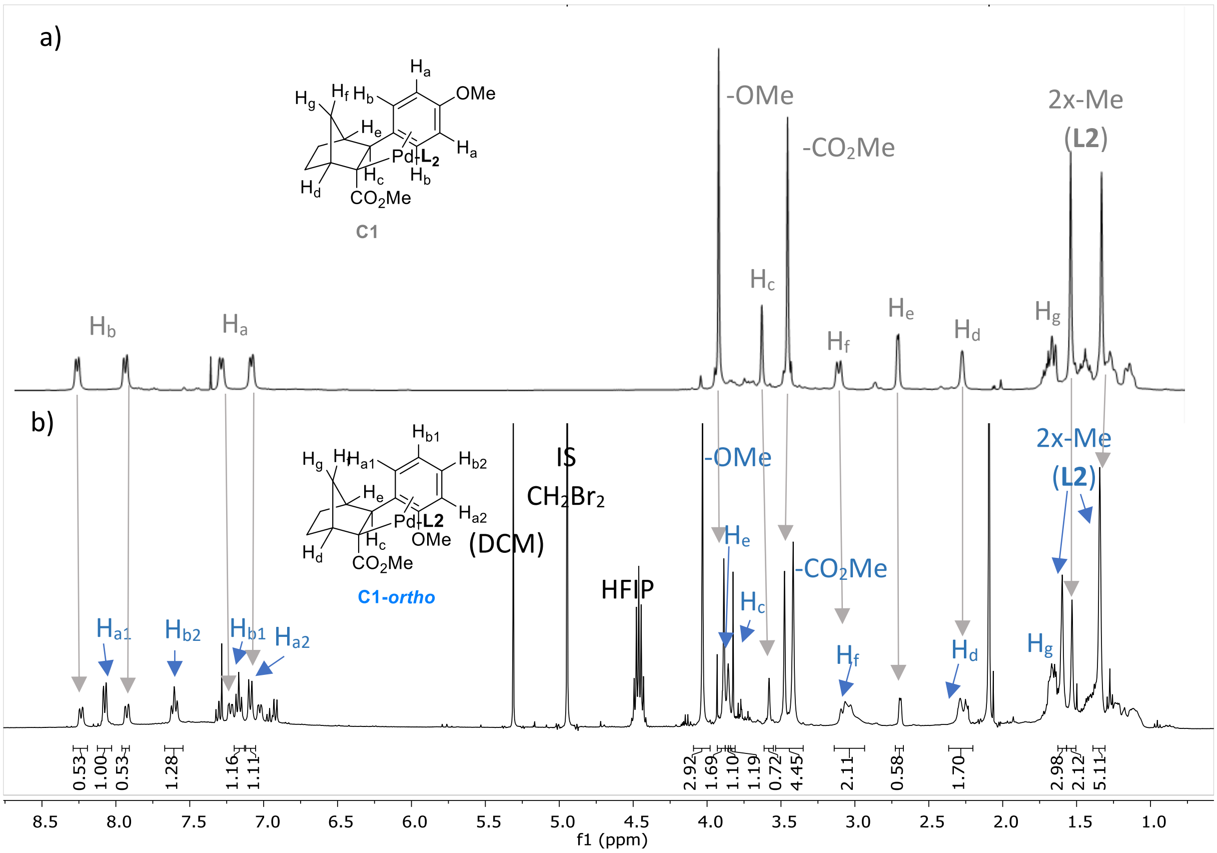

Figure S17. The structure assignment of complex **C1-ortho** by 2D COSY NMR spectroscopy (selected region). A mixture of complex **C1:C1-*ortho*** 1:1.5.

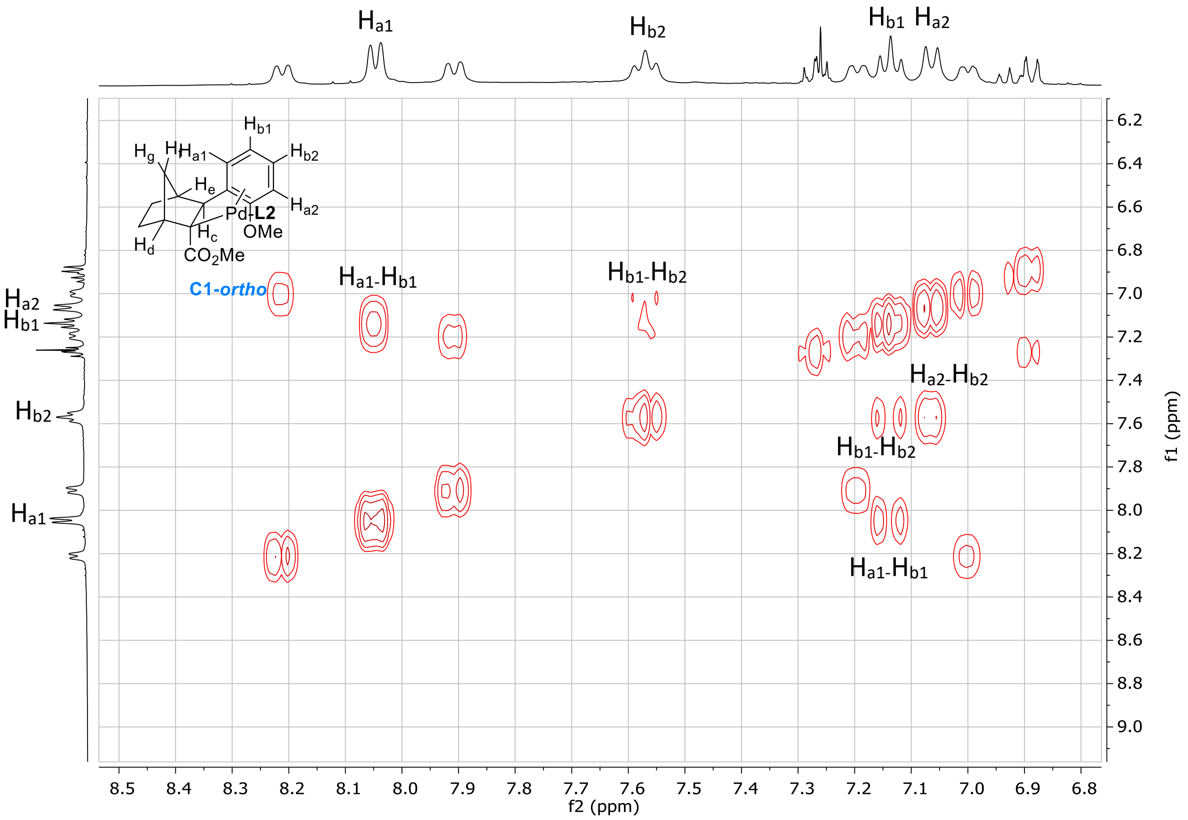

Figure S18. The structure assignment of complex **C1-ortho** by 2D NOSEY NMR spectroscopy (selected region). A mixture of complex **C1:C1-*ortho*** 1:1.5.

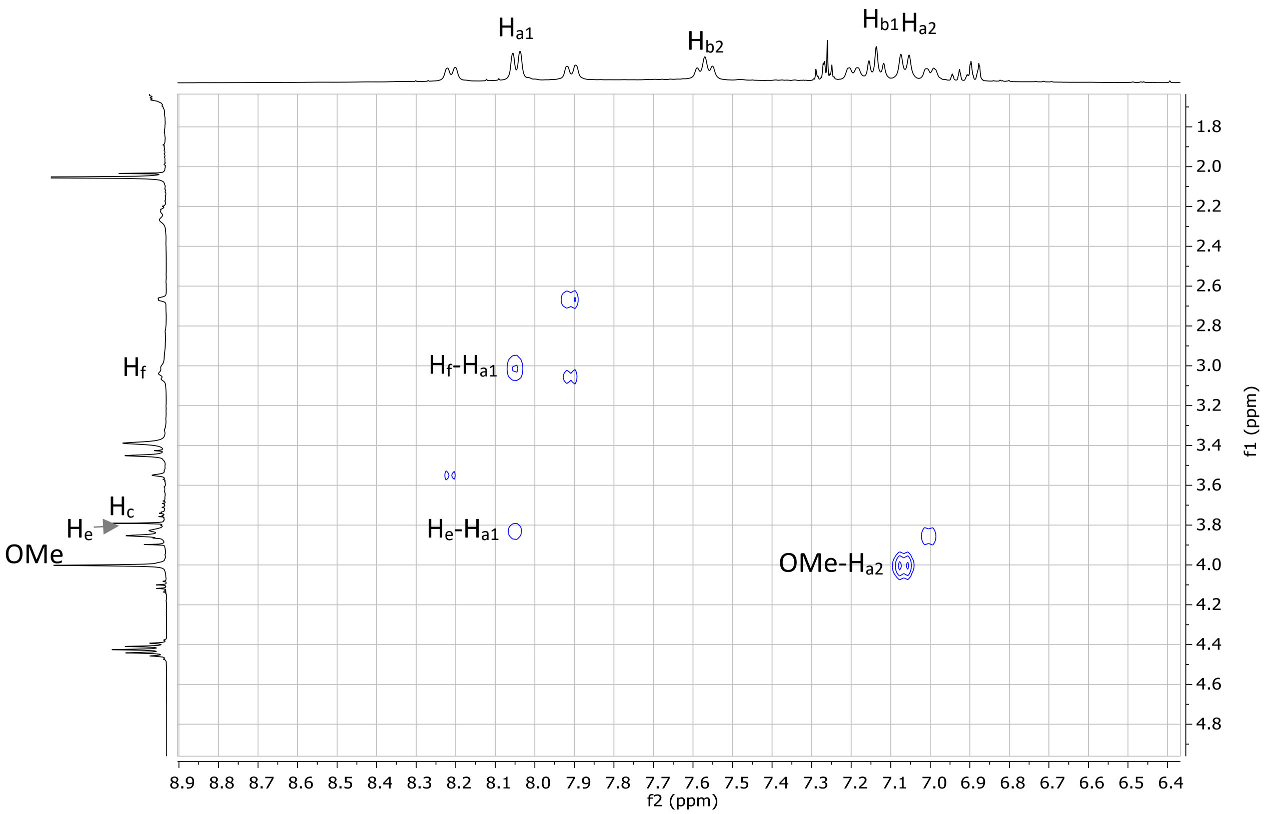

Figure 19. Indicative peaks of complex **C1**-ortho in crude ^{13}C NMR compared to NMR of complex **C1.** a) ^{13}C-NMR of Complex C1; b) Crude ^{13}C-NMR of complex **C1-*ortho*** (A mixture of complex **C1:C1-*ortho*** 1:1.5).

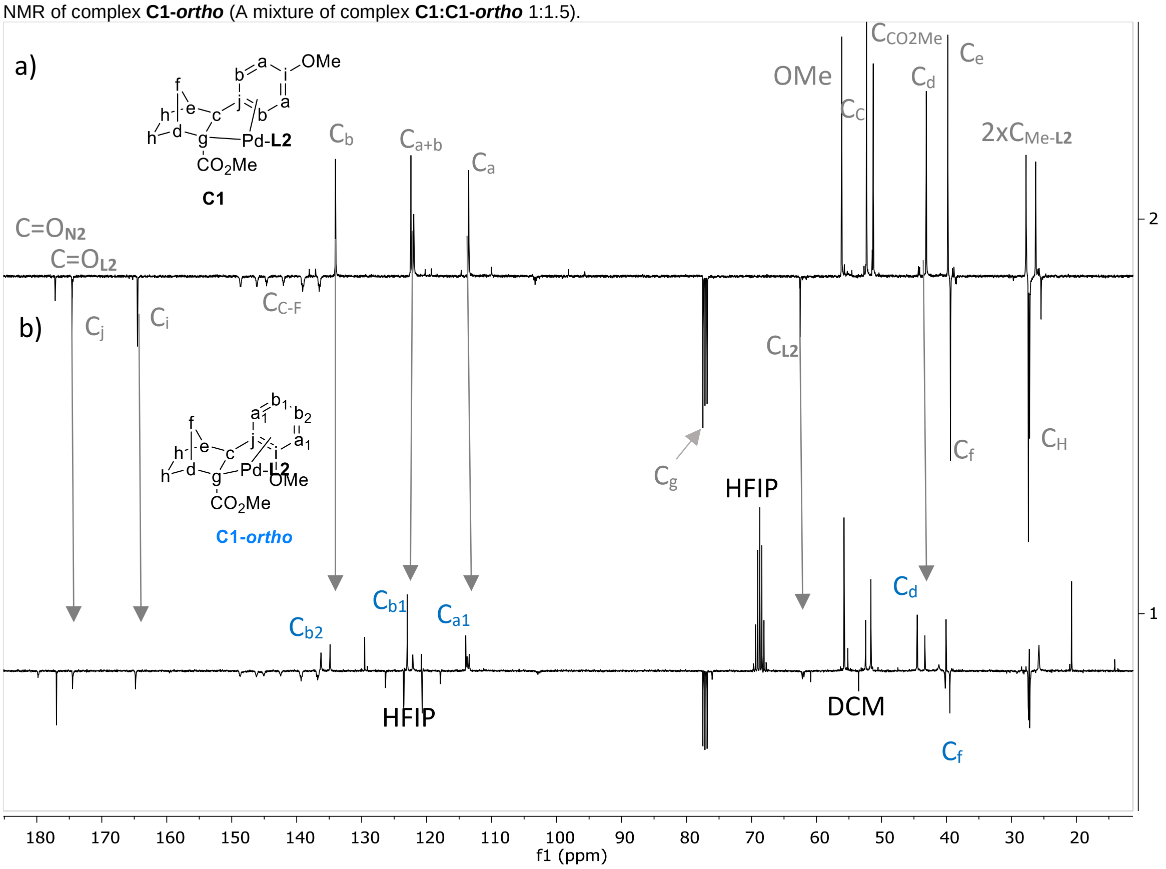

Figure S20. The structure assignment of complex **C1-*ortho*** by HSQC NMR spectroscopy. A mixture of complex **C1:C1-*ortho*** 1:1.5.

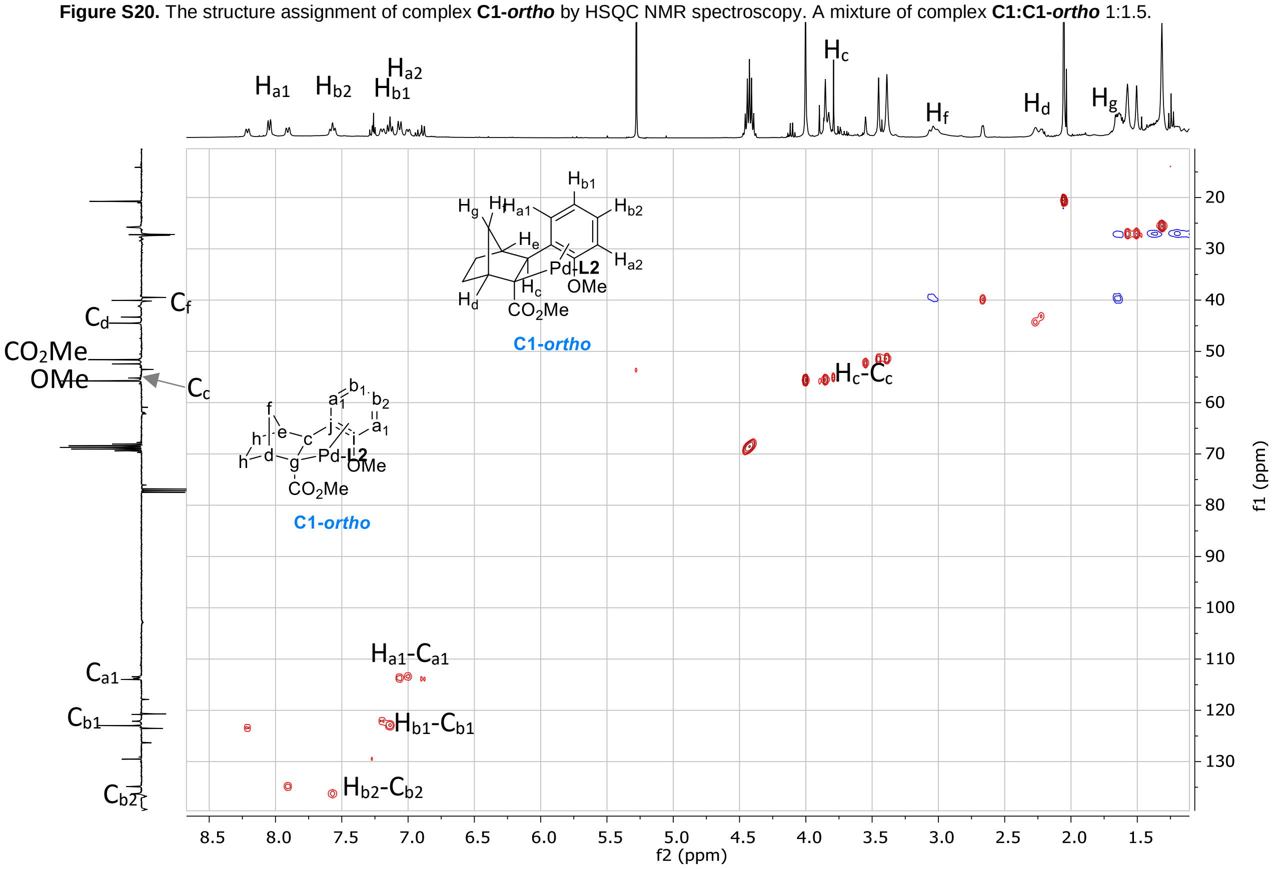

Complex C2

Figure S215. Structure assignment of complex **C2** in ^{1}H-NMR.

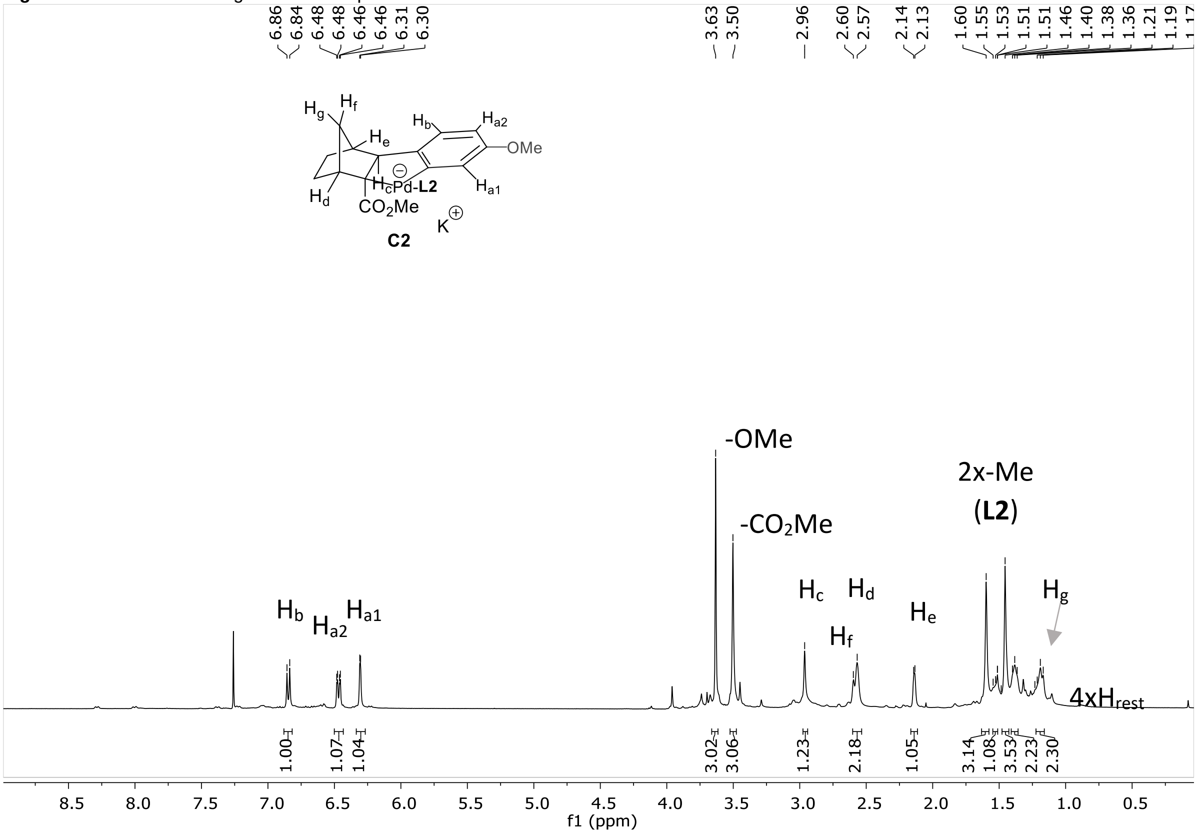

Figure 22. Structure assignment of Complex **C2** in ^{13}C-NMR.

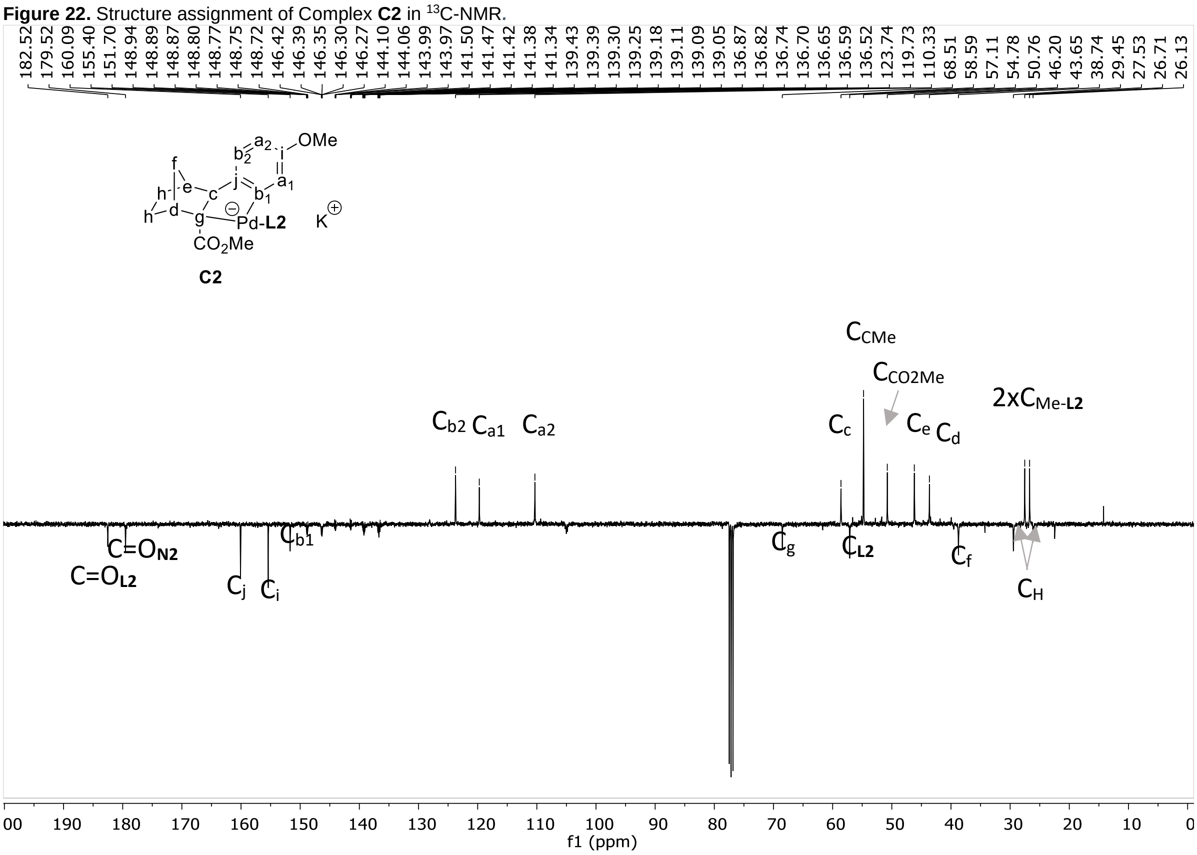

Figure S23. The structure assignment of Complex **C2** by NOE NMR spectroscopy.

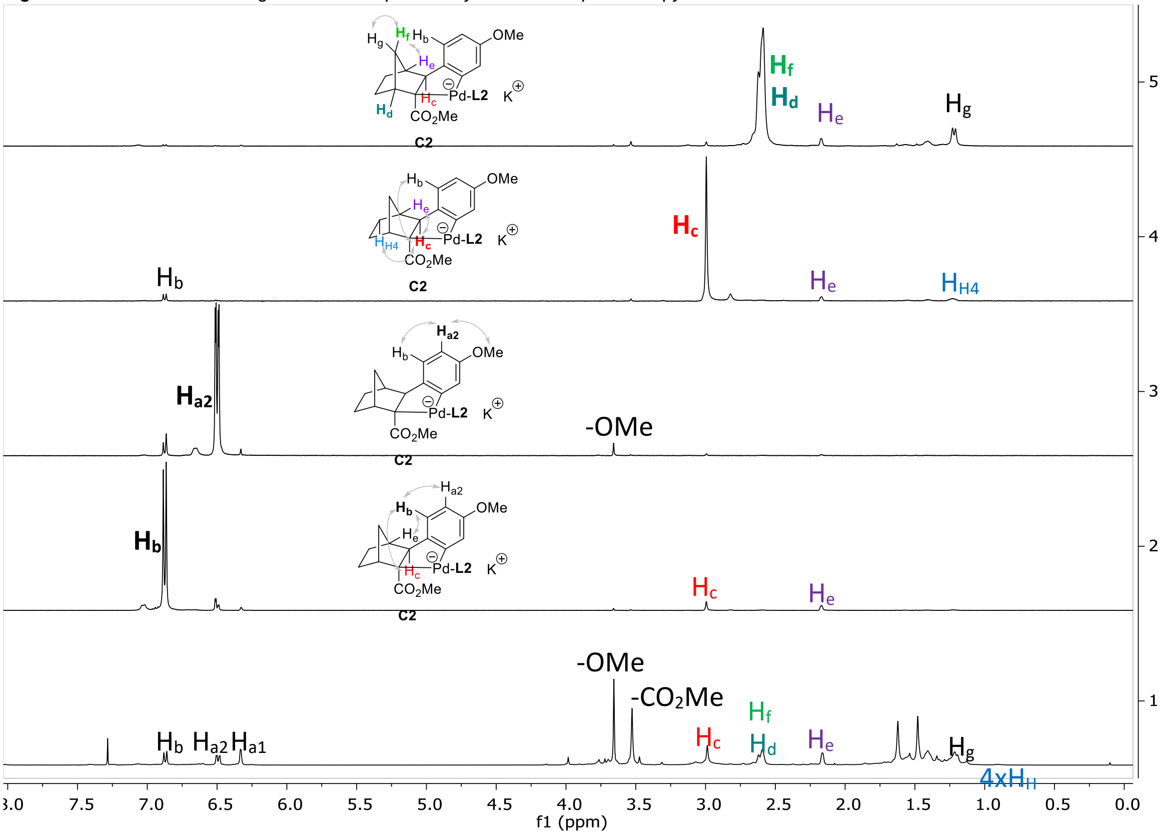

Figure S24 The structure assignment of complex **C2** by HSQC NMR spectroscopy.

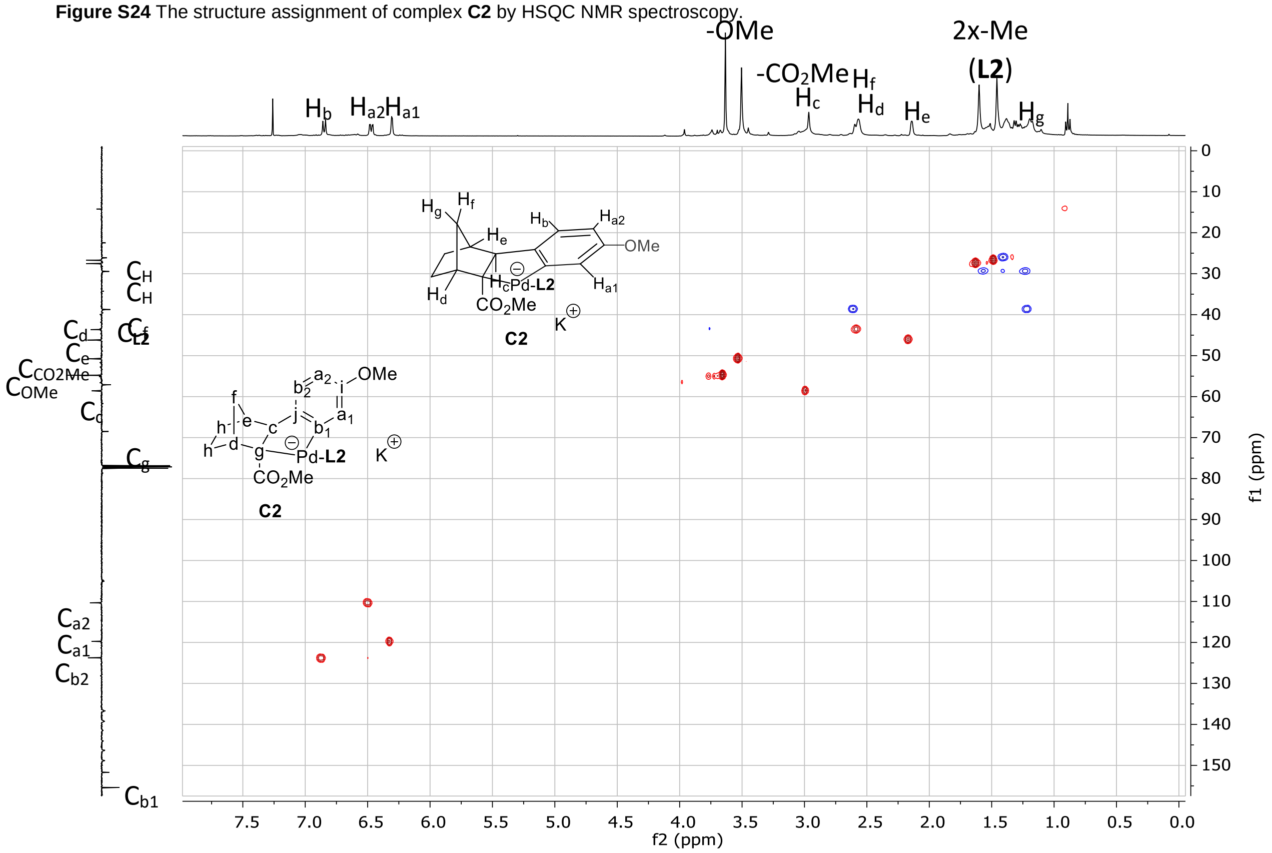

Complex C3

Figure S25 The structure assignment of Complex **C3** by COSY NMR spectroscopy.

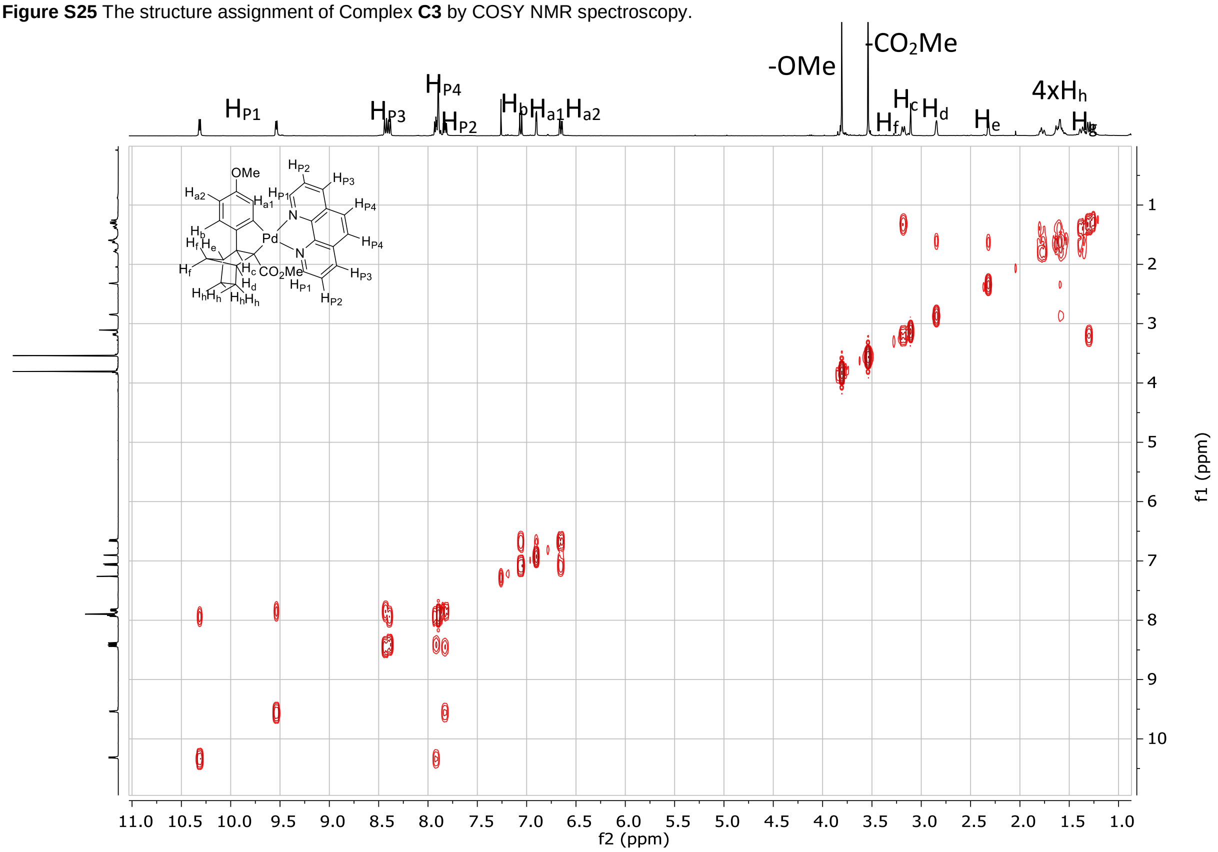

Figure 26. The structure assignment of Complex **C3** by NOSEY NMR spectroscopy.

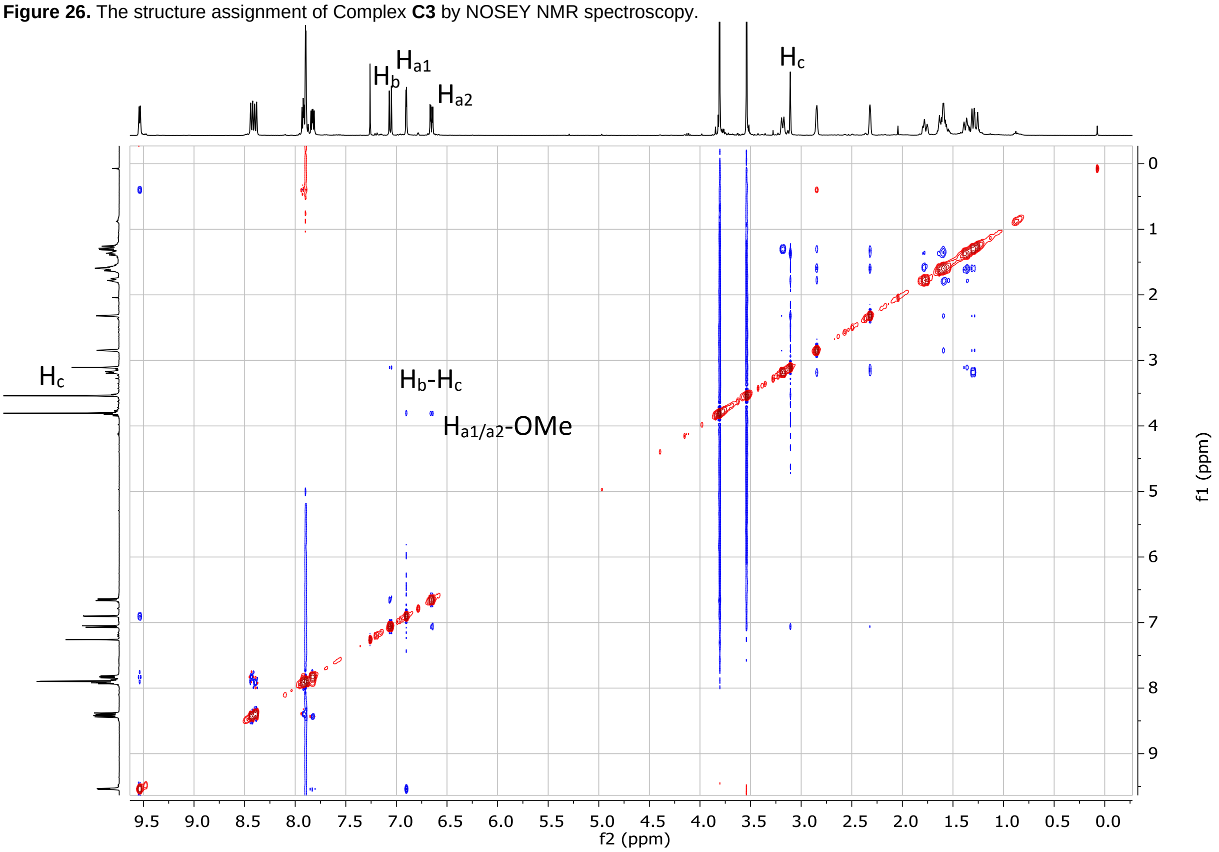

Figure S27. The structure assignment of Complex **C3** by NOSEY NMR spectroscopy (selected area).

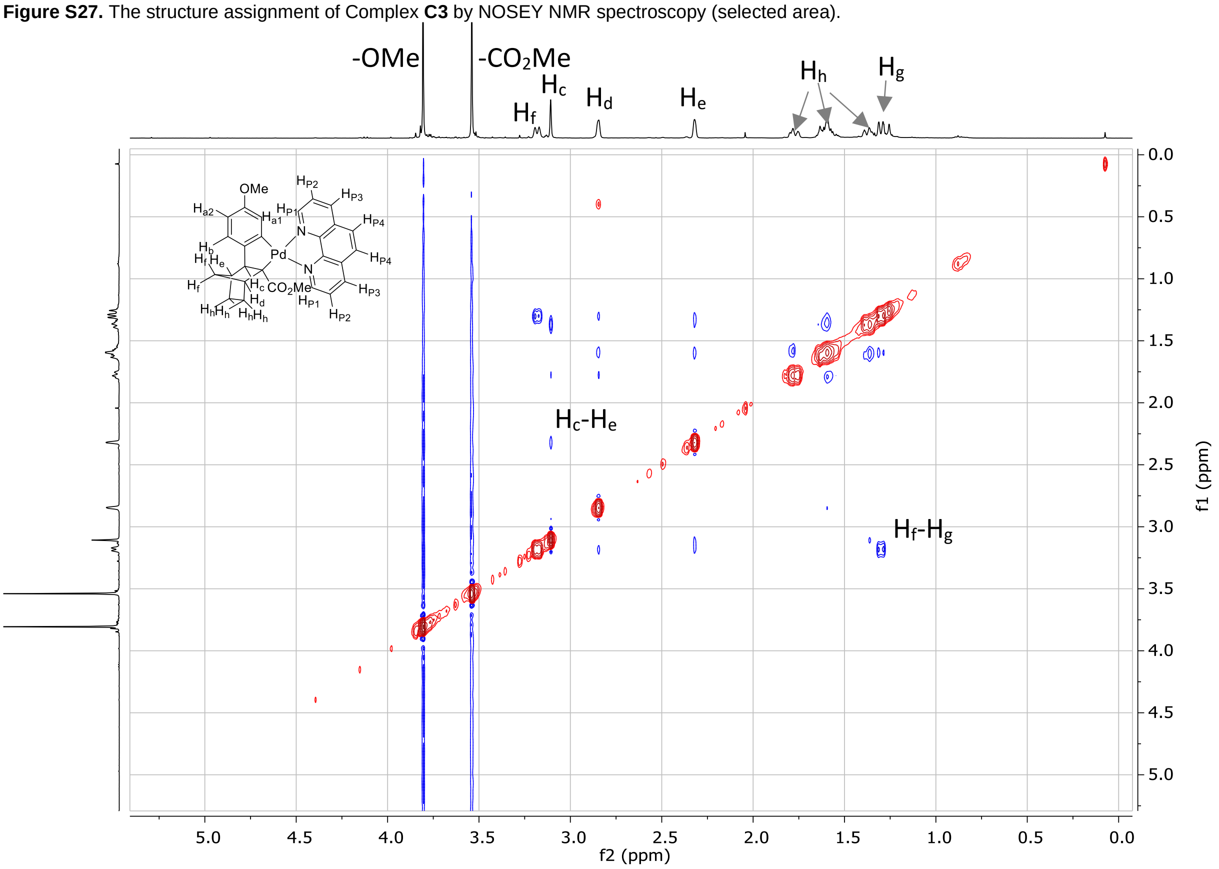

Figure S28. The structure assignment of complex **C3** by HSQC NMR spectroscopy.

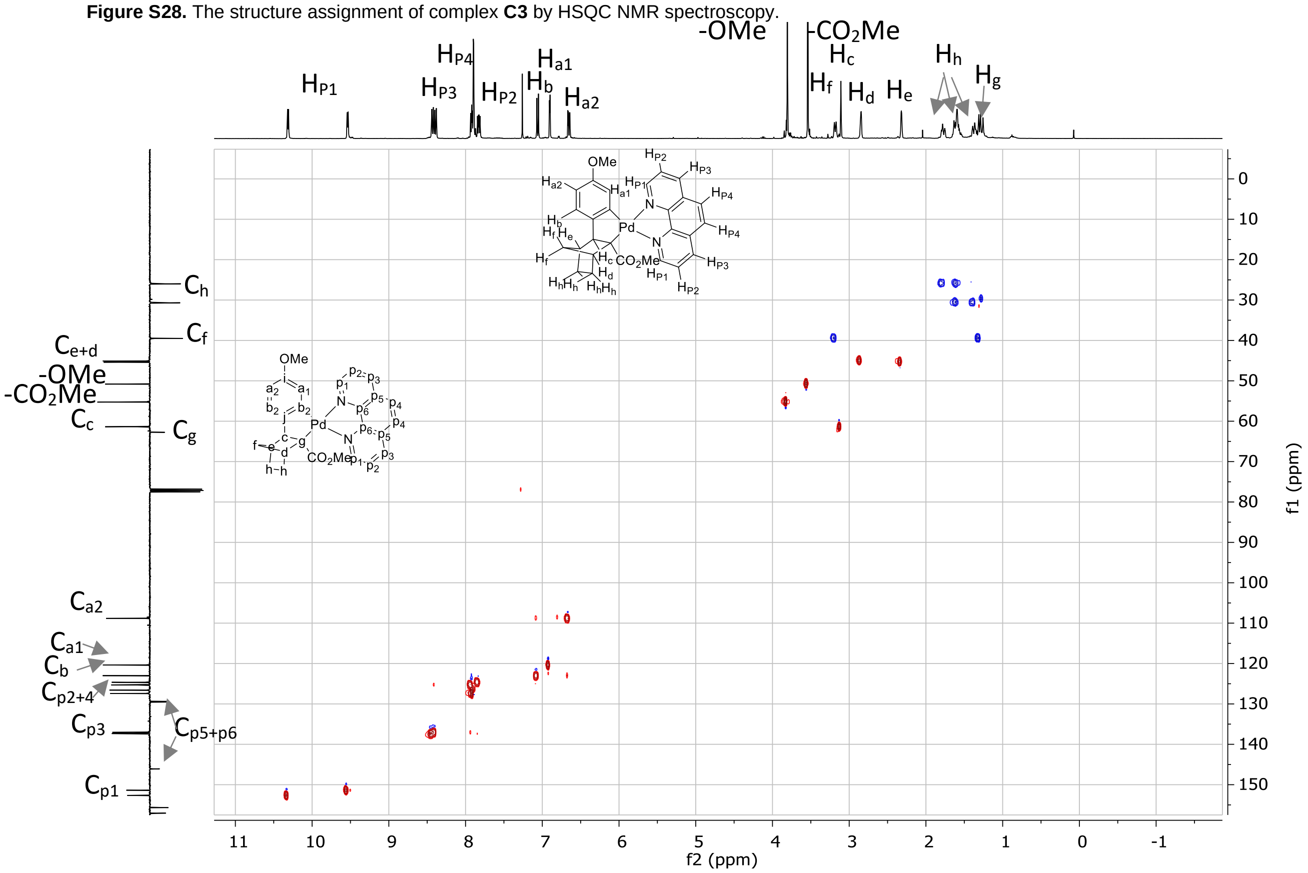

11.6. Evaluation of the Catalytic Activity of Complexes C1-C3

In a pressure tube containing a suitable stirring bar the indicated complex (10mol%), **N2** (6.0 mg, 0.04 mmol, 0.4 eq.), silver acetate (25 mg, 0.15 mmol, 1.5 eq.), aryl halide **2** (40.1 mg, 0.15 mmol, 1.5 eq.), anisole (11µL, 0.1 mmol, 1.0 eq.) and HFIP (0.25 mL, 0.4 M) were added. The tube was put into a pre-heated oil bath at 90 °C and was stirred for 24 h. After cooling to room temperature, the reaction was filtered through Celite® and rinsed with EtOAc. The solvent was evaporated under reduced pressure. To the crude mixture, CH_2Br_2 (7.08 µL, 0.1 mmol) was added as internal standard, the mixture was dissolved in $CDCl_3$ and ^{1}H NMR was measured.

Table S17. Determination of catalytically active complex

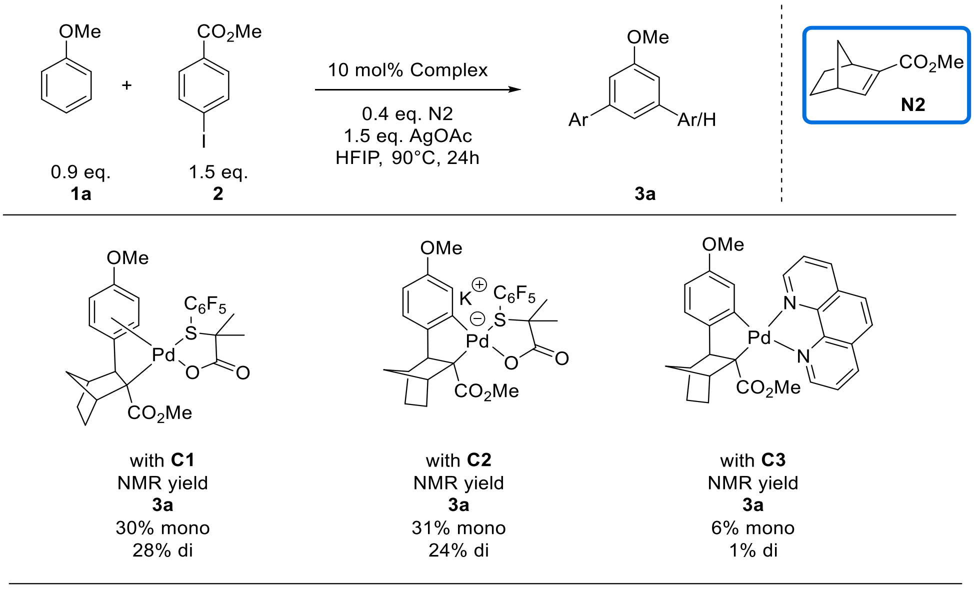

11.7. Deuterium Experiments

For the deuterium experiments the indicated complex (0.05 mmol) and AcOD-d_4 (0.25 mL, 0.2M) was added to a pressure tube containing a suitable stirring bar. The tube was put into a pre-heated oil bath and was stirred for the indicated time. The solvent was evaporated under reduced pressure. To the crude mixture CH_2Br_2 (7.08 µL, 0.1 mmol) was added as internal standard, the mixture was dissolved in $CDCl_3$ and ^{1}H NMR was measured.

Figure S29 Reversibility experiment with deuterated acetic acid and HFIP-d with complex **C1.**

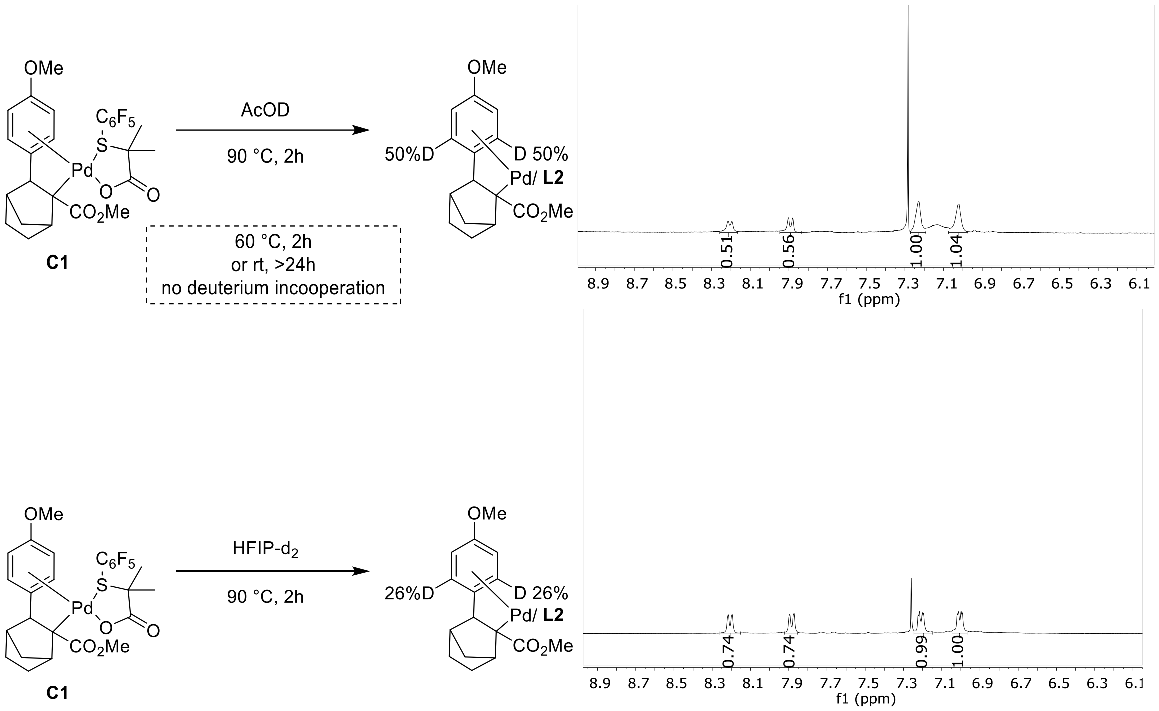

Figure S30 Treatment of complex **C2** with deuterated acetic acid.

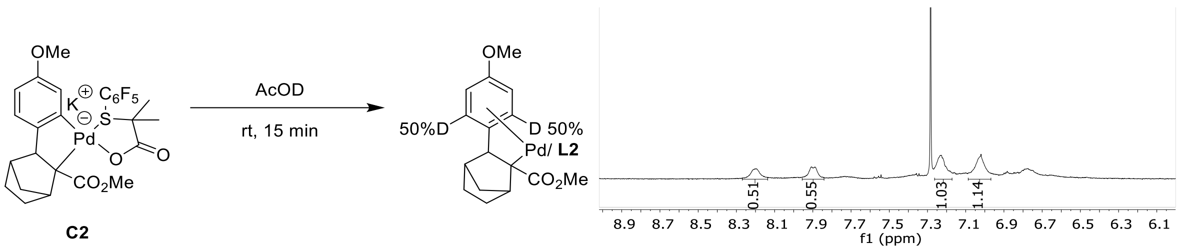

In a pressure tube containing a suitable stirring bar, **N2** (7.6 mg, 0.05 mmol, 0.5 eq.), $Pd(OAc)_2$ (2.2 mg, 0.01 mmol, 10 mol%), silver acetate (25.0 mg, 0.15 mmol, 1.5 eq.), aryl halide **2a** (40.1 mg, 0.15 mmol, 1.5 eq.), anisole (11 µL ,0.25 mmol, 1.0 eq.), **L2** (2.8 mg, 0.01mmol, 10 mol%) and HFIP-d_2 (0.25 mL, 0.4 M) were added. The tube was put into a pre-heated oil bath at 90 °C and was stirred for 24 h. After cooling to room temperature, the reaction was filtered through Celite® and rinsed with EtOAc. The solvent was evaporated under reduced pressure. To the crude mixture, CH_2Br_2 (17.7 µL, 0.25 mmol) was added as internal standard, the mixture was dissolved in $CDCl_3$ and the ^{1}H NMR was measured. Purification by column chromatography on silica gel using Cy / DCM (9:1-7:3 v/v) was done to separate the mono and di product **3a**.

Additionally, complex **C2** was stirred in HFIP for 15 min at room temperature. After the solvent was removed, a mixture of **C1**:**C2** in a ratio 2 to 1 was observed by ^{1}H-NMR ($CDCl_3$), further confirming the reversibility of the *meta*-C–H activation.

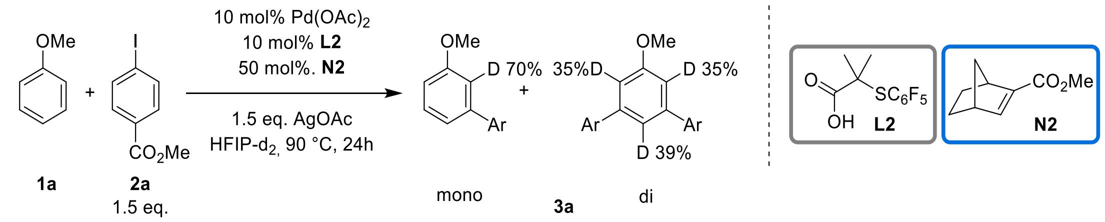

Figure S31. ^{1}H-NMR of isolated products of standard reaction performed in deuterated HFIP.

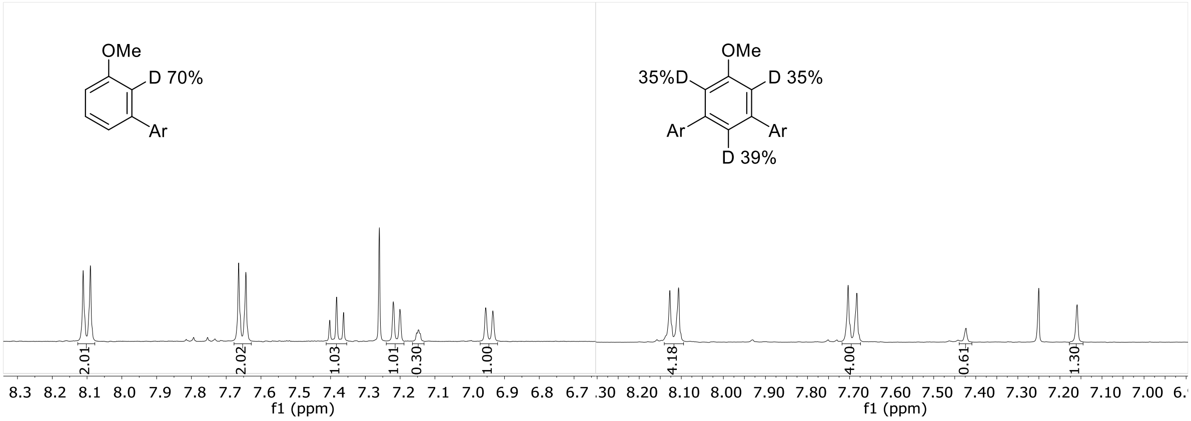

11.8. Kinetic Order of Anisole in the Catalytic *meta*-C–H Arylation

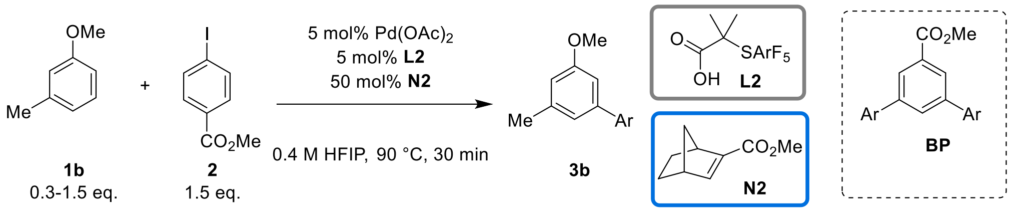

The reaction order of anisole in the arylation reaction with 4-iodotoluene was determined using the method of initial rates.[30] A stock solution of **N2** (60.8 mg, 8*0.05 mmol), Pd(OAc)$_2$ (8.8 mg, 8*0.005 mmol), S,O-ligand **L2** (11.4 mg, 8*0.005 mmol) in 0.8 mL HFIP was prepared. Six pressure tubes containing a suitable stirring bar were equipped with methyl 4-iodobenzoate (40.1 mg, 0.15 mmol, 1.5 eq.), silver acetate (25 mg, 0.15 mmol, 1.5 eq.), the indicated amount of 3-methylanisole **1b** (3.8-19.2 µL, 0.03-0.15 mmol, 0.3-1.5 eq.), 100 µL of the stock solution (reaction scale: 0.1 mmol) and 0.15 mL HFIP. The tubes were put into a pre-heated oil bath at 90 °C and were stirred for 30 min. After cooling to room temperature, each reaction was filtered through Celite® and rinsed with EtOAc. To each crude mixture, CH_2Br_2 (7.08 µL, 0.1 mmol) was added as internal standard, the mixture was dissolved in $CDCl_3$ and ^{1}H NMR was measured.

Table S28. Reaction Order of Anisole

#	Anisole **1b** [eq.]	Anisole **1b** [mmol]	Anisole **1b** [µL]	Anisole 1**b** [M]	^{1}H-NMR yield **3b** [%]	Rate [M·min^{-1}]
1	0.3	0.03	3.8	0.12	8	0,00027
2	0.5	0.05	6.4	0.2	8	0,00027
3	0.75	0.075	9.6	0.3	9	0,0003
4	1.0	0.10	12.8	0.4	8	0,00027
5	1.25	0.125	16.0	0.5	8	0,00027
5	1.5	0.15	19.2	0.6	8	0,00027

Figure S32. Plots of Reaction Order of Anisole (right logarithms)

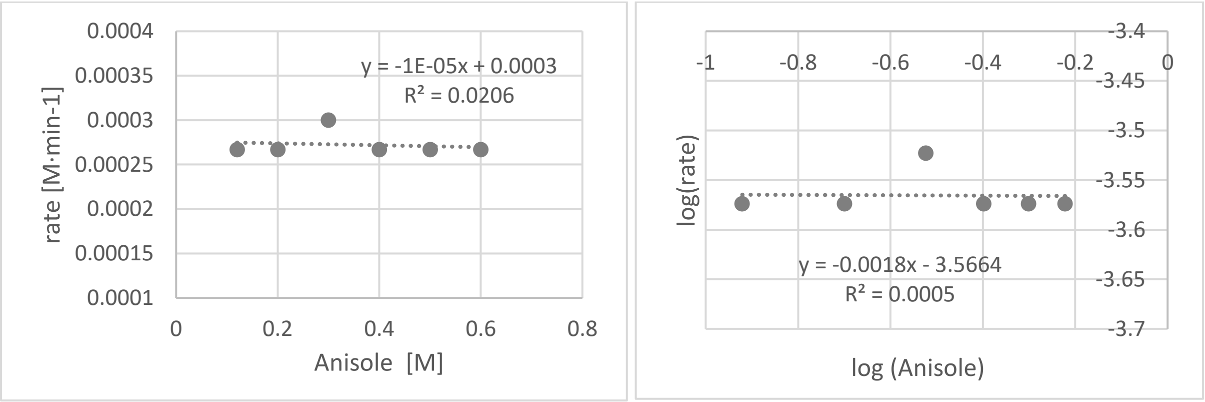

The plot of the logarithms of the reaction rate against the concentration of anisole **1b** provides a straight line with slope close to zero, revealing that the reaction is zero order in anisole.

12. Single-Crystal X-Ray Diffraction Studies

A crystal suitable for X-ray crystallography was obtained by dissolving 4 mg of C3 in 0.1 mL of DCM. The solution was transferred to a NMR tube, layered with heptane and left standing overnight.

X-ray diffraction data of compound **C3** were measured on a Bruker D8 Quest Eco diffractometer using graphite-monochromated (Triumph) Mo Ka radiation (l = 0.71073 Å) and a CPAD Photon III C14 detector. The sample was cooled with N_2 to 150 K with a Cryostream 700 (Oxford Cryosystems). Intensity data were integrated using the SAINT software.[31] Absorption correction and scaling was executed with SADABS.[32] The structures were solved using intrinsic phasing with the program SHELXT 2018/2[33] against F2 of all reflections. Least-squares refinement was performed with SHELXL-2018/3.[34] All non-hydrogen atoms were refined with anisotropic displacement parameters. The hydrogen atoms were introduced at calculated positions with a riding model. The X-ray crystallographic data for **C3** was deposited at the Cambridge Crystallographic Data Centre (CCDC), under the deposition number CCDC 2128582.

Figure S33. ORTEP diagram of **C3** with 50% probability ellips.

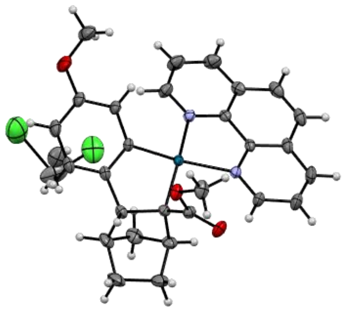

C3: $C_{28}H_{26}N_2O_3Pd \cdot CH_2Cl_2$, Fw = 629.83, 0.331 × 0.283 × 0.108, triclinic, $P\bar{1}$, (No:2), a = 10.2156(4), b = 11.4530(4), c = 12.6393(4) Å, α = 99.6810(10) , β = 99.3050(10) , γ = 112.1040(10)°, V = 1308.97(8) Å^3, Z = 2, D_x = 1.598 g cm^{-3}, μ = 0.948 mm^{-1}. 94029 Reflections were measured up to a resolution of $(\sin \theta/\lambda)_{max}$ = 0.61 Å. 12073 Reflections were unique (R_{int} = 0.0371), of which 10951 were observed [I>2σ(I)]. 346 Parameters were refined with 0 restraints. R1/wR2 [I > 2σ(I)]: 0.0483 /_0.1306. R1/wR2 [all refl.]: 0.0549 /_0.1355. S = 1.098. Residual electron density between -2.109 and 2.389 e Å^{-3}. CCDC 2128582

13. References

[1] M. Tobisu, Y. Kita, Y. Ano, N. Chatani, *J. Am. Chem. Soc.* **2008**, *130*, 15982–15989.

[2] A. A. H. Elmehriki, J. L. Gleason, *Org. Lett.* **2019**, *21*, 9729–9733.

[3] J. C. Conrad, J. Kong, B. N. Laforteza, D. W. C. MacMillan, *J. Am. Chem. Soc.* **2009**, *131*, 11640–11641.

[4] M. M. Gruza, J. C. Chambron, E. Espinosa, E. Aubert, *Eur. J. Org. Chem.* **2009**, *36*, 6318–6327.

[5] K. Naksomboon, C. Valderas, M. Gómez-Martínez, Y. Álvarez-Casao, M. Á. Fernández-Ibáñez, *ACS Catal.* **2017**, *7*, 6342–6346.

[6] R. Li, G. Dong, *Angew. Chem., Int. Ed.* **2018**, *57*, 1697–1701.

[7] J. Wang, Z. Dong, C. Yang, G. Dong, *Nat. Chem.* **2019**, *11*, 1106–1112.

[8] J. Wang, R. Li, Z. Dong, P. Liu, G. Dong, *Nat. Chem.* **2018**, *10*, 866–872.

[9] H. D. Verkruijsse, L. Brandsma, *Recl. des Trav. Chim. des Pays-Bas* **1986**, *105*, 66–68.

[10] P. X. Shen, X. C. Wang, P. Wang, R. Y. Zhu, J. Q. Yu, *J. Am. Chem. Soc.* **2015**, *137*, 11574–11577.

[11] Y. Wang, J. Liu, L. Huang, R. Zhu, X. Huang, R. Moir, J. Huang, *Chem. Commun.* **2017**, *53*, 4589–4592.

[12] D. Liang, Y. Zou, Q. Wang, A. Goeke, *J. Org. Chem.* **2014**, *79*, 6726–6731.

[13] L. A. Paquette, K. Dahnke, J. Doyon, Wei He, K. Wyant, D. Friedrich, *J. Org. Chem* **1991**, *56*, 6199–6205.

[14] S. A. Green, T. R. Huffman, R. O. McCourt, V. Van Der Puyl, R. A. Shenvi, *J. Am. Chem. Soc.* **2019**, *141*, 7709–7714.

[15] X. Jiang, Z. Pan, C. J. Douglas, *Tetrahedron Lett.* **2015**, *56*, 5324–5327.

[16] B. D. Schwartz, E. Matoušová, R. White, M. G. Banwell, A. C. Willis, *Org. Lett.* **2013**, *15*, 1934–1937.

[17] D. R. Marshall, P. Reynolds-Warnhoff, E. W. Warnhoff, J. R. Robinson, *Can. J. Chem.* **1971**, *49*, 885–903.

[18] D. D. Gültekin, Y. Taşkesenligil, A. Daştan, M. Balci, *Tetrahedron* **2008**, *64*, 4377–4383.

[19] P. Laszlo, P. von Ragué Schleyer, *J. Am. Chem. Soc.* **1964**, *86*, 1171–1179.

[20] A. Mamantov, *Prog. React. Kinet. Mech.* **2004**, *29*, 243–288.

[21] B. M. Pretsch E., Bühlmann P., *^{1}H NMR Spectroscopy. In: Structure Determination of Organic Compounds.*, Springer, Berlin, Heidelberg, **2009**.

[22] H. Günther, *NMR Spectroscopy - Basic Principles, Concepts, and Applications in Chemistry*, Wiley-VCH Verlag GmbH & Co. KGaA, **2013**.

[23] S. A. Ponomarev, R. V. Larkovich, A. S. Aldoshin, A. A. Tabolin, S. L. Ioffe, J. Groß, T. Opatz, V. G. Nenajdenko, *Beilstein J. Org. Chem.* **2021**, *17*, 283–292.

[24] a) R. Li, Y. Zhou, X. Xu, G. Dong, *J. Am. Chem. Soc.* **2019**, *141*, 18958-18963; b) R. Li, G. Dong, *Angew. Chem. Int. Ed*. **2021**, *60*, 26184-26191; *Angew.Chem.* **2021**, *133*, 26388-26395

[25] L. Y. Liu, J. X. Qiao, K. S. Yeung, W. R. Ewing, J. Q. Yu, *J. Am. Chem. Soc.* **2019**, *141*, 14870–14877

[26] P. Wang, P. Verma, G. Xia, J. Shi, J. X. Qiao, S. Tao, P. T. W. Cheng, M. A. Poss, M. E. Farmer, K. S. Yeung, J. Q. Yu, *Nature* **2017**, *551*, 489-493

[27] a) B. J. Gorsline, L. Wang, P. Ren, B. P. Carrow, *J. Am. Chem. Soc.* **2017**, *139*, 9605–9614; b) L. Wang, B. P. Carrow, *ACS Catal.* **2019**, *9*, 6821–6836.

[28] L. Y. Liu, J. X. Qiao, K. S. Yeung, W. R. Ewing, J. Q. Yu, *Angew. Chem., Int. Ed.* **2020**, *59*, 13831–13835.

[29] P. Leowanawat, N. Zhang, A. M. Resmerita, B. M. Rosen, V. Percec, *J. Org. Chem.* **2011**, *76*, 9946–9955.

[30] J. H. Espenson, *Chemical kinetics and reaction mechanisms* (2nd ed.). McGraw Hill: 1987.

[31] Bruker, SAINT V8.40B, Bruker AXS Inc., Madison, Wisconsin, USA, 2001.

[32] L. Krause, R. Herbst-Irmer, G. M. Sheldrick, D. Stalke, *J. Appl. Cryst.* **2015**, *48*, 3–10

[33] G. M. Sheldrick, *Acta Cryst. Sect. A Found. Cryst.* **2015**, *A71*, 3–8.

[34] G. M. Sheldrick, *Acta Cryst. Sect. C Struct. Chem.* **2015**, *C71*, 3–8.

14. ^{1}H and ^{13}C NMR Spectra

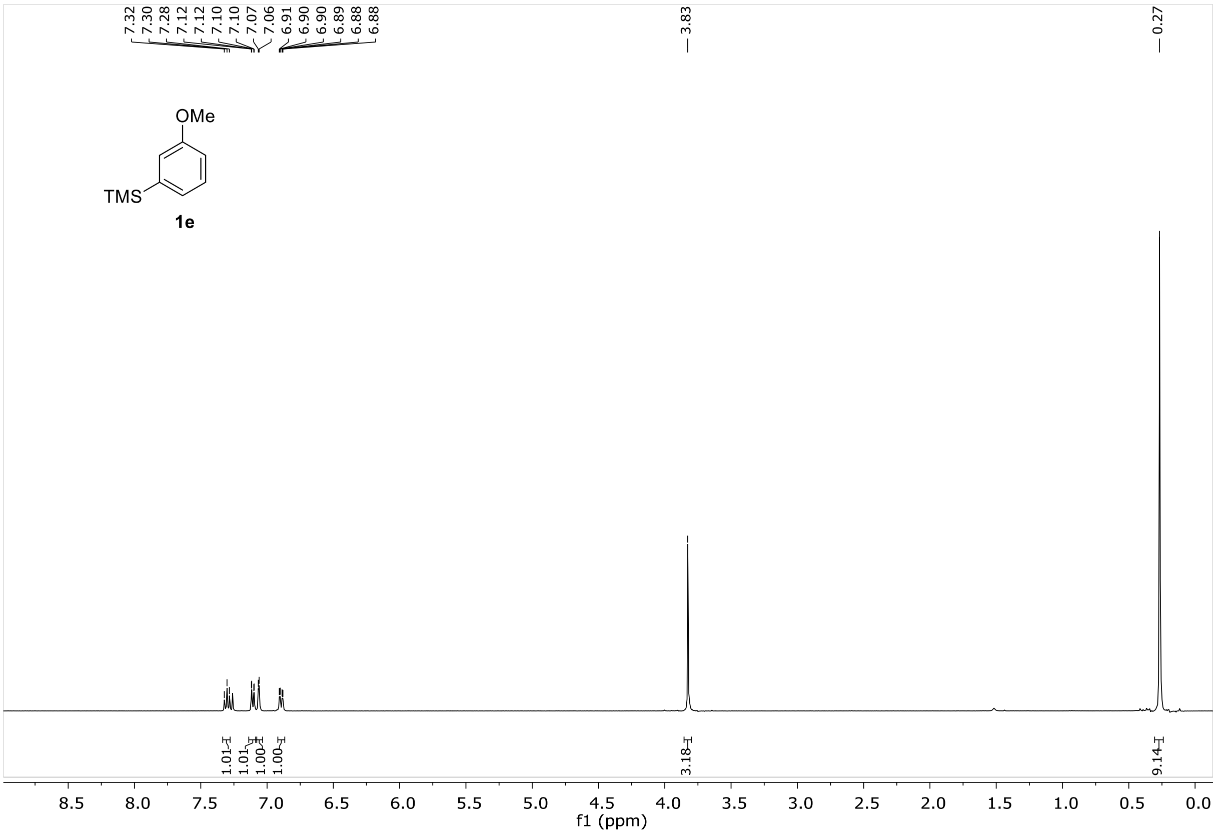

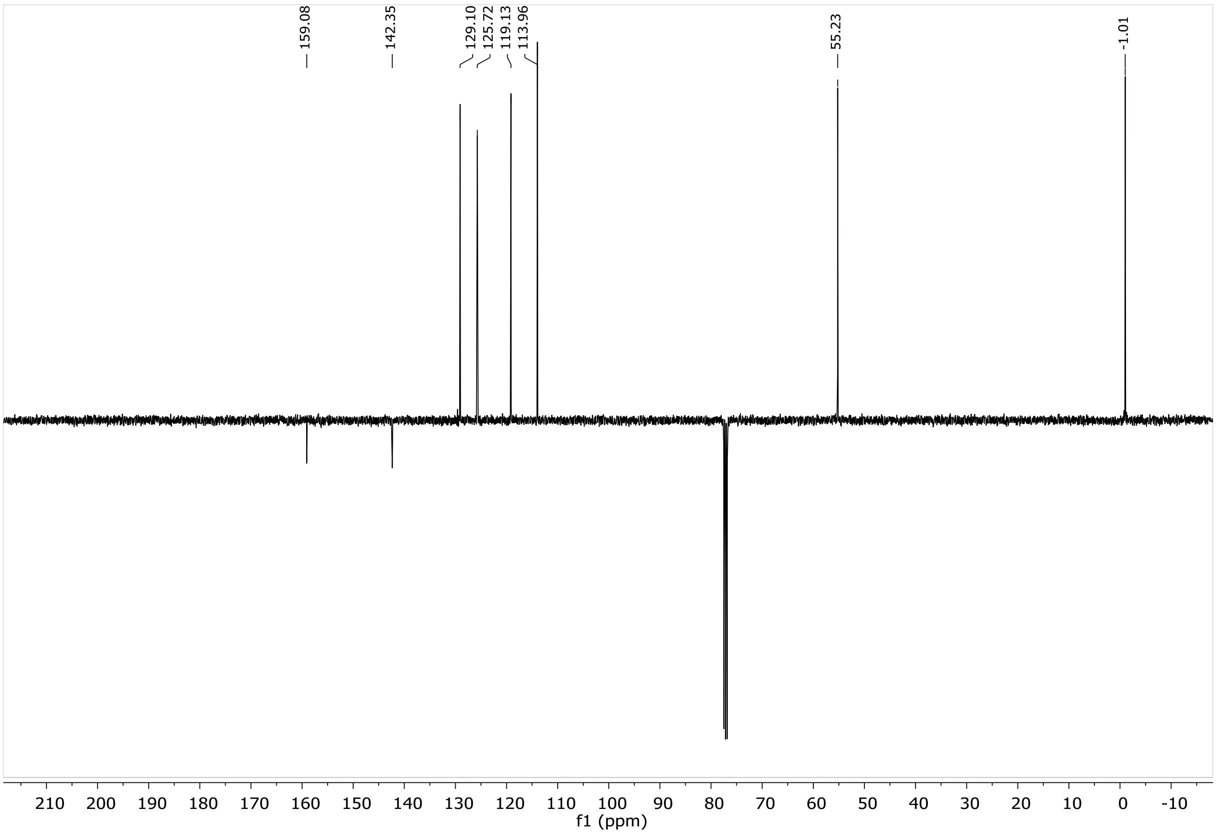

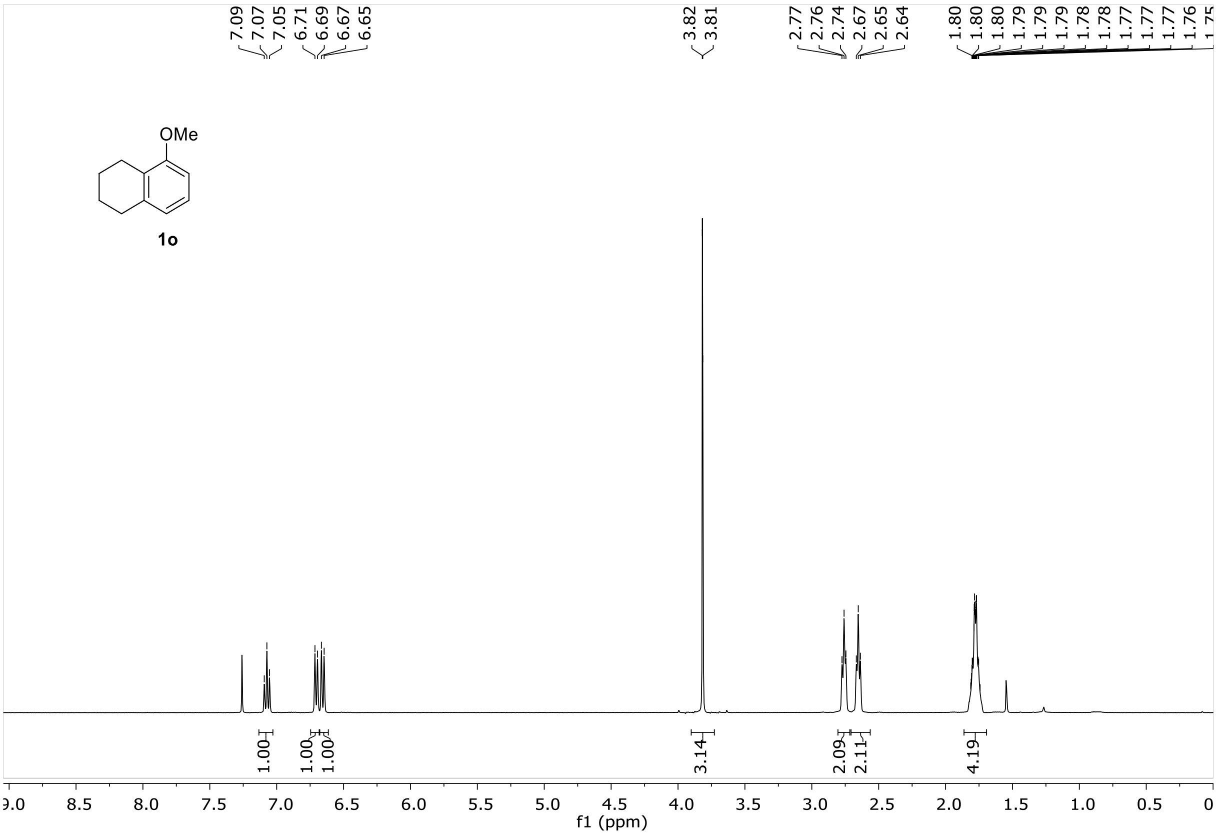
7.09
7.07
7.05
6.71
6.69
6.67
6.65
3.82
3.81
2.77
2.76
2.74
2.67
2.65
2.64
1.80
1.80
1.80
1.79
1.79
1.79
1.78
1.78
1.77
1.77
1.77
1.76
OMe
1o
1.00
1.00
1.00
3.14
2.09
2.11
4.19
8.5
8.0
7.5
7.0
6.5
6.0
5.5
5.0
4.5
4.0
3.5
3.0
2.5
2.0
1.5
1.0
0.5
f1 (ppm)

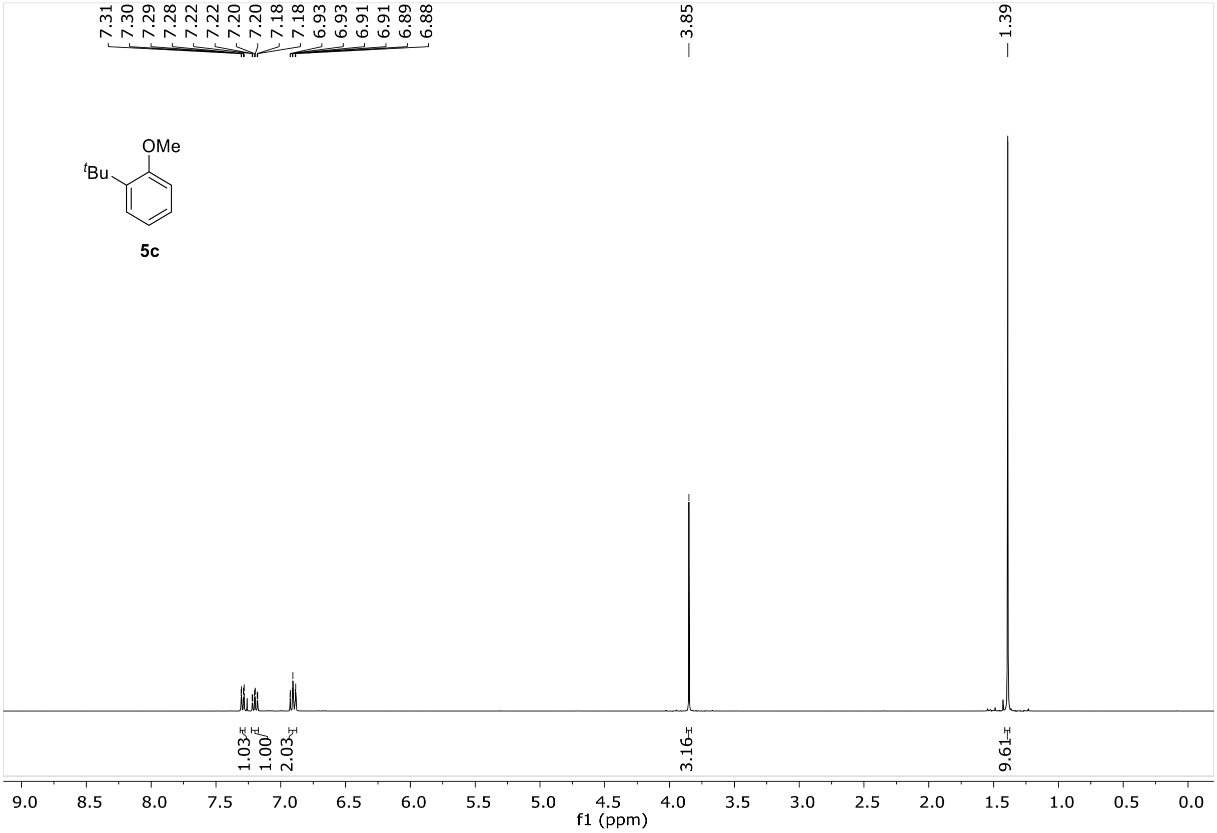
7.31
7.30
7.29
7.28
7.22
7.22
7.20
7.20
7.18
7.18
6.93
6.93
6.91
6.91
6.89
6.88
3.85
1.39
OMe
tBu
5c
1.03
1.00
2.03
3.16
9.61
9.0 8.5 8.0 7.5 7.0 6.5 6.0 5.5 5.0 4.5 4.0 3.5 3.0 2.5 2.0 1.5 1.0 0.5 0.0
f1 (ppm)

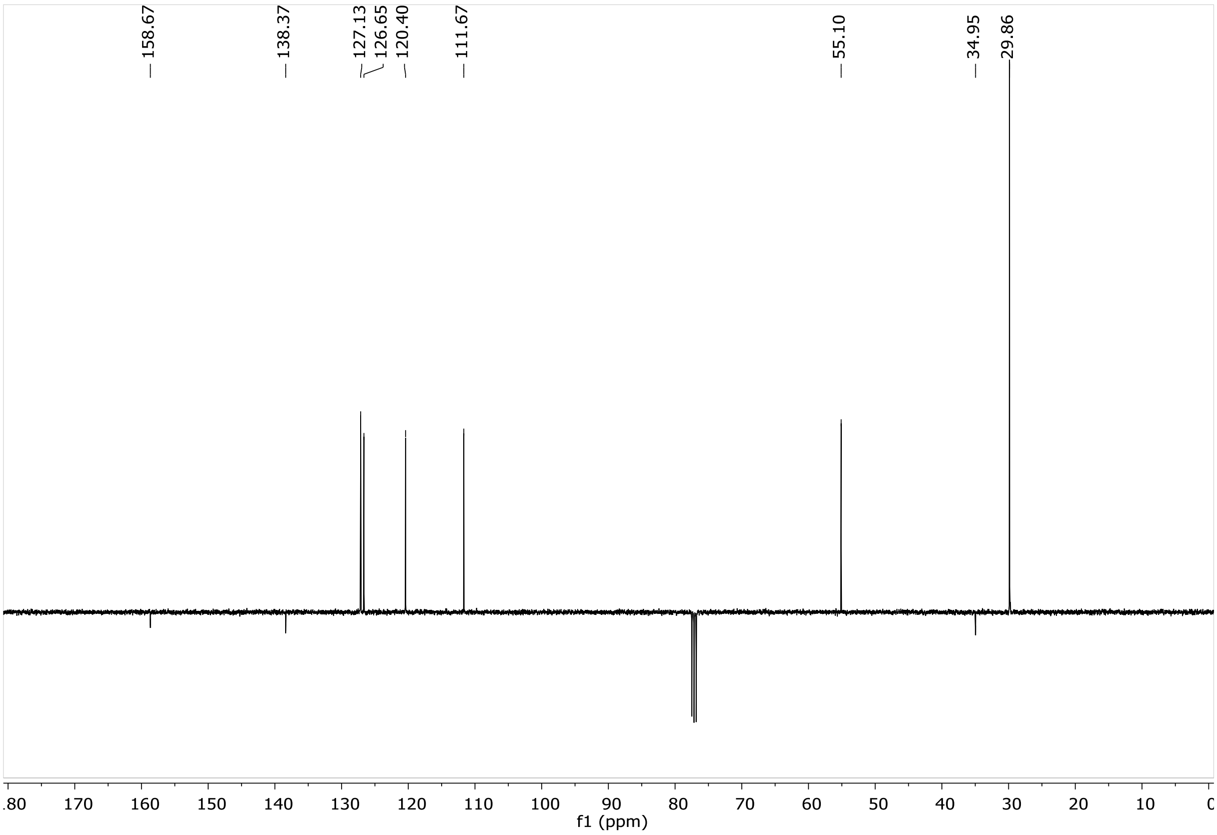
158.67
138.37
127.13
126.65
120.40
111.67
55.10
34.95
29.86
180 170 160 150 140 130 120 110 100 90 80 70 60 50 40 30 20 10 0
f1 (ppm)

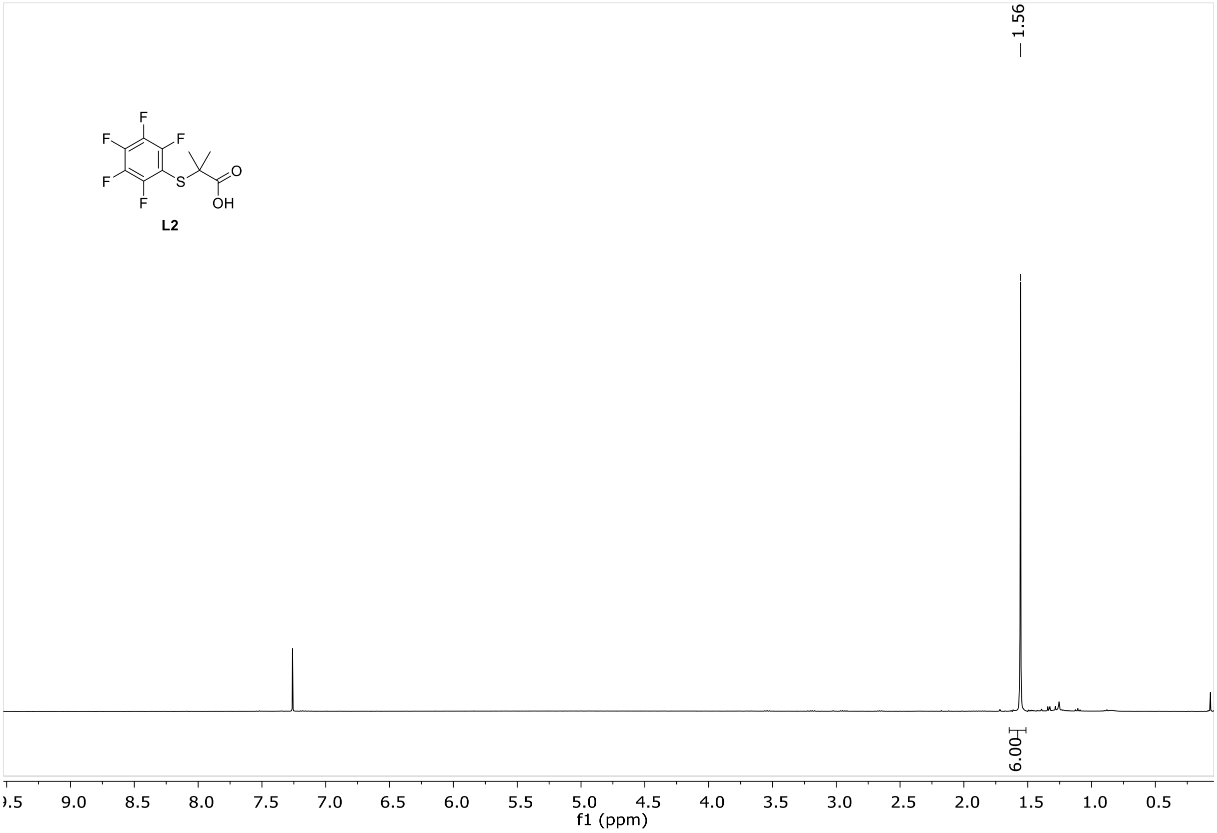
1.56
F
F
F
F
F
S
O
OH
L2
6.00
9.5 9.0 8.5 8.0 7.5 7.0 6.5 6.0 5.5 5.0 4.5 4.0 3.5 3.0 2.5 2.0 1.5 1.0 0.5
f1 (ppm)

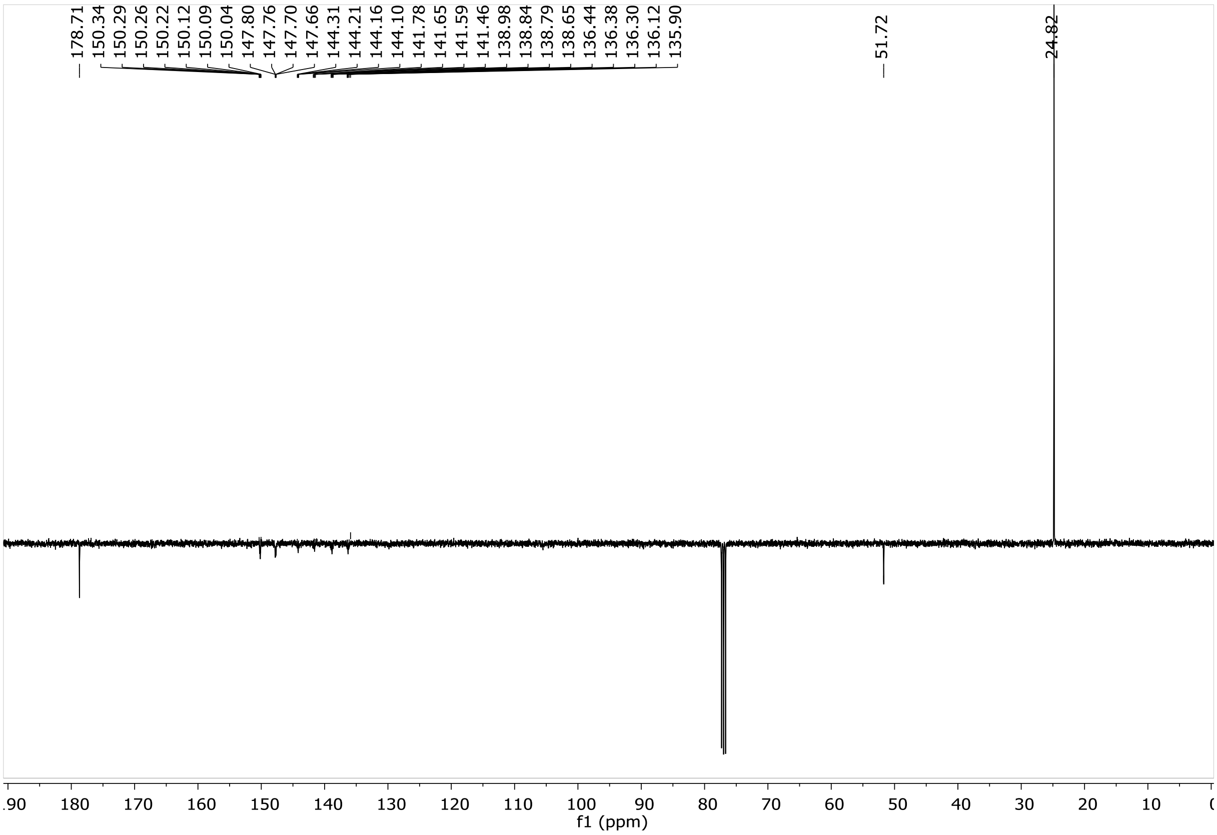
178.71
150.34
150.29
150.26
150.22
150.12
150.09
150.04
147.80
147.76
147.70
147.66
144.31
144.21
144.16
144.10
141.78
141.65
141.59
141.46
138.98
138.84
138.79
138.65
136.44
136.38
136.30
136.12
135.90
51.72
24.82
190 180 170 160 150 140 130 120 110 100 90 80 70 60 50 40 30 20 10 0
f1 (ppm)

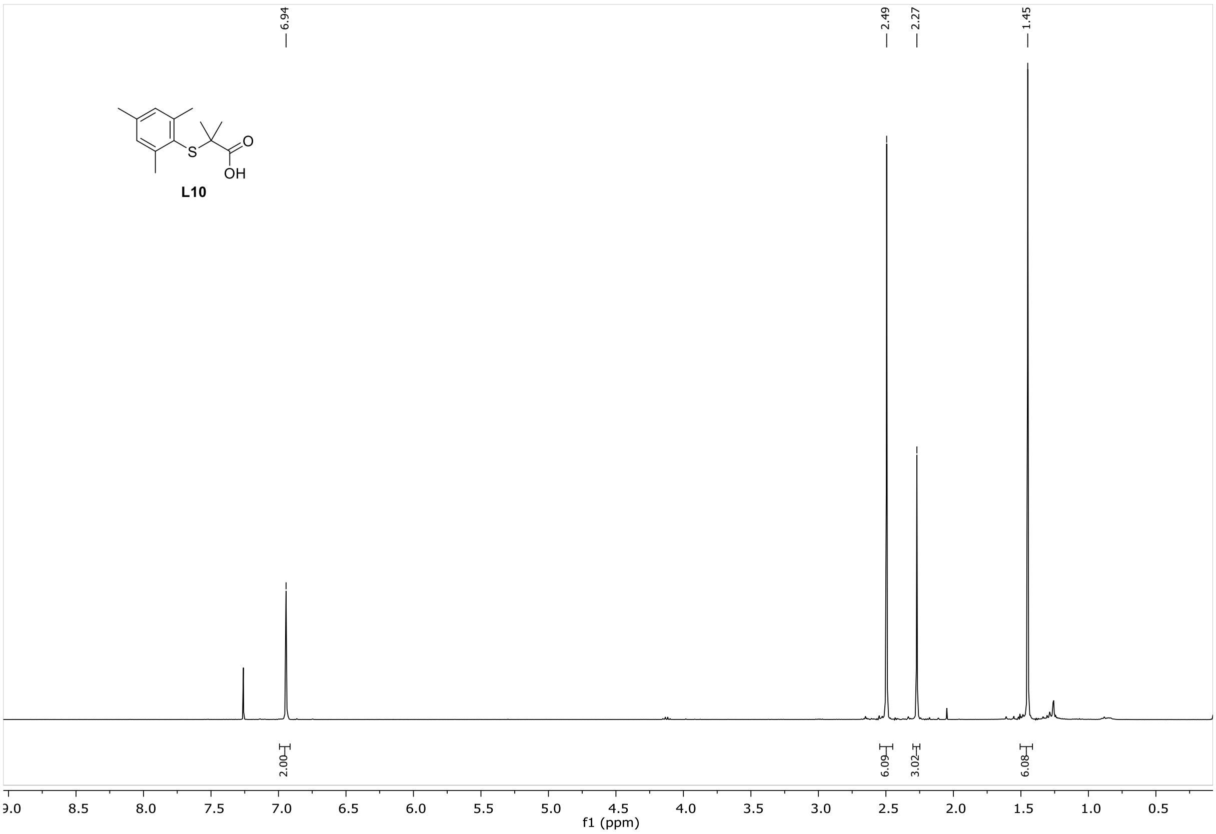
6.94
2.49
2.27
1.45
S
O
OH
L10
2.00
6.09
3.02
6.08
f1 (ppm)

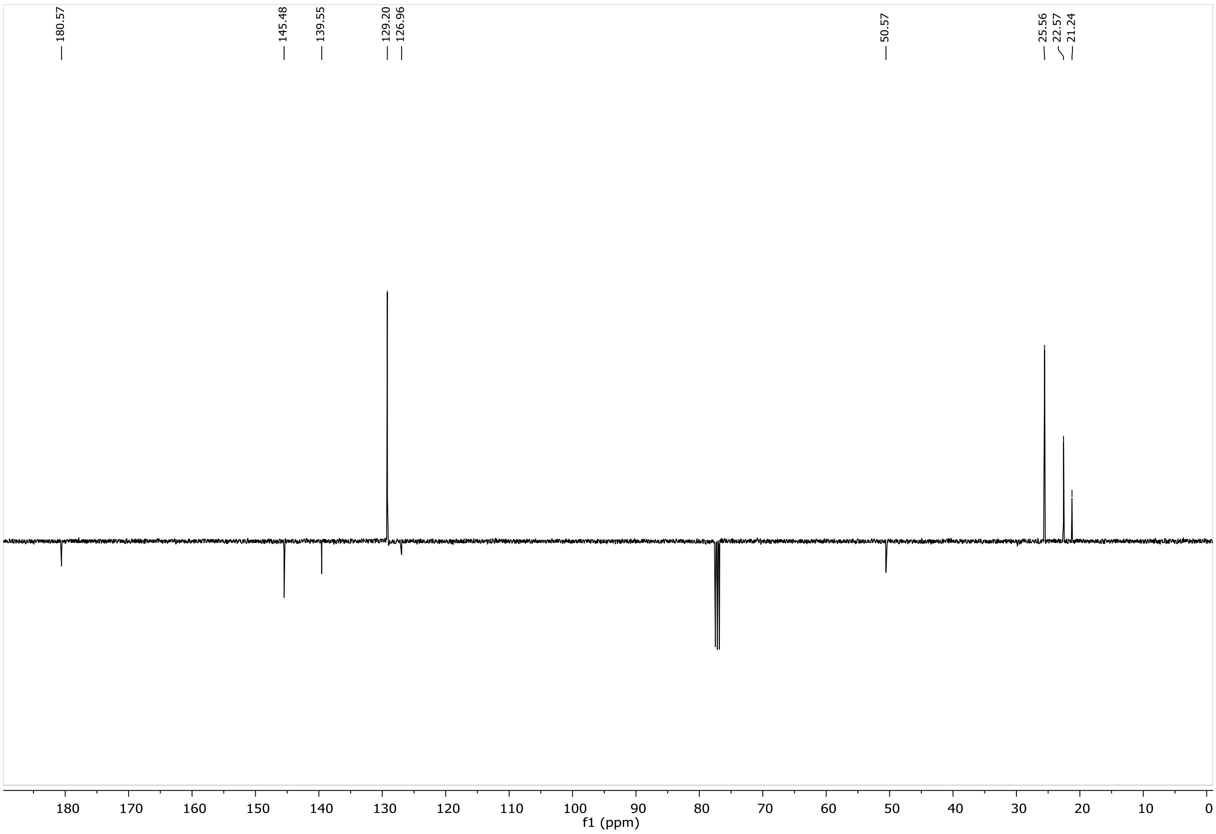
180.57
145.48
139.55
129.20
126.96
50.57
25.56
22.57
21.24
f1 (ppm)

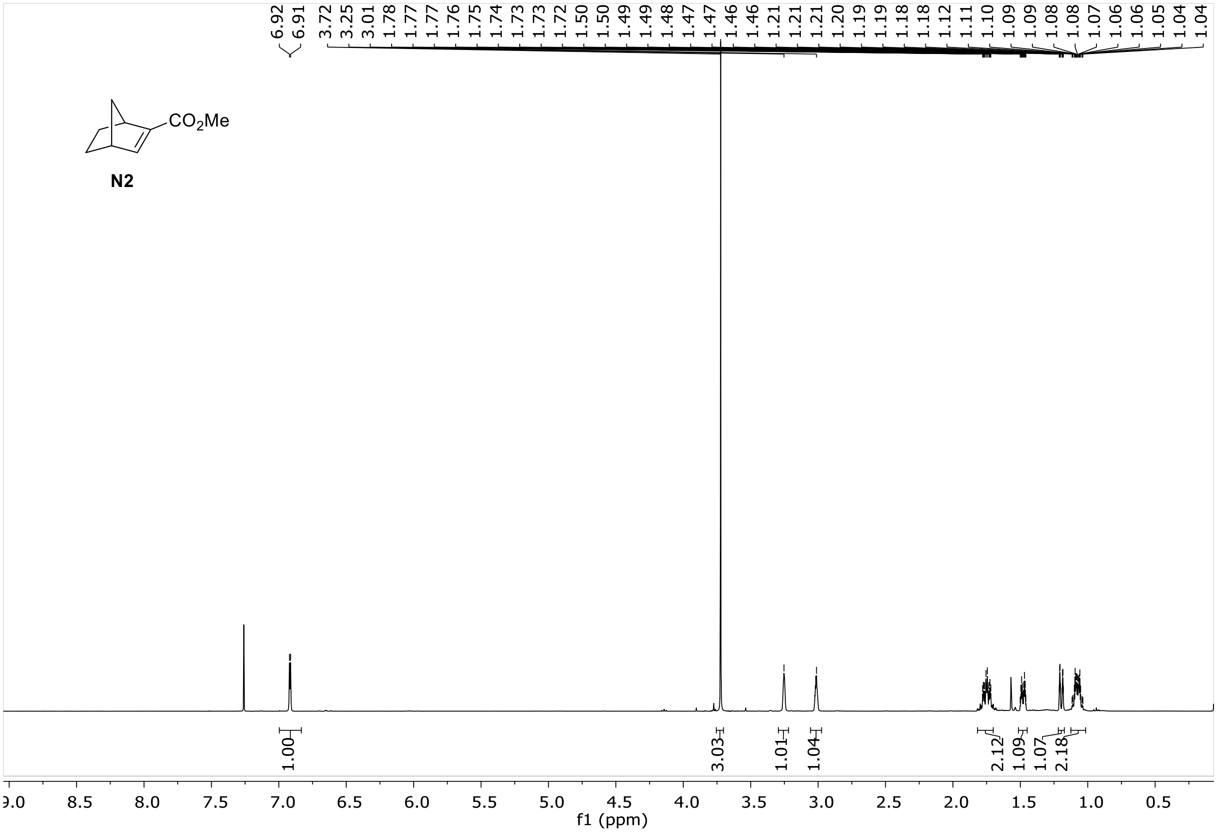
CO2Me
N2
6.92
6.91
3.72
3.25
3.01
1.78
1.77
1.77
1.76
1.75
1.74
1.73
1.73
1.72
1.50
1.50
1.49
1.49
1.48
1.47
1.46
1.46
1.21
1.21
1.21
1.20
1.19
1.19
1.18
1.18
1.12
1.11
1.10
1.09
1.09
1.08
1.08
1.07
1.06
1.06
1.05
1.04
1.04
1.00
3.03
1.01
1.04
2.12
1.09
1.07
2.18
9.0 8.5 8.0 7.5 7.0 6.5 6.0 5.5 5.0 4.5 4.0 3.5 3.0 2.5 2.0 1.5 1.0 0.5
f1 (ppm)

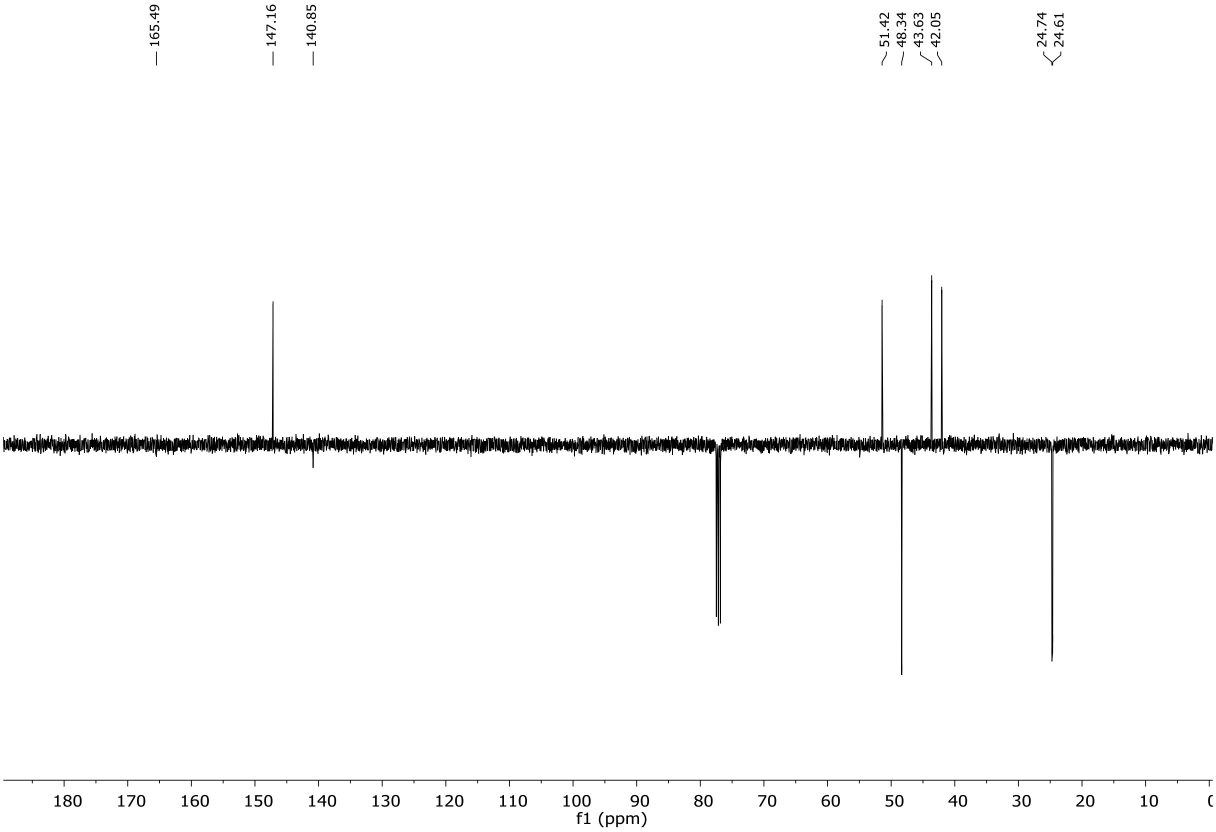
165.49
147.16
140.85
51.42
48.34
43.63
42.05
24.74
24.61
180 170 160 150 140 130 120 110 100 90 80 70 60 50 40 30 20 10 0
f1 (ppm)

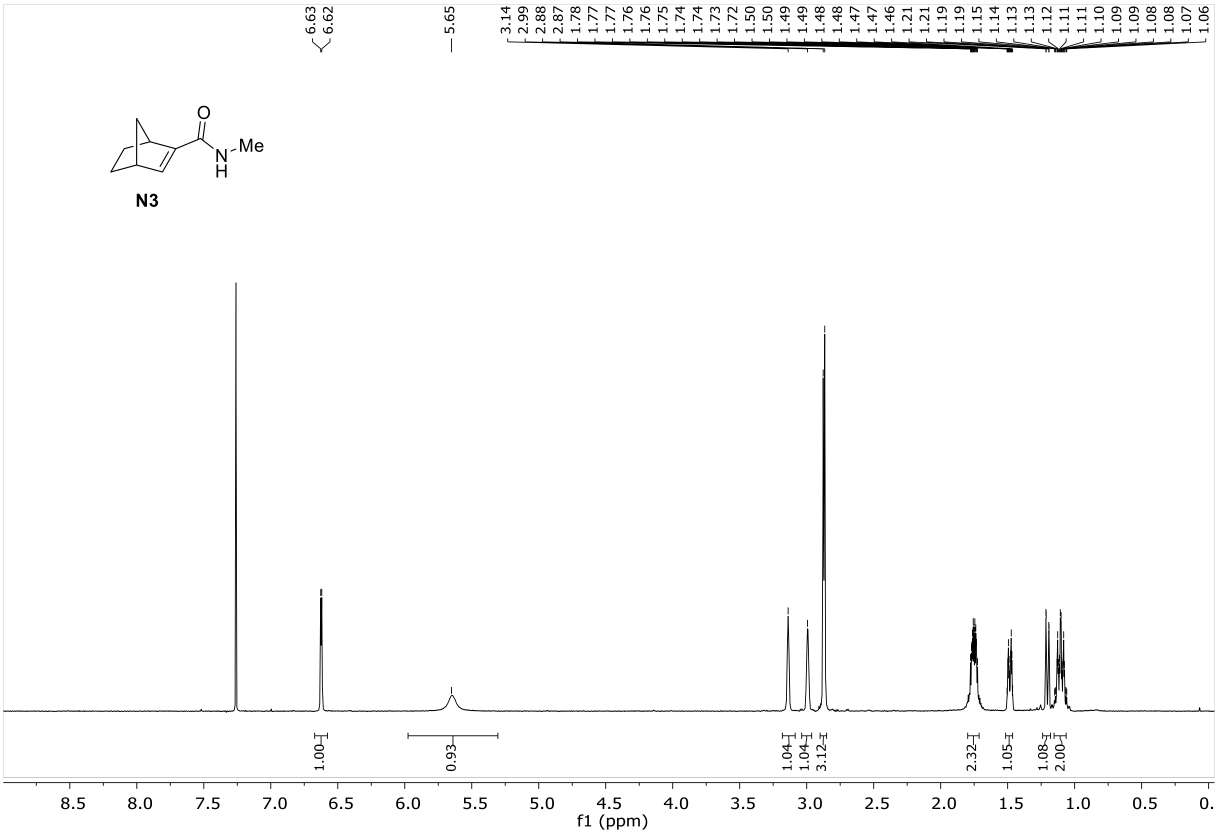
N3
6.63
6.62
5.65
3.14 2.99 2.88 2.87 1.78 1.77 1.77 1.76 1.76 1.75 1.74 1.74 1.73 1.72 1.50 1.49 1.49 1.48 1.48 1.47 1.47 1.46 1.21 1.21 1.19 1.19 1.15 1.14 1.13 1.13 1.12 1.11 1.11 1.10 1.09 1.09 1.08 1.08 1.07 1.06
1.00
0.93
1.04
1.04
3.12
2.32
1.05
1.08
2.00
f1 (ppm)

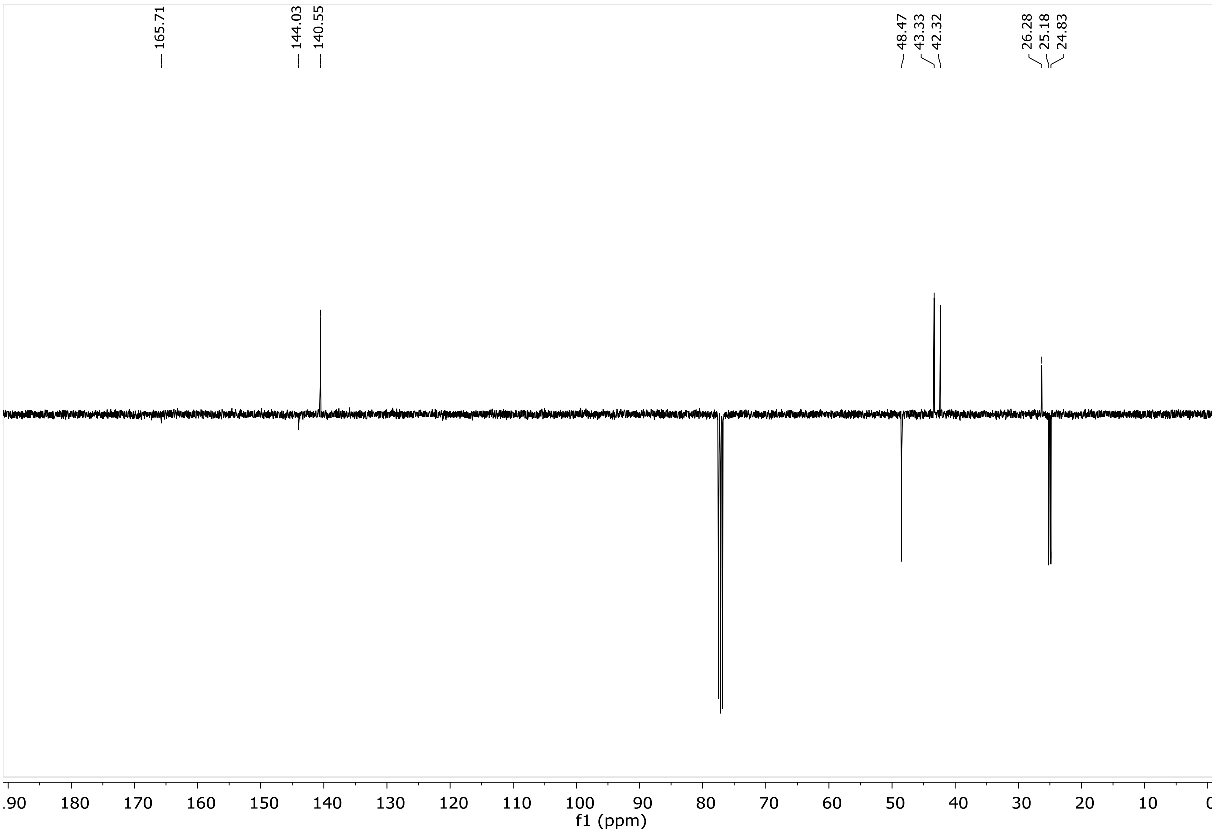
165.71
144.03
140.55
48.47
43.33
42.32
26.28
25.18
24.83
f1 (ppm)

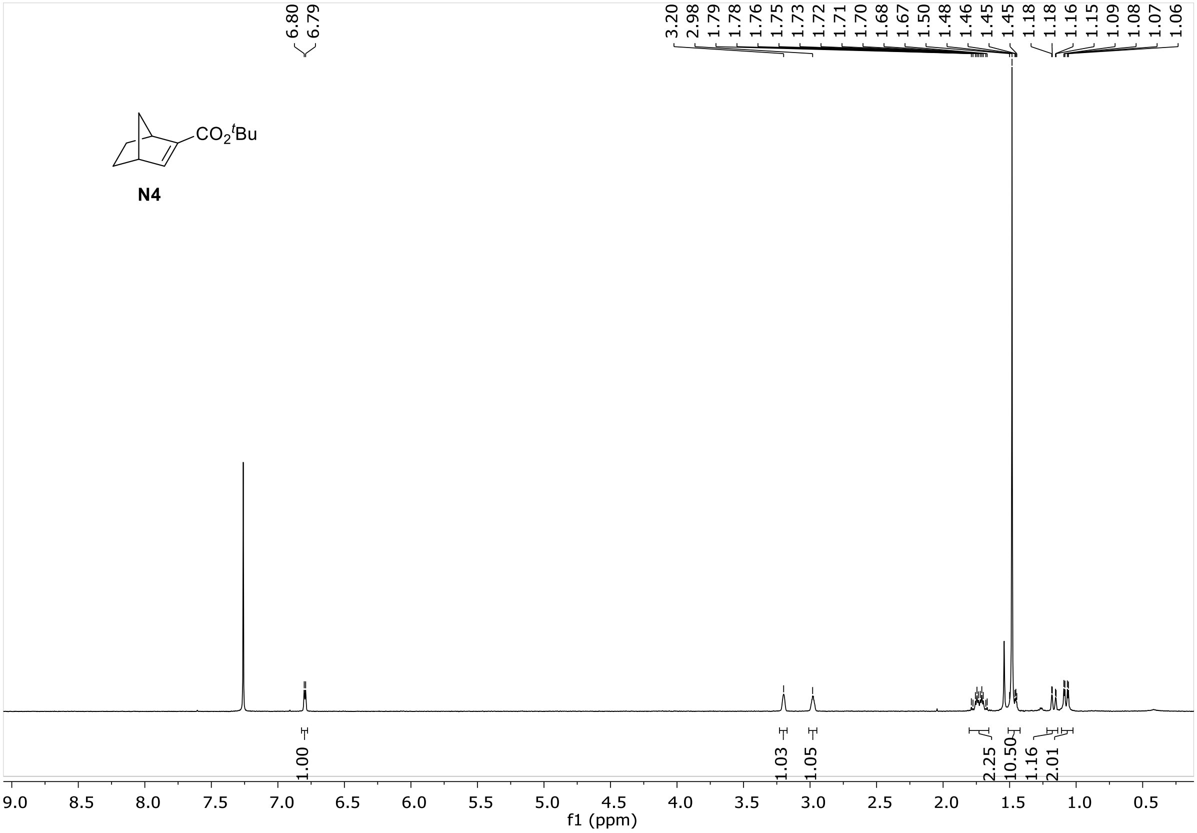
6.80
6.79
3.20
2.98
1.79
1.78
1.76
1.75
1.73
1.72
1.71
1.70
1.68
1.67
1.50
1.48
1.46
1.45
1.45
1.18
1.18
1.16
1.15
1.09
1.08
1.07
1.06
CO2tBu
N4
1.00
1.03
1.05
2.25
10.50
1.16
2.01
9.0 8.5 8.0 7.5 7.0 6.5 6.0 5.5 5.0 4.5 4.0 3.5 3.0 2.5 2.0 1.5 1.0 0.5
f1 (ppm)

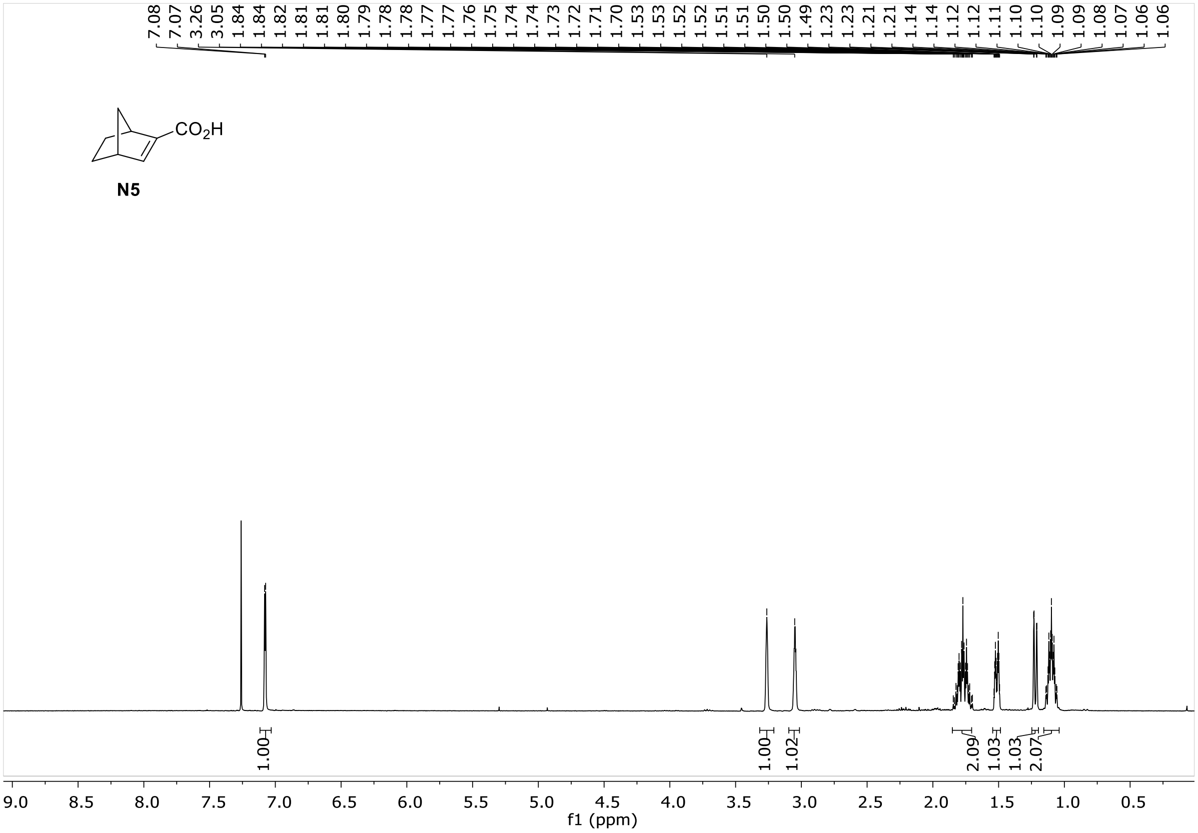
7.08
7.07
3.26
3.05
1.84
1.84
1.82
1.81
1.81
1.80
1.79
1.78
1.78
1.77
1.77
1.76
1.75
1.74
1.74
1.73
1.72
1.71
1.70
1.53
1.53
1.52
1.52
1.51
1.51
1.50
1.50
1.49
1.23
1.23
1.21
1.21
1.14
1.14
1.12
1.12
1.11
1.10
1.09
1.09
1.08
1.07
1.06
1.06
CO_2H
N5
1.00
1.00
1.02
2.09
1.03
1.03
2.07
9.0 8.5 8.0 7.5 7.0 6.5 6.0 5.5 5.0 4.5 4.0 3.5 3.0 2.5 2.0 1.5 1.0 0.5
f1 (ppm)

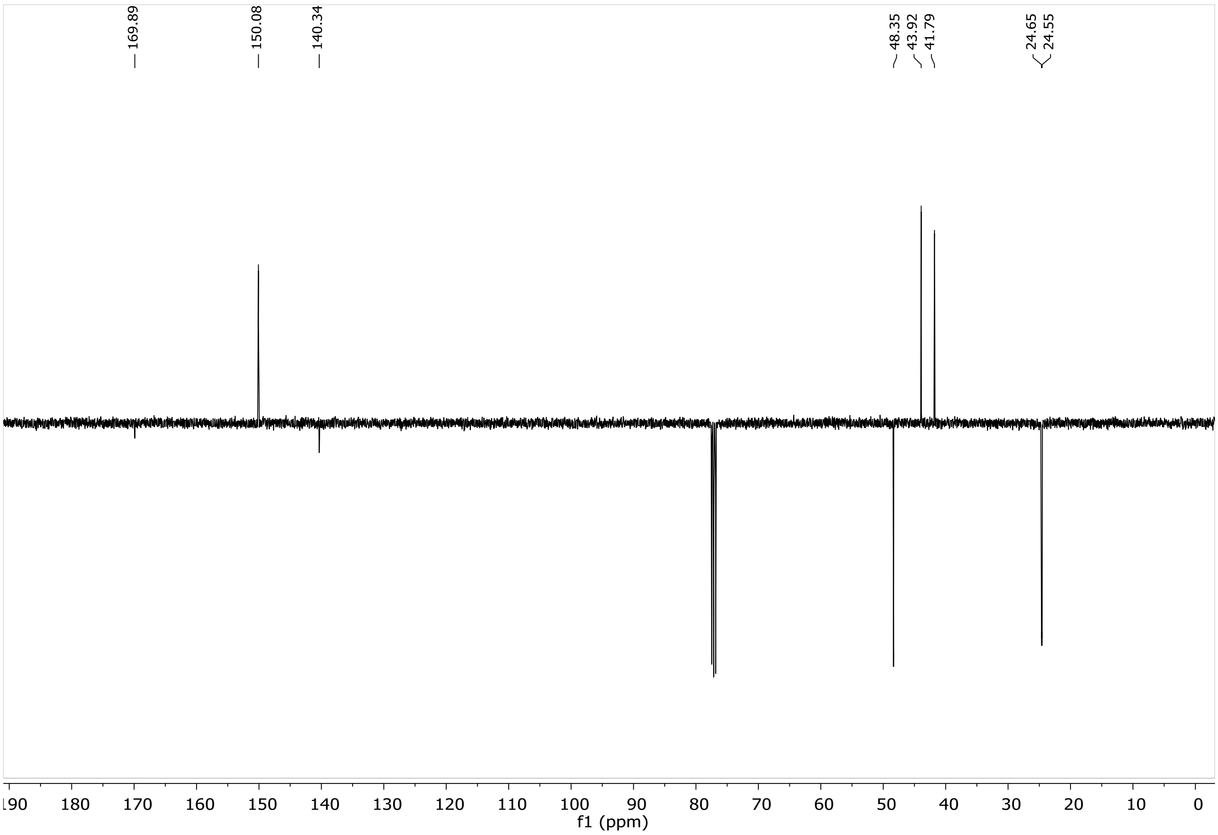
169.89
150.08
140.34
48.35
43.92
41.79
24.65
24.55
190 180 170 160 150 140 130 120 110 100 90 80 70 60 50 40 30 20 10 0
f1 (ppm)

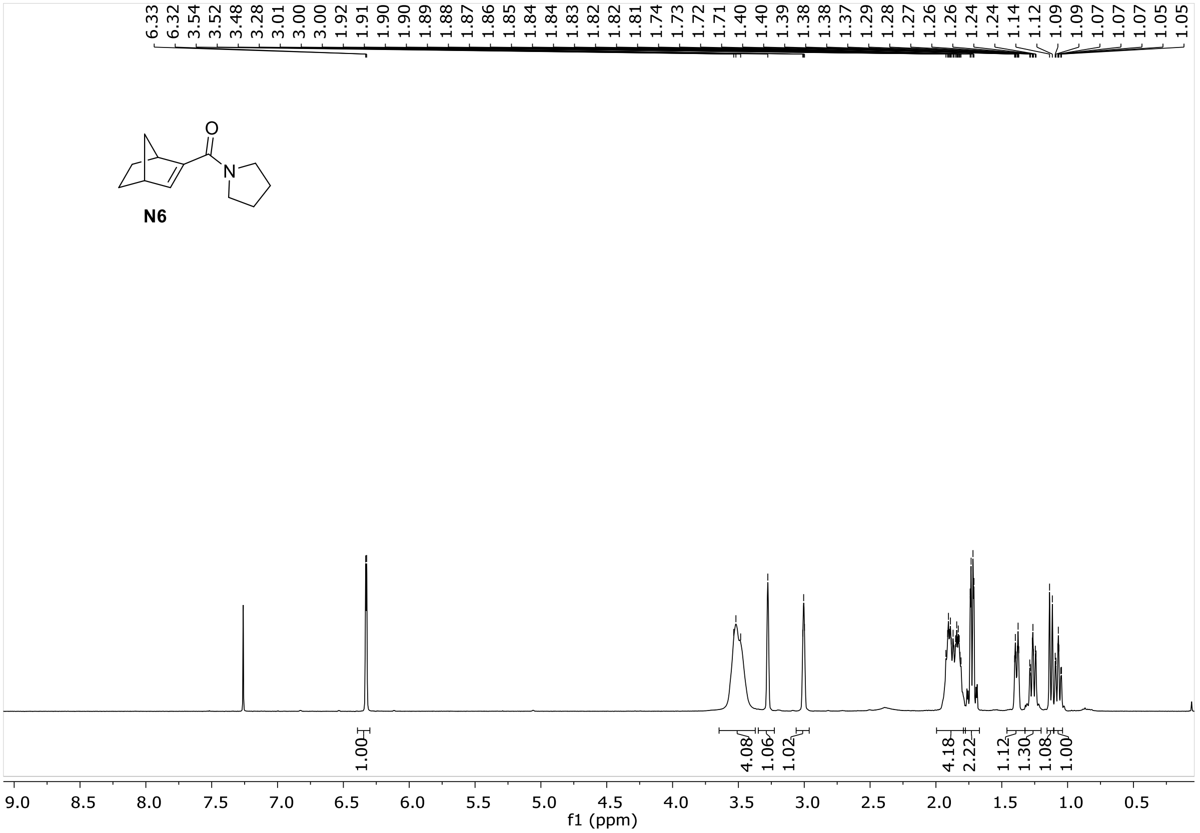
6.33
6.32
3.54
3.52
3.48
3.28
3.01
3.00
3.00
1.92
1.91
1.90
1.90
1.89
1.88
1.87
1.86
1.85
1.84
1.84
1.83
1.82
1.82
1.81
1.74
1.73
1.72
1.71
1.40
1.40
1.39
1.38
1.38
1.37
1.29
1.28
1.27
1.26
1.26
1.24
1.24
1.14
1.12
1.09
1.09
1.07
1.07
1.07
1.05
1.05
O
N
N6
1.00
4.08
1.06
1.02
4.18
2.22
1.12
1.30
1.08
1.00
9.0
8.5
8.0
7.5
7.0
6.5
6.0
5.5
5.0
4.5
4.0
3.5
3.0
2.5
2.0
1.5
1.0
0.5
f1 (ppm)

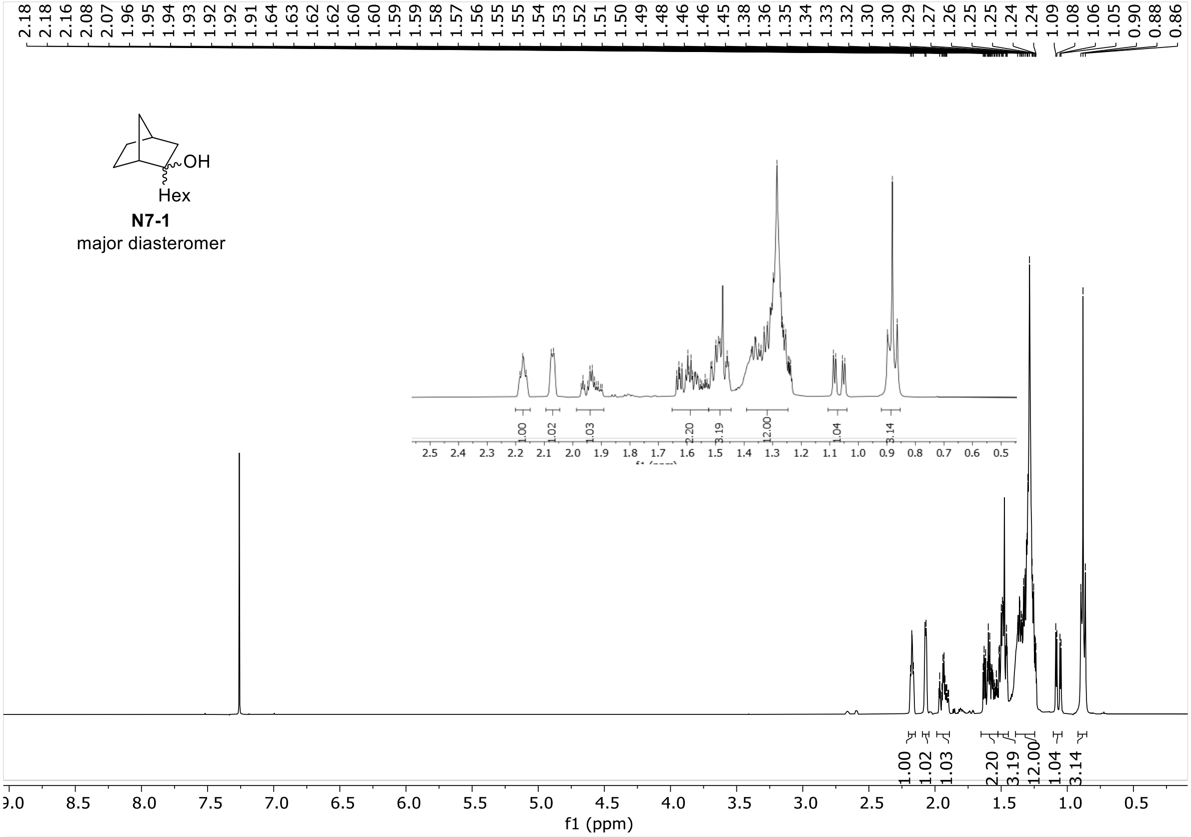
2.18
2.18
2.16
2.08
2.07
1.96
1.95
1.94
1.93
1.92
1.92
1.91
1.64
1.63
1.62
1.62
1.60
1.60
1.59
1.59
1.58
1.57
1.56
1.55
1.55
1.54
1.53
1.52
1.51
1.50
1.49
1.48
1.46
1.46
1.45
1.38
1.36
1.35
1.34
1.33
1.32
1.30
1.30
1.29
1.27
1.26
1.25
1.25
1.24
1.24
1.09
1.08
1.06
1.05
0.90
0.88
0.86
OH
Hex
N7-1
major diasteromer
1.00
1.02
1.03
2.20
3.19
12.00
1.04
3.14
f1 (ppm)

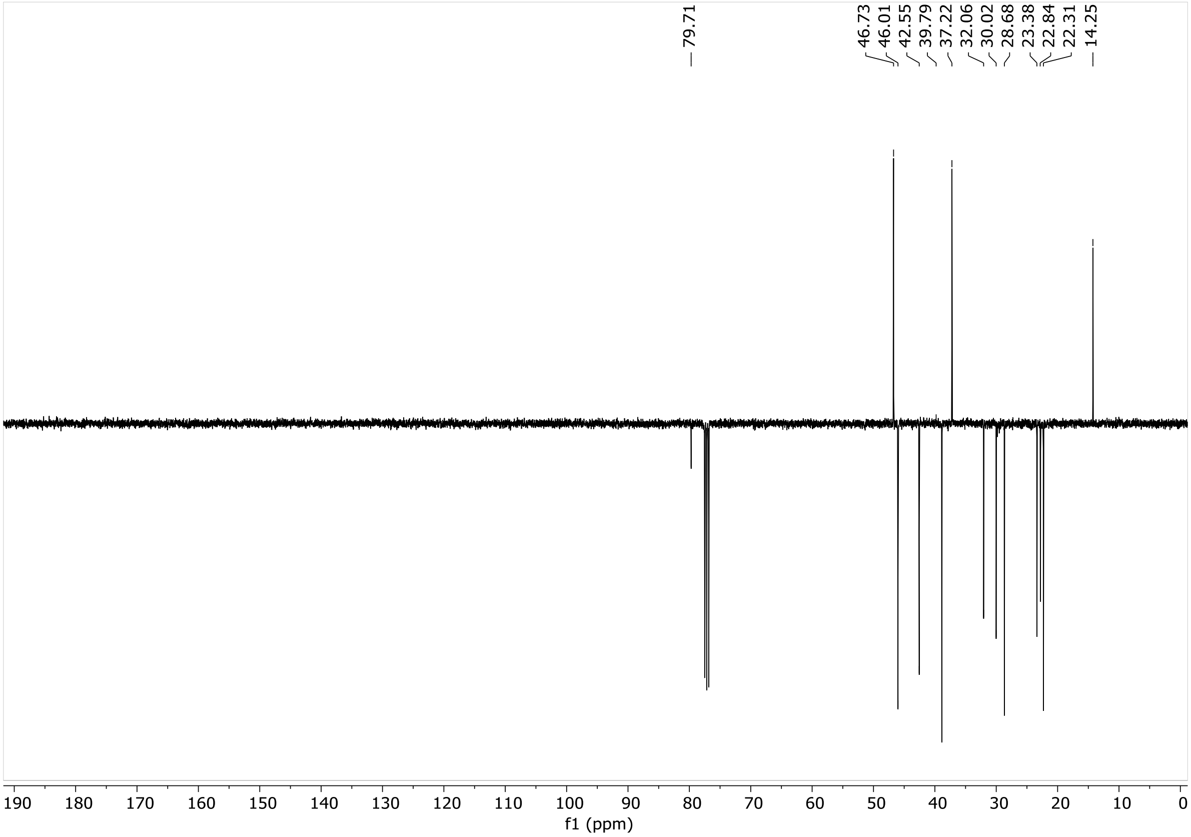
79.71
46.73
46.01
42.55
39.79
37.22
32.06
30.02
28.68
23.38
22.84
22.31
14.25
f1 (ppm)

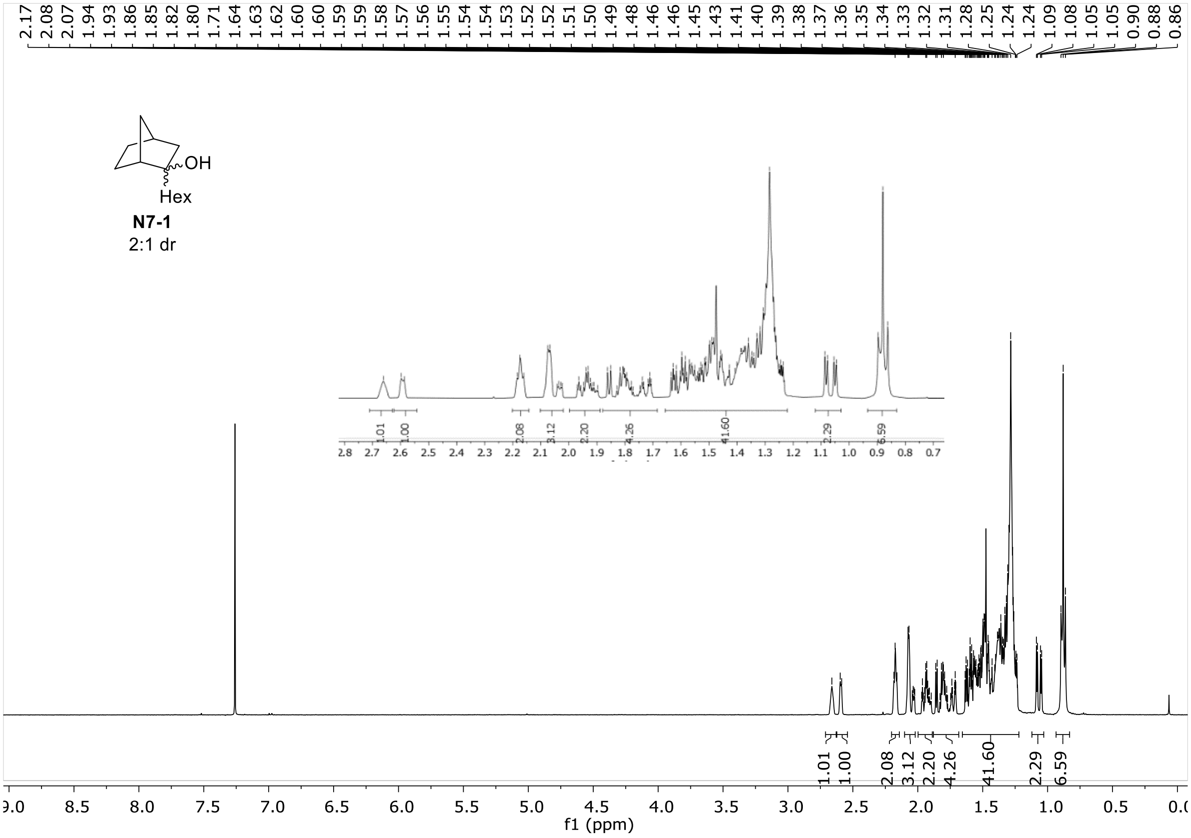
2.17
2.08
2.07
1.94
1.93
1.86
1.85
1.82
1.80
1.71
1.64
1.63
1.62
1.60
1.60
1.59
1.59
1.58
1.57
1.56
1.55
1.54
1.54
1.53
1.52
1.52
1.51
1.50
1.49
1.48
1.46
1.46
1.45
1.43
1.41
1.40
1.39
1.38
1.37
1.36
1.35
1.34
1.33
1.32
1.31
1.28
1.25
1.24
1.24
1.09
1.08
1.05
1.05
0.90
0.88
0.86
OH
Hex
N7-1
2:1 dr
1.01
1.00
2.08
3.12
2.20
4.26
41.60
2.29
6.59
f1 (ppm)

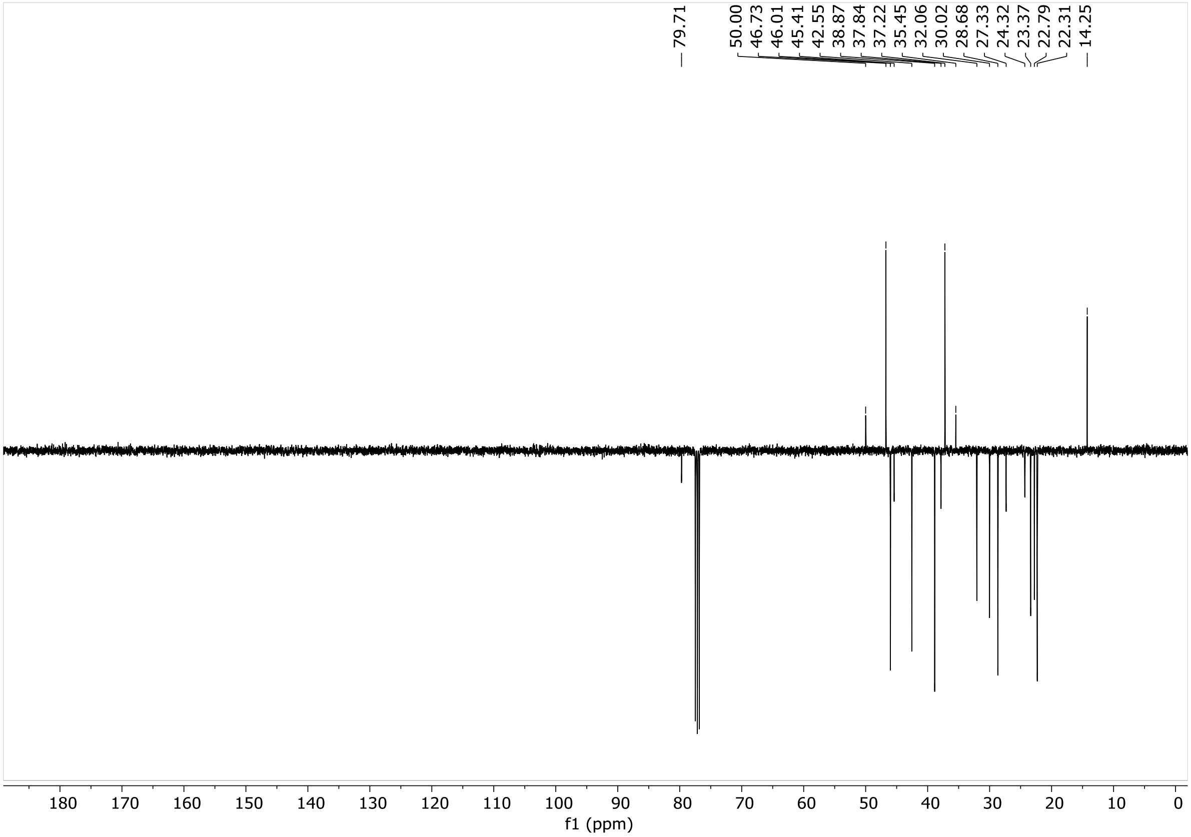
79.71
50.00
46.73
46.01
45.41
42.55
38.87
37.84
37.22
35.45
32.06
30.02
28.68
27.33
24.32
23.37
22.79
22.31
14.25
f1 (ppm)

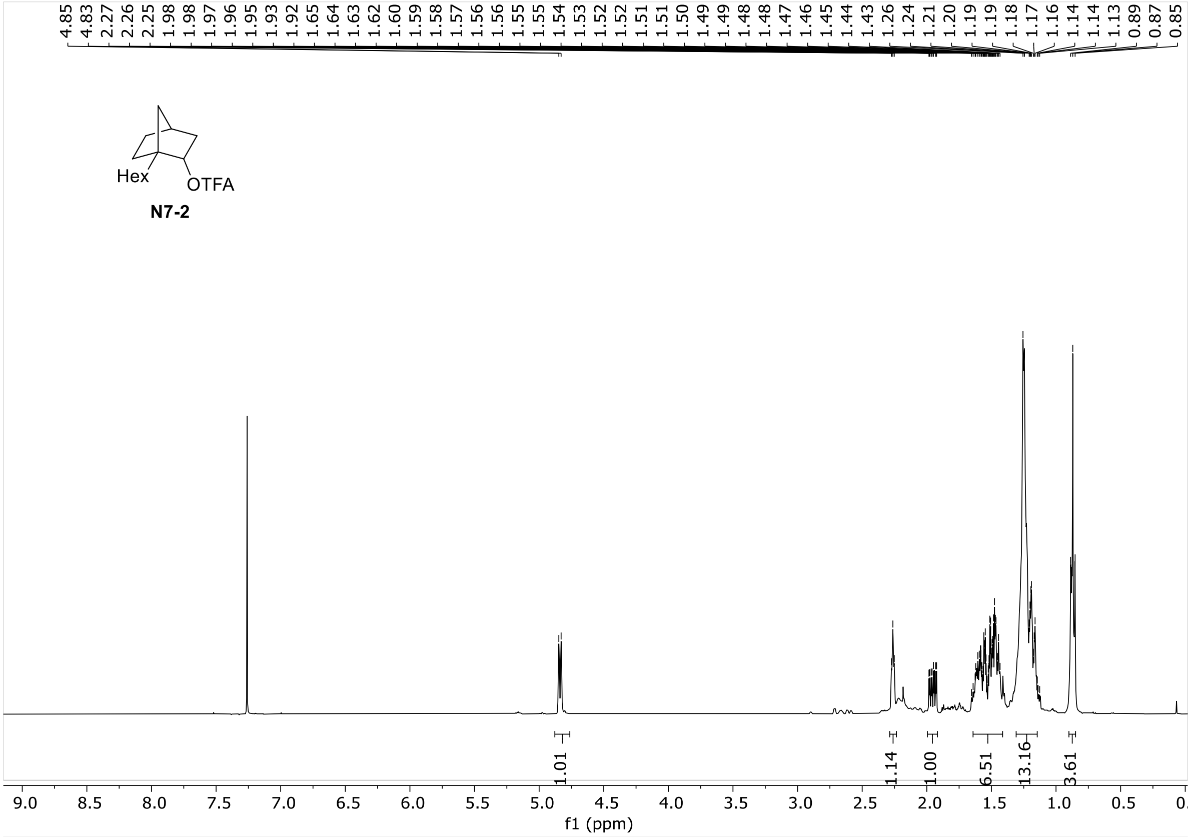
Hex
OTFA
N7-2
f1 (ppm)
1.01
1.14
1.00
6.51
13.16
3.61

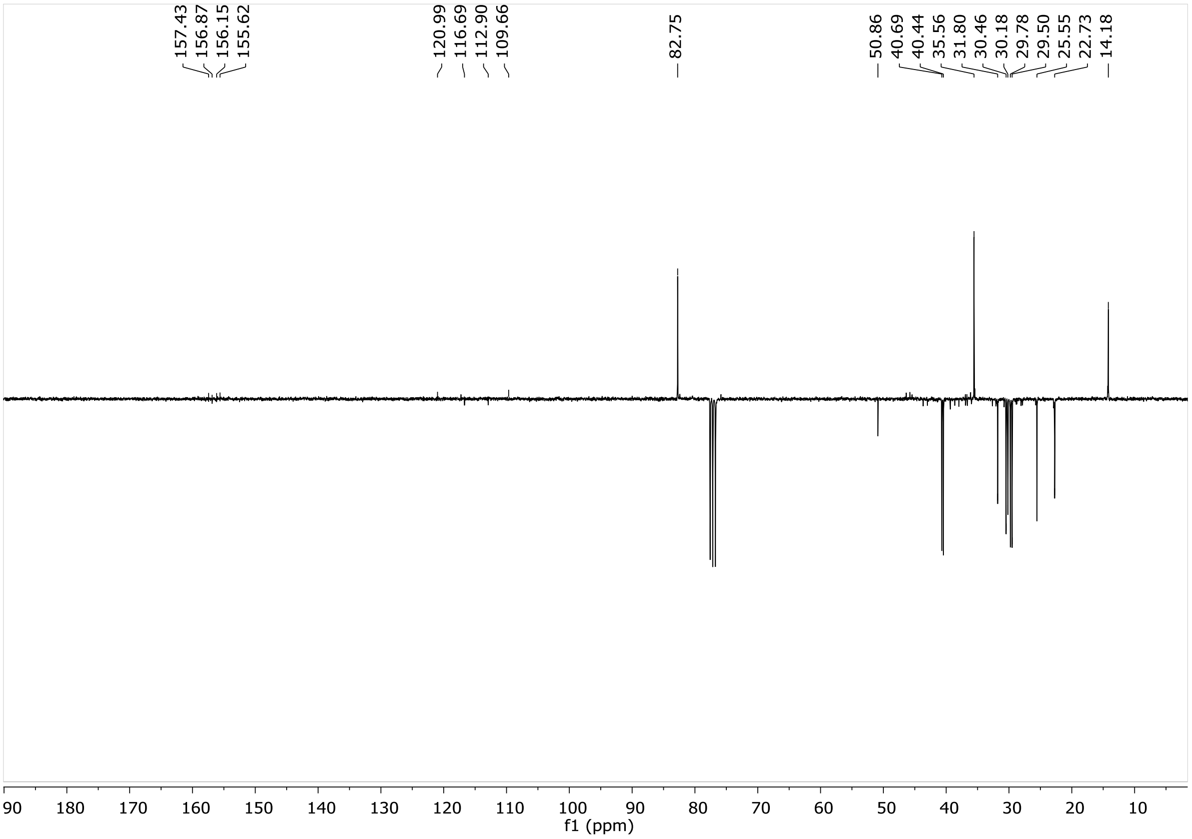
157.43
156.87
156.15
155.62
120.99
116.69
112.90
109.66
82.75
50.86
40.69
40.44
35.56
31.80
30.46
30.18
29.78
29.50
25.55
22.73
14.18
f1 (ppm)

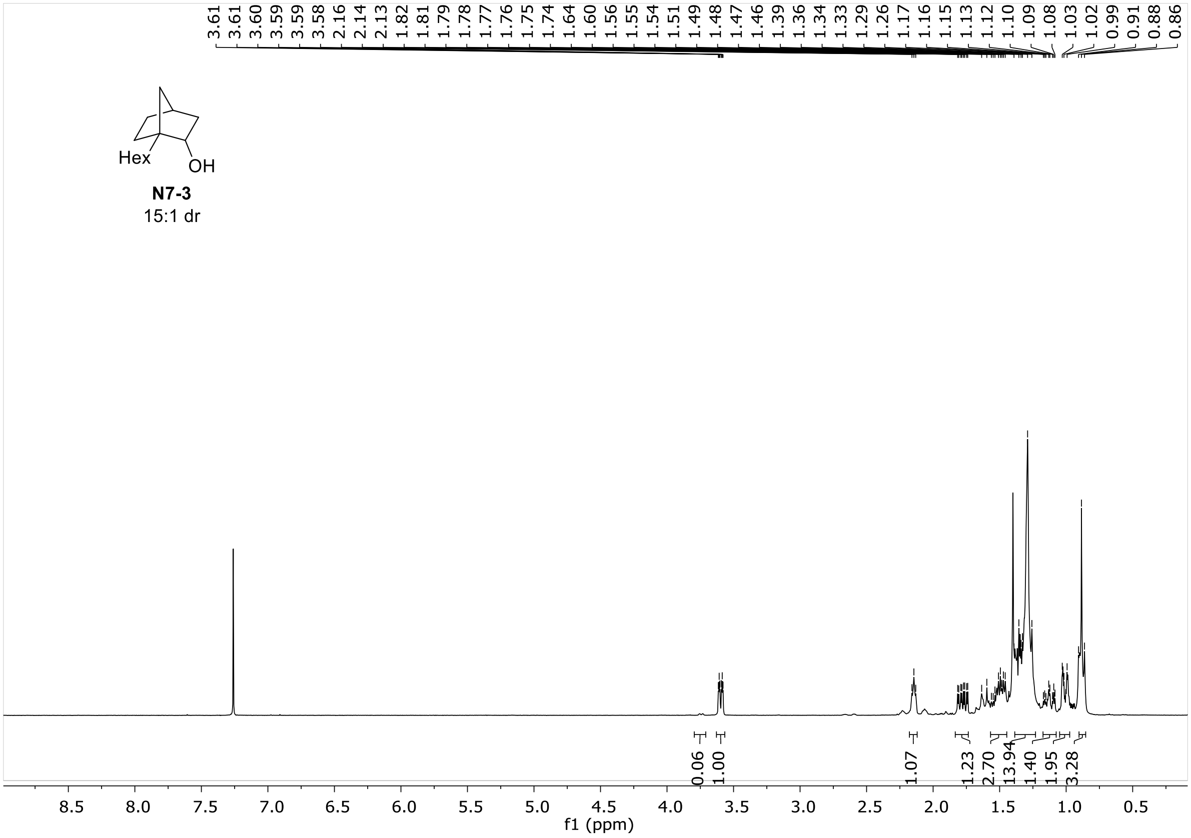
3.61
3.61
3.60
3.59
3.59
3.58
2.16
2.14
2.13
1.82
1.81
1.79
1.78
1.77
1.76
1.75
1.74
1.64
1.60
1.56
1.55
1.54
1.51
1.49
1.48
1.47
1.46
1.39
1.36
1.34
1.33
1.29
1.26
1.17
1.16
1.15
1.13
1.12
1.10
1.09
1.08
1.03
1.02
0.99
0.91
0.88
0.86
Hex
OH
N7-3
15:1 dr
0.06
1.00
1.07
1.23
2.70
13.94
1.40
1.95
3.28
f1 (ppm)

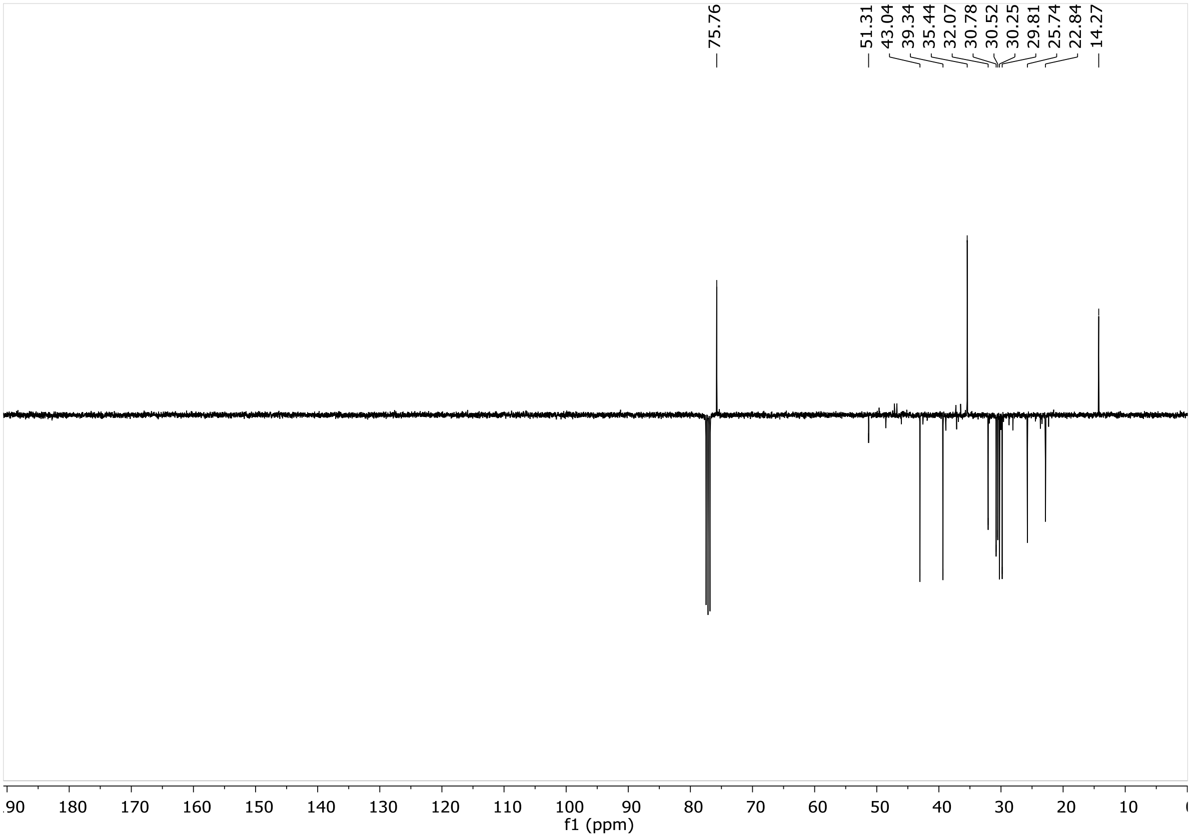
75.76
51.31
43.04
39.34
35.44
32.07
30.78
30.52
30.25
29.81
25.74
22.84
14.27
f1 (ppm)

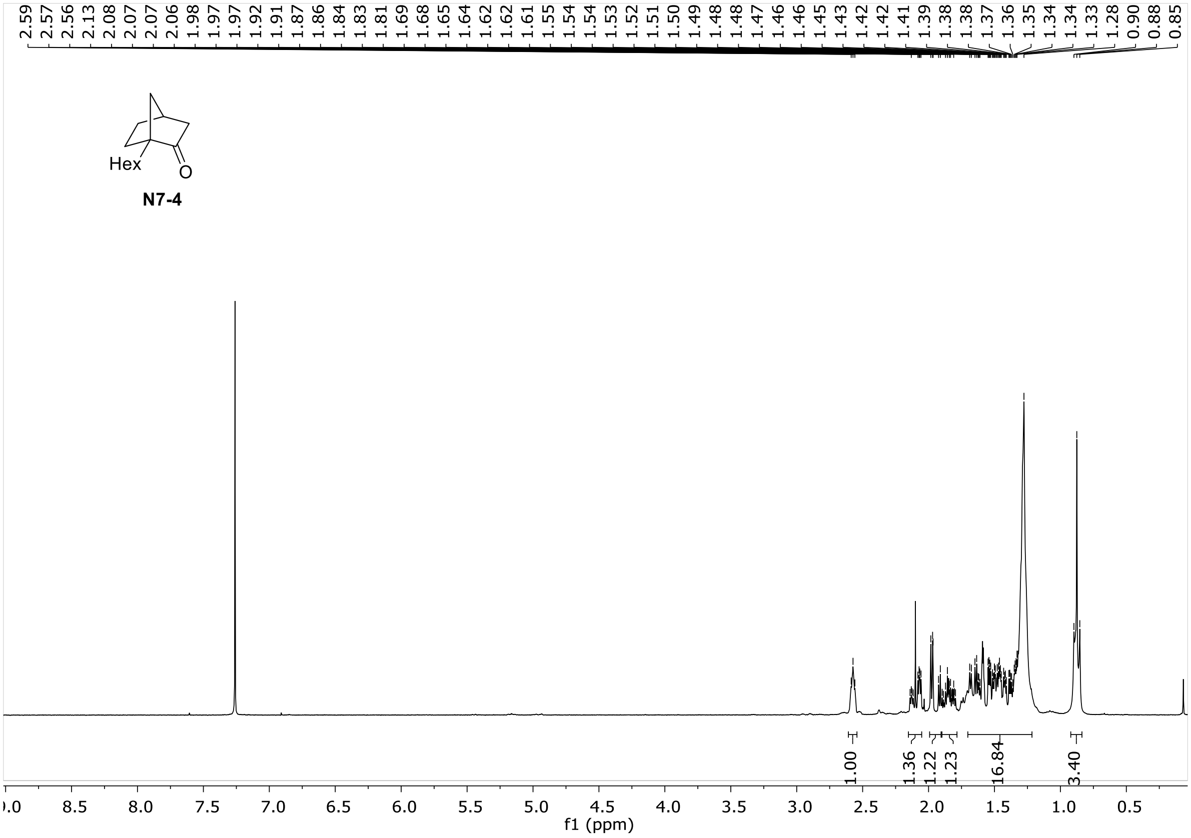
2.59
2.57
2.56
2.13
2.08
2.07
2.07
2.06
1.98
1.97
1.97
1.92
1.91
1.87
1.86
1.84
1.83
1.81
1.69
1.68
1.65
1.64
1.62
1.62
1.61
1.55
1.54
1.54
1.53
1.52
1.51
1.50
1.49
1.48
1.48
1.47
1.46
1.46
1.45
1.43
1.42
1.42
1.41
1.39
1.38
1.38
1.37
1.36
1.35
1.34
1.34
1.33
1.28
0.90
0.88
0.85
Hex
O
N7-4
1.00
1.36
1.22
1.23
16.84
3.40
8.5
8.0
7.5
7.0
6.5
6.0
5.5
5.0
4.5
4.0
3.5
3.0
2.5
2.0
1.5
1.0
0.5
f1 (ppm)

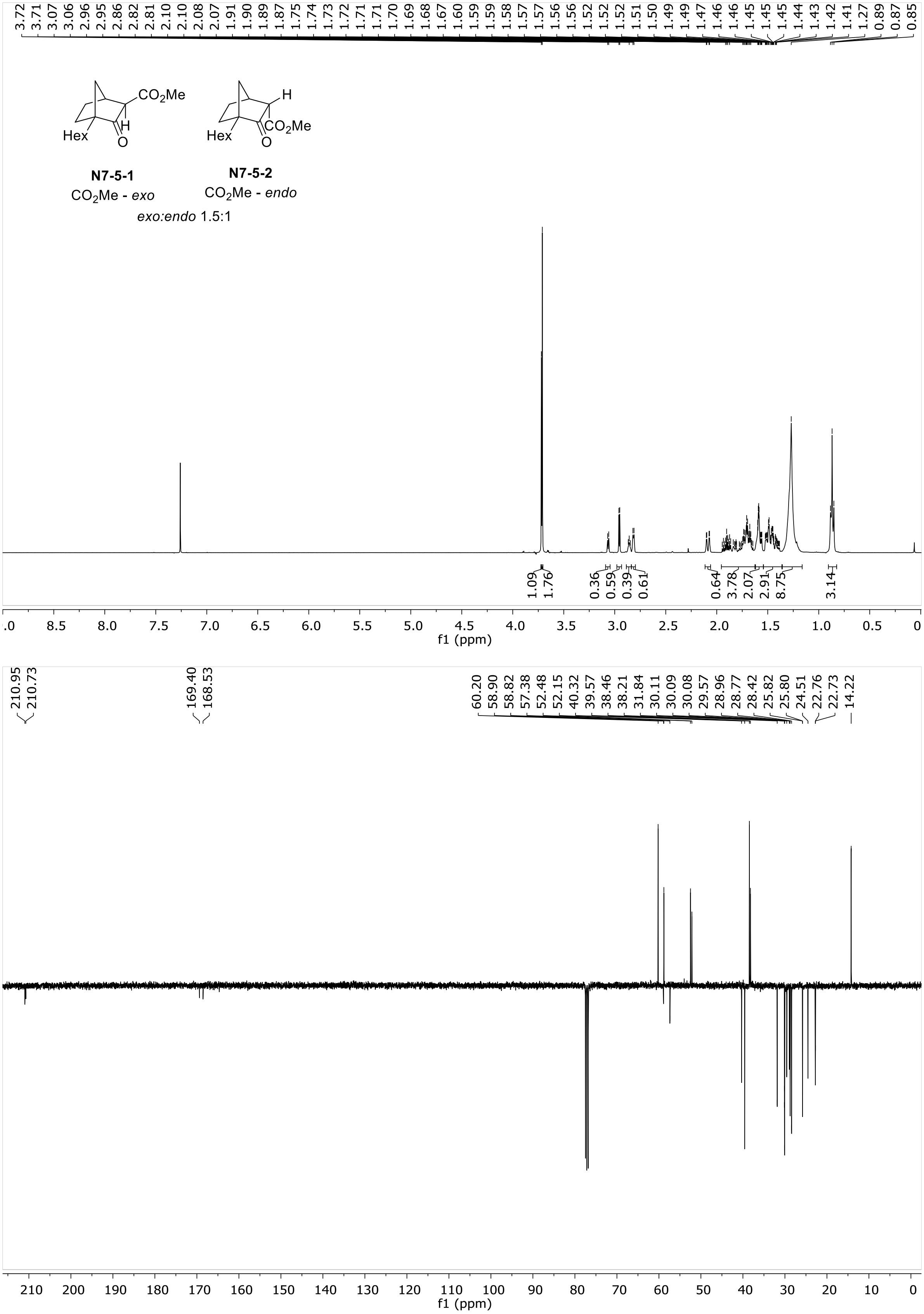
CO2Me
Hex
H
O
H
Hex
CO2Me
O
N7-5-1
CO2Me - exo
N7-5-2
CO2Me - endo
exo:endo 1.5:1
f1 (ppm)
f1 (ppm)

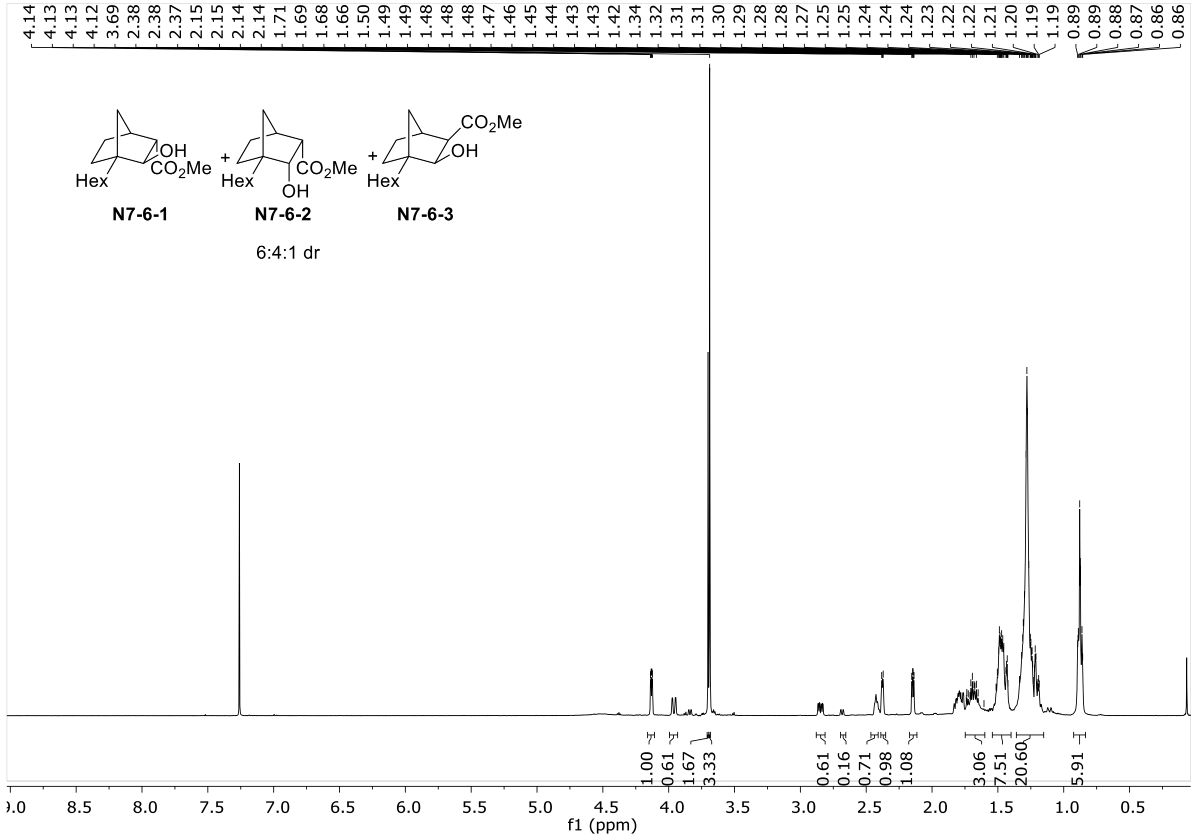
4.14 4.13 4.13 4.12 3.69 2.38 2.38 2.37 2.15 2.15 2.14 2.14 1.71 1.69 1.68 1.66 1.50 1.49 1.49 1.48 1.48 1.48 1.47 1.46 1.45 1.44 1.43 1.43 1.42 1.34 1.32 1.31 1.31 1.30 1.29 1.28 1.28 1.27 1.25 1.25 1.24 1.24 1.24 1.23 1.22 1.22 1.21 1.20 1.19 1.19 0.89 0.89 0.88 0.87 0.86 0.86
OH
CO2Me
Hex
+
CO2Me
Hex
OH
+
CO2Me
OH
Hex
N7-6-1
N7-6-2
N7-6-3
6:4:1 dr
1.00 0.61 1.67 3.33 0.61 0.16 0.71 0.98 1.08 3.06 7.51 20.60 5.91
f1 (ppm)

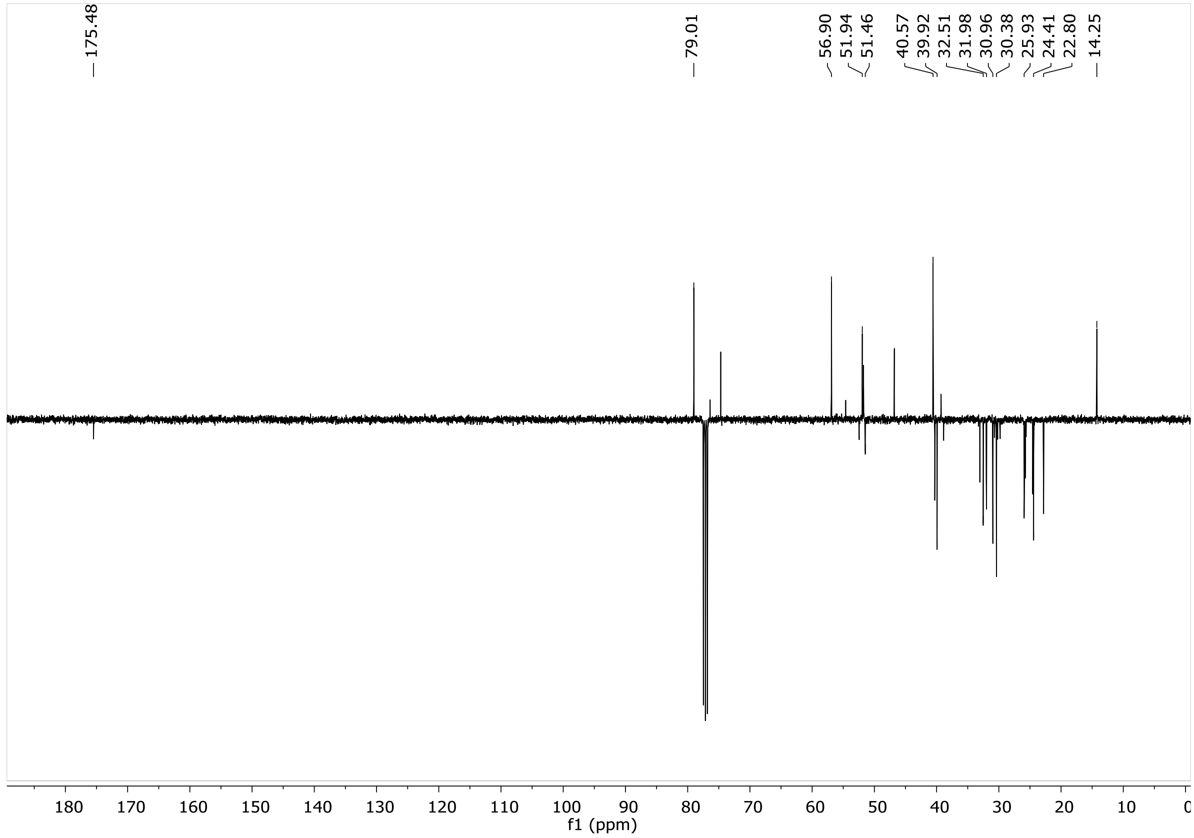
175.48
79.01
56.90 51.94 51.46 40.57 39.92 32.51 31.98 30.96 30.38 25.93 24.41 22.80 14.25
f1 (ppm)

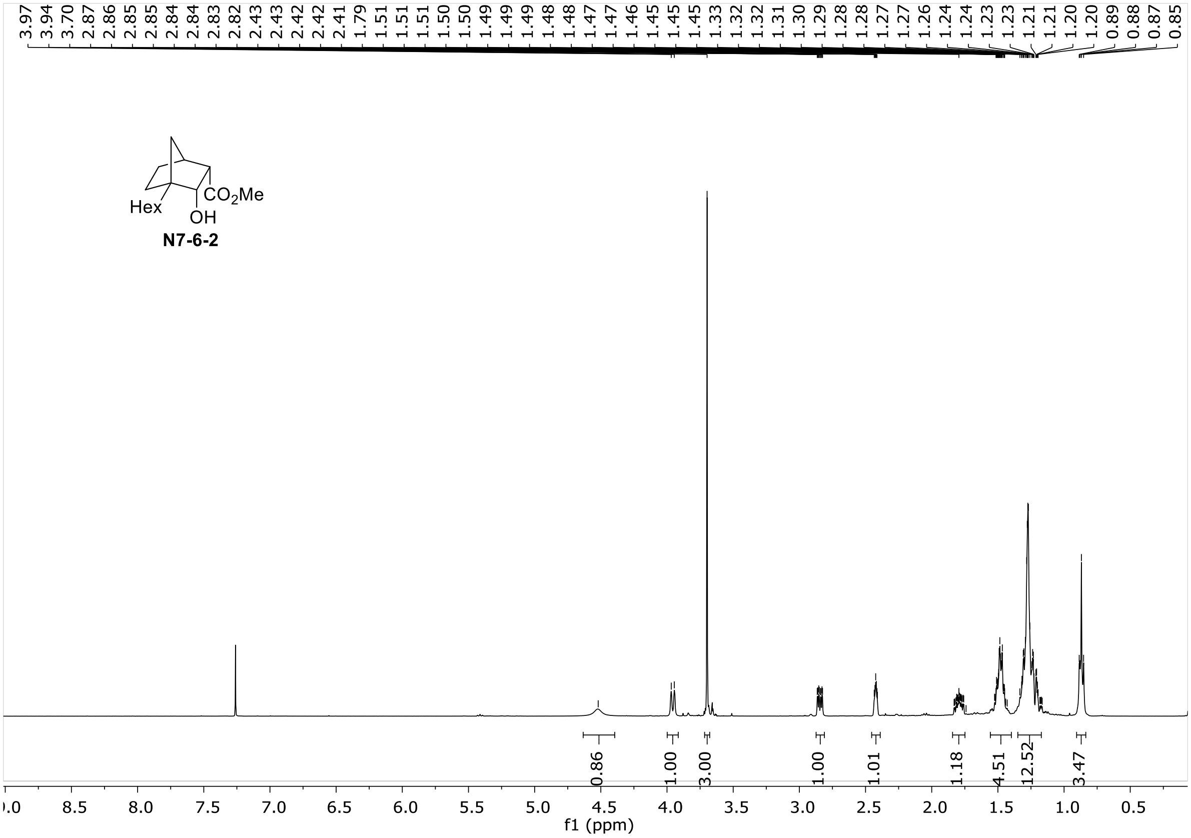
3.97
3.94
3.70
2.87
2.86
2.85
2.85
2.84
2.84
2.83
2.82
2.43
2.43
2.42
2.42
2.41
1.79
1.51
1.51
1.51
1.50
1.50
1.49
1.49
1.49
1.48
1.48
1.47
1.47
1.46
1.45
1.45
1.45
1.33
1.32
1.32
1.31
1.30
1.29
1.28
1.28
1.27
1.27
1.26
1.24
1.24
1.23
1.23
1.21
1.21
1.20
0.89
0.88
0.87
0.85
Hex
CO_2Me
OH
N7-6-2
0.86
1.00
3.00
1.00
1.01
1.18
4.51
12.52
3.47
f1 (ppm)

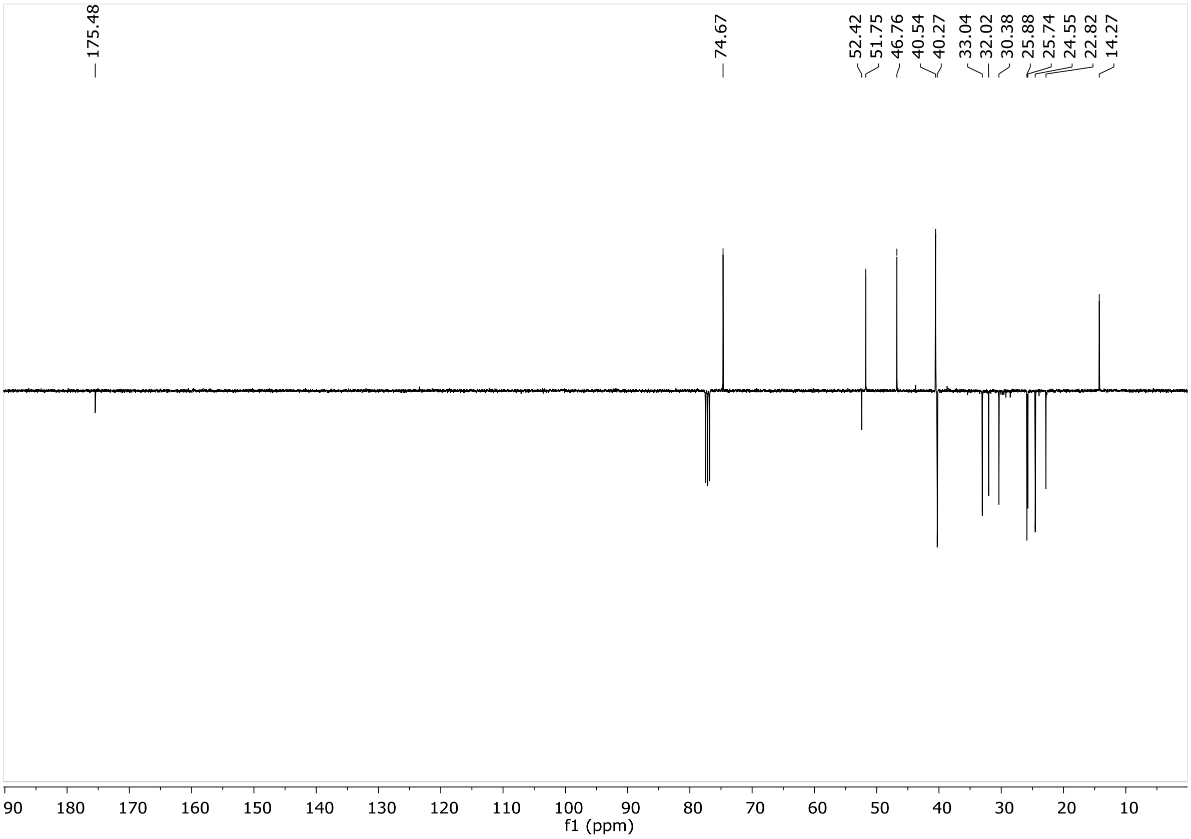
175.48
74.67
52.42
51.75
46.76
40.54
40.27
33.04
32.02
30.38
25.88
25.74
24.55
22.82
14.27
f1 (ppm)

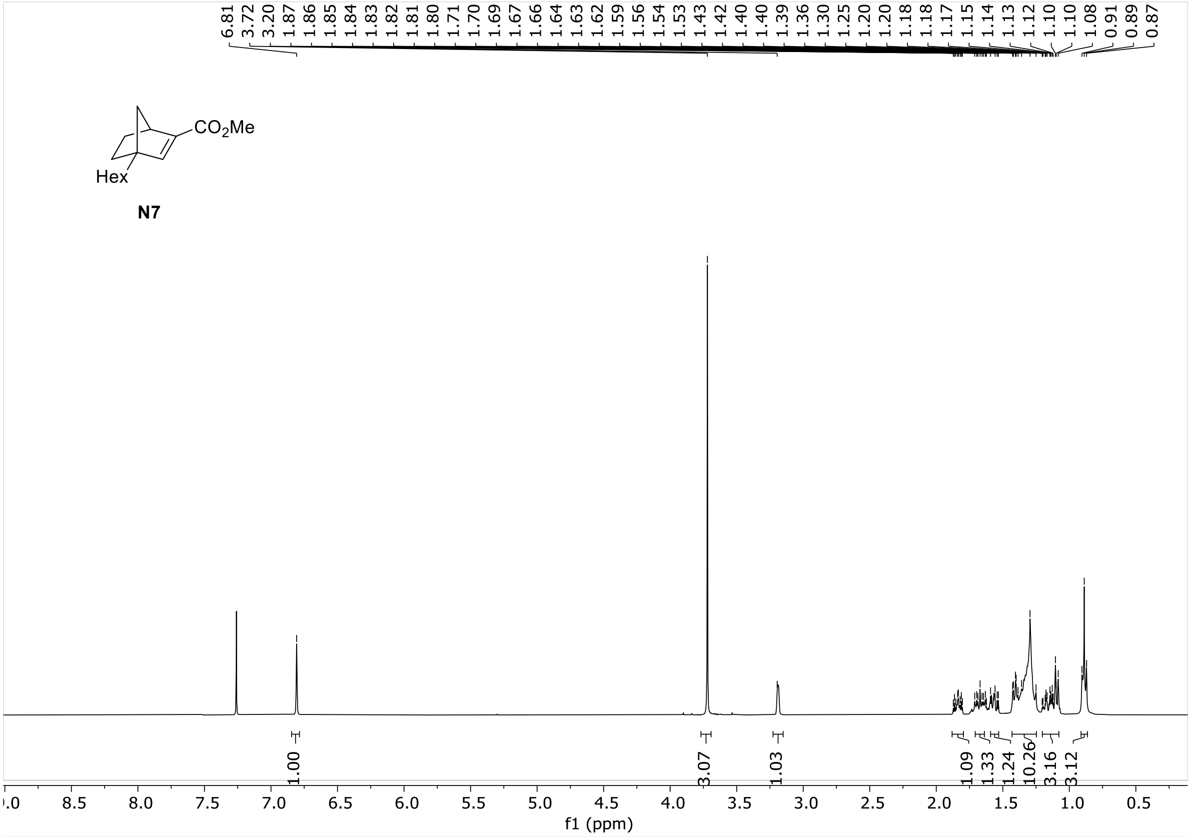
6.81
3.72
3.20
1.87
1.86
1.85
1.84
1.83
1.82
1.81
1.80
1.71
1.70
1.69
1.67
1.66
1.64
1.63
1.62
1.59
1.56
1.54
1.53
1.43
1.42
1.40
1.40
1.39
1.36
1.30
1.25
1.20
1.20
1.18
1.18
1.17
1.15
1.14
1.13
1.12
1.10
1.10
1.08
0.91
0.89
0.87
CO_2Me
Hex
N7
1.00
3.07
1.03
1.09
1.33
1.24
10.26
3.16
3.12
f1 (ppm)

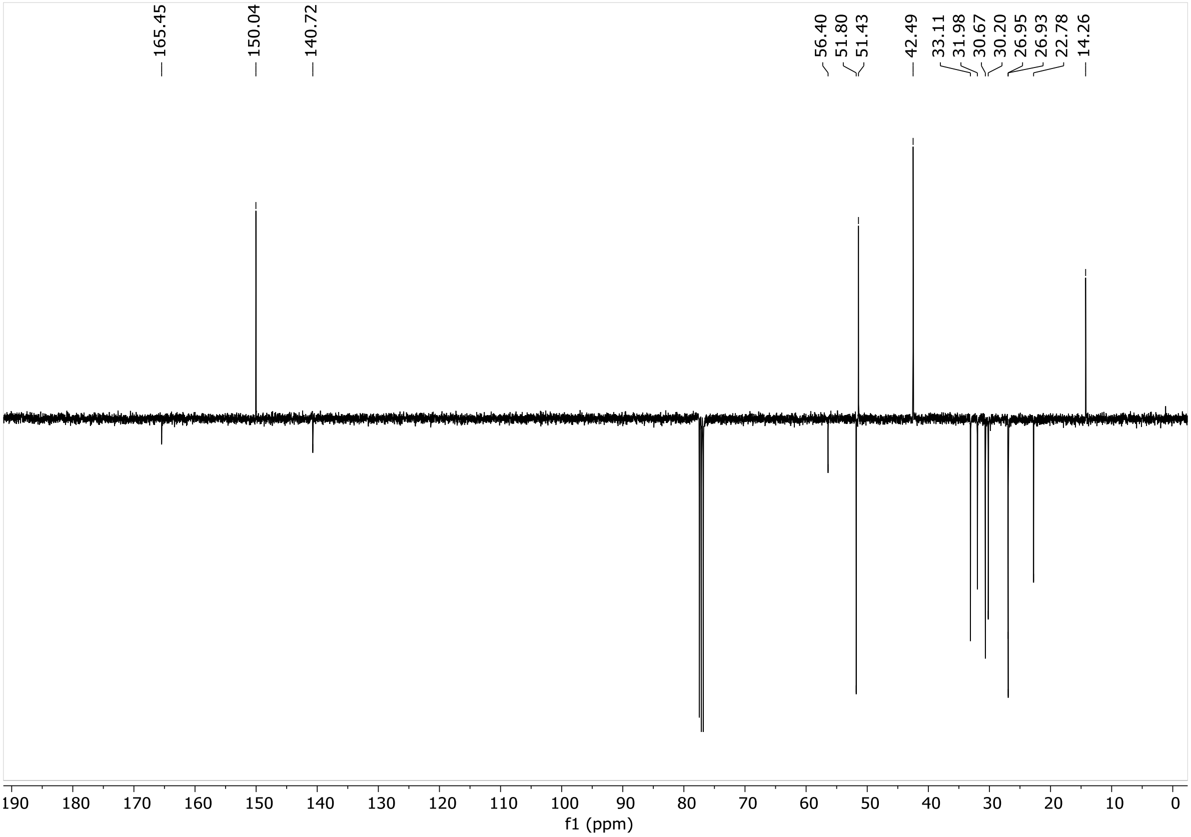
165.45
150.04
140.72
56.40
51.80
51.43
42.49
33.11
31.98
30.67
30.20
26.95
26.93
22.78
14.26
f1 (ppm)

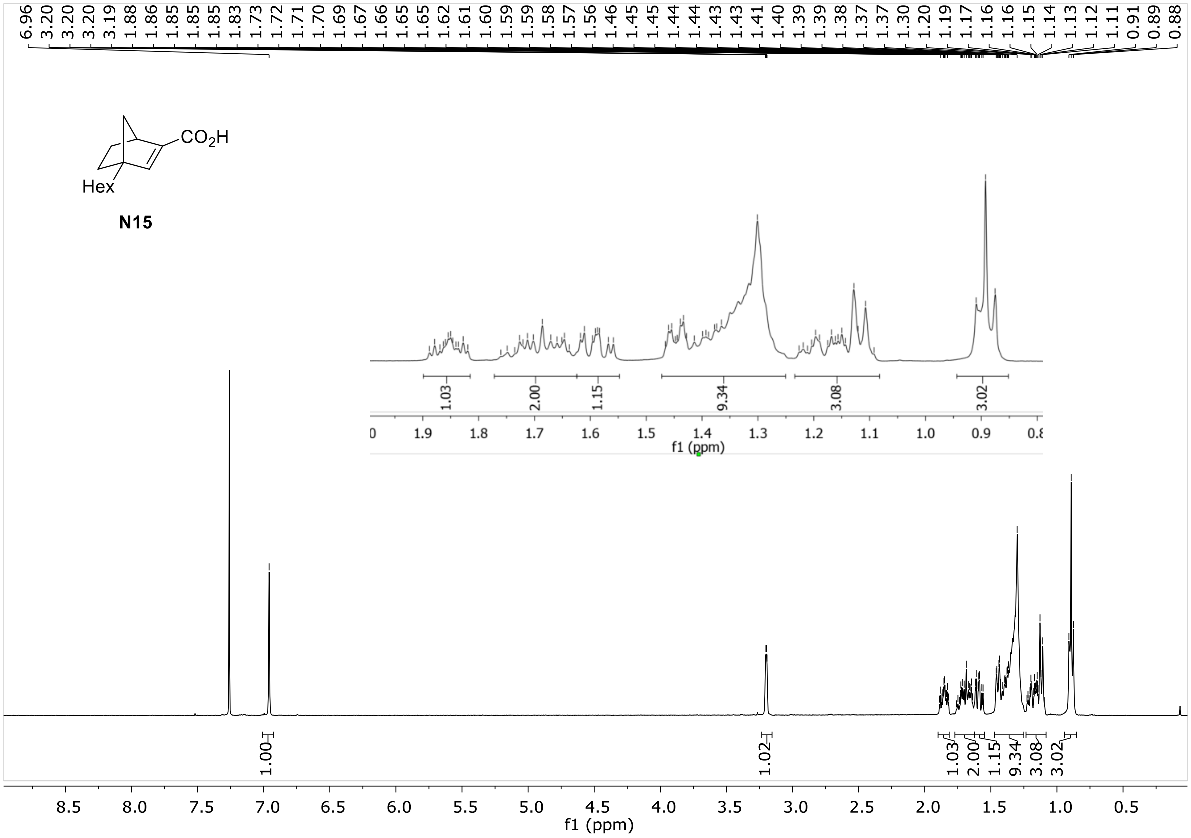
6.96
3.20
3.20
3.20
3.19
1.88
1.86
1.85
1.85
1.85
1.83
1.73
1.72
1.71
1.70
1.69
1.67
1.66
1.65
1.65
1.62
1.61
1.60
1.59
1.59
1.58
1.57
1.56
1.46
1.45
1.45
1.44
1.44
1.44
1.43
1.43
1.41
1.40
1.39
1.39
1.38
1.37
1.37
1.30
1.20
1.19
1.17
1.16
1.16
1.15
1.14
1.13
1.12
1.11
0.91
0.89
0.88
CO2H
Hex
N15
1.03
2.00
1.15
9.34
3.08
3.02
f1 (ppm)
1.00
1.02
1.03
2.00
1.15
9.34
3.08
3.02
f1 (ppm)

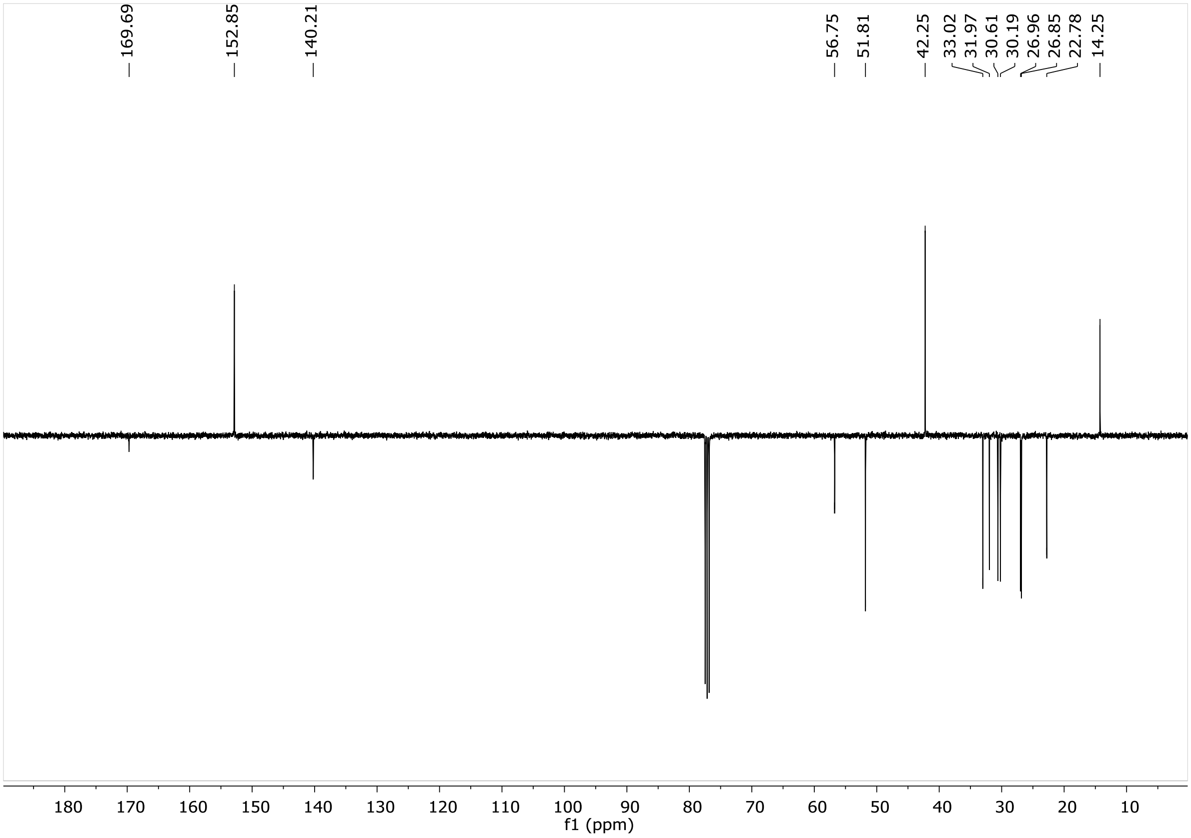
169.69
152.85
140.21
56.75
51.81
42.25
33.02
31.97
30.61
30.19
26.96
26.85
22.78
14.25
f1 (ppm)

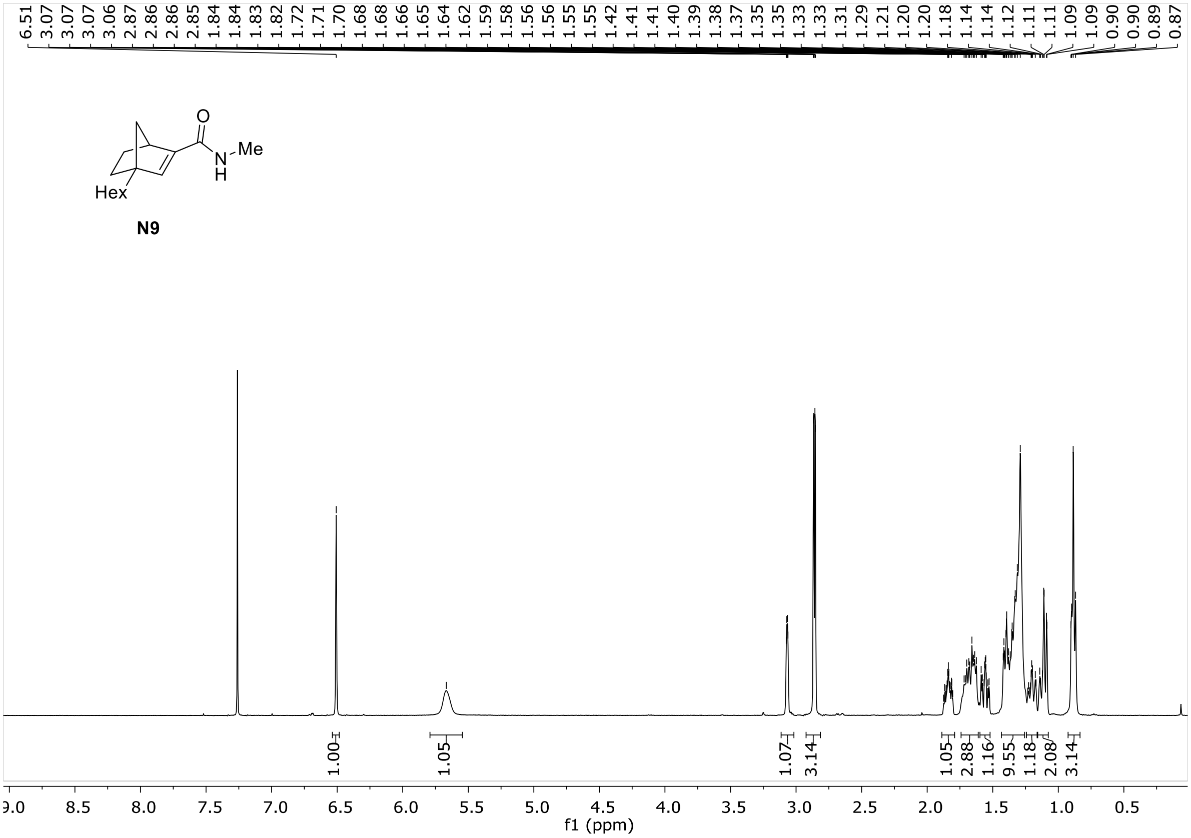
6.51
3.07
3.07
3.07
3.06
2.87
2.86
2.86
2.85
1.84
1.84
1.83
1.82
1.72
1.71
1.70
1.68
1.68
1.66
1.65
1.64
1.62
1.59
1.58
1.56
1.56
1.55
1.55
1.42
1.41
1.41
1.40
1.39
1.38
1.37
1.35
1.35
1.33
1.33
1.31
1.29
1.21
1.20
1.20
1.18
1.14
1.14
1.12
1.11
1.11
1.09
1.09
0.90
0.90
0.89
0.87
O
N
Me
H
Hex
N9
1.00
1.05
1.07
3.14
1.05
2.88
1.16
9.55
1.18
2.08
3.14
9.0 8.5 8.0 7.5 7.0 6.5 6.0 5.5 5.0 4.5 4.0 3.5 3.0 2.5 2.0 1.5 1.0 0.5
f1 (ppm)

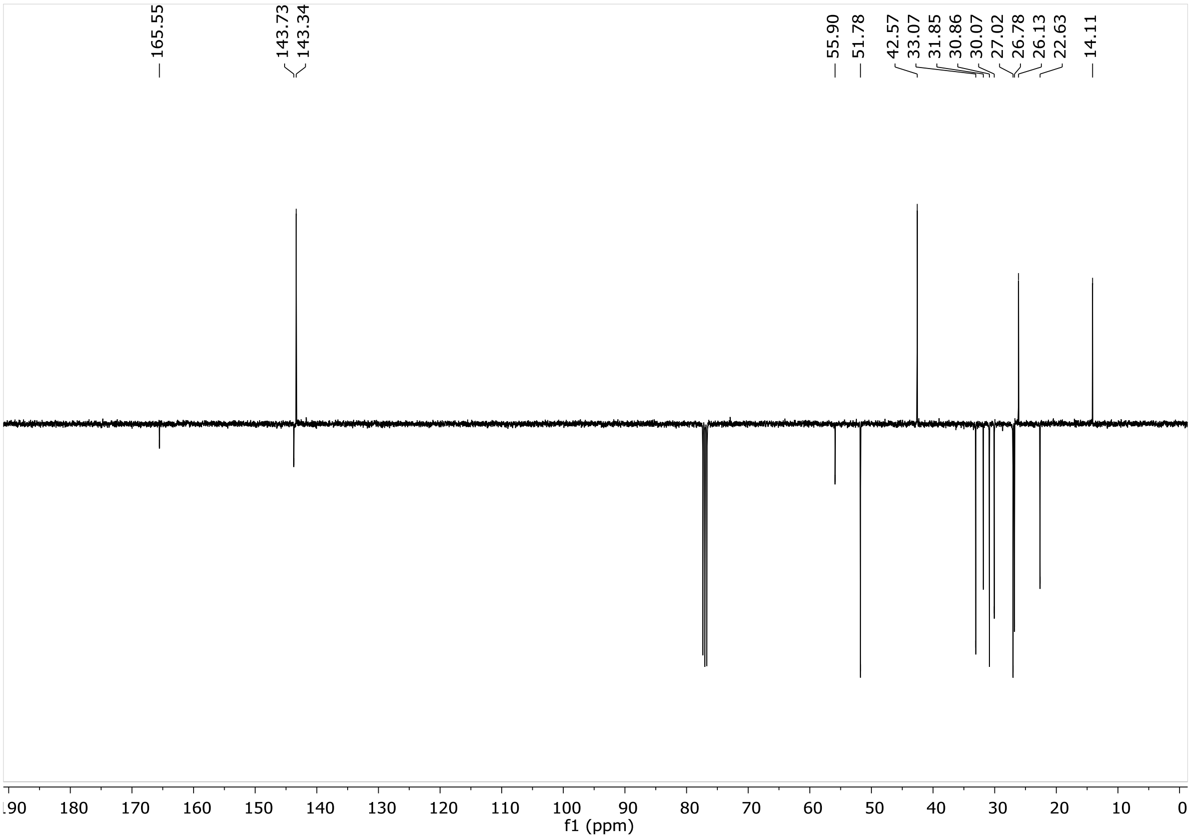
165.55
143.73
143.34
55.90
51.78
42.57
33.07
31.85
30.86
30.07
27.02
26.78
26.13
22.63
14.11
190 180 170 160 150 140 130 120 110 100 90 80 70 60 50 40 30 20 10 0
f1 (ppm)

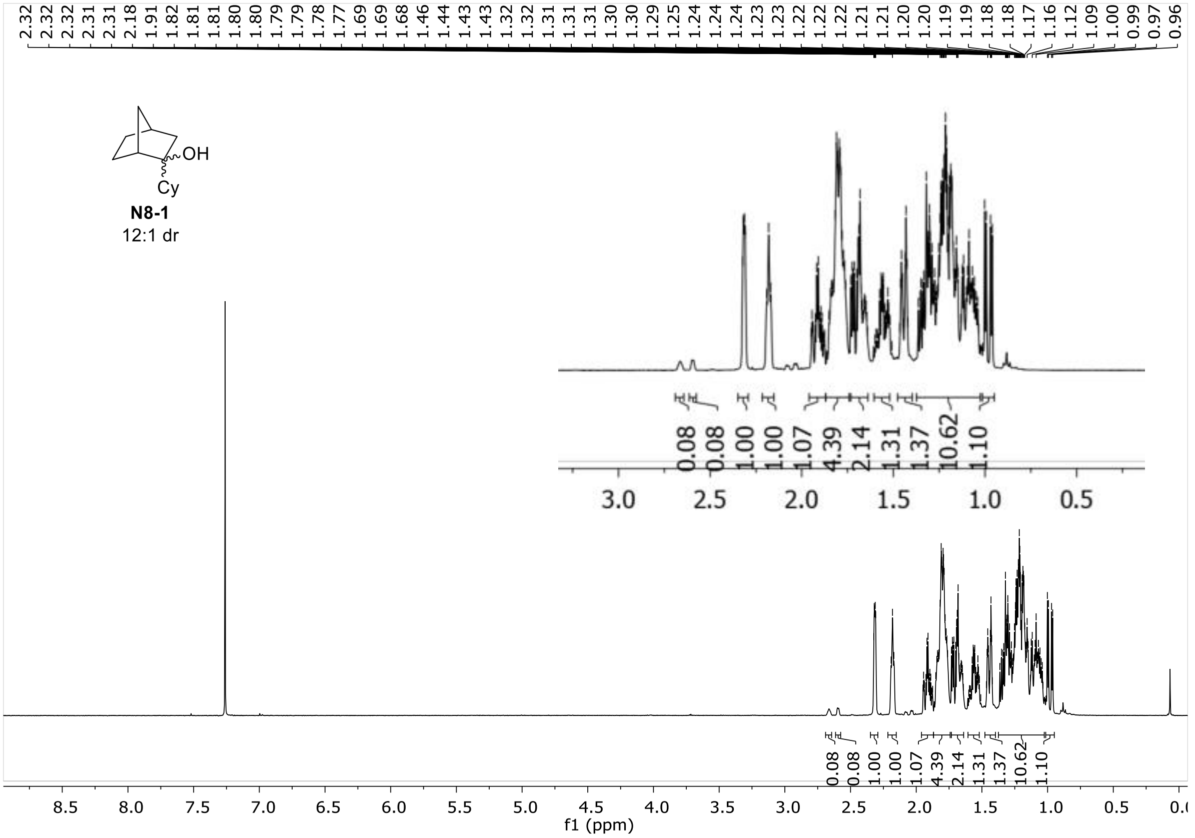
2.32 2.32 2.32 2.31 2.31 2.18 1.91 1.82 1.81 1.81 1.80 1.80 1.79 1.79 1.78 1.77 1.69 1.69 1.68 1.46 1.44 1.43 1.43 1.32 1.32 1.31 1.31 1.31 1.30 1.30 1.29 1.25 1.24 1.24 1.24 1.23 1.23 1.22 1.22 1.22 1.22 1.21 1.21 1.20 1.20 1.19 1.19 1.18 1.18 1.17 1.16 1.12 1.09 1.00 0.99 0.97 0.96
OH
Cy
N8-1
12:1 dr
0.08 0.08 1.00 1.00 1.07 4.39 2.14 1.31 1.37 10.62 1.10
3.0 2.5 2.0 1.5 1.0 0.5
8.5 8.0 7.5 7.0 6.5 6.0 5.5 5.0 4.5 4.0 3.5 3.0 2.5 2.0 1.5 1.0 0.5 0.0
f1 (ppm)

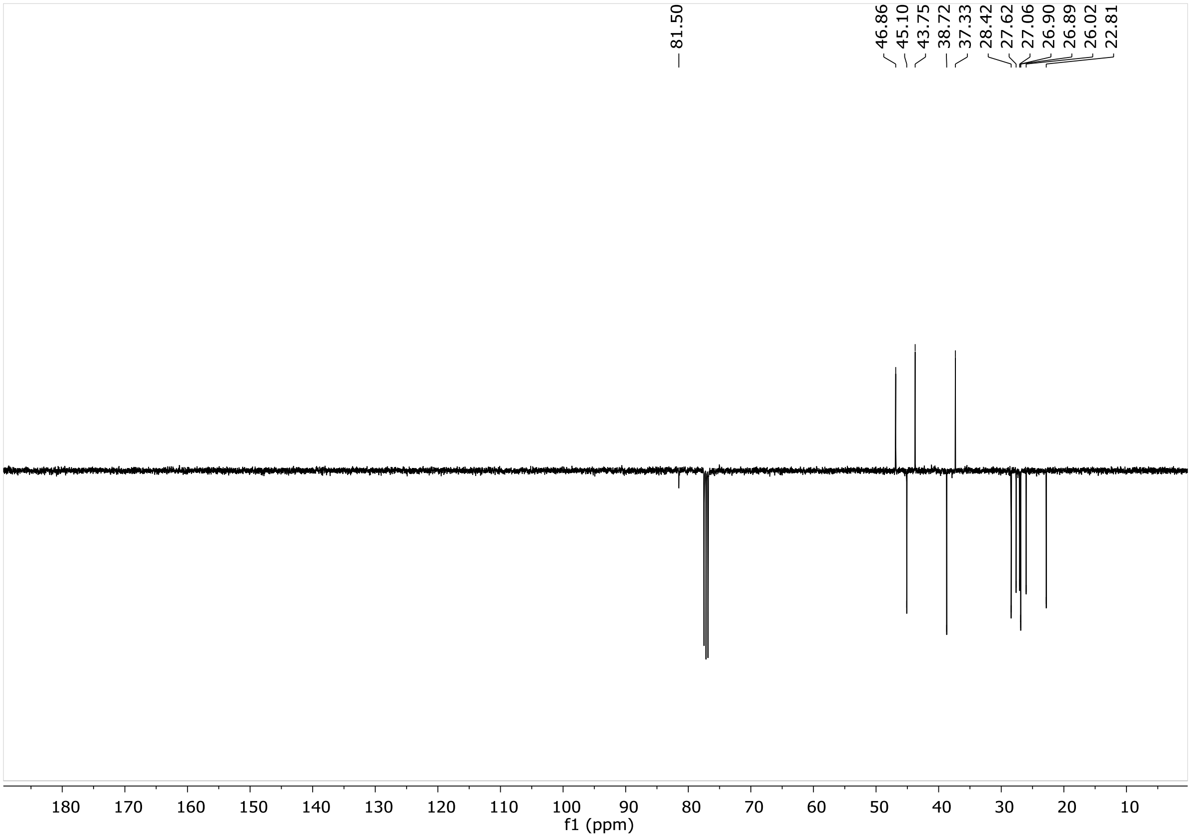
81.50
46.86 45.10 43.75 38.72 37.33 28.42 27.62 27.06 26.90 26.89 26.02 22.81
180 170 160 150 140 130 120 110 100 90 80 70 60 50 40 30 20 10
f1 (ppm)

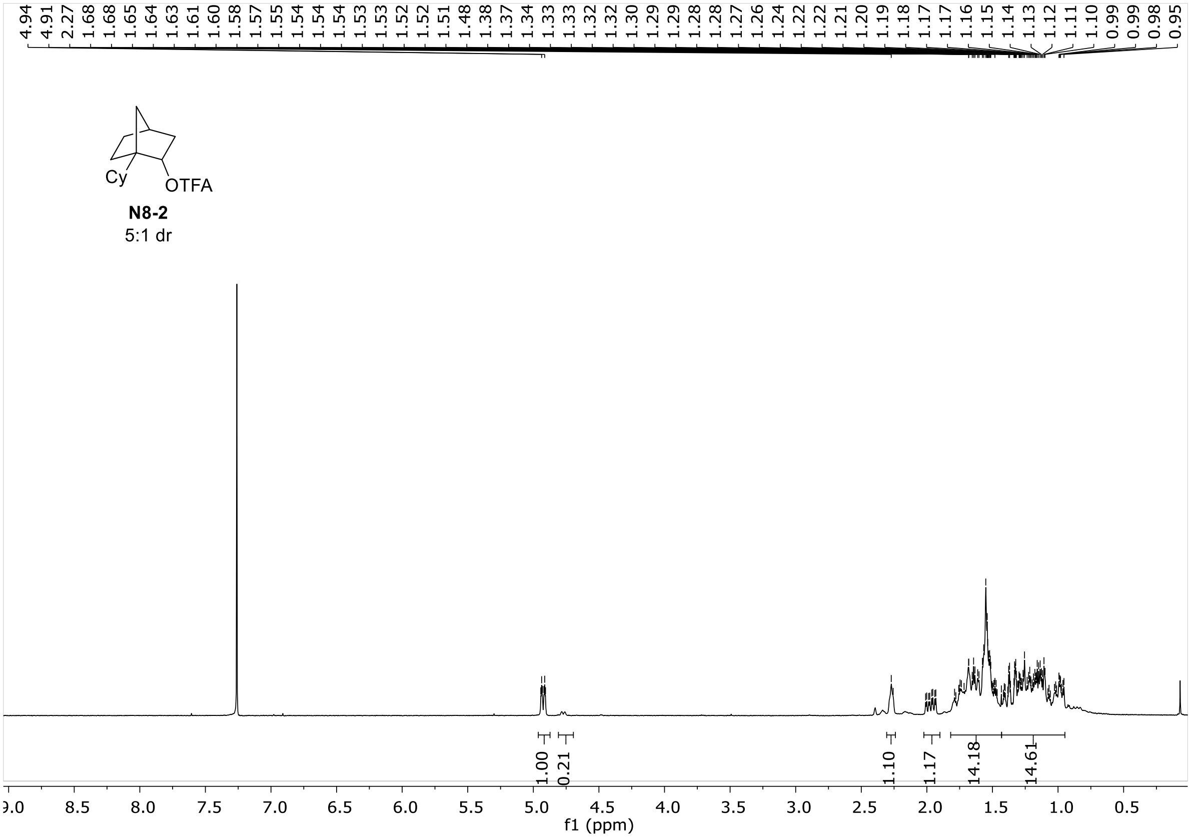
4.94
4.91
2.27
1.68
1.68
1.65
1.64
1.63
1.61
1.60
1.58
1.57
1.55
1.54
1.54
1.54
1.53
1.53
1.52
1.52
1.51
1.48
1.38
1.37
1.34
1.33
1.33
1.32
1.32
1.30
1.29
1.29
1.28
1.28
1.27
1.26
1.24
1.22
1.22
1.21
1.20
1.19
1.18
1.17
1.17
1.16
1.15
1.14
1.13
1.12
1.11
1.10
0.99
0.99
0.98
0.95
Cy
OTFA
N8-2
5:1 dr
1.00
0.21
1.10
1.17
14.18
14.61
9.0 8.5 8.0 7.5 7.0 6.5 6.0 5.5 5.0 4.5 4.0 3.5 3.0 2.5 2.0 1.5 1.0 0.5
f1 (ppm)

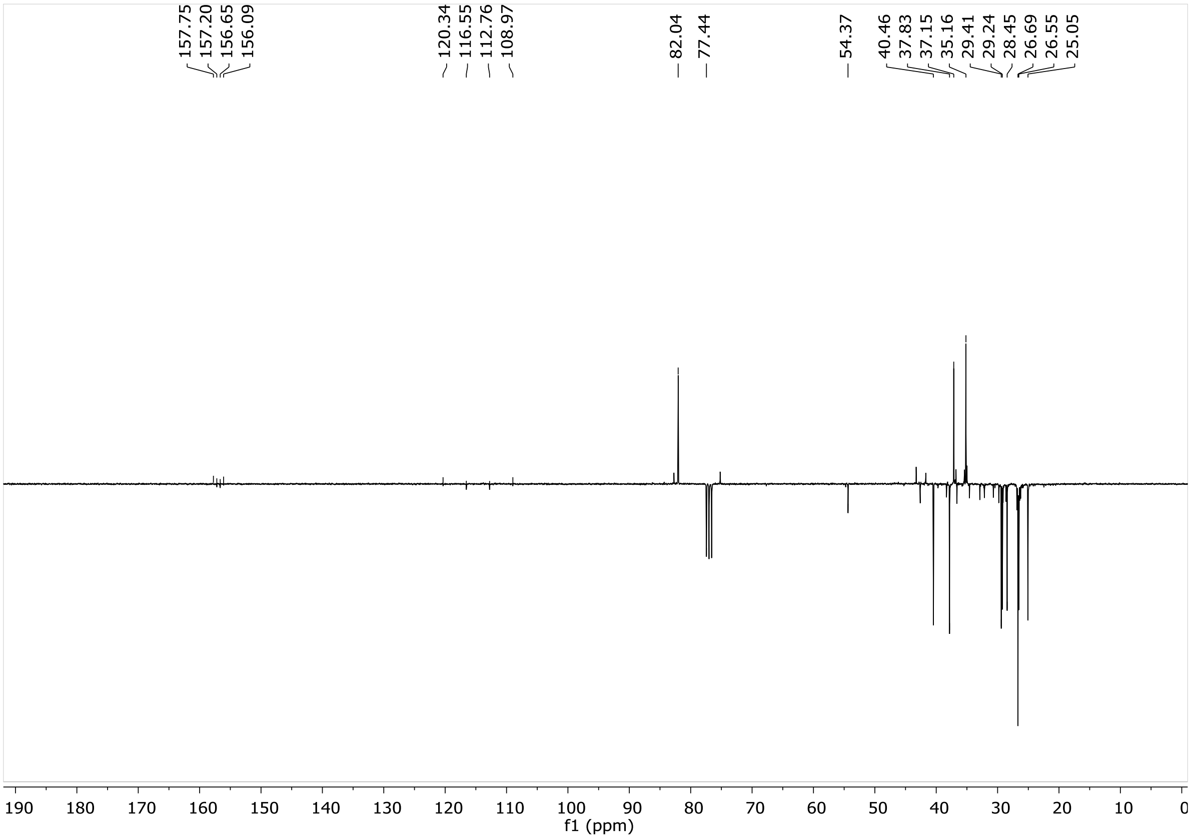
157.75
157.20
156.65
156.09
120.34
116.55
112.76
108.97
82.04
77.44
54.37
40.46
37.83
37.15
35.16
29.41
29.24
28.45
26.69
26.55
25.05
190 180 170 160 150 140 130 120 110 100 90 80 70 60 50 40 30 20 10 0
f1 (ppm)

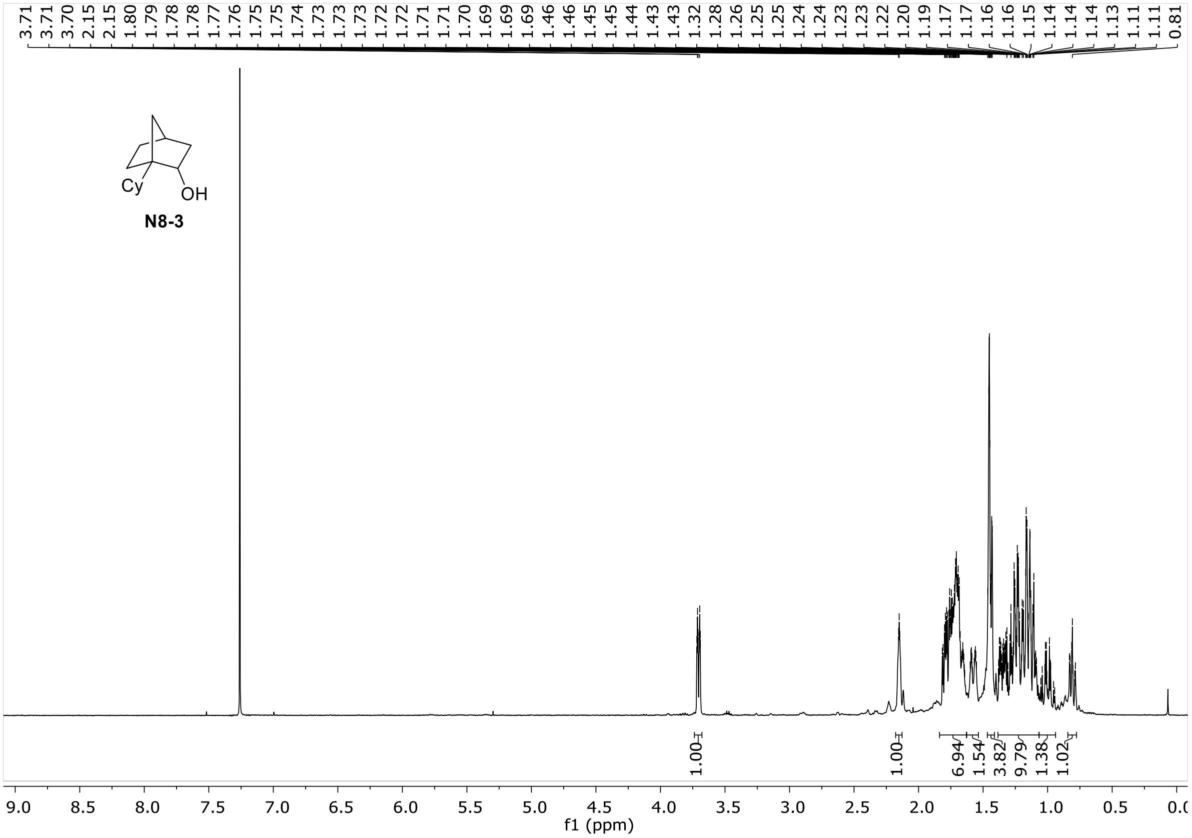
3.71
3.71
3.70
2.15
2.15
1.80
1.79
1.78
1.78
1.77
1.76
1.75
1.75
1.74
1.73
1.73
1.73
1.72
1.72
1.71
1.71
1.70
1.69
1.69
1.69
1.46
1.46
1.45
1.45
1.44
1.43
1.43
1.32
1.28
1.26
1.25
1.25
1.24
1.24
1.23
1.23
1.22
1.20
1.19
1.17
1.17
1.16
1.16
1.15
1.14
1.14
1.14
1.13
1.11
1.11
0.81
Cy
OH
N8-3
1.00
1.00
6.94
1.54
3.82
9.79
1.38
1.02
9.0 8.5 8.0 7.5 7.0 6.5 6.0 5.5 5.0 4.5 4.0 3.5 3.0 2.5 2.0 1.5 1.0 0.5 0.0
f1 (ppm)

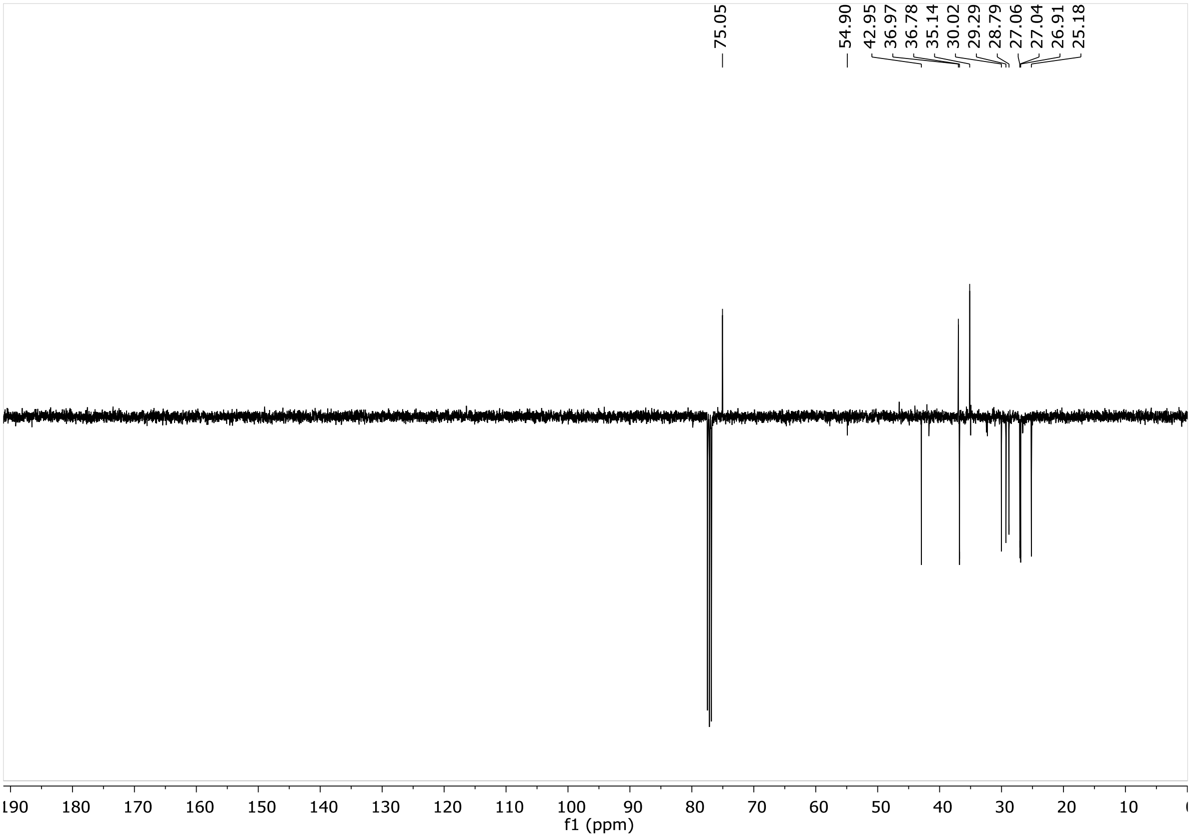
75.05
54.90
42.95
36.97
36.78
35.14
30.02
29.29
28.79
27.06
27.04
26.91
25.18
190 180 170 160 150 140 130 120 110 100 90 80 70 60 50 40 30 20 10
f1 (ppm)

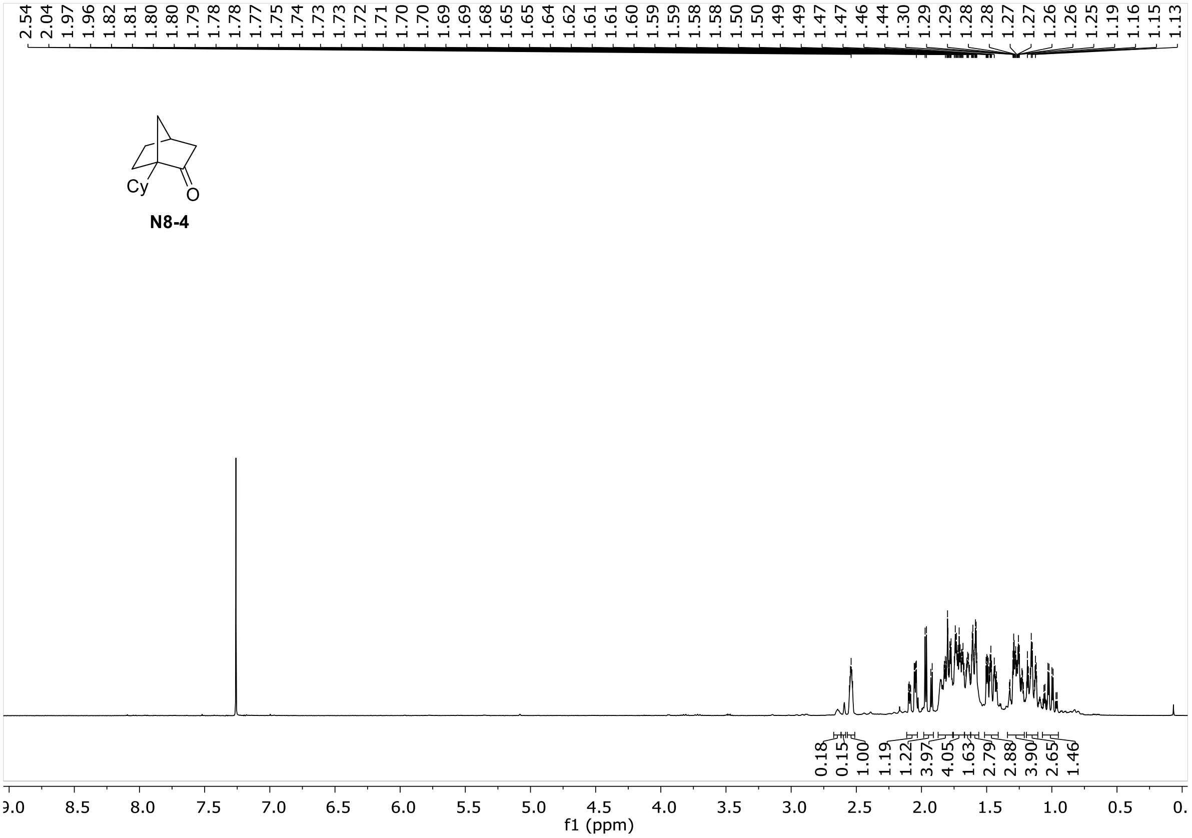
2.54
2.04
1.97
1.96
1.82
1.81
1.80
1.80
1.79
1.78
1.78
1.77
1.75
1.74
1.73
1.73
1.72
1.71
1.70
1.70
1.69
1.69
1.68
1.65
1.65
1.64
1.62
1.61
1.61
1.60
1.59
1.59
1.58
1.58
1.50
1.50
1.49
1.49
1.47
1.47
1.46
1.44
1.30
1.29
1.29
1.28
1.28
1.27
1.27
1.26
1.26
1.25
1.19
1.16
1.15
1.13
Cy
O
N8-4
0.18
0.15
1.00
1.19
1.22
3.97
4.05
1.63
2.79
2.88
3.90
2.65
1.46
9.0 8.5 8.0 7.5 7.0 6.5 6.0 5.5 5.0 4.5 4.0 3.5 3.0 2.5 2.0 1.5 1.0 0.5 0.
f1 (ppm)

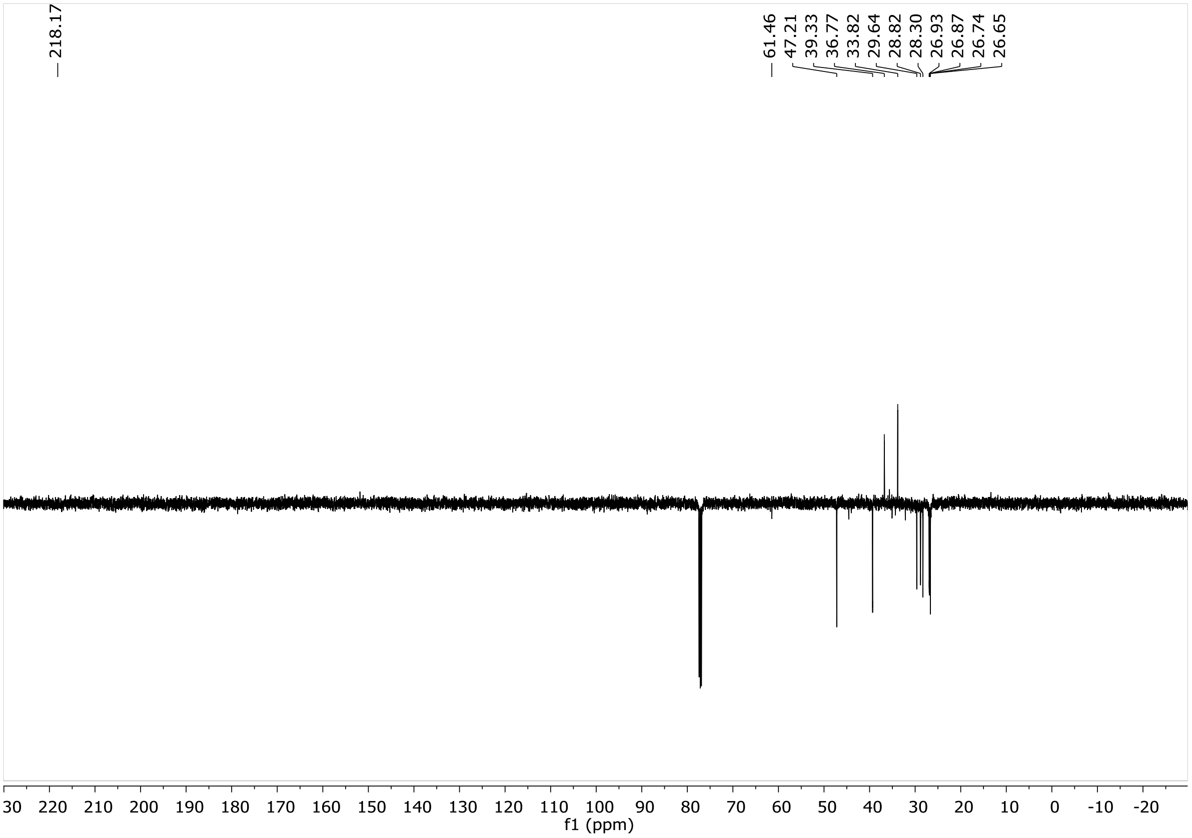
218.17
61.46
47.21
39.33
36.77
33.82
29.64
28.82
28.30
26.93
26.87
26.74
26.65
30 220 210 200 190 180 170 160 150 140 130 120 110 100 90 80 70 60 50 40 30 20 10 0 -10 -20
f1 (ppm)

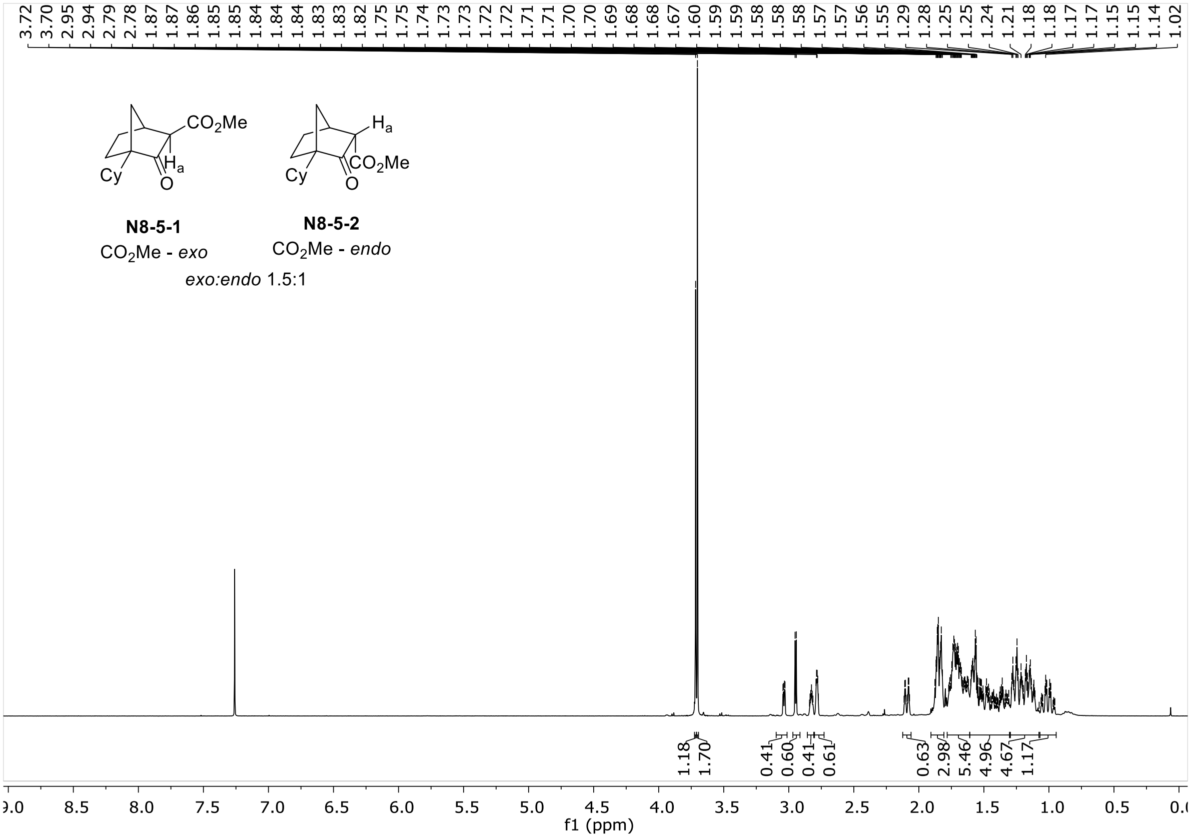
3.72
3.70
2.95
2.94
2.79
2.78
1.87
1.87
1.86
1.85
1.85
1.84
1.84
1.84
1.83
1.83
1.82
1.75
1.75
1.74
1.73
1.73
1.72
1.72
1.71
1.71
1.70
1.70
1.69
1.68
1.68
1.67
1.60
1.59
1.59
1.58
1.58
1.58
1.57
1.57
1.56
1.55
1.29
1.28
1.25
1.25
1.24
1.21
1.18
1.18
1.17
1.17
1.15
1.15
1.14
1.02
CO2Me
Ha
Cy
O
Ha
CO2Me
Cy
O
N8-5-1
CO2Me - exo
N8-5-2
CO2Me - endo
exo:endo 1.5:1
1.18
1.70
0.41
0.60
0.41
0.61
0.63
2.98
5.46
4.96
4.67
1.17
8.5 8.0 7.5 7.0 6.5 6.0 5.5 5.0 4.5 4.0 3.5 3.0 2.5 2.0 1.5 1.0 0.5
f1 (ppm)

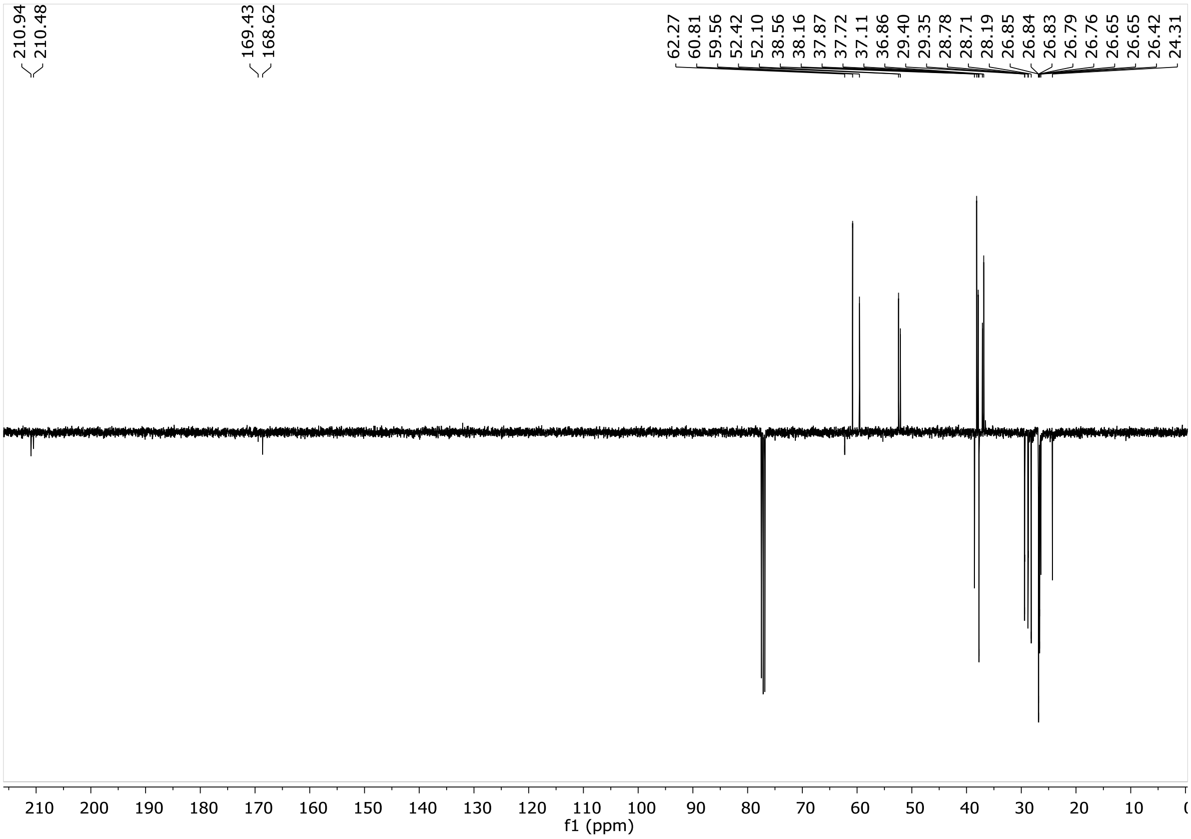
210.94
210.48
169.43
168.62
62.27
60.81
59.56
52.42
52.10
38.56
38.16
37.87
37.72
37.11
36.86
29.40
29.35
28.78
28.71
28.19
26.85
26.84
26.83
26.79
26.76
26.65
26.65
26.42
24.31
210 200 190 180 170 160 150 140 130 120 110 100 90 80 70 60 50 40 30 20 10
f1 (ppm)

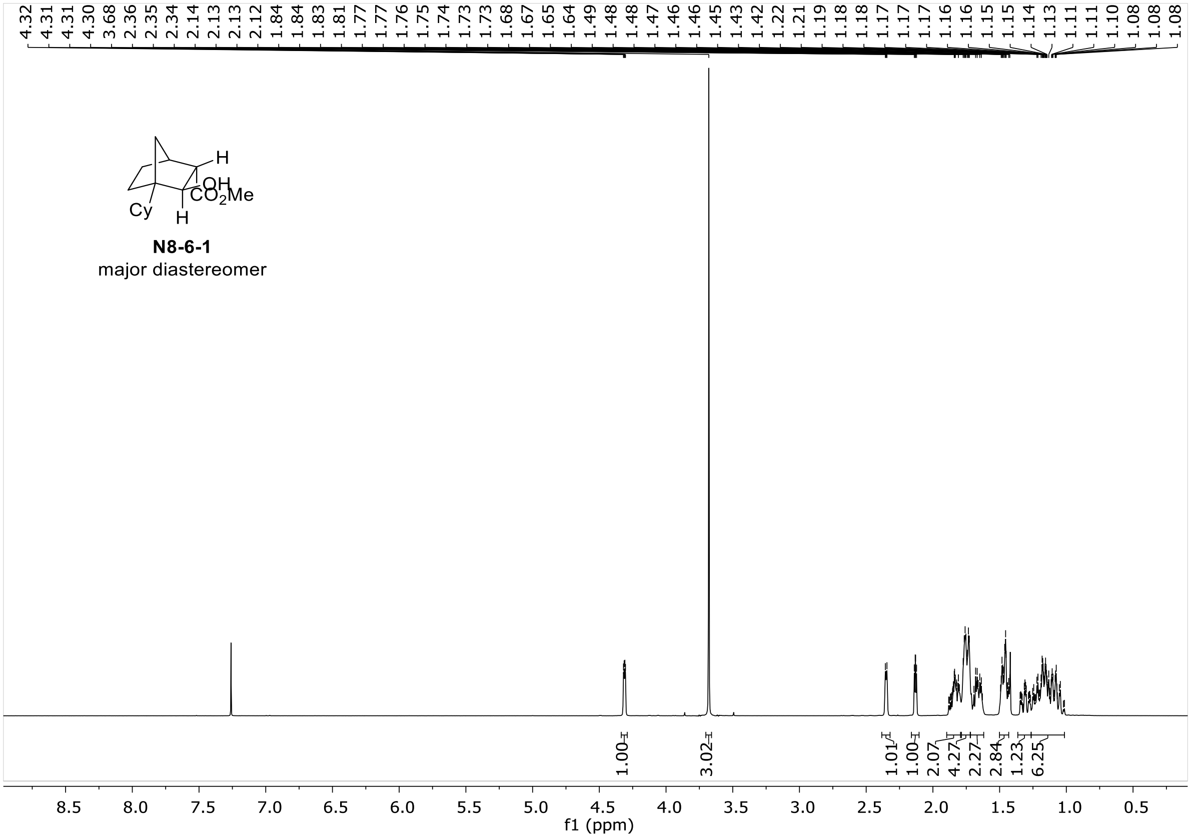
4.32 4.31 4.31 4.30 3.68 2.36 2.35 2.34 2.14 2.13 2.12 1.84 1.84 1.83 1.81 1.77 1.77 1.76 1.75 1.74 1.73 1.73 1.68 1.67 1.65 1.64 1.49 1.48 1.48 1.47 1.46 1.46 1.45 1.43 1.42 1.22 1.21 1.19 1.18 1.18 1.17 1.17 1.17 1.16 1.16 1.15 1.15 1.14 1.13 1.11 1.10 1.08 1.08 1.08
H
OH
CO_2Me
Cy
H
N8-6-1
major diastereomer
1.00
3.02
1.01
1.00
2.07
4.27
2.27
2.84
1.23
6.25
8.5 8.0 7.5 7.0 6.5 6.0 5.5 5.0 4.5 4.0 3.5 3.0 2.5 2.0 1.5 1.0 0.5
f1 (ppm)

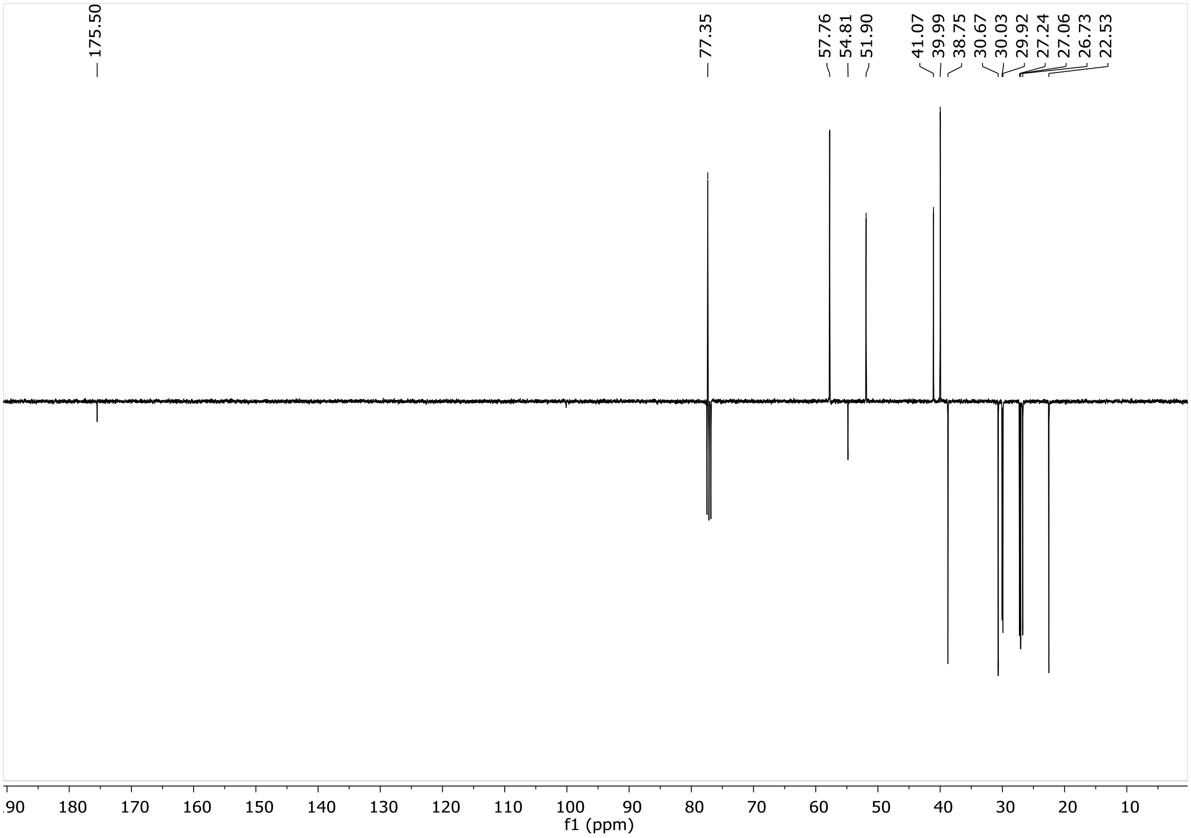
175.50
77.35
57.76
54.81
51.90
41.07
39.99
38.75
30.67
30.03
29.92
27.24
27.06
26.73
22.53
190 180 170 160 150 140 130 120 110 100 90 80 70 60 50 40 30 20 10
f1 (ppm)

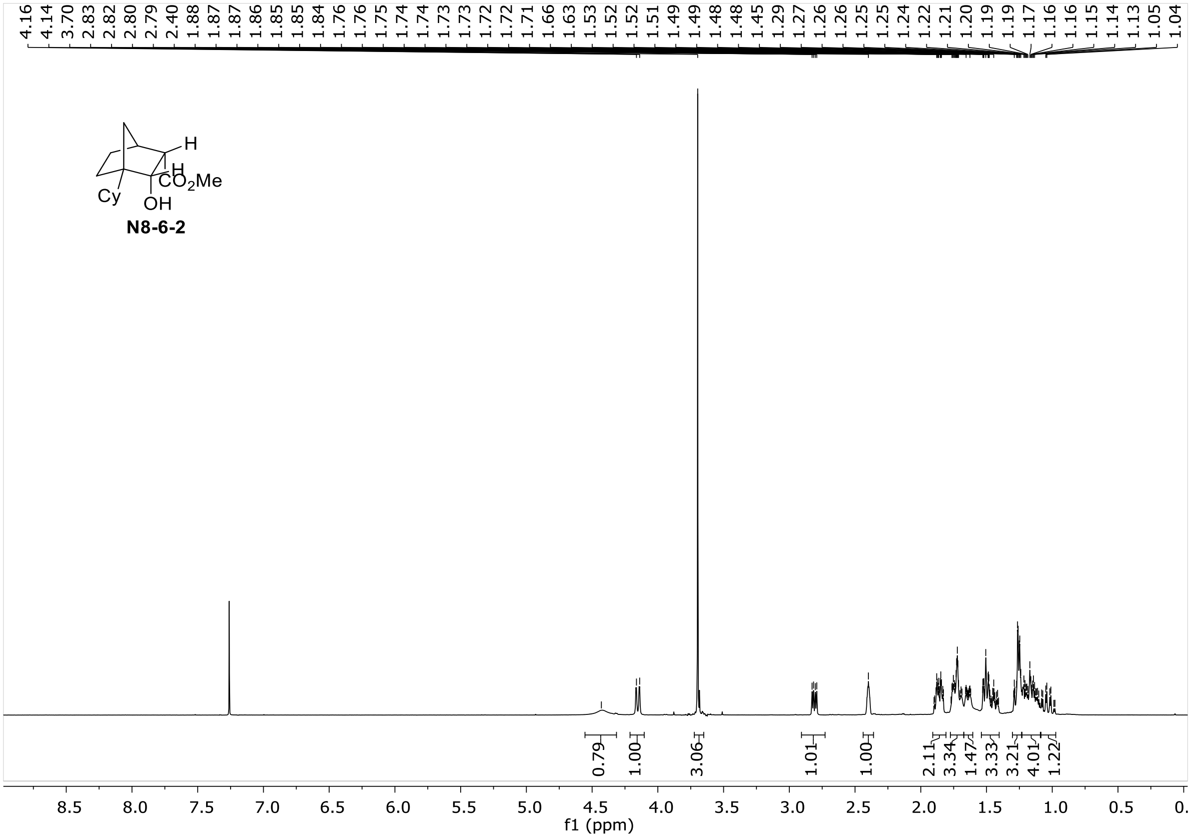
4.16 4.14 3.70 2.83 2.82 2.80 2.79 2.40 1.88 1.87 1.87 1.86 1.85 1.85 1.84 1.76 1.76 1.75 1.74 1.74 1.73 1.73 1.72 1.72 1.71 1.66 1.63 1.53 1.52 1.52 1.51 1.49 1.49 1.48 1.48 1.45 1.29 1.27 1.26 1.26 1.25 1.25 1.24 1.22 1.21 1.20 1.19 1.19 1.17 1.16 1.16 1.15 1.14 1.13 1.05 1.04
H
H
CO_2Me
Cy
OH
N8-6-2
0.79
1.00
3.06
1.01
1.00
2.11
3.34
1.47
3.33
3.21
4.01
1.22
8.5 8.0 7.5 7.0 6.5 6.0 5.5 5.0 4.5 4.0 3.5 3.0 2.5 2.0 1.5 1.0 0.5 0.
f1 (ppm)

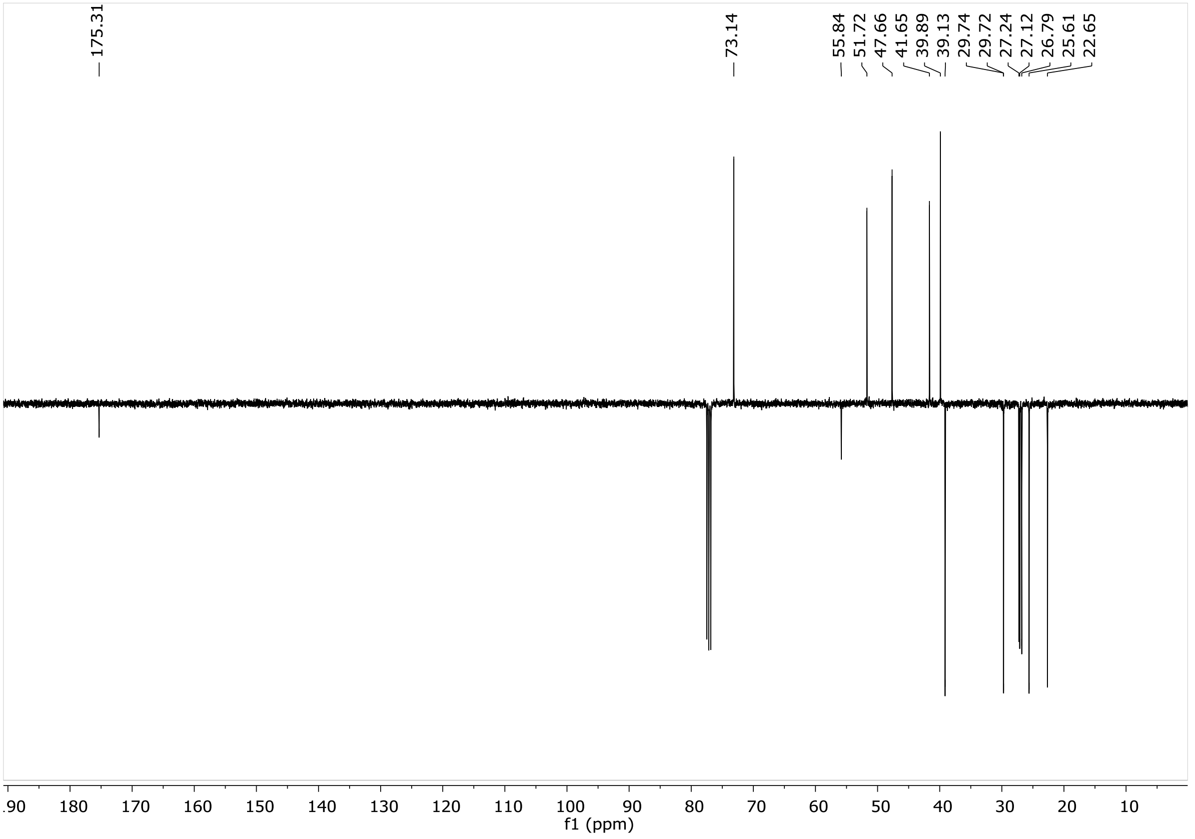
175.31
73.14
55.84 51.72 47.66 41.65 39.89 39.13 29.74 29.72 27.24 27.12 26.79 25.61 22.65
190 180 170 160 150 140 130 120 110 100 90 80 70 60 50 40 30 20 10
f1 (ppm)

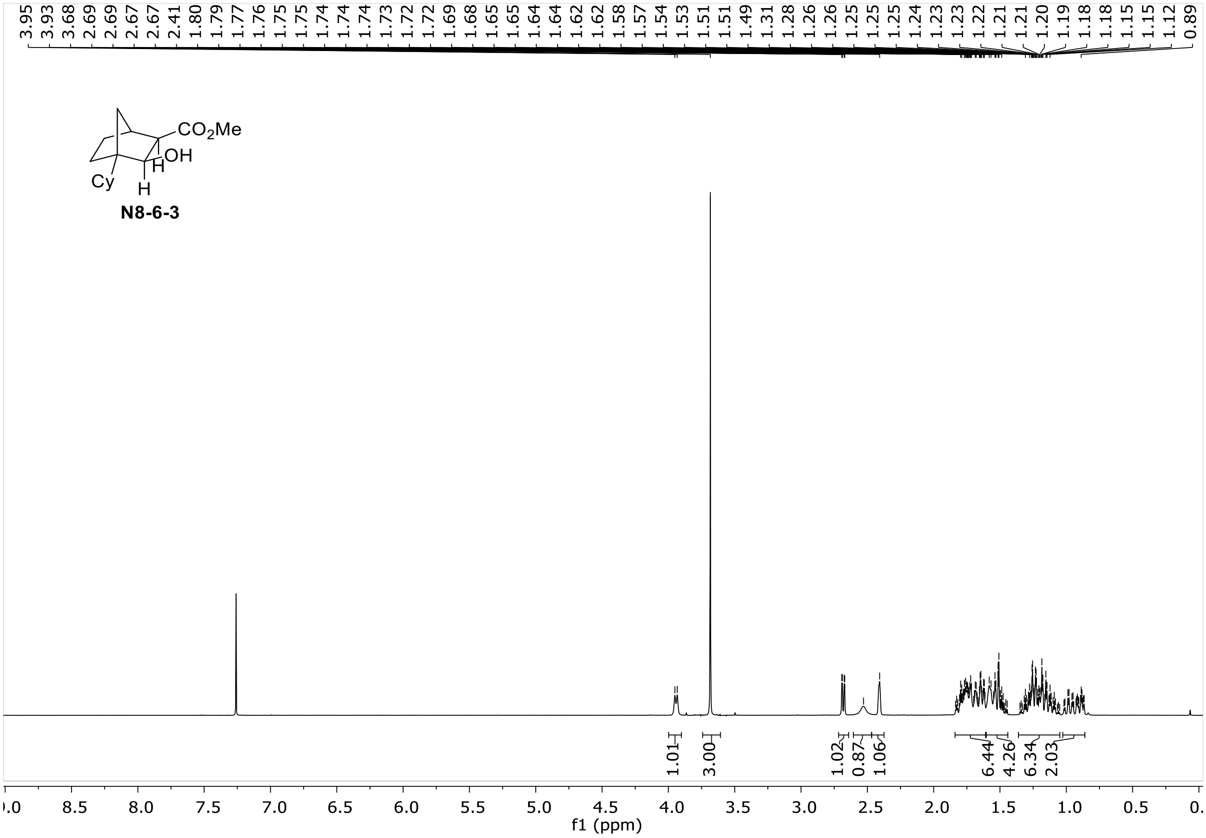
3.95 3.93 3.68 2.69 2.69 2.67 2.67 2.41 1.80 1.79 1.77 1.76 1.75 1.75 1.74 1.74 1.74 1.73 1.72 1.72 1.69 1.68 1.65 1.65 1.64 1.64 1.62 1.62 1.58 1.57 1.54 1.53 1.51 1.51 1.49 1.31 1.28 1.26 1.26 1.25 1.25 1.25 1.24 1.23 1.23 1.22 1.21 1.21 1.20 1.19 1.18 1.18 1.15 1.12 0.89
CO2Me
OH
H
Cy
H
N8-6-3
1.01
3.00
1.02
0.87
1.06
6.44
4.26
6.34
2.03
8.5 8.0 7.5 7.0 6.5 6.0 5.5 5.0 4.5 4.0 3.5 3.0 2.5 2.0 1.5 1.0 0.5
f1 (ppm)

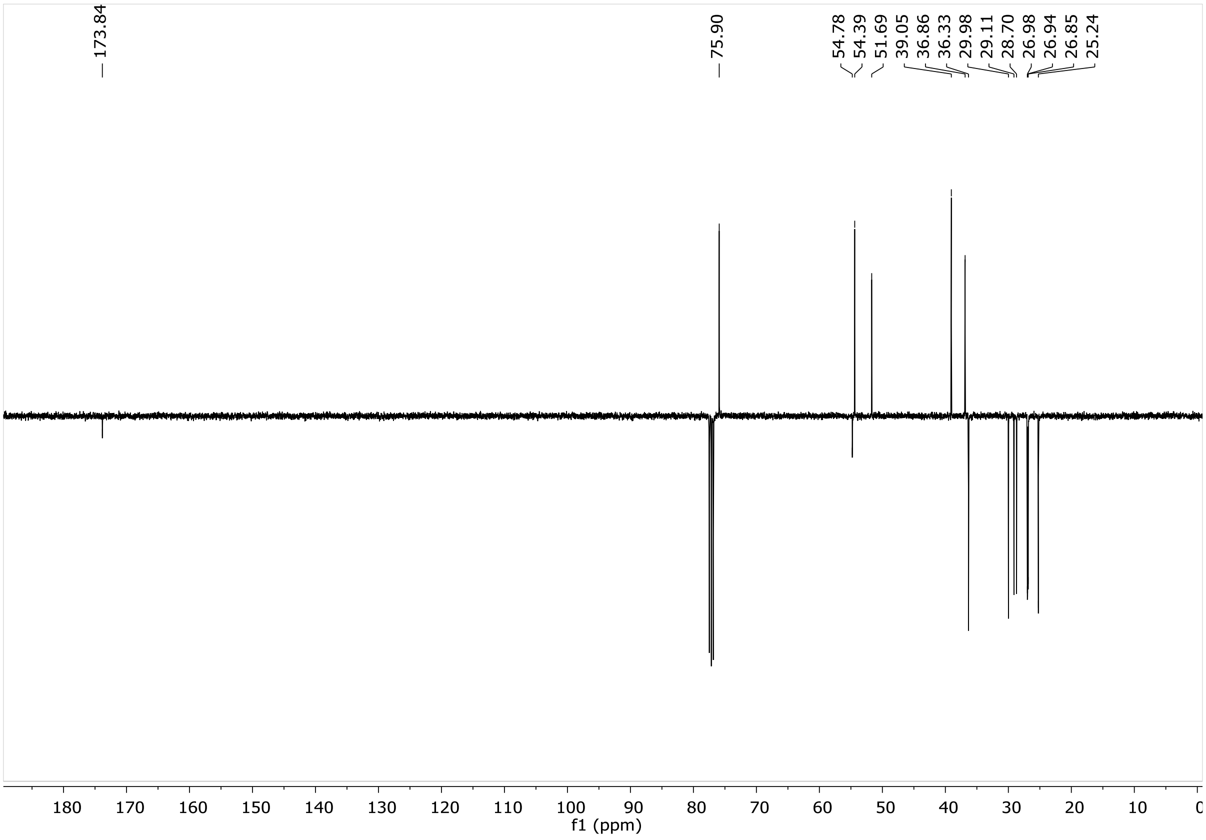
173.84
75.90
54.78 54.39 51.69 39.05 36.86 36.33 29.98 29.11 28.70 26.98 26.94 26.85 25.24
180 170 160 150 140 130 120 110 100 90 80 70 60 50 40 30 20 10
f1 (ppm)

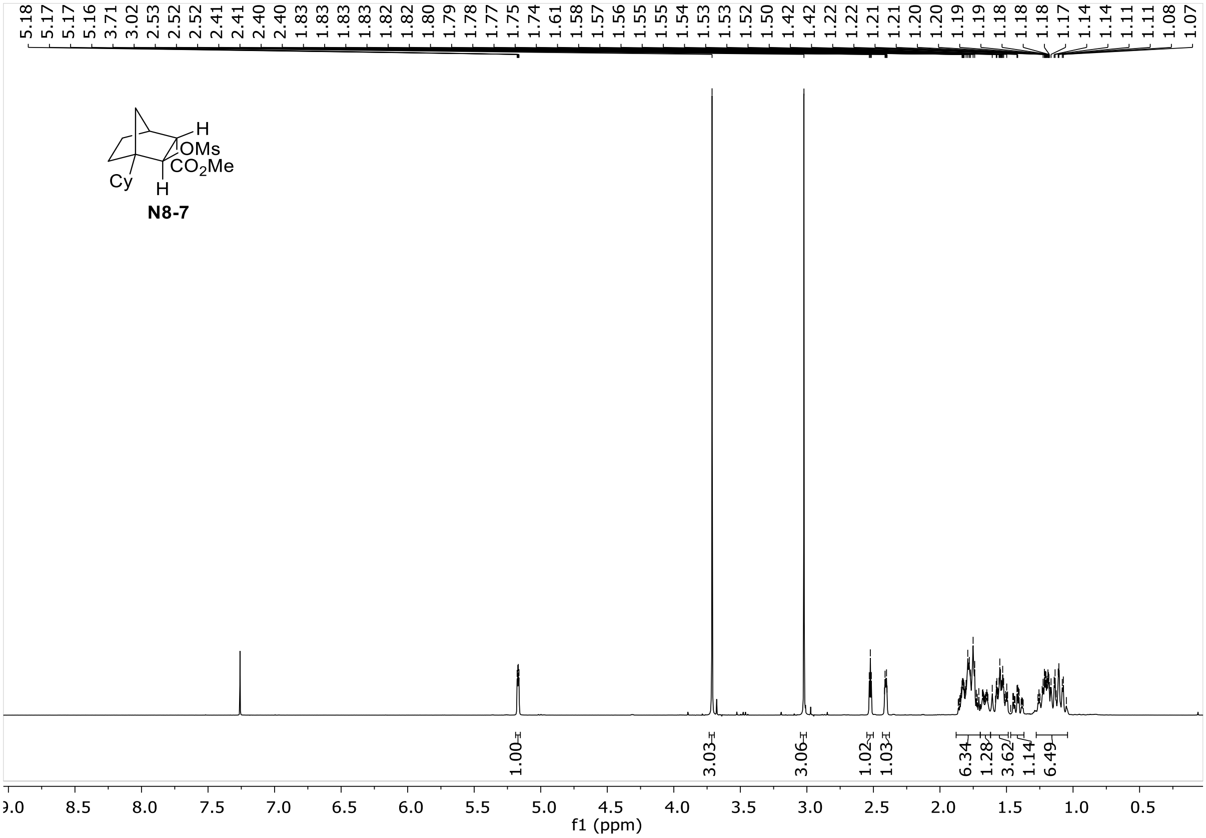
5.18 5.17 5.17 5.16 3.71 3.02 2.53 2.52 2.52 2.41 2.41 2.40 2.40 1.83 1.83 1.83 1.83 1.82 1.82 1.80 1.79 1.78 1.77 1.75 1.74 1.61 1.58 1.57 1.56 1.55 1.55 1.54 1.53 1.53 1.53 1.52 1.50 1.42 1.42 1.22 1.22 1.21 1.21 1.20 1.20 1.19 1.19 1.18 1.18 1.18 1.17 1.14 1.14 1.11 1.11 1.08 1.07
H
OMs
CO_2Me
Cy
H
N8-7
1.00 3.03 3.06 1.02 1.03 6.34 1.28 3.62 1.14 6.49
f1 (ppm)

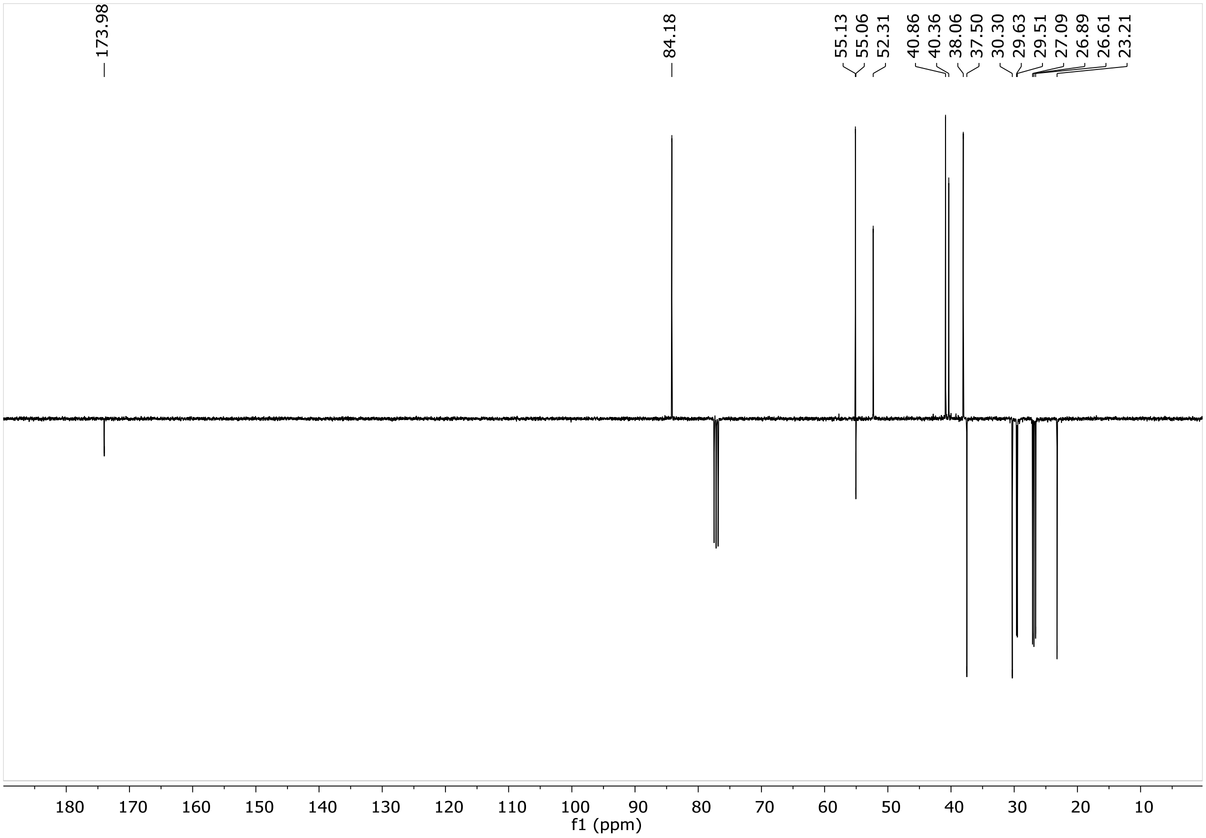
173.98
84.18
55.13 55.06 52.31 40.86 40.36 38.06 37.50 30.30 29.63 29.51 27.09 26.89 26.61 23.21
f1 (ppm)

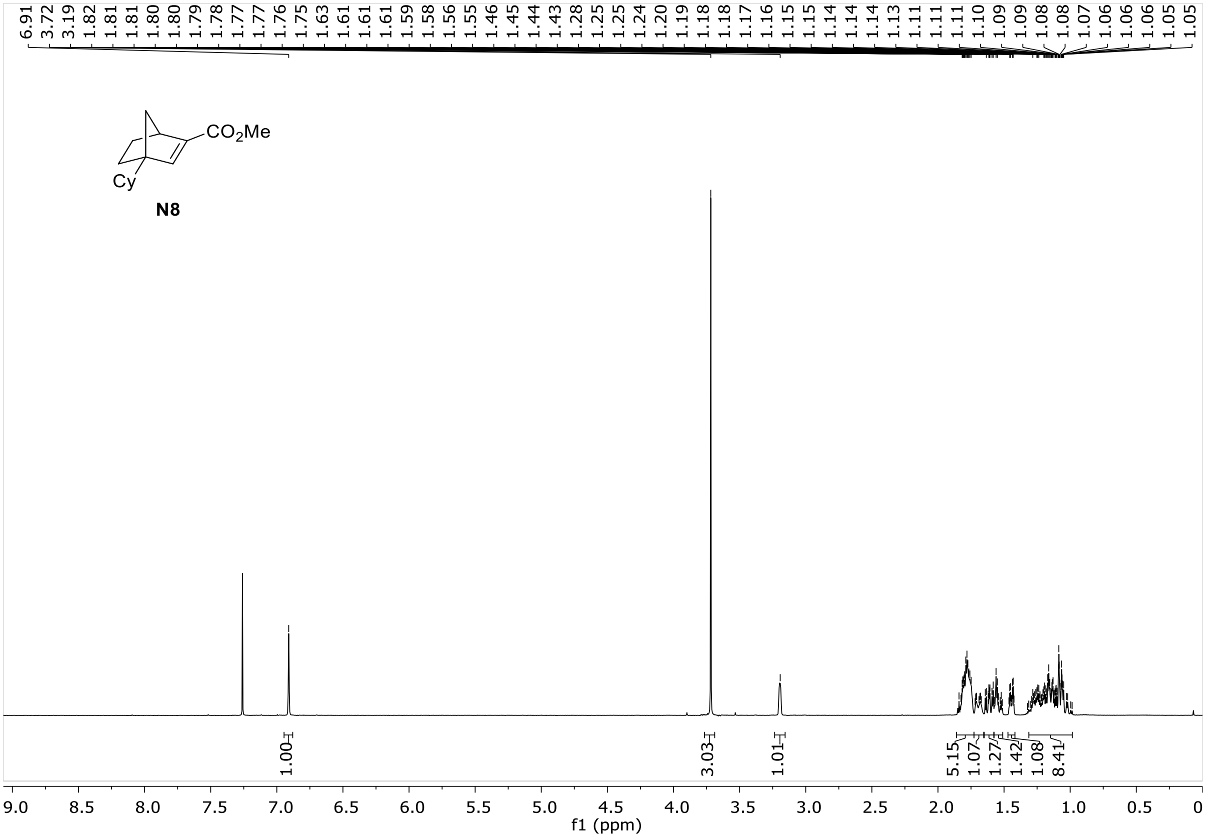
6.91
3.72
3.19
1.82
1.81
1.81
1.80
1.80
1.79
1.78
1.77
1.77
1.76
1.75
1.63
1.61
1.61
1.61
1.59
1.58
1.56
1.55
1.46
1.45
1.44
1.43
1.28
1.25
1.25
1.24
1.20
1.19
1.18
1.18
1.17
1.16
1.15
1.15
1.14
1.14
1.14
1.13
1.11
1.11
1.11
1.10
1.09
1.09
1.08
1.08
1.07
1.06
1.06
1.05
1.05
CO2Me
Cy
N8
1.00
3.03
1.01
5.15
1.07
1.27
1.42
1.08
8.41
9.0 8.5 8.0 7.5 7.0 6.5 6.0 5.5 5.0 4.5 4.0 3.5 3.0 2.5 2.0 1.5 1.0 0.5 0
f1 (ppm)

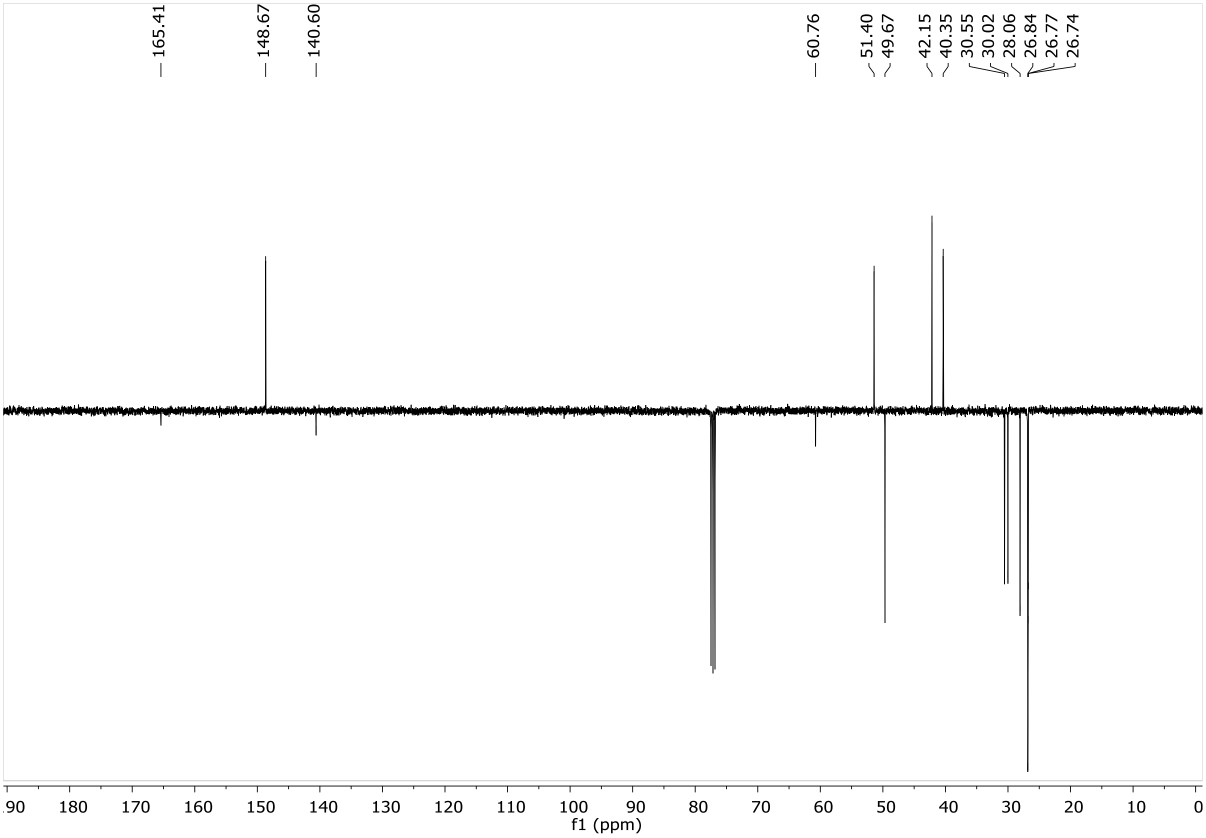
165.41
148.67
140.60
60.76
51.40
49.67
42.15
40.35
30.55
30.02
28.06
26.84
26.77
26.74
90 180 170 160 150 140 130 120 110 100 90 80 70 60 50 40 30 20 10 0
f1 (ppm)

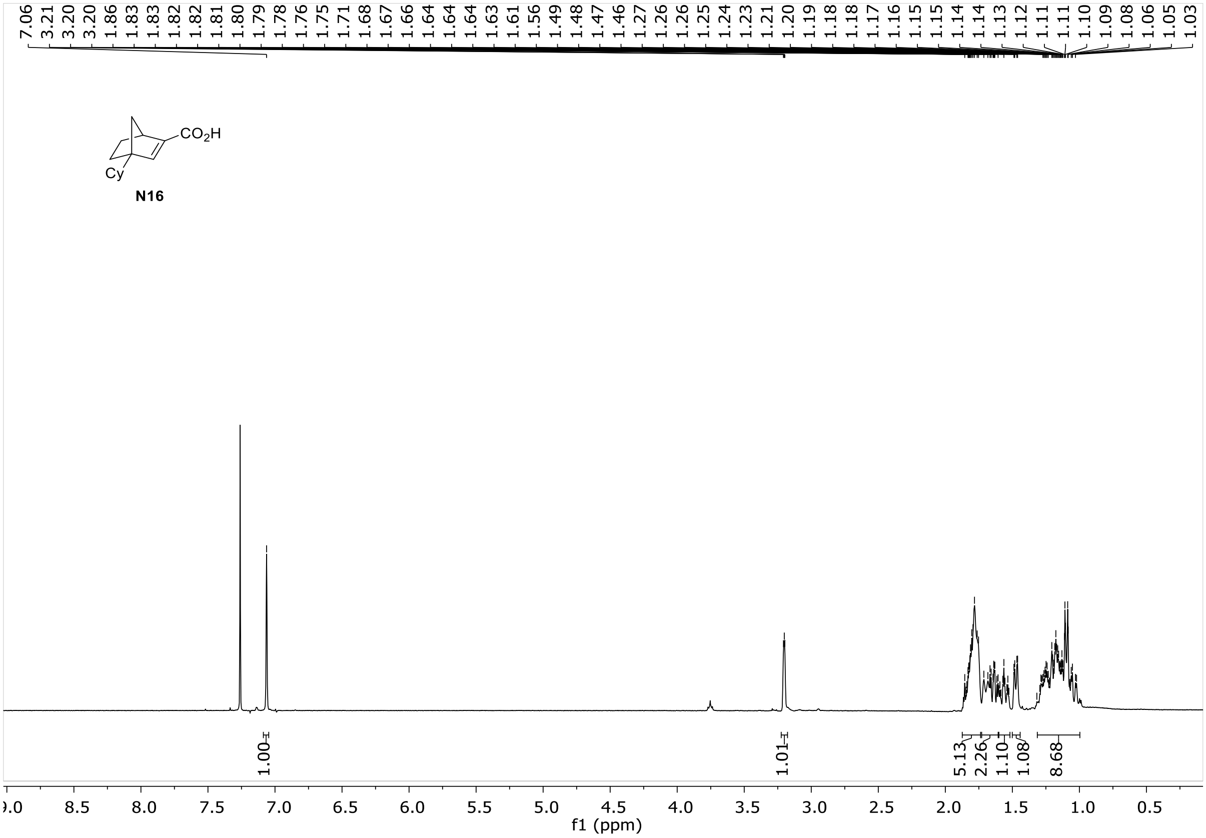
7.06
3.21
3.20
3.20
1.86
1.83
1.83
1.82
1.82
1.81
1.80
1.79
1.78
1.76
1.75
1.71
1.68
1.67
1.66
1.64
1.64
1.64
1.63
1.61
1.56
1.49
1.48
1.47
1.46
1.27
1.26
1.26
1.25
1.24
1.23
1.21
1.20
1.19
1.18
1.18
1.17
1.16
1.15
1.15
1.14
1.14
1.13
1.12
1.11
1.11
1.10
1.09
1.08
1.06
1.05
1.03
CO_2H
Cy
N16
1.00
1.01
5.13
2.26
1.10
1.08
8.68
9.0
8.5
8.0
7.5
7.0
6.5
6.0
5.5
5.0
4.5
4.0
3.5
3.0
2.5
2.0
1.5
1.0
0.5
f1 (ppm)

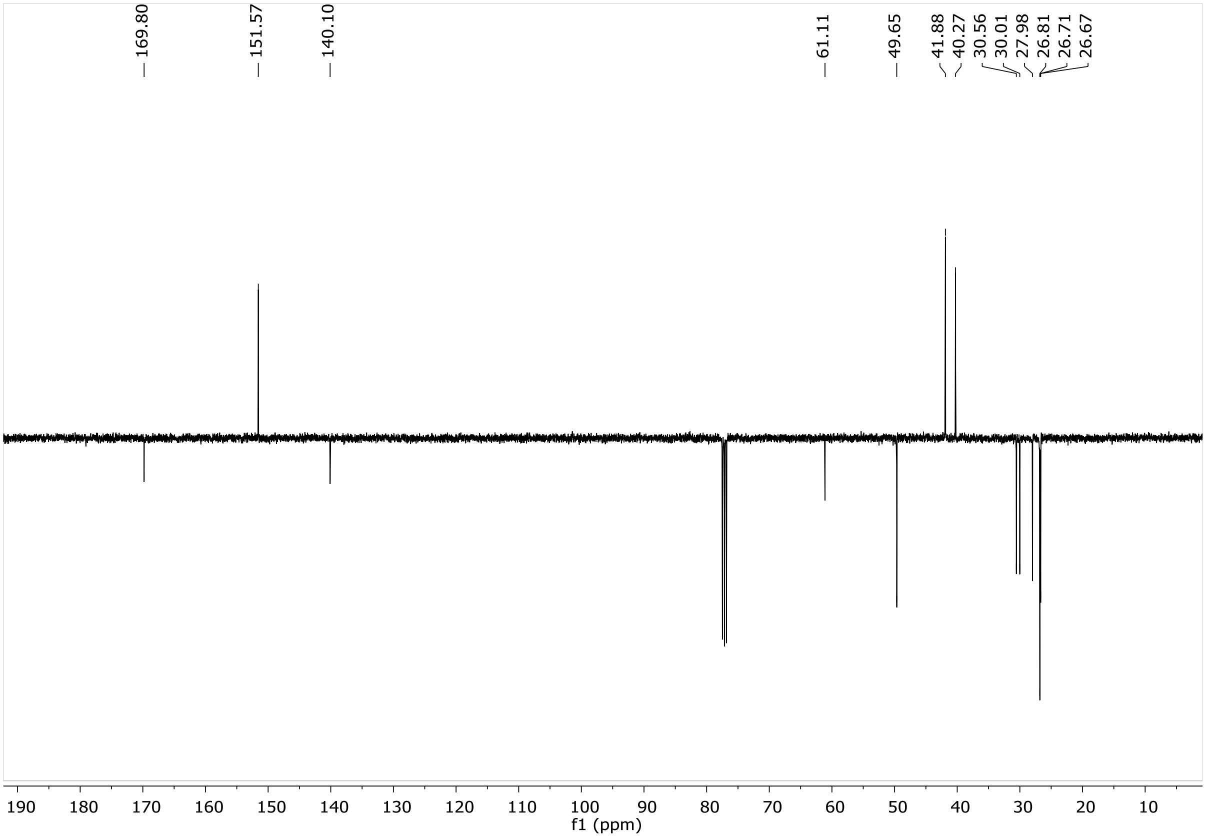
169.80
151.57
140.10
61.11
49.65
41.88
40.27
30.56
30.01
27.98
26.81
26.71
26.67
190
180
170
160
150
140
130
120
110
100
90
80
70
60
50
40
30
20
10
f1 (ppm)

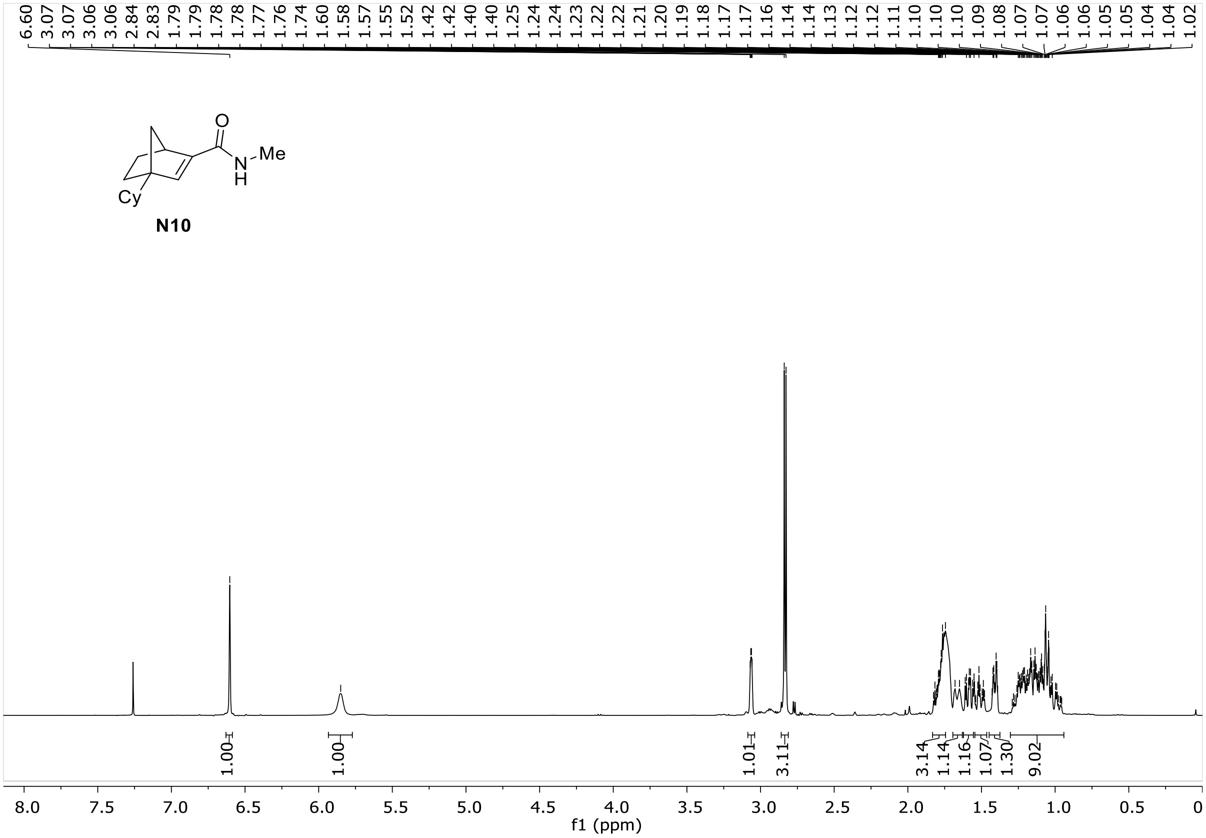
6.60 3.07 3.07 3.06 3.06 2.84 2.83 1.79 1.79 1.78 1.78 1.77 1.76 1.74 1.60 1.58 1.57 1.55 1.52 1.42 1.42 1.40 1.40 1.25 1.24 1.24 1.23 1.22 1.22 1.21 1.20 1.19 1.18 1.17 1.17 1.16 1.14 1.14 1.13 1.12 1.12 1.11 1.10 1.10 1.10 1.09 1.08 1.07 1.07 1.06 1.06 1.05 1.05 1.04 1.04 1.02
O
N
Me
H
Cy
N10
1.00
1.00
1.01
3.11
3.14
1.14
1.16
1.07
1.30
9.02
8.0 7.5 7.0 6.5 6.0 5.5 5.0 4.5 4.0 3.5 3.0 2.5 2.0 1.5 1.0 0.5 0
f1 (ppm)

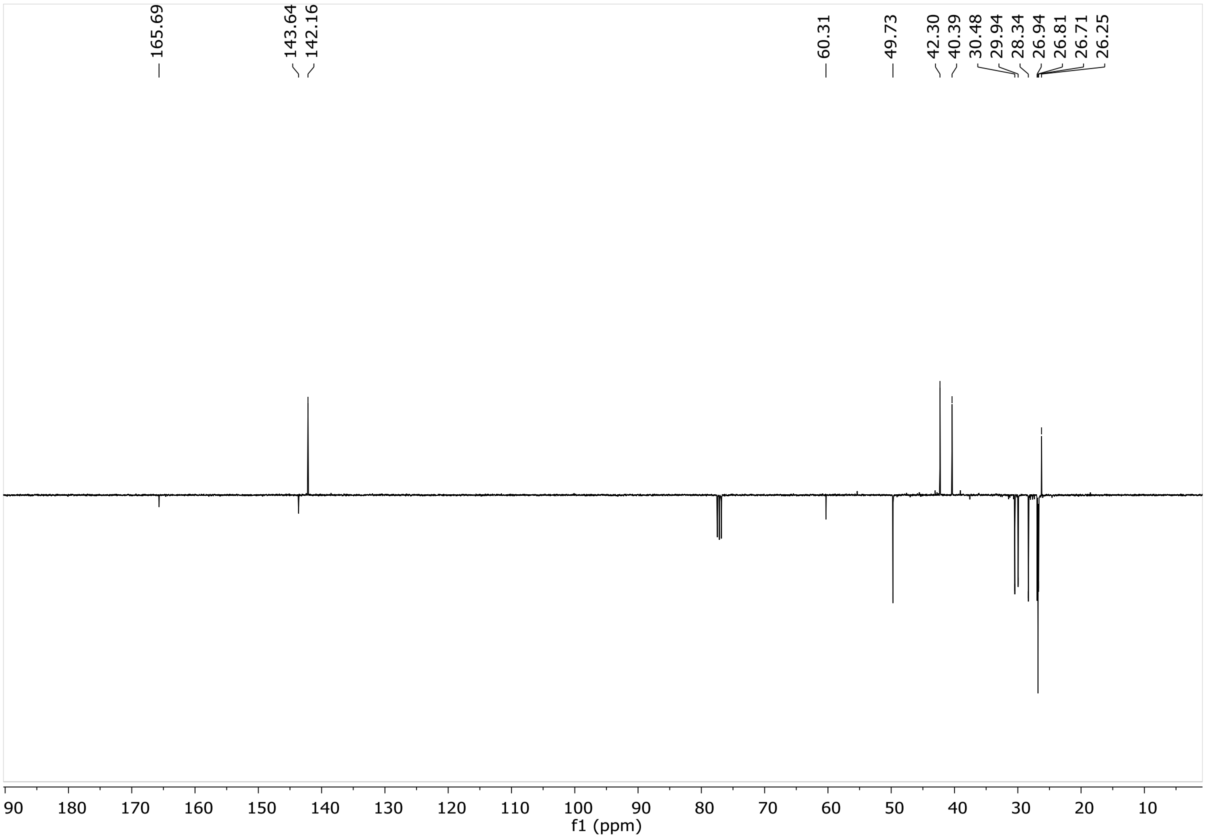
165.69
143.64
142.16
60.31
49.73
42.30
40.39
30.48
29.94
28.34
26.94
26.81
26.71
26.25
90 180 170 160 150 140 130 120 110 100 90 80 70 60 50 40 30 20 10
f1 (ppm)

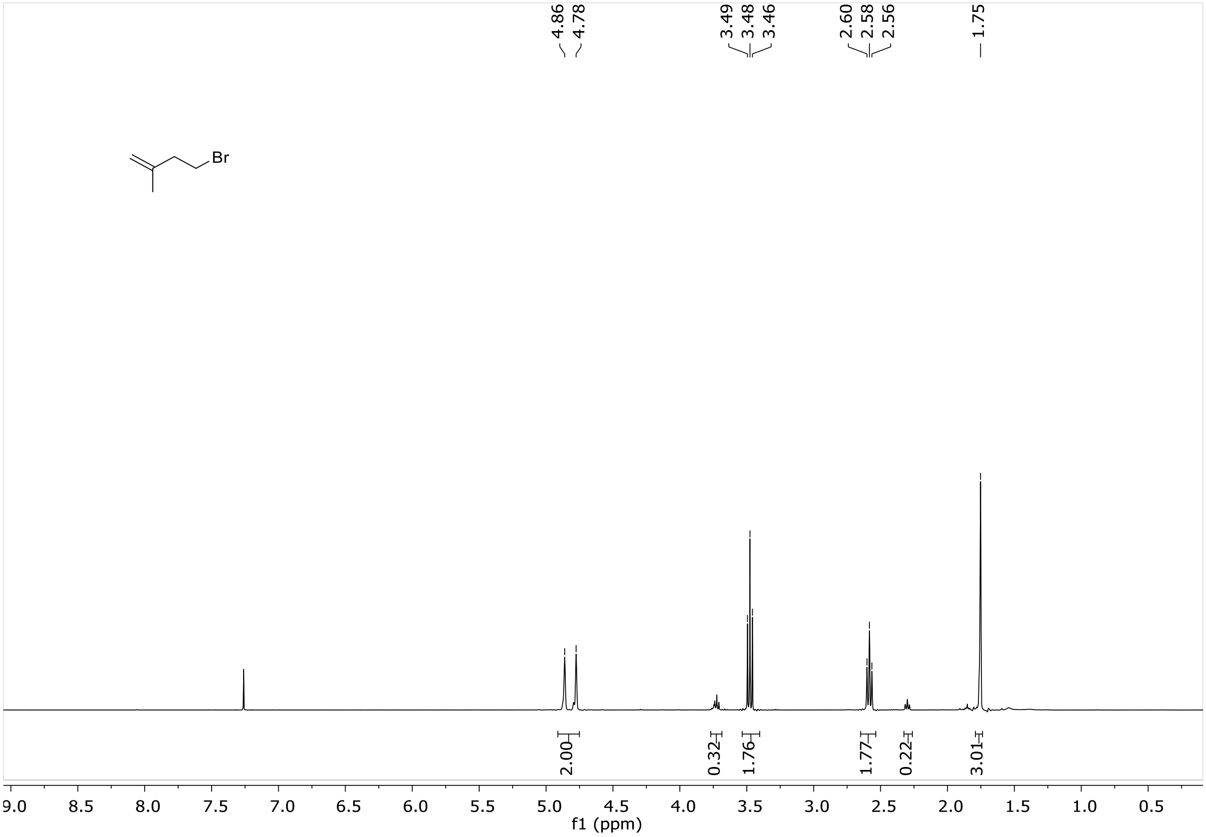
4.86
4.78
3.49
3.48
3.46
2.60
2.58
2.56
1.75
Br
2.00
0.32
1.76
1.77
0.22
3.01
9.0 8.5 8.0 7.5 7.0 6.5 6.0 5.5 5.0 4.5 4.0 3.5 3.0 2.5 2.0 1.5 1.0 0.5
f1 (ppm)

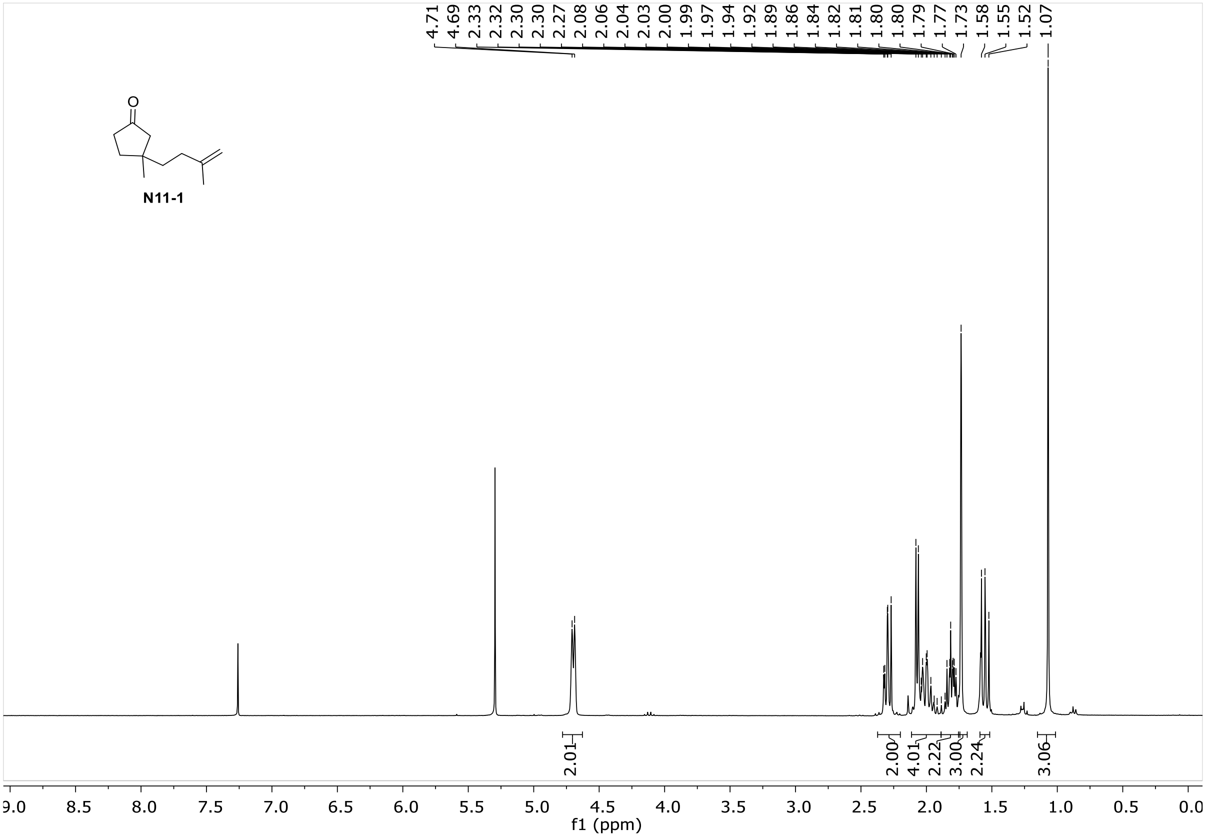
4.71
4.69
2.33
2.32
2.30
2.30
2.27
2.08
2.06
2.04
2.03
2.00
1.99
1.97
1.94
1.92
1.89
1.86
1.84
1.82
1.81
1.80
1.80
1.79
1.77
1.73
1.58
1.55
1.52
1.07
O
N11-1
2.01
2.00
4.01
2.22
3.00
2.24
3.06
9.0 8.5 8.0 7.5 7.0 6.5 6.0 5.5 5.0 4.5 4.0 3.5 3.0 2.5 2.0 1.5 1.0 0.5 0.0
f1 (ppm)

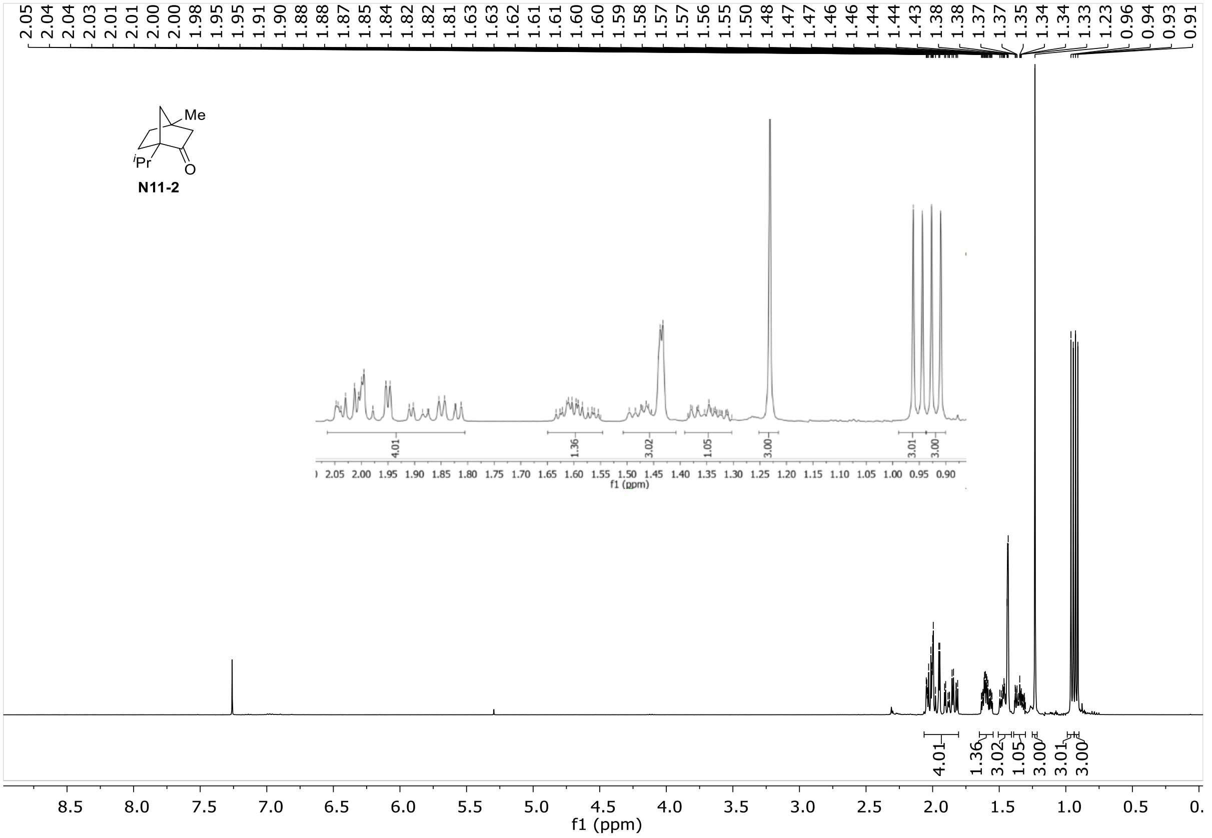
2.05 2.04 2.04 2.03 2.01 2.01 2.00 2.00 1.98 1.95 1.95 1.91 1.90 1.88 1.88 1.87 1.85 1.84 1.82 1.82 1.81 1.63 1.63 1.62 1.61 1.61 1.60 1.60 1.59 1.58 1.57 1.57 1.56 1.55 1.50 1.48 1.47 1.47 1.46 1.46 1.44 1.44 1.43 1.38 1.38 1.37 1.37 1.35 1.34 1.34 1.33 1.23 0.96 0.94 0.93 0.91
Me
iPr
O
N11-2
4.01
1.36
3.02
1.05
3.00
3.01
3.00
f1 (ppm)

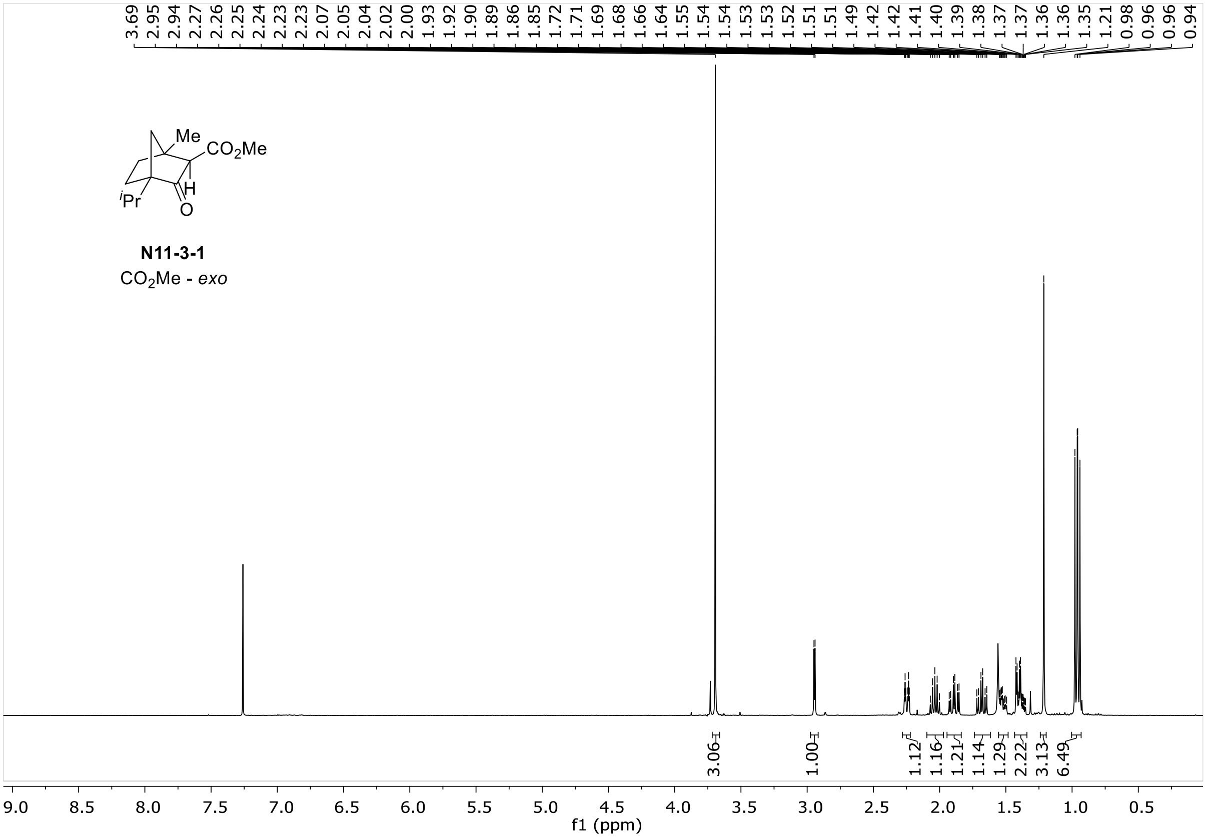
3.69
2.95
2.94
2.27
2.26
2.25
2.24
2.23
2.23
2.07
2.05
2.04
2.02
2.00
1.93
1.92
1.90
1.89
1.86
1.85
1.72
1.71
1.69
1.68
1.66
1.64
1.55
1.54
1.54
1.53
1.53
1.52
1.51
1.51
1.49
1.42
1.42
1.41
1.40
1.39
1.38
1.37
1.37
1.36
1.36
1.35
1.21
0.98
0.96
0.94
Me
CO_2Me
H
^{i}Pr
O
N11-3-1
CO_2Me - exo
3.06
1.00
1.12
1.16
1.21
1.14
1.29
2.22
3.13
6.49
9.0
8.5
8.0
7.5
7.0
6.5
6.0
5.5
5.0
4.5
4.0
3.5
3.0
2.5
2.0
1.5
1.0
0.5
f1 (ppm)

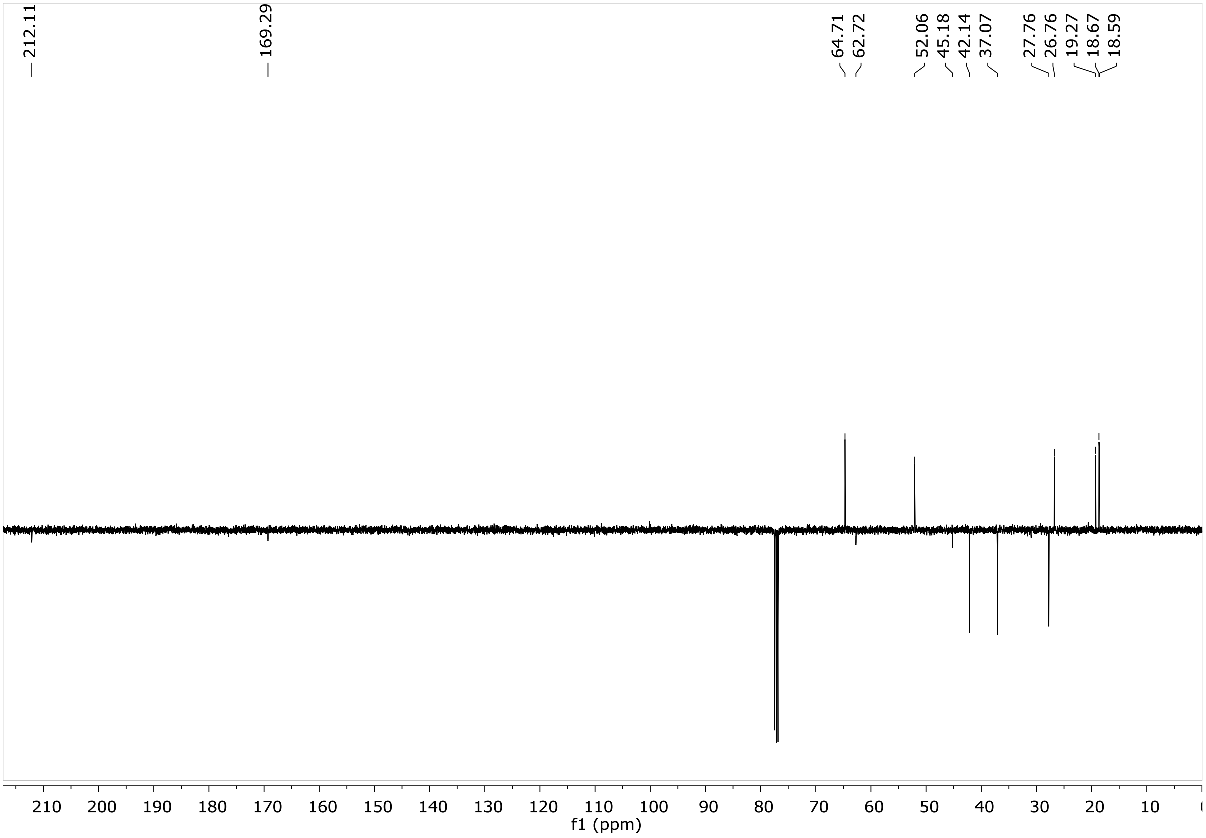
212.11
169.29
64.71
62.72
52.06
45.18
42.14
37.07
27.76
26.76
19.27
18.67
18.59
210
200
190
180
170
160
150
140
130
120
110
100
90
80
70
60
50
40
30
20
10
f1 (ppm)

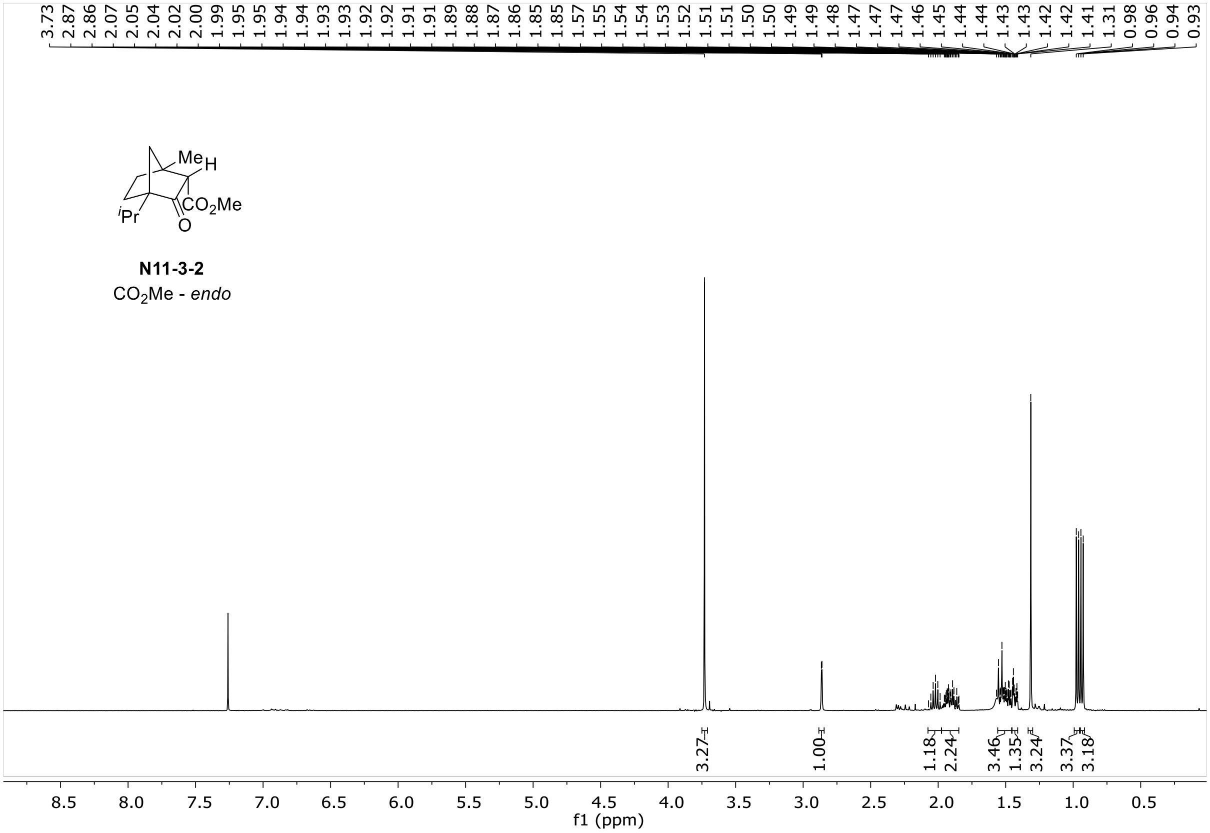
3.73 2.87 2.86 2.07 2.05 2.04 2.02 2.00 1.99 1.95 1.95 1.94 1.94 1.93 1.93 1.92 1.92 1.91 1.91 1.89 1.88 1.87 1.86 1.85 1.85 1.57 1.55 1.54 1.54 1.53 1.52 1.51 1.51 1.50 1.50 1.49 1.49 1.48 1.47 1.47 1.47 1.46 1.45 1.44 1.44 1.43 1.43 1.42 1.42 1.41 1.31 0.98 0.96 0.94 0.93
Me
H
CO2Me
iPr
O
N11-3-2
CO2Me - endo
3.27
1.00
1.18
2.24
3.46
1.35
3.24
3.37
3.18
8.5 8.0 7.5 7.0 6.5 6.0 5.5 5.0 4.5 4.0 3.5 3.0 2.5 2.0 1.5 1.0 0.5
f1 (ppm)

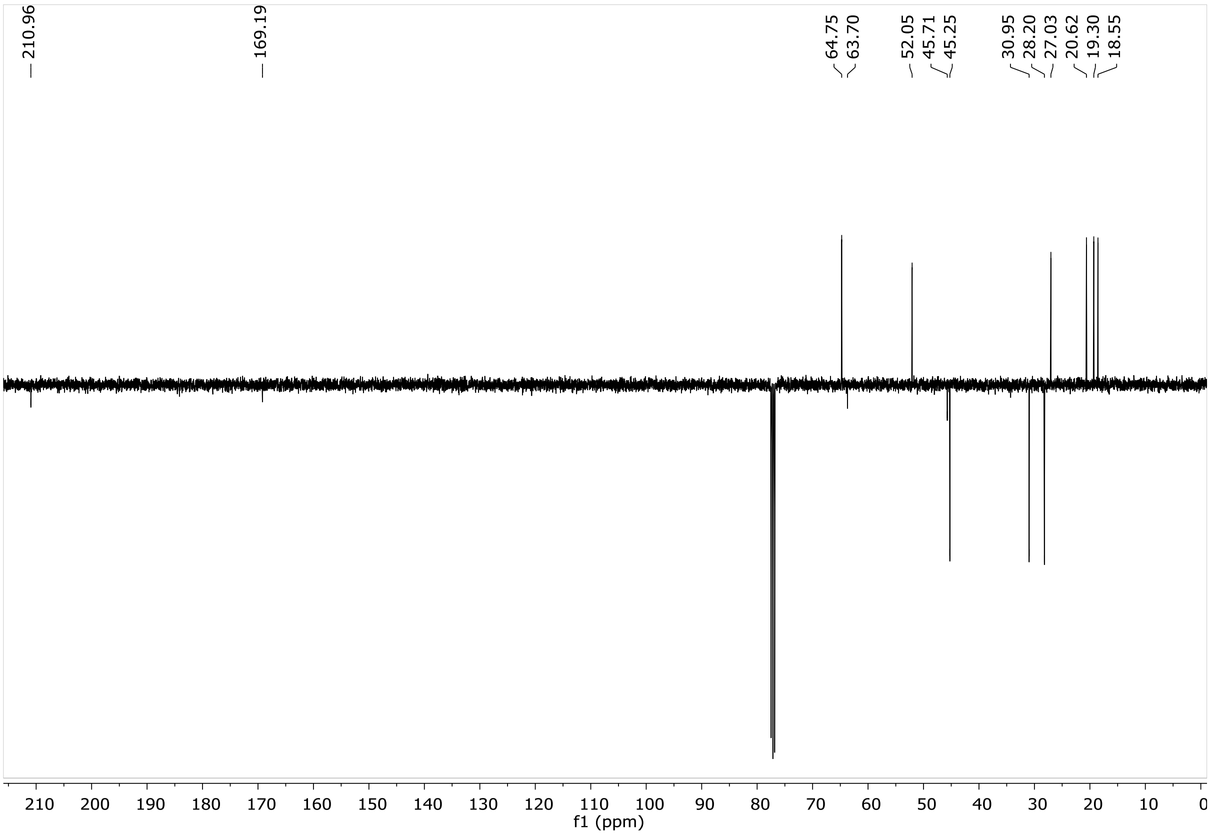
210.96
169.19
64.75
63.70
52.05
45.71
45.25
30.95
28.20
27.03
20.62
19.30
18.55
210 200 190 180 170 160 150 140 130 120 110 100 90 80 70 60 50 40 30 20 10 0
f1 (ppm)

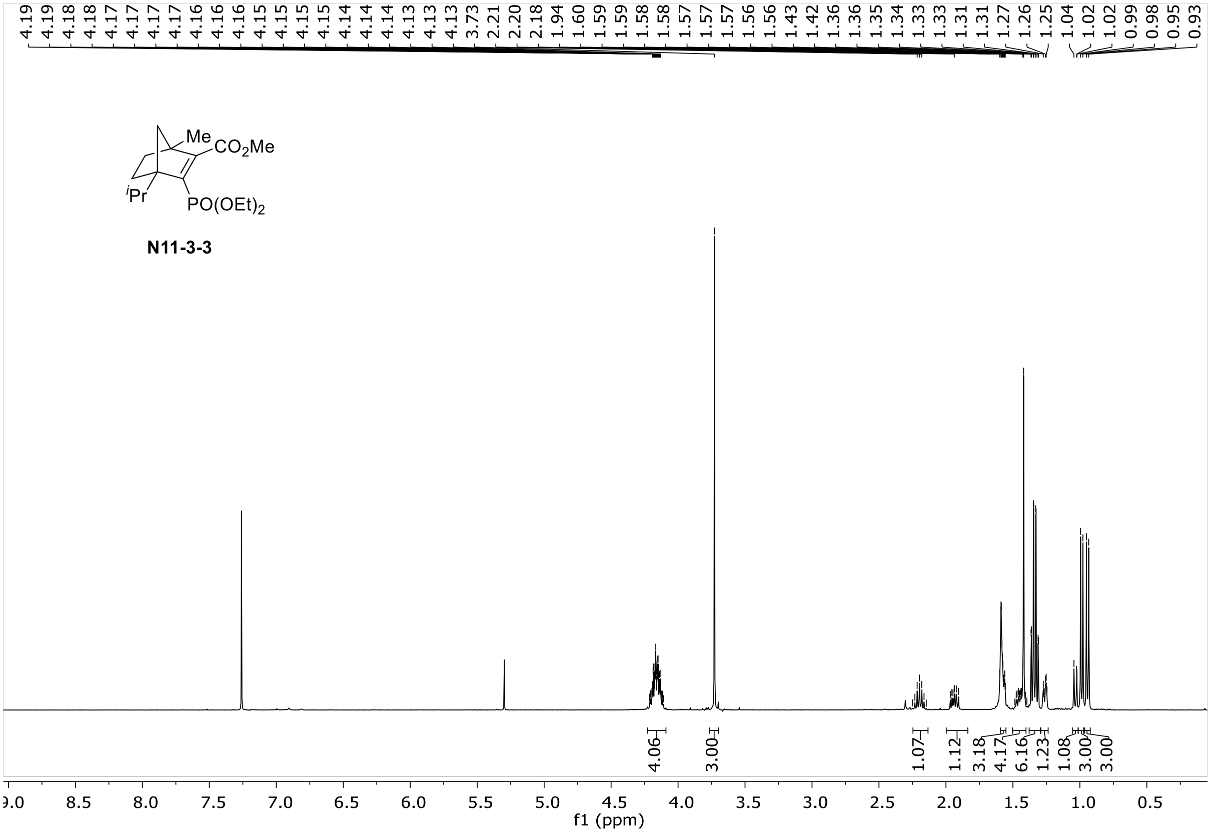
4.19 4.19 4.18 4.18 4.17 4.17 4.17 4.17 4.16 4.16 4.15 4.15 4.15 4.15 4.14 4.14 4.14 4.13 4.13 4.13 4.13 3.73 2.21 2.20 2.18 1.94 1.60 1.59 1.59 1.58 1.58 1.57 1.57 1.57 1.56 1.56 1.43 1.42 1.36 1.36 1.35 1.34 1.33 1.33 1.31 1.31 1.27 1.26 1.25 1.04 1.02 1.02 0.99 0.98 0.95 0.93
Me
CO2Me
iPr
PO(OEt)2
N11-3-3
4.06
3.00
1.07
1.12
3.18
4.17
6.16
1.23
1.08
3.00
3.00
f1 (ppm)

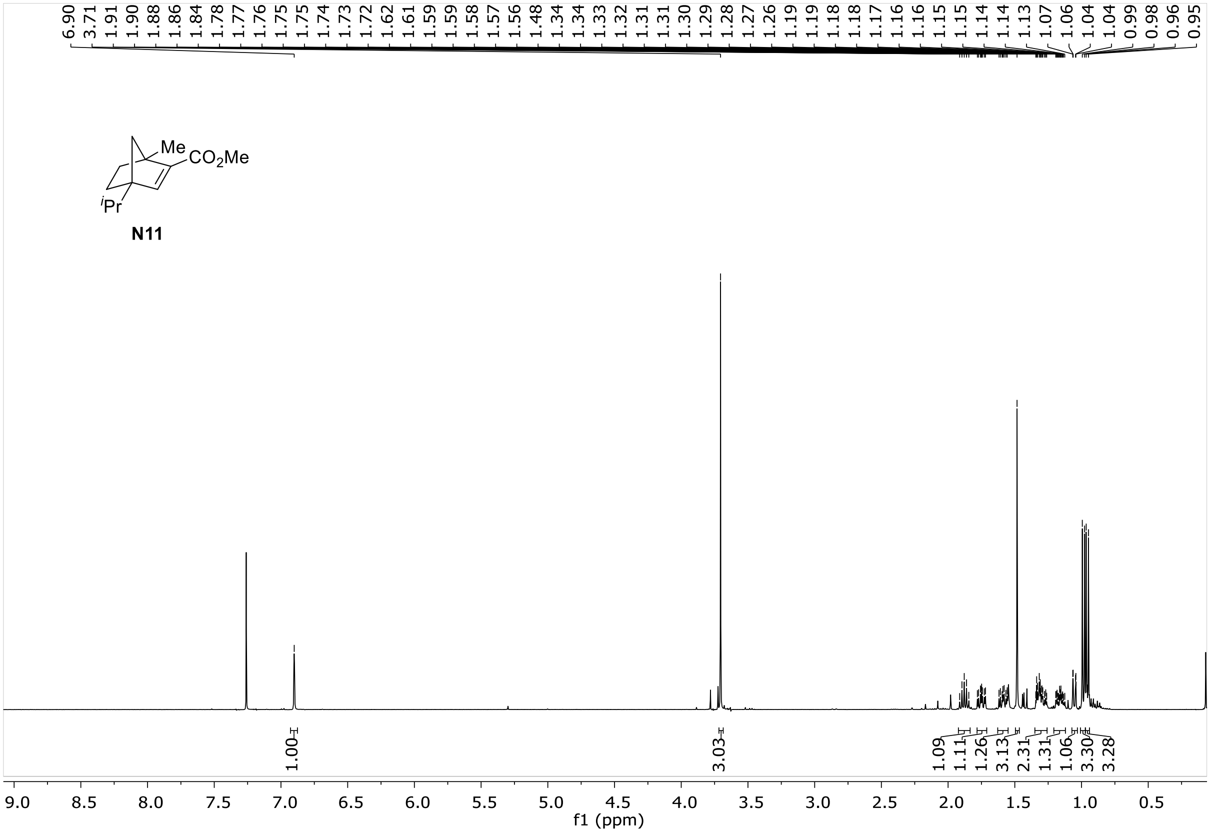
6.90
3.71
1.91
1.90
1.88
1.86
1.84
1.78
1.77
1.76
1.75
1.75
1.74
1.73
1.72
1.62
1.61
1.59
1.59
1.58
1.57
1.56
1.48
1.34
1.34
1.33
1.32
1.31
1.31
1.30
1.29
1.28
1.27
1.26
1.19
1.19
1.18
1.18
1.17
1.16
1.16
1.15
1.15
1.14
1.14
1.13
1.07
1.06
1.04
1.04
0.99
0.98
0.96
0.95
Me
CO2Me
iPr
N11
1.00
3.03
1.09
1.11
1.26
3.13
2.31
1.31
1.06
3.30
3.28
9.0 8.5 8.0 7.5 7.0 6.5 6.0 5.5 5.0 4.5 4.0 3.5 3.0 2.5 2.0 1.5 1.0 0.5
f1 (ppm)

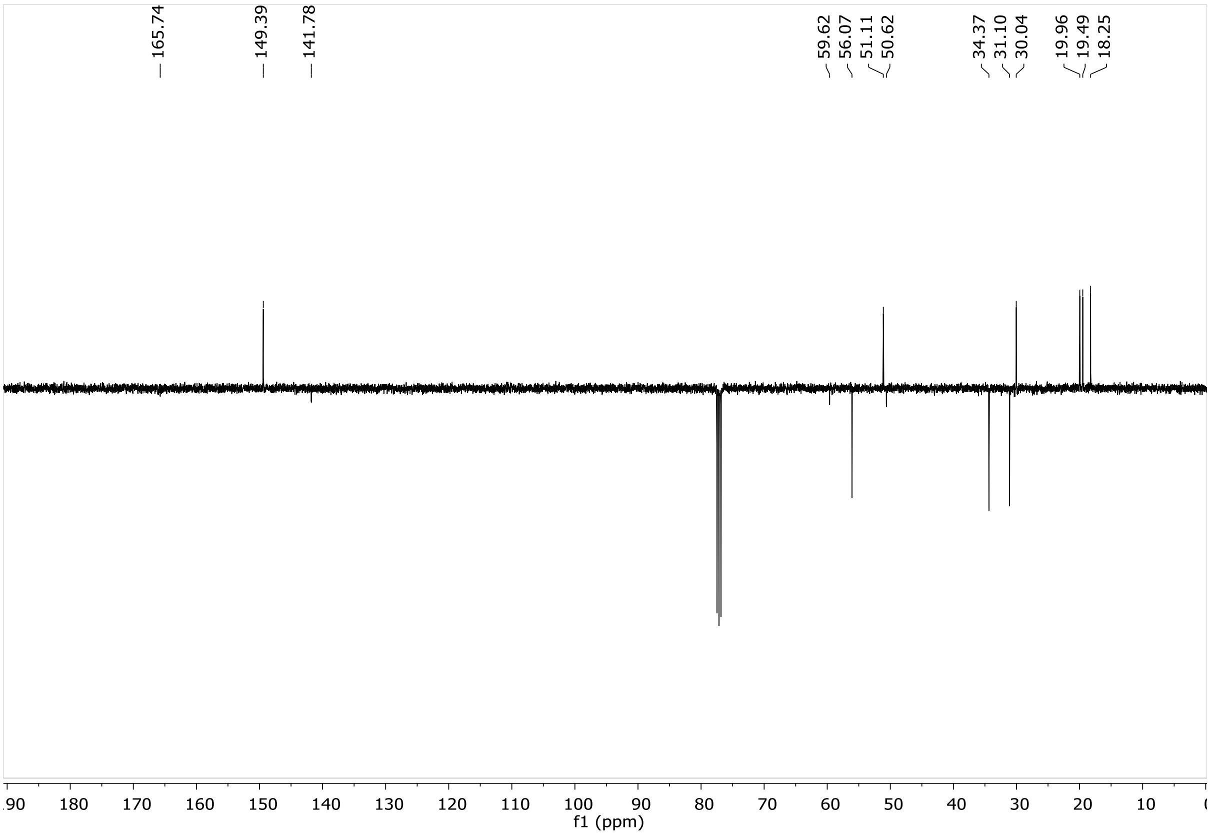
165.74
149.39
141.78
59.62
56.07
51.11
50.62
34.37
31.10
30.04
19.96
19.49
18.25
190 180 170 160 150 140 130 120 110 100 90 80 70 60 50 40 30 20 10 0
f1 (ppm)

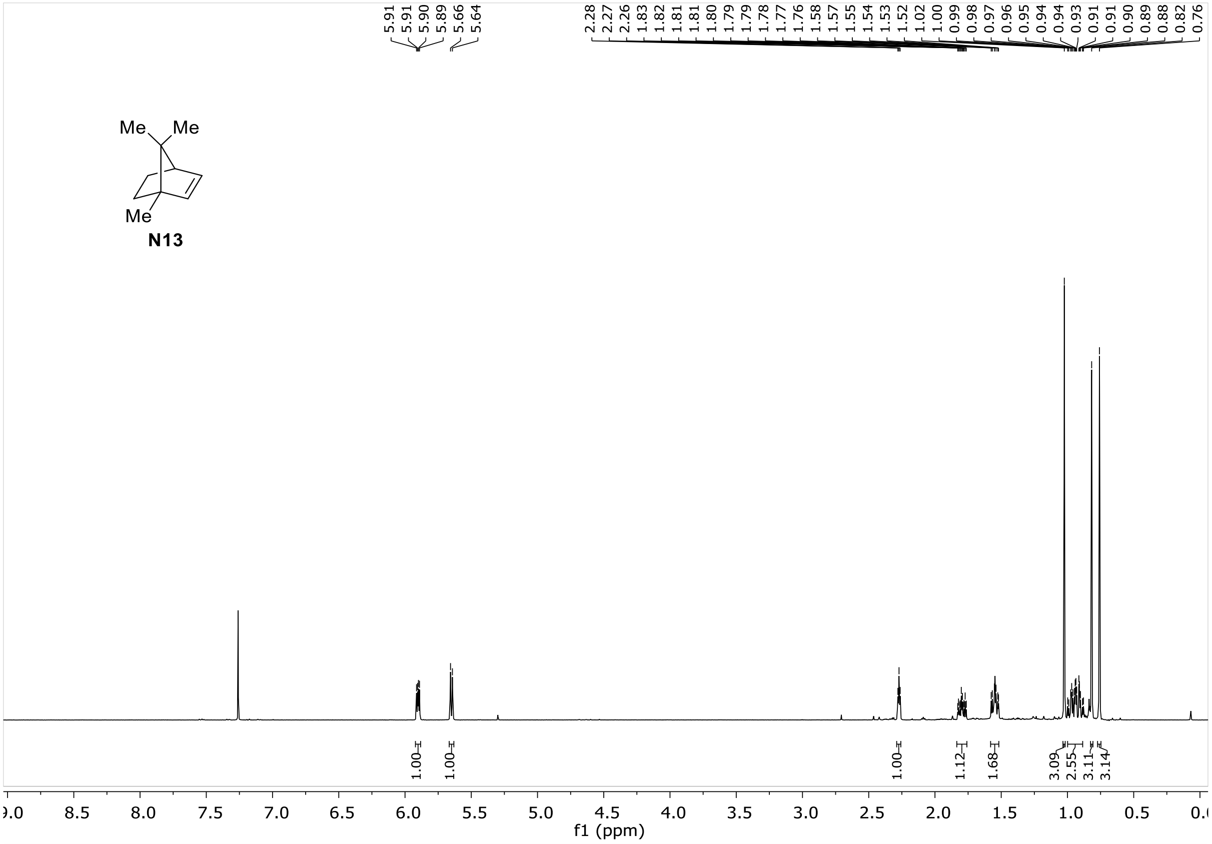
5.91
5.91
5.90
5.89
5.66
5.64
2.28
2.27
2.26
1.83
1.82
1.81
1.81
1.80
1.79
1.79
1.78
1.77
1.76
1.58
1.57
1.55
1.54
1.53
1.52
1.02
1.00
0.99
0.98
0.97
0.96
0.95
0.94
0.94
0.93
0.91
0.91
0.90
0.89
0.88
0.82
0.76
Me
Me
Me
N13
1.00
1.00
1.00
1.12
1.68
3.09
2.55
3.11
3.14
8.5
8.0
7.5
7.0
6.5
6.0
5.5
5.0
4.5
4.0
3.5
3.0
2.5
2.0
1.5
1.0
0.5
f1 (ppm)

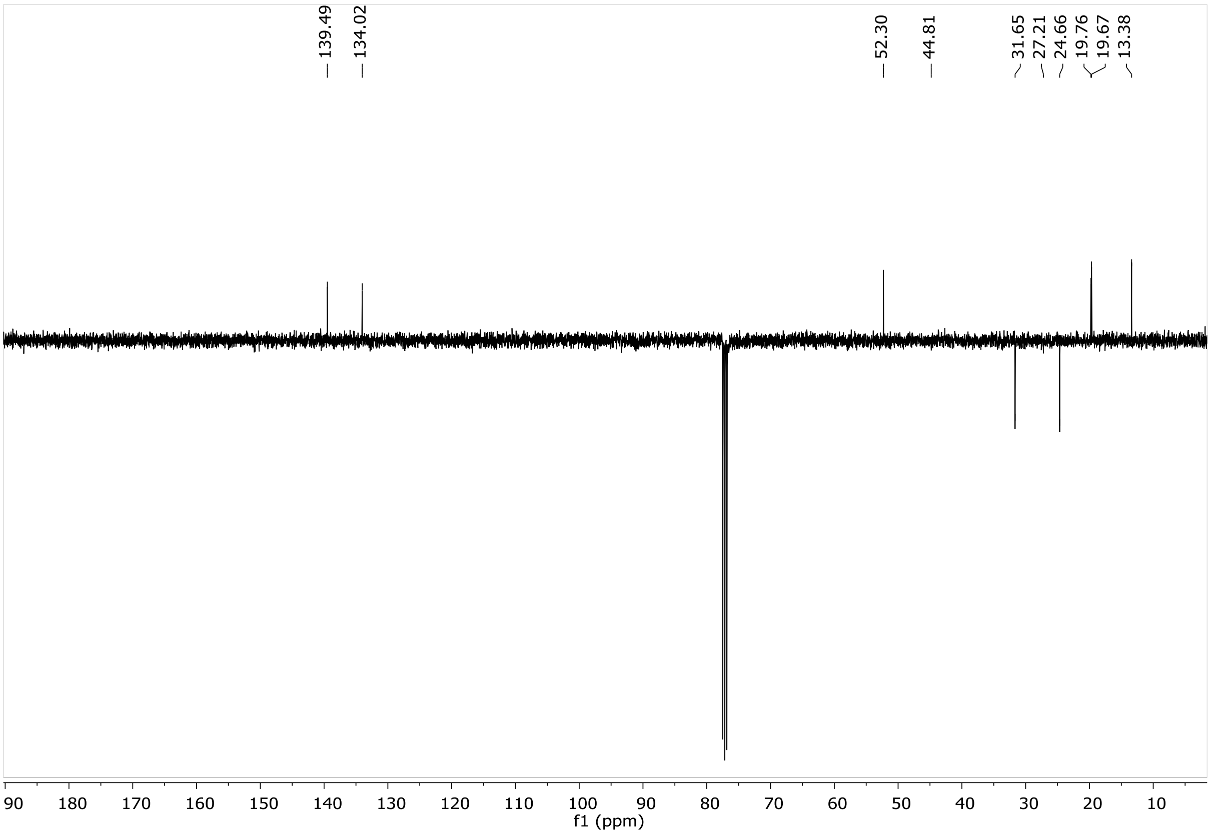
139.49
134.02
52.30
44.81
31.65
27.21
24.66
19.76
19.67
13.38
180
170
160
150
140
130
120
110
100
90
80
70
60
50
40
30
20
10
f1 (ppm)

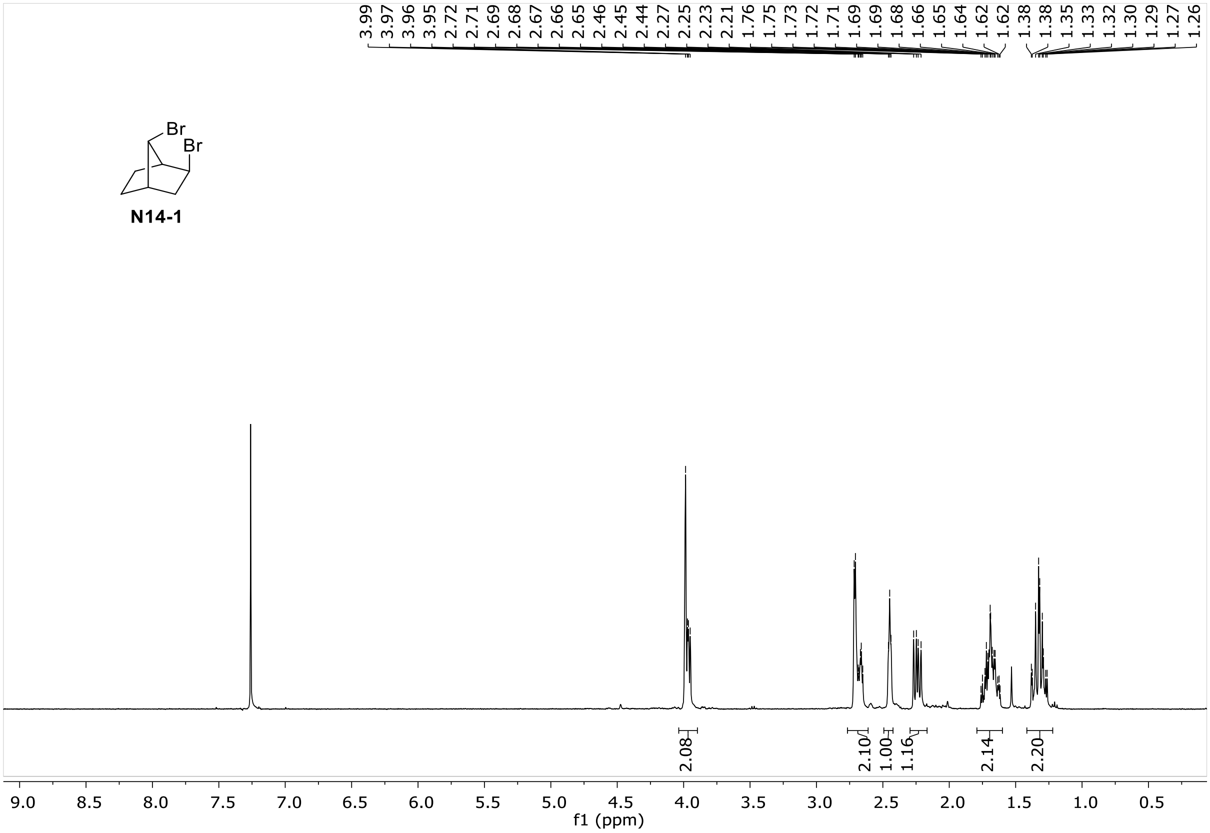
3.99 3.97 3.96 3.95 2.72 2.71 2.69 2.68 2.67 2.66 2.65 2.46 2.45 2.44 2.27 2.25 2.23 2.21 1.76 1.75 1.73 1.72 1.71 1.69 1.69 1.68 1.66 1.65 1.64 1.62 1.62 1.38 1.38 1.35 1.33 1.32 1.30 1.29 1.27 1.26
Br
Br
N14-1
2.08
2.10
1.00
1.16
2.14
2.20
9.0 8.5 8.0 7.5 7.0 6.5 6.0 5.5 5.0 4.5 4.0 3.5 3.0 2.5 2.0 1.5 1.0 0.5
f1 (ppm)

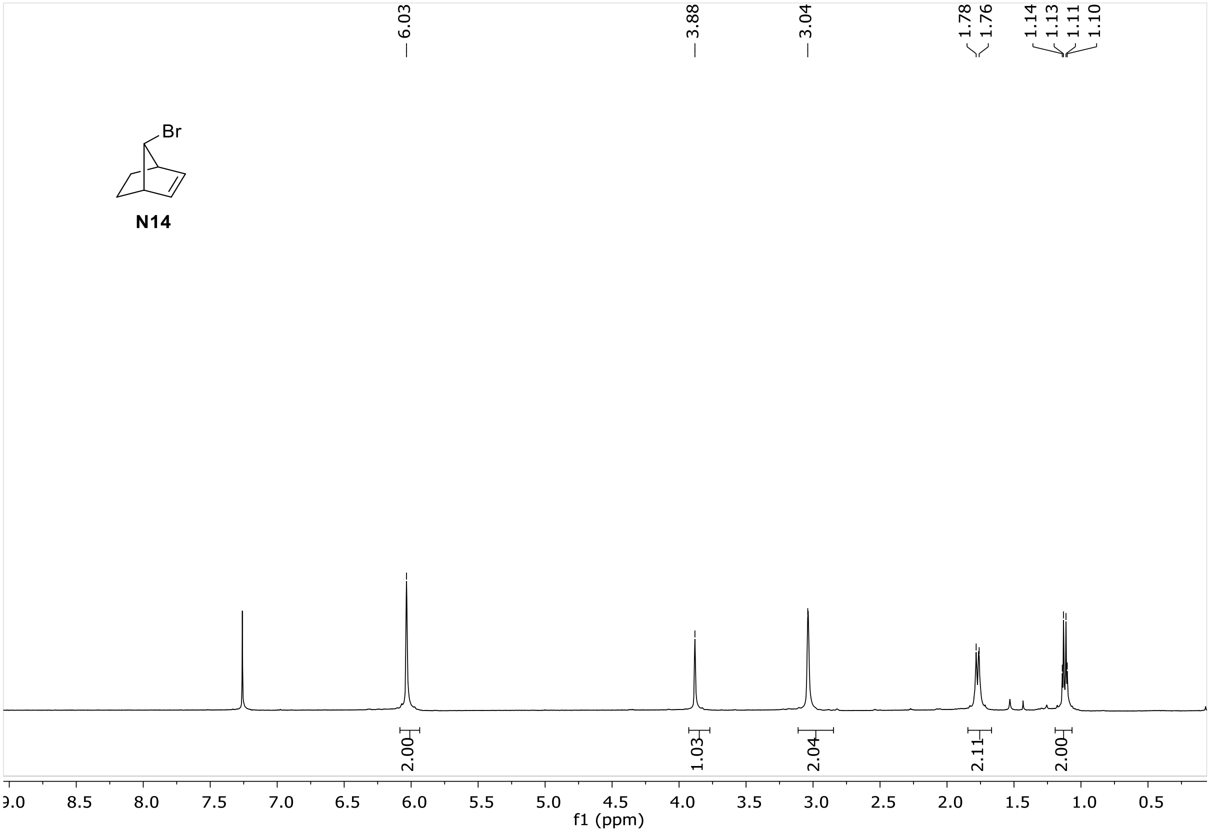
6.03
3.88
3.04
1.78 1.76
1.14 1.13 1.11 1.10
Br
N14
2.00
1.03
2.04
2.11
2.00
9.0 8.5 8.0 7.5 7.0 6.5 6.0 5.5 5.0 4.5 4.0 3.5 3.0 2.5 2.0 1.5 1.0 0.5
f1 (ppm)

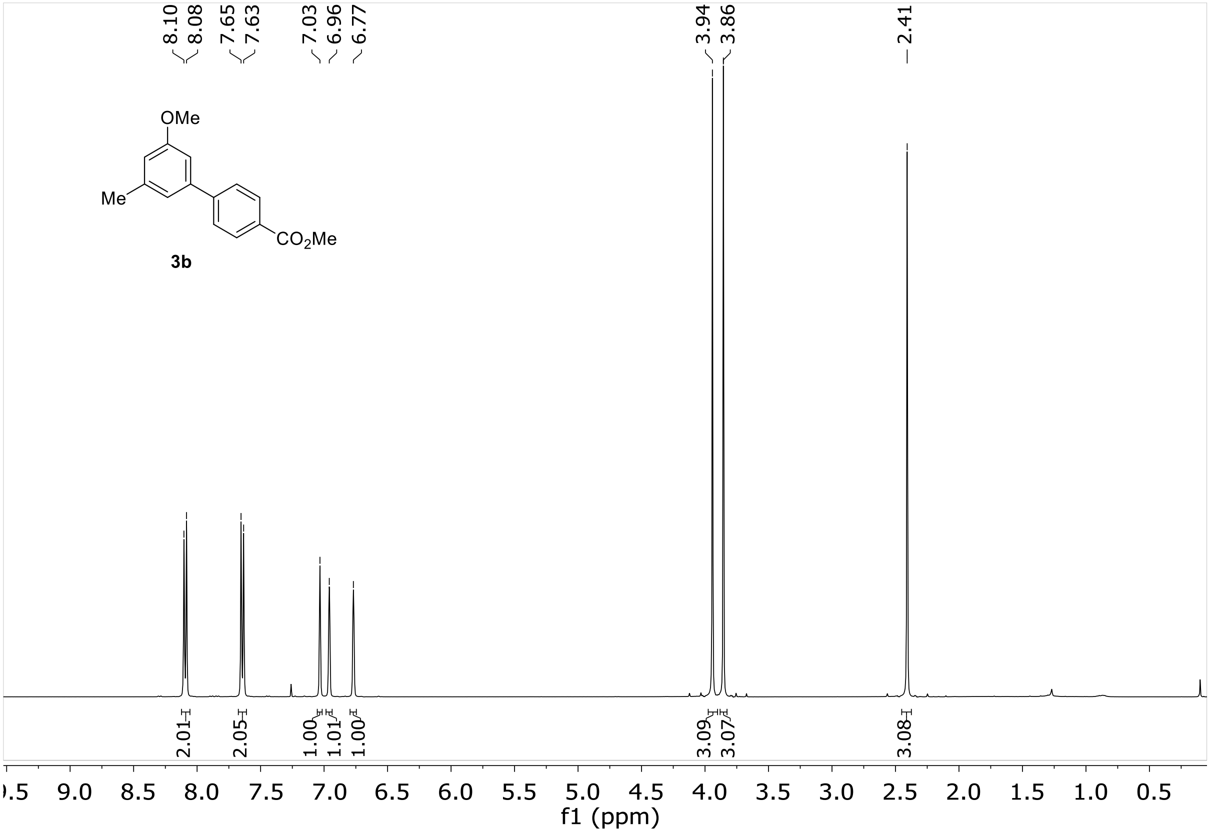
8.10
8.08
7.65
7.63
7.03
6.96
6.77
3.94
3.86
2.41
OMe
Me
CO2Me
3b
2.01
2.05
1.00
1.01
1.00
3.09
3.07
3.08
f1 (ppm)

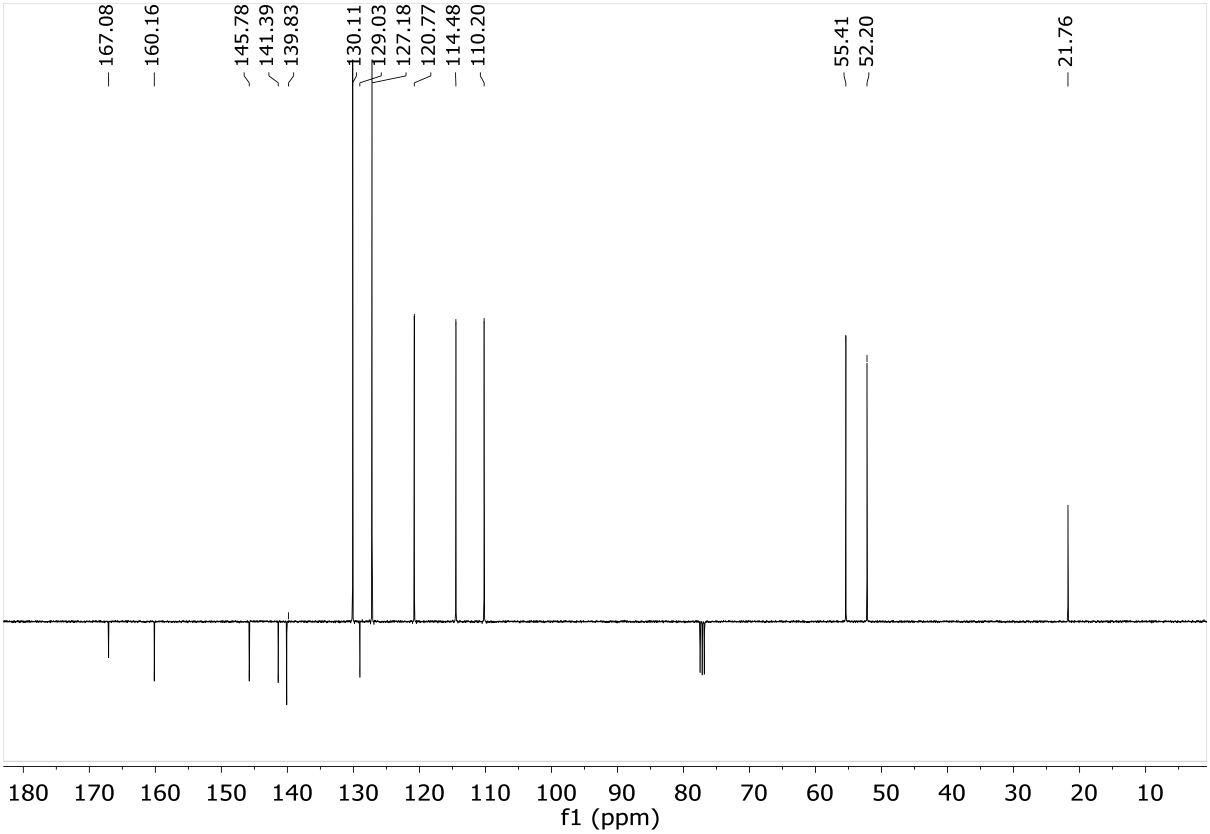
167.08
160.16
145.78
141.39
139.83
130.11
129.03
127.18
120.77
114.48
110.20
55.41
52.20
21.76
f1 (ppm)

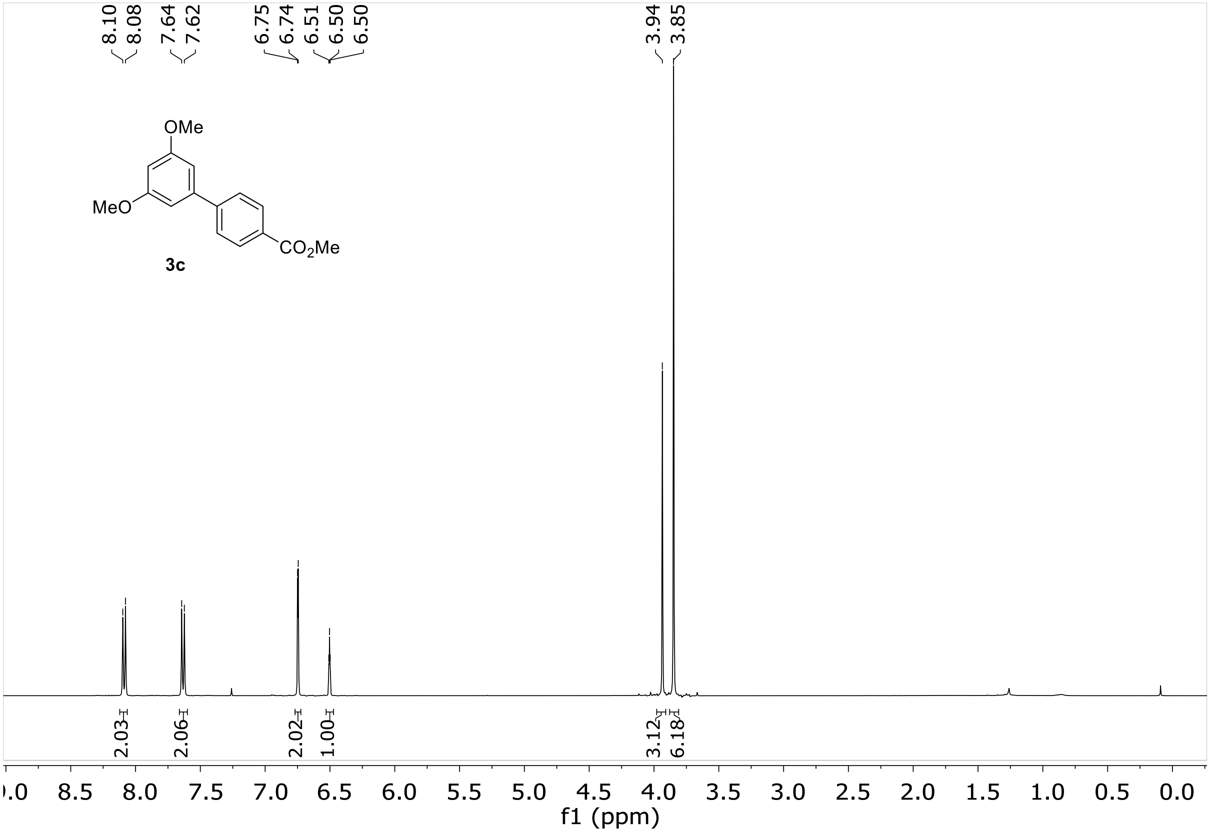
8.10
8.08
7.64
7.62
6.75
6.74
6.51
6.50
6.50
3.94
3.85
OMe
MeO
CO2Me
3c
2.03
2.06
2.02
1.00
3.12
6.18
f1 (ppm)

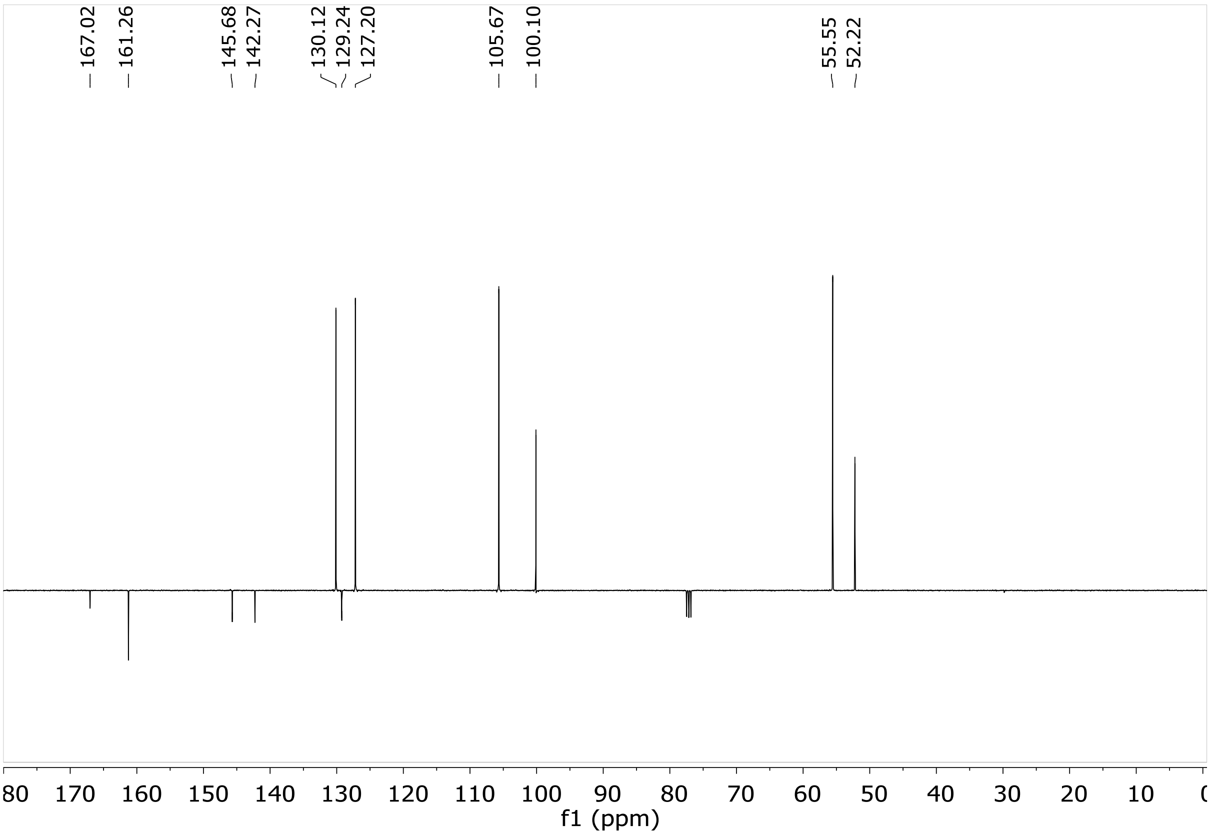
167.02
161.26
145.68
142.27
130.12
129.24
127.20
105.67
100.10
55.55
52.22
f1 (ppm)

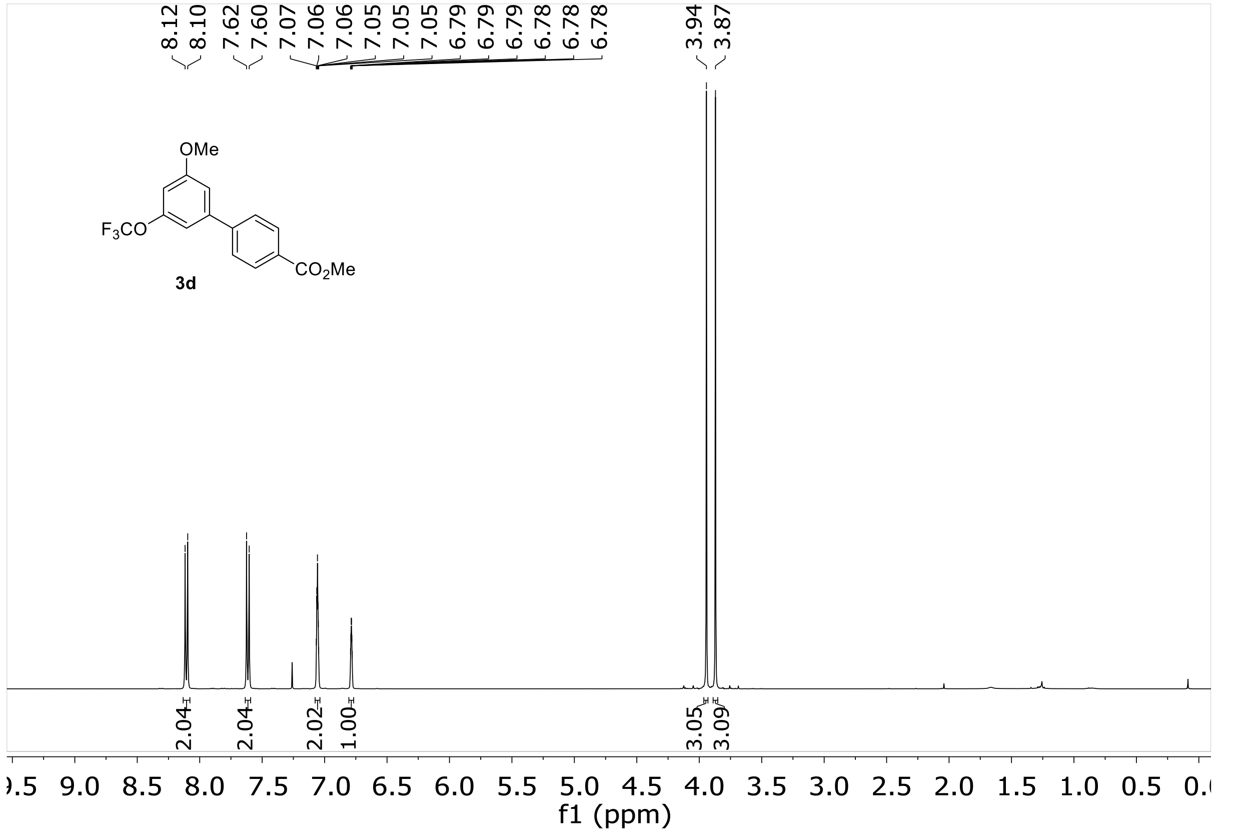
8.12
8.10
7.62
7.60
7.07
7.06
7.06
7.05
7.05
7.05
6.79
6.79
6.79
6.78
6.78
6.78
3.94
3.87
OMe
F3CO
CO2Me
3d
2.04
2.04
2.02
1.00
3.05
3.09
9.5 9.0 8.5 8.0 7.5 7.0 6.5 6.0 5.5 5.0 4.5 4.0 3.5 3.0 2.5 2.0 1.5 1.0 0.5 0.0
f1 (ppm)

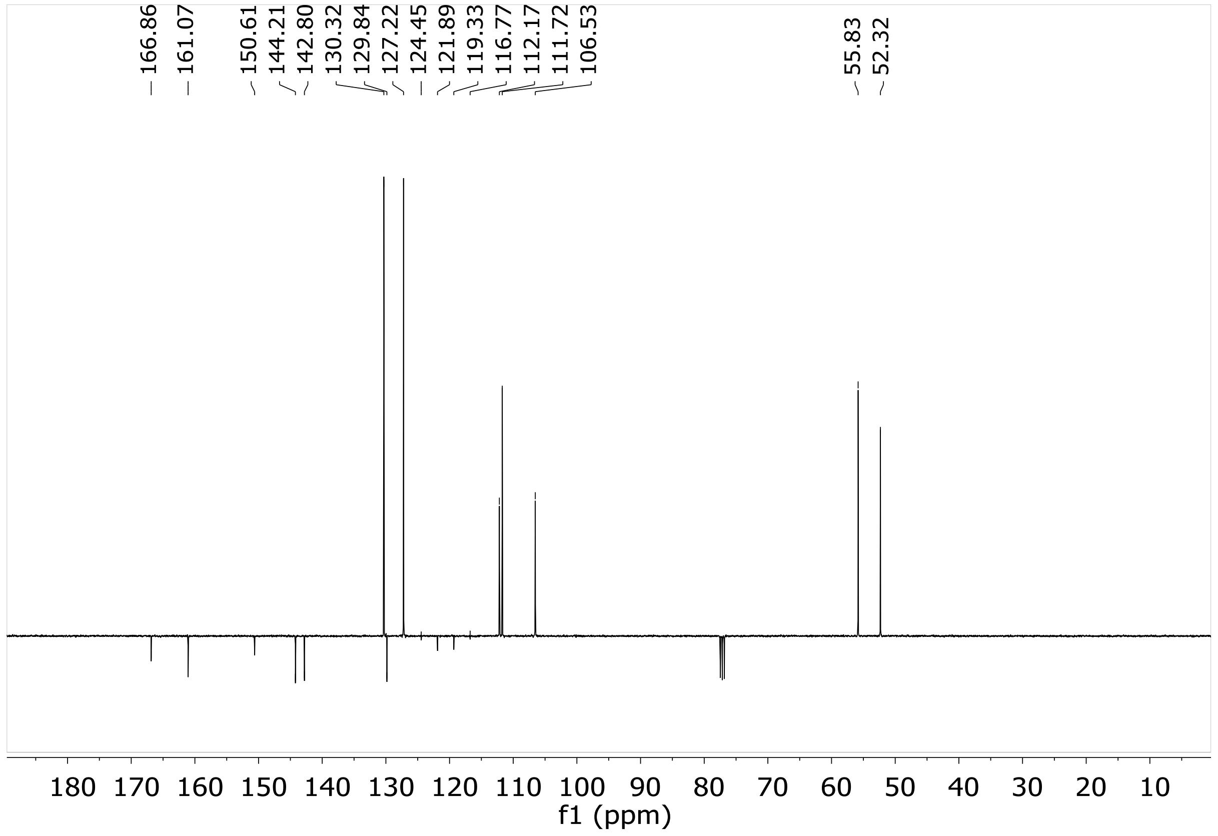
166.86
161.07
150.61
144.21
142.80
130.32
129.84
127.22
124.45
121.89
119.33
116.77
112.17
111.72
106.53
55.83
52.32
180 170 160 150 140 130 120 110 100 90 80 70 60 50 40 30 20 10
f1 (ppm)

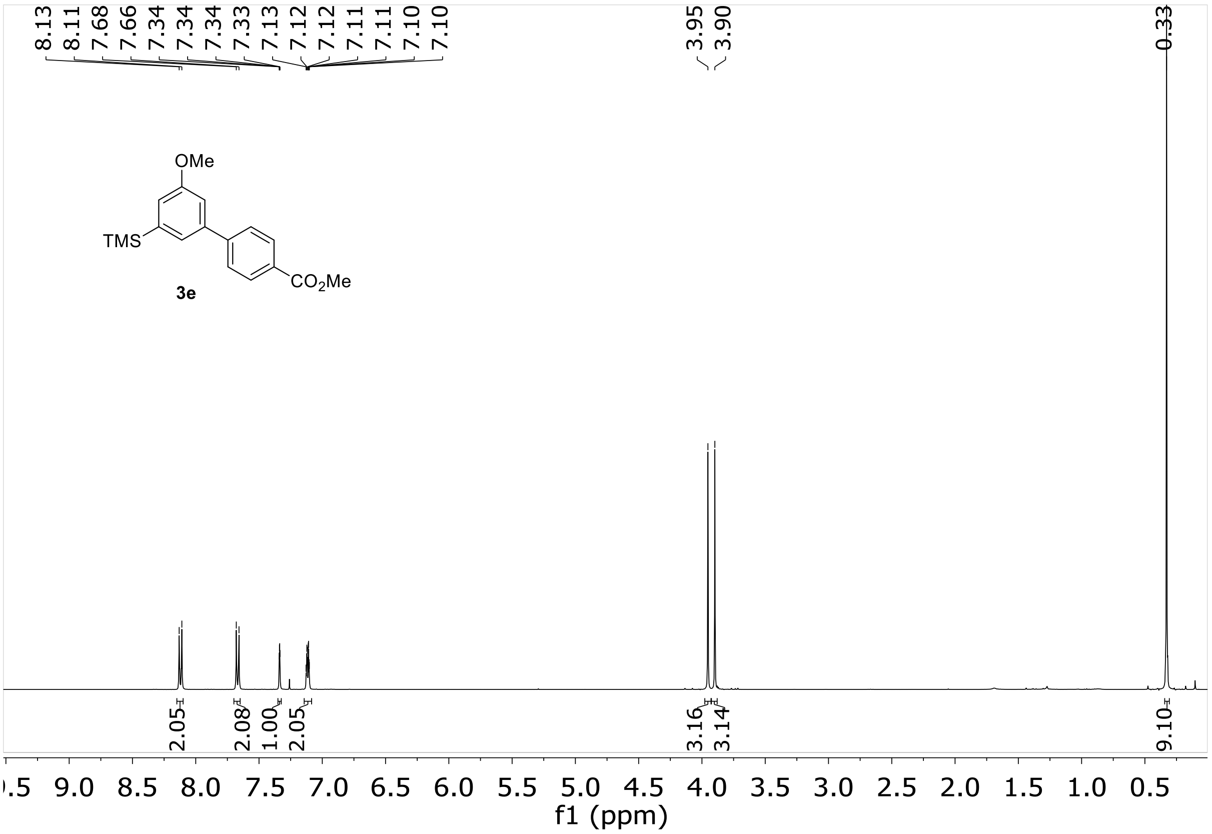
8.13 8.11 7.68 7.66 7.34 7.34 7.34 7.33 7.13 7.12 7.12 7.11 7.11 7.10 7.10
3.95 3.90
0.33
OMe
TMS
CO2Me
3e
2.05 2.08 1.00 2.05 3.16 3.14 9.10
9.5 9.0 8.5 8.0 7.5 7.0 6.5 6.0 5.5 5.0 4.5 4.0 3.5 3.0 2.5 2.0 1.5 1.0 0.5
f1 (ppm)

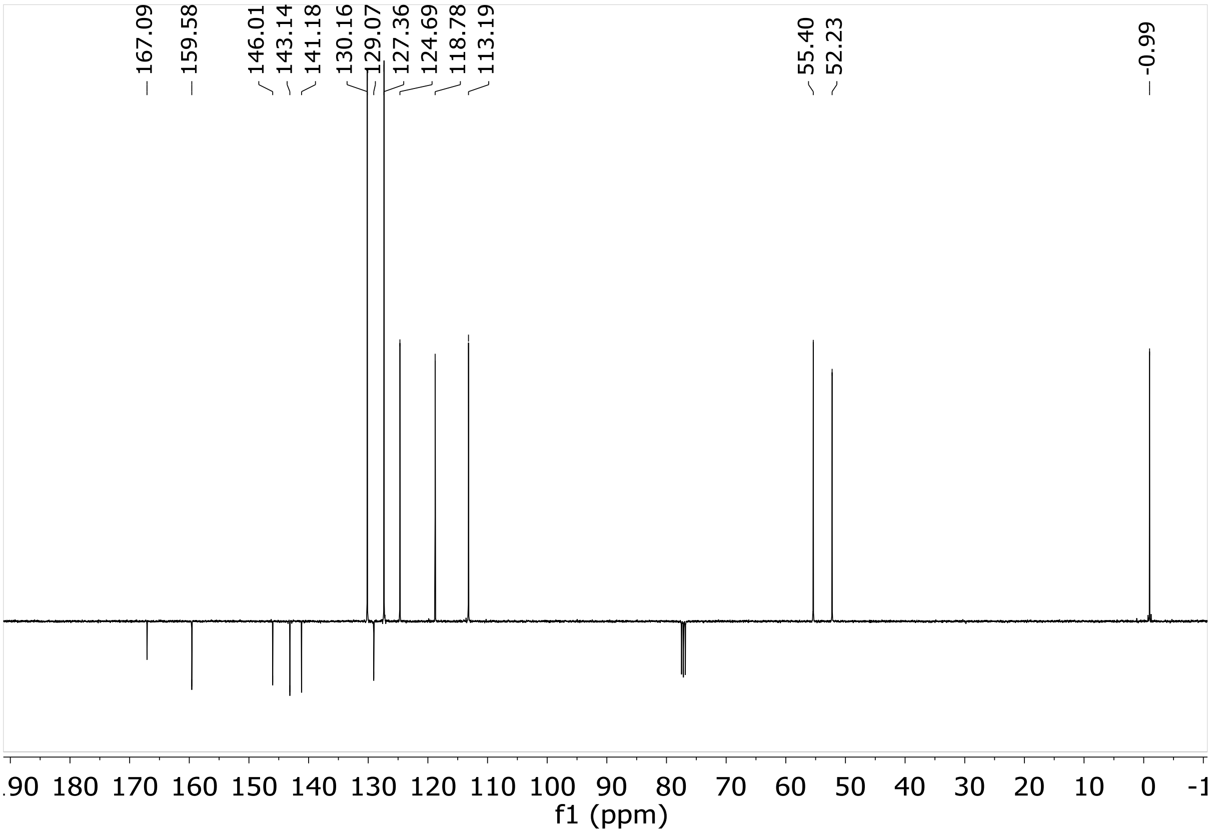
167.09 159.58 146.01 143.14 141.18 130.16 129.07 127.36 124.69 118.78 113.19
55.40 52.23
-0.99
190 180 170 160 150 140 130 120 110 100 90 80 70 60 50 40 30 20 10 0 -10
f1 (ppm)

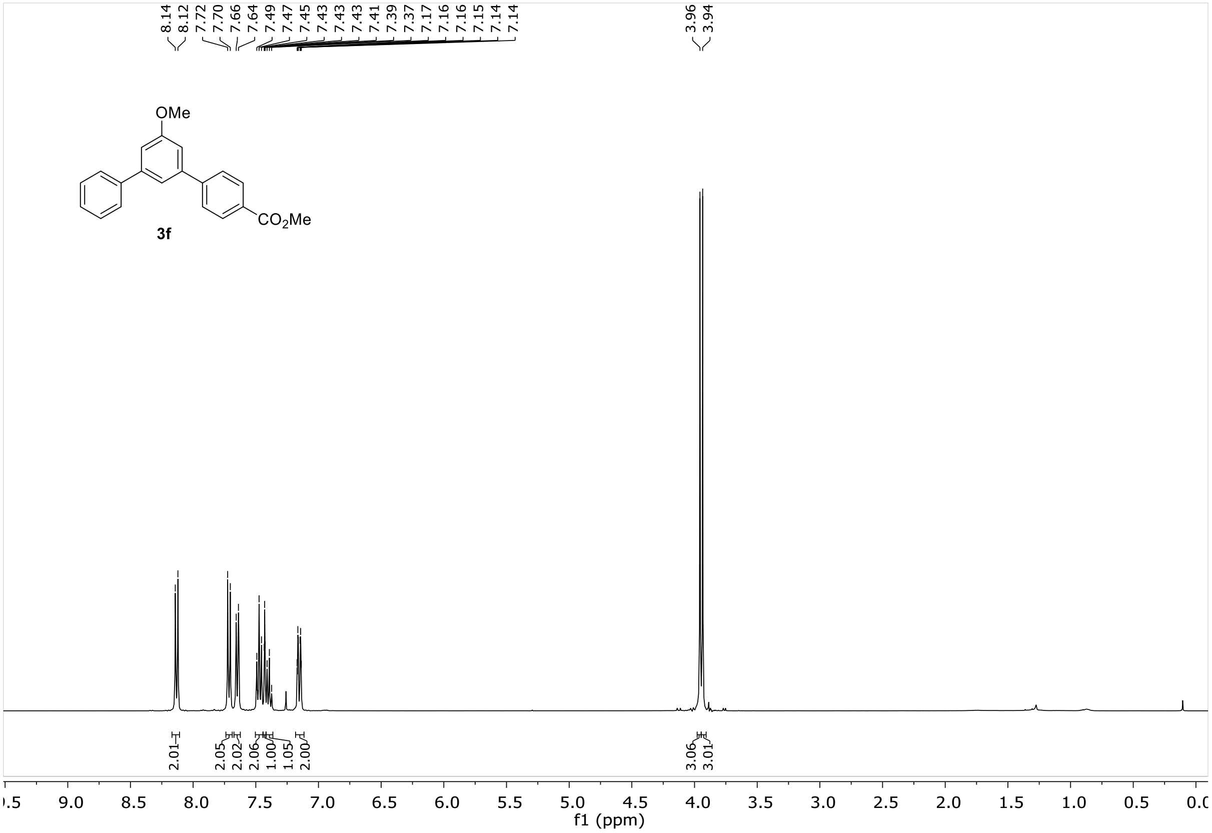
8.14
8.12
7.72
7.70
7.66
7.64
7.49
7.47
7.45
7.43
7.43
7.43
7.41
7.39
7.37
7.17
7.16
7.16
7.15
7.14
7.14
3.96
3.94
OMe
CO2Me
3f
2.01
2.05
2.02
2.06
1.00
1.05
2.00
3.06
3.01
f1 (ppm)

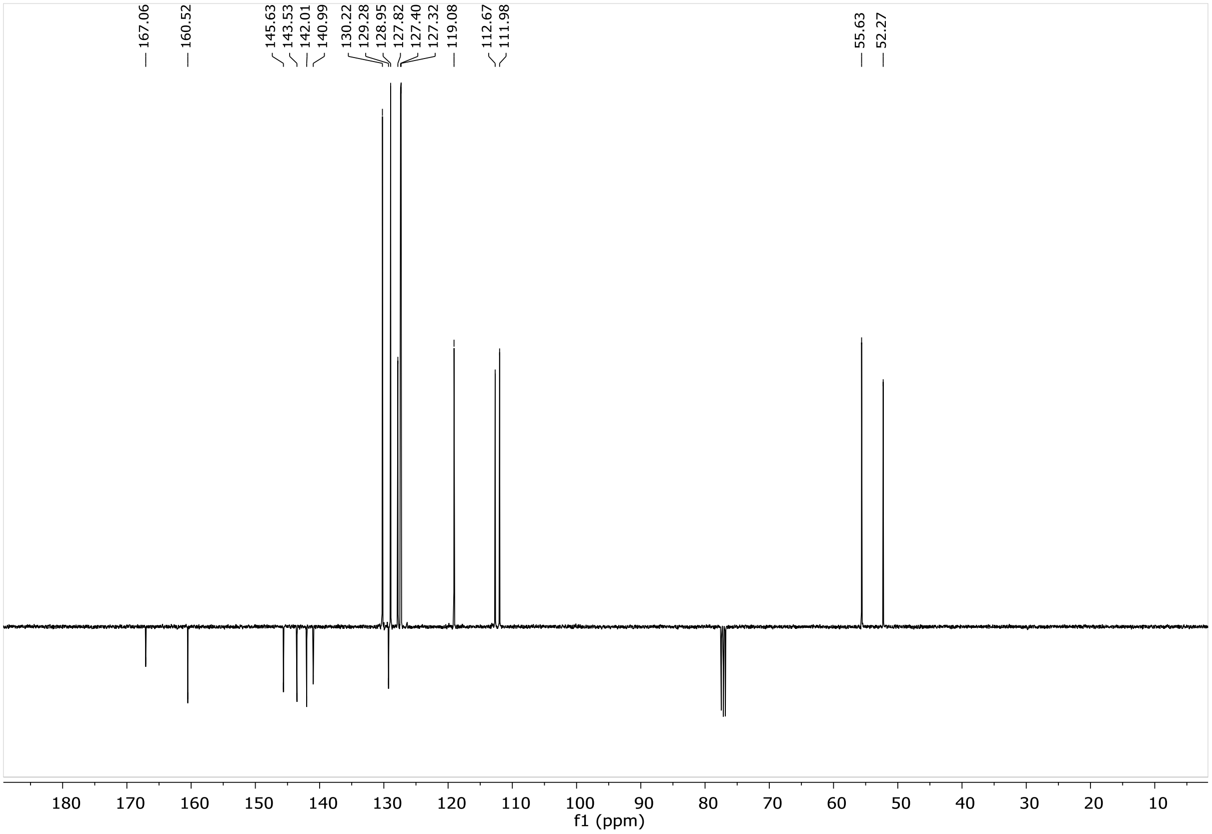
167.06
160.52
145.63
143.53
142.01
140.99
130.22
129.28
128.95
127.82
127.40
127.32
119.08
112.67
111.98
55.63
52.27
f1 (ppm)

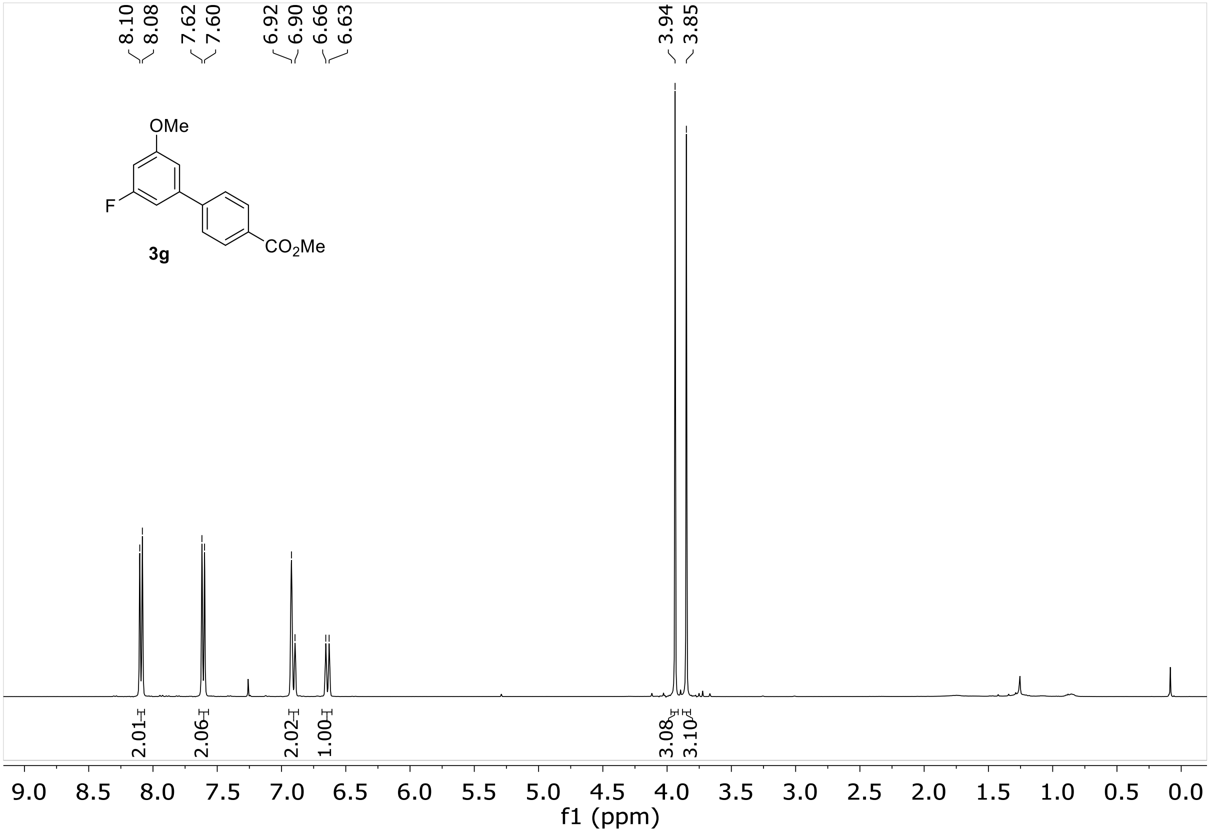
8.10
8.08
7.62
7.60
6.92
6.90
6.66
6.63
3.94
3.85
OMe
F
CO2Me
3g
2.01
2.06
2.02
1.00
3.08
3.10
9.0 8.5 8.0 7.5 7.0 6.5 6.0 5.5 5.0 4.5 4.0 3.5 3.0 2.5 2.0 1.5 1.0 0.5 0.0
f1 (ppm)

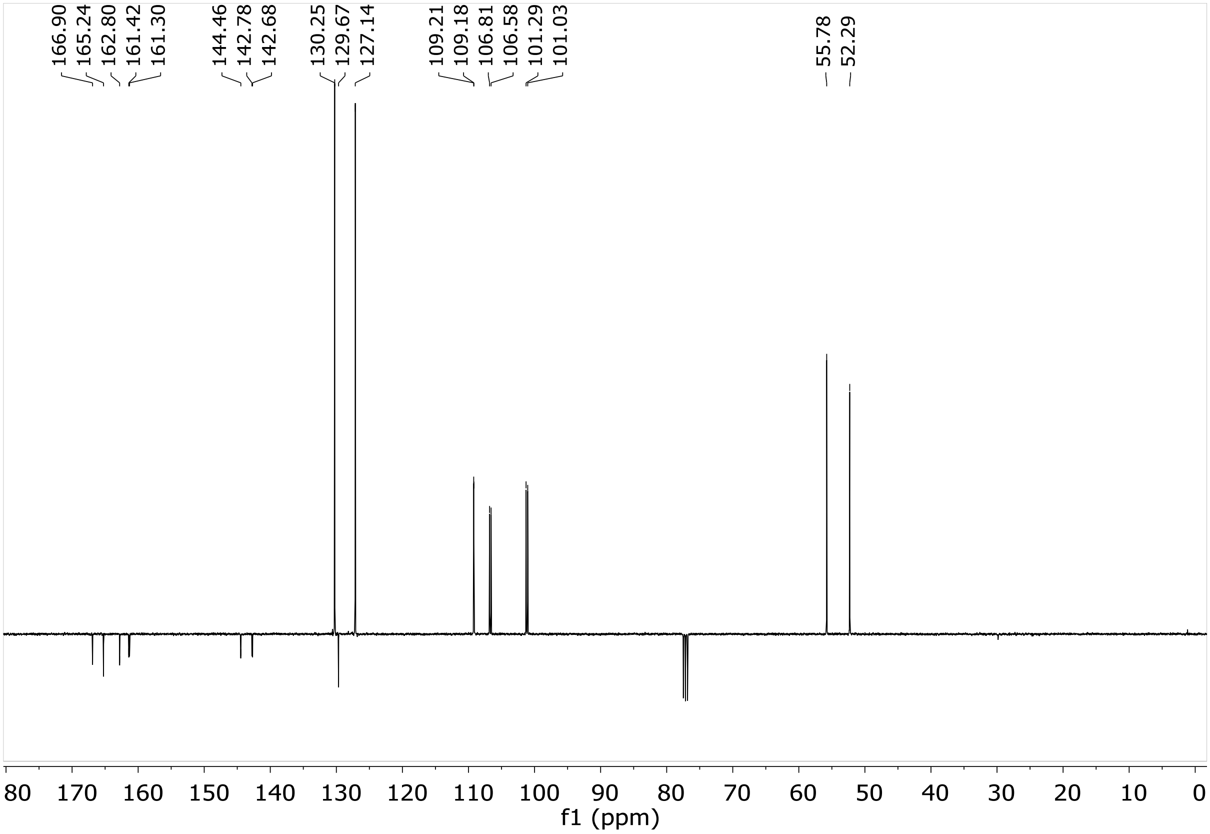
166.90
165.24
162.80
161.42
161.30
144.46
142.78
142.68
130.25
129.67
127.14
109.21
109.18
106.81
106.58
101.29
101.03
55.78
52.29
80 170 160 150 140 130 120 110 100 90 80 70 60 50 40 30 20 10 0
f1 (ppm)

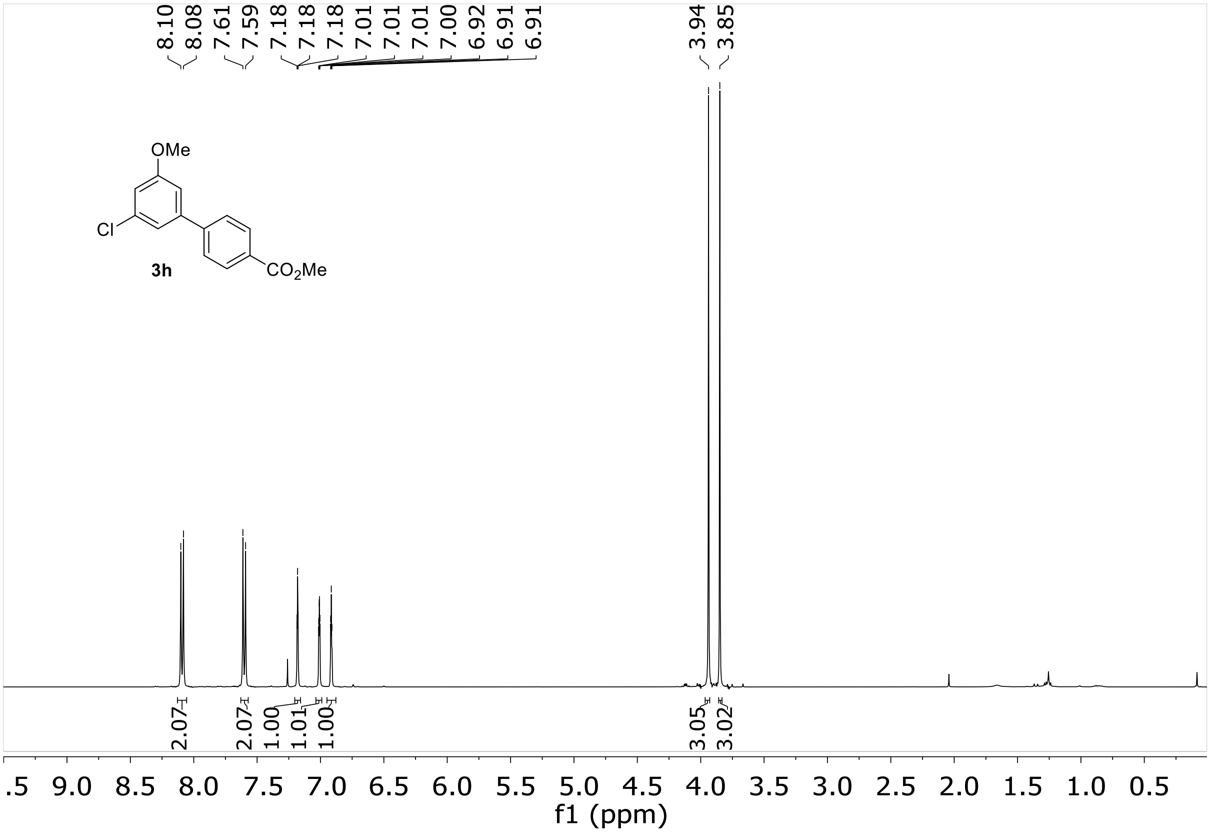
8.10
8.08
7.61
7.59
7.18
7.18
7.18
7.01
7.01
7.01
7.00
6.92
6.91
6.91
3.94
3.85
OMe
Cl
3h
CO_2Me
2.07
2.07
1.00
1.01
1.00
3.05
3.02
.5 9.0 8.5 8.0 7.5 7.0 6.5 6.0 5.5 5.0 4.5 4.0 3.5 3.0 2.5 2.0 1.5 1.0 0.5
f1 (ppm)

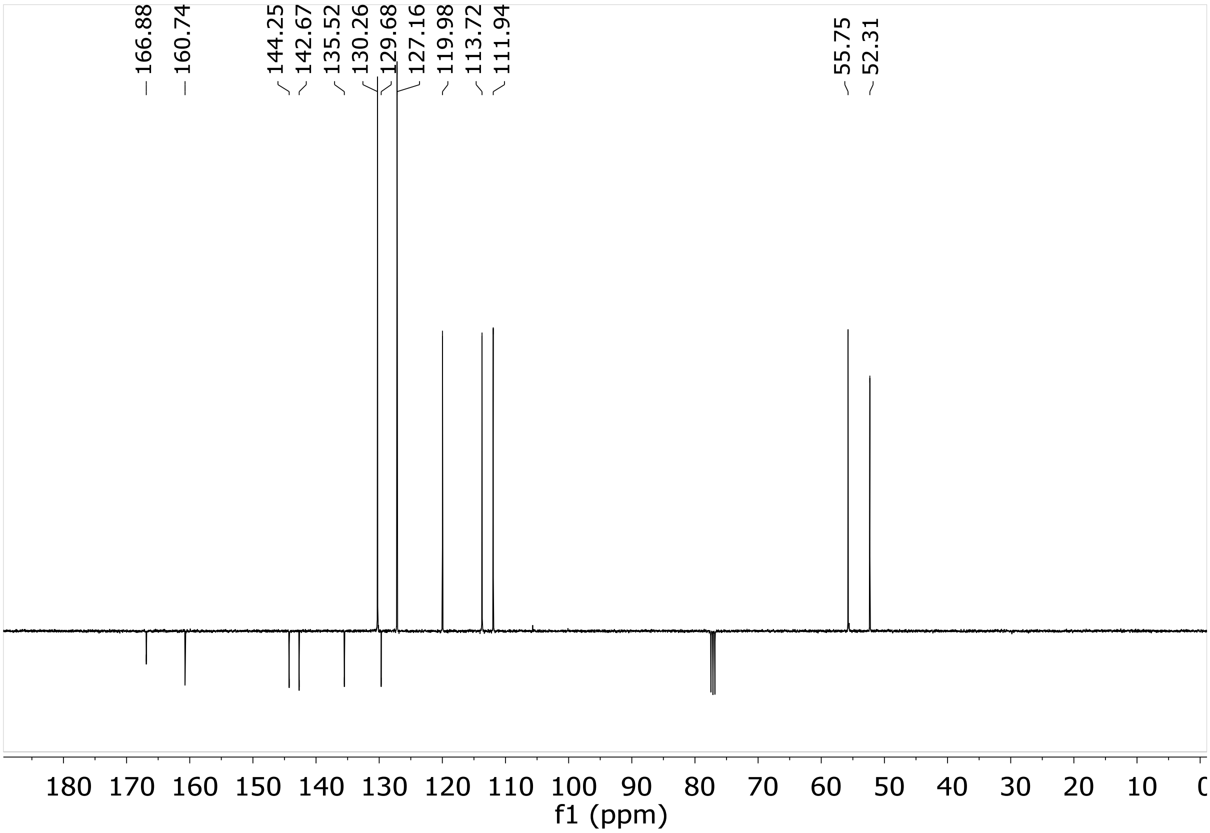
166.88
160.74
144.25
142.67
135.52
130.26
129.68
127.16
119.98
113.72
111.94
55.75
52.31
180 170 160 150 140 130 120 110 100 90 80 70 60 50 40 30 20 10 0
f1 (ppm)

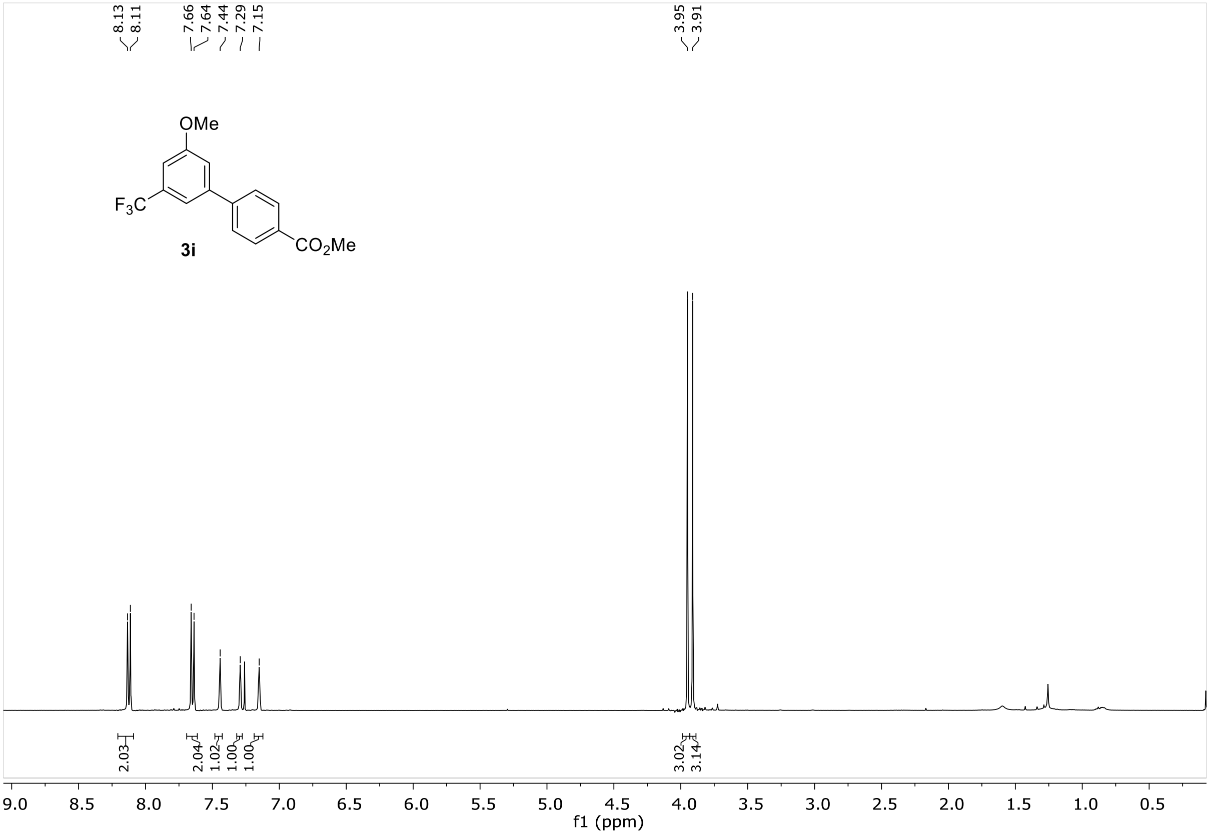
8.13
8.11
7.66
7.64
7.44
7.29
7.15
3.95
3.91
OMe
F3C
3i
CO2Me
2.03
2.04
1.02
1.00
1.00
3.02
3.14
9.0 8.5 8.0 7.5 7.0 6.5 6.0 5.5 5.0 4.5 4.0 3.5 3.0 2.5 2.0 1.5 1.0 0.5
f1 (ppm)

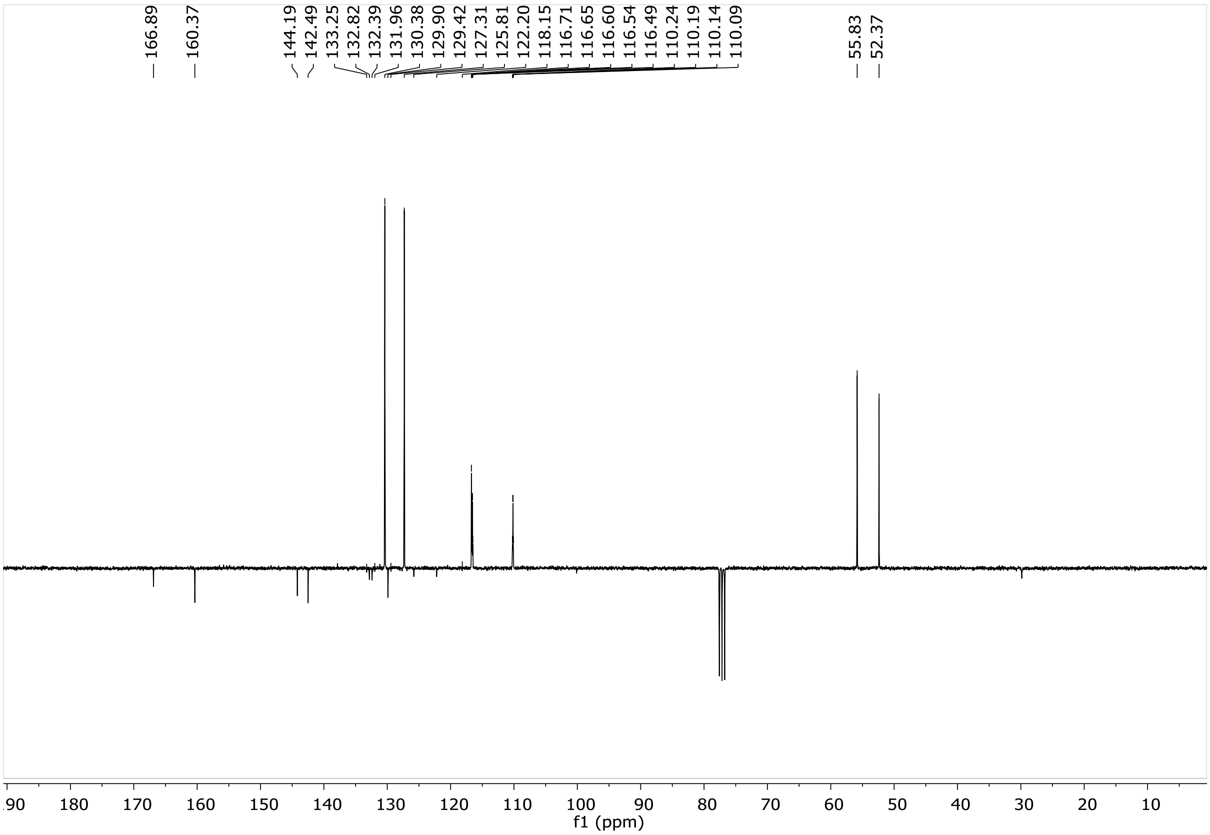
166.89
160.37
144.19
142.49
133.25
132.82
132.39
131.96
130.38
129.90
129.42
127.31
125.81
122.20
118.15
116.71
116.65
116.60
116.54
116.49
110.24
110.19
110.14
110.09
55.83
52.37
90 180 170 160 150 140 130 120 110 100 90 80 70 60 50 40 30 20 10
f1 (ppm)

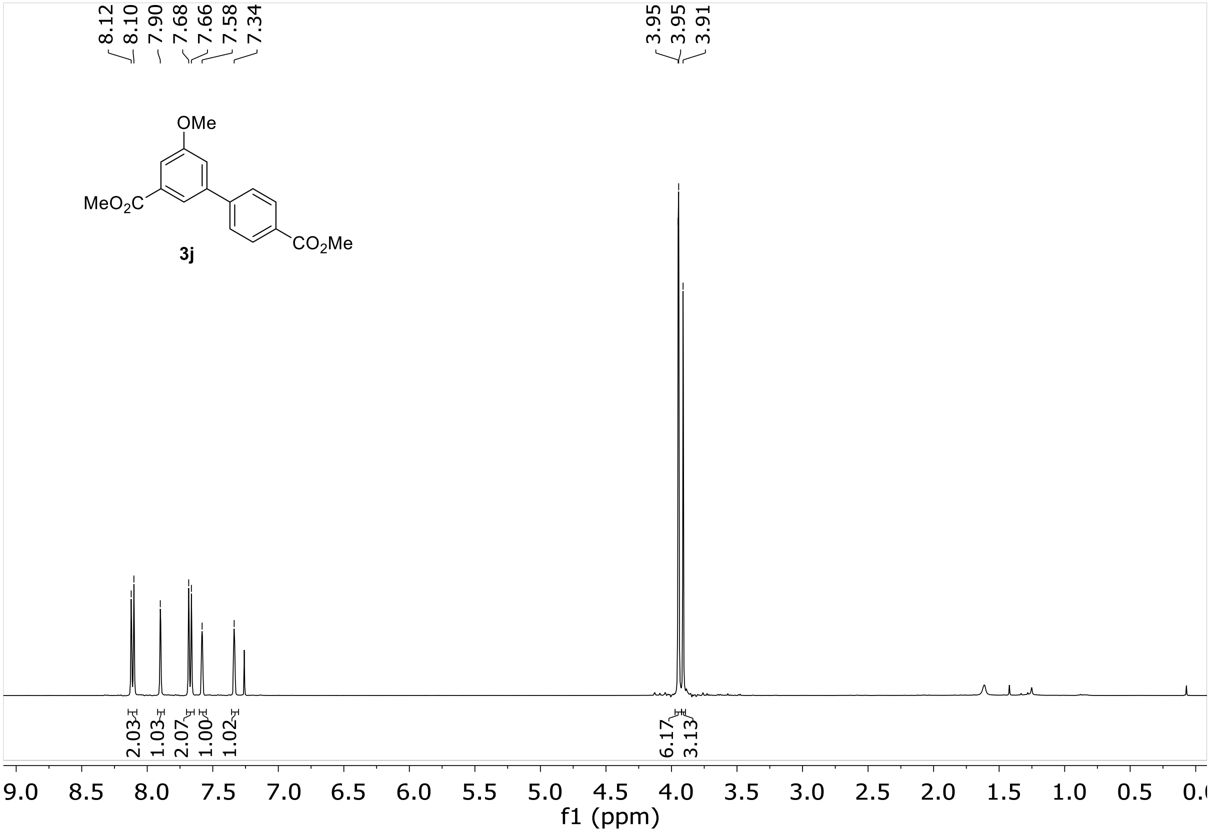
8.12
8.10
7.90
7.68
7.66
7.58
7.34
3.95
3.95
3.91
OMe
MeO2C
CO2Me
3j
2.03
1.03
2.07
1.00
1.02
6.17
3.13
9.0 8.5 8.0 7.5 7.0 6.5 6.0 5.5 5.0 4.5 4.0 3.5 3.0 2.5 2.0 1.5 1.0 0.5
f1 (ppm)

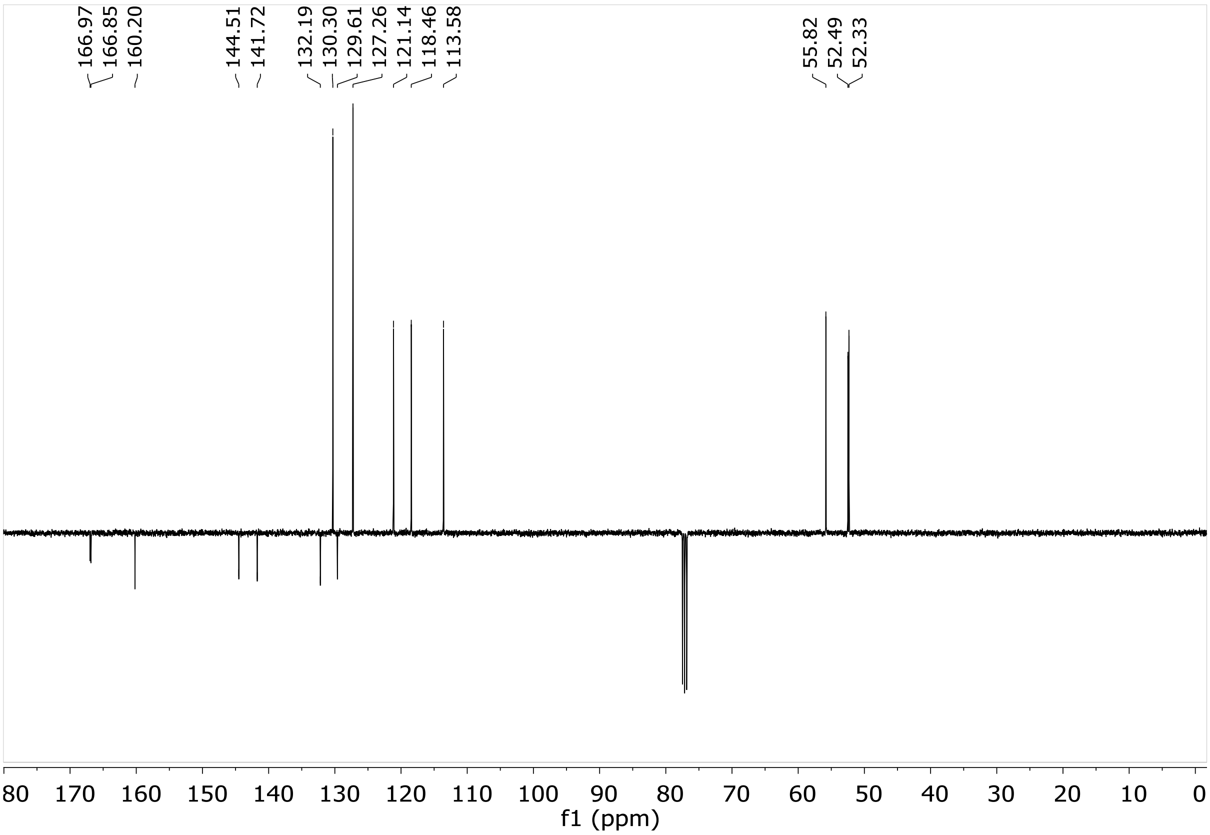
166.97
166.85
160.20
144.51
141.72
132.19
130.30
129.61
127.26
121.14
118.46
113.58
55.82
52.49
52.33
170 160 150 140 130 120 110 100 90 80 70 60 50 40 30 20 10 0
f1 (ppm)

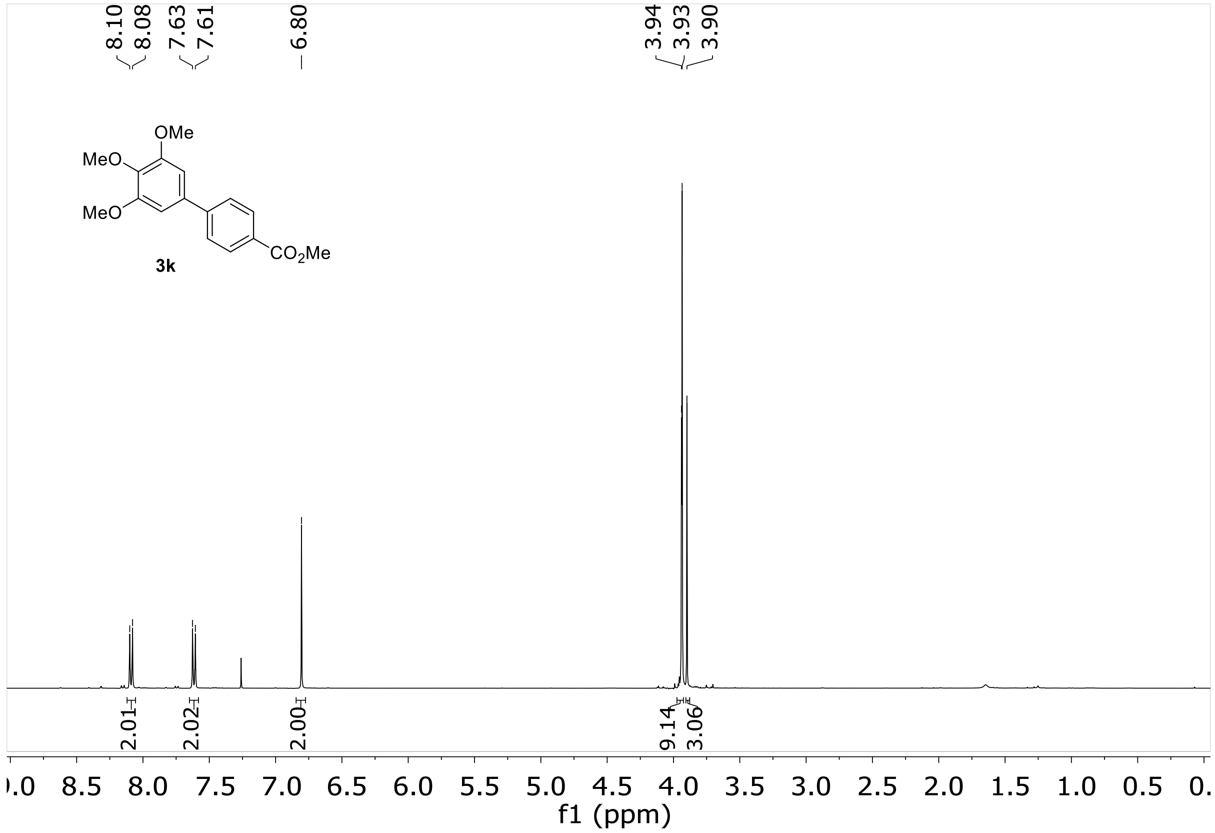
8.10
8.08
7.63
7.61
6.80
3.94
3.93
3.90
OMe
MeO
MeO
CO_2Me
3k
2.01
2.02
2.00
9.14
3.06
f1 (ppm)

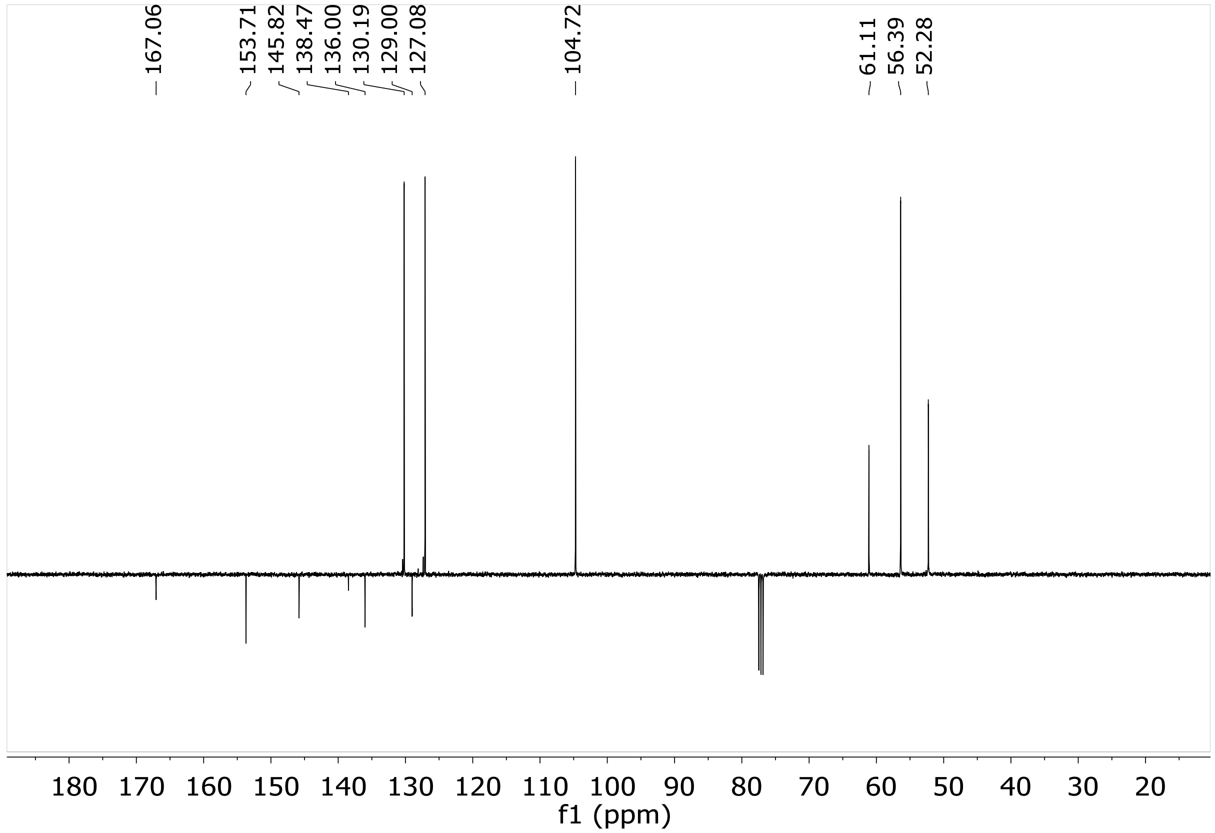
167.06
153.71
145.82
138.47
136.00
130.19
129.00
127.08
104.72
61.11
56.39
52.28
f1 (ppm)

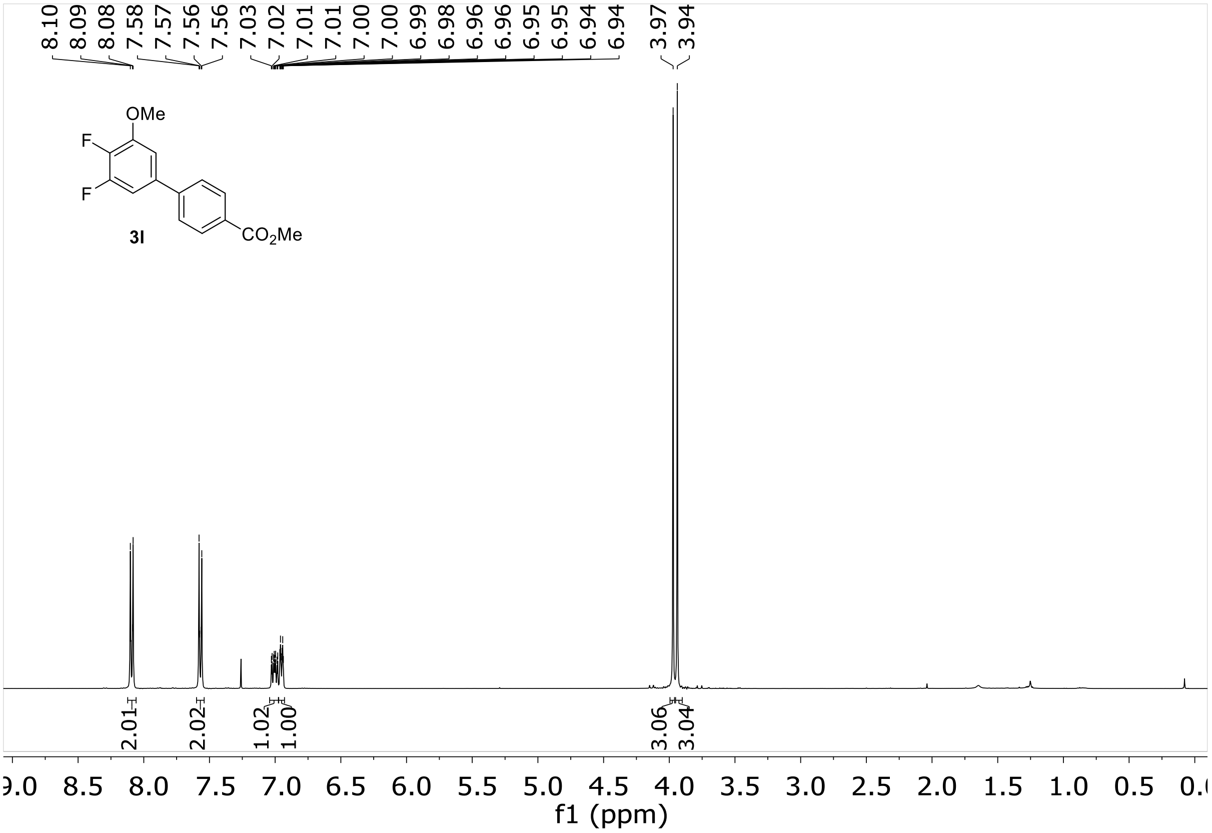
8.10
8.09
8.08
7.58
7.57
7.56
7.56
7.03
7.02
7.01
7.01
7.00
7.00
6.99
6.98
6.96
6.96
6.95
6.95
6.94
6.94
3.97
3.94
OMe
F
F
3l
CO2Me
2.01
2.02
1.02
1.00
3.06
3.04
9.0 8.5 8.0 7.5 7.0 6.5 6.0 5.5 5.0 4.5 4.0 3.5 3.0 2.5 2.0 1.5 1.0 0.5 0.0
f1 (ppm)

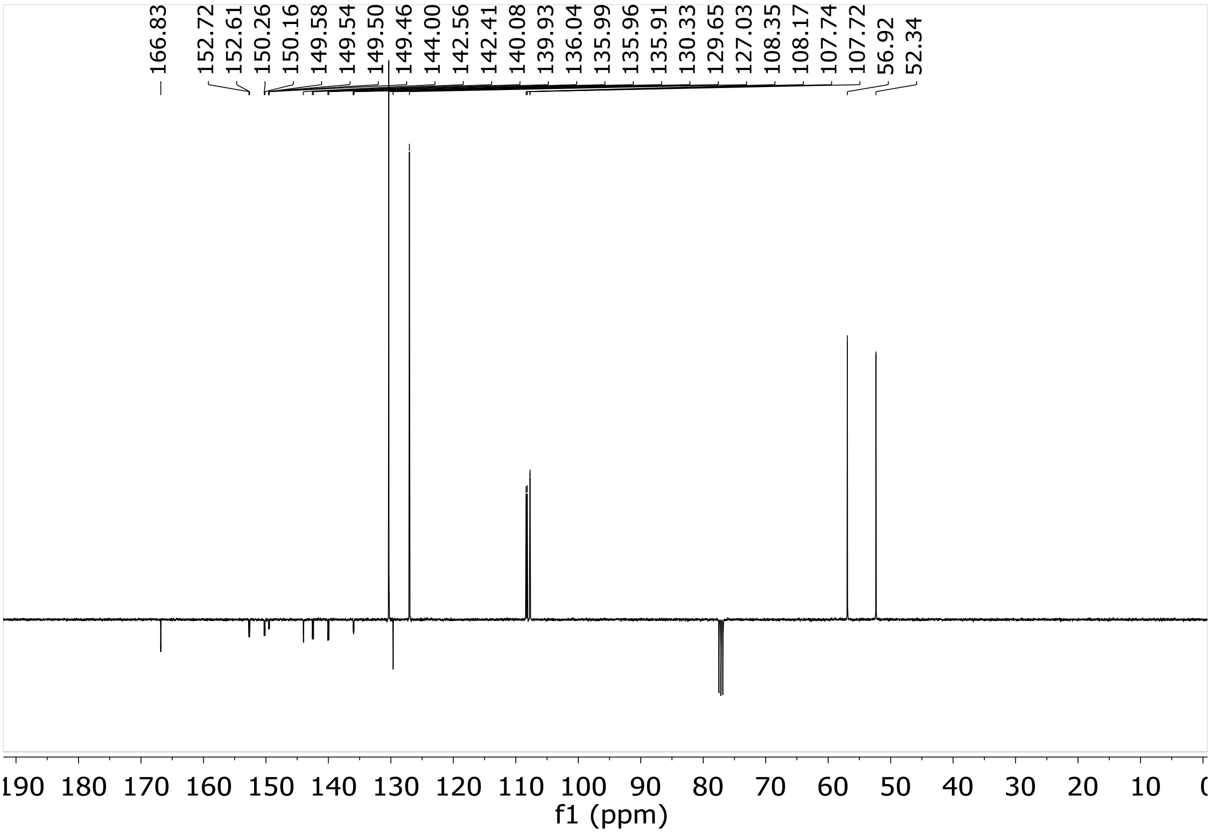
166.83
152.72
152.61
150.26
150.16
149.58
149.54
149.50
149.46
144.00
142.56
142.41
140.08
139.93
136.04
135.99
135.96
135.91
130.33
129.65
127.03
108.35
108.17
107.74
107.72
56.92
52.34
190 180 170 160 150 140 130 120 110 100 90 80 70 60 50 40 30 20 10 0
f1 (ppm)

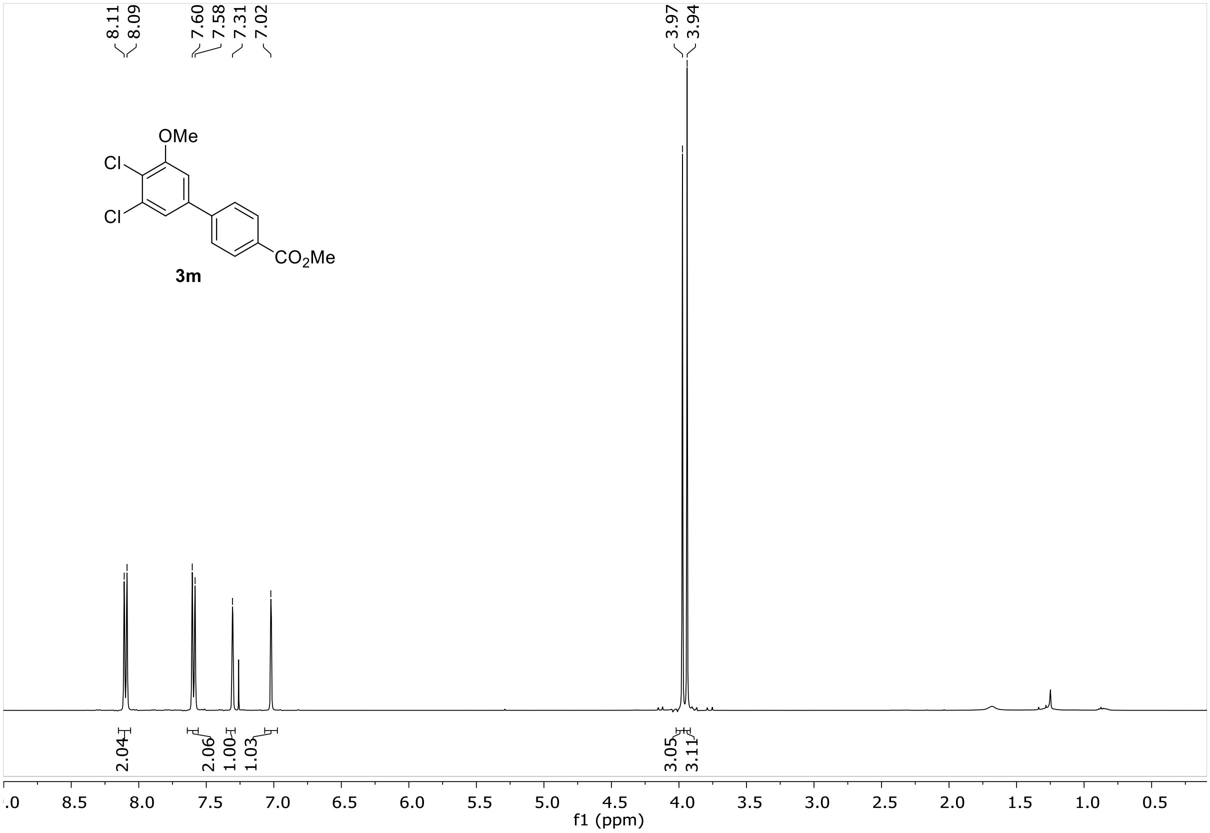
8.11
8.09
7.60
7.58
7.31
7.02
3.97
3.94
OMe
Cl
Cl
CO2Me
3m
2.04
2.06
1.00
1.03
3.05
3.11
.0 8.5 8.0 7.5 7.0 6.5 6.0 5.5 5.0 4.5 4.0 3.5 3.0 2.5 2.0 1.5 1.0 0.5
f1 (ppm)

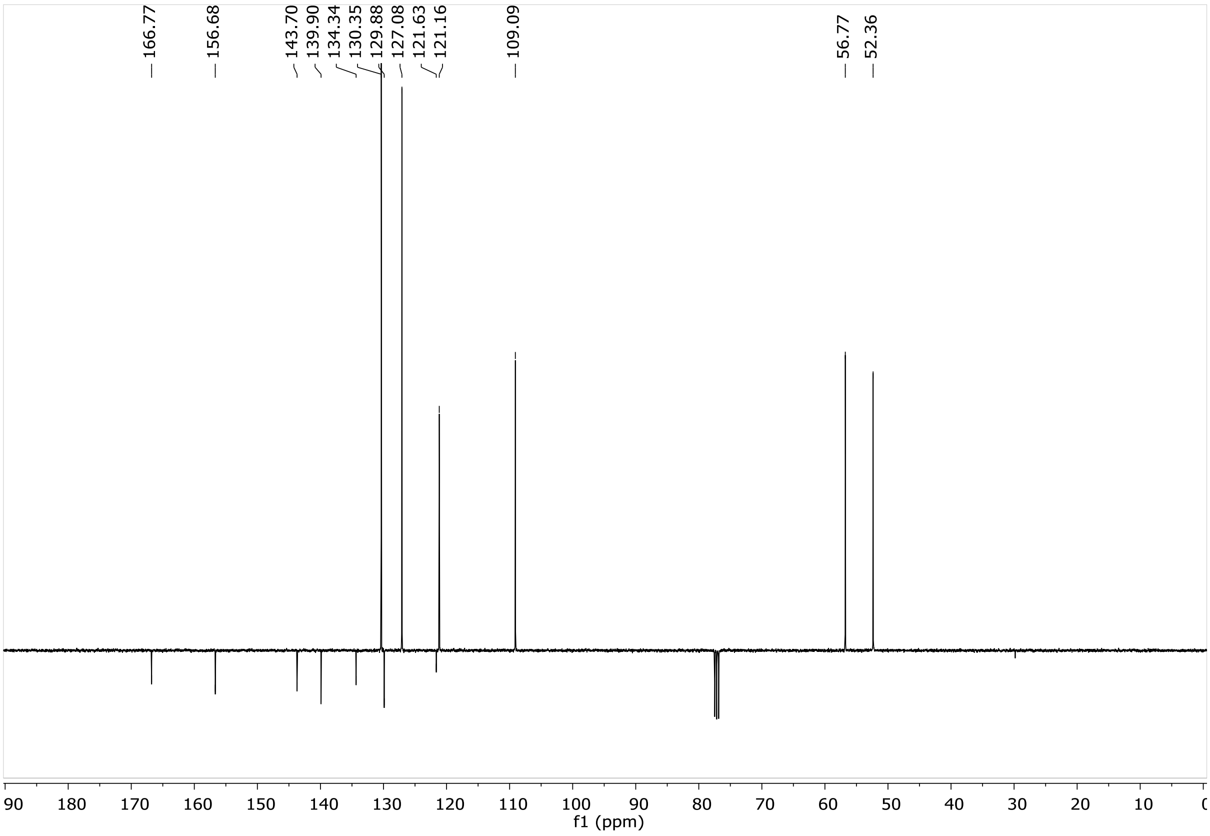
166.77
156.68
143.70
139.90
134.34
130.35
129.88
127.08
121.63
121.16
109.09
56.77
52.36
90 180 170 160 150 140 130 120 110 100 90 80 70 60 50 40 30 20 10
f1 (ppm)

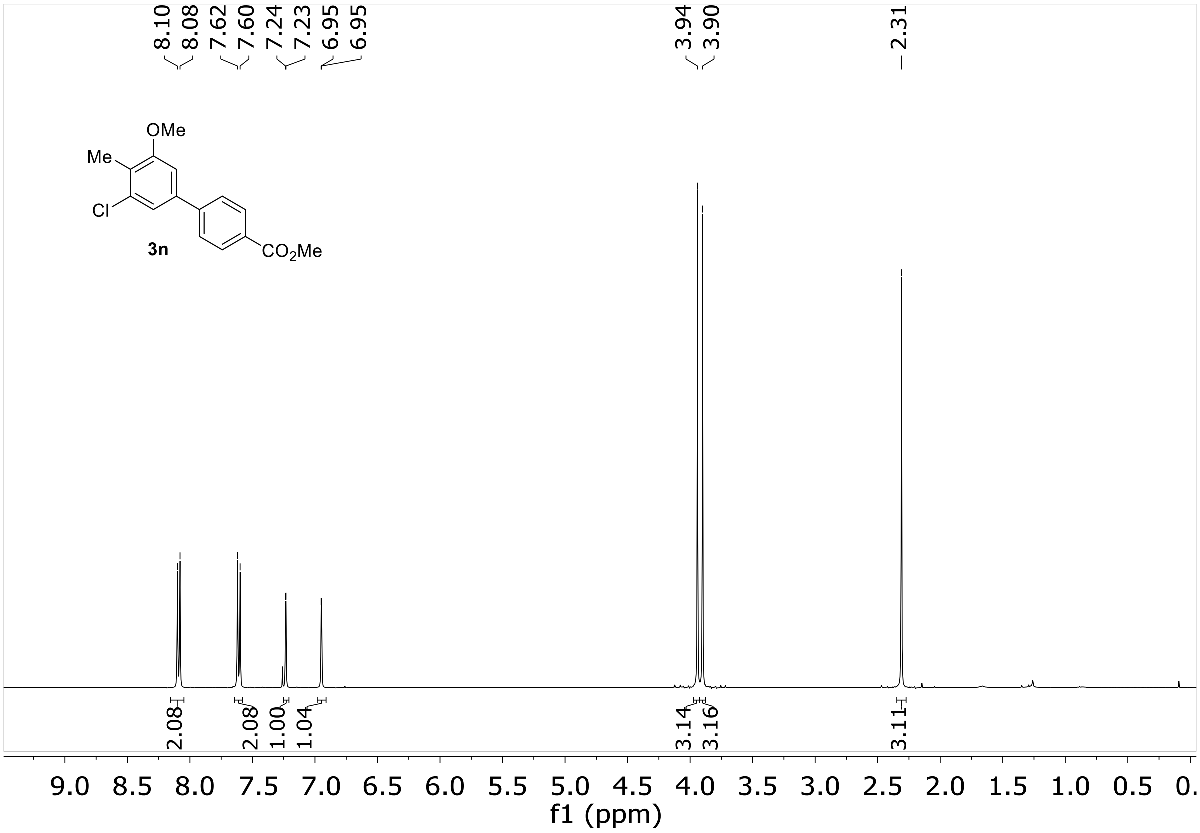
8.10
8.08
7.62
7.60
7.24
7.23
6.95
6.95
3.94
3.90
2.31
OMe
Me
Cl
3n
CO_2Me
2.08
2.08
1.00
1.04
3.14
3.16
3.11
9.0 8.5 8.0 7.5 7.0 6.5 6.0 5.5 5.0 4.5 4.0 3.5 3.0 2.5 2.0 1.5 1.0 0.5 0.
f1 (ppm)

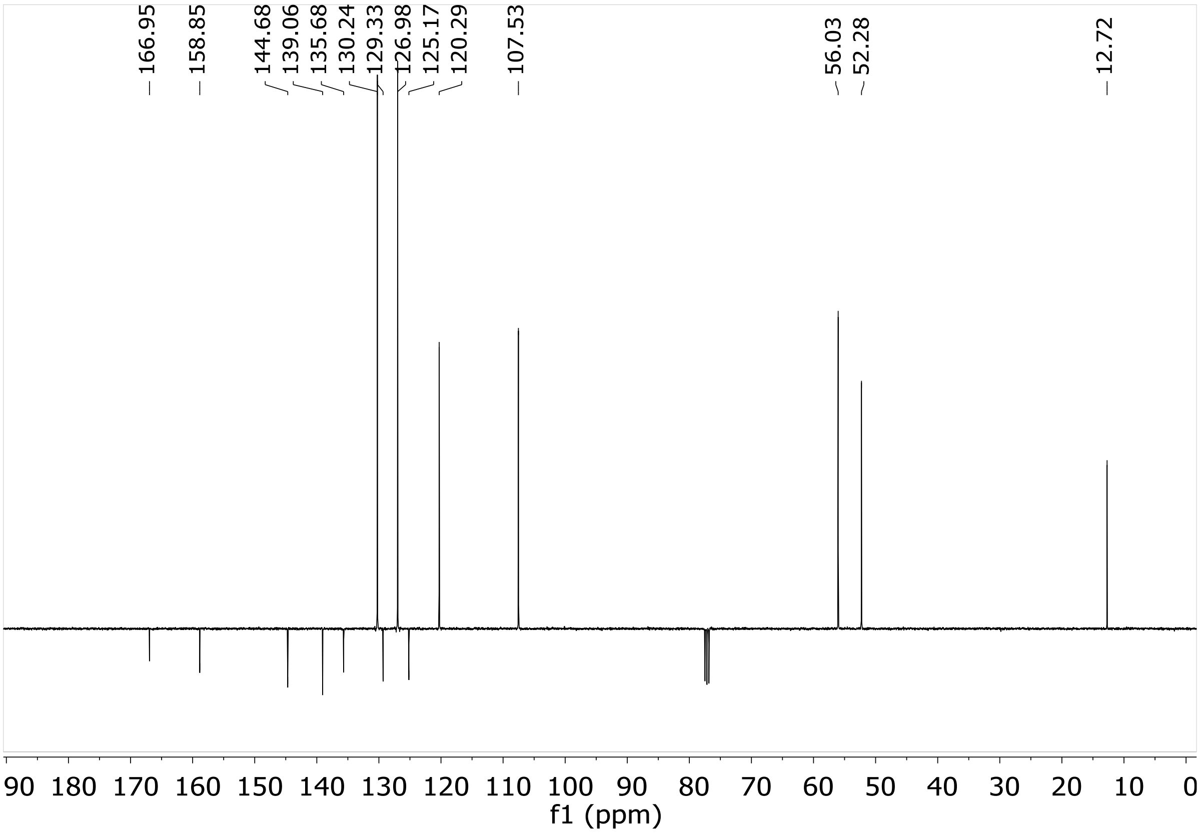
166.95
158.85
144.68
139.06
135.68
130.24
129.33
126.98
125.17
120.29
107.53
56.03
52.28
12.72
90 180 170 160 150 140 130 120 110 100 90 80 70 60 50 40 30 20 10 0
f1 (ppm)

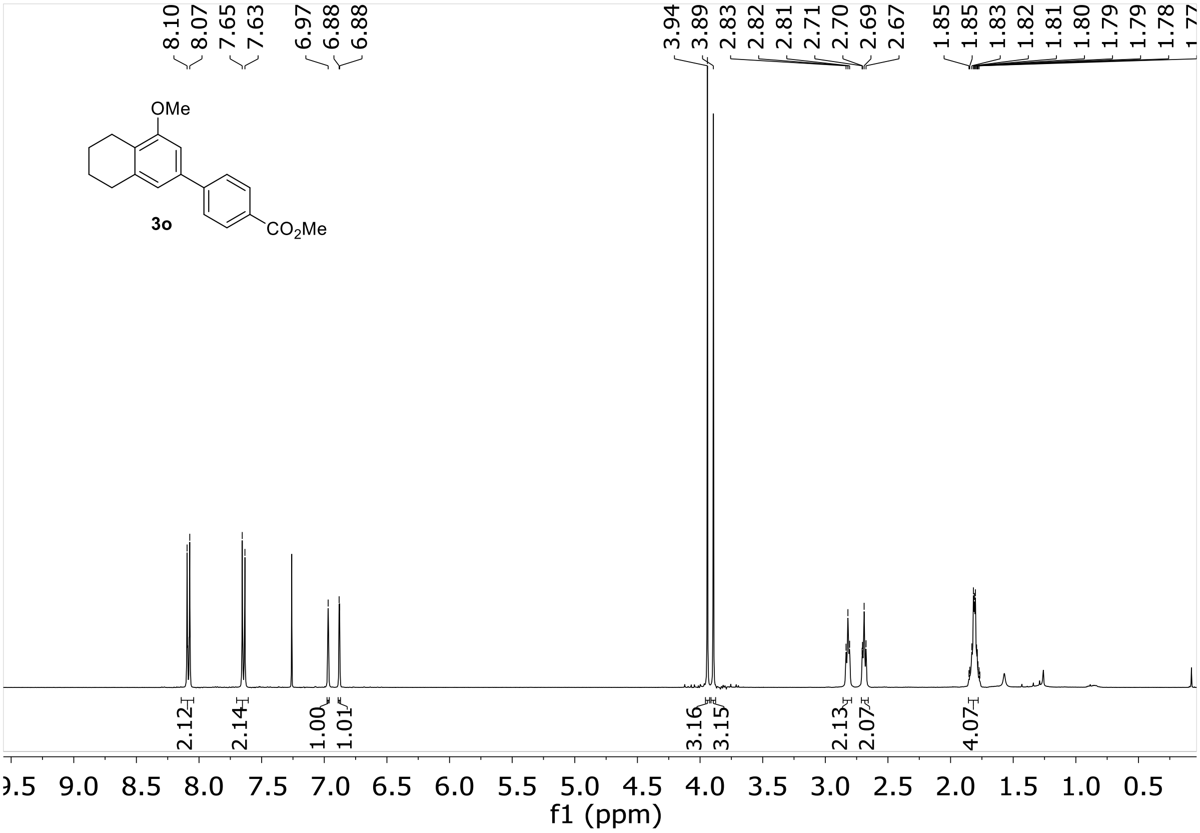
8.10
8.07
7.65
7.63
6.97
6.88
6.88
3.94
3.89
2.83
2.82
2.81
2.71
2.70
2.69
2.67
1.85
1.85
1.83
1.82
1.81
1.80
1.79
1.79
1.78
OMe
3o
CO_2Me
2.12
2.14
1.00
1.01
3.16
3.15
2.13
2.07
4.07
9.5 9.0 8.5 8.0 7.5 7.0 6.5 6.0 5.5 5.0 4.5 4.0 3.5 3.0 2.5 2.0 1.5 1.0 0.5
f1 (ppm)

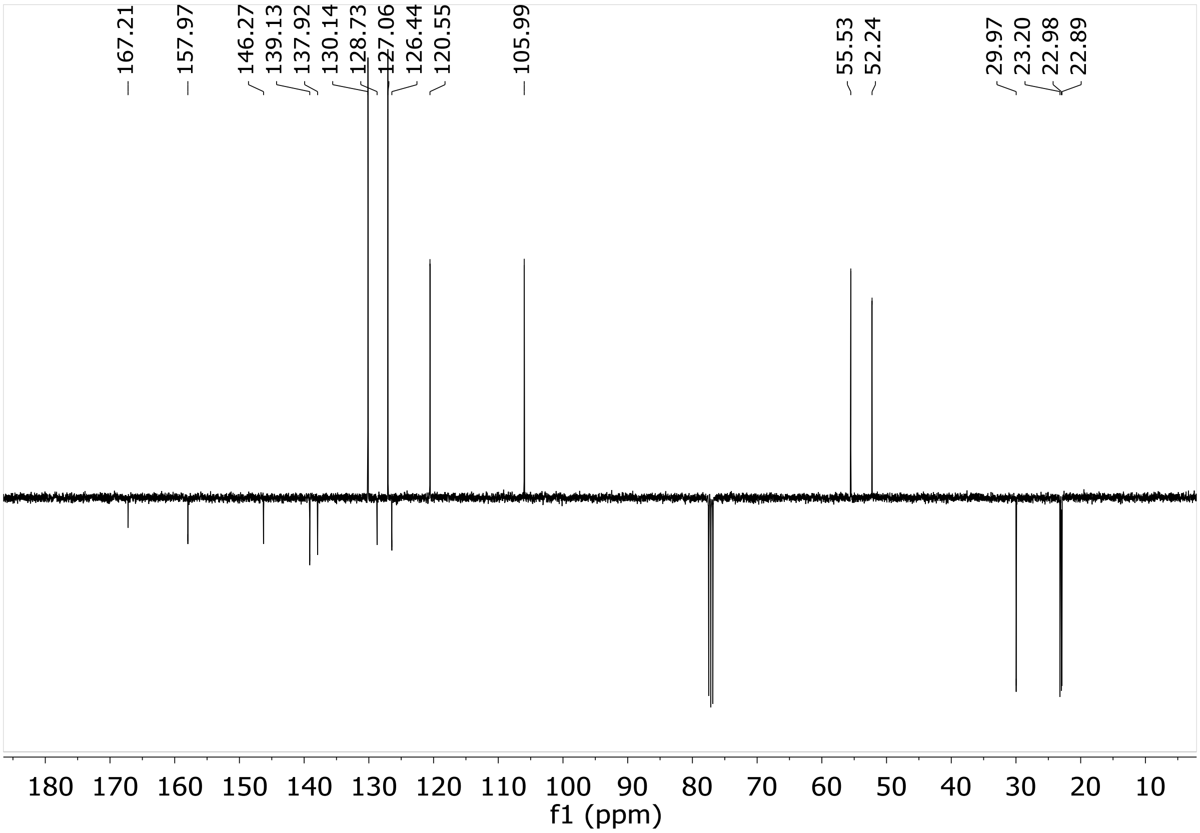
167.21
157.97
146.27
139.13
137.92
130.14
128.73
127.06
126.44
120.55
105.99
55.53
52.24
29.97
23.20
22.98
22.89
180 170 160 150 140 130 120 110 100 90 80 70 60 50 40 30 20 10
f1 (ppm)

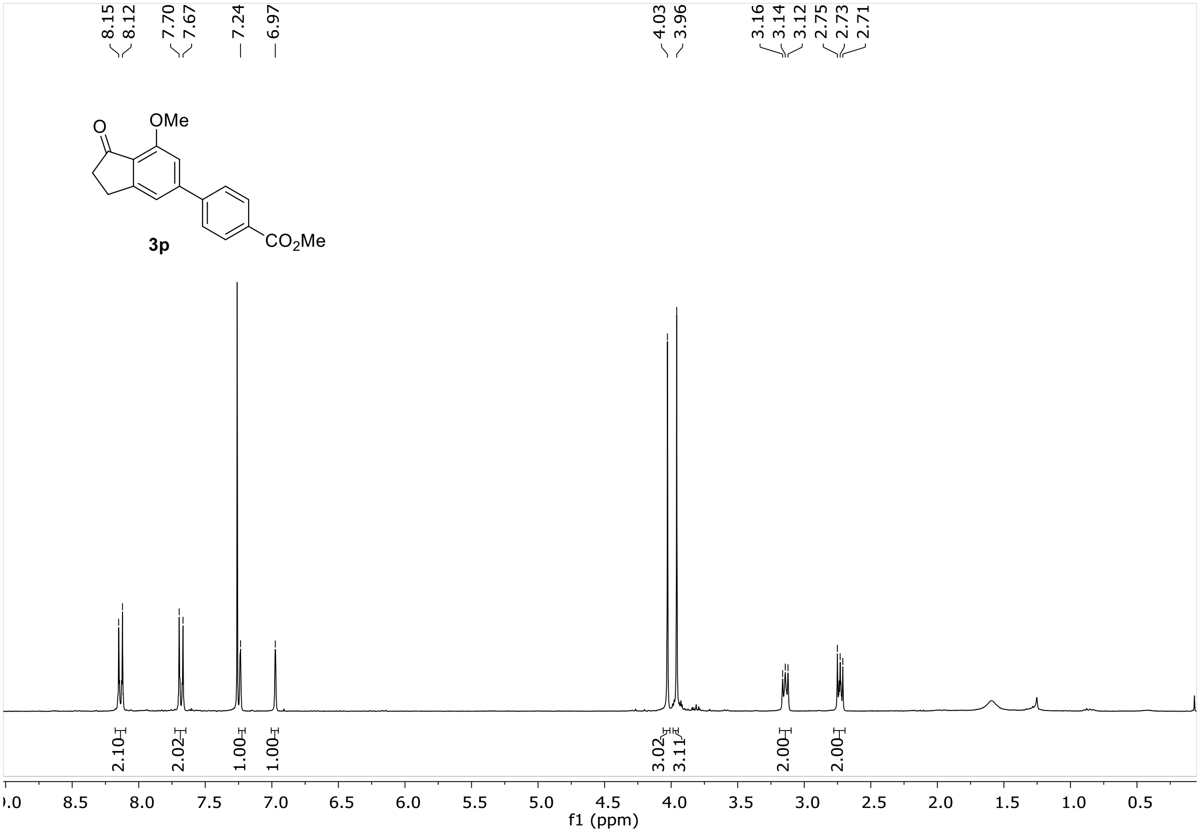
8.15
8.12
7.70
7.67
7.24
6.97
4.03
3.96
3.16
3.14
3.12
2.75
2.73
2.71
O
OMe
3p
CO2Me
2.10
2.02
1.00
1.00
3.02
3.11
2.00
2.00
f1 (ppm)

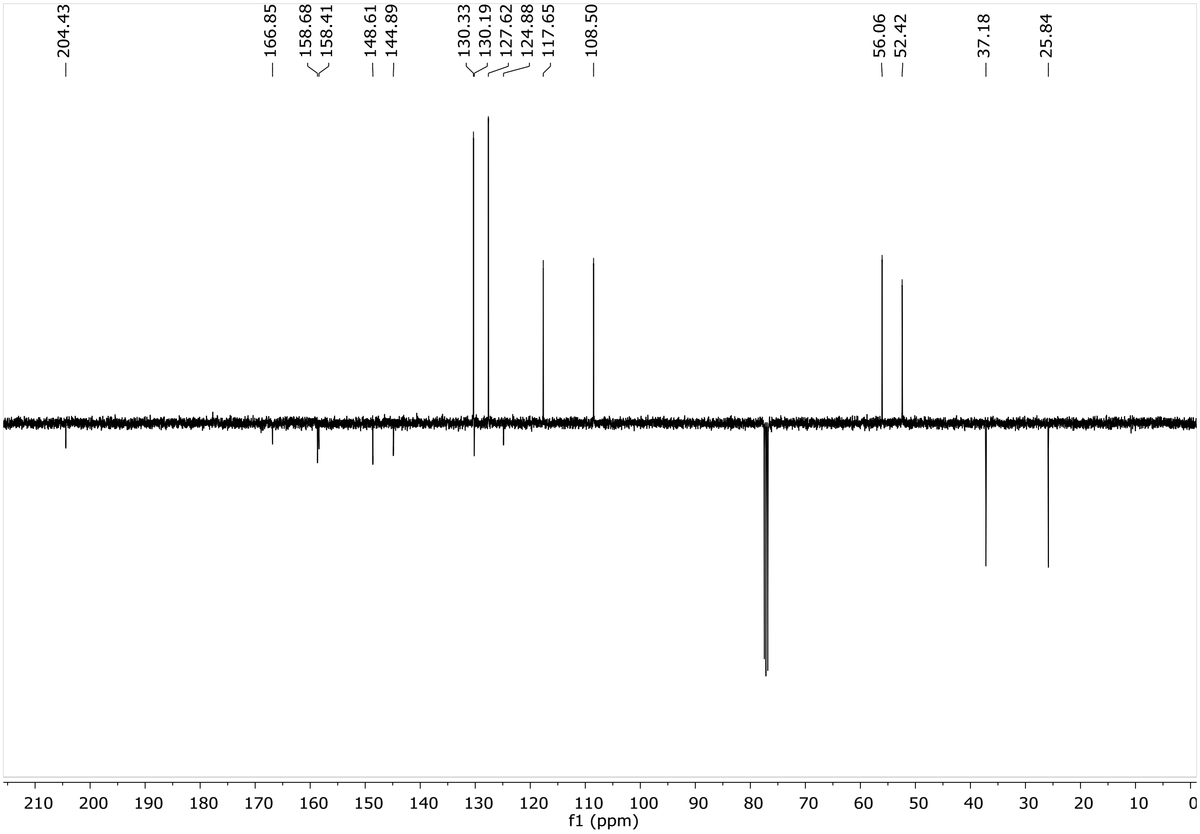
204.43
166.85
158.68
158.41
148.61
144.89
130.33
130.19
127.62
124.88
117.65
108.50
56.06
52.42
37.18
25.84
f1 (ppm)

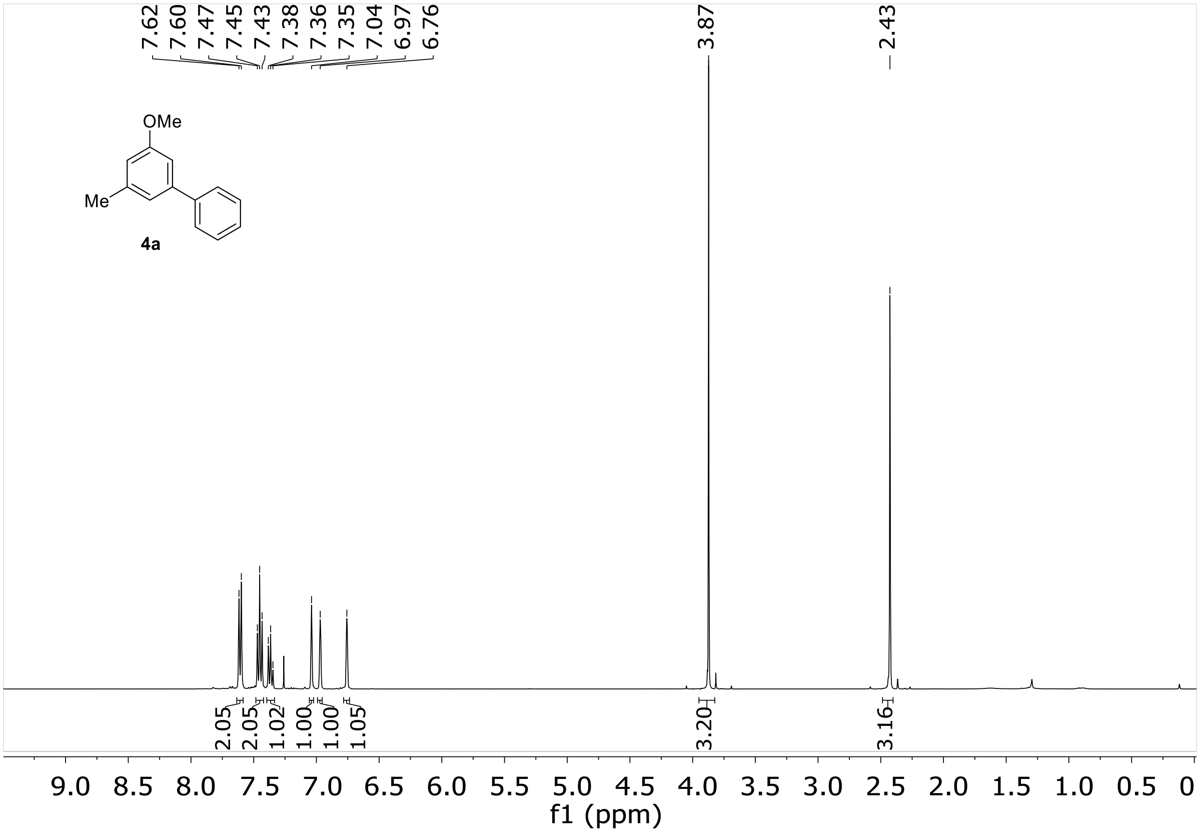
7.62
7.60
7.47
7.45
7.43
7.38
7.36
7.35
7.04
6.97
6.76
3.87
2.43
OMe
Me
4a
2.05
2.05
1.02
1.00
1.00
1.05
3.20
3.16
9.0 8.5 8.0 7.5 7.0 6.5 6.0 5.5 5.0 4.5 4.0 3.5 3.0 2.5 2.0 1.5 1.0 0.5 0
f1 (ppm)

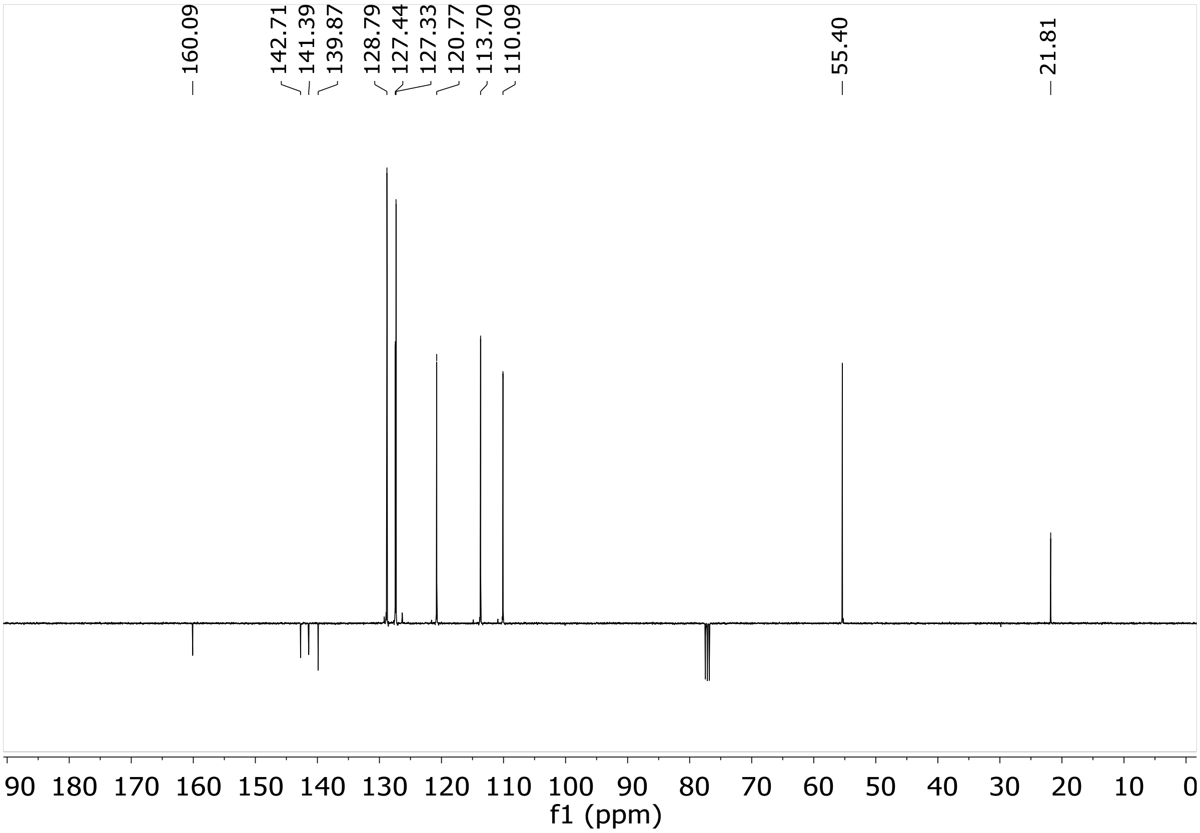
160.09
142.71
141.39
139.87
128.79
127.44
127.33
120.77
113.70
110.09
55.40
21.81
90 180 170 160 150 140 130 120 110 100 90 80 70 60 50 40 30 20 10 0
f1 (ppm)

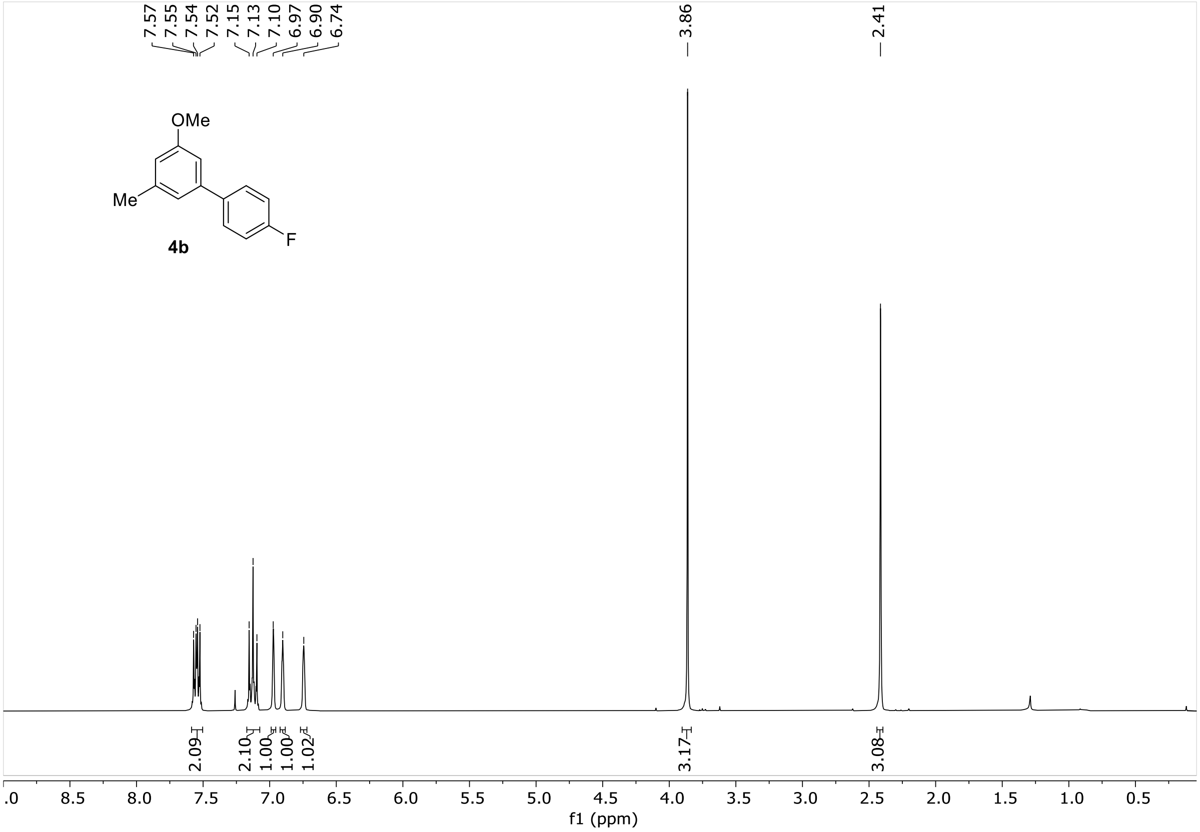
7.57
7.55
7.54
7.52
7.15
7.13
7.10
6.97
6.90
6.74
3.86
2.41
OMe
Me
F
4b
2.09
2.10
1.00
1.00
1.02
3.17
3.08
9.0
8.5
8.0
7.5
7.0
6.5
6.0
5.5
5.0
4.5
4.0
3.5
3.0
2.5
2.0
1.5
1.0
0.5
f1 (ppm)

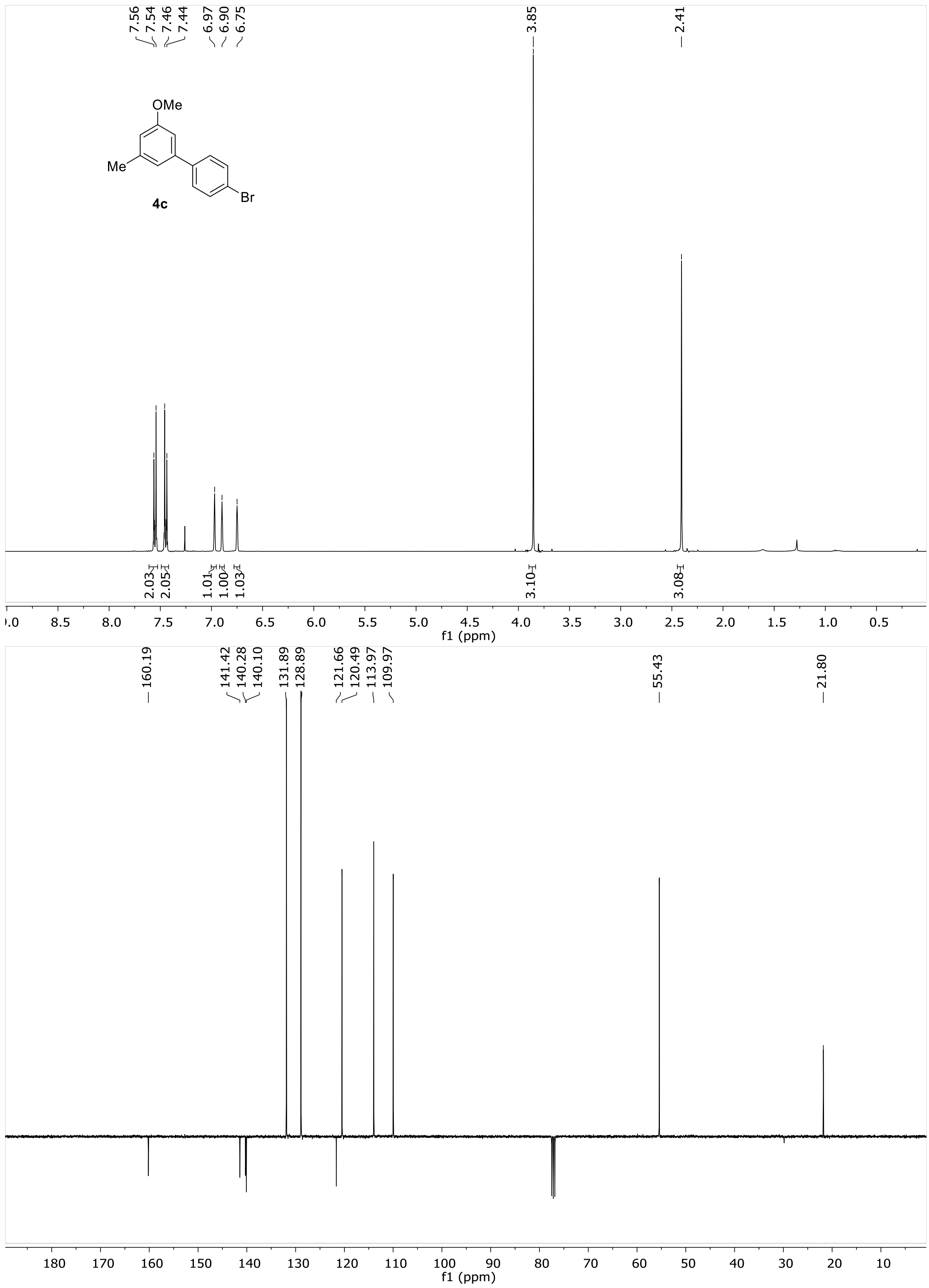
7.56
7.54
7.46
7.44
6.97
6.90
6.75
3.85
2.41
OMe
Me
Br
4c
2.03
2.05
1.01
1.00
1.03
3.10
3.08
f1 (ppm)
160.19
141.42
140.28
140.10
131.89
128.89
121.66
120.49
113.97
109.97
55.43
21.80
f1 (ppm)

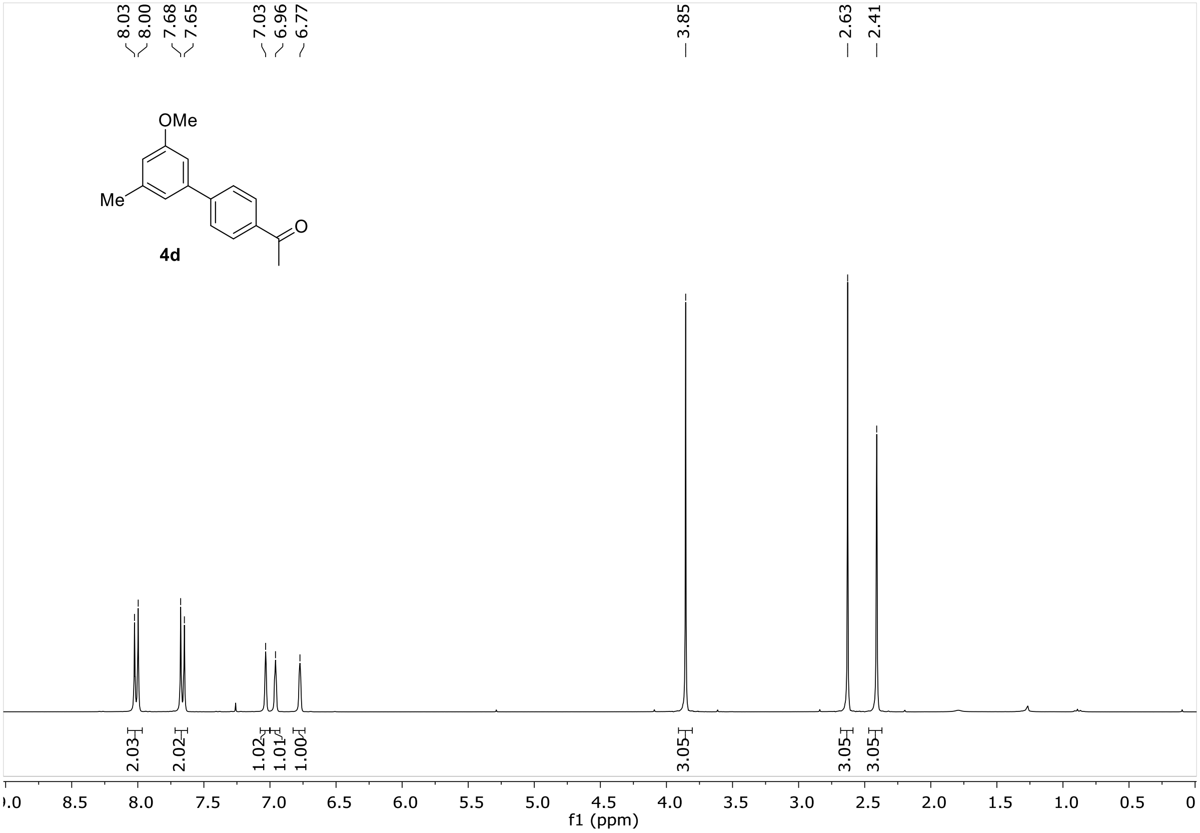
8.03
8.00
7.68
7.65
7.03
6.96
6.77
3.85
2.63
2.41
OMe
Me
O
4d
2.03
2.02
1.02
1.01
1.00
3.05
3.05
3.05
f1 (ppm)

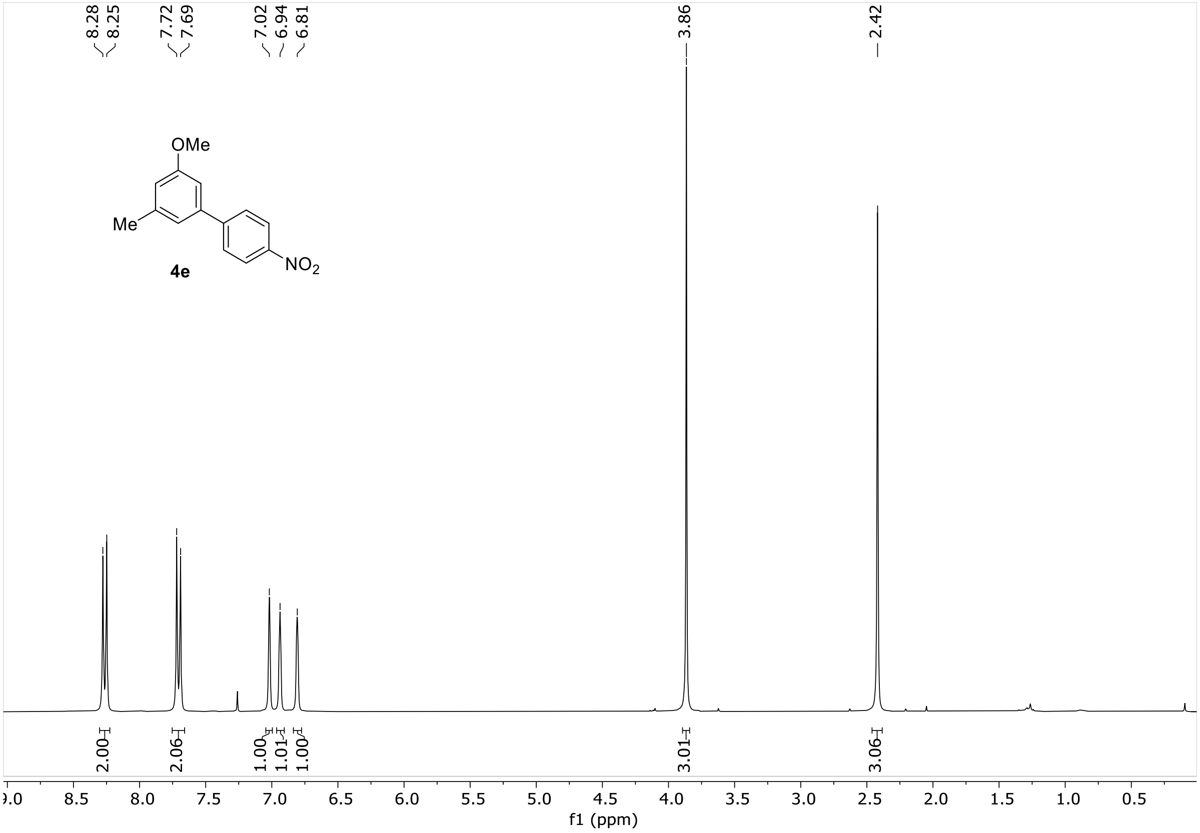
8.28
8.25
7.72
7.69
7.02
6.94
6.81
3.86
2.42
OMe
Me
NO_2
4e
2.00
2.06
1.00
1.01
1.00
3.01
3.06
f1 (ppm)

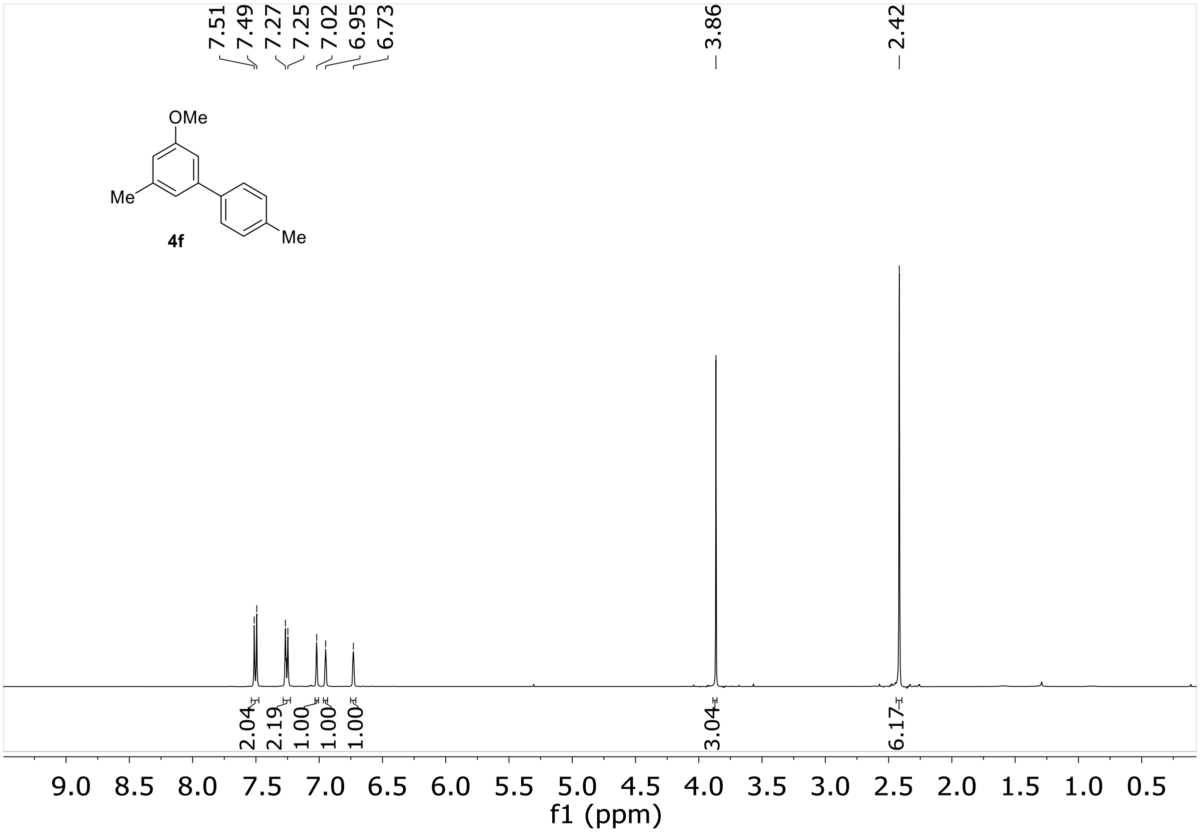
7.51
7.49
7.27
7.25
7.02
6.95
6.73
3.86
2.42
OMe
Me
Me
4f
2.04
2.19
1.00
1.00
1.00
3.04
6.17
9.0 8.5 8.0 7.5 7.0 6.5 6.0 5.5 5.0 4.5 4.0 3.5 3.0 2.5 2.0 1.5 1.0 0.5
f1 (ppm)

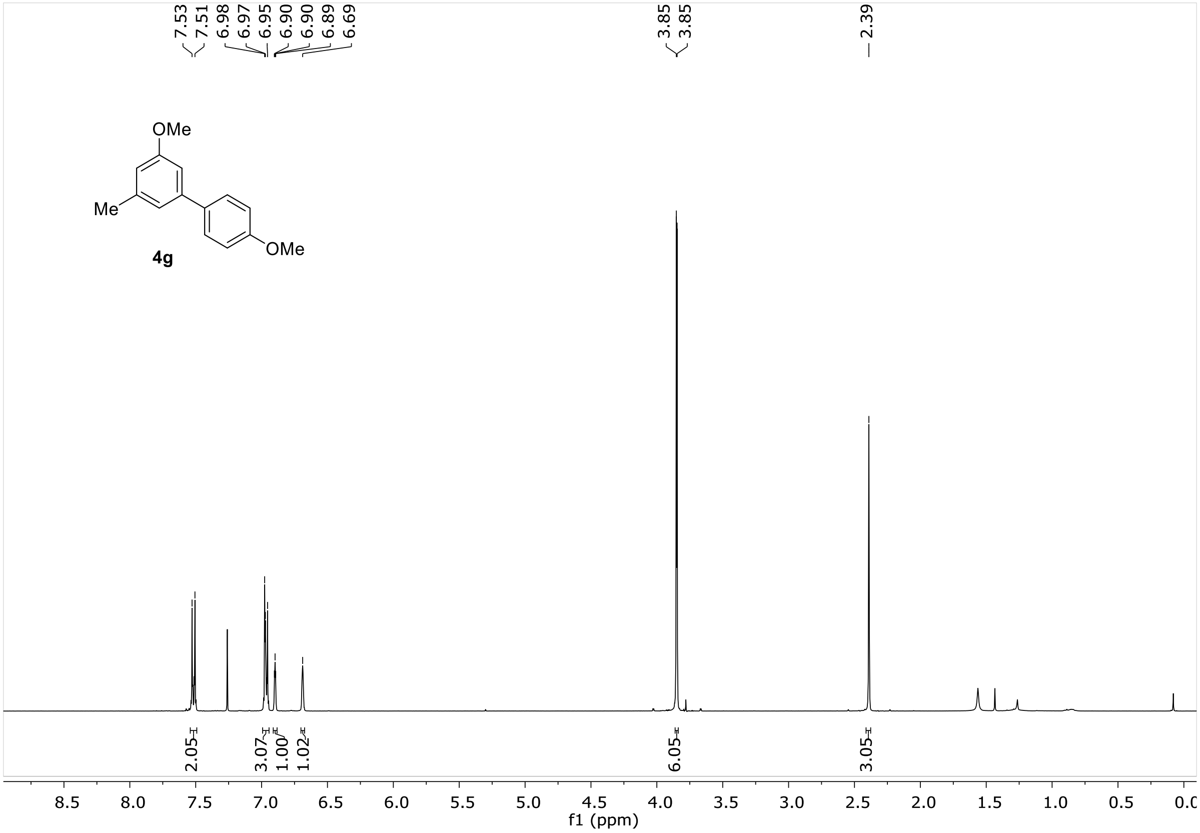
7.53
7.51
6.98
6.97
6.95
6.90
6.90
6.89
6.69
3.85
3.85
2.39
OMe
Me
OMe
4g
2.05
3.07
1.00
1.02
6.05
3.05
f1 (ppm)

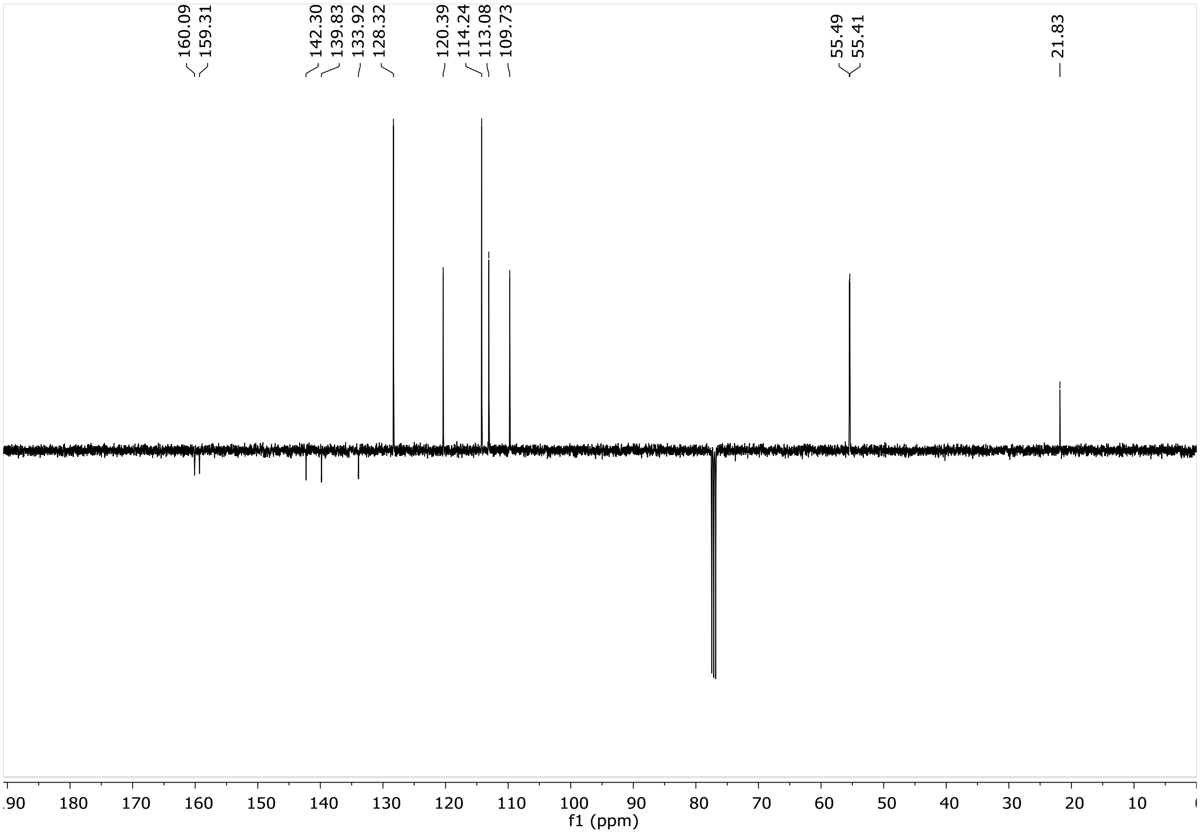
160.09
159.31
142.30
139.83
133.92
128.32
120.39
114.24
113.08
109.73
55.49
55.41
21.83
f1 (ppm)

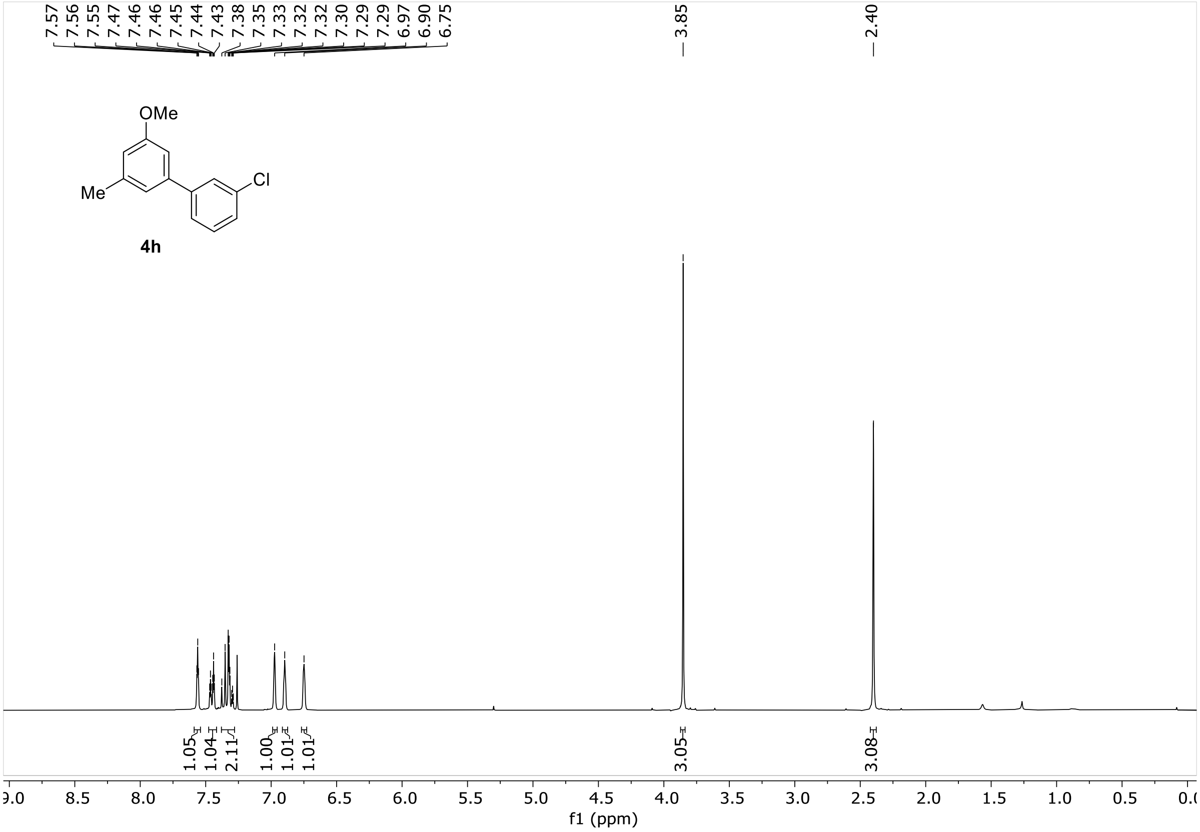
7.57
7.56
7.55
7.47
7.46
7.46
7.45
7.44
7.43
7.38
7.35
7.33
7.32
7.32
7.30
7.29
7.29
6.97
6.90
6.75
3.85
2.40
OMe
Me
Cl
4h
1.05
1.04
2.11
1.00
1.01
1.01
3.05
3.08
9.0
8.5
8.0
7.5
7.0
6.5
6.0
5.5
5.0
4.5
4.0
3.5
3.0
2.5
2.0
1.5
1.0
0.5
0.0
f1 (ppm)

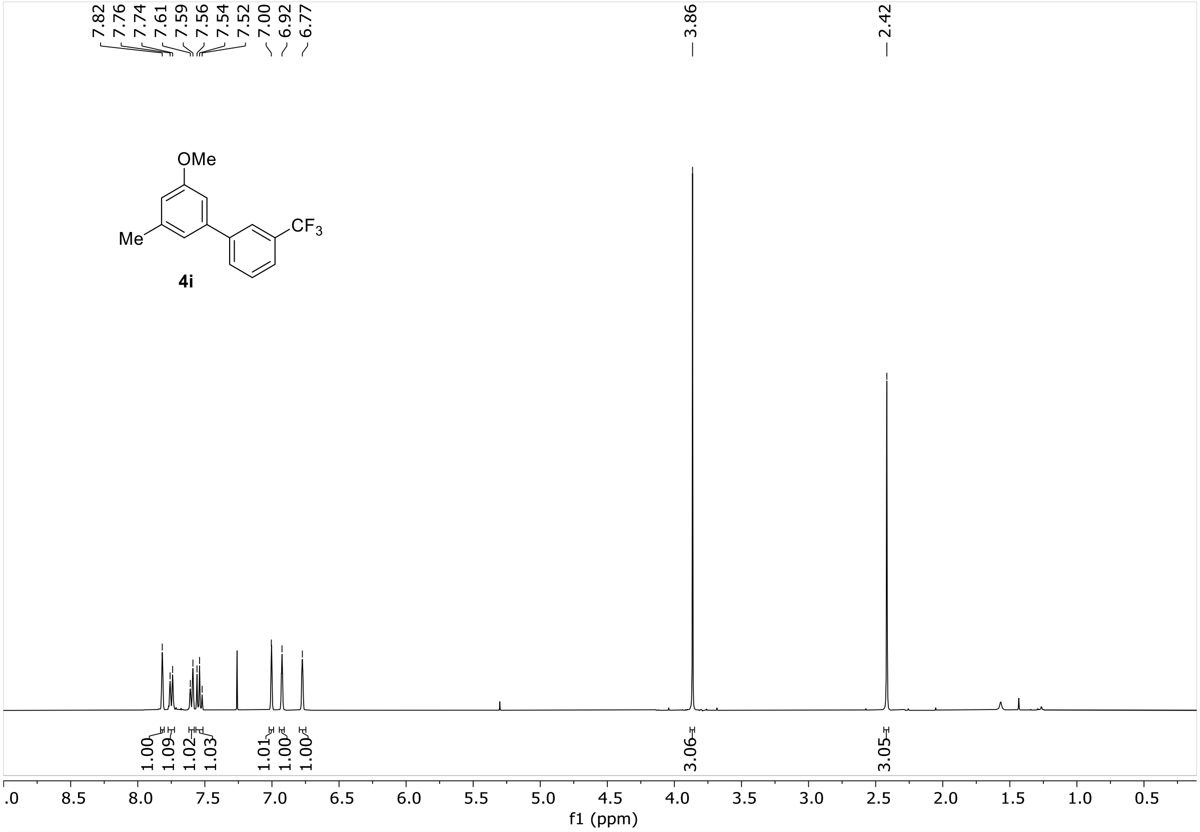
7.82
7.76
7.74
7.61
7.59
7.56
7.54
7.52
7.00
6.92
6.77
3.86
2.42
OMe
Me
CF3
4i
1.00
1.09
1.02
1.03
1.01
1.00
1.00
3.06
3.05
.0 8.5 8.0 7.5 7.0 6.5 6.0 5.5 5.0 4.5 4.0 3.5 3.0 2.5 2.0 1.5 1.0 0.5
f1 (ppm)

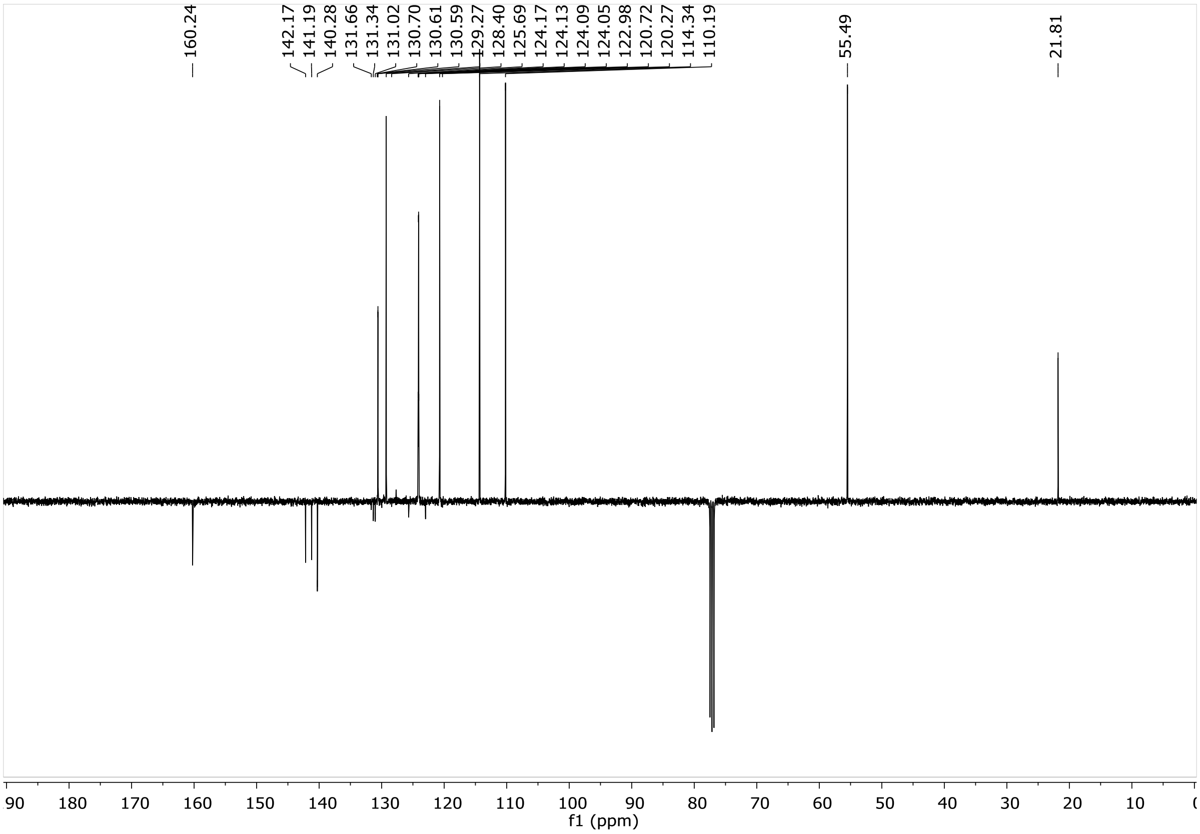
160.24
142.17
141.19
140.28
131.66
131.34
131.02
130.70
130.61
130.59
129.27
128.40
125.69
124.17
124.13
124.09
124.05
122.98
120.72
120.27
114.34
110.19
55.49
21.81
90 180 170 160 150 140 130 120 110 100 90 80 70 60 50 40 30 20 10
f1 (ppm)

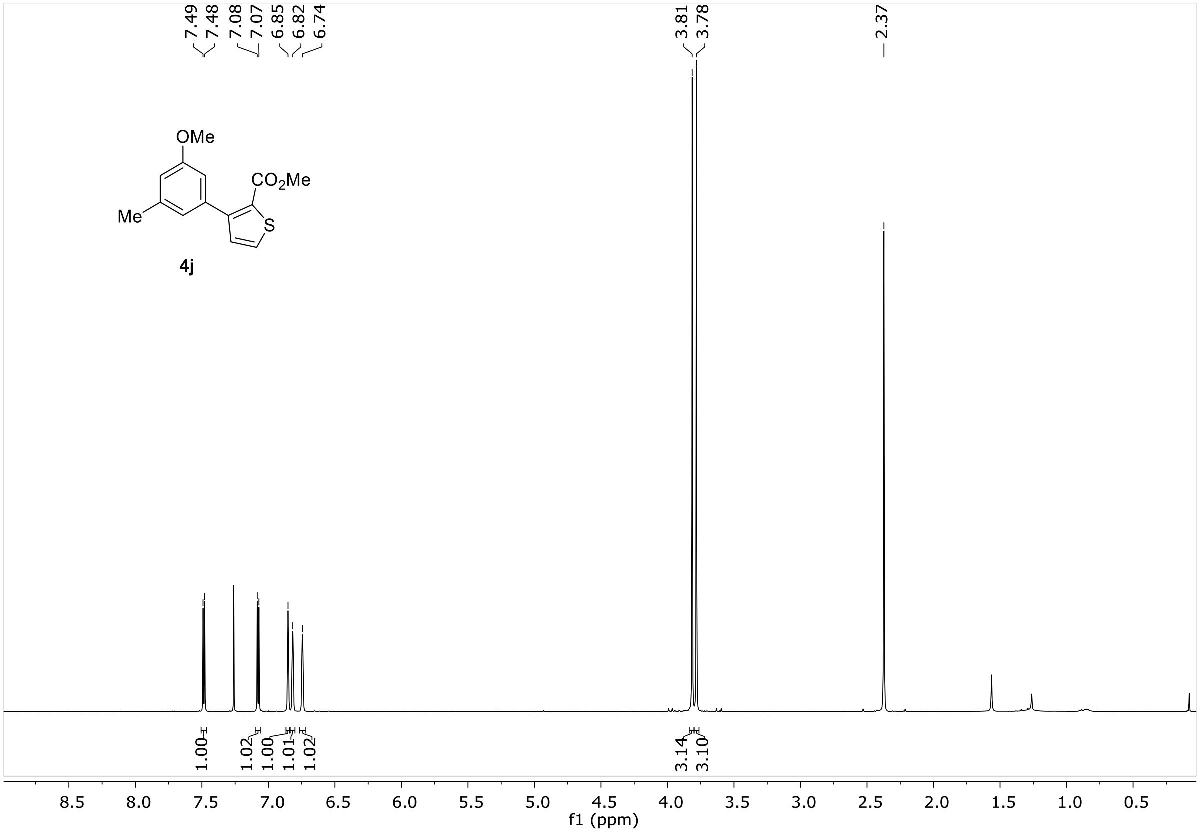
7.49
7.48
7.08
7.07
6.85
6.82
6.74
3.81
3.78
2.37
OMe
CO2Me
Me
S
4j
1.00
1.02
1.00
1.01
1.02
3.14
3.10
8.5 8.0 7.5 7.0 6.5 6.0 5.5 5.0 4.5 4.0 3.5 3.0 2.5 2.0 1.5 1.0 0.5
f1 (ppm)

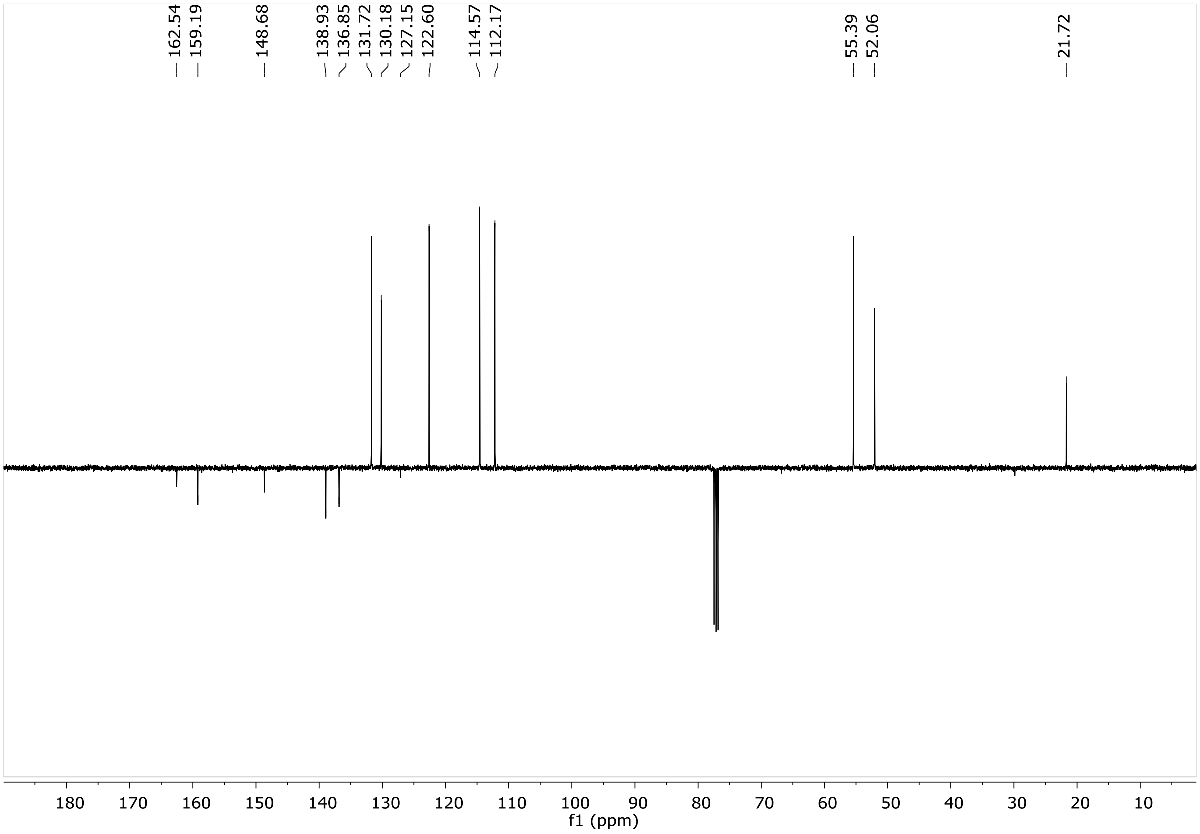
162.54
159.19
148.68
138.93
136.85
131.72
130.18
127.15
122.60
114.57
112.17
55.39
52.06
21.72
180 170 160 150 140 130 120 110 100 90 80 70 60 50 40 30 20 10
f1 (ppm)

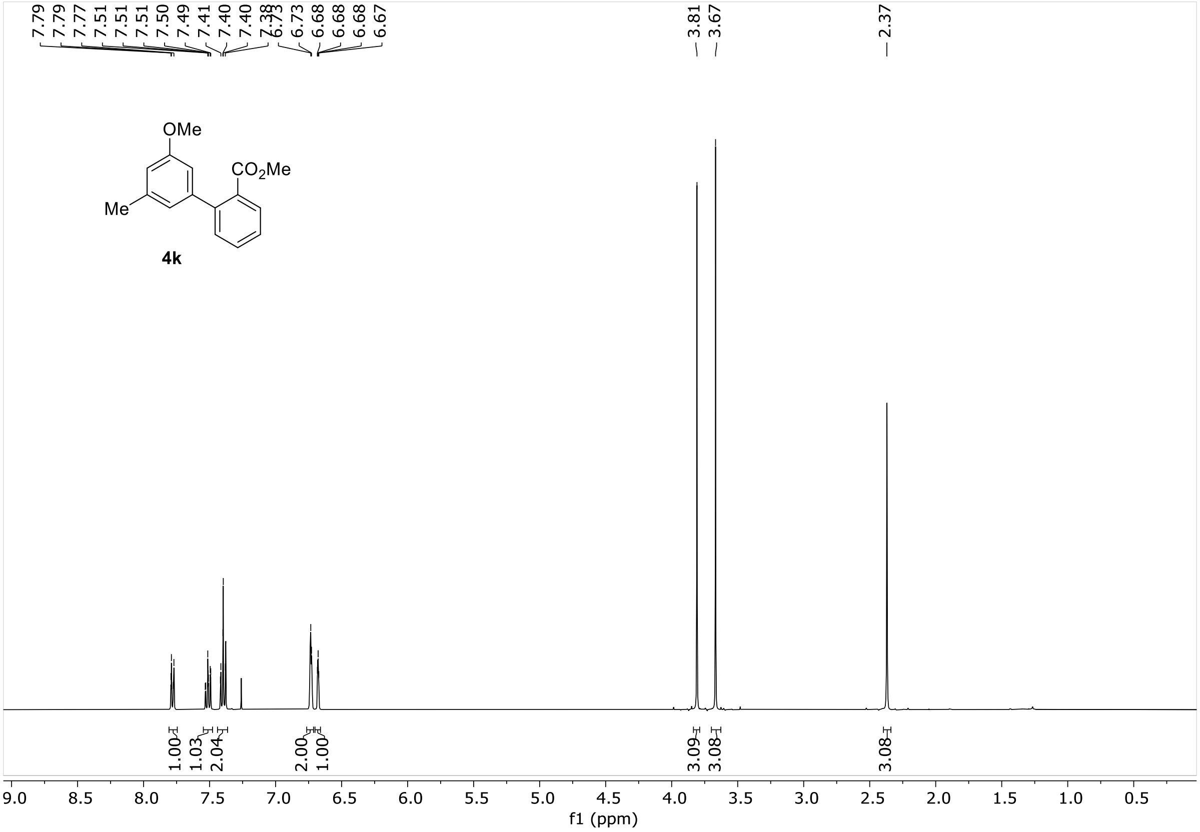
7.79
7.79
7.77
7.51
7.51
7.51
7.50
7.49
7.41
7.40
7.40
7.38
6.73
6.73
6.68
6.68
6.68
6.67
3.81
3.67
2.37
OMe
CO_2Me
Me
4k
1.00
1.03
2.04
2.00
1.00
3.09
3.08
3.08
9.0
8.5
8.0
7.5
7.0
6.5
6.0
5.5
5.0
4.5
4.0
3.5
3.0
2.5
2.0
1.5
1.0
0.5
f1 (ppm)

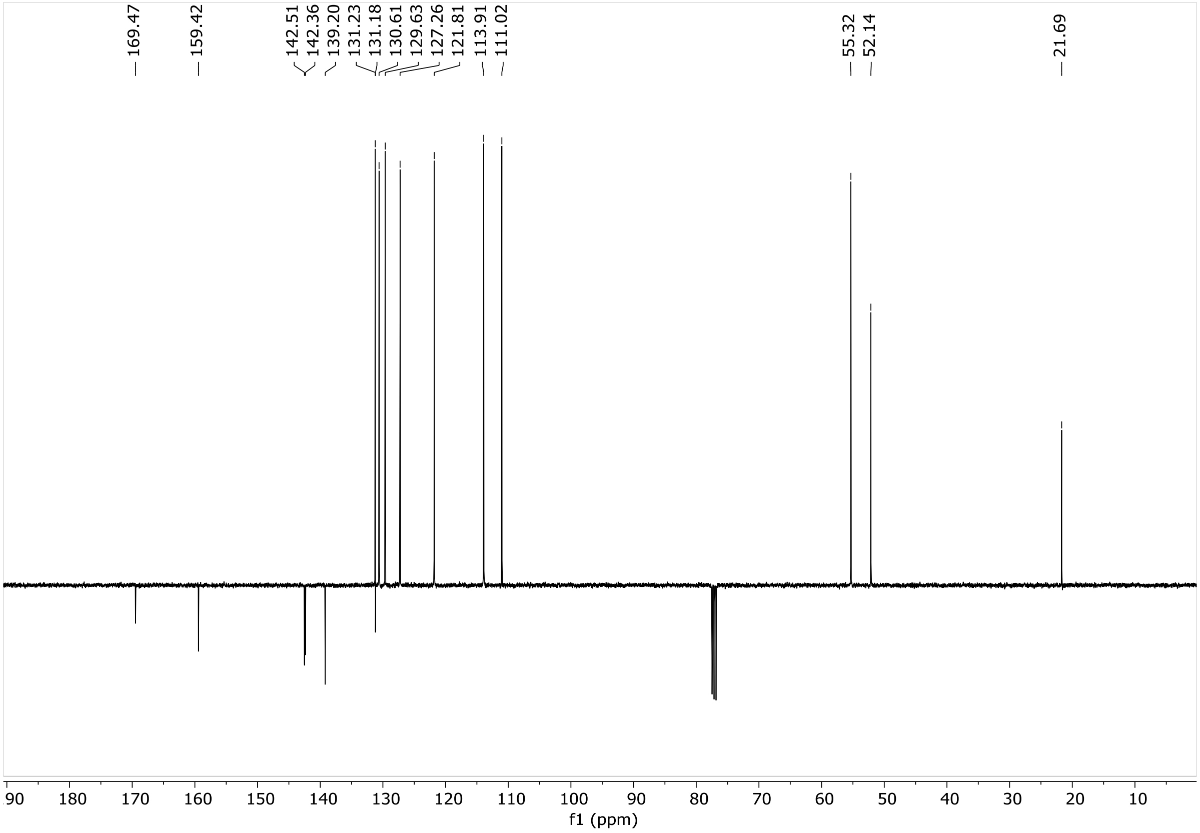
169.47
159.42
142.51
142.36
139.20
131.23
131.18
130.61
129.63
127.26
121.81
113.91
111.02
55.32
52.14
21.69
90
180
170
160
150
140
130
120
110
100
90
80
70
60
50
40
30
20
10
f1 (ppm)

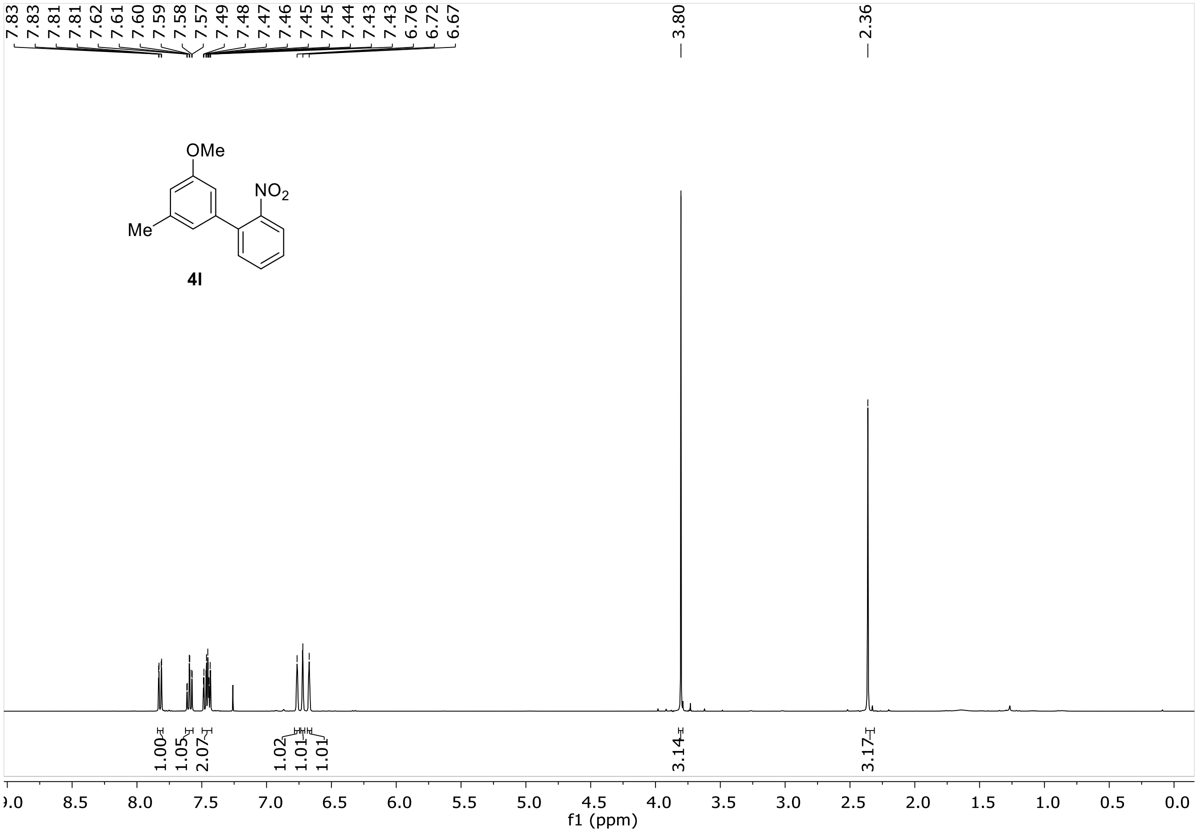
7.83 7.83 7.81 7.81 7.62 7.61 7.60 7.59 7.58 7.57 7.49 7.48 7.47 7.46 7.45 7.45 7.44 7.43 7.43 6.76 6.72 6.67
3.80
2.36
OMe
NO_2
Me
4l
1.00 1.05 2.07 1.02 1.01 1.01 3.14 3.17
f1 (ppm)

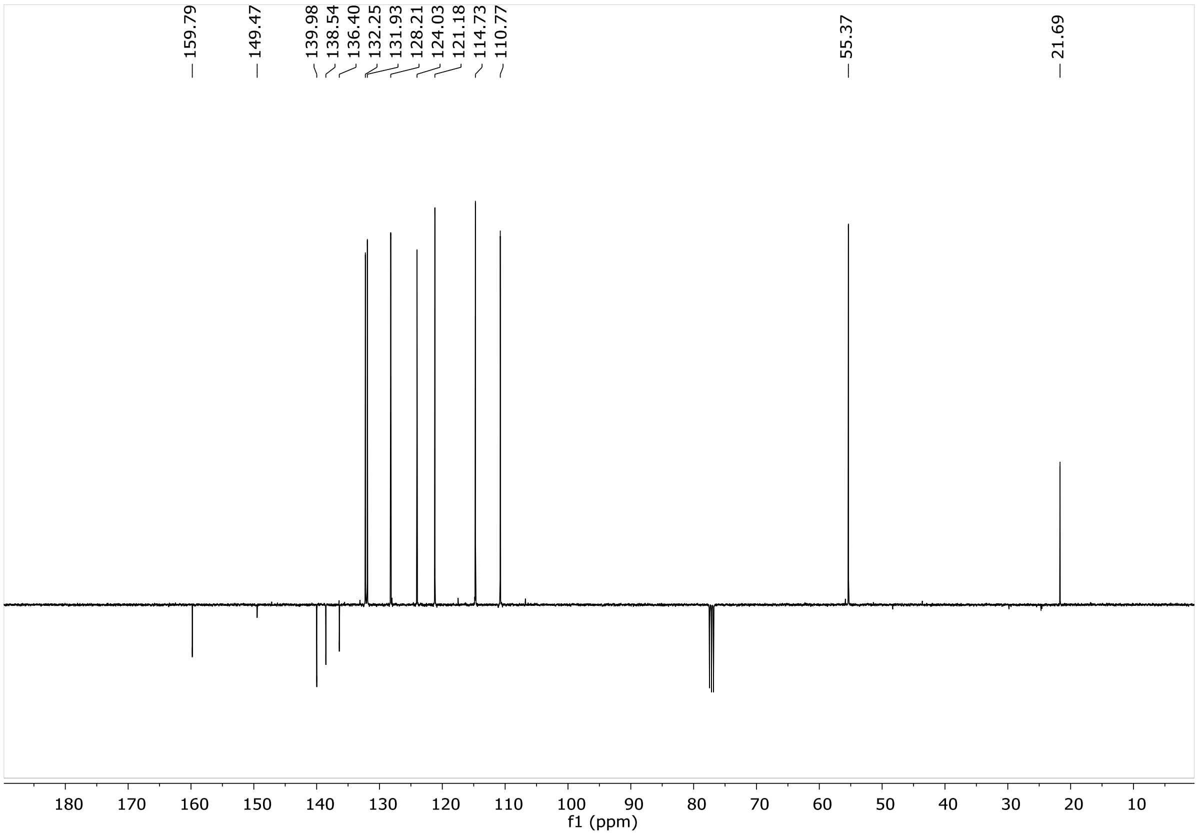
159.79
149.47
139.98 138.54 136.40 132.25 131.93 128.21 124.03 121.18 114.73 110.77
55.37
21.69
f1 (ppm)

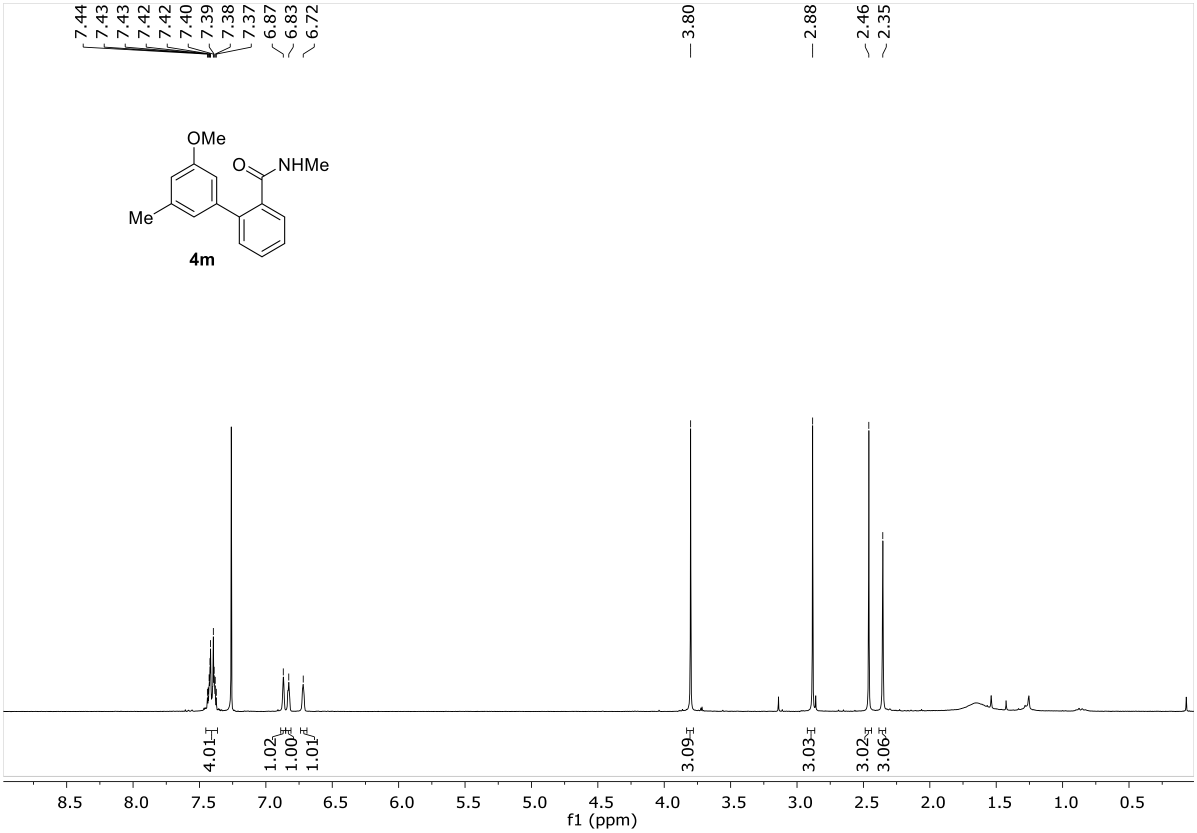
7.44
7.43
7.43
7.42
7.42
7.40
7.39
7.38
7.37
6.87
6.83
6.72
3.80
2.88
2.46
2.35
OMe
O
NHMe
Me
4m
4.01
1.02
1.00
1.01
3.09
3.03
3.02
3.06
8.5 8.0 7.5 7.0 6.5 6.0 5.5 5.0 4.5 4.0 3.5 3.0 2.5 2.0 1.5 1.0 0.5
f1 (ppm)

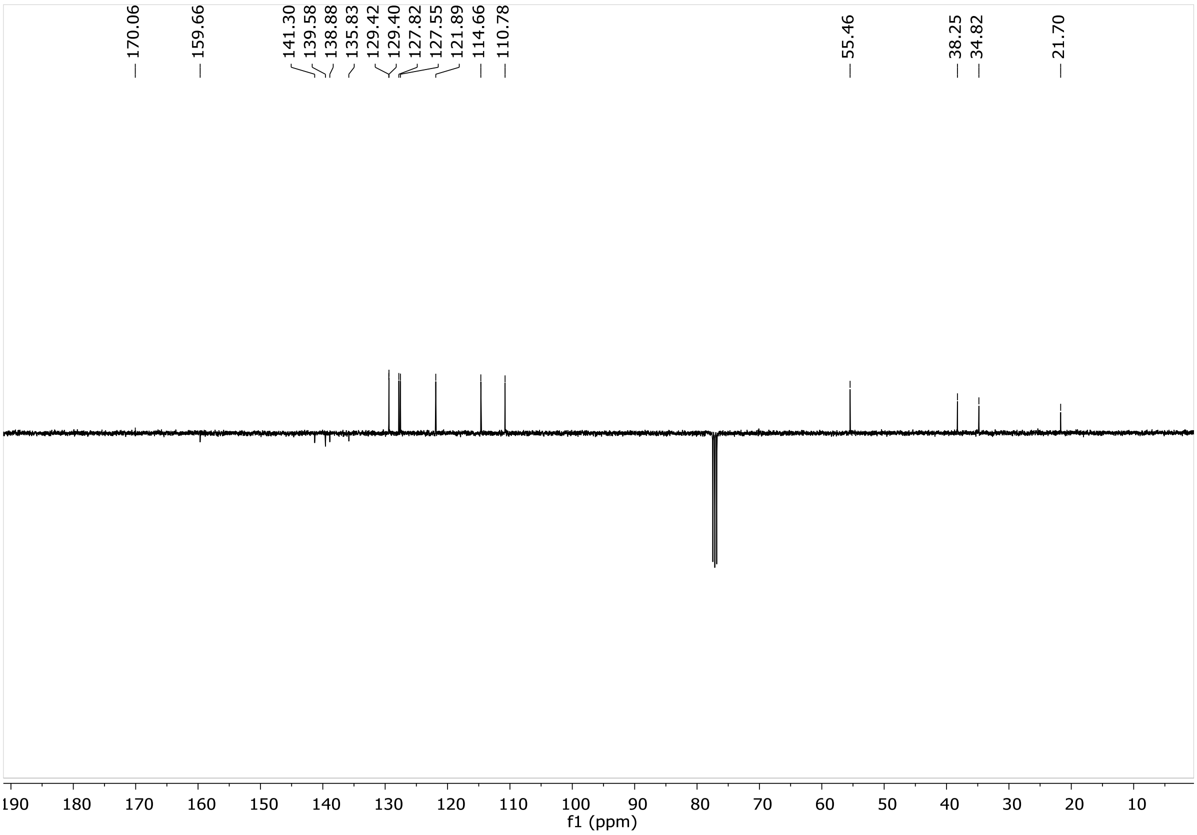
170.06
159.66
141.30
139.58
138.88
135.83
129.42
129.40
127.82
127.55
121.89
114.66
110.78
55.46
38.25
34.82
21.70
190 180 170 160 150 140 130 120 110 100 90 80 70 60 50 40 30 20 10
f1 (ppm)

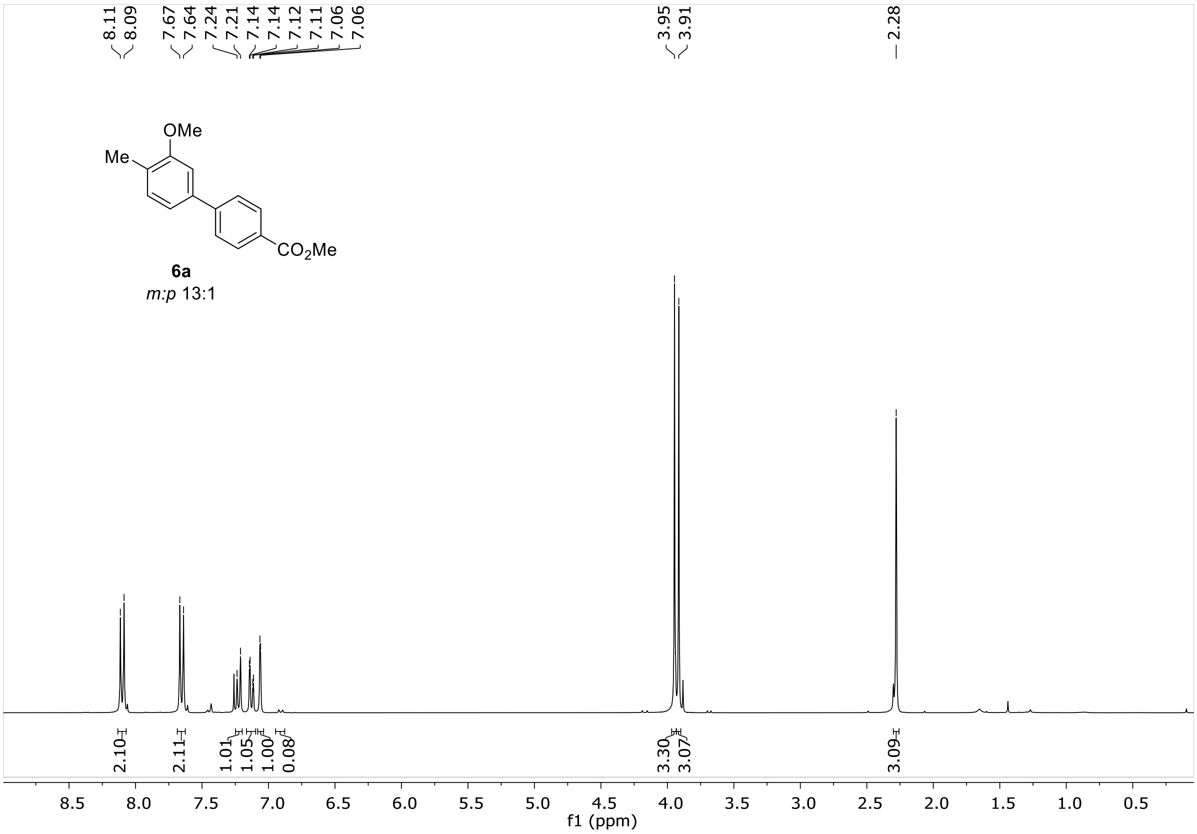
8.11
8.09
7.67
7.64
7.24
7.21
7.14
7.14
7.12
7.11
7.06
7.06
3.95
3.91
2.28
OMe
Me
CO2Me
6a
m:p 13:1
2.10
2.11
1.01
1.05
1.00
0.08
3.30
3.07
3.09
8.5
8.0
7.5
7.0
6.5
6.0
5.5
5.0
4.5
4.0
3.5
3.0
2.5
2.0
1.5
1.0
0.5
f1 (ppm)

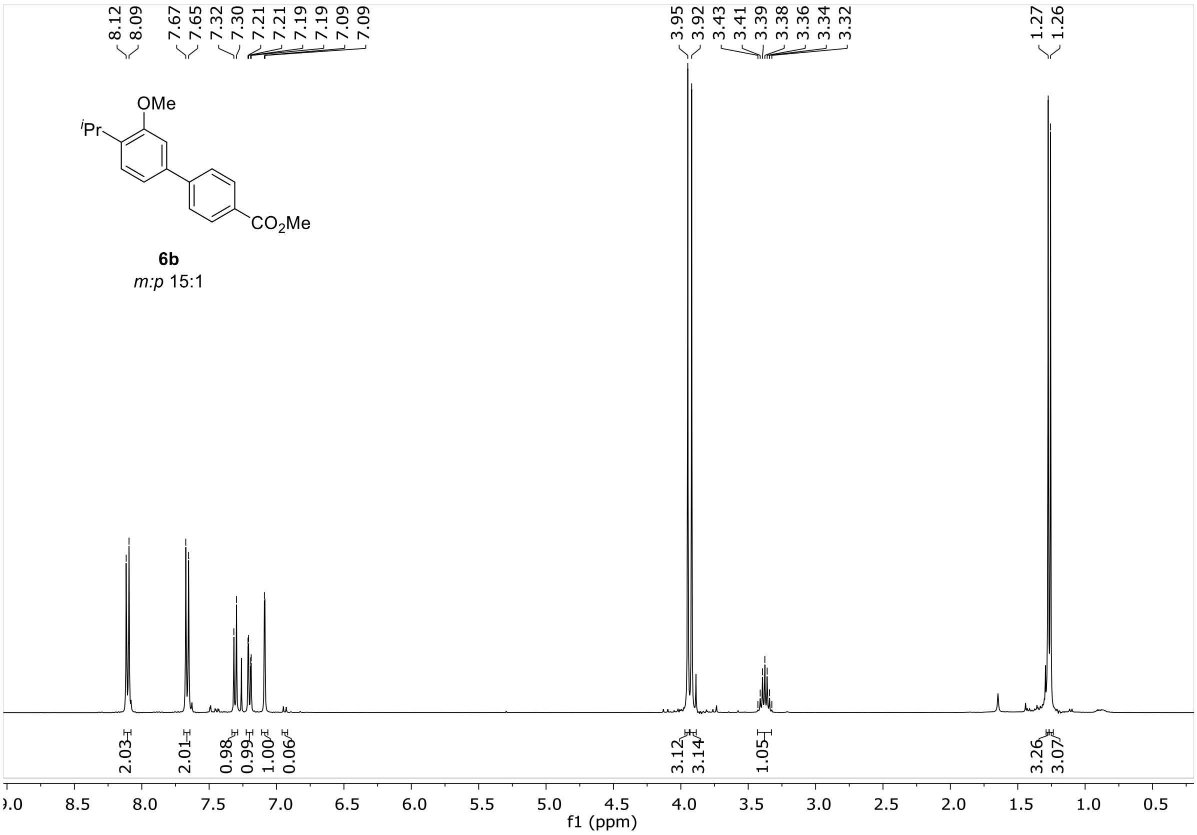
8.12
8.09
7.67
7.65
7.32
7.30
7.21
7.21
7.19
7.19
7.09
7.09
3.95
3.92
3.43
3.41
3.39
3.38
3.36
3.34
3.32
1.27
1.26
OMe
iPr
CO2Me
6b
m:p 15:1
2.03
2.01
0.98
0.99
1.00
0.06
3.12
3.14
1.05
3.26
3.07
8.5
8.0
7.5
7.0
6.5
6.0
5.5
5.0
4.5
4.0
3.5
3.0
2.5
2.0
1.5
1.0
0.5
f1 (ppm)

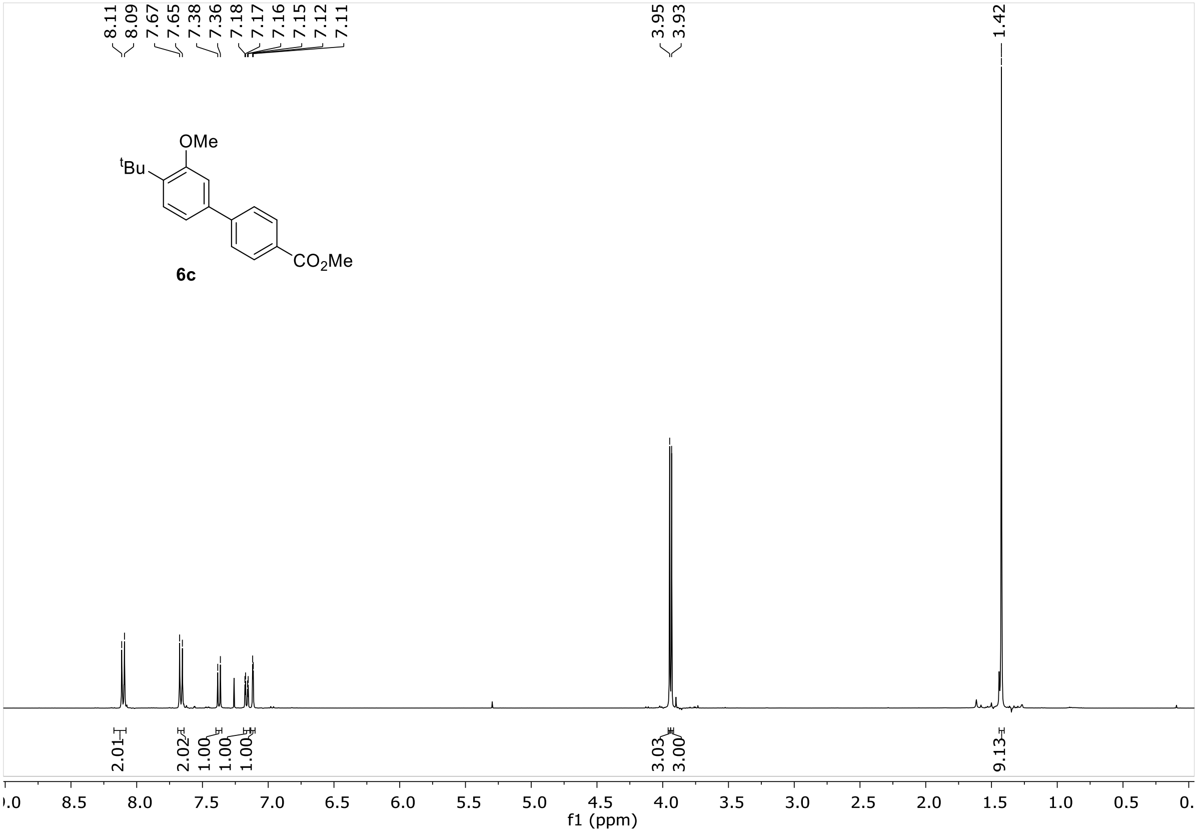
8.11
8.09
7.67
7.65
7.38
7.36
7.18
7.17
7.16
7.15
7.12
7.11
3.95
3.93
1.42
OMe
tBu
CO2Me
6c
2.01
2.02
1.00
1.00
1.00
3.03
3.00
9.13
f1 (ppm)

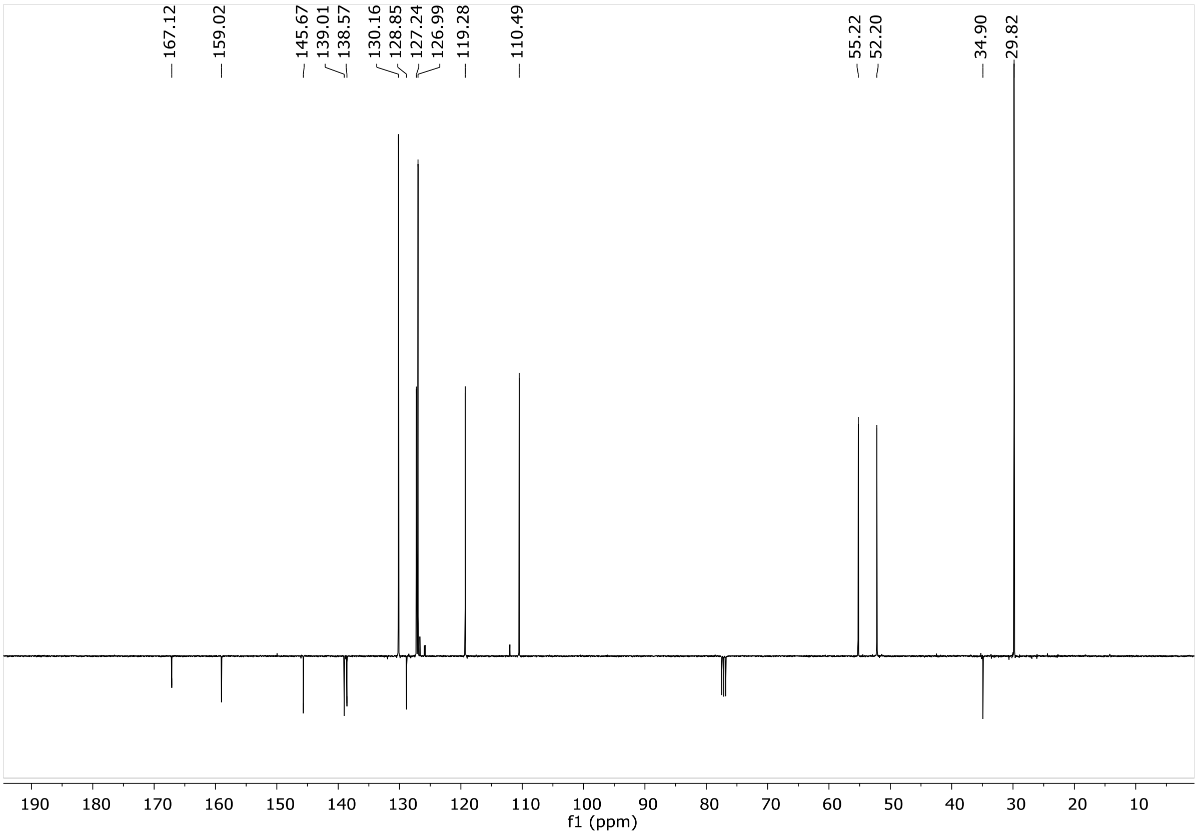
167.12
159.02
145.67
139.01
138.57
130.16
128.85
127.24
126.99
119.28
110.49
55.22
52.20
34.90
29.82
f1 (ppm)

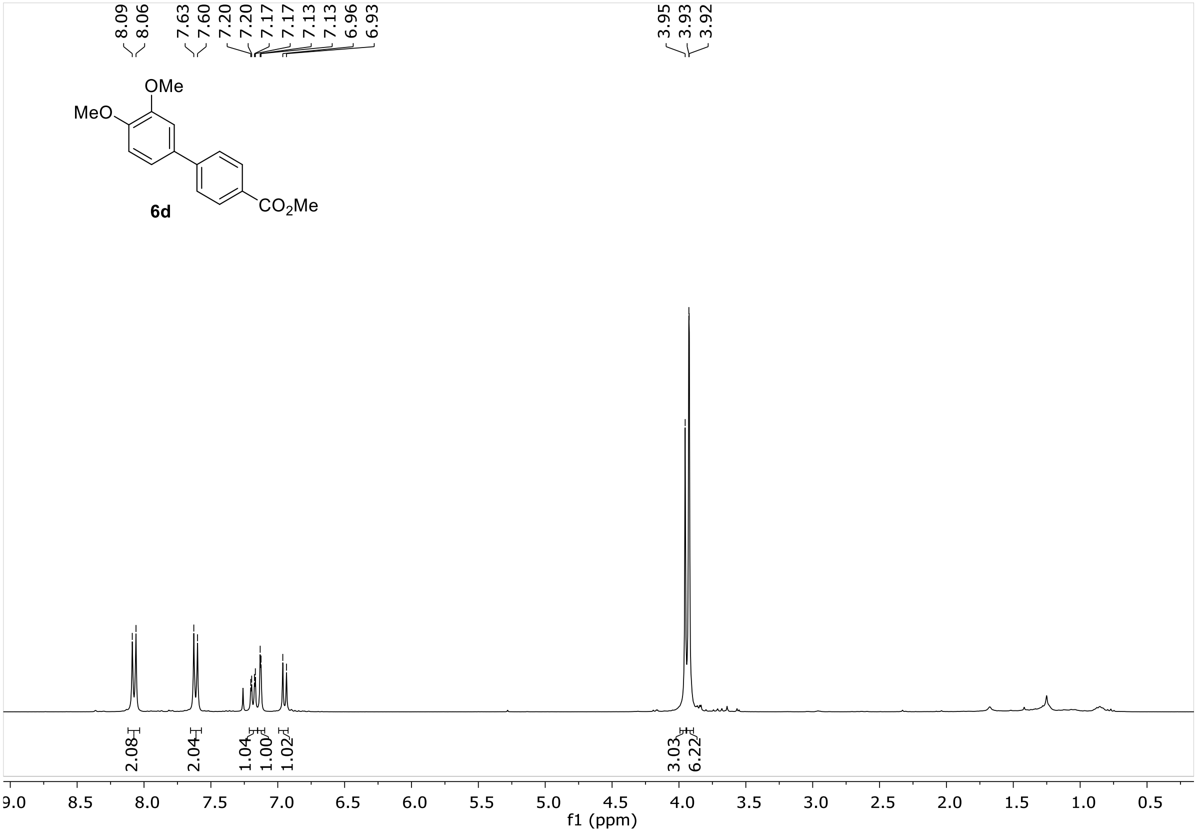
8.09
8.06
7.63
7.60
7.20
7.20
7.17
7.17
7.13
7.13
6.96
6.93
3.95
3.93
3.92
OMe
MeO
CO2Me
6d
2.08
2.04
1.04
1.00
1.02
3.03
6.22
9.0 8.5 8.0 7.5 7.0 6.5 6.0 5.5 5.0 4.5 4.0 3.5 3.0 2.5 2.0 1.5 1.0 0.5
f1 (ppm)

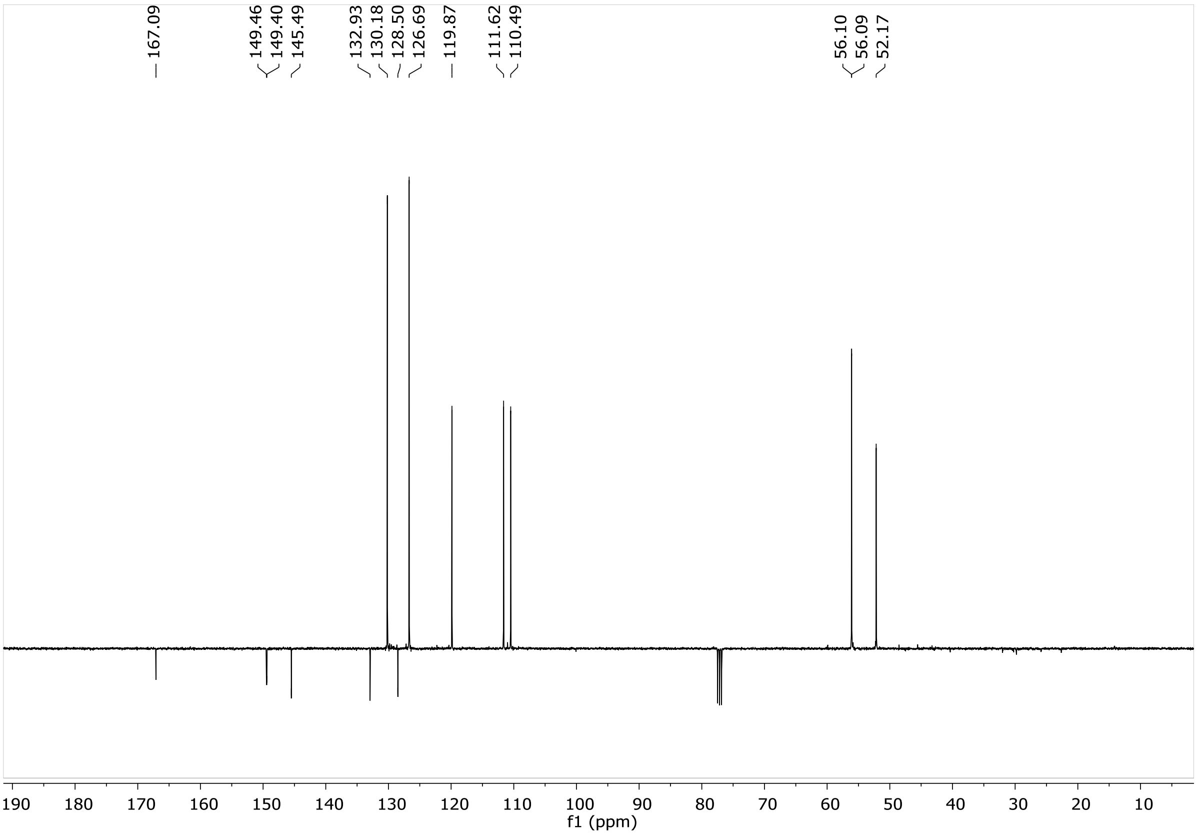
167.09
149.46
149.40
145.49
132.93
130.18
128.50
126.69
119.87
111.62
110.49
56.10
56.09
52.17
190 180 170 160 150 140 130 120 110 100 90 80 70 60 50 40 30 20 10
f1 (ppm)

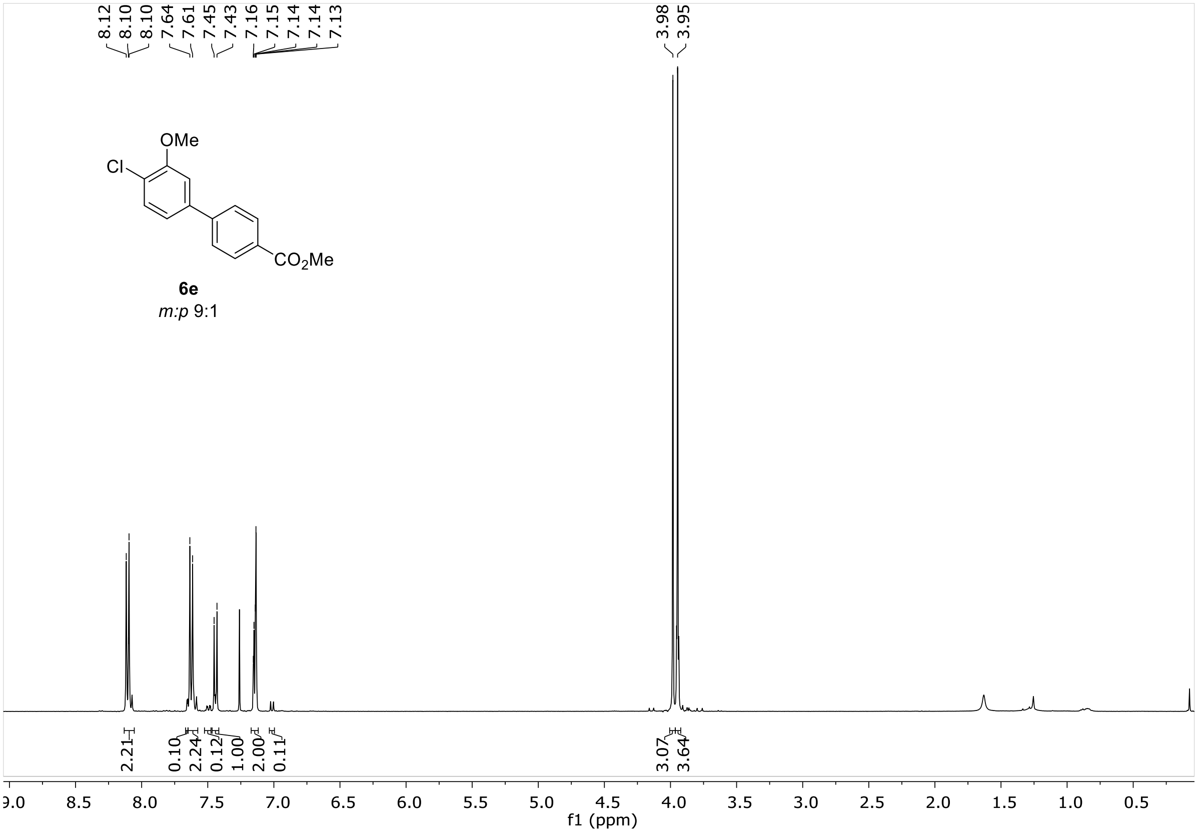
8.12
8.10
8.10
7.64
7.61
7.45
7.43
7.16
7.15
7.14
7.14
7.13
3.98
3.95
OMe
Cl
CO2Me
6e
m:p 9:1
2.21
0.10
2.24
0.12
1.00
2.00
0.11
3.07
3.64
9.0 8.5 8.0 7.5 7.0 6.5 6.0 5.5 5.0 4.5 4.0 3.5 3.0 2.5 2.0 1.5 1.0 0.5
f1 (ppm)

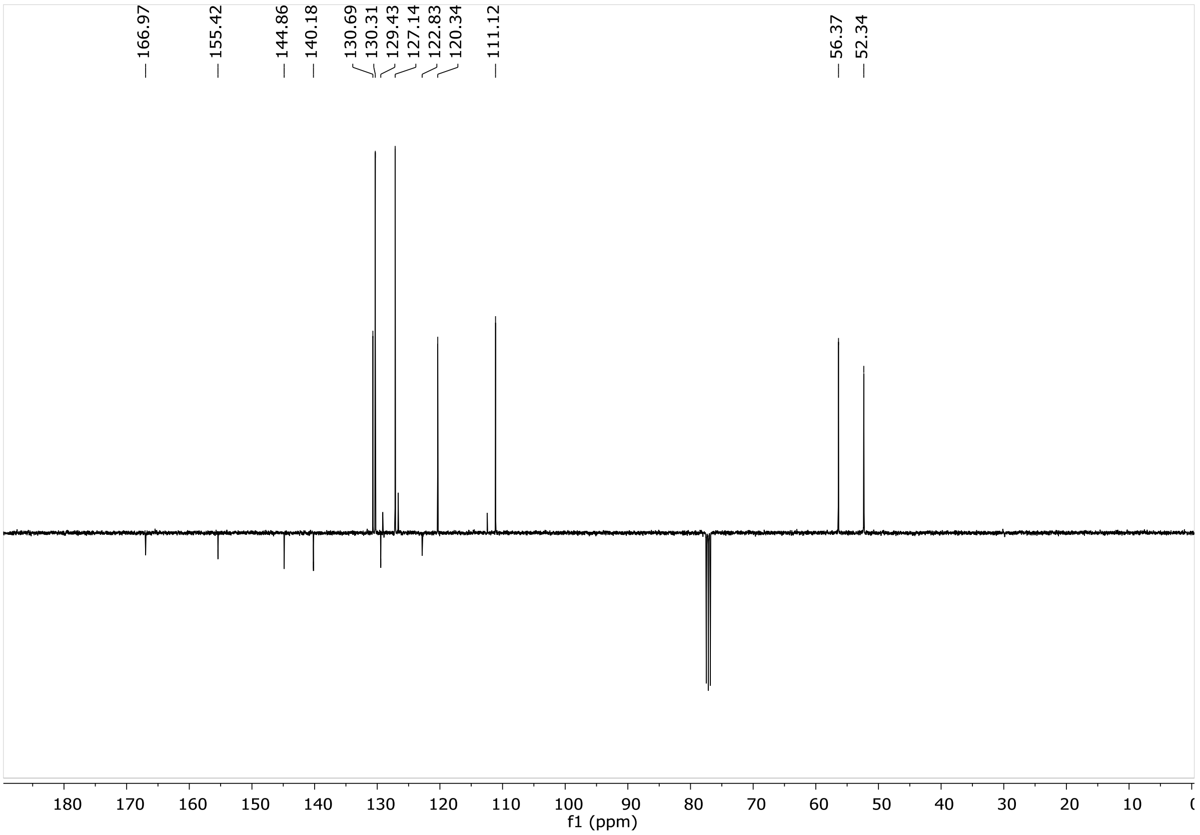
166.97
155.42
144.86
140.18
130.69
130.31
129.43
127.14
122.83
120.34
111.12
56.37
52.34
180 170 160 150 140 130 120 110 100 90 80 70 60 50 40 30 20 10
f1 (ppm)

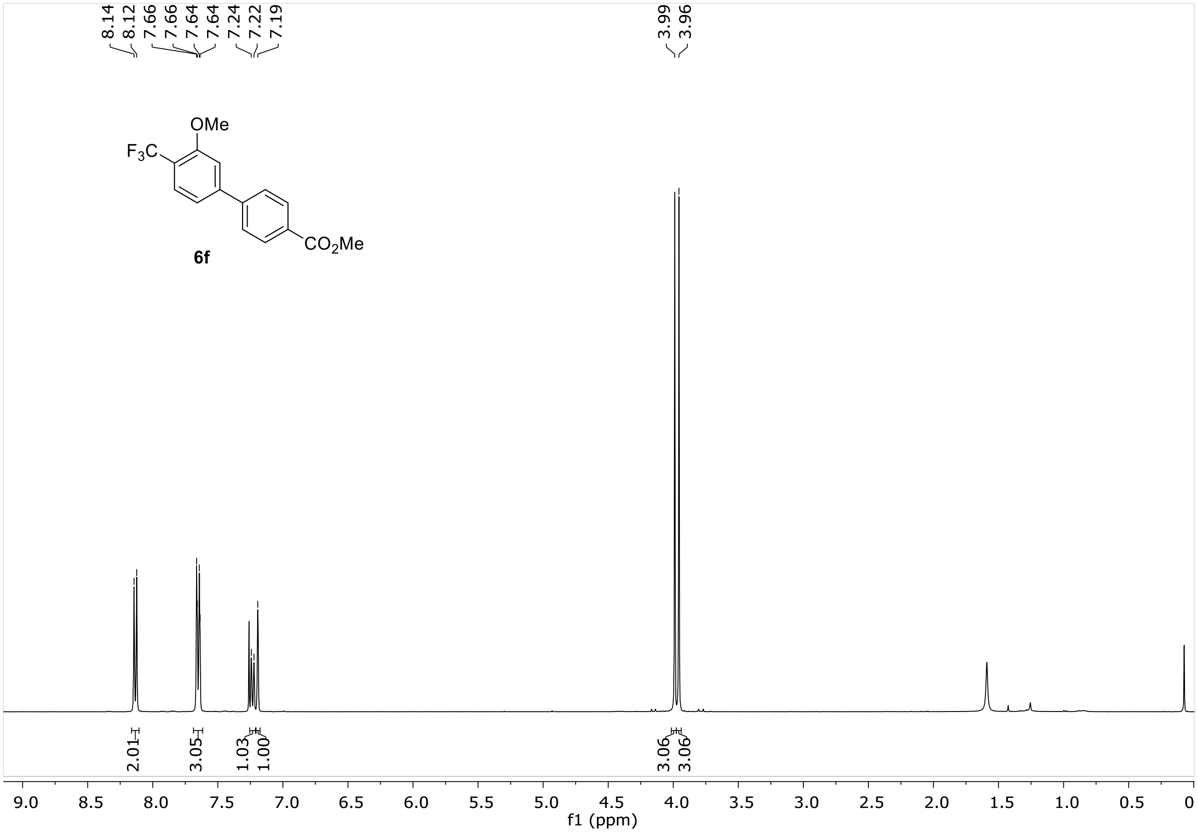
8.14
8.12
7.66
7.66
7.64
7.64
7.24
7.22
7.19
3.99
3.96
OMe
F3C
CO2Me
6f
2.01
3.05
1.03
1.00
3.06
3.06
f1 (ppm)

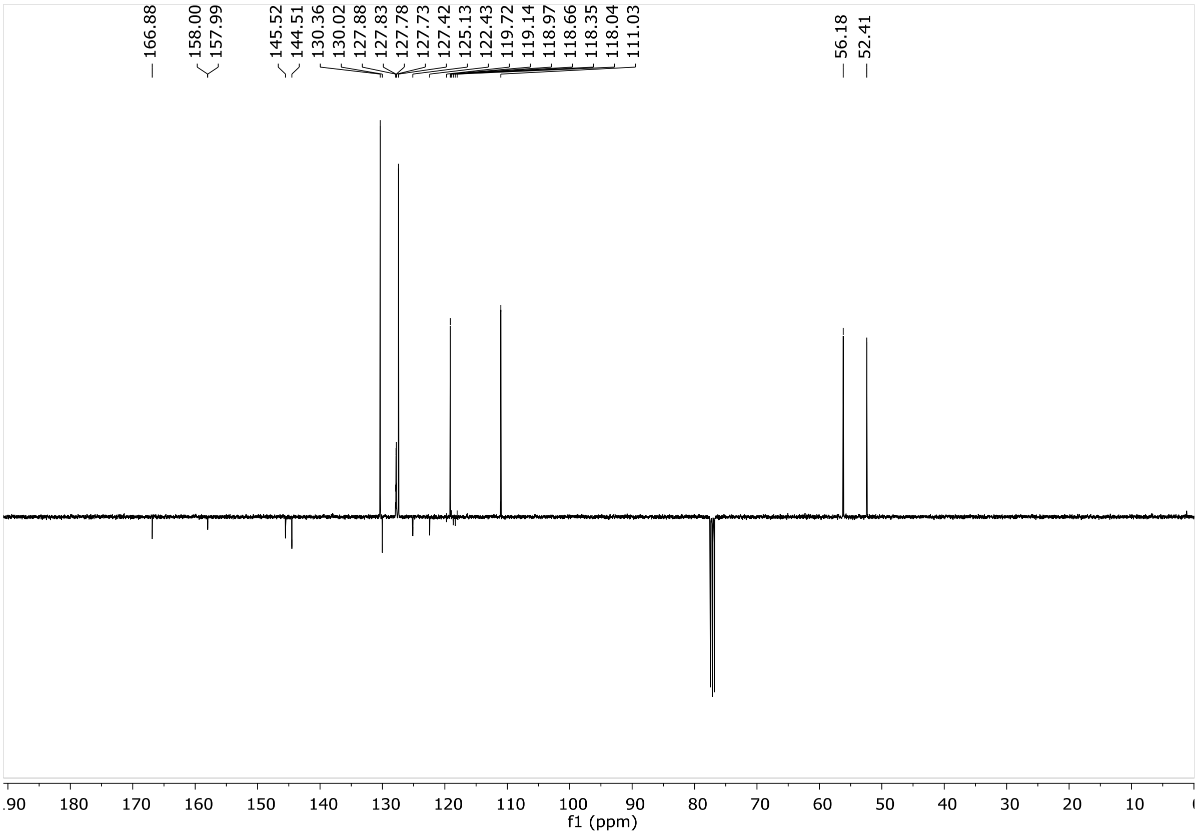
166.88
158.00
157.99
145.52
144.51
130.36
130.02
127.88
127.83
127.78
127.73
127.42
125.13
122.43
119.72
119.14
118.97
118.66
118.35
118.04
111.03
56.18
52.41
f1 (ppm)

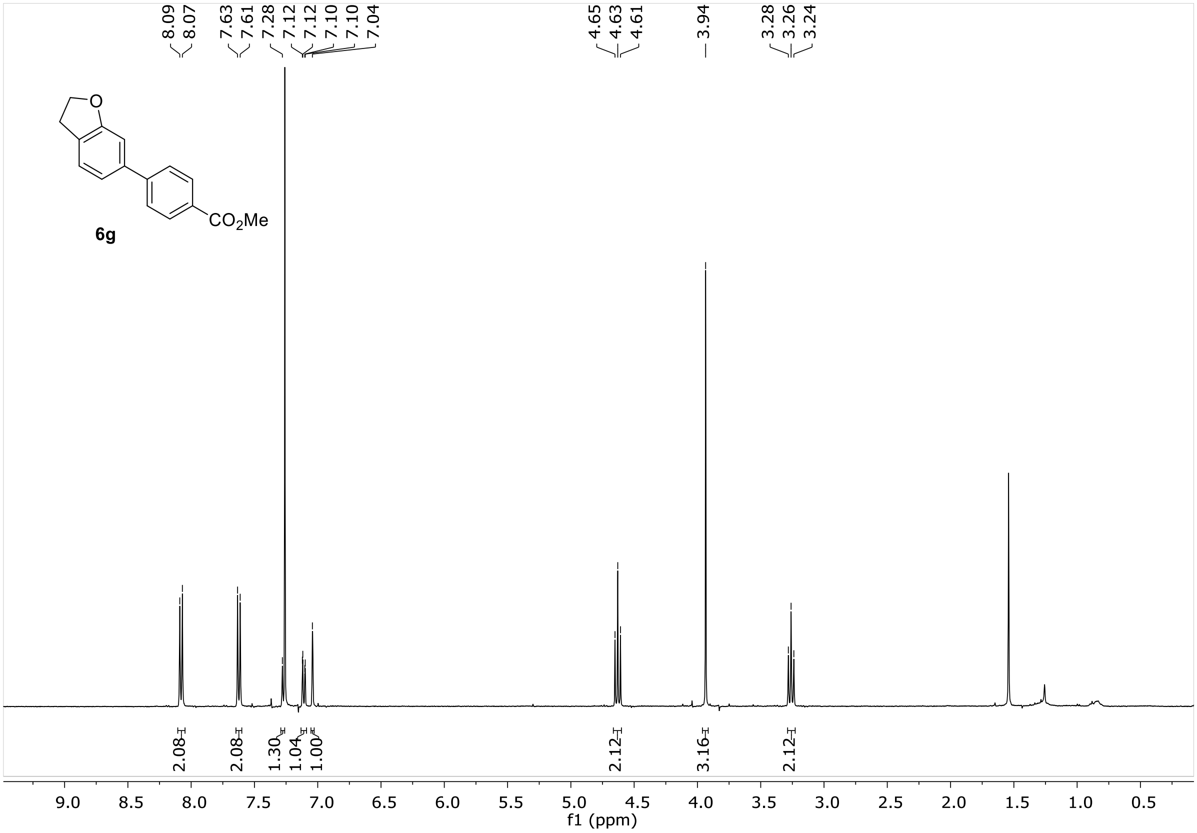
8.09
8.07
7.63
7.61
7.28
7.12
7.12
7.10
7.10
7.04
4.65
4.63
4.61
3.94
3.28
3.26
3.24
O
CO_2Me
6g
2.08
2.08
1.30
1.04
1.00
2.12
3.16
2.12
9.0
8.5
8.0
7.5
7.0
6.5
6.0
5.5
5.0
4.5
4.0
3.5
3.0
2.5
2.0
1.5
1.0
0.5
f1 (ppm)

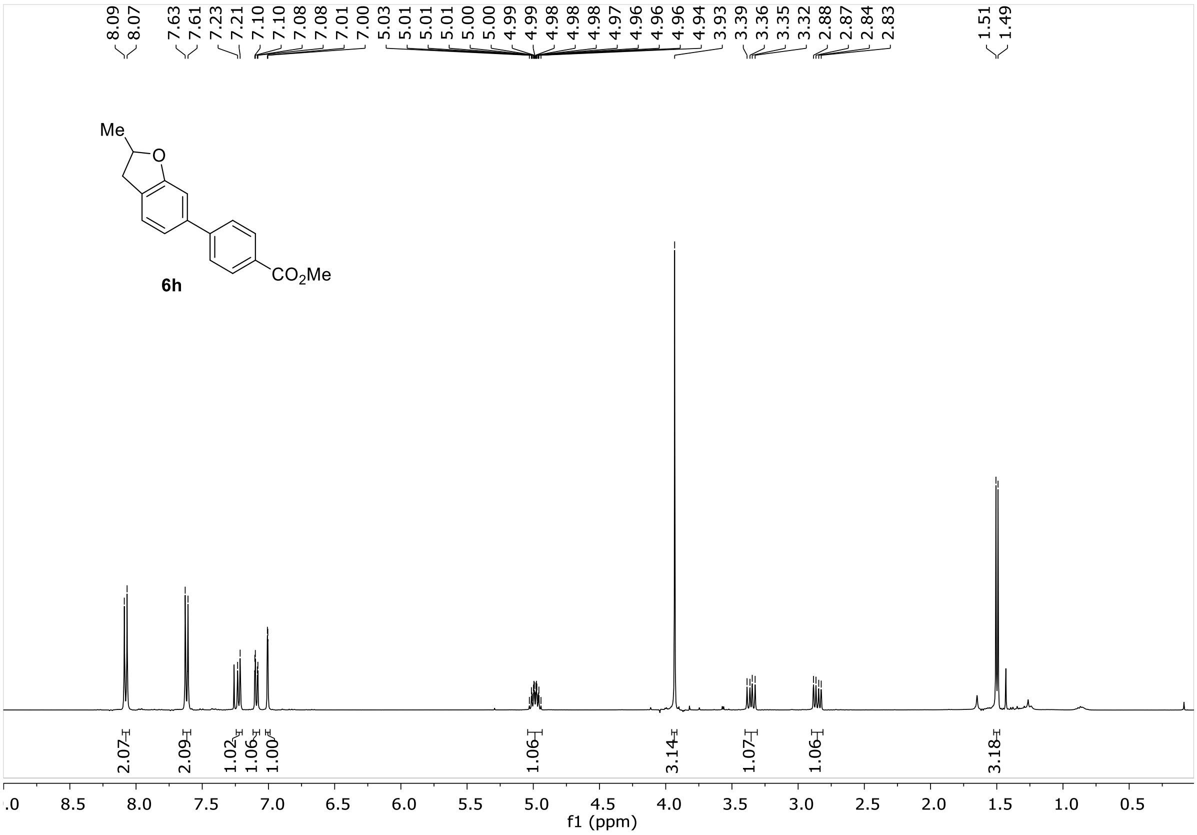
8.09
8.07
7.63
7.61
7.23
7.21
7.10
7.10
7.08
7.08
7.01
7.00
5.03
5.01
5.01
5.00
5.00
4.99
4.99
4.98
4.98
4.97
4.96
4.96
4.94
3.93
3.39
3.36
3.35
3.32
2.88
2.87
2.84
2.83
1.51
1.49
Me
O
CO_2Me
6h
2.07
2.09
1.02
1.06
1.00
1.06
3.14
1.07
1.06
3.18
.0
8.5
8.0
7.5
7.0
6.5
6.0
5.5
5.0
4.5
4.0
3.5
3.0
2.5
2.0
1.5
1.0
0.5
f1 (ppm)

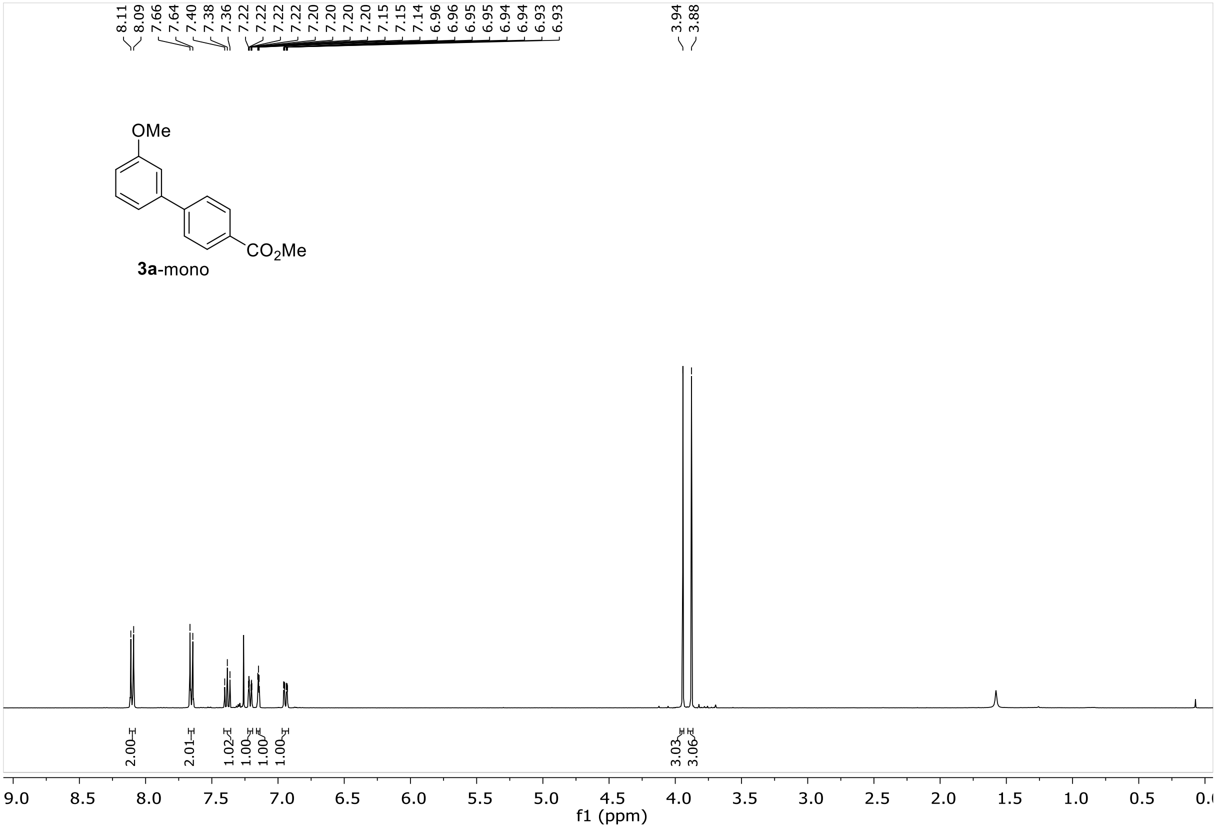
8.11
8.09
7.66
7.64
7.40
7.38
7.36
7.22
7.22
7.22
7.22
7.20
7.20
7.20
7.20
7.15
7.15
7.14
6.96
6.96
6.95
6.95
6.94
6.94
6.93
6.93
3.94
3.88
OMe
CO2Me
3a-mono
2.00
2.01
1.02
1.00
1.00
1.00
3.03
3.06
9.0
8.5
8.0
7.5
7.0
6.5
6.0
5.5
5.0
4.5
4.0
3.5
3.0
2.5
2.0
1.5
1.0
0.5
f1 (ppm)

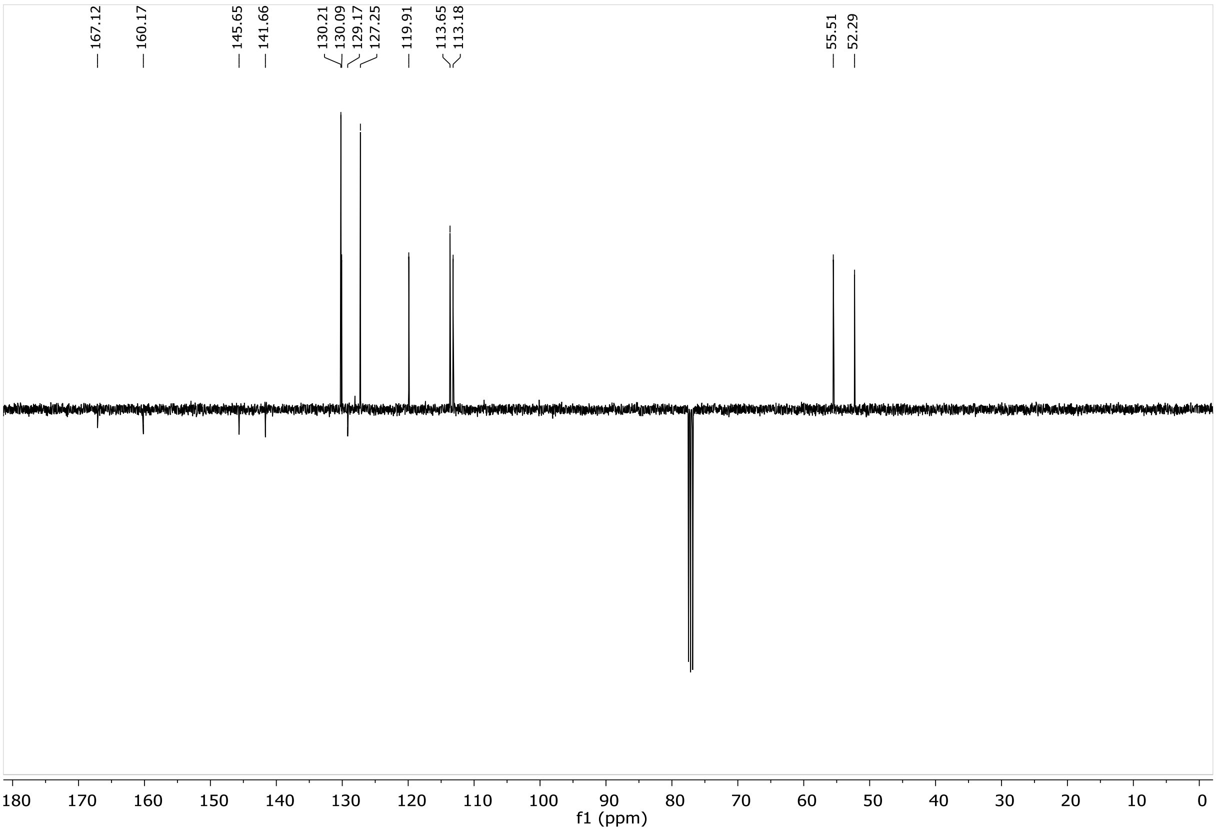
167.12
160.17
145.65
141.66
130.21
130.09
129.17
127.25
119.91
113.65
113.18
55.51
52.29
180
170
160
150
140
130
120
110
100
90
80
70
60
50
40
30
20
10
0
f1 (ppm)

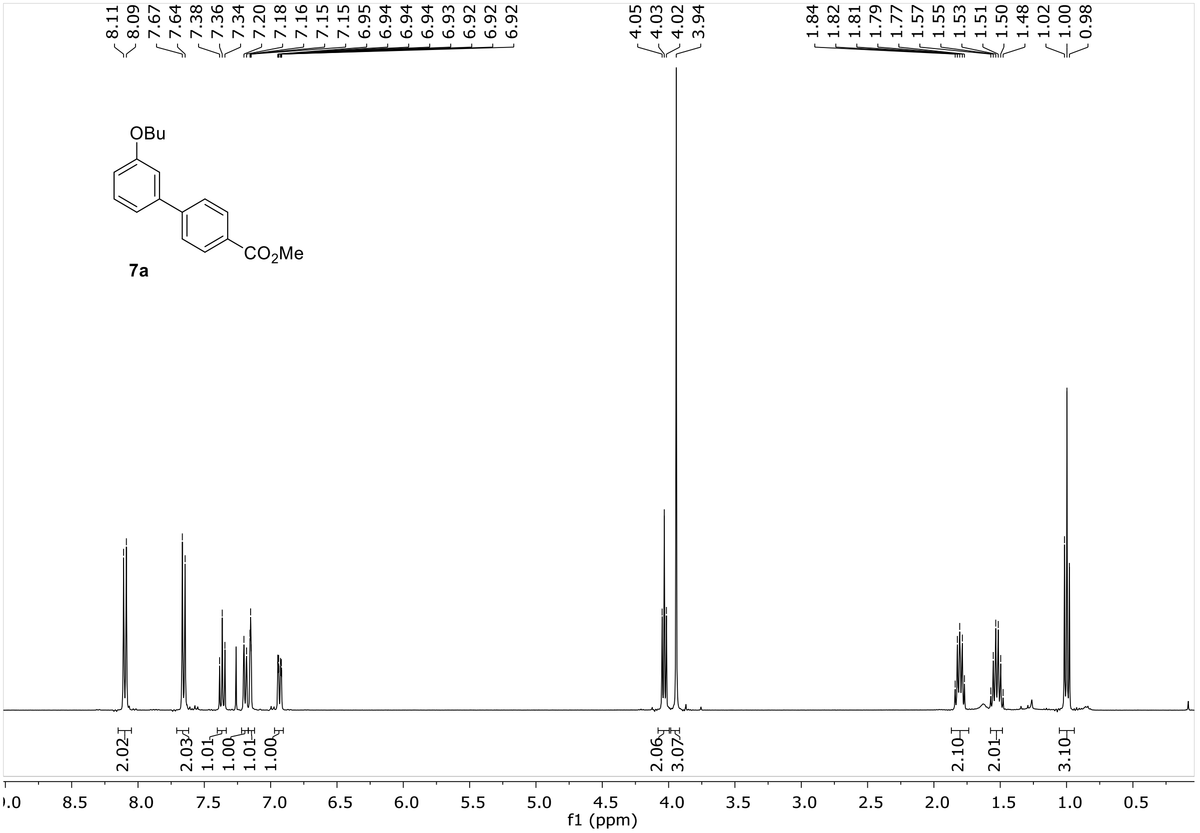
8.11 8.09 7.67 7.64 7.38 7.36 7.34 7.20 7.18 7.16 7.15 7.15 6.95 6.94 6.94 6.94 6.93 6.92 6.92 6.92
4.05 4.03 4.02 3.94
1.84 1.82 1.81 1.79 1.77 1.57 1.55 1.53 1.51 1.50 1.48 1.02 1.00 0.98
OBu
CO_2Me
7a
2.02 2.03 1.01 1.00 1.01 1.00 2.06 3.07 2.10 2.01 3.10
f1 (ppm)

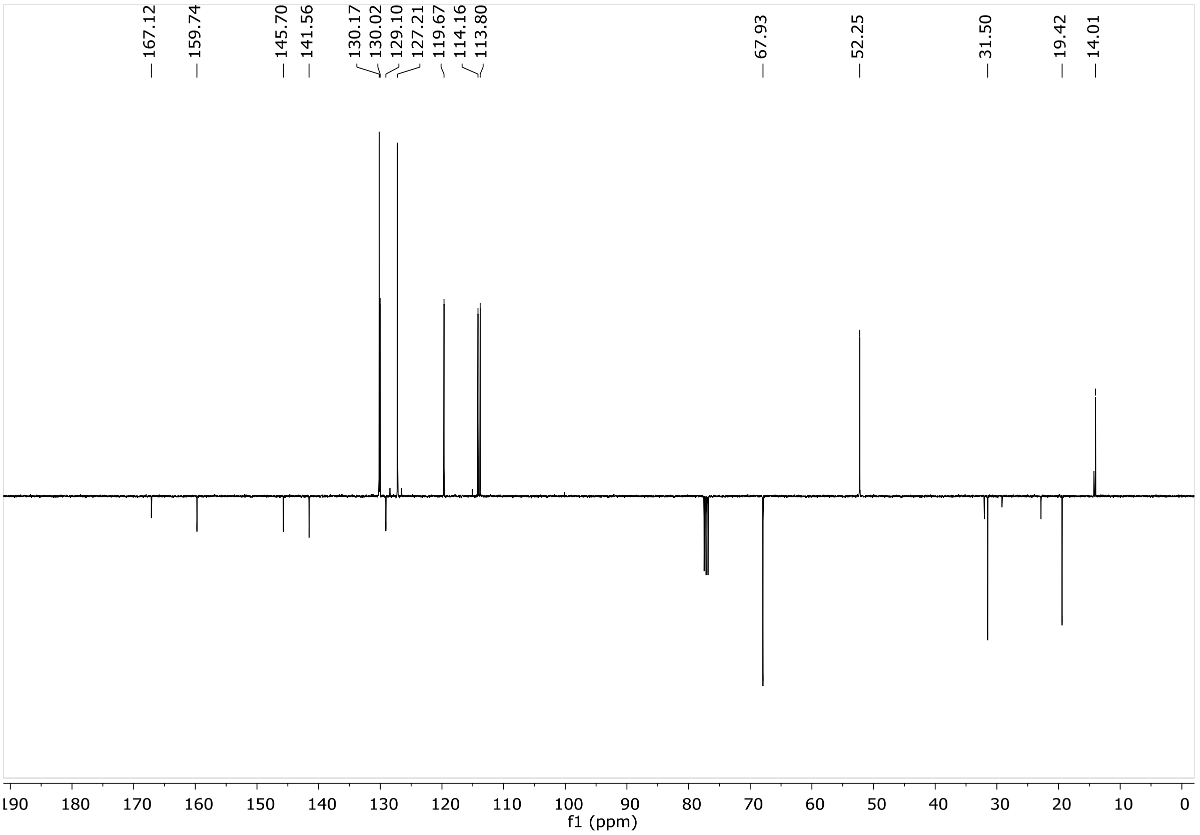
167.12 159.74 145.70 141.56 130.17 130.02 129.10 127.21 119.67 114.16 113.80 67.93 52.25 31.50 19.42 14.01
f1 (ppm)

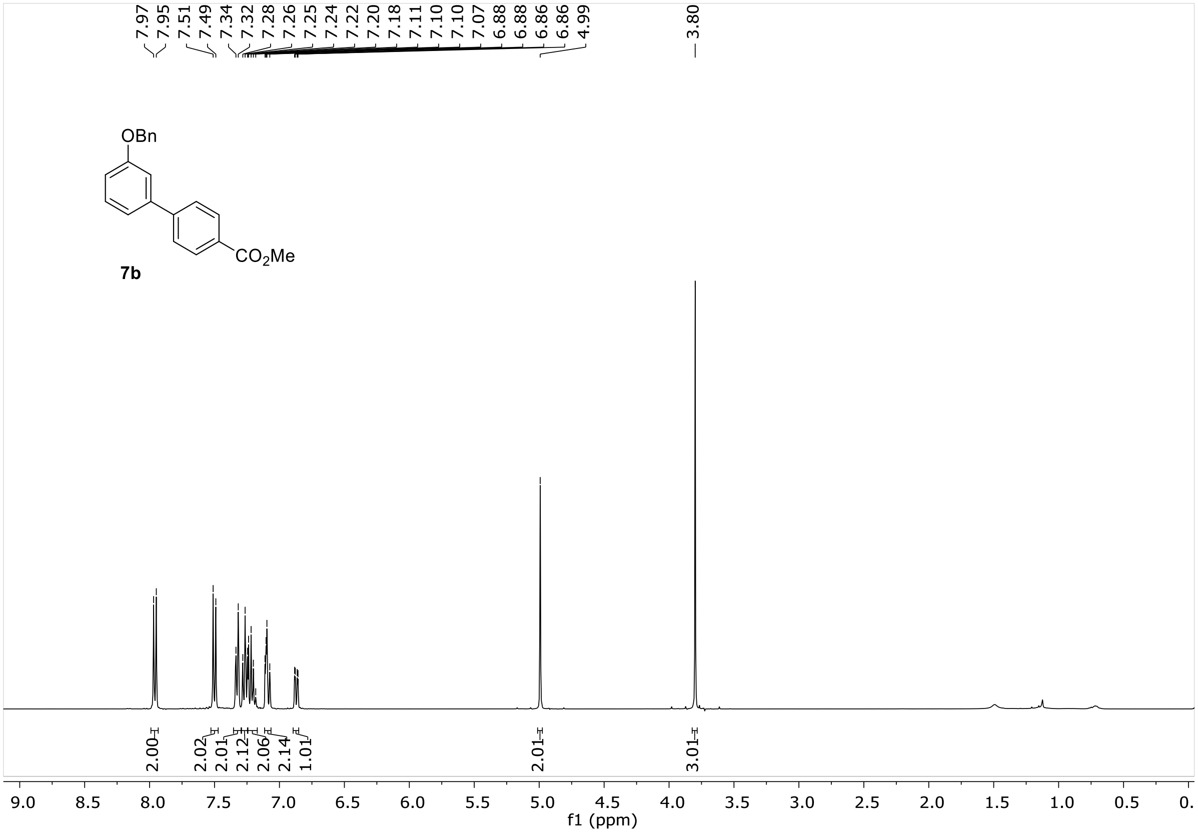
7.97
7.95
7.51
7.49
7.34
7.32
7.28
7.26
7.25
7.24
7.22
7.20
7.18
7.11
7.10
7.10
7.07
6.88
6.88
6.86
6.86
4.99
3.80
OBn
CO2Me
7b
2.00
2.02
2.01
2.12
2.06
2.14
1.01
2.01
3.01
f1 (ppm)

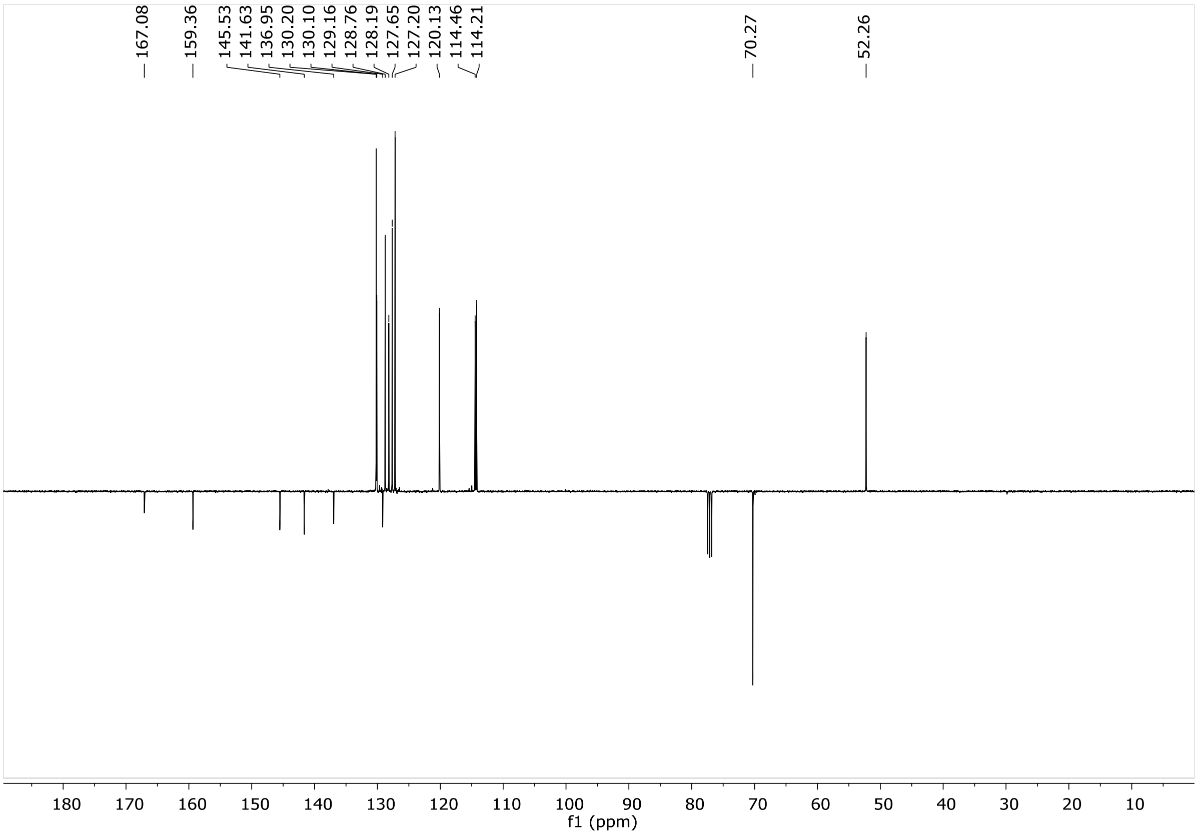
167.08
159.36
145.53
141.63
136.95
130.20
130.10
129.16
128.76
128.19
127.65
127.20
120.13
114.46
114.21
70.27
52.26
f1 (ppm)

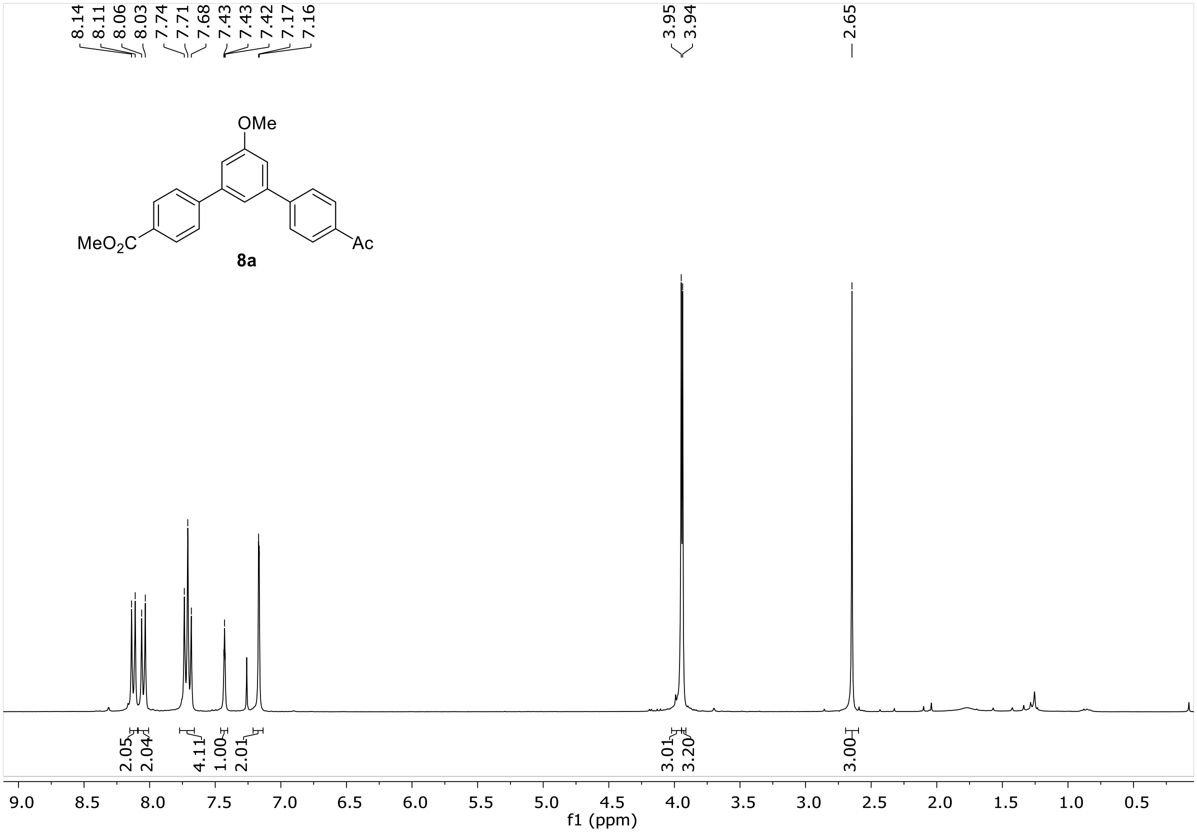
8.14
8.11
8.06
8.03
7.74
7.71
7.68
7.43
7.43
7.42
7.17
7.16
3.95
3.94
2.65
OMe
MeO2C
Ac
8a
2.05
2.04
4.11
1.00
2.01
3.01
3.20
3.00
f1 (ppm)

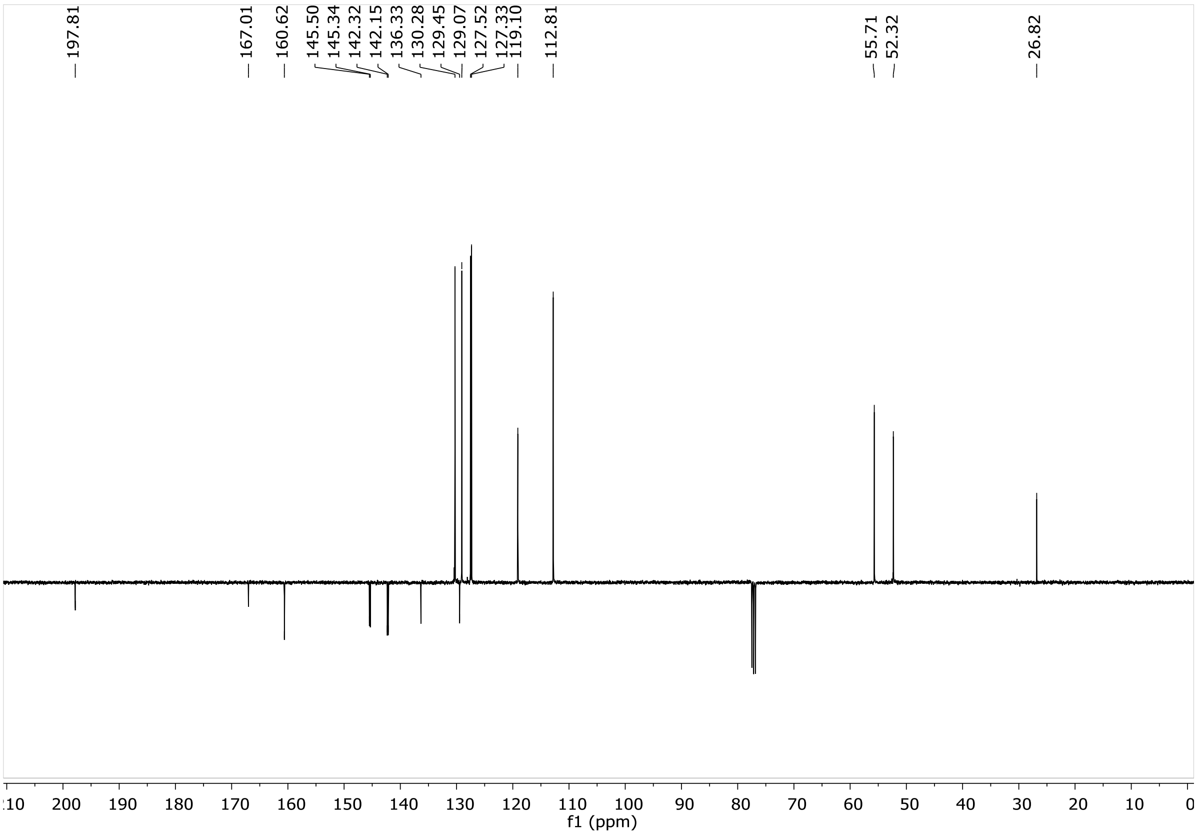
197.81
167.01
160.62
145.50
145.34
142.32
142.15
136.33
130.28
129.45
129.07
127.52
127.33
119.10
112.81
55.71
52.32
26.82
f1 (ppm)

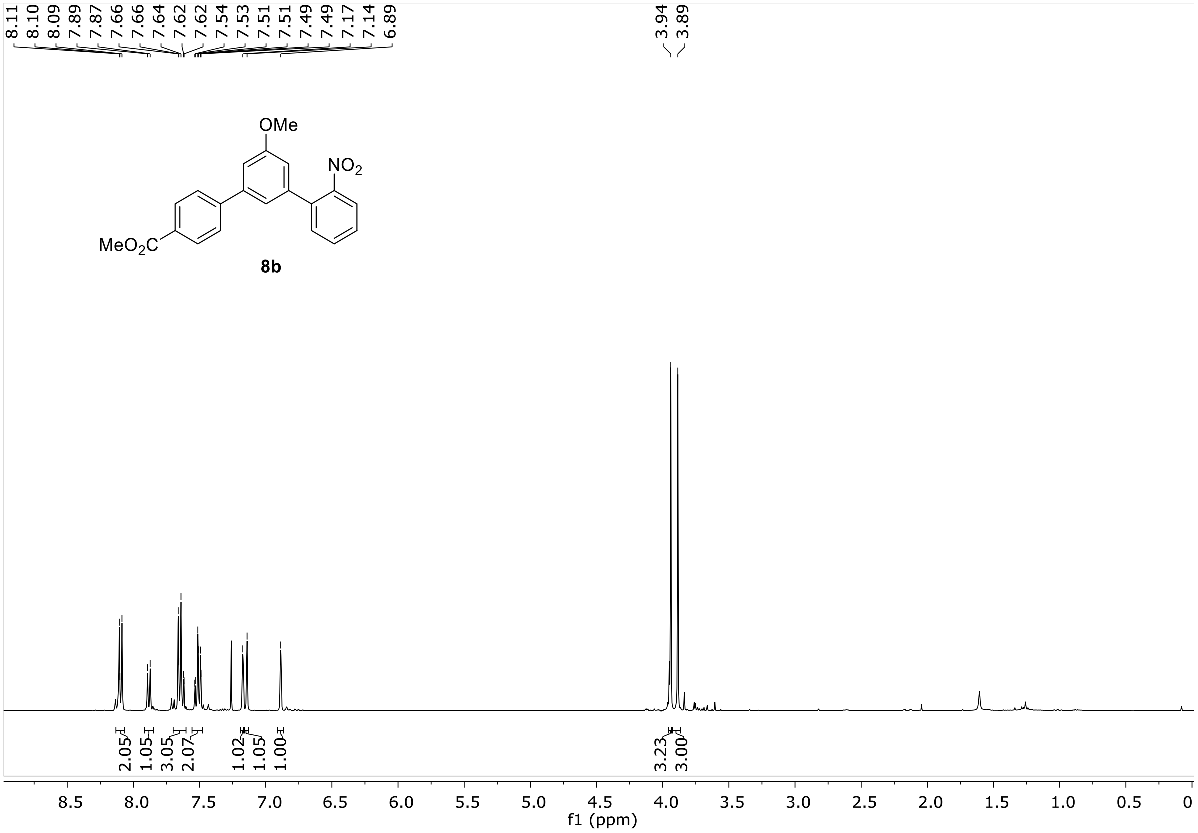
8.11
8.10
8.09
7.89
7.87
7.66
7.66
7.64
7.62
7.62
7.54
7.53
7.51
7.51
7.49
7.49
7.17
7.14
6.89
3.94
3.89
OMe
NO2
MeO2C
8b
2.05
1.05
3.05
2.07
1.02
1.05
1.00
3.23
3.00
8.5 8.0 7.5 7.0 6.5 6.0 5.5 5.0 4.5 4.0 3.5 3.0 2.5 2.0 1.5 1.0 0.5 0
f1 (ppm)

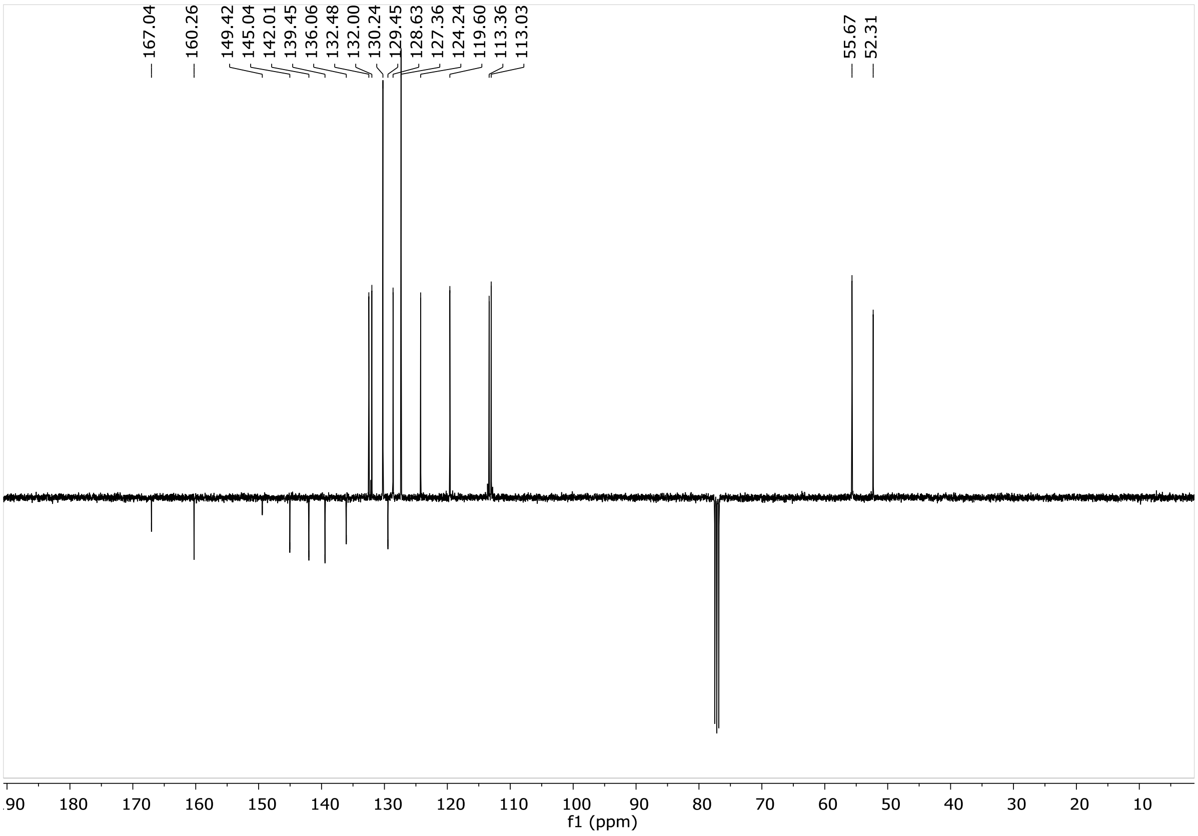
167.04
160.26
149.42
145.04
142.01
139.45
136.06
132.48
132.00
130.24
129.45
128.63
127.36
124.24
119.60
113.36
113.03
55.67
52.31
190 180 170 160 150 140 130 120 110 100 90 80 70 60 50 40 30 20 10
f1 (ppm)

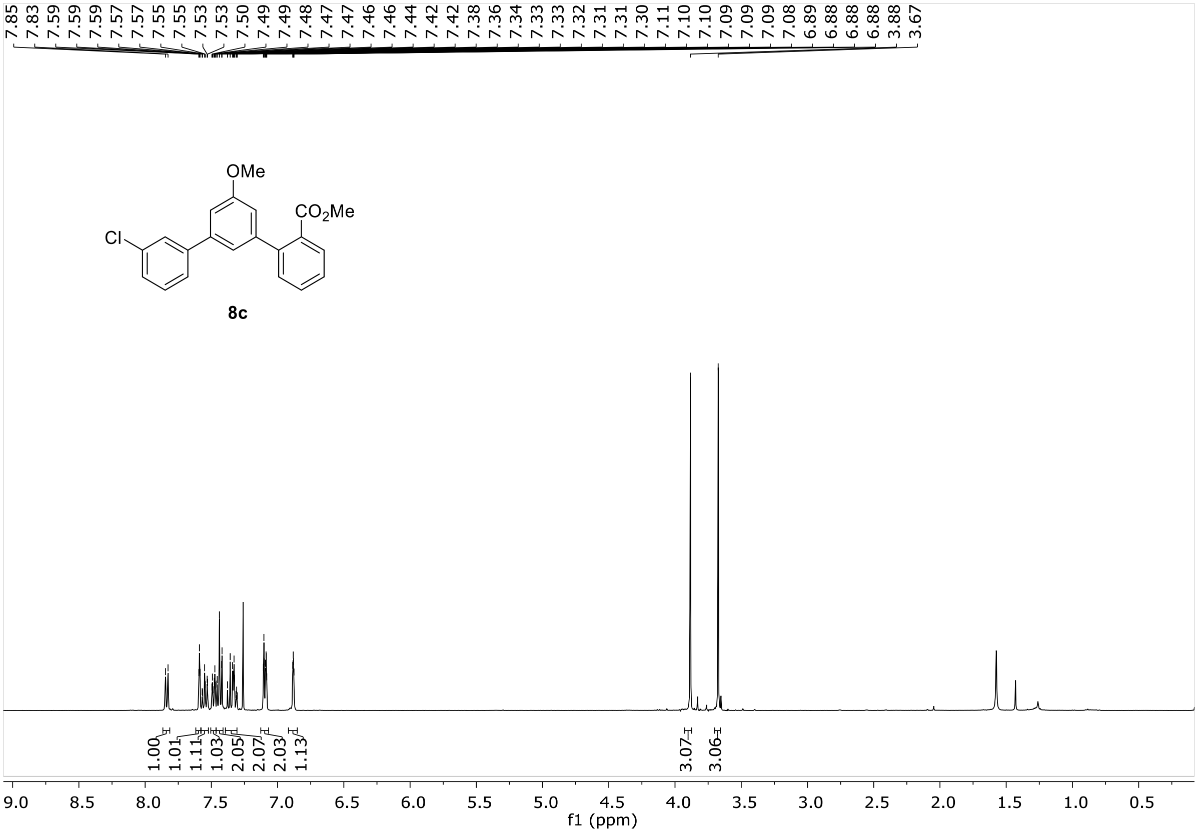
7.85 7.83 7.59 7.59 7.59 7.57 7.57 7.55 7.55 7.53 7.53 7.50 7.49 7.49 7.48 7.47 7.47 7.46 7.46 7.44 7.42 7.42 7.38 7.36 7.34 7.33 7.33 7.32 7.31 7.31 7.30 7.11 7.10 7.10 7.09 7.09 7.08 6.89 6.88 6.88 6.88 3.88 3.67
OMe
CO_2Me
Cl
8c
1.00 1.01 1.11 1.03 2.05 2.07 2.03 1.13
3.07 3.06
9.0 8.5 8.0 7.5 7.0 6.5 6.0 5.5 5.0 4.5 4.0 3.5 3.0 2.5 2.0 1.5 1.0 0.5
f1 (ppm)

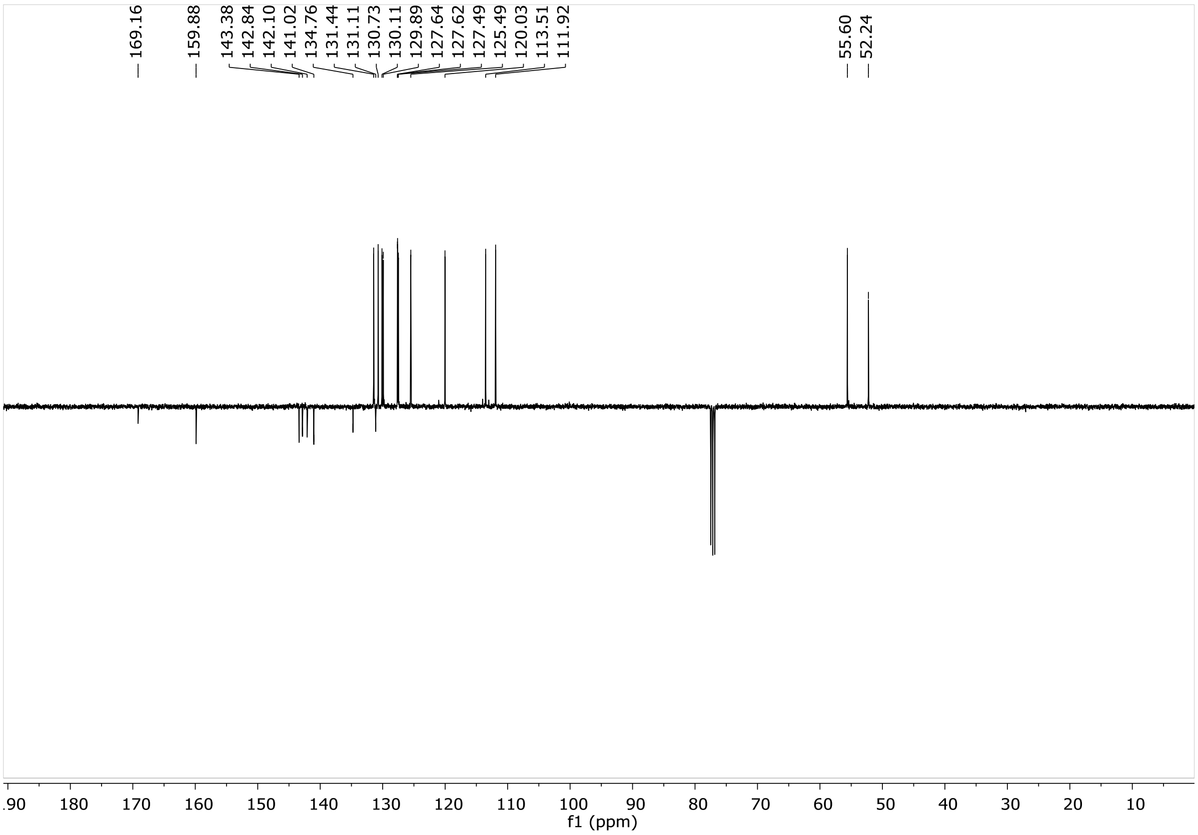
169.16
159.88
143.38 142.84 142.10 141.02 134.76 131.44 131.11 130.73 130.11 129.89 127.64 127.62 127.49 125.49 120.03 113.51 111.92
55.60
52.24
190 180 170 160 150 140 130 120 110 100 90 80 70 60 50 40 30 20 10
f1 (ppm)

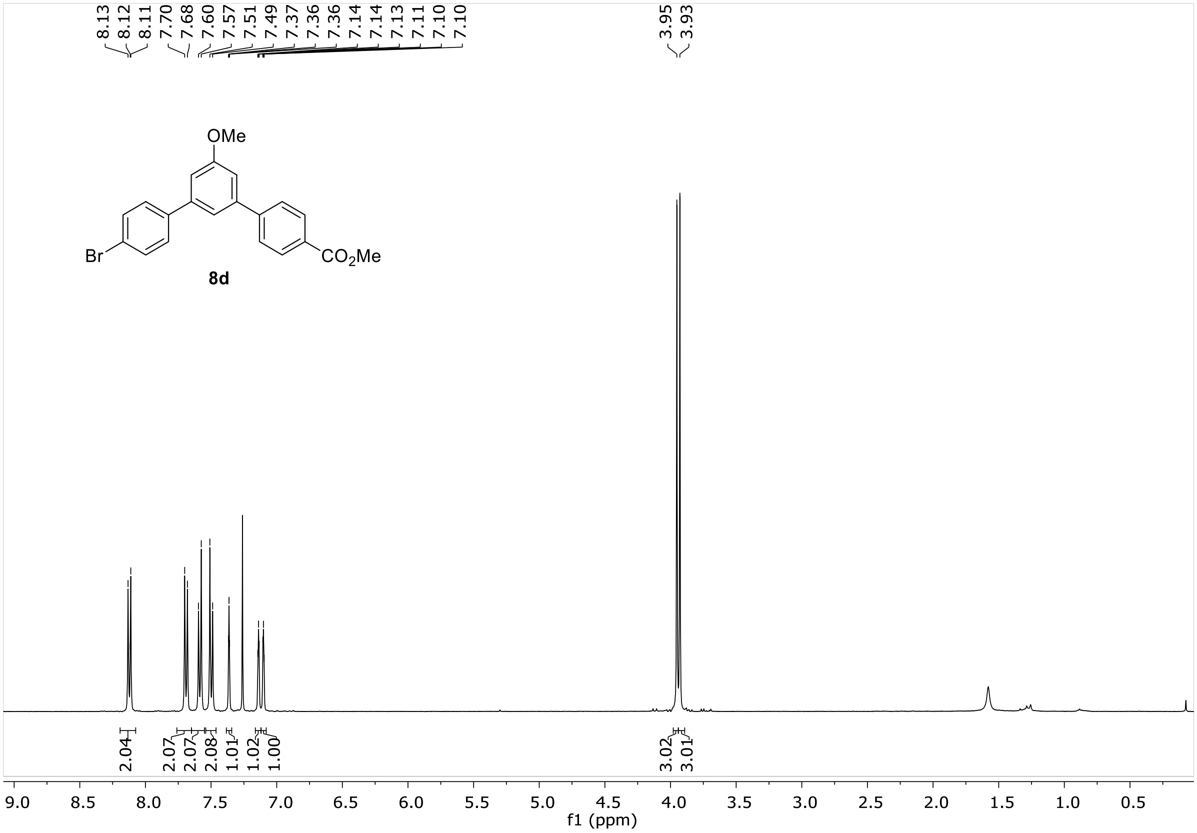
8.13
8.12
8.11
7.70
7.68
7.60
7.57
7.51
7.49
7.37
7.36
7.36
7.14
7.14
7.13
7.11
7.10
7.10
3.95
3.93
OMe
Br
CO_2Me
8d
2.04
2.07
2.07
2.08
1.01
1.02
1.00
3.02
3.01
9.0 8.5 8.0 7.5 7.0 6.5 6.0 5.5 5.0 4.5 4.0 3.5 3.0 2.5 2.0 1.5 1.0 0.5
f1 (ppm)

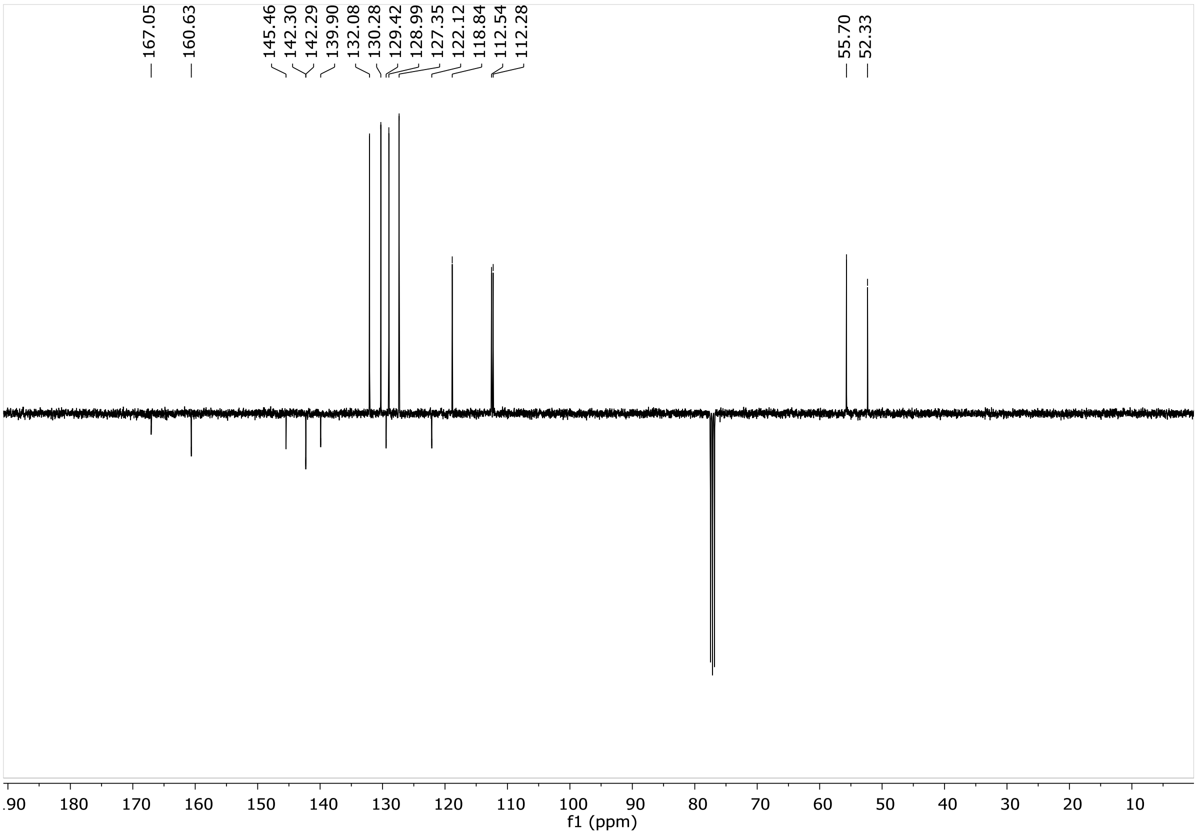
167.05
160.63
145.46
142.30
142.29
139.90
132.08
130.28
129.42
128.99
127.35
122.12
118.84
112.54
112.28
55.70
52.33
190 180 170 160 150 140 130 120 110 100 90 80 70 60 50 40 30 20 10
f1 (ppm)

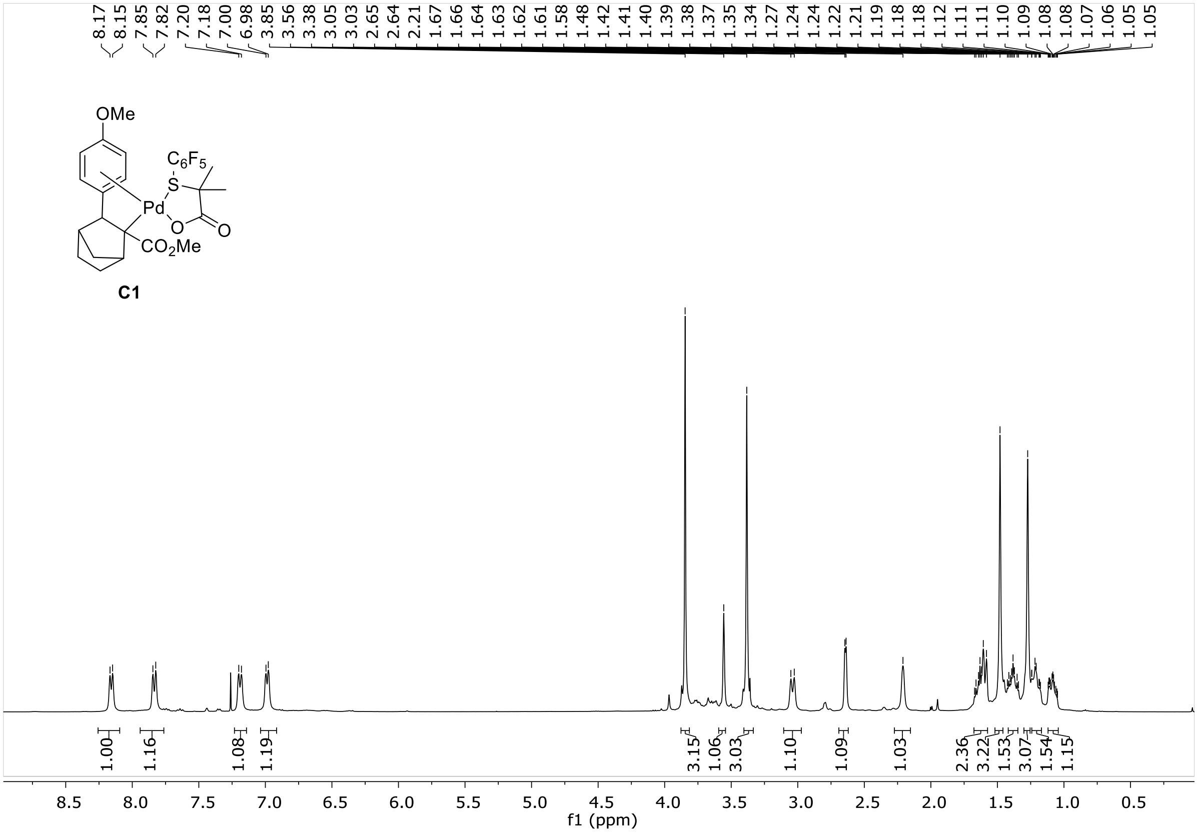
8.17
8.15
7.85
7.82
7.20
7.18
7.00
6.98
3.85
3.56
3.38
3.05
3.03
2.65
2.64
2.21
1.67
1.66
1.64
1.63
1.62
1.61
1.58
1.48
1.42
1.41
1.40
1.39
1.38
1.37
1.35
1.34
1.27
1.24
1.24
1.22
1.21
1.19
1.18
1.18
1.12
1.11
1.11
1.10
1.09
1.08
1.08
1.07
1.06
1.05
1.05
OMe
C6F5
S
Pd
O
O
CO2Me
C1
1.00
1.16
1.08
1.19
3.15
1.06
3.03
1.10
1.09
1.03
2.36
3.22
1.53
3.07
1.54
1.15
f1 (ppm)

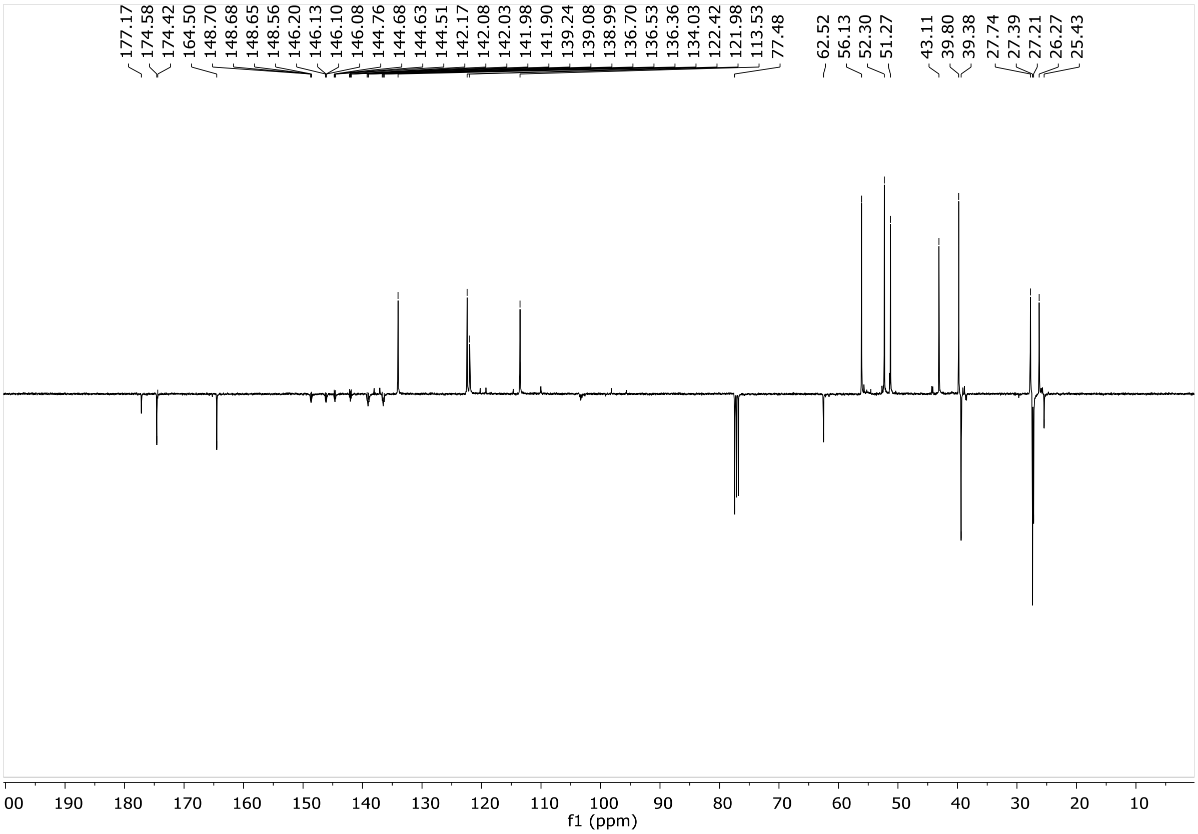
177.17
174.58
174.42
164.50
148.70
148.68
148.65
148.56
146.20
146.13
146.10
146.08
144.76
144.68
144.63
144.51
142.17
142.08
142.03
141.98
141.90
139.24
139.08
138.99
136.70
136.53
136.36
134.03
122.42
121.98
113.53
77.48
62.52
56.13
52.30
51.27
43.11
39.80
39.38
27.74
27.39
27.21
26.27
25.43
f1 (ppm)

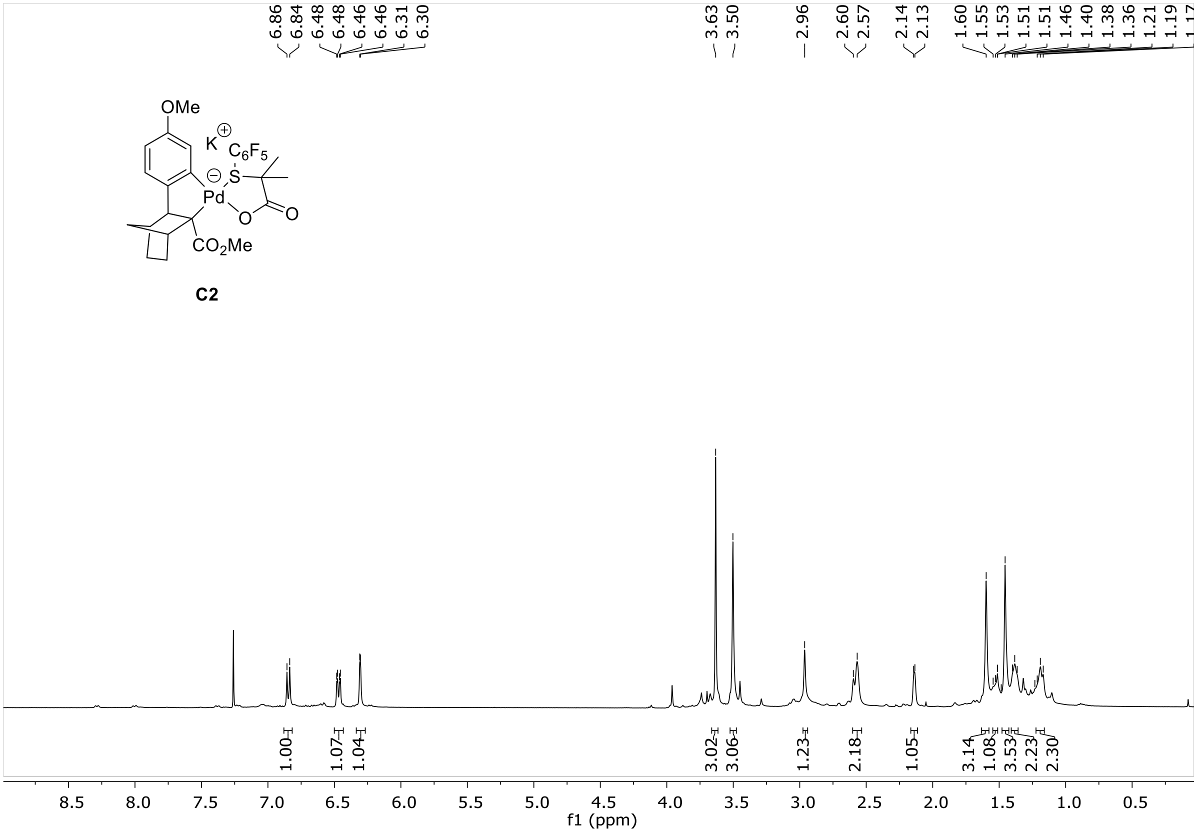
6.86
6.84
6.48
6.48
6.46
6.46
6.31
6.30
3.63
3.50
2.96
2.60
2.57
2.14
2.13
1.60
1.55
1.53
1.51
1.51
1.46
1.40
1.38
1.36
1.21
1.19
OMe
K
C6F5
S
Pd
O
O
CO2Me
C2
1.00
1.07
1.04
3.02
3.06
1.23
2.18
1.05
3.14
1.08
3.53
2.23
2.30
f1 (ppm)

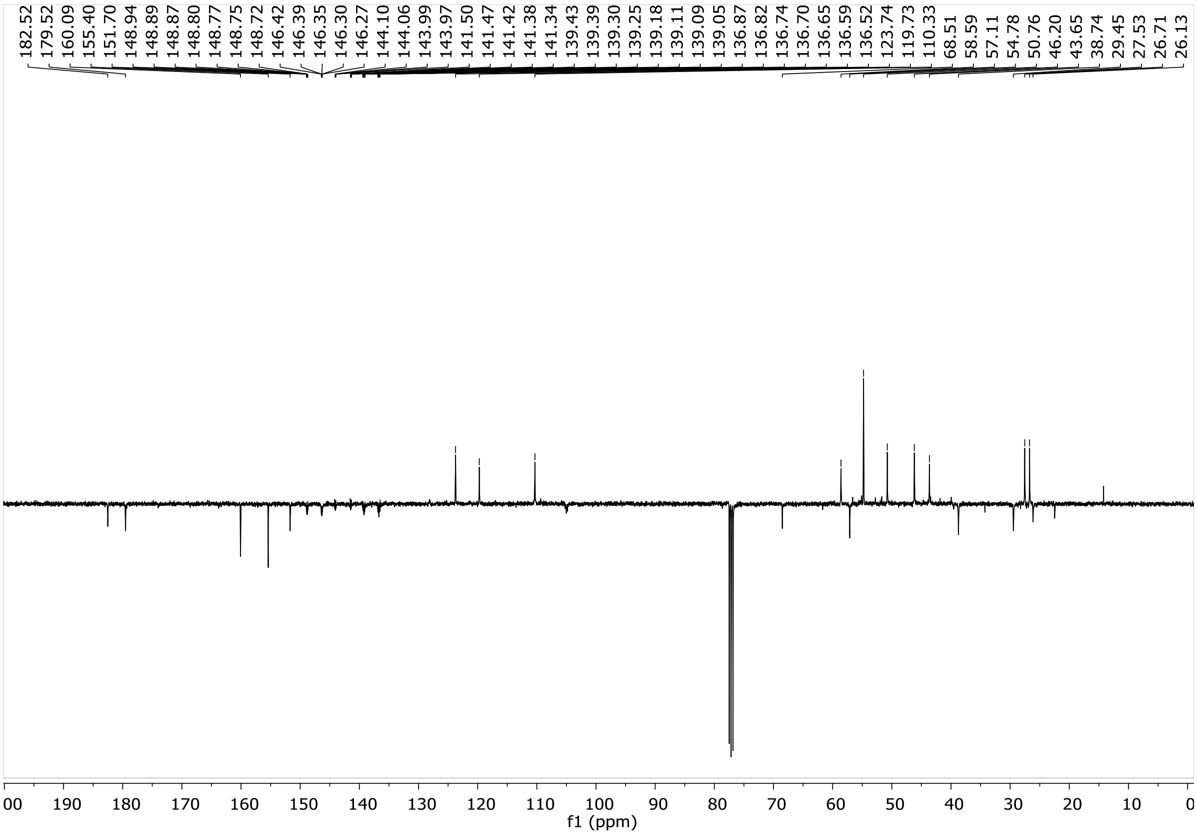
182.52
179.52
160.09
155.40
151.70
148.94
148.89
148.87
148.80
148.77
148.75
148.72
146.42
146.39
146.35
146.30
146.27
144.10
144.06
143.99
143.97
141.50
141.47
141.42
141.38
141.34
139.43
139.39
139.30
139.25
139.18
139.11
139.09
139.05
136.87
136.82
136.74
136.70
136.65
136.59
136.52
123.74
119.73
110.33
68.51
58.59
57.11
54.78
50.76
46.20
43.65
38.74
29.45
27.53
26.71
26.13
f1 (ppm)

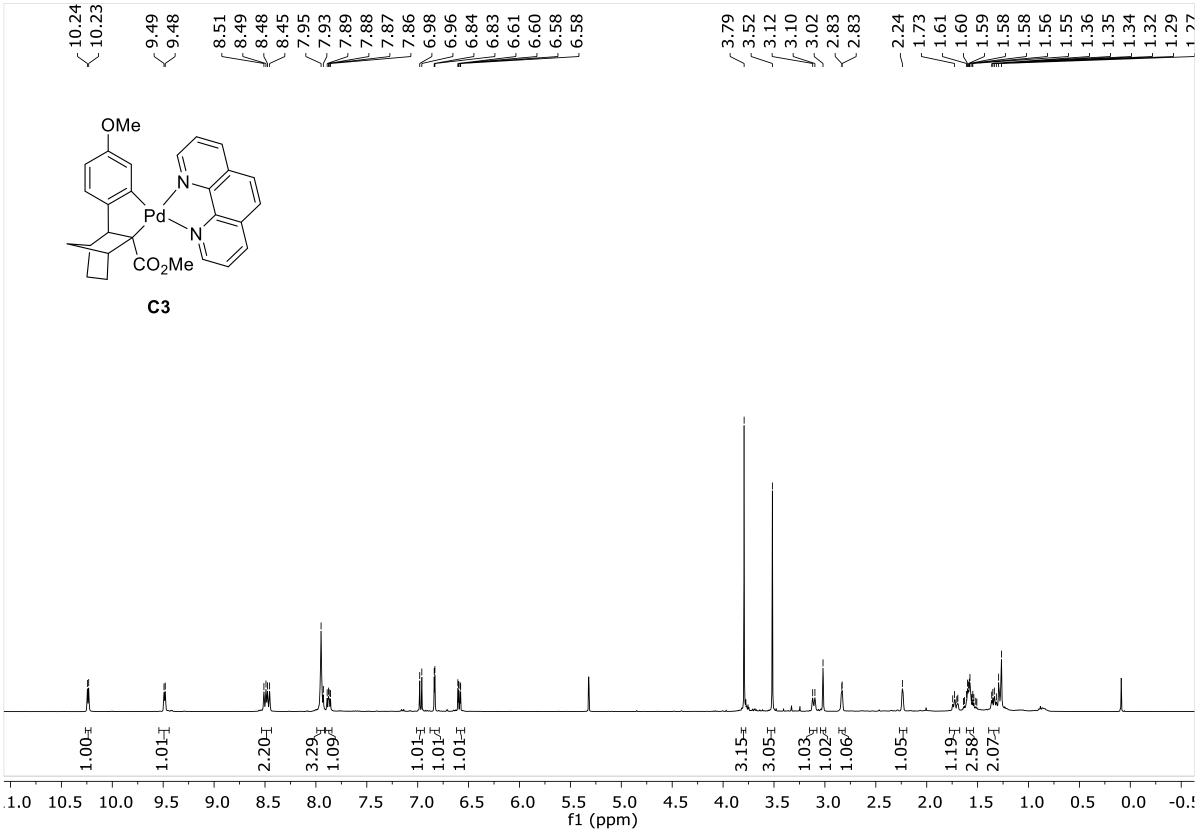
10.24
10.23
9.49
9.48
8.51
8.49
8.48
8.45
7.95
7.93
7.89
7.88
7.87
7.86
6.98
6.96
6.84
6.83
6.61
6.60
6.58
6.58
3.79
3.52
3.12
3.10
3.02
2.83
2.83
2.24
1.73
1.61
1.60
1.59
1.58
1.58
1.56
1.55
1.36
1.35
1.34
1.32
1.29
[illegible]
OMe
N
Pd
N
CO_2Me
C3
1.00
1.01
2.20
3.29
1.09
1.01
1.01
1.01
3.15
3.05
1.03
1.02
1.06
1.05
1.19
2.58
2.07
f1 (ppm)

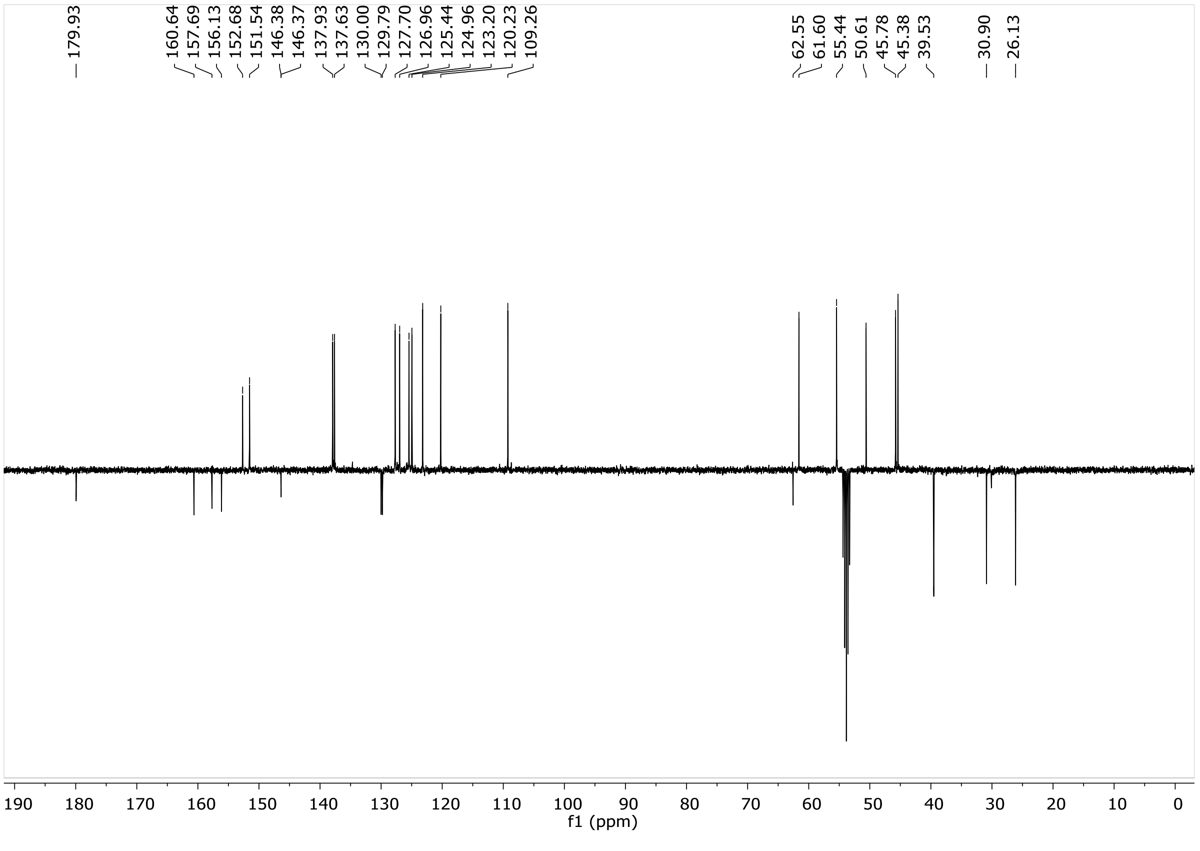
179.93
160.64
157.69
156.13
152.68
151.54
146.38
146.37
137.93
137.63
130.00
129.79
127.70
126.96
125.44
124.96
123.20
120.23
109.26
62.55
61.60
55.44
50.61
45.78
45.38
39.53
30.90
26.13
f1 (ppm)

4
